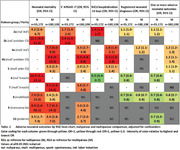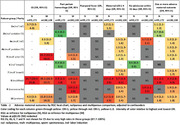# Poster Session 3: Friday, January 31, 2025 10:30 AM – 12:30 PM

**DOI:** 10.1002/pmf2.12028

**Published:** 2025-01-05

## 666 | Diagnostic Performance of Three Methods of Fetal Growth Restriction Diagnosis

Abby R. Rubenstein^1^; Stephanie L. Pierce^1^; Morgan McDougal^1^; Jennifer Peck^1^; Erin Schone^1^; Angela Xing^1^; Matthew Harter^1^; Rachel Jillson^1^; Dakota St Pierre^1^; Rodney Edwards^2^

*^1^University of Oklahoma Health Sciences Center, Oklahoma City, OK; ^2^University of Florida College of Medicine, Gainesville, FL*

10:30 AM ‐ 12:30 PM

**Objective**: Recent guidelines changed the diagnostic criteria for fetal growth restriction (FGR) from estimated fetal weight (EFW) < 10%ile alone to EFW and/or abdominal circumference (AC) < 10%ile. Prior studies suggested this change would not substantially affect the number of pregnancies diagnosed with FGR. This study compares 3 methods of FGR diagnosis.

**Study Design**: This retrospective cohort study classified three groups of women delivering at our institution based on timing of how we diagnosed FGR: Group 1 (7/1/17‐6/30/18; EFW < 10%ile), Group 2 (4/1/20‐3/31/21; EFW < 10%ile or EFW 10‐19%ile and AC < 5%ile), and Group 3 (8/1/21‐7/31/22; EFW and/or AC < 10%ile). Multifetal gestations and fetuses with major anomalies were excluded. Cases of neonatal small for gestational age (SGA) were also identified in each time period. The primary outcome was prevalence of FGR among all deliveries in each time period. Test performance characteristics of the three methods of FGR diagnosis for predicting SGA were evaluated.

**Results**: 320 pregnancies with FGR were identified (n = 44 Group 1, n = 96 Group 2, n = 180 Group 3). Maternal characteristics were similar between groups (Table 1). For groups 1, 2 and 3 respectively, the prevalence of FGR was 1.16%, 2.74% and 4.82% (p< 0.0001), and the prevalence of SGA was 3.39%, 4.44% and 4.53% (p = 0.02). Test performance characteristics of each approach are shown in Table 2.

**Conclusion**: Application of the newest diagnostic criteria for FGR resulted in a quadrupling of the rate of FGR diagnosis. Though the prevalence of FGR diagnosis with the newest criteria was similar to the prevalence of SGA, this approach identified less than half of neonates with SGA. Given this substantial increase in diagnoses, most of which are false positives, further investigation regarding resource utilization and neonatal outcomes is warranted.

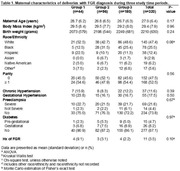

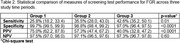

## 667 | Resource Utilization and Neonatal Outcomes with Three Methods of Fetal Growth Restriction Diagnosis

Abby R. Rubenstein^1^; Stephanie L. Pierce^1^; Morgan McDougal^1^; Jennifer Peck^1^; Erin Schone^1^; Angela Xing^1^; Matthew Harter^1^; Rachel Jillson^1^; Dakota St Pierre^1^; Rodney Edwards^2^

*^1^University of Oklahoma Health Sciences Center, Oklahoma City, OK; ^2^University of Florida College of Medicine, Gainesville, FL*

10:30 AM ‐ 12:30 PM

**Objective**: Fetal growth restriction (FGR) is a known cause of neonatal morbidity. Recent new guidelines changed the diagnostic criteria from estimated fetal weight (EFW) < 10%ile alone to EFW and/or abdominal circumference (AC) < 10%ile. Diagnosing and treating FGR is important to optimize fetal and neonatal outcomes, however does require increased antenatal surveillance. This study compares neonatal outcomes across 3 methods of FGR diagnosis.

**Study Design**: This secondary analysis of a retrospective cohort study classified three groups of women who delivered at our institution based on timing of how we diagnosed FGR: Group 1 (7/1/2017‐6/30/2018; EFW < 10%ile), Group 2 (4/1/2020‐3/31/2021; EFW < 10%ile or EFW 10‐19%ile and AC < 5%ile), and Group 3 (8/1/2021‐7/31/2022; EFW and/or AC < 10%ile). Inclusion criteria were FGR diagnosis and dating ultrasound (US) prior to 22 weeks. Multifetal gestations and fetuses with major anomalies were excluded. The primary outcome was a composite of neonatal complications. Continuous outcome variables were compared across the three groups using ANOVA. Categorical outcomes variables were compared using X^2^. P‐value < 0.05 was statistically significant.

**Results**: 320 pregnancies with FGR diagnosis were identified (n = 44 Group 1, n = 96 Group 2, n = 180 Group 3). Overall, gestational age at delivery was similar between groups (Table 1). Compared to Group 1, numbers of growth US and BPP were higher in Group 2 and highest in Group 3. The neonatal complications composite rate was 56.8% in Group 1, 45.8% in Group 2, and 37.2% in Group 3 (p = 0.046; Table 2). NICU admission rate was 54.6% in Group 1, 41.1% in Group 2, and 34.1% in Group 3 (p = 0.04).

**Conclusion**: The newest method of FGR diagnosis resulted in the highest numbers of prenatal growth US and BPP. The composite complication rate for neonates with prenatal FGR diagnosis was lower during the time period using the new FGR criteria, however this may be due to the inclusion of neonates with less severe FGR and/or absence of SGA. Further investigation is needed into whether broader definitions of FGR result in improved neonatal outcomes.

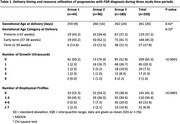

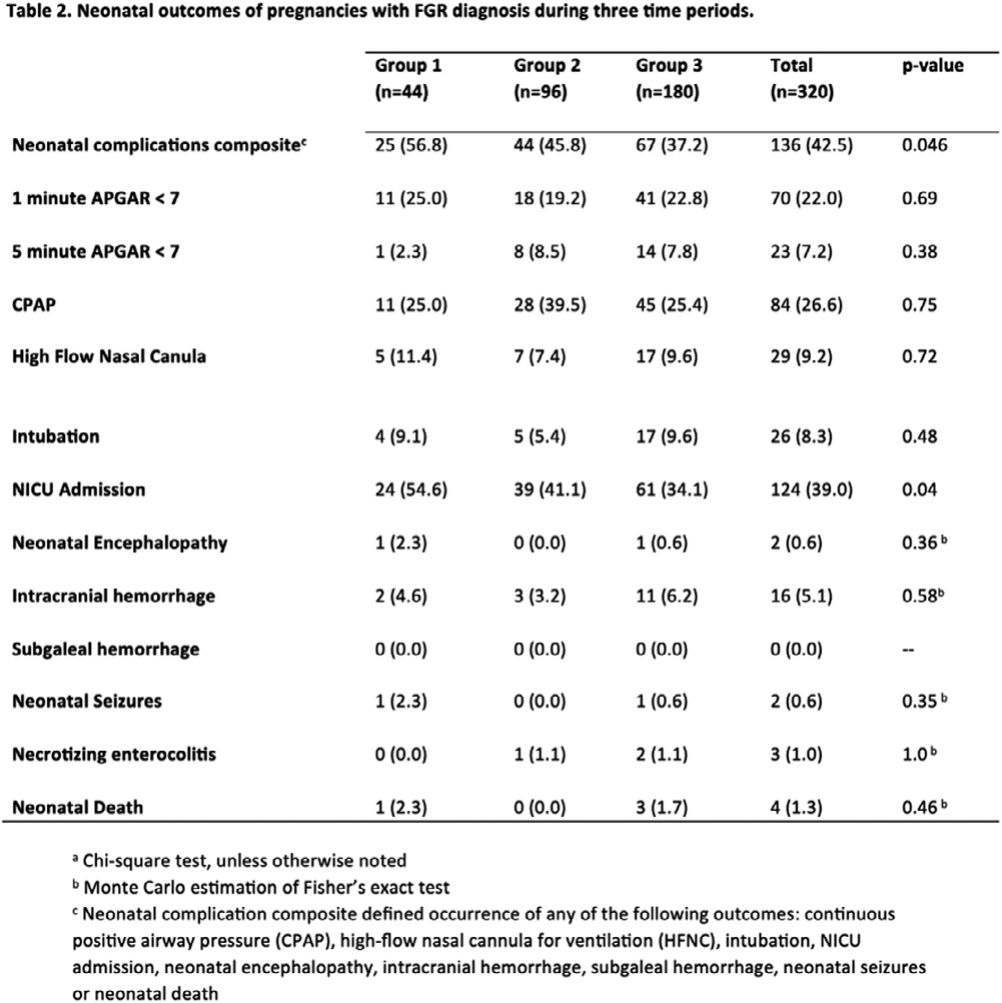

## 668 | Wernicke'S Encephalopathy in Pregnancy: a Systematic Review of Pregnancy and Neonatal Outcomes

Abirami Kirubarajan^1^; Julia Hollingsworth^1^; Misgav Rottenstreich^1^; Katherine Steckham^1^; Conor Cox^1^; Bryon DeFrance^2^; Jon F. Barrett^2^; Eran Ashwal^2^

*^1^McMaster University, Hamilton, ON; ^2^McMaster university, Hamilton, ON*

10:30 AM ‐ 12:30 PM

**Objective**: To assess and report pregnancy and neonatal outcomes related to Wernicke's encephalopathy in pregnancy.

**Study Design**: We conducted a systematic review of six databases (MEDLINE, EMBASE, Emcare, the Cochrane Library, Web of Science: Core Collection, and Scopus). Articles from January 1^st^, 2000 until June 1^st^, 2024 were included.

We included original observational articles that reported neonatal or fetal outcomes following a diagnosis of Wernicke's encephalopathy in the context of a live intrauterine pregnancy.

Descriptive statistics were used to aggregate results, as well as a nonparametric chi‐square test and ANOVA. The Oxford Center of Evidence‐Based Medicine grading tool was used to assess quality of evidence

**Results**: After screening 1005 references, a total of 65 studies encompassing 88 patients were eligible for analysis, all of which were case series or case reports. The mean gestational age of Wernicke's diagnosis was 16 ± 4.4 weeks. The majority of patients (55/88, 62.5%) had a live delivery, with 35 (63.6%) being reported as term delivery. Mean birthweight was 2523.64 grams. A total of 29 (32.9%) patients experienced pregnancy loss, with the majority (22/29, 75.9%) occurring before 20 weeks of gestation. Pregnancy loss was significantly associated with severe Wernicke's disease (p = 0.006), as well as delayed diagnosis (p = 0.02). A total of 40 studies reported the mode of delivery, with 22 (55%) involving Cesarean deliveries and 18 (45%) involving vaginal deliveries. There was limited data available regarding neonatal outcomes, especially regarding long‐term follow‐up. Among the 54 live births, 35 (64.8%) were described simply as “healthy” or “eutrophic”. Seven cases specifically noted no neonatal neurological concerns at birth.

**Conclusion**: Although evidence regarding Wernicke's encephalopathy in pregnancy is limited, it is crucial to counsel patients about the increased risk of pregnancy loss associated with severe Wernicke's disease. Prompt diagnosis and treatment are essential, as delayed diagnosis is also more likely to result in pregnancy loss.

## 669 | Evaluating the Impact of a Novel Shared Medical Appointment Model for Gestational Diabetes Care

Adina R. Kern‐Goldberger^1^; Easha Patel^2^; Maeve Hopkins^2^; Cara D. Dolin^2^; Stacey Ehrenberg^2^

*^1^Cleveland Clinic Lerner College of Medicine, Cleveland, OH; ^2^Cleveland Clinic Foundation, Cleveland, OH*

10:30 AM ‐ 12:30 PM

**Objective**: Optimizing care delivery for gestational diabetes (GDM) has the potential to improve maternal and fetal/neonatal outcomes. The Shared Medical Appointment (SMA) has emerged as a novel tool to improve access as well as patient engagement and satisfaction by providing group care for patients sharing a common diagnosis. This study evaluated the impact of introducing an SMA model for GDM care.

**Study Design**: This a retrospective cohort study with a pre/post design at 2 hospitals in a single health system. All patients with a diagnosis of GDM who delivered >20 weeks from 1/1/2018‐12/31/2022 were included. The SMA model was initiated 1/1/2021 with 8 patient slots/week, capturing 45% of the GDM patient population. Prior to this, all patients with GDM had traditional MFM consults. Patient demographic and clinical data were compared before and after initiation of the SMA. The primary outcome was a composite of GDM‐related adverse obstetric outcomes including fetal demise, macrosomia (> 4000g), NTSV cesarean delivery, and NICU admission. Multivariable logistic regression evaluated the primary outcome before and after instituting the GDM SMA. Yearly incidence of the primary outcome was evaluated for the entire obstetric population during this period to assess for a secular trend.

**Results**: 2,309 patients with GDM were included (4.8% of all delivered patients during the study period) with 821 cases of the primary outcome (35.6%). Patient characteristics pre/post introduction of the SMA are depicted in Table 1. Significant differences were noted in maternal age and OBCMI (higher post‐SMA) and nulliparity and pregravid BMI (higher pre‐SMA). On adjusted analysis, there was a lower adjusted odds ratio (aOR) for the primary outcome (0.826, 95% CI 0.685‐0.995, p = 0.044) with the SMA, driven by decreased odds of NICU admission (Table 2). There was no evidence of a secular trend in the primary outcome.

**Conclusion**: Innovations in care delivery for pregnancy complications like GDM have potential to improve obstetric outcomes. Further research can clarify implementation strategies and generalizability of this approach.

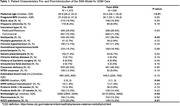

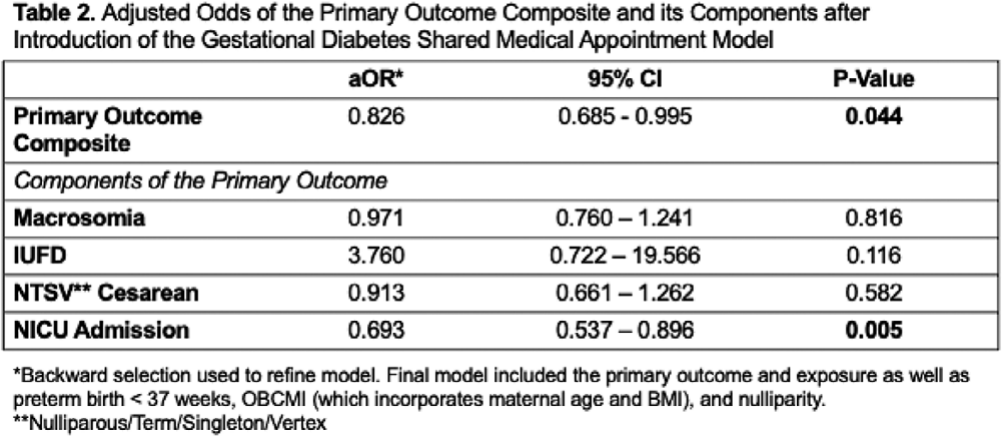

## 670 | Predictors of Successful Induction of Labor in Patients with Class 3 Obesity

Adina R. Kern‐Goldberger^1^; Easha Patel^2^; Kirat Sandhu^2^; Stacey Ehrenberg^2^; Cara D. Dolin^2^; Maeve Hopkins^2^

*^1^Cleveland Clinic Lerner College of Medicine, Cleveland, OH; ^2^Cleveland Clinic Foundation, Cleveland, OH*

10:30 AM ‐ 12:30 PM

**Objective**: Maternal obesity is a known risk factor for cesarean delivery, but risk may be differential based on obesity severity. This study explores predictors of successful vaginal delivery in patients with class 3 obesity undergoing induction of labor.

**Study Design**: This is a retrospective cohort study of singleton deliveries with BMI > 40 following induction of labor > 20 weeks at a multi‐hospital academic health system from 1/1/2022‐6/30/2024. Patients with prior cesarean were excluded. The primary outcome was vaginal delivery. Patient clinical data were extracted from the medical record and demographics, clinical risk factors, and outcomes were compared in univariable analyses by mode of delivery. Based on identified significance in these analyses, a multivariable logistic regression model was utilized to evaluate the impact of potential predictors on adjusted likelihood of vaginal delivery.

**Results**: 610 total patients were included, 457 of whom delivered vaginally (74.9%). Patients who delivered vaginally had lower pregravid BMI (43.6 v. 44.5, p = 0.01) and lower delivery BMI (46.3 v. 49.4, p < 0.01) [Table 1]. Patients with Medicaid compared to commercial insurance were more likely to delivery vaginally, as were patients who were not nulliparous, did not require cervical ripening at induction, and did not have severe preeclampsia. Patients who delivered by cesarean had higher rates of NICU admission and extended postpartum length of stay, as well as blood loss. Nulliparity was identified as the most significant negative predictor of vaginal delivery on adjusted analysis (aOR 0.01, 95% CI 0.06‐0.18, p < 0.01) [Table 2]. Other significant negative predictors were higher delivery BMI, cervical ripening, and chronic hypertension.

**Conclusion**: Nulliparous patients with class 3 obesity who require cervical ripening have significantly lower odds of vaginal delivery. These data can help guide counseling and physician decision making surrounding optimizing induction of labor for this patient group.

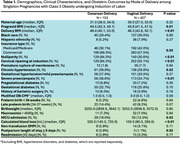

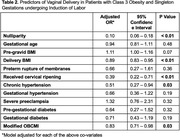

## 671 | Neonatal Outcomes in Fetuses with Resolved Growth Restriction

Aleksandra Polic; Trey McGonigle; Shi Huang; Catherine Phillips; Matthew Grace

*Vanderbilt University Medical Center, Nashville, TN*

10:30 AM ‐ 12:30 PM

**Objective**: Fetal growth restriction (FGR) is associated with neonatal morbidity and mortality, but 17% of fetuses with FGR will return to normal growth prior to delivery (resolved FGR, rFGR). Neonatal outcomes of this group have not been studied, and insufficient data exist to guide management. The aim of this study was to compare neonatal outcomes of pregnancies complicated by rFGR to pregnancies with no history of FGR.

**Study Design**: This was a retrospective cohort study of live, term singleton deliveries without known or suspected structural or genetic abnormalities from 2018‐2023 at a tertiary medical center. The exposed cohort had an ultrasound diagnosis of FGR (either by AC or EFW < 10^th^ percentile) followed by resolution of FGR on a subsequent ultrasound preceding delivery. Maternal demographics, clinical characteristics and delivery outcomes were compared by FGR status (no FGR, FGR and rFGR) at the time of delivery. We fit separate logistic regression models to estimate the association between rFGR and NICU admission, hypoxic ischemic encephalopathy (HIE), and neonatal death, adjusting for confounders. Birth weight, length of hospital stay, and APGAR scores were modeled using linear and proportional odds ordinal regression models.

**Results**: A total of 11,957 pregnancies were analyzed, of which 393 were complicated by rFGR. No differences were observed between rFGR and no FGR patients in rates of hypertensive disorders, diabetes, gestational age at delivery, or mode of delivery (Table 1). Compared to no FGR, birth weights in the rFGR cohort were lower by 366g (95% CI ‐422.37, ‐310.39; p< 0.001). No other significant effects of rFGR were observed in any of the neonatal outcomes assessed (Table 2). Although not statistically significant, the direction of the effect suggests that neonates with rFGR (vs. no FGR) may have increased odds of NICU admission, HIE, neonatal death, and longer duration of hospital stay.

**Conclusion**: When compared to pregnancies not complicated by FGR, the diagnosis of rFGR did not increase the risk of adverse neonatal outcomes in this cohort.

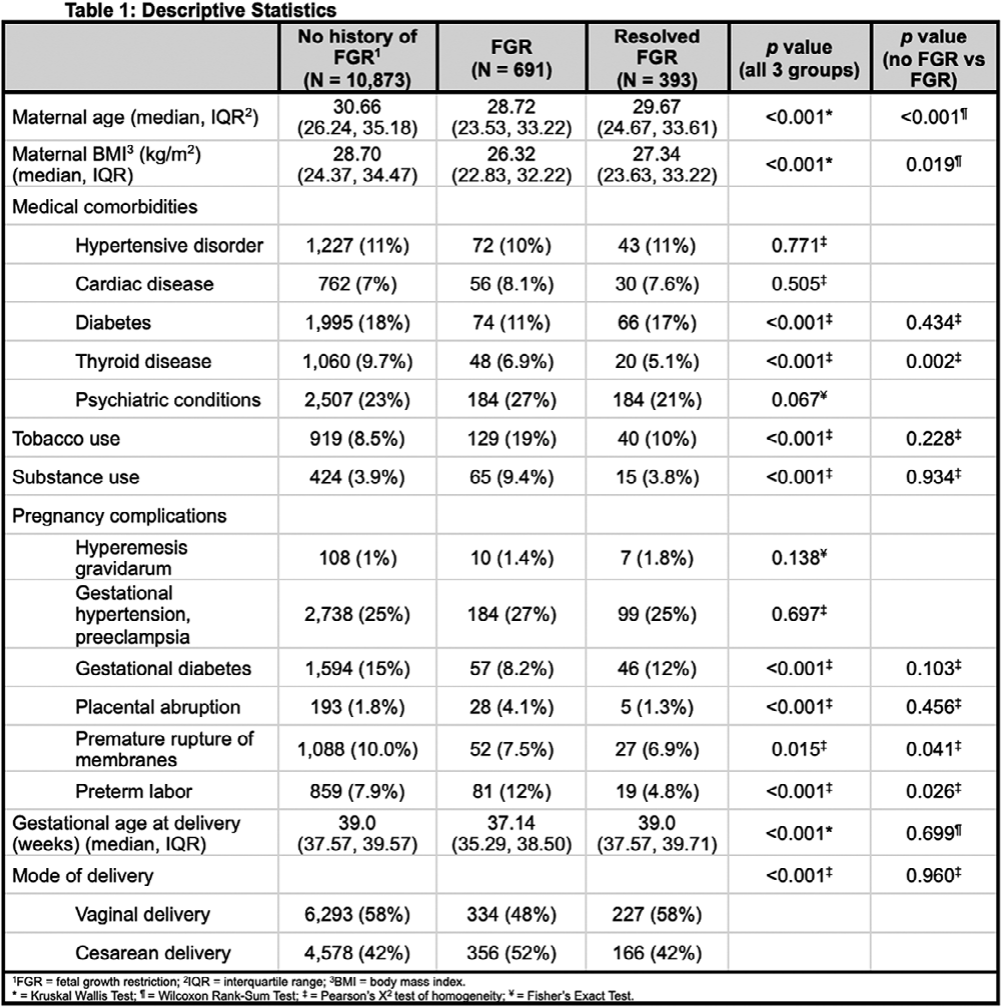

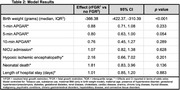

## 672 | Mid‐Trimester Rupture of the Membranes:Irish Perspective from a Tertiary Unit Following Liberalisation of Top Legislation

Alex K L Taylor^1^; Ruta Petkute^2^; Hayley Jackson^2^; Sorcha Lynch^2^; Silvia Carlotta Rosalia^3^; Jack Diviney^4^; Gillian Corbett^5^; Helena C. Bartels^6^; Siobhan Corcoran^7^

*^1^Royal College of Physicians Ireland, National Maternity Hospital, Dublin; ^2^National Maternity Hospital, National Maternity Hospital, Dublin; ^3^University of Insubria, Varese, Lombardia; ^4^Royal College of Surgeons in Ireland, Dublin, Dublin; ^5^UCD Perinatal Research Centre, Dublin, Dublin; ^6^University College Dublin, University College Dublin, Dublin; ^7^National Maternity Hospital, Dublin, Dublin*

10:30 AM ‐ 12:30 PM

**Objective**: Midtrimester pre‐mature pre‐labour rupture of membranes(MTPPROM) between 14+0‐23+6 gestation age(GA) is a rare complication of pregnancy associated with poor maternal and fetal outcomes. This study measures outcomes between 2018‐2024 at a tertiary referral centre in Ireland with approximately 7000 deliveries per annum. Since the Health (Regulation of Termination of Pregnancy (TOP)) Act, 2018, parents can opt to TOP after 12+GA where there is a risk to the “life or health” of the woman or where the fetus is “likely to die within 28 days of life”.This legislative change means that accurate data is critical for counselling families. Our objective is to describe the outcomes following MTPPROM and compare to a similar study conducted prior to liberalisation of the TOP legislation.

**Study Design**: Retrospective cohort study of consecutive MTPPROM January 2018 to February 2024.

**Results**: 109 patients met inclusion criteria, at a prevalence rate of 0.2%. The median maternal age was 33 and median GA at MTPPROM was 21+0, with an median latency period of 7 days(IQR 26 days, range 0‐147 days). 77% experienced maternal morbidity, with no maternal mortalities. The neonatal survival to discharge (NSTD) was 27.5%. Outcomes are compared to the publication by Linehan et al. paper(Table 1) prior to the Health Act 2018 showing a 5% NSTD rate. MTPPROM between 14+0‐19+6 had a lower NSTD compared to those over 20+ GA(10%vs41%, p value = 0.0003). These cases also had a higher rate of MROP(30%vs13%, p value = 0.02). Anhydramnios was associated with 0% NSTD. Singleton pregnancies with oligohydramnios had a lower NSTD compared to those with normal liquor volume(21%vs51%, p value = 0.007).  90% of the patients who opted for TOP had a deepest vertical pool of less than 2cm and had a higher rate of chorioamnionitis(34%vs73%, p value = 0.01) (Table 2).

**Conclusion**: While NSTD is higher for patients with normal liquor volume and PPROM over 20+ GA, mortality and morbidity still continues to be high. The majority of patients who opted for TOP were diagnosed with chorioamnionitis and oligohydramnios reflecting a higher NSTD percentage post legislation.

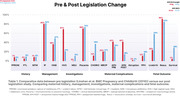

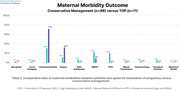

## 673 | Mechanical Cervical Ripening Among Patients with a Bmi Greater Than 30

Alexander M. Saucedo^1^; Miriam Alvarez^2^; Alison G. Cahill^3^; Lorie M. Harper^2^

*^1^Baylor College of Medicine, Houston, TX; ^2^Dell Medical School, Austin, TX; ^3^Dell Medical School Health Transformation Research Institute, Dell Medical School, The University of Texas at Austin, Austin, TX*

10:30 AM ‐ 12:30 PM

**Objective**: Patients with obesity undergoing induction of labor (IOL) are at an increased risk for a prolonged labor duration. The efficacy of a combination of a cervical ripening balloon (CRB) plus vaginal misoprostol (VM) in this population is not well described. We aimed to determine the effect of CRB + VM versus VM alone on time from IOL to delivery in patients with obesity.

**Study Design**: Secondary analysis of a single center, double‐blinded RCT between 6/2022 and 7/2023, which randomized near‐term, singleton gestations with a BMI ≥ 30 kg/m^2^ undergoing IOL, with a cervical dilation ≤ 3cm, to either 25mcg or 50mcg of VM. For this analysis, we included only those patients from the primary study who received a CRB +VM upon induction initiation and compared them to those patients receiving only VM. The primary outcome was time from induction initiation to delivery. Multivariable linear regression was used for the primary outcome with adjustments made for randomization group, nulliparity, and cervical dilation on admission.

**Results**: This secondary analysis included 125 participants: 55 (44%) receiving CRB + VM versus 70 (56%) receiving VM alone. Baseline characteristics were similar, however those with a CRB were younger (26.2 years vs. 29.1 years; p = 0.03) and with fewer Hispanic patients (69.1% vs. 82.9%; p = 0.04). 65 (52%) patients received the 50mcg dose versus 60 (48%) received the 25mcg dose which was used as a covariate in our final model. Following adjustment, patients with a CRB+VM had a reduced time to delivery (14.7 hrs vs. 17.7 hrs; p = 0.02) and reduced time to active labor (11.8 hrs vs. 14.6 hrs; p = 0.02). No differences in cesarean delivery or adverse maternal/neonatal outcomes were seen.

**Conclusion**: In patients with obesity undergoing IOL, a combination of CRB+VM reduced time to delivery and active labor by an average of 3 hours. Given this population's increased risk for prolonged delivery times and perinatal morbidity, a combination of mechanical dilation with misoprostol administration should be considered.

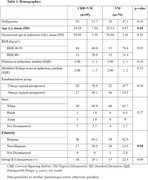

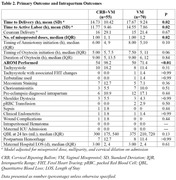

## 674 | Factors Related to Patient Participation in an Obstetric Clinical Trial

Alexander M. Saucedo^1^; Miriam Alvarez^2^; Alison G. Cahill^3^; Lorie M. Harper^2^

*^1^Baylor College of Medicine, Houston, TX; ^2^Dell Medical School, Austin, TX; ^3^Dell Medical School Health Transformation Research Institute, Dell Medical School, The University of Texas at Austin, Austin, TX*

10:30 AM ‐ 12:30 PM

**Objective**: Factors related to patient participation in an obstetric clinical trial have not been well described in literature which is vital towards appropriate study design. We aimed to determine factors which may influence participation in an obstetric clinical trial.

**Study Design**: Cross‐sectional survey of persons eligible for a single center, double‐blinded, labor induction RCT between 6/2022 and 7/2023. Prior to trial enrollment, we administered a 15‐question validated survey which assessed both motivation and barrier factors on a scale from 0 (no influence) to 4 (most influence). Patients were excluded if they voluntarily declined the survey. Comparisons were made between those participating in the trial versus those that declined. The primary outcomes were mean scores obtained in each of the four subset of questions (motivations and barriers). Secondarily, we analyzed differences in these subsets between self‐reported ethnicity. Multivariable linear regression was used for all outcomes.

**Results**: A total of 273 eligible patients were approached for the trial with 180 participating and 93 declining. There were 73 survey respondents with 60 (82.2%) participating in the trial versus 13 (17.8%) declining. Individual questions of the motivation/barrier scales all reached an acceptable level of agreement with Cronbach's alpha scores all > 0.7. Patients who declined the trial expressed less overall influence (0.96 vs. 1.99; p< 0.01) and lower influence by either barriers (p< 0.01) or motivating factors (p = 0.03). Hispanic patients expressed increased influence by motivation 2 questions, such as concordant provider language (1.84 vs. 0.67; p< 0.01) in addition to experiencing an increased impact to barrier 1 and 2 questions, such as family and religious concern (p< 0.05).

**Conclusion**: Patients who declined trial participation had lower motivating and barrier factors which may be due to overall disinterest in research participation. However, Hispanic patients had an increased desire to participate when there was concordant race/ethnicity, language, and sex with their providers.

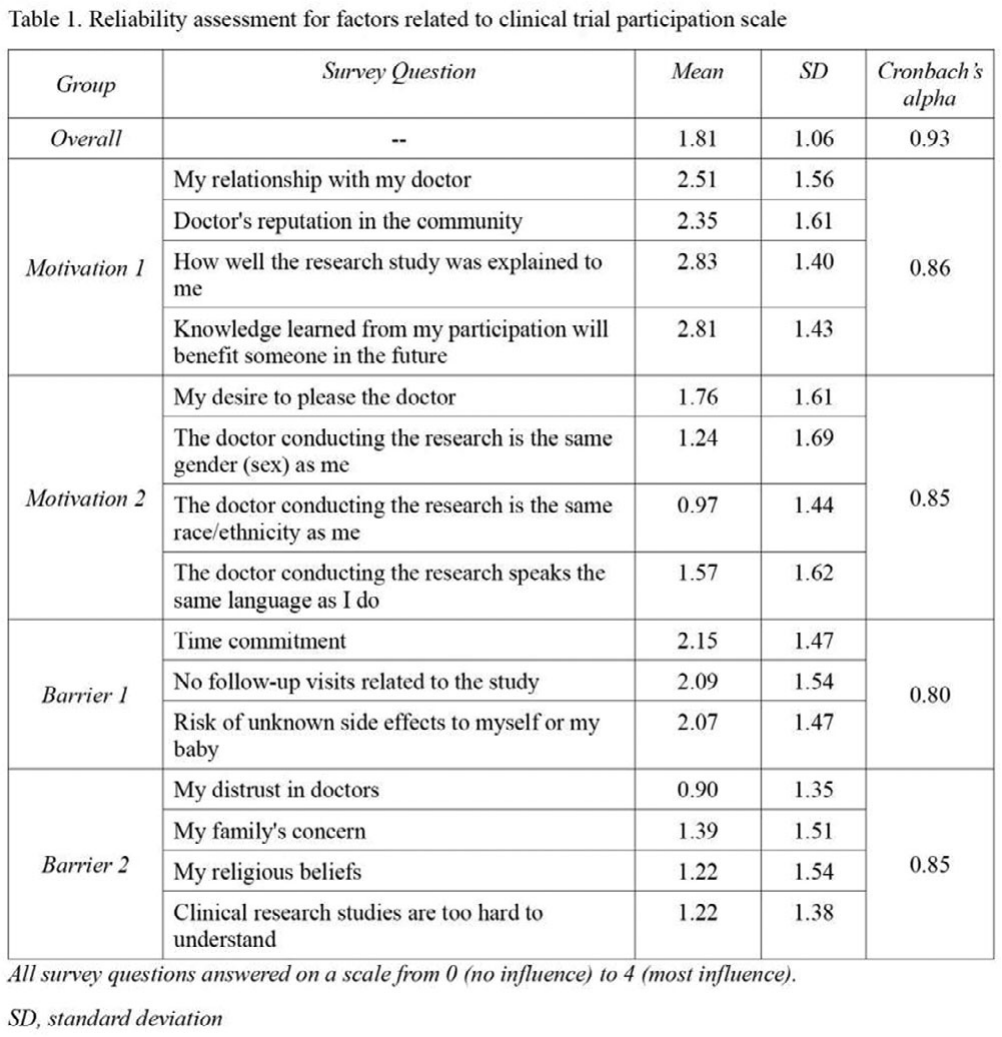

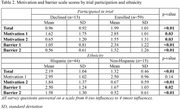

## 675 | Deepest Vertical Pocket with Reflex Amniotic Fluid Index as a Screening Approach for Polyhydramnios

Alexandra JD Phelps^1^; Amelie Pham^1^; Niharika Ravichandran^1^; Matthew Grace^1^; Trey McGonigle^1^; Shi Huang^1^; Aleksandra Polic^1^; Catherine Phillips^1^; Lisa C. Zuckerwise^2^

*^1^Vanderbilt University Medical Center, Nashville, TN; ^2^University of Virginia Health, Charlottesville, VA*

10:30 AM ‐ 12:30 PM

**Objective**: To describe concordance between amniotic fluid index (AFI) and deepest vertical pocket (DVP) in the assessment of amniotic fluid volume and to determine the utility of using DVP alone as a screening test for clinically significant polyhydramnios.

**Study Design**: We performed a retrospective cohort study of all outpatient ultrasounds (US) performed at 20.0‐41.6 weeks gestation at an academic institution from 2018‐2022. Measurements from the US most proximate to delivery were used to analyze the occurrence of polyhydramnios, defined by DVP (≥ 8cm) or AFI (≥ 24cm). We classified severity using accepted definitions by each methodology. Tests of diagnostic performance were calculated using AFI as the accepted gold standard. Pearson correlation coefficient was calculated to describe the relationship between DVP and AFI.

**Results**: Measurements from 11,688 US met inclusion criteria. Polyhydramnios was diagnosed in 739 (6.3%) US using AFI and 875 (7.5%) using DVP. Using DVP of ≥ 8cm as a cutoff identified all cases of moderate or severe polyhydramnios by AFI (Table). However, it also indicated polyhydramnios in 337 cases where AFI was normal, a false positive rate of 3.1%. Of 193 patients with moderate or severe polyhydramnios by AFI, 171 (88.6%) were misclassified as mild by DVP. Using DVP as a screening tool to identify moderate or severe polyhydramnios yielded sensitivity of 100% (95% CI 98.1‐100%), specificity of 94.1% (95% CI 93.6‐94.5%), positive predictive value of 53.1% (95% CI 51.3‐54.9%), and negative predictive value of 100% (95% CI 99.9‐100%). DVP and AFI were highly correlated (Figure, r = 0.84, p < 0.01).

**Conclusion**: Using DVP as a screening tool, with reflex measurement of AFI if DVP is ≥ 8cm, is predicted to identify all cases of moderate or severe polyhydramnios and reduce over‐diagnosis of mild polyhydramnios both in cases of normal AFI and in those with more significant polyhydramnios.

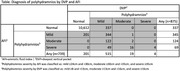

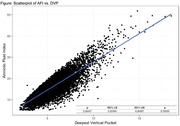

## 676 | Self‐Perception of Pregnancy Risk and Contraceptive Safety Among Women with Cardiac Disease

Alisa M. Goldrich^1^; Jamie Woodley^2^; Margaret English^2^; Hindi Stohl, JD^3^

*^1^University of California Davis Medical Center, University of California Davis Medical Center, CA; ^2^Harbor UCLA Medical Center, Harbor UCLA Medical Center, CA; ^3^UCLA David Geffen School of Medicine Harbor‐UCLA Medical Center, Los Angeles, CA*

10:30 AM ‐ 12:30 PM

**Objective**: To assess perceived safety of contraceptive use and pregnancy among women with cardiac disease in a public hospital setting.

**Study Design**: Women ages 18‐45 with cardiac disease were recruited from the adult congenital cardiac disease or high risk obstetrics clinic at Harbor‐UCLA Medical Center from June 2018 ‐ September 2019. A cross‐sectional survey was administered regarding contraception use, pregnancy history, and perceptions of risk associated with pregnancy in the context of their cardiac condition. Participants were stratified by WHO Classification (Class 1/2 versus Class 3/4) and tested for differences using Wilcoxon Rank Sum tests for numeric variables and Fisher's Exact tests for categorical variables.

**Results**: 42 participants completed the survey: 31 with Class 1/2 disease (73.8%) and 11 with Class 3/4 disease (26.2%). 72.7% of participants with WHO Class 3/4 disease, as well as 61.3% of Class 1/2 participants perceived pregnancy as “Dangerous” or “Very Dangerous”. All (100%) of Class 3/4 group and 87% of Class 1/2 group reported oral contraceptives, regardless of hormone type, as “not safe” or “unsure” about safety. For non‐pill, progesterone‐only methods, most participants, regardless of WHO Class, regarded these as “not safe” or “unsure” (DMPA 80.5%, Nexplanon 85.4%, LNG IUD 75%).

**Conclusion**: High risk cardiac participants accurately recognize pregnancy as dangerous in the context of their disease; however, they are uncertain of safe methods of preventing pregnancy. Low‐risk cardiac patients also perceive pregnancy as dangerous, even though their risk is not significantly different from the general population. The majority of participants with cardiac disease perceive hormonal birth control to be unsafe regardless of disease severity or hormone type. This study highlights the need for improved patient education to increase utilization of effective methods of contraception in this high‐risk patient population.

## 677 | Antenatal Mood Disorders in Placenta Accreta Spectrum

Alison M. Asirwatham^1^; Lindsay Issokson^2^; Hannah Caldwell^2^; Gianna L. Wilkie^2^; Anna R. Whelan^3^

*^1^UMass Chan Medical School, Worcester, MA; ^2^University of Massachusetts Chan Medical School, Worcester, MA; ^3^University of Massachusetts Chan School of Medicine, Worcester, MA*

10:30 AM ‐ 12:30 PM

**Objective**: Placenta Accreta Spectrum (PAS) is a disorder of abnormal invasion of the placenta into or through the myometrium typically treated by cesarean hysterectomy and is associated with increased morbidity and mortality. PAS is associated with increased risks of postpartum mood disorders, but no studies have directly explored antenatal mood disorders in patients with PAS. We aimed to assess antenatal rates of depression and anxiety in those with PAS compared to those with a diagnosis of placenta previa and all pregnancies without these placental diagnoses.

**Study Design**: We performed a retrospective cohort study using data from the EpicCosmos dataset which combines information from >1500 hospitals and >260 million patients. We queried all pregnancies between 7/1/2015‐7/1/2024. We identified those with a diagnosis of placenta accreta, increta, percreta, or placenta previa during pregnancy as well as new diagnoses of depression or anxiety during pregnancy using ICD‐10 codes. Demographic and mood disorder data was compared between groups using chi‐square for categorical variables and ANOVA for continuous variables.

**Results**: In our cohort, we identified 13,944 patients with PAS, 315,756 patients with placenta previa, and 8,153,662 other pregnancies. Mean maternal age was older in those with PAS (37 +/‐6years). Placenta previa had a significantly higher association with antenatal diagnosis of depression (12.1%) and anxiety (18.2%) compared to both PAS (9.9% and 14.5%) and all other pregnancies (9.5% and 13.6%). PAS had non‐significantly increased rates of depression, but significantly increased rates of anxiety compared to all other pregnancies (14.5% vs 13.6%). There were no differences between the subgroups of PAS.

**Conclusion**: In our large cohort, placenta previa was associated with higher rates of depression and anxiety. PAS was associated with higher rates of anxiety compared to all other pregnancies. Given these findings, further investigations focused on how to best screen for perinatal mood disorders and support those affected by placental conditions requiring surgical delivery is needed.

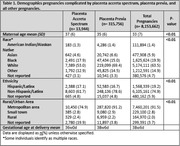

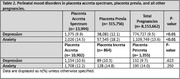

## 678 | Risk of Hypertension 2‐7 Years After Delivery by Timing of Hypertensive Disorder of Pregnancy

Alisse Hauspurg^1^; Lynn M. Yee^2^; Hyagriv Simhan^3^; Judith H. Chung^4^; Lisa D. Levine^5^; Lauren H. Theilen^6^; Philip Greenland^7^; C. Noel Bairey Merz^8^; Jasmina Varagic^9^; Kartik K. Venkatesh^10^; McKenzie K. Jancsura^11^; William A. Grobman^10^; Janet Catov^12^

*^1^Alpert Medical School of Brown University, Providence, RI; ^2^Northwestern University Feinberg School of Medicine, Chicago, IL; ^3^University of Pittsburgh Medical Center, Pittsburgh, PA; ^4^University of California‐ Irvine, Irvine, CA; ^5^University of Pennsylvania Perelman School of Medicine, Philadelphia, PA; ^6^University of Utah, Salt Lake City, UT; ^7^Northwestern, Northwestern/Chicago, IL; ^8^Cedars‐Sinai Medical Center, Los Angeles, CA; ^9^NIH/NHLBI, Bethesda, MD; ^10^The Ohio State University, Columbus, OH; ^11^The Ohio State University College of Nursing, Columbus, OH; ^12^Magee‐Womens Hospital, Pittsburgh, PA*

10:30 AM ‐ 12:30 PM

**Objective**: We sought to assess whether the timing of onset of a hypertensive disorder of pregnancy (HDP) was related to the risk of incident hypertension 2‐7 years after delivery.

**Study Design**: This is a secondary analysis of the NuMoM2b‐Heart Health Study (NuMoM2b‐HHS), a multi‐site cohort that enrolled nulliparous pregnant individuals in their first trimester who were followed during pregnancy and subsequently underwent a cardiovascular screening visit at 2‐7 years after delivery. For this analysis, we excluded individuals with pre‐pregnancy chronic hypertension. We compared rates of hypertension (blood pressure ≥130/80 mmHg or use of anti‐hypertensive medications) at the 2‐7 year postpartum study visit based on timing of onset of HDP (categorized as antepartum, intrapartum, postpartum) versus no HDP (referent). Multivariable logistic regression models adjusted for baseline covariates (age, insurance, tobacco use, diabetes, and early pregnancy body mass index). Interaction analysis was performed to evaluate effect modification by the presence of severe features of HDP.

**Results**: Of 4,342 individuals included in this analysis, 23% had a HDP and underwent follow up at a mean of 3.2± 0.9 years after their first birth. Hypertension at follow up was more prevalent among those with antepartum (37.6%) or postpartum HDP (40.0%) compared to those with intrapartum HDP (26.0%) or no HDP pregnancies (16.5%). Compared to those with non‐HDP pregnancies, those with HDP had higher odds of incident hypertension at follow up regardless of the timing of onset (Table). Findings were similar regardless of the presence of severe features (interaction p = 0.56).

**Conclusion**: Individuals with HDP, regardless of whether it is diagnosed antepartum, intrapartum or postpartum appear to have similarly increased odds of incident hypertension at 2‐7 years postpartum, compared with individuals without HDP in their first birth.

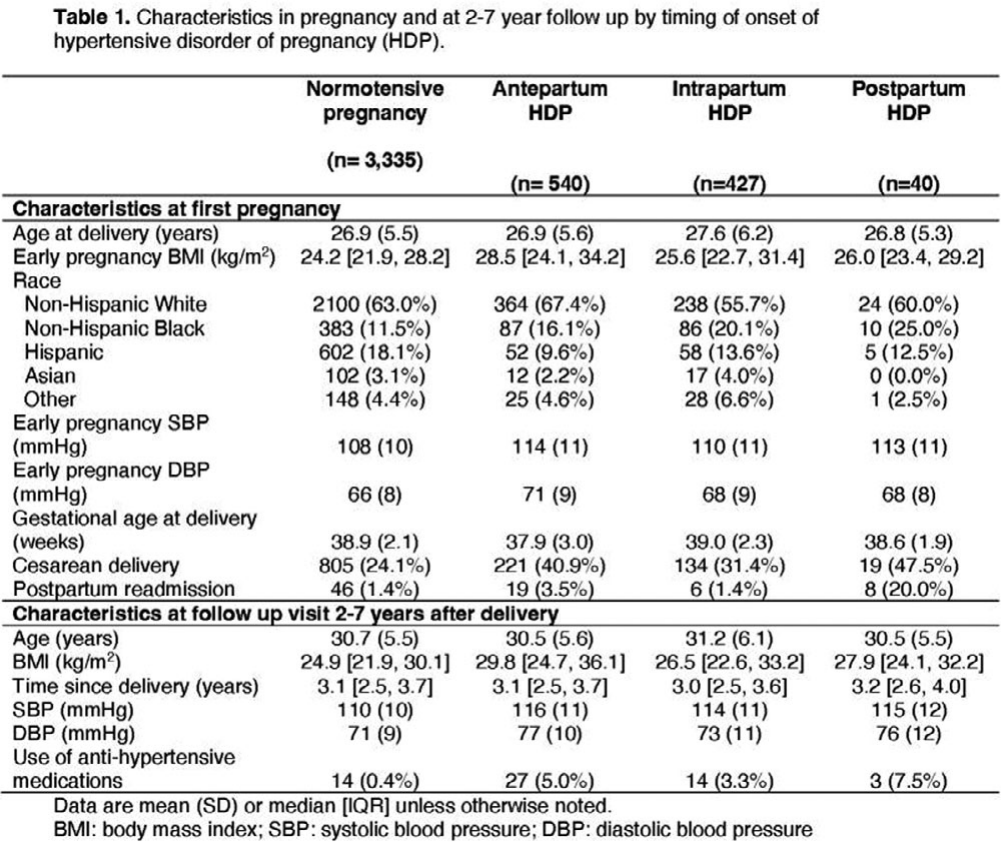

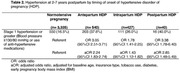

## 679 | The Influence of Epidural Analgesia on Neonatal Outcomes After Balloon Induction of Labor at Term

Alla Saban^1^; Noa Leybovitz Haleluya^2^; Adi Y Y. Weintraub^3^; Reli Hershkovitz^4^; Maayan Elnir Katz^3^; Tamar Eshkoli^3^

*^1^Soroka University Medical Center, Beer Sheva, HaDarom; ^2^Soroka Medical Center, Meitar, HaDarom; ^3^Soroka University Medical Center, Soroka University Medical Center, HaDarom; ^4^Soroka medical center, Soroka medical center, HaDarom*

10:30 AM ‐ 12:30 PM

**Objective**: Epidural analgesia is commonly used for labor and delivery and is generally considered safe. However, it can affect the fetus either directly, through the placental transfer of local anesthetics or opioids, or indirectly, via maternal hypotension. Previous studies have largely focused on comparisons between epidural analgesia and systemic opioids regarding neonatal status. Balloon induction of labor has also become popular due to its minimal side effects. However, only a few studies have specifically addressed the influence of epidural analgesia in the context of balloon induction of labor. This study aims to investigate the association between the use of epidural analgesia following balloon induction of labor and the risk of neonatal adverse outcomes.

**Study Design**: A population‐based retrospective cohort study was conducted, including all term (>37 weeks of gestation) singleton pregnancies that underwent balloon induction of labor at a tertiary medical center between

February 2020 and January 2022. Neonatal adverse outcomes were compared based on the use of epidural analgesia during delivery.

**Results**: The study population included 3,424 deliveries, of which 2,400 (70.1%) used epidural anesthesia and 1,024 (29.9%) delivered without epidural. Neonatal adverse outcomes for both groups are shown in the table. Rates of neonatal adverse outcomes, including low Apgar scores, low cord pH, neonatal intensive care unit (NICU) admission, mortality, and a composite of adverse neonatal outcomes, were comparable between the groups. No significant differences were observed in these outcomes between the groups.

**Conclusion**: The use of epidural analgesia during delivery following balloon induction of labor at term is not associated with an increased risk of short‐term neonatal adverse outcomes.

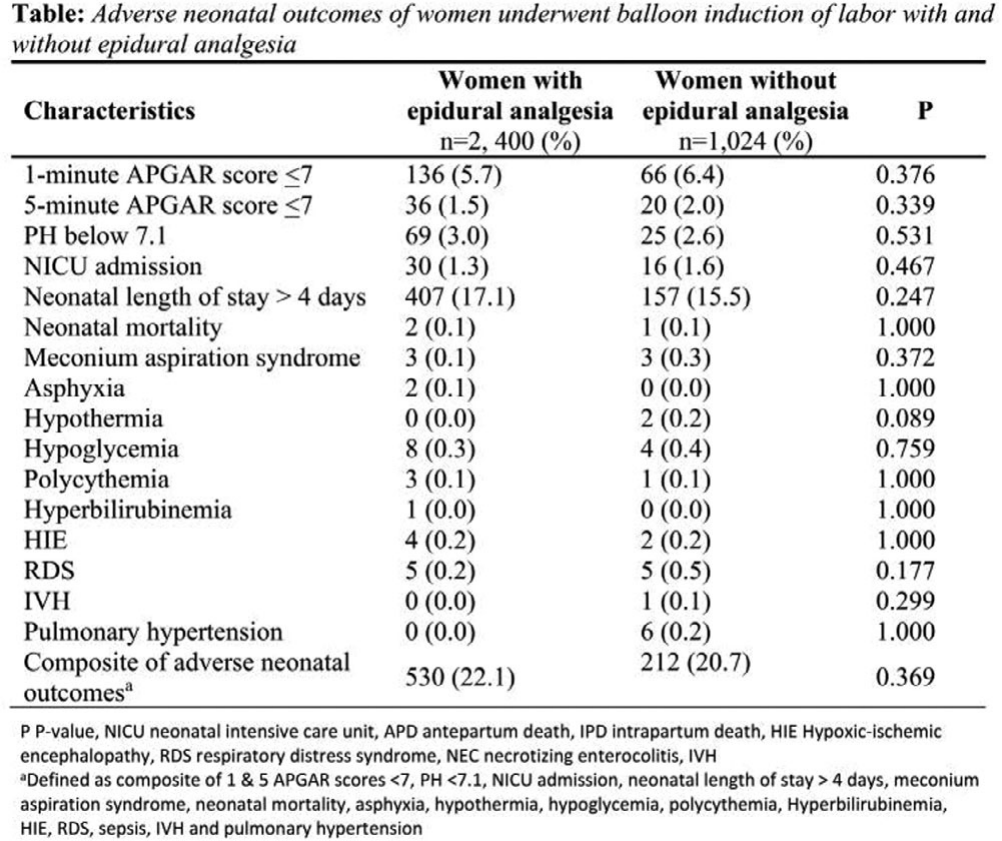

## 680 | The Association Between Timing of Tobacco Smoking Cessation and Fetal Growth Restriction

Allie Sakowicz^1^; Jordan Buzzett^1^; Reyna Segovia Molina^1^; Elizabeth Malone^1^; David M. Stamilio^2^

*^1^Wake Forest University School of Medicine, Winston‐Salem, NC; ^2^Wake Forest University School of Medicine, Winston Salem, NC*

10:30 AM ‐ 12:30 PM

**Objective**: Tobacco smoking is a leading cause of fetal growth restriction (FGR). Our objective was to estimate the association between timing of tobacco smoking cessation and FGR in a cohort of current or former smokers.

**Study Design**: This retrospective cohort study includes all pregnant patients who received prenatal care and delivered in a university health system, delivered a viable neonate between 4/1/22 and 3/31/23, and reported tobacco smoking either during pregnancy or within one year prior to conception. FGR was defined as estimated fetal weight (EFW) or abdominal circumference (AC) < 10% on the most recent ultrasound prior to delivery. Bivariable and multivariable logistic regression analyses were performed. Adjusted odds ratios (aOR) are reported.

**Results**: Of 343 patients included in this study (10.5% of the delivery cohort), 148 (43.2%) quit smoking in the year prior to conception or in the first trimester of pregnancy, 22 (6.1%) quit in the 2^nd^ trimester, 16 (4.4%) quit in the 3^rd^ trimester and 157 (43.6%) continued smoking. Compared to patients who quit smoking early, those who continued smoking beyond the 1^st^ trimester were more likely to identify as Black or Other/unknown race and to be nulliparous. After controlling for confounders, patients who quit tobacco in the year prior to pregnancy or in the first trimester were significantly less likely to be diagnosed with FGR (aOR 0.48, 95% CI 0.24–0.96). Smoking cessation any time prior to delivery was also associated with reduced odds of FGR (aOR 0.46, 95% CI 0.23–0.89) but risk reduction from quitting in the 3^rd^ trimester appeared attenuated.

**Conclusion**: Smoking cessation pre‐pregnancy or in the first trimester is associated with a 52% reduction in FGR compared to continued smoking later in pregnancy. These findings can be used to direct counseling on the benefits of tobacco cessation as early as possible.

## 681 | The Relationship between Neighborhood Food Environment and Readmissions for Postpartum Hypertension and Cardiovascular Complications

Allison Kurzeja; Nicole Beckley; Lindsay Yeh; Sean Young; Donald D. McIntire; David B. Nelson

*University of Texas Southwestern Medical Center, Dallas, TX*

10:30 AM ‐ 12:30 PM

**Objective**: Postpartum readmissions have negative consequences on patients and the healthcare system. Some of the most frequent etiologies for postpartum readmission are hypertensive and cardiovascular‐related. Few studies have assessed the effects of social factors on postpartum readmissions. We aimed to determine whether the neighborhood food environment impacts the frequency of readmission for hypertensive and cardiovascular complications.

**Study Design**: A regional hospital dataset was queried for cardiovascular and hypertension‐related readmissions in the postpartum period using ICD 9/10 codes. We looked at readmissions within 12 weeks of delivery for hypertension and 12 months for cardiovascular complications between 2014 and 2018. Each encounter included a patient's GEO‐ID which allowed linkage to geospatial datasets. The neighborhood food environment was then assessed by measuring the density/distance to select business points‐of‐interest obtained from Data Axle. The Wilcoxon rank‐sum test was used to compare the cohort of patients requiring readmission to those who did not. The effect size was then quantified by quantile regression estimating with 95% confidence the difference in medians.

**Results**: There were 443,578 deliveries in the study timeframe. Of these, 432,947 had associated Data Axle information available and were included in the study. 5,363 (1.2%) patients had a readmission for hypertension within 12 weeks. 2,165 (0.5%) patients had a cardiovascular readmission within 365 days. Patients who required hypertensive‐related readmission were shown to have a significantly lower density of eating places within one kilometer (p = < 0.001). Similarly, individuals who required readmission for cardiovascular‐related complications also had a lower density of eating places within one kilometer (p = < 0.002).

**Conclusion**: It is imperative to consider the interplay of social factors such as the neighborhood food environment on pregnancy outcomes. Differences in food density may impact patients’ ability to access healthy foods, which may relate to subsequent increased readmissions and other health consequences.

## 682 | Postpartum Glycemic Trends Among People with Gestational Diabetes Using Continuous Glucose Monitoring

Alyssa R. Hersh^1^; Alexandra C. Gallagher^2^; Lucy Ward^1^; Christian Huertas‐Pagán^1^; Monica Rincon^1^; Amy M. Valent^1^

*^1^Oregon Health & Science University, Portland, OR; ^2^Stanford University, Palo Alto, CA*

10:30 AM ‐ 12:30 PM

**Objective**: The immediate changes in glycemia following delivery have not been well characterized among pregnant people with gestational diabetes mellitus (GDM), particularly considering the heterogeneity of GDM. Therefore, we sought to characterize postpartum glycemia using continuous glucose monitoring (CGM) data and explore postpartum glycemic trends among people with GDM by GDM and delivery characteristics.

**Study Design**: This was a secondary analysis of pregnant people with GDM randomized to using CGM versus capillary blood glucose for management of GDM. Our primary outcome was mean glucose by hour from delivery through 48 hours after delivery. We stratified by GDM type, mode of delivery and time in range during pregnancy (≥90%, 70‐140) to determine influence on glycemia postpartum.

**Results**: Of the 111 participants in the original analysis, 79 participants with postpartum data were included in this analysis, 22.8% with A1GDM and 77.2% with A2GDM. In the overall cohort, glucose values had a slight downward trend over the first 48 hours post‐delivery (Figure 1a). Upon stratification, participants with A1GDM had lower glucose by 48 hours compared to those with A2GDM (Figure 1b), as did participants with high time‐in‐range compared with lower time‐in‐range during pregnancy (Figure 1c). After cesarean delivery, participants initially had lower glucose values compared to vaginal delivery, which then rose after the first 24 hours prior to returning to baseline by 48 hours (Figure 1d).

**Conclusion**: In this study, we found that postpartum glycemia had a downward trend after delivery and was impacted by multiple pregnancy and delivery characteristics. These results highlight the physiologic changes that are occurring in regard to glycemia during the postpartum period. Future studies should assess whether differences in postpartum glycemic parameters are associated with clinically relevant outcomes.

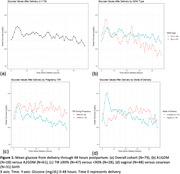

## 683 | Postpartum Glucose Tolerance Test Results Among People with Gestational Diabetes Using Continuous Glucose Monitoring

Alyssa R. Hersh^1^; Alexandra C. Gallagher^2^; Lucy Ward^1^; Christian Huertas‐Pagán^1^; Monica Rincon^1^; Amy M. Valent^1^

*^1^Oregon Health & Science University, Portland, OR; ^2^Stanford University, Palo Alto, CA*

10:30 AM ‐ 12:30 PM

**Objective**: The glycemic profile of people with gestational diabetes mellitus (GDM) at the time of postpartum oral glucose tolerance testing (OGTT; 75 gram 2‐hour challenge) is not well understood. Therefore, we assessed continuous glucose monitoring (CGM) data during the postpartum OGTT, stratifying by normal versus abnormal OGTT and GDM type.

**Study Design**: This was a secondary analysis of pregnant people with GDM randomized to using CGM versus capillary blood glucose (CBG) for management of GDM. Our primary outcome was mean glucose during an OGTT comparing people who had abnormal postpartum OGTT (fasting ≥100 mg/dL or 2‐hour ≥140 mg/dL) compared to normal OGTT results. We performed additional stratified analyses of those by GDM type.

**Results**: Of the 111 participants in the initial trial, there were 51 that had postpartum data available for inclusion of this secondary analysis. Participants had higher fasting (104.3 ± 10.5 vs 89.8 ± 5.2) and 2‐hour plasma glucose (129.9 ± 37.5 vs 103.5 ± 17.9) in the group with abnormal postpartum OGTT results compared to those with a normal OGTT response. Additionally, the glucose excursion following the 75 grams of glucose and mean CGM glucose at all time points were higher in the group with abnormal OGTT results (Figure). When stratified by GDM type, mean CGM glucose was similar between groups.

**Conclusion**: This study demonstrates that CGM values follow the expected trend regarding physiologic response to the OGTT, and those with abnormal OGTT results had a distinct glycemic pattern in response to the OGTT with higher mean CGM glucose at all time points assessed. Future studies will need to assess the predictability of CGM in identifying those with abnormal glycemia postpartum (i.e. prediabetes) with an elevated risk for development of type 2 diabetes mellitus.

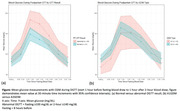

## 684 | Impact of a Surgical Bundle on Hospital Readmission Rates in Obese Women Undergoing Cesarean Delivery

Alyssa Savelli Binsted; Emily Peters; George R. Saade; Tetsuya Kawakita

*Macon & Joan Brock Virginia Health Sciences Eastern Virginia Medical School at Old Dominion University, Norfolk, VA*

10:30 AM ‐ 12:30 PM

**Objective**: To assess whether implementing an obesity surgical bundle for patients with a BMI of 40 kg/m^2^ or greater reduces hospital readmission rates after unscheduled cesarean delivery.

**Study Design**: This was a pre‐ and post‐intervention study of individuals with a BMI > 40 kg/m^2^ undergoing unscheduled cesarean delivery at 24 weeks of gestation or greater between January 2018 and December 2022 at a single tertiary care center. In January of 2021, we implemented an obesity bundle that included vaginal preparation with povidone‐iodine in the setting of ruptured membranes, and a 48‐hour course of oral cephalexin and metronidazole. Our primary outcome was hospital readmission or emergency department visits within 6 weeks postpartum. We also examined the rate of hospital readmission alone. Relative risks with 95% confidence intervals (95% CI) were calculated using modified Poisson regression, adjusting for potential confounders.

**Results**: Of 1823 individuals who underwent unscheduled cesarean, 980 (54%) were in the pre‐intervention period and 843 (46%) in the post‐intervention period. Maternal demographics were overall similar, except for lower gestational age at delivery in the post‐intervention period (Table 1). Compared to the pre‐intervention period, the rate of hospital readmission or emergency department visits was significantly lower in the post‐intervention period (35.0% vs. 28.7%, aRR 0.82 95%CI 0.72‐0.94, P < 0.01). Hospital readmission alone was also significantly lower in the post‐intervention period (33.0% vs. 26.2%, aRR 0.80 95%CI 0.69‐0.92, P < 0.01) (Table 2).

**Conclusion**: The rate of hospital readmissions and emergency department visits decreased significantly after the implementation of a surgical bundle for individuals with a BMI greater than 40 kg/m^2^ undergoing cesarean delivery.

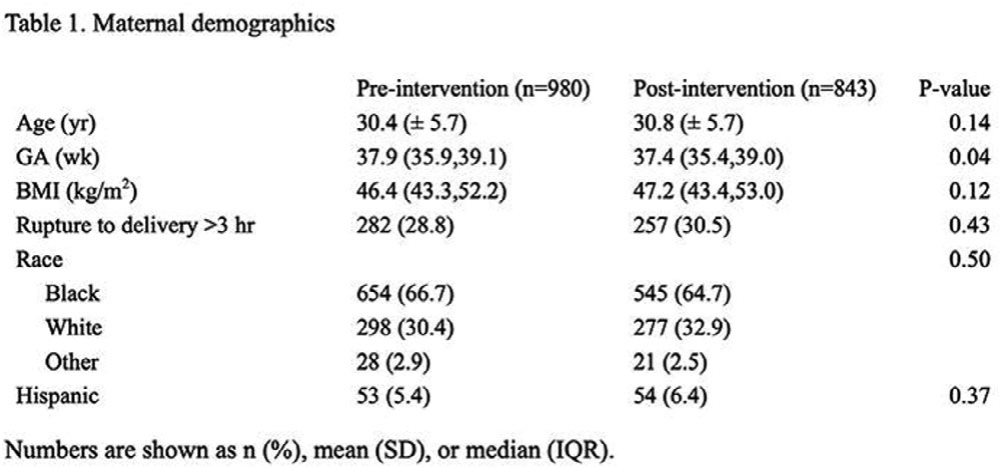

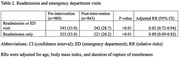

## 685 | Smartphone Application use for Gestational Diabetes Management and Immediate Postpartum Glucose Tolerance Outcomes

Alyssa L. Trochtenberg^1^; Alysa St. Charles^1^; Devika Lekshmi^1^; Erika F. Werner^2^; Sebastian Z. Ramos^2^

*^1^Tufts University School of Medicine, Tufts University School of Medicine, MA; ^2^Tufts University School of Medicine, Boston, MA*

10:30 AM ‐ 12:30 PM

**Objective**: Use of smartphone applications for gestational diabetes (GDM) management has increased in recent years, but their effect on maternal postpartum glucose tolerance is unknown. This pilot randomized controlled trial investigated the effect of the Malama smartphone application, a glucose monitoring and interactive diet education tool, on early postpartum glucose tolerance and perinatal outcomes.

**Study Design**: Patients with GDM receiving prenatal care at an urban academic medical center were randomized 1:1 to Malama application use compared to standard care. The primary outcome was 2 hour oral glucose tolerance test (OGTT) level measured during the delivery hospitalization. Secondary outcomes included patient satisfaction, postpartum hemoglobin A1c (HbA1c), delivery outcomes, and perinatal morbidity.

**Results**: Twenty‐eight participants were randomized (14 participants per group). Malama users had lower median 2 hour OGTT levels compared to patients receiving standard care (120 vs 155 mg/dL; *P* = .05; incident rate ratio, 0.78 [95% CI 0.61‐0.99]). There were no differences in postpartum HbA1c, mode of delivery, birthweight, or other perinatal outcomes. Patient satisfaction with glucose logging methods was equivalent between groups.  When the groups’ baseline characteristics were compared, Malama users were younger than those receiving standard care (30.9±4.1 vs 36.4±3.8 years; *p* = .001), while other characteristics were similar between groups. After adjusting for maternal age, the difference in 2 hour OGTT was no longer significant (adjusted incident rate ratio, 0.92 [95% CI 0.69‐1.23]).

**Conclusion**: Compared with standard glucose logging methods, Malama application use was acceptable to patients with GDM and was associated with improved early postpartum glucose tolerance values in the unadjusted model, but not after adjusting for differences in age. Larger studies are needed, stratifying by age, to fully evaluate the impact of smartphone applications on postpartum glucose and perinatal outcomes.

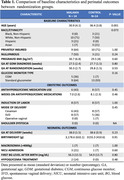

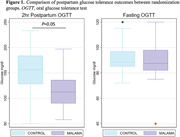

## 686 | Association of Periviable Birth and Postpartum Psychiatric Morbidity in the Medicaid Population

Alyssa L. Trochtenberg^1^; Sebastian Z. Ramos^2^; Benjamin C. Koethe^2^; Mohak Mhatre^2^

*^1^Tufts University School of Medicine, Tufts University School of Medicine, MA; ^2^Tufts University School of Medicine, Boston, MA*

10:30 AM ‐ 12:30 PM

**Objective**: Publicly insured patients have increased risk of adverse obstetric outcomes and perinatal psychiatric morbidity. Although periviable birth is a significantly stressful obstetric event, its association with postpartum psychiatric outcomes has not been studied in this high‐risk population.

**Study Design**: Singleton liveborn periviable births (22 0/7 to 25 6/7 weeks) vs non‐periviable births (≥26 0/7 weeks) were identified using claims data in the Merative MarketScan Medicaid database from 2020‐2022. The primary outcome was a composite of psychiatric morbidity, defined as one or more of the following within 12 months postpartum: emergency department (ED) or outpatient encounters associated with depression, anxiety, psychosis, posttraumatic stress disorder, adjustment disorder, self‐harm, or suicide attempt; new psychotropic medication prescription; or inpatient psychiatry admission. Analysis was adjusted for maternal age, cesarean delivery (CD), severe maternal morbidity (SMM), maternal comorbidity index, and history of mental health disorder.

**Results**: Out of 639,971 deliveries, there were 3,518 (0.5%) periviable births (PB) and 636,453 non‐periviable births (NPB) included for analysis. Baseline prevalence of mental health disorders was similar between groups. The incidence of SMM, hypertensive disorders of pregnancy, and CD was higher among individuals with PB. Compared to NPB, PB was associated with greater incidence of composite psychiatric morbidity (27.3% vs 22.5%, aOR 1.32 [95% CI 1.22‐1.42]) and increased ED utilization for mental health disorders (5.1% vs 3.9%, aOR 1.37 [95% CI 1.17‐1.60]); furthermore, patients with PB were more than twice as likely to have postpartum inpatient psychiatric admission (5.8% vs 2.8%, aOR 2.26 [95% CI 1.95‐2.62]). PB was associated with increased incidence of new psychotropic medication prescriptions and outpatient behavioral health visits.

**Conclusion**: Despite increased outpatient behavioral healthcare utilization, publicly insured patients who experience periviable birth are at high risk for psychiatric morbidity requiring emergency or inpatient‐level of care.

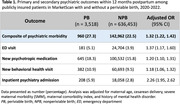

## 687 | Umbilical Artery Doppler Pulsatility Index as a Predictive Biomarker for Preterm Premature Rupture of Membranes

Amanda J. Jones^1^; Ernest Graham^2^; Irina Burd^3^; Ahizechukwu C. Eke^4^

*^1^Christiana Care Health System, Newark, DE; ^2^Johns Hopkins School of Medicine, Baltimore, MD; ^3^University of Maryland, Baltimore, MD; ^4^Johns Hopkins University School of Medicine, Baltimore, MD*

10:30 AM ‐ 12:30 PM

**Objective**: Although known risk factors for preterm premature rupture of the fetal membranes (PPROM) can be assessed, otherwise predicting PPROM is not currently clinically feasible. The objective of this study was to assess the predictive value of umbilical artery Doppler pulsatility index (UA‐PI) alone for PPROM, and when combined with previously established predictive risk factors.

**Study Design**: This was a retrospective cohort study of liveborn non‐anomalous chromosomally normal infants admitted to the neonatology intensive care unit at a single tertiary care center from April 2009 to March 2016. All cases had umbilical artery (UA) Doppler studies routinely measured during second and third trimester ultrasounds. The pre‐rupture UA‐PI of patients whose pregnancies were complicated by PPROM were compared to gestational age‐matched controls without PPROM. The area under the receiver operating characteristic curve (AUC) was used to estimate the predictive ability of pulsatility index (UA‐PI) for predicting PPROM, and the Liu method was used to identify the UA‐PI threshold that maximized both sensitivity and specificity. The AUC, sensitivity, and specificity were also assessed when maternal age, parity, and singleton vs multiple gestation as predictive variables were combined by forward selection with UA‐PI.

**Results**: Of 1,145 high‐risk gestations studied, 262 (23%) were complicated by PPROM and 883 (77%) were without PPROM. Multivariable analysis identified elevated UA‐PI (AOR 1.24, 95% CI 1.18‐1.31, *p* = 0.002) as an independent risk factor for PPROM. The AUC for UA‐PI was 0.57 (95% CI 0.54‐0.61) for an elevated UA‐PI. When maternal age, parity, and singleton vs multiple gestation as predictive variables were combined by forward selection with UA‐PI, the AUC increased to 0.65 (95% CI 0.62‐0.68). The optimal UA‐PI threshold was 0.95 (26% sensitivity, 73% specificity).

**Conclusion**: Elevated UA‐PI is moderately predictive of PPROM, and inclusion in a predictive model for PPROM in high‐risk pregnancies may be justified pending validation.

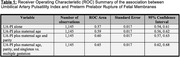

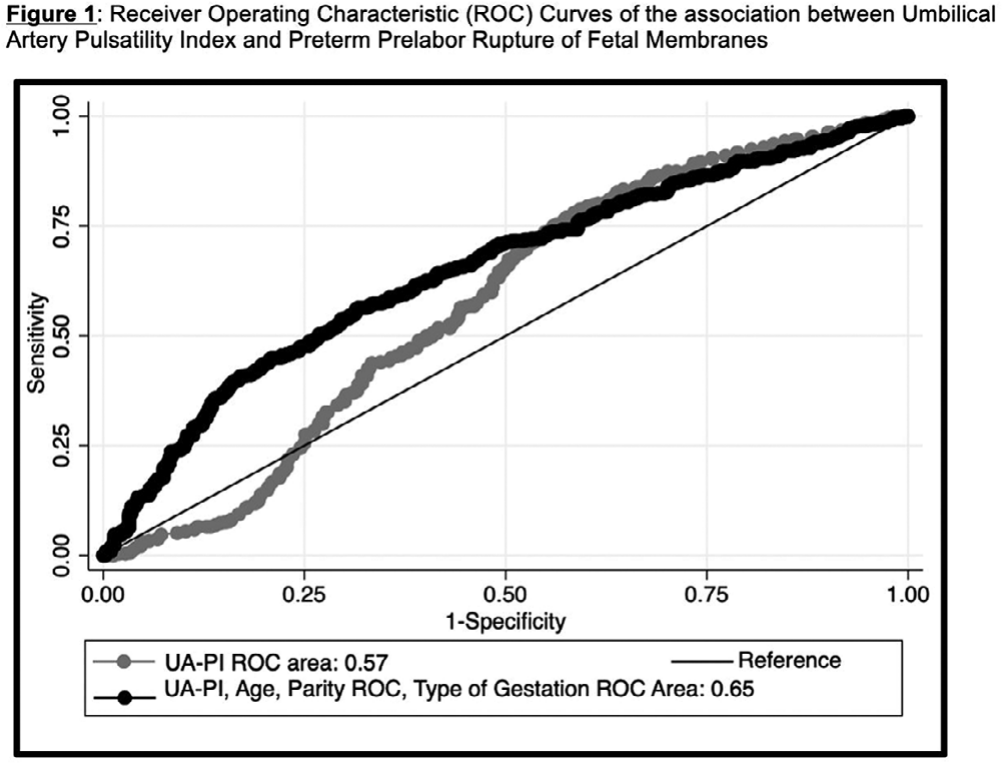

## 688 | Hybrid versus Natural Immunity to Protect Against Neonatal Hospitalization: A Cost‐Effectiveness Analysis

Amelia H. Gagliuso^1^; Lily Ben Avi^2^; Sarah K. Dzubay^2^; Nicole E. Marshall^2^; Aaron B. Caughey^2^

*^1^Oregon Health & Sciences University, Portland, OR; ^2^Oregon Health & Science University, Portland, OR*

10:30 AM ‐ 12:30 PM

**Objective**: Neonates are ineligible for COVID‐19 vaccination and are at increased risk for complications from COVID‐19 infections compared with vaccine‐eligible populations. Passive immunity from maternal anti‐SARS‐COV‐2 immunoglobulins crossing the placenta and in human milk demonstrates that vaccine‐mediated immunity compared to natural immunity is clinically beneficial for both maternal and infant outcomes as shown with TdaP and influenza. The purpose of this analysis was to determine the cost‐effectiveness of maternal hybrid immunity in protection against neonatal outcomes in the United States.

**Study Design**: A TreeAge model was constructed to compare outcomes for a theoretical cohort that were vaccinated with one dose and had a previous infection (Hybrid immunity) with those who received no vaccine and had a previous infection (Natural immunity). Our cohort contained 3,664,292 individuals, the estimated number of annual live births in the United States. Model outcomes included cases of neonatal deaths, neurodevelopmental disabilities, and neonatal hospitalization. Probabilities, utilities, and costs were derived from literature. QALYs were discounted at a rate of 3%.

**Results**:  Hybrid immunity in our theoretical cohort was associated with a decrease in 462 neonatal deaths, 482 cases of neurodevelopmental delay, and 132 cases of neonatal hospitalization (Table 1). Hybrid immunity was the dominant strategy as it saved $2,981,154,191 and led to 32,619 additional QALYs (Table 1). Univariate sensitivity analysis demonstrated that the COVID‐19 vaccine is cost‐saving until the cost of the vaccine exceeds $500, which is far above the current price (Figure 1).

**Conclusion**: This study demonstrates that vaccination even among those with a previous infection improves outcomes and is cost saving in pregnant persons. Ensuring that all pregnant people are aware of the positive impact on their neonates from obtaining the COVID vaccine during pregnancy is important to achieve these potential benefits.

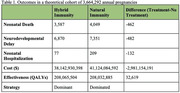

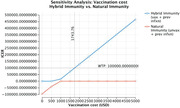

## 689 | Recurrent Intrahepatic Cholestasis of Pregnancy‐ Incidence and Risk Factors

Inbar Lifshitz^1^; Geffen Kleinstern^2^; Roy Zigron^1^; Amihai Rottenstreich^3^

*^1^Hadassah Medical Center, Hadassah Medical Center, NY; ^2^Northwell Health and Rockefeller University, Haifa University, NY; ^3^Northwell Health, Northwell Health, NY*

10:30 AM ‐ 12:30 PM

**Objective**: The incidence of intrahepatic cholestasis of pregnancy (ICP) varies throughout the world and is influenced by genetic, hormonal and environmental factors. However, data is highly limited regarding the risk of ICP recurrence in subsequent pregnancies. We aimed to incidence of recurrent ICP and its associated risk factors.

**Study Design**: A retrospective cohort study conducted at a university hospital, including all pregnant women who underwent serum bile acids (BA) levels testing due to suspected ICP in a 13‐year period. Among women diagnosed with ICP, records were reviewed to identify those with a subsequent gestation.

**Results**: Of 640 women who met the inclusion criteria, 22.2% (n = 142) were diagnosed with ICP (fasting serum BA >10 mmol/L) in the index pregnancy. Of them, 51 (35.9%) had a subsequent pregnancy. The rate of recurrent ICP in the subsequent pregnancy was 29.4% (15/51). Timing of ICP diagnosis did not differ significantly between the index and subsequent gestation (median 33 [IQR 30‐36] vs. 34 [IQR 31‐35] weeks, P = 0.90). In multivariable analysis, fasting serum BA levels at the index pregnancy above 40 mmol/L was the only independent factor associated with ICP recurrence.

**Conclusion**: Recurrence of ICP is common, occurring in over one fourth of women with a history of ICP. BA levels at the first pregnancy in which ICP was diagnosed were found as the only independent factor associated with the occurrence of ICP in subsequent gestation.

## 690 | Long‐Term Gastrointestinal Morbidity Among Twins Conceived by Assisted Reproductive Technology

Amir Snir^1^; Tamar Wainstock^2^; Gil Gutvirtz^3^; Eyal Sheiner^2^

*^1^Soroka University Medical Center, Omer, HaDarom; ^2^Department of Obstetrics and Gynecology, Soroka University Medical Center, Ben‐Gurion University of the Negev, Beer‐Sheva, Israel., Beer Sheva, HaDarom; ^3^Soroka University Medical Center, Metar, HaDarom*

10:30 AM ‐ 12:30 PM

**Objective**: Using assisted reproductive technologies (ART) increased the incidence of multiple pregnancies, which has a negative effect on offspring health outcomes. The long‐term health outcomes for singletons born after ART is well studied with numerous publications, however, studies on ART twin's long‐term morbidities are scarce. This study aimed to investigate a possible association between ART resulting in twin pregnancy and long‐term gastrointestinal (GI) morbidity of the offspring.

**Study Design**: A population‐based cohort study was performed in a tertiary medical center including twin deliveries born between 1991‐2021. Long‐term GI morbidities among twins conceived via ART including ovulation induction (OI) and in‐vitro fertilization (IVF) were compared with twins born following spontaneous pregnancies. The diagnoses of GI morbidities were defined based on ICD‐9 codes as recorded in community clinics and hospitalization files. A Kaplan–Meier survival curve was used to compare the cumulative incidence of GI morbidity among the study group and a Cox proportional hazards model was constructed to control for confounders.

**Results**: A total of 7,790 twins met the inclusion criteria: 2,076 twins (26.6%) were conceived by ART. The total GI morbidity rate was significantly higher in twins conceived by ART as compared with twins from spontaneous pregnancies (34.9% for IVF, 34.3% for OI and 27.0% for spontaneous twins, *p*< 0.001, **Table**). In addition, the cumulative incidence of GI morbidity over time was elevated for twins conceived by ART (log‐rank test, *p* < 0.001, **Figure**). The Cox model, controlling for confounders such as maternal age, gestational age, hypertensive disorders and diabetes mellitus found that using ART resulting in twin pregnancy is an independent risk factor for long‐term GI morbidity of twin offspring (adjusted hazards ratio (aHR) for IVF vs. spontaneous = 1.42 (95%CI 1.27‐1.58, *p*< 0.001; aHR for OI vs spontaneous = 1.38 (95%CI 1.20–1.60, *p*< 0.001).

**Conclusion**: In our cohort, twins conceived by ART have a higher risk for long‐term GI morbidity as compared with twins from spontaneous pregnancies.

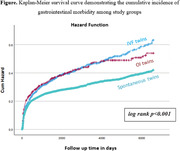

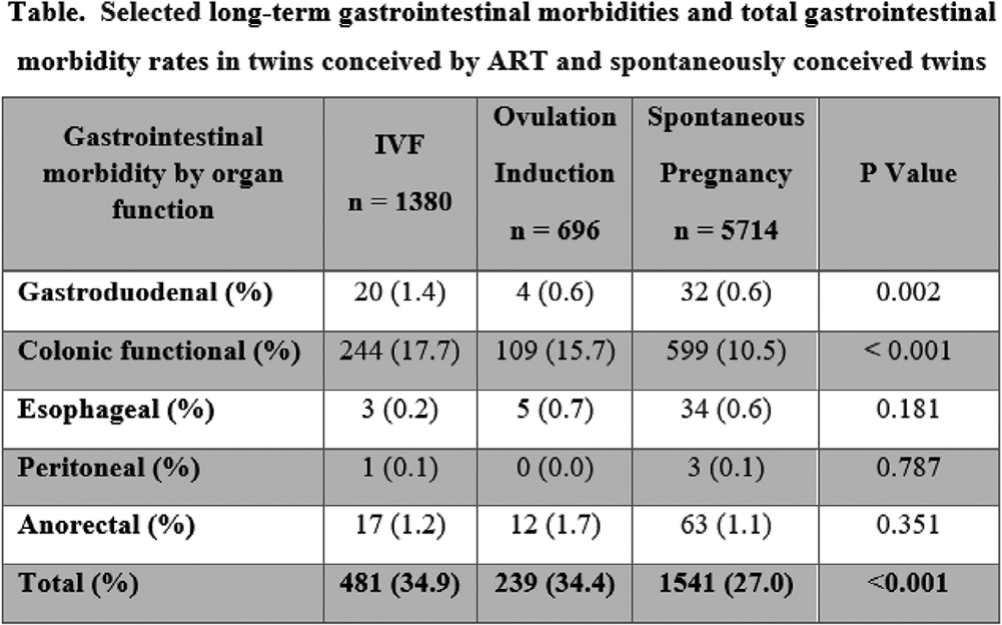

## 691 | Empowering Patients to Conduct Nsts from Home: Success Rates from Over 2000 Remote Nsts

Nadav Schwartz^1^; Barak Laish^2^; Ohad Regev^3^; Amit Reches^4^

*^1^University of Pennsylvania, Perelman School of Medicine, Philadelphia, PA; ^2^Nuvo Group Ltd, Tel Aviv, Tel Aviv; ^3^Nuvo Group Ltd, NuvoCares, HaMerkaz; ^4^Nuvo Group Ltd, Tel aviv, Tel Aviv*

10:30 AM ‐ 12:30 PM

**Objective**: Many pregnancies require multiple, in‐person non‐stress tests (NSTs), adding significant patient burden. Novel technologies can enable remote NSTs; however, the feasibility of this solution in real‐world, clinical settings remains uncertain. We analyzed success rates for patients conducting home NSTs under the remote supervision of their healthcare providers.

**Study Design**: In this retrospective study, we queried an anonymized database to identify remote NST appointments conducted using an FDA‐cleared, remote fetal monitoring device. All NSTs were prescribed and remotely monitored by local healthcare providers. We included remote NSTs conducted ≥32 wks outside of a medical office or research trial and excluded sites with < 10 NSTs. The primary outcome was successful NST appointment, defined as one with a clinically interpretable NST, as documented by the provider. An appointment window was defined as a day in which a remote NST was attempted. For one site conducting twice daily NSTs for de‐hospitalized patients, the appointment was a half‐day. We analyzed maternal age, pre‐gravid BMI, gestational age (GA) and appointment number (appt#) as predictors of success.

**Results**: Between July 2022 and June 2024, 2080 NST appointments we performed in 227 subjects across 10 clinical sites. After excluding 5 subjects and 59 NSTs with no provider documentation, 2031 NST appointments in 222 patients were analyzed. A successful NST was obtained in 92.9% (N = 1887) of appointments. NST success was not related to maternal age (p = 0.51) but was associated with GA (P< 0.01), lower BMI (P< 0.01) and higher appt# (P< 0.01), with the appt# having the greatest impact on success variance (**Table**). Success rates < 85% were observed for first appt# (81%) and BMI≥50 (72%) (**Figure**).

**Conclusion**: Antenatal fetal surveillance is critical to reducing adverse pregnancy outcomes but adds significant burden. Remote NSTs can empower patients to access care from home, while remaining under healthcare team supervision. Our results demonstrate that patients can successfully obtain clinically interpretable NSTs at home in a real‐world setting.

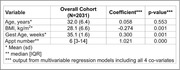

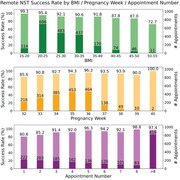

## 692 | Novel Amniotic Fluid Characterization Shows Significant Elevation of EPO and Sflt‐1 in Early FGR Pregnancies

Amrita Roy^1^; Hannah Spector^2^; Sarah Crimmins^3^; Ponnila S. Marinescu^2^

*^1^Bridgeport Hospital / Yale New Haven Health, Bridgeport, CT; ^2^University of Rochester Medical Center, Rochester, NY; ^3^University of Rochester, Rochester, NY*

10:30 AM ‐ 12:30 PM

**Objective**: Fetal growth restriction is a major contributor to fetal and neonatal morbidity/mortality. Early onset FGR [eFGR, diagnosed < 32 weeks] has a strong association with catabolism, and markers of chronic hypoxia are proportionally increased in this state, likely in amniotic fluid (AF). However, comparisons of AF analytes between eFGR and appropriately grown for gestational age (AGA) pregnancies are limited. We analyzed concentrations of AF lactate, endothelin‐1 (ET‐1), soluble fms‐like tyrosine kinase 1 (sFlt‐1), erythropoietin (EPO), and indoleamine‐2,3‐dioxygenase (IDO) in eFGR vs AGA control groups.

**Study Design**: Retrospective case control study using cryopreserved AF samples; eFGR (cases) and AGA (control) pregnancies were identified via EMR. Analyses of lactate, ET‐1, EPO, IDO‐1, and sFlt‐1 were performed using ELISA. Primary outcome was concentration of AF analytes in eFGR versus control groups. Subgroup analyses assessed concentrations of AF analytes in eFGR, eFGR with brain sparing (eFGR+BS) and control groups.

**Results**: 44 AF samples were evaluated, 11 with eFGR [7 of which were eFGR+BS], 33 with AGA. AF concentrations of EPO and sFlt‐1 were higher in the eFGR group (EPO eFGR 4.41mIU/mL vs. control 1.54 mIU/mL p = 0.005; sFlt‐1 eFGR 0.51 ng/mL vs. control 0.27 ng/mL p = 0.002). AF lactate concentrations were lower in the eFGR group (eFGR 4.99 mmol/L vs. control 5.37 mmol/L p = 0.034). There were no statistically significant differences in ET‐1 or IDO‐1 between groups. AF concentrations of EPO and sFlt‐1 were higher in the eFGR and eFGR+BS cohorts when compared to control (EPO eFGR 7.82 mIU/mL, eFGR+BS 4.41 mIU/mL vs. control 1.54 mIU/mL p = 0.005; sFlt‐1 eFGR 0.56 ng/mL, eFGR+BS 0.40 ng/mL vs. control 0.27 ng/mL p = 0.002).

**Conclusion**: Our novel characterization of AF analytes shows that EPO and sFLt‐1 are significantly elevated in pregnancies with eFGR and eFGR+BS. Ability to identify at risk pregnancies has the potential to meaningfully impact management, particularly in identifying time frame for intervention.  Further studies will assess predictability of eFGR by AF analyte combinations.

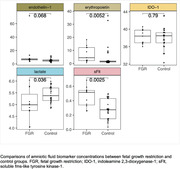

## 693 | Aortocaval Compression during Maternal Cardiac Arrest: An MRI Study of Uterus‐Great Vessel Dynamics Across Trimesters

Andrea D. Shields^1^; Ava G. Holland^2^; Olatoyosi Olafuyi^3^; Makayla Murphy^4^

*^1^University of Connecticut Health, Avon, CT; ^2^UConn Hospital, UConn Hospital, CT; ^3^University of Connecticut, Farmington, CT; ^4^University of Connecticut Health, University of Connecticut Health, CT*

10:30 AM ‐ 12:30 PM

**Objective**: During maternal cardiac arrest (MCA), two crucial maneuvers to alleviate aortocaval compression caused by the gravid uterus are left uterine displacement (LUD) and resuscitative cesarean delivery (RCD). This study aims to examine the anatomic relationship between the uterus and aorta, as well as the presence of inferior vena cava (IVC) compression, using magnetic resonance imaging (MRI).

**Study Design**: Post‐acquisition measurements were obtained from MRI imaging of pregnant women with singleton fetuses between 12+4–35+0 weeks for various indications. Measurements were obtained with patients in the supine position at the transverse plane just above the level of the aortic bifurcation, including the distance between the left aortic wall and right uterine wall (LAo‐RUt) and presence of inferior vena cava (IVC) compression (Figure).

**Results**: Fifty MRIs in pregnant patients were analyzed and stratified by gestational age (Table 1). As expected, the LAo‐RUt distance increased with advancing gestational age, with a range of 67.7–180.5 mm. MRI evidence of compression of the IVC was present in 36% of pregnant patients between 12+0–19+6 weeks, 75% of gravidas between weeks 20+0–23+6 weeks, and 90% of gravidas between 32+0 –35+0 weeks. The earliest gestational age IVC compression was noted was 15+5 weeks.

**Conclusion**: These findings indicate that most pregnant patients experience IVC compression beyond 20 weeks’ gestation due to the uterus overlying the great vessels. LUD and RCD are most likely to be effective in alleviating aortocaval compression after 20 weeks. However, given that 1/3 of patients less than 20 weeks have evidence of IVC compression, LUD and RCD may also play a role in the resuscitation of patients after 15 weeks, especially those in refractory MCA.

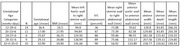

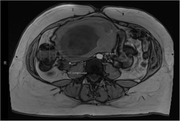

## 694 | Care‐Ier to Be Screened Today? an Insight on Partner Genetic Screening Uptake Rates

Ajitha Chivukula^1^; Brittany Gancarz^2^; Makayla Murphy^2^; Andrea D. Shields^3^

*^1^University of Connecticut School of Medicine, University of Connecticut School of Medicine, CT; ^2^University of Connecticut Health, University of Connecticut Health, CT; ^3^University of Connecticut Health, Avon, CT*

10:30 AM ‐ 12:30 PM

**Objective**: We aimed to analyze the uptake rates for genetic carrier screening in partners of pregnant people with abnormal carrier screens and investigate the reasons for deferred screening.

**Study Design**: A retrospective cohort analysis was conducted on pregnant patients with positive carrier screens and their partners at a tertiary academic medical center between 6/1/2021 and 5/31/2024. Inclusion criteria were pregnant people (18+) with singleton pregnancies who tested positive for an autosomal recessive condition in the first or second trimester and underwent genetic counseling. The primary outcome was uptake rates for genetic screening in partners of pregnant people with positive carrier screens. Secondary outcomes were reasons for deferring partner screening. Baseline characteristics were summarized using frequencies and percentages for categorical variables and mean and standard deviation for continuous variables.

**Results**: During the study period, 701 abnormal genetic screens were identified, of which 587 were in pregnant people who met inclusion criteria. Most abnormal screens (56.4%) were identified from pan‐ethnic screening while 8% were identified through targeted carrier screening. The uptake rate of partner screening was 48.04%, with 33.3% undergoing targeted screening for a specific condition and 60% undergoing a panethnic panel. 38.9% of partners had discordant positive carrier results for conditions unrelated to the pregnant person's condition. Genetic screening by blood samples occurred in 76.7% of partner tests, while 23.3% were from saliva samples. The most common reasons partners deferred genetic screening included perceived low risk of inheritance for the condition (61%), busy work schedules/life factors (21%), and costs (9%).

**Conclusion**: Our results show that nearly half of the partners of pregnant individuals who receive genetic counseling for abnormal carrier screens accept targeted or enhanced genetic screening, mostly through blood sampling. Partners’ perceptions of risk and screening convenience significantly influenced their decisions to defer screening.

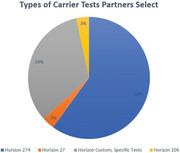

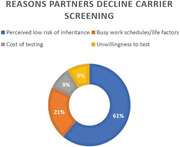

## 695 | Increased Chronic Inflammation of the Placenta in COVID‐19‐Infected Pregnant Patients

Jasmine Rios^1^; Renee Odom‐Konja^2^; Lauren S. Keenan‐Devlin^3^; Linda M. Ernst^2^; Alexa A. Freedman^4^; Greg E. Miller^4^; Ann EB Borders^5^

*^1^Pritzker School of Medicine, University of Chicago, Chicago, IL; ^2^Endeavor Health, Evanston, IL; ^3^Endeavor Health/ University of Chicago Pritzker School of Medicine, Endeavor Health, IL; ^4^Northwestern University, Chicago, IL; ^5^Endeavor Health, Evanston Hospital, Evanston, IL*

10:30 AM ‐ 12:30 PM

**Objective**: COVID‐19 infection during pregnancy has been linked to adverse birth outcomes, potentially due to increased inflammation. This study compared placental inflammation in individuals with COVID‐19 infection during pregnancy to placentas of those who delivered prior to the pandemic to understand inflammation associated with COVID‐19 infection and timing.

**Study Design**: Participants were enrolled in the Stress, Pregnancy, and Health study from 2018‐2022. Placentas were collected < 2 hours from delivery and histologically examined. Medical records were manually reviewed for evidence of COVID‐19; infection timing, severity, symptoms, vaccination status were abstracted for those infected. The control group included placentas from births before December 1, 2019. Models were adjusted for maternal age, race/ethnicity, and socioeconomic characteristics.

**Results**: Among the 605 participants, 57 had confirmed COVID‐19 infections and 214 delivered before December 1, 2019. Of the 57 positive cases, 5(8.7%) were asymptomatic, 39(68.4%) mild, 1(1.7%) severe, and 6(10.5%) did not have documented symptoms. Those infected with COVID‐19 during pregnancy had increased odds of high‐grade placental chronic inflammation (OR = 2.23, CI 1.06‐4.69), particularly amongst those infected before 20 weeks gestation (OR = 3.57, CI 1.25‐10.18). Prevalence of chronic basal villitis was higher in those infected with COVID‐19 (p< 0.001). There were no significant differences in acute inflammation (p = 0.169), fetal vascular pathology (p = 0.488), or maternal vascular pathology (p = 0.255) between the infected and uninfected cohorts.

**Conclusion**: In a cohort of predominantly mild COVID‐19 infections during pregnancy, COVID‐19 was associated with increased odds of placental chronic inflammation, driven primarily by chronic basal villitis. Inflammatory patterns differed by timing of infection, with increased odds of chronic placental lesions among those infected early in pregnancy. These findings enhance understanding of COVID‐19 infection's inflammatory effects in pregnancy, aiding in prevention and treatment of at‐risk pregnant patients.

## 696 | Implementation of Birth Equity Strategies in a Statewide Quality Improvement Initiative Supports Equitable Care Efforts

Patricia Lee King^1^; Aleena Surenian^1^; Lavisha Singh^2^; Alana Rivera^3^; Ann EB Borders^2^

*^1^Illinois Perinatal Quality Collaborative, Northwestern University, Chicago, IL; ^2^Endeavor Health, Evanston Hospital, Evanston, IL; ^3^Illinois Perinatal Quality Collaborative, Endeavor Health, Evanston Hospital, Evanston, IL*

10:30 AM ‐ 12:30 PM

**Objective**: The Illinois Perinatal Quality Collaborative (ILPQC) Birth Equity (BE) quality improvement (QI) initiative, launched in July 2021 to implement key actionable strategies to address birth equity across Illinois hospitals. We aim to identify hospital team progress towards meeting initiative goals from baseline (Oct‐Dec 2020) to initiative end and transition to sustainability (Jan‐Mar/Q1 2024).

**Study Design**: ILPQC facilitated collaborative learning, rapid‐response data and QI support for hospitals. Hospitals reported monthly progress on implementation of key systems changes, including staff respectful care and implicit bias education. Hospitals also conducted a monthly review of 10 randomly sampled patient charts to evaluate progress on Social Determinants of Health (SDOH) screening and documentation of linkage to resources. Chi‐square tests analyzed birthing hospitals’ progress on key systems changes and implementation differences by hospital characteristics from baseline to initiative end Q1 2024. Generalized linear mixed models, adjusted for hospital characteristics, were used to evaluate progress on staff education, SDOH screening and SDOH linkage.

**Results**: 63 participating hospitals were included. Hospital teams significantly increased implementation of all BE key systems changes between baseline and Q1 2024, regardless of hospital characteristics (Table 1). Staff receiving education on respectful care and implicit bias increased from 21% at baseline to 93% in Q1 2024 (p< .0001). SDOH screening increased from 17% at baseline to 92% in Q1 2024 (7% per quarter, p< .0001). SDOH linkage to resources increased from 57% at baseline to 78% in Q1 2024 (1% per quarter, p = .034, Figure 1).

**Conclusion**: The results suggest that a statewide collaborative can facilitate equitable implementation of key Birth Equity strategies through a QI initiative. Hospital teams submitted plans to sustain these improvements by engaging patient partners to obtain respectful care feedback, partner with community organizations to improve SDOH linkages and continue to disaggregate data by race, ethnicity and insurance status.

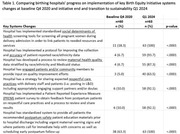

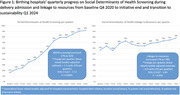

## 697 | Cardiac and Obstetric Outcomes Among Individuals with Hereditary Thoracic Aortic Diseases: Leveraging Large EMR Data

Anna R. Whelan^1^; Lara Kovell^2^; Melissa L. Russo^3^; Gianna L. Wilkie^4^

*^1^University of Massachusetts Chan School of Medicine, Worcester, MA; ^2^UMass Memorial Health, Worcester, MA; ^3^Women & Infants Hospital of Rhode Island / Alpert Medical School of Brown University, Providence, RI; ^4^University of Massachusetts Chan Medical School, Worcester, MA*

10:30 AM ‐ 12:30 PM

**Objective**: Pregnancy, delivery, and the postpartum time period are times of increased risk for aortic dissection (AD) among patients with hereditary thoracic aortic diseases (HTAD). Due to the relative rarity of AD and HTAD, data on the risks of AD and other cardiac complications comes from small case series. We aimed to leverage Epic Cosmos, a large dataset comprised of electronic medical records from across the country to estimate current risks of AD and cardiac complications among patients with HTAD during pregnancy.

**Study Design**: We performed a descriptive analysis of a cohort of pregnancies from The Epic Cosmos dataset, which combines data from over 1500 hospitals and >250 million patients. Epic Cosmos was queried for patients with simultaneous diagnoses codes for HTAD (Marfan Syndrome, Loeys‐Dietz Syndrome, Vascular Ehlers‐Danlos Syndrome and bicuspid associated aortopathy) and pregnancy between July 5, 2021 and July 4, 2024. Rates of AD, other cardiac complications (including aortic rupture, cardiac arrest, cardiac arrhythmia), and pregnancy outcomes were abstracted for all included individuals.

**Results**: We identified 1171 individuals with HTAD and pregnancy in the selected time frame. Most individuals identified as White (81.6%). Marfan syndrome was the most common HTAD diagnosis (74.6%). The rate of AD in this cohort was 1.1% (n = 13). The most common cardiac complication experienced by these individuals was arrhythmia (18.4%). For 698 individuals, complete pregnancy and obstetric outcome data was available, and 46.8% were nulliparous. Approximately 20.8% of patients had a cesarean delivery and 30.5% had a preterm delivery (< 37 weeks). Twelve percent of individuals opted for a form a long‐acting reversible contraception following pregnancy (Implant n = 27, IUD n = 113).

**Conclusion**: In this large, contemporary cohort rates of AD were 1.1% among patients with HTAD in pregnancy.  Prospective studies are needed to assess the optimal management of pregnancy among patients with HTAD to decrease cardiac and obstetric complications.

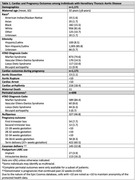

## 698 | Association of CDC Social Vulnerability Index and Postpartum Care Attendance

Anna P. Staniczenko^1^; Julie Robin Dean^1^; Kristin M. Voegtline^2^; Caitlin Radford^2^; Charlene Thomas^2^; Steven Yen^2^; Evan Sholle^2^; Lauren M. Osborne^2^; Julianne R. Lauring^2^; Heather S. Lipkind^2^; Moeun Son^2^

*^1^Weill Cornell Medical College, New York, NY; ^2^Weill Cornell Medicine, New York, NY*

10:30 AM ‐ 12:30 PM

**Objective**: Increasing the rate of postpartum follow‐up has been identified as a national priority to decrease maternal morbidity and promote maternal health. We sought to evaluate whether there is an association between the CDC's Social Vulnerability Index (SVI) and postpartum care attendance.

**Study Design**: This is a retrospective case control study of patients who delivered at >20 weeks’ gestation at any of 3 hospitals within a New York City healthcare system from 1/1/2022 to 2/1/2024 and had at least one prenatal visit at an affiliated site prior to delivery. The outcome of postpartum care was dichotomized as at least one outpatient obstetric visit within 12 weeks of delivery. The exposure was CDC‐defined SVI, using a validated technique for geocoding patient addresses to assign overall SVI as well as SVI theme scores. SVI was categorized into quartiles representing low to high social vulnerability; more vulnerable patients have higher SVI scores. Demographic, medical, and obstetric factors were examined, and bivariate and multivariate logistic regressions performed.

**Results**: A total of 21,864 patients were included. Overall, 89.2% attended their PPV, but 33.3% of those with public insurance and 37.8% of those who delivered at community‐based sites did not attend their PPV. Among all patients, the distribution of PPV attendance substantially varied based on SVI quartile (Figure), and the odds of attending at least one PPV significantly decreased as SVI increased in a dose‐dependent fashion (Table). These patterns persisted among the socioeconomic, household, and housing/transport themes for SVI (Table). After adjusting for important potential confounders, the significant association between high SVI and decreased odds of attending PPV persisted (aOR 0.72, 95% CI 0.59, 0.88).

**Conclusion**: Social vulnerability is significantly associated with likelihood of postpartum visit attendance within 12 weeks of delivery: those with the highest vulnerability have the lowest odds of following up for postpartum care. The CDC's SVI may be an effective screening tool to identify those at risk of loss to follow‐up postpartum.

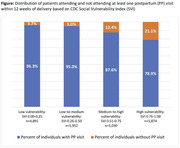

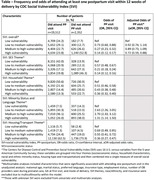

## 699 | Association Between Breastfeeding and Neurodevelopmental Outcomes After Late Preterm Birth

Anna Palatnik; On behalf of the Eunice Kennedy Shriver National Institute of Child Health and Human Development Maternal‐Fetal Medicine Units Network

*Medical College of Wisconsin, Milwaukee, WI*

10:30 AM ‐ 12:30 PM

**Objective**: Despite the known benefits, it remains unclear whether breastfeeding improves neurodevelopmental outcomes. We tested the hypothesis that breastfeeding is associated with improved neurodevelopmental outcomes among children delivered late preterm.

**Study Design**: Secondary analysis of a prospective, follow‐up study of ≥ 6 years old children enrolled in the NICHD Antenatal Late Preterm Steroid (ALPS) Trial, a multicenter study that assessed betamethasone administration in pregnancies at risk for late preterm birth (34‐36wks). Term births and those with missing breastfeeding data or the Differential Ability Scales‐II (DAS‐II) were excluded. The exposure was breastfeeding at 3 months of life collected via interviews. The primary outcome was cognitive function at age ≥ 6, measured by DAS‐II core components of the General Conceptual Ability (GCA) that includes verbal, non‐verbal reasoning, and spatial abilities. General linear models were used to evaluate the association of breastfeeding with DAS‐II GCA after controlling for pre‐specified baseline characteristics (maternal age and education, gestational age at birth, child age, sex, tobacco use in pregnancy, ALPS study group, study center).

**Results**: Of 541 participants included in the analysis, 273 (50.5%) were breastfeeding at 3 months of life. Participants who breastfed were older, more likely to be white, have a college degree, be married, have private insurance, and less likely to use tobacco in pregnancy (Table 1). There was no difference in birthweight or gestational age at birth by breastfeeding status. The primary outcome, DAS‐II GGA, was higher among children who were breastfed at 3 months, 98.9±13.3 vs. 93.0±12.9, mean difference of 5.90 (95% CI 3.59, 8.21, adjusted mean difference 2.27, 95% CI 0.03, 4.50) (Table 2). In secondary analyses, differences in the overall GCA appear to be driven by increased scores for spatial ability.

**Conclusion**: Among children born late preterm at 34‐36 weeks, breastfeeding at 3 months of life was associated with improved neurodevelopmental outcomes at age 6 or older.

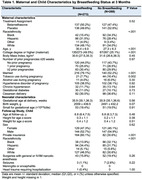

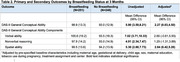

## 700 | Risk Factors of Prostaglandin Failure in the Management of Postpartum Hemorrhage After Vaginal Delivery

Anne‐Sophie BOUCHERIE^1^; ANNE ROUSSEAU^2^; PATRICK ROZENBERG^3^; THIBAUD QUIBEL^2^

*^1^UVSQ, POISSY, Ile‐de‐France; ^2^UVSQ, Poissy, Ile‐de‐France; ^3^UVSQ, Neuilly sur seine, Ile‐de‐France*

10:30 AM ‐ 12:30 PM

**Objective**: To identify risk factors for prostaglandin E2 (sulprostone) failure in postpartum hemorrhage after vaginal delivery.

**Study Design**: We conducted a secondary analysis of the TUB trial, a multicenter randomized trial assessing the efficacy of intrauterine balloon tamponade (IUBT) used simultaneously with prostaglandin versus its use after prostaglandin failure. We selected women of the control group who received standard care, *i.e*. prostaglandin administration and then IUBT if bleeding persisted. Prostaglandin failure was defined as the need for IUBT to stop bleeding. We compared maternal and labor characteristics according to prostaglandin failure. Multivariate logistic regression was used to identify independent risk factors for prostaglandin failure and calculate adjusted odds ratios with 95% confidence intervals.

**Results**: Among the 193 women, 39 (20.2%) required IUBT for persistent bleeding despite prostaglandin administration. Maternal and labor characteristics did not differ between the groups. The mean time from birth to prostaglandin administration was 81±63 minutes in the success group and 76±55 minutes in the failure group (p‐value .6). The rate of women receiving prostaglandin within 30 minutes after birth was 8.7% in the success group versus 10% in the failure group (p‐value .8). The mean quantified blood loss at prostaglandin administration was 872 ± 286 mL in the success group and 955 ± 411 mL in the failure group (p‐value .2). Thrombocytopenia (OR 15.8 [1.89‐329], p‐value .02) and multiple pregnancy (OR 2.76 [1.01‐7.16], p‐value .04) were identified as independent risk factors for prostaglandin failure.

**Conclusion**: Neither the time of administration of prostaglandins nor the amount of bleeding predicts failure of this treatment. Only thrombocytopenia and multiple pregnancy were independently associated with prostaglandin failure.

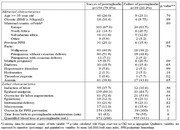

## 701 | Outcomes of Cesarean and Vaginal Delivery in Nulliparous Deliveries by Hospital Cesarean Delivery Rate

Annika Willy^1^; Bharti Garg^2^; Aaron B. Caughey^2^

*^1^Oregon Health & Science University, Bend, OR; ^2^Oregon Health & Science University, Portland, OR*

10:30 AM ‐ 12:30 PM

**Objective**: It is unknown how outcomes differ among patients delivering in hospitals with differing rates of cesarean delivery. Therefore, we sought to assess an association between patients delivering at hospitals with different cesarean delivery rates and adverse perinatal outcomes stratified by cesarean or vaginal delivery.

**Study Design**: This was a retrospective cohort study of singleton, non‐anomalous, term deliveries with vertex presentation in nulliparous individuals of California (2016‐2020). Using the cesarean delivery rate in each hospital, we categorized hospitals into low (< 22%), medium (22.1‐26.3%) and high (≥26.3%) cesarean rate hospitals. Multivariable Poisson regression models were used to examine the association of low and medium cesarean rate hospitals with adverse outcomes, using high cesarean rate hospitals as reference. These association were examined separately among patients delivering via cesarean or vaginally. Adjusted risk ratios (aRR) with 95% CI were estimated.

**Results**: In this study, we included 507,455 deliveries, of which 102,486 (20.2%) were cesarean and 404,968 (79.8%) were vaginal. As compared to high CD rate hospitals, individuals with cesareans in low CD rate hospitals had higher risk of SMM (3.31% vs 1.67%; aRR = 2.07 (1.57‐2.73)), wound infection (0.50% vs 0.32%; aRR = 1.67 (1.11‐2.51)), NICU admissions (7.94% vs. 6.01%; aRR = 1.42 (1.11‐1.81)), and lower Apgar scores (3.16% vs. 1.63%; aRR = 1.79 (1.45‐2.20)). Results were similar in individuals with vaginal deliveries, such as low CD rate hospitals had higher risk of SMM (1.93% vs 0.84%; aRR = 1.92 (1.44‐2.58)), wound infection (0.13% vs 0.08%; aRR = 1.59 (1.09‐2.32)) and lower Apgar scores (1.44% vs 0.93%; aRR = 1.43 (1.17‐1.74)).

**Conclusion**: We found that patients delivering at hospitals with a low cesarean delivery rate had higher rates of several adverse outcomes in both cesarean and vaginal deliveries.  Further investigation should be conducted to further evaluate this relationship.

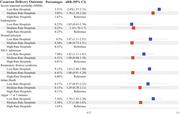

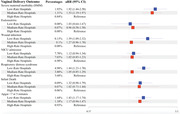

## 702 | Cost‐Effectiveness of Universal Screening for Measles Immunity in Pregnancy

Anuradha Devabhaktuni^1^; Sarah Boudova^2^; Elias Kassir^3^; Sohum Shah^4^; Neil Silverman^5^; Christina S. Han^1^

*^1^UCLA, Los Angeles, CA; ^2^Thomas Jefferson University, Philadelphia, PA; ^3^University of Texas Health Science Center at Houston, McGovern Medical School, Houston, TX; ^4^University of California, Los Angeles, Los Angeles, CA; ^5^David Geffen School of Medicine at UCLA, Department of Obstetrics and Gynecology, Division of Maternal‐Fetal Medicine, Los Angeles, CA*

10:30 AM ‐ 12:30 PM

**Objective**: Routine screening for measles immunity is not currently recommended in pregnancy. However, vaccination rates have declined and there have been over a dozen measles outbreaks in the United States in 2024. We aim to assess the cost‐effectiveness of universal screening for measles immunity in pregnancy.

**Study Design**: We conducted a cost‐effectiveness analysis comparing outcomes and costs associated with universal screening for measles immunity in pregnancy compared to no screening. We designed a Markov model that ran over 3 pregnancy cycles. Our outcomes included: measles exposure, measles infection including mild and severe infections, maternal death, fetal death, preterm delivery, vaccination, and vaccine response in addition to cost and quality‐adjusted life years (QALYs). Model inputs were derived from the literature, with a willingness‐to‐pay threshold of $100,000 per QALY. Sensitivity analyses were conducted to evaluate the robustness of the results.

**Results**: Universal screening for measles immunity was the dominant strategy. Using the current measles rate of 0.1%, the incremental cost effectiveness ratio (ICER) of universal screening versus no screening was $25.07/QALY. Tornado one‐way sensitivity analyses demonstrated that the costs of measles screening and cases, maternal death, and MMR vaccination, along with the probability of measles exposure had greatest impact on the cost‐effectiveness of the screening strategy. Univariate sensitivity analysis demonstrated that universal screening for measles was cost‐saving until the cost of testing for measles immunity passed $3,957, far exceeding the current average cost of screening, $44. When varying the measles exposure rate, screening remained the dominant strategy.

**Conclusion**: Universal screening for measles immunity during pregnancy was identified as a cost‐effective strategy supporting its implementation. Until adopted, providers should consider screening their pregnant patients and recommending postpartum vaccination to ensure protection from measles in a subsequent pregnancy.

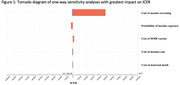

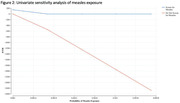

## 703 | Diagnostic Accuracy of Sonographic Fetal Weight Estimation for Intertwin Size Discordance

Arietta Vayenas^1^; Sophia Rahimi^2^; Nir Melamed^3^

*^1^Sunnybrook Health Sciences Centre, Toronto, OR; ^2^Temerty Faculty of Medicine, University of Toronto, Toronto, OR; ^3^Division of Maternal Fetal Medicine, Department of Obstetrics and Gynecology, Sunnybrook Health Sciences Centre, Toronto, ON*

10:30 AM ‐ 12:30 PM

**Objective**: Antenatal diagnosis of intertwin size discordance can affect management decisions. Therefore, care providers need to be aware of the accuracy of antenatal ultrasound for size discordance at birth. The current study aimed to estimate the diagnostic accuracy of estimated fetal weight (EFW) and abdominal circumference (AC) for large birthweight discordance and the diagnosis of selective fetal growth restriction (sFGR) at birth.

**Study Design**: Retrospective cohort study of twin pregnancies followed at a single center (2011‐2023) who had ultrasound within 14 days before birth. We compared the diagnostic accuracy of EFW discordance and AC discordance for birthweight discordance and the diagnosis of sFGR at birth.

**Results**: Of the 1,065 twin pregnancies included, the rate of birthweight discordance > 20% and > 25% was 15.5% and 6.7%, respectively. EFW discordance was more accurate than AC discordance in estimating birthweight discordance, as reflected by a lower systematic error and mean absolute percentage error (**Table**). Still, both EFW and AC discordance had low diagnostic accuracy for large birthweight discordance and sFGR. For example, EFW discordance >20% had a sensitivity of 49.1% and a positive predictive value of 57.0% for birthweight discordance >20%, and the antenatal diagnosis of sFGR had a sensitivity of 45.8‐54.4% and a positive predictive value of 50.9‐55.2% for the postnatal diagnosis of sFGR. A false positive diagnosis of large birthweight discordance was mainly the result of overestimation of EFW percentile in the larger twin (**Figure A**), while failure to detect large birthweight discordance was mainly the result of overestimation of EFW percentile in the smaller twin (**Figure B**).

**Conclusion**: EFW discordance is more accurate than AC discordance in estimating birthweight discordance. However, both antenatal measures have low diagnostic accuracy for large birthweight discordance and sFGR. Care providers should be aware of the limited diagnostic accuracy when making management decisions on the timing and mode of delivery.

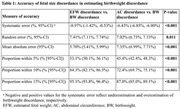

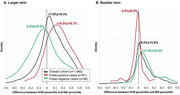

## 704 | Equity in First Trimester Congenital Anomaly Detection: Population Based Analysis in >1 Million Women in England

Jehan Karim^1^; Pranav Pandya^2^; Aris T. Papageorghiou^1^

*^1^University of Oxford, Oxford, England; ^2^University College Hospital, London, England*

10:30 AM ‐ 12:30 PM

**Objective**: As there is no unified policy for first trimester anatomical screening in England, our aim was to establish whether differences in screening methods impact on the timing of diagnosis of major congenital anomalies.

**Study Design**: We linked data from two sources: a nationwide survey of current first trimester practice distributed to all NHS maternity units in England (n = 131) that established current first trimester ultrasound practice; and data from the National Congenital Anomalies Disease Registry (NCARDRS) database. The proportion of major anomalies diagnosed prior to 16 weeks gestation was then evaluated based on current first trimester screening policy.

**Results**: We included 1,030,224 pregnancies including 5,598 fetuses with one of 14 major conditions. Overall, n = 1,929 (32.7%, 95% CI 31.5–33.9) of these anomalies were diagnosed < 16 weeks. The screening policy in the 110 responding was grouped into four first trimester anatomy screening policies: A. no routine evaluation (n = 27), B. basic (n = 22), C. intermediate (n = 45), and D. advanced assessment (n = 16). Early detection rates were lowest in Group A (27.7%, 95% CI 25.4–30.0) and an increase in detection was seen in Group B (31.2%,  95% CI 28.5–34.1), C (33.2%, 95% CI 31.3–35.2) and D (40.4%, 95% CI 37.3–43.4, p< 0.0001).

**Conclusion**: Most NHS hospitals in England offer women some form of first trimester anomaly screening. The absence of national recommendations means there are significant variations in practice, which are associated with differences in fetal anomaly detection rates prior to 16 weeks gestation at population level. Centres with clear protocols for first trimester fetal anomaly screening have higher early detection rates for major congenital anomalies. The differences in the standard of antenatal screening care offered to women across England has resulted in regional and local inequities in maternal care.

## 705 | Utilization of Genetic‐Counseling and Carrier‐Screening at a Single Center: A Quality Assessment Study

Maura Jones Pullins^1^; Mia Hodges^1^; Emily Hardisty^2^; Madeline Dyke^2^; Neeta L. Vora^2^; Asha N. Talati^2^

*^1^University of North Carolina, Chapel Hill, NC; ^2^University of North Carolina at Chapel Hill, Chapel Hill, NC*

10:30 AM ‐ 12:30 PM

**Objective**: To evaluate completion of basic carrier screening in a first pregnancy  (G1) and factors associated with receipt.

**Study Design**: Chart review of G1 pregnancies at a single center between 01/2017–01/2024. Charts were randomly selected for quality assessment and were manually reviewed for provider notes to assess documentation (offered, accepted, declined);  to review partner charts; and review neonatal charts for newborn screening  (NBS) results. Patients were excluded if they received preconception care, infertility services, transferred into care above 20 weeks, or prenatal records were not available. Screening for variants in Cystic Fibrosis (CF), Spinal Muscular Atrophy (SMA), and Hemoglobinopathies (HB) defined complete carrier screening. Participants that elected for expanded carrier screening (ECS) were noted. Data was analyzed using descriptive statistics, t‐test, and chi‐square as appropriate. Analyses were completed using Stata 15.

**Results**: 450 maternal charts were reviewed. 158 were excluded (as above). The final cohort included 292 maternal, paternal, and neonatal charts (Table 1). 109 (37.3%) had genetic counseling and 98 (33.6%) had complete carrier screening. Of those that had genetic counseling, 54% had carrier screening; those that declined had ‘declined’ documented in the clinical note 100% of the time. Obstetric care providers did not routinely document declining carrier screening; this was identified 6 times in obstetric provider notes. 129 (44.2%) had CF screening, 109 (37.2%) had SMA screening, and 178 (61%) were screened for HB. Eleven (4%) of participants had ECS. No carrier couples or abnormal NBS were identified. Frequency of complete paternal testing when a maternal screen was positive is shown in Table 2. There were no significant differences in complete carrier screening or use of genetic counseling services based on race or insurance status.

**Conclusion**: One‐third of randomly sampled G1 pregnancies since 2017 received complete carrier screening suggesting need to improve implementation and larger sample.

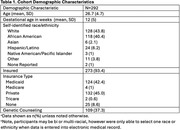

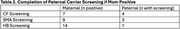

## 706 | Trends in Postpartum Sflt‐1 Among Patients Diagnosed with Preeclampsia with Severe Features

Ashten B. Waks^1^; Devesh Naidoo^2^; Dongbao Chen^1^; Megan C. Oakes^3^

*^1^University of California Irvine, Orange, CA; ^2^MemorialCare Miller Children's and Women's Hospital Long Beach, Long Beach, CA; ^3^MemorialCare Miller Children's and Women's Hospital Long Beach, Miller Children's and Women's Hospital, Long Beach, CA*

10:30 AM ‐ 12:30 PM

**Objective**: Because an excess of anti‐angiogenic proteins like soluble fms‐like tyrosine kinase 1 (sFlt‐1) relative to pro‐angiogenic proteins is thought to result in preeclampsia, placental delivery is regarded as curative for this condition. As there is little research on the impact of delivery on such placental proteins, this study sought to examine postpartum trends in sFlt‐1 among patients with preeclampsia.

**Study Design**: A prospective pilot study of pregnant persons without chronic hypertension delivered for preeclampsia with severe features between 7/2023–6/2024. Subjects had at least 2 postpartum sFlt‐1 levels drawn, the first within 24 hours of delivery and the second within 10 days of delivery. Primary outcome measures included change in sFlt‐1 over time and postpartum half‐life of sFlt‐1. The normality of distribution for these continuous outcomes was tested by Kolmogorov‐Smirnov test, and outcomes with normal distributions were presented as means while those with non‐normal distributions were presented as medians. Half‐life was calculated according to first‐order elimination principles.

**Results**: Among 20 subjects with preeclampsia with severe features, 16 (80%) had at least 2 postpartum sFlt‐1 levels drawn. Median sFlt‐1 was 9,304pg/mL (IQR 7,060–25,051pg/mL) and 508pg/mL (IQR 402–855pg/mL) on postpartum day 1 and 7, respectively. This translated to a median reduction in sFlt‐1 of 8,800 pg/mL (IQR 7,076–24,650pg/mL) and a median half‐life of only 1.51 days (IQR 1.13–1.73 days). These trends did not vary when assessed according to variables including maternal age, ethnicity, body mass index, medical comorbidities, antenatal aspirin exposure, gestational age at diagnosis, blood pressure at delivery, or need for antihypertensives postpartum (p > 0.32).

**Conclusion**: Even in patients with preeclampsia with severe features, serum sFlt‐1 levels declined rapidly over the first week postpartum. This suggests that factors other than clearance of abnormal placental proteins are likely responsible for persistent postpartum hypertensive disease.

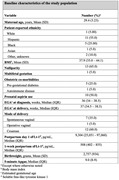

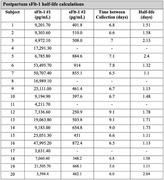

## 707 | Results of Diagnostic Cardiac Testing Among Pregnant Persons with Positive Cardiovascular Disease Screening

Ashten B. Waks^1^; Heike Thiel de Bocanegra^1^; Jenny Chang^1^; Omotayo Balogun^1^; Anna Grodzinsky^2^; Maryam Tarsa^3^; Afshan B. Hameed^4^

*^1^University of California Irvine, Orange, CA; ^2^University of Missouri‐Kansas City/Saint Luke's Hospital, Kansas City, MO; ^3^University of California San Diego, San Diego, CA; ^4^University of California, Irvine, Orange, CA*

10:30 AM ‐ 12:30 PM

**Objective**: Cardiovascular disease (CVD) is a significant cause of pregnancy‐related morbidity and mortality, and global CVD risk assessment is a key step in mitigating this trend. Our practice includes referring patients with a positive CVD risk assessment for further cardiac testing. This study aimed to establish the rate of abnormal cardiac test results among this group.

**Study Design**: A prospective cohort study of 3 academic medical centers using our CVD risk assessment between 2020–2024. Primary outcome was an abnormal composite of cardiac test results (e.g. abnormal brain natriuretic peptide (BNP), electrocardiogram (EKG), or echocardiogram) calculated as a percentage. Bivariate analyses with two‐sample t‐tests and chi‐square tests were performed to compare demographic and clinical characteristics between those with normal and abnormal results. Multivariate logistic regression was also fitted to evaluate the association between an abnormal test and patients' sociodemographic characteristics.

**Results**: A total of 16,900 patients had CVD risk assessment, of whom 334 (2%) had a positive and 16,655 (98%) had a negative result. Among those who screened positive, 207 (62%) went on to have diagnostic cardiac testing, including 117 BNP levels, 159 EKGs, and 81 echocardiograms. In this cohort, 78 (38%) had an abnormal composite cardiac test result, encompassing 14 (12%) abnormal BNP levels, 78 (38%) abnormal EKGs, and 2 (3%) abnormal echocardiograms. There were no significant differences between those with normal and abnormal composite cardiac test results with respect to age, ethnicity, insurance, medical conditions, symptoms, or total score on the CVD risk assessment (p > 0.05); however, in bivariate analysis, those with a heart rate above 110 were more likely to have an abnormal test (p = 0.0458).

**Conclusion**: Over one‐third of patients with a positive CVD risk assessment had abnormal findings on subsequent cardiac testing. As there was inconsistency in the cardiac tests ordered following a positive screening, further research is required to determine which testing strategies have the highest yield for future use.

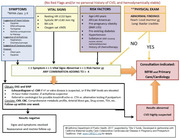

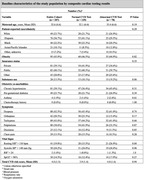

## 708 | Neonatal Outcomes in Maternal Heart Disease: A Multidisciplinary Approach toward Congenital and Acquired Conditions

Aura S. Eguizabal^1^; Yaiza Fernandez Munoz^2^; Ijeoma Iweakogwu^3^; Jiaqi Zhang^3^; Elizabeth B. Sherwin^3^; Karl‐Stephane Louis‐Jacques^4^; Elisa Padron^3^; Sabrina Montgomery^5^; Gabriela Briceno^6^; Alex Goldman^7^; Abha Khandelwal^8^; Katherine Bianco^3^

*^1^Stanford University, Stanford University School of Medicine, CA; ^2^University of California, Berkeley, University of California, Berkeley, CA; ^3^Stanford University, Palo Alto, CA; ^4^Meharry Medical College, Nashville, TN; ^5^Charles R. Drew University of Medicine and Science, College of Medicine, Inglewood, CA; ^6^Stanford University, Sunnyvale, CA; ^7^Northwestern University, Northwestern University, IL; ^8^Stanford University, Stanford, CA*

10:30 AM ‐ 12:30 PM

**Objective**: Maternal heart disease remains one of the leading causes of preventable pregnancy‐related deaths in the US. Limited data compares specific maternal and neonatal outcomes among this high‐risk population. Therefore, we sought to investigate how multidisciplinary clinical care affects neonatal outcomes.

**Study Design**: This was a cohort study of pregnancies (n = 466) affected by maternal cardiac diseases that received multidisciplinary care (MFM/Cardiology/OB/Anesthesia/Cardiac Surgery/Genetics) in a single tertiary center between 2012 and 2024. The study groups consisted of pregnancies with maternal acquired heart disease (AHD) (n = 242), maternal congenital disease (CHD) (n = 224), and pregnancies without any cardiac disease (n = 183). Neonatal outcomes—birth weight, gestational age, Apgar scores, and NICU admissions—across these groups were compared by Pearson Chi‐Square, Fisher Exact, and Kruskal‐Wallis rank sum tests. A pairwise comparison was conducted for significant differences. The significance level was set at alpha = 0.05.

**Results**: This study showed increased adverse neonatal outcomes in the heart disease cohort, especially within the maternal CHD group compared to the group without heart disease. Infants born to people with CHD or AHD had lower birth weights, shorter gestational ages, lower Apgar scores, and higher rates of NICU admissions compared to those without heart disease (Table 1) (p value < 0.01 for all comparisons). The proportion of small for gestational age infants born to people with CHD was significantly higher than those born to people with AHD (p  = 0.007). All other outcomes were similar in the CHD and AHD cohorts.

**Conclusion**: Maternal heart diseases are associated with an increased risk of adverse neonatal outcomes, necessitating a multidisciplinary approach in prenatal care and tailored strategies for CHD and AHD. In this study, infants born to people with CHDs had an increased risk for small for gestational age as compared to AHD, thus further studies are necessary to better understand this biological difference.

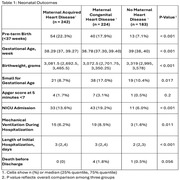

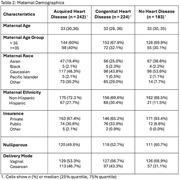

## 709 | Immediate Delivery versus Expectant Management in Twin Pregnancies with Late Preterm Rupture of Membranes

Avishag Abecassis^1^; Keren Zloto^2^; Chen Berkovitz^3^; Hadel Watad^4^; Shali Mazaki‐Tovi^5^; Michal Fishel Bartal^6^

*^1^Sheba Medical Center Tel Hashomer, Israel, Ramat Gan, HaMerkaz; ^2^Sheba Medical Center, Ramat Gan, HaMerkaz; ^3^Tel Aviv University Israel, Tel Aviv University Israel, HaMerkaz; ^4^Sheba Medical Center, Jatt Village, HaZafon; ^5^Department of Obstetrics and Gynecology, Sheba Medical Center, Tel HaShomer, Ramat Gan, HaMerkaz; ^6^UTH Houston & Sheba Medical Center Israel, Houston, TX*

10:30 AM ‐ 12:30 PM

**Objective**: In recent years, the management of late preterm premature rupture of membrane (PPROM) has changed from expedient delivery towards expected management. Current literature provides limited information on preferred management in twin pregnancy. We aimed to compare maternal and neonatal outcomes in twin pregnancies presented with PPROM at 34‐36 weeks.

**Study Design**:  

A retrospective study was conducted at a single tertiary care center between 2011 and 2024. All individuals with twin pregnancies who presented with PPROM between 34.0 to 36.0 weeks of gestation were included. We compared those who were dispositioned for immediate delivery versus those managed expectantly. Maternal and neonatal outcomes were evaluated using frequentist statistics.

**Results**: During the study period, 146 twin pregnancies presented with PPROM and met inclusion criteria; 37 (25.3%) were managed expectantly, and 109 (74.7%) were dispositioned for delivery. Baseline and pregnancy characteristics were similar between the groups (Table 1).  Expectant management was associated with a higher rate of intrapartum fever (8.1% vs. 0.9%, p = 0.05) and Cesarean delivery due to arrest of dilatation (13.5% vs. 1.8% p = 0.01) compared to immediate delivery. Furthermore, expectant management was associated with a higher rate of NICU admission (36.5% vs 23.9%, p = 0.03), respiratory distress syndrome (6.8% vs 1.4%, p = 0.01), and need for antibiotic treatment (63.5% vs. 28.9%, p< 0.01) compared to immediate delivery (Table 2).  

**Conclusion**: In our cohort, expectant management for individuals with twin pregnancies presenting with PPROM at 34‐36 weeks of gestation was associated with a higher rate of maternal and neonatal outcomes compared to immediate delivery.

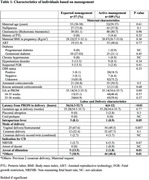

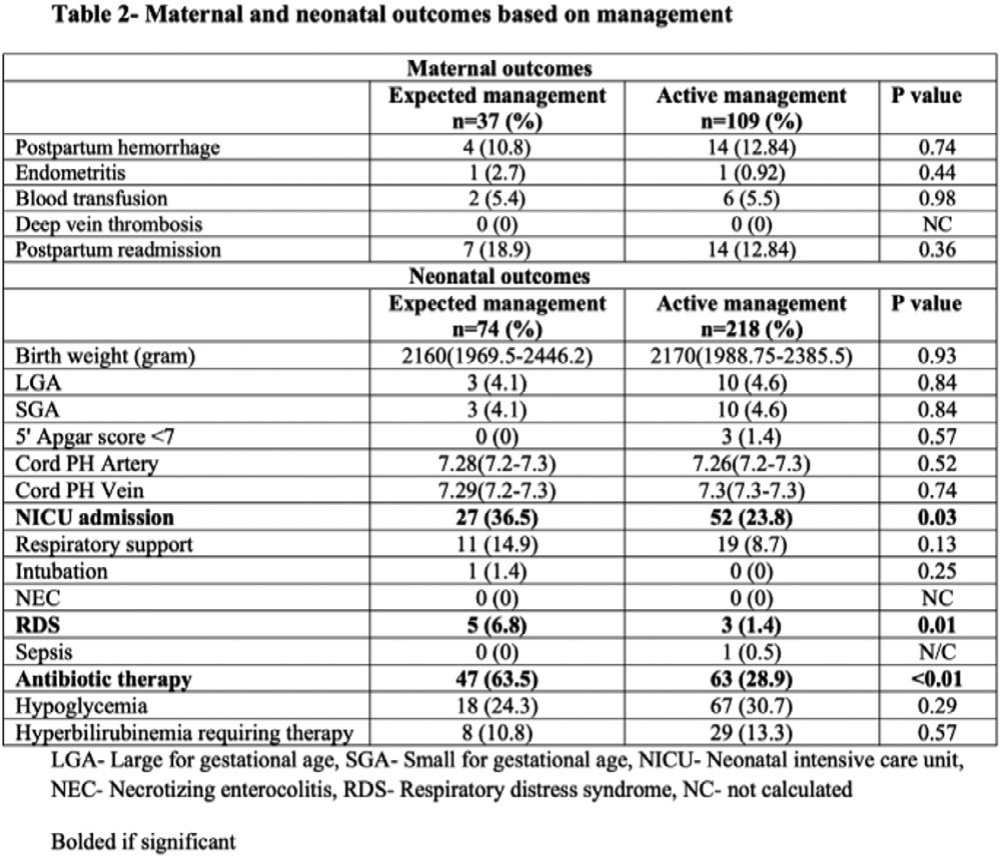

## 710 | Association of Glucagon Like Peptide‐1 Agonist (GLP1) use With Pregnancy Outcomes

Ayodeji Sanusi^1^; Yumo Xue^1^; Victoria C. Jauk^2^; Lynda Ugwu^2^; Charlotte B. McCarley^2^; Adrianna Tilton^2^; Ashley N. Battarbee^1^; Akila Subramaniam^1^; Alan T. Tita^2^; Rachel G. Sinkey^2^

*^1^Center for Women's Reproductive Health, University of Alabama at Birmingham, Birmingham, AL; ^2^University of Alabama at Birmingham, Birmingham, AL*

10:30 AM ‐ 12:30 PM

**Objective**: Treatment of obesity with GLP1 improves metabolic health and national use is rising since approval in 2017. As unintended pregnancy rates are >40%, GLP1 pregnancy exposures may be increasing. Data are limited on pregnancy outcomes after exposure. We quantified the risks of adverse pregnancy outcomes after GLP1 exposure and how duration of use modifies these associations.

**Study Design**: Retrospective cohort study of singleton deliveries at a single US center from 2017‐2024.  Pregnancies after GLP1 use (Semaglutide, Tirzepatide) were matched (1:many ratio) on BMI and demographics to unexposed pregnancies using propensity scores. The primary outcome was a composite of hypertensive disorders of pregnancy (HDP), fetal macrosomia or growth restriction. Secondary outcomes included fetal anomalies, NICU admission, mode of delivery and gestational weight gain (GWG). Regression models estimated the association between GLP1 use and study outcomes. Stratification by duration of use prior to pregnancy (>1yr, ≤1yr) was performed.

**Results**: Fifty‐eight exposed patients were matched to 1578 unexposed patients. Among 1636 patients, 86% were obese, 31% had pregestational diabetes and mean weight at first prenatal visit was 110±27kg. Forty‐five patients used GLP1 for ≤1 year. Mean weight change with GLP1 use until pregnancy was ‐0.11±6.44kg. There were no significant differences between groups after propensity score matching. The primary outcome occurred in 37(64.0%) exposed patients and 864 (54.8%) unexposed patients (OR 1.46, 95% CI 0.84‐2.51). Exposed patients had a higher odds of GWG (difference 2.36kg, 95% CI 1.43‐3.29) and NICU admission (OR 1.85, 95% CI 1.10‐3.13). Other secondary outcomes were similar between both groups.  In stratified analysis, NICU admission and GWG risks were significantly higher only with preconception use ≤1yr.

**Conclusion**: GLP1 use was not associated with HDP/fetal growth disorders. However, preconception use ≤1yr may have higher NICU admission/GWG risks. Future studies of weight trajectories by use duration and timing of discontinuation are needed.

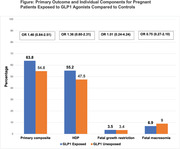

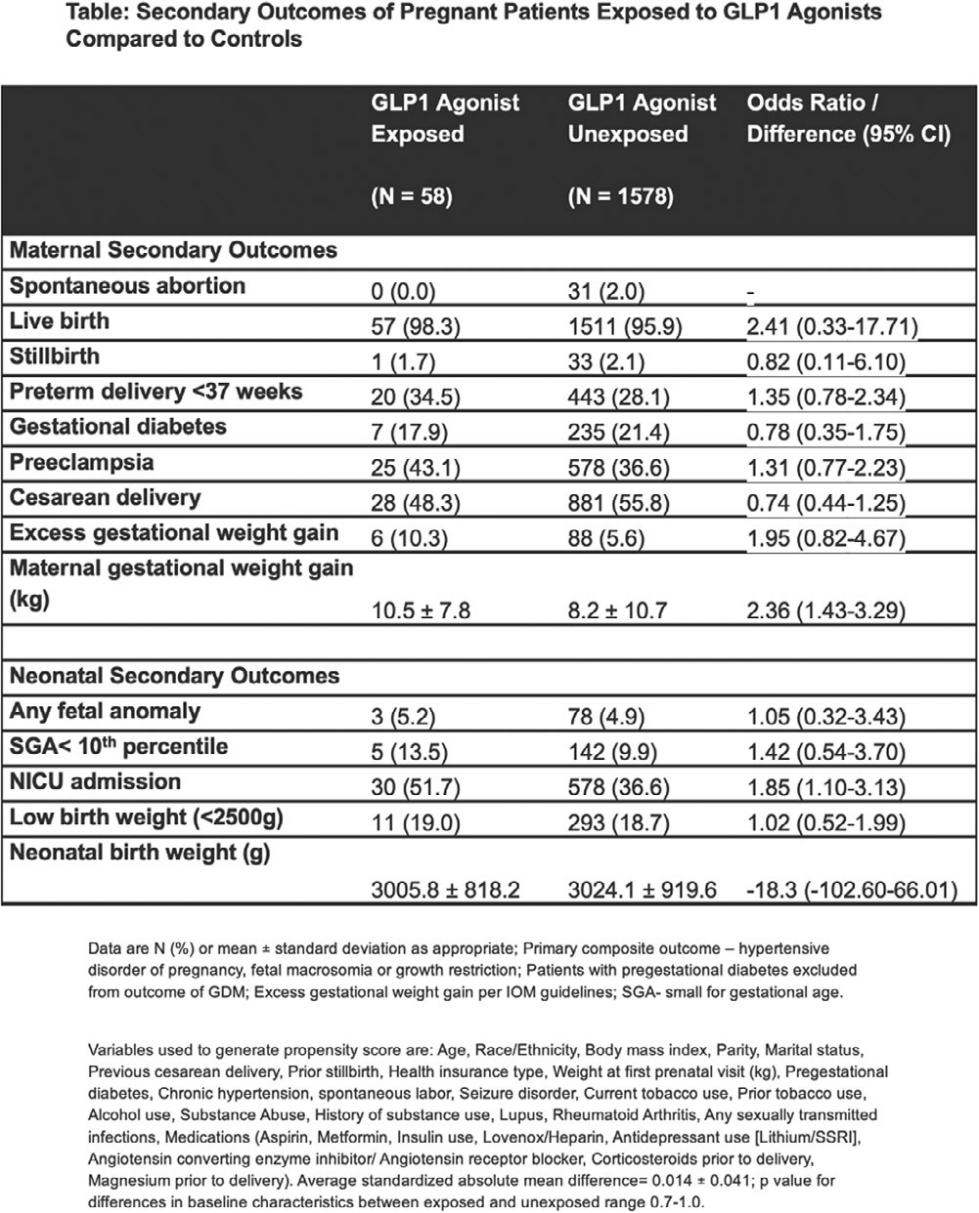

## 711 | Late Gestation Iuts Enable Later Delivery Without Adverse Outcomes in Fetuses with Alpha Thalassemia Major

Billie R. Lianoglou^1^; Evangelia Vlachodimitropoulou^2^; Akos Herzeg^3^; Tippi C. MacKenzie^4^; Greg Ryan^5^; Juan Gonzalez^1^

*^1^Center for Maternal‐Fetal Precision Medicine, University of California San Francisco, San Francisco, CA; ^2^Mount Sinai Hospital and University of Toronto, Toronto, ON; ^3^UCSF, San Francisco, CA; ^4^University of California, San Francisco, San Francisco, CA; ^5^University of Toronto, Toronto, ON*

10:30 AM ‐ 12:30 PM

**Objective**: To understand the impact of in‐utero blood transfusion (IUT) performed > 35 weeks’ gestation (WG) on perinatal outcomes in fetuses with alpha thalassemia major (ATM).

**Study Design**: UCSF established an international patient registry for patients affected by ATM which includes all patients referred to our institution and collaborating sites. We conducted a retrospective chart review and prospective enrollment of registry participants, including all singleton pregnancies with ATM who underwent IUTs and received their last IUT >32 WG. Data collected covered prenatal and postnatal outcomes, including details of IUTs. Patients were stratified into two groups based on the timing of the final IUT: **A**: 32‐35 WG (N = 17), **B**: > 35 WG (N = 10). Fisher's exact test and unpaired t‐test were used to assess the association between the gestational age (GA) of the final IUT and key outcomes such as survival rate, 5’ APGAR scores, the need for mechanical ventilation, prolonged neonatal hospitalization, GA at delivery, and time between last IUT and delivery.

**Results**: There were no differences between groups in the mean age at first IUT (A 25.2, B 23.8; P = 0.41) or the presence of hydrops upon presentation. Extension of IUTs beyond 35 weeks allowed for delivery later in gestation (A 37.4, B 38.3; P< 0.0001), with a shorter time interval between IUT and delivery (A 3.4, B 2.07; P = 0.01). There were no adverse outcomes associated with extended IUTs as evidenced by survival rate (A 100%, B 90%; P = 0.37), 5’ APGAR < 7 scores (A 88%, B 90%; P = >0.99), need for mechanical ventilation (A 24%, B 20%; P >0.99); there was a trend for decreased rates of prolonged hospitalization (A 53%, B 22%; P = 0.22).

**Conclusion**: Expert opinion recommends performing IUTs up to 35 WG, with subsequent planned delivery 2‐3 weeks later. However, our analysis indicates that IUTs performed beyond 35 WG are not associated with higher morbidity but allow for later GA at delivery without an additional risk of urgent delivery or other adverse perinatal outcomes.

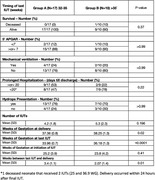

## 712 | Cost‐Effectiveness of Implementing a Multidisciplinary Team Approach the Management of Placenta Accreta Spectrum

Binh Luu^1^; Megha Arora^2^; Ava D. Mandelbaum^2^; Andrew H. Chon^2^; Tania F. Esakoff^3^; Deirdre J. Lyell^4^; Aaron B. Caughey^2^

*^1^Oregon Health and Science University, Vancouver, WA; ^2^Oregon Health & Science University, Portland, OR; ^3^Cedars Sinai Medical Center, Los Angeles, CA; ^4^Stanford University, Palo Alto, CA*

10:30 AM ‐ 12:30 PM

**Objective**: **Objective**: Placenta accreta spectrum (PAS) is associated with high maternal morbidity. Recent studies have shown that a multidisciplinary team approach to managing PAS can improve outcomes. This study aimed to evaluate the cost‐effectiveness of implementing a multidisciplinary team for the management of PAS.

**Study Design**: **Methods**: A decision‐analytic model was built using TreeAge software to compare the outcomes and cost‐effectiveness of the multidisciplinary team approach versus standard of care (i.e. without immediate availability of a gynecologic oncologist, anesthesiologist, etc.) The theoretical cohort included 5,000 individuals in the U.S. with PAS. Maternal outcomes included severe postpartum hemorrhage (>2000 mL estimated blood loss), adjacent organ injury, ICU admission, maternal death, and hospital readmission. The cost of the multidisciplinary team was estimated by assuming there would be specific MFM physician and nursing leadership assigned. Model inputs were derived from the literature. The cost‐effectiveness threshold was $100,000/quality‐adjusted life‐year (QALY). Sensitivity analyses were performed to assess the robustness of the results.

**Results**: **Results**: A multidisciplinary team approach for the management of PAS resulted in 3,724 fewer severe postpartum hemorrhages, 139 fewer adjacent organ injuries, 506 fewer ICU admissions, 87 fewer hospital readmissions within 6 months, and 1 fewer maternal death compared to standard of care (Table 1). When assuming a minimum baseline of 15 PAS cases per year, the multidisciplinary team approach was a dominant strategy resulting in a $8.8 million cost reduction and an increase of 59 QALYs relative to standard care. A one‐way sensitive analysis on the number of PAS cases per medical center demonstrated the multidisciplinary team strategy remains cost‐effective when centers treat ^3^ 8 PAS cases annually (Figure 1).

**Conclusion**: Implementing a multidisciplinary team was cost‐effective and improved maternal outcomes. These findings may inform interventions that promote multidisciplinary teams in the management of this high‐risk condition.

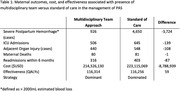

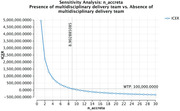

## 713 | Fetal Sex Influences the Maternal Plasma Proteome in Sars‐Cov‐2 Including Anti‐Viral Response

Bogdan Panaitescu^1^; Roberto Romero^2^; Nardhy Gomez‐Lopez^3^; Maria Fernanda Escobar^4^; Gaurav Bhatti^5^; Victor Tarca^6^; Tanay Panja^6^; Arun Mezzayhagan^1^; Awoniyi Awonuga^1^; Tinnakorn Chaiworapongsa^1^; David R. Bryant^1^; Adi L. Tarca^5^

*^1^Wayne State University School of Medicine, Detroit, MI; ^2^Pregnancy Research Branch, Division of Obstetrics and Maternal‐Fetal Medicine, Division of Intramural Research, Eunice Kennedy Shriver NICHD, NIH, DHHS, Bethesda, Maryland, USA, Bethesda, MD; ^3^Center for Reproductive Health Sciences, Washington University School of Medicine, St. Louis, MO; ^4^Universidad Icesi, School of Medicine, Cali, Valle del Cauca; ^5^Center for Molecular Medicine and Genetics, Wayne State University School of Medicine, Detroit, MI; ^6^Huron High School, Ann Arbor, MI*

10:30 AM ‐ 12:30 PM

**Objective**: Fetal sex influences physiological and pathological processes during pregnancy. This study was designed to determine the impact of fetal sex on the maternal response to SARS‐CoV‐2 infection using large‐scale proteomics.

**Study Design**: This was a prospective observational study of 101 pregnant patients, including 72 with confirmed SARS‐CoV‐2 infection and 29 controls without infection. Severity was assigned according to the NIH classification for COVID‐19. Large‐scale plasma proteomics was undertaken with the SomaScan platform, which analyzes 7,288 proteins. Fetal sex‐specific changes in the maternal proteome was assessed using linear models and moderated t‐tests while accounting for COVID‐19 severity and relevant clinical co‐variates.

**Results**: 1) In normal pregnancy, fetal sex did not alter the maternal plasma proteome; 2) SARS‐CoV‐2 infection during pregnancy exhibited a fetal sex‐dependent modulation of the plasma proteome; 3) pregnancies with a male fetus had a lower magnitude of plasma proteomic responses to SARS‐CoV‐2 infection compared to those with a female fetus (see figure; slope = 0.44, r = 0.61, p< 0.001); 4) the dampened maternal plasma proteomic response to infection in pregnancies with a male fetus was also noted according to disease severity; and 5) in severe COVID‐19, proteins showing fetal sex‐specific magnitude of change (interaction p< 0.05) were enriched for antiviral response pathways, endocytosis, and VEGF signaling,  among others (adjusted p< 0.1).

**Conclusion**: 1) Fetal sex dimorphism in the maternal proteome is not present in normal pregnancy, but in patients with COVID‐19; 2) pregnancies with male fetuses had a dampened proteomic response; and 3) these findings suggest that fetal sex is an important factor modulating the maternal response during SARS‐CoV‐2 infection and may determine disease progression and outcome.

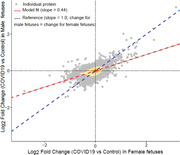

## 714 | Nanoparticle‐Based Fetal Intraperitoneal Injection is an Alternative to Intravascular Drug Delivery to the Pulmonary Endothelium

Braxton Forde^1^; Vladimir Kalinichenko^2^; Fatemeh Kohram^3^; Jose L. Peiro^4^; Alan Kenny^3^

*^1^University of Cincinnati College of Medicine, Cincinnati, OH; ^2^University of Arizona, Tempe, AZ; ^3^Cincinnati Children's Hospital Medical Center, Cincinnati, OH; ^4^Cincinnati Children's Fetal Care Center, Cincinnati, OH*

10:30 AM ‐ 12:30 PM

**Objective**:  Fetal intravascular injection of nanoparticle‐based therapy is proposed as a technique for targeted in‐utero therapies. However, in humans, umbilical cord access is limited both by gestational age and physician skillset, thus limiting the application of emerging therapies. Specifically in disorders of the fetal lungs, critical phases of development would be missed by waiting for the opportunity for umbilical cord access. Intraperitoneal transfusions have been used in early pregnancy when intravascular access is not feasible, therefore we sought to assess the feasibility of fetal intraperitoneal nanoparticle injections.

**Study Design**: Polyethylenimine‐(5) myristic acid/ poly(ethylene glycol)‐oleic acid/cholesterol (PEI‐PEG) nanoparticles were created as per prior publications and were tagged with fluorescent‐AF647 Dylight. Pregnant rats underwent midline laparotomy at E17.5‐E18.5 to fetal injection. Three arms were pre‐determined, either fetal intraperitoneal injection of nanoparticles, intra‐amniotic injection of nanoparticles, or sham. Fetuses were harvested at E20.5 and cells were immediately isolated and sorted via flow cytometry. As these nanoparticles localize to the pulmonary endothelium in newborn rodents, flow cytometry was used to evaluate pulmonary vascular endothelial cell uptake.

**Results**: Cell sorting successfully isolated fetal lung tissue and CD31+ CD45‐ cell lines were considered pulmonary vascular endothelium. Pulmonary endothelium of fetuses that underwent intraperitoneal injection were found to have 50.2% of cells positive for Dylight, vs 0.2% in fetuses that had intra‐amniotic injection and 0.0% in control (p < 0.001), Figure 1.  Every fetus that had an intraperitoneal injection had at least 42% of pulmonary endothelial cells with nanoparticles present, and no intra‐amniotic injection had greater than 1%.

**Conclusion**: Fetal intraperitoneal injection of PEI‐PEG nanoparticles successfully targets pulmonary endothelium. IP injections of PEI‐PEG nanoparticles provides a vehicle for targeted treatment of the fetal lungs early in gestation.

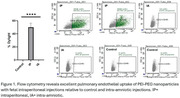

## 715 | The Association with Severe Lacerations and Increased Risk of Perinatal Depressive Symptoms

Brianna C. Sohl^1^; Mary F. Smith^2^; José Ibarra Rodriguez^2^; Nicole M. Hwang^2^; Samantha de los Reyes^3^

*^1^Rush University Medical Center, Justice, IL; ^2^Rush Medical College, Chicago, IL; ^3^Rush University Medical Center, Chicago, IL*

10:30 AM ‐ 12:30 PM

**Objective**: To evaluate the association of severe perineal lacerations and increased risk of perinatal depressive symptoms at the 6‐week postpartum visit.

**Study Design**: We performed a retrospective cohort study of patients who had a vaginal delivery between July 1, 2021‐July 1, 2023. Perineal lacerations were classified as either severe (3^rd^ or 4^th^ degree) or non‐severe (1^st^, 2^nd^ degree or intact perineum). Positive perinatal depressive symptoms were defined as a score of ≥10 on the Edinburgh Postnatal Depression Scale (EPDS) or positive response to question 10 at the 6‐week postpartum visit. Univariable analyses of demographic and clinical characteristics and positive EPDS scores at the 6‐week postpartum visit were performed. Multivariable logistic regression was performed using variables determined a priori (multiparity, gestational age (GA) at delivery, mode of delivery, race/ethnicity, exclusively breastfeeding at 6‐weeks postpartum) to evaluate the independent association of severe perineal laceration and positive EPDS scores at the 6‐week postpartum visit.

**Results**: 689 patients met inclusion criteria with 661 (95.9%) with non‐severe perineal lacerations and 28 (4.1%) with severe. In univariable analysis, those with severe perineal lacerations were more likely to be White non‐Hispanic or of Asian race/ethnicity, be multiparous, have a lower admission body mass index, have an operative assisted delivery, be delivered at a later GA, have greater blood loss, have a shoulder dystocia or episiotomy, and require a laceration revision within 6 weeks postpartum compared to those without (Table 1). Patients with severe lacerations were less likely to have a positive EPDS at 6‐weeks postpartum but these results were not significant (7.1 vs 9.9%, p = 0.66) and these findings persisted in adjusted analysis (aOR 0.62, 95%CI 0.13, 2.84).

**Conclusion**: Severe perineal lacerations was not associated with an increased risk of perinatal depressive symptoms at the 6‐week postpartum visit.

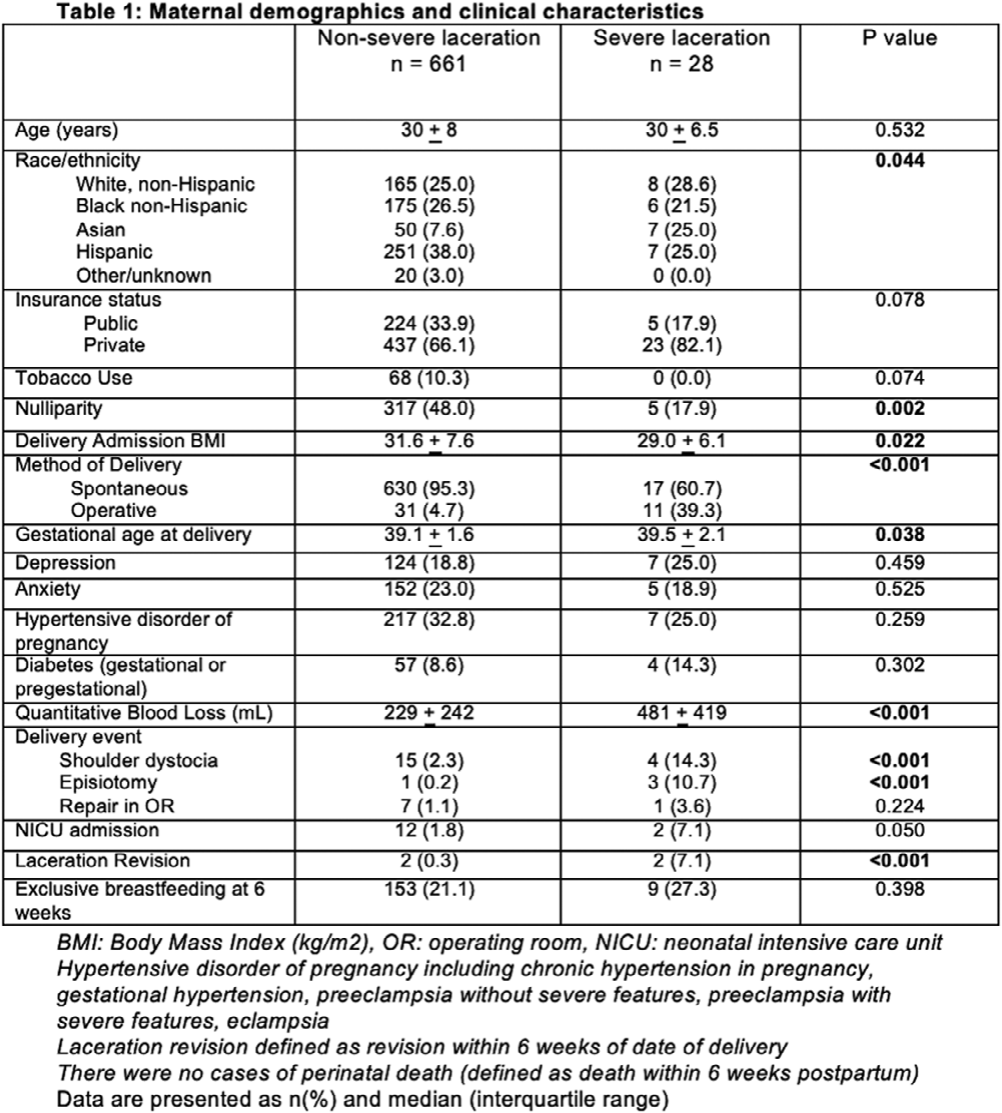

## 716 | Monochorionic Twins with Selective Fetal Growth Restriction: characteristics, course, management, and outcomes by type

Camille Shantz^1^; Laura Tan^2^; Christina Rivera^2^; Amelia Jiang‐Yu^2^; Lindsey Herlands^3^; Michelle Kush^3^; Jena L. Miller^3^; Ahmet A. Baschat^3^; Mara Rosner^3^

*^1^Johns Hopkins University, Baltmore, MD; ^2^Johns Hopkins University, Baltimore, MD; ^3^Johns Hopkins Medicine, Baltimore, MD*

10:30 AM ‐ 12:30 PM

**Objective**: To compare characteristics, Doppler course, and outcomes in Type I, II, and III selective fetal growth restriction (sFGR).

**Study Design**: Single‐center retrospective review of monochorionic twins with sFGR from 6/2014 to 1/2024. Twin to Twin Transfusion Syndrome, high order multiples, and severe fetal anomalies were excluded. Type at diagnosis was assigned by Gratacos criteria. Doppler course (deterioration or improvement) was defined as progression of umbilical artery (UA) end diastolic flow patterns from best to worst: persistent forward →intermittent forward, absent →intermittent forward, absent, reversed→persistent absent→persistent reversed. Ultrasound characteristics at presentation, antenatal course, and obstetric outcomes were compared between groups via Kruskal‐Wallis and Pearson Chi‐Squared.

**Results**: Of 199 patients, 133 (67%) were Type I, 18 (9%) Type II, and 48 (24%) Type III. There was no difference in gestational age (GA) at diagnosis. The estimated fetal weight discordance was lowest in Type I and highest in Type II (p< 0.001). UA Doppler deterioration was frequent in all groups and occurred in 28.4% of Type 1, 76.9% of Type II, and 51.4% of Type III (P< 0.001).  UA Dopplers also improved in some Type II (15.4%) and Type III (25.7%, p = 0.449). Management varied but was expectant for most (178/199, 89%) though 7 (4%) underwent laser (6/7 had Twin Anemia Polycythemia Sequence), 12 (6%) had selective reduction of the small fetus, and 2 (1%) terminated. Double survival was 89.6% in Type I, 25% in Type II, and 77.8% in Type III (p< 0.001). There was no difference in delivery GA between types, however, the majority of Type II were delivering a single fetus, and amongst all patients, those with 1 vs. 2 livebirths delivered later (36.1 [31.7‐38.7] vs. 33.1 [30.9‐34.3] weeks, p = 0.002). (Table 1)

**Conclusion**: Doppler deterioration is common in all Types of sFGR. Types II and III can improve in some cases and can be successfully managed to achieve favorable delivery gestational ages, however double survival is significantly lower in Type II.

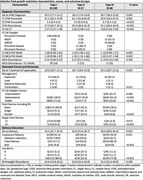

## 717 | Effect of Dental Treatments on Reduction of Preterm Birth: a Systematic Review and Meta‐Analysis

Camille Thomas^1^; Shakil Ahmed^2^; Rohan D'Souza^2^; Vincenzo Berghella^3^; Romina Brignardello‐Petersen^2^; Mohamed El‐Rabanny^2^; Catherine Devion^4^; Stefania Ronzoni^5^

*^1^University of Toronto, Toronto, ON; ^2^McMaster University, Hamilton, ON; ^3^Sidney Kimmel Medical College of Thomas Jefferson University, Philadelphia, PA; ^4^Sunnybrook Health Sciences Centre, Toronto, ON; ^5^Ontario Fetal Center and Mount Sinai Hospital, Toronto, ON*

10:30 AM ‐ 12:30 PM

**Objective**: This study aims to assess the effectiveness of treating dental disease during pregnancy in reducing the risk of preterm birth.

**Study Design**: We conducted a systematic review and meta‐analysis of randomized controlled trials (RCTs). We searched EMBASE, MEDLINE, PubMed, and Cochrane Central Register of Controlled Trials from inception to December 2023 with no language restrictions for RCTs enrolling pregnant persons with any dental disease who were randomized to receiving dental treatment versus a control. Pairs of independent reviewers screened studies, and abstracted and assessed the risk of bias of the studies using the Cochrane Risk of Bias tool, RoB 2. We conducted meta‐analysis using a random effects model with the Mantel‐Haenszel variance estimate. We assessed the certainty of evidence using the Grading of Recommendations Assessment, Development, and Evaluation (GRADE) approach.

**Results**: We included 19 RCTs, enrolling 8526 participants. The risk of bias for most of the RCTs ranged from some concerns to high risk. The most frequent interventions addressed were scaling and root planing (SRP) with oral hygiene instructions (OHI, 10 RCTs), and SRP with OHI and chlorhexidine mouthwash (6 RCTs). Meta‐analysis found low‐quality evidence suggesting that dental treatment results in a 22% relative risk reduction of preterm birth (RR 0.78; 95% CI 0.63‐0.95) when compared to placebo or control, which was usually minimal dental treatment or oral examination only (absolute difference 29 fewer preterm births per 1000 individuals; 95% CI from 48 fewer to 6 fewer).

**Conclusion**: This systematic review suggests that treating dental disease during pregnancy reduces the risk of preterm birth. The main limitations of the evidence are risk of bias and inconsistency. Additional well‐powered RCTs at low risk of bias are necessary to increase the certainty about the effect of dental disease treatments during pregnancy on reduction of preterm births.

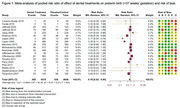

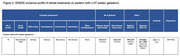

## 718 | Perinatal Outcomes of Resolved Fetal Cystic Hygromas

Carmen MA Santoli^1^; Emma EH Peek^2^; Lucas C. Collins^3^; Teresa N. Sparks^4^; Jeffrey A. Kuller^3^; Sarah K. Dotters‐Katz^3^

*^1^University Of California San Francisco, San Francisco, CA; ^2^University of Colorado, Aurora, CO; ^3^Duke University School of Medicine, Durham, NC; ^4^Center for Maternal‐Fetal Precision Medicine, University of California San Francisco, San Francisco, CA*

10:30 AM ‐ 12:30 PM

**Objective**: We described the perinatal phenotype of pregnancies with resolved fetal cystic hygroma and compared outcomes among fetuses with and without genetic abnormalities.

**Study Design**: A retrospective cohort study (2013‐2023) at a single tertiary center identified fetuses with cystic hygroma in the first or second trimester that resolved on subsequent ultrasound imaging. Pregnancies resulting in live birth with prenatal and delivery data were included. We describe perinatal characteristics, delivery outcomes, and genetic diagnoses. Bivariate statistics compared outcomes among fetuses with and without genetic abnormalities. A p‐value < 0.05 was considered statistically significant.

**Results**: Of 297 fetal cystic hygromas, we identified 59(20%) fetuses with cystic hygromas that resolved on subsequent imaging and resulted in live birth. Of these, 46 pursued prenatal and/or postnatal genetic diagnosis; 31(67%) had normal results and 15(33%) had a genetic abnormality, such as aneuploidy(n = 10), copy number variant(n = 2), or single‐gene disorder(n = 3).(Figure) Compared to fetuses with normal genetic testing, fetuses with genetic abnormalities were more likely to have a later gestational age at cystic hygroma diagnosis(12.3 vs 11.6 weeks, p = 0.007) and resolution(20.1 vs 17.3 weeks p = 0.006).(Table) Compared to fetuses with normal genetic evaluations, fetuses with genetic abnormalities were more likely to have additional structural anomalies on ultrasound(67% vs 13%,p = 0.001). The most common ultrasound findings were cardiac anomalies, followed by abdominal and renal abnormalities. Fetuses with resolved cystic hygromas and genetic abnormalities were more likely to have growth restriction, an earlier delivery, longer length of neonatal stay, and neonatal complications.

**Conclusion**: Fetuses with resolved cystic hygromas and genetic abnormalities were more likely to have other structural anomalies and obstetric complications when compared to those with normal results of genetic testing. These data are important for framing expectations and counseling of patients who experience resolution of fetal cystic hygroma.

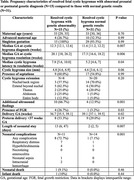

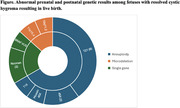

## 719 | Antenatal Characteristics Predict Need for Dental Referral Using the ACOG‐Recommended Oral Health Screening Tool

Carolina Martinez‐King; Charlie Rose; Annika Sahota; Krystle M. Perez; Tommy J. Yang; Shruti Kulkarni; Sophia Tibayan; Kanika Nallaseth; Abdul Hameed; Kathryn J. Gray; Tommy Wood; Gregory C. Valentine

*University of Washington, Seattle, WA*

10:30 AM ‐ 12:30 PM

**Objective**: The American College of Obstetricians and Gynecologists (ACOG) recommends screening pregnant patients with a 3‐question survey at their first prenatal visit for oral health. We sought to evaluate which characteristics are associated with an overall positive screen (defined as a positive response on any of the three questions) or with positive screen for each specific question.

**Study Design**: We screened pregnant individuals at a university‐based prenatal clinic using the 3‐question ACOG screening tool (Figure 1). We performed generalized linear models with stepwise forward AIC regression to evaluate antenatal characteristics (Table 1) with a positive overall screen and each specific question including adjusted odds ratios per included variable. Missing data for binary risk factors was imputed assuming the predictor was unknown. The dataset was divided 80:20 for model development and validation, respectively.

**Results**: Among 817 patients that completed the ACOG screen, 44.9% screened positive (367/817) with 14.9% (122/817) screening positive on question 1, 37.1% (303/817) on question 2, and 5.8% (48/817) on question 3. The models predicted 89.5%, 78.0%, and 93.2% accuracy with a positive screen for questions 1, 2, or 3, respectively. The model also predicted a positive overall screen with 66.7% accuracy. Antenatal factors with >1.5‐fold increased odds of a positive overall screen (Figure 1A) and symptomatic oral disease (Figure 1B) were Medicaid insurance, self‐identifying as Hispanic, having COVID, or having substance abuse during pregnancy.

**Conclusion**: Our models using antenatal characteristics can accurately (≥89%) predict symptomatic oral disease in pregnancy. Medicaid insurance, self‐identifying as Hispanic, having COVID or substance abuse during pregnancy were strongly associated with increased odds of having symptomatic oral disease or needing referral to a dentist underscoring that health disparities are present related to oral care in pregnancy. Antenatal efforts improving access to oral health in pregnancy are needed to reduce health disparities.

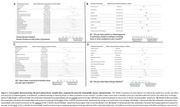

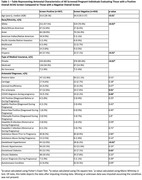

## 720 | Severe Maternal Morbidity Surveillance in Maryland: Trends Observed between 2021‐2023

Carrie Wolfson^1^; Robert Atlas^2^; Megan Carey^3^; Jan Chiang^3^; Pamela Chin^2^; Cathy Downey^4^; Robyn Duafala^5^; Ernest Graham^6^; Clark Johnson^3^; Monica Jones^3^; Carole Louis^5^; Ichchha Madan^4^; Donna Neale^4^; Joanne Olaku^7^; Michelle Phillips^6^; Jeanne Sheffield^6^; Danielle Silldorff^5^; David Silverman^8^; Rhoda Vandyck^3^; Andreea A. Creanga^9^

*^1^Johns Hopkins Bloomberg School of Public Health, Baltimore, MD; ^2^Mercy Medical Center, Baltimore, MD; ^3^Luminis Health Anne Arundel Medical Center, Annapolis, MD; ^4^Johns Hopkins Howard County Medical Center, Columbia, MD; ^5^Sinai Hospital of Baltimore, Baltimore, MD; ^6^Johns Hopkins School of Medicine, Baltimore, MD; ^7^Sinai Hospital of Baltimore, Sinai Hospital of Baltimore, MD; ^8^Sinai Hospital of Baltimore, Baltiomre, MD; ^9^Johns Hopkins University, Baltimore, MD*

10:30 AM ‐ 12:30 PM

**Objective**: Maryland implemented facility‐based severe maternal morbidity (SMM) surveillance and review in 2020 following AGOG/SMFM guidance. This study aims to analyze trends in primary cause of SMM, preventability and contributing factors. We also examine policy and practice changes that have resulted from the program.

**Study Design**: Data are from SMM surveillance for 2021‐2023 among the 5 hospitals that participated over the full period (level III & IV birthing facilities in rural and urban settings). Using X^2^ tests, we analyzed changes in primary cause of SMM, patient characteristics, and contributing factors. We further examined policy and practice changes that resulted from SMM reviews.

**Results**: Of 428 SMM events identified and reviewed, 80%, 78%, and 86% of patients had preexisting comorbidities in 2021, 2022, and 2023, respectively (p = 0.24). A growing proportion of patients did not receive prenatal care (1.6% in 2021, 5.4% in 2022, and 8.7% in 2023; p = 0.035). Obstetric hemorrhage was the leading cause (>50%) of SMM across all 3 years; however, the distribution of other causes varied (Table 1). COVID‐19 was the second primary cause of SMM in 2021 (18%), but resulted in only 3 SMM events in 2022‐23. Over the 3‐year period, review committees determined that 37% (increasing from 34% to 40% between 2021 and 2023) of SMM events were preventable with changes to one or more provider, system, or patient factor. System and patient factors were identified in a larger percentage of preventable SMM events in 2023 than 2021‐2022, while the contribution of provider factors did not change significantly (Table 2). In response to SMM reviews, all participating hospitals made specific policy and practice changes to address factors that contributed to leading causes of preventable SMM.

**Conclusion**: 40% of SMM events were deemed preventable by review committees in 2023. With hemorrhage being the leading cause of SMM, participating hospitals introduced policy and practice changes targeting hemorrhage and implementation of AIM's Obstetric Hemorrhage bundle is underway in Maryland.

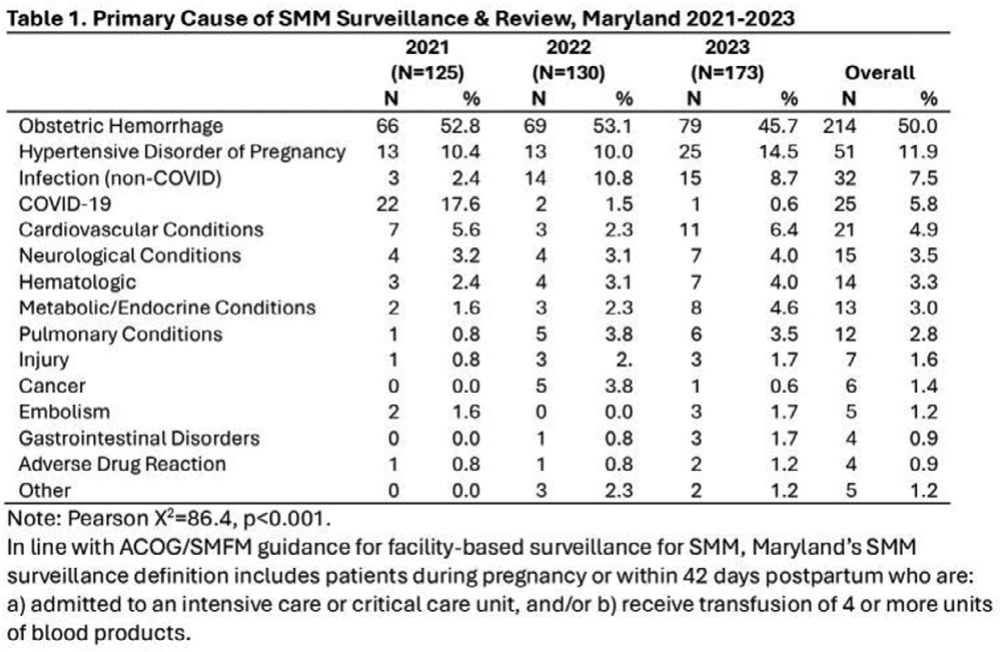

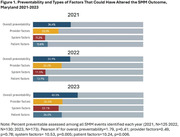

## 721 | Urine Culture Speciation During Pregnancy and Association with Maternal Attributes and Neonatal Outcomes

Chaarushi Ahuja^1^; Lisbet S. Lundsberg^2^; Jennifer F. Culhane^2^; Caitlin Partridge^2^; Mert Ozan Bahtiyar^3^; Katherine Kohari^2^

*^1^Yale New Haven Hospital, New Haven, CT; ^2^Yale School of Medicine, New Haven, CT; ^3^Yale University, New Haven, CT*

10:30 AM ‐ 12:30 PM

**Objective**: Urine culture(UC) is commonly ordered during pregnancy for urinary tract infection(UTI) symptoms or abdominopelvic pain. Little is known about the positivity rate of UC during pregnancy or associated maternal and neonatal outcomes. We aimed to assess the rate of positivity of UCs, and to assess maternal attributes and neonatal outcomes associated with different speciations.

**Study Design**: This is a retrospective study of pregnant patients who had a UC performed in our hospital system in non‐ambulatory settings from 2013‐2022. Patients with UCs were classified as 1) always negative (< 10k colony forming units‐CFU), 2) ever positive for GBS (>10k CFU) or UTI‐causing microbes (>100k CFU) or 3) ever positive for non‐UTI microbes (>100k CFU). Maternal characteristics of age, insurance, race, parity, language, BMI, chronic hypertension(cHTN), hypertensive disorders of pregnancy(HDP), gestational diabetes(GDM), and pregestational diabetes were compared between the 3 groups using chi‐square or ANOVA. Pregnancy outcomes of preterm birth(PTB), small for gestational age(SGA), mode of delivery, and neonatal intensive care unit(NICU) admission were also compared.

**Results**: 19,698 patients had a UC performed. The ever positive rate for GBS/UTI was 3.3% in patients with 1 UC, increasing to 18% among patients with ≥7 UC(Fig 1). Statistically significant differences across groups were noted in age, insurance, race, parity, language, BMI, and smoking(Table 1). There were also significant differences for cHTN, HDP, pregestational diabetes, PTB, NICU admission and SGA (Table 1). There was no statistical difference between groups for mode of delivery.

**Conclusion**: This is one of the first studies to classify rate of positivity by UC speciation during pregnancy. Maternal attributes were significantly different between groups, suggesting a need for further exploration of the basis of these differences. Surprisingly, PTB was more common in the always negative UC group compared to the positive UC groups. Thus, more studies are needed to understand relationships between positive UC, sociodemographics, and obstetric outcomes.

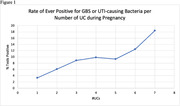

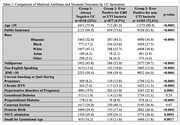

## 722 | Progesterone Supplementation in Preeclamptic Women Reduces Blood Pressure and Inflammatory Mediators of Hypertension During Pregnancy

Charles H. Brewerton; Christy Chambers; Richard Rein; Nathan Campbell; Baoying Zheng; Ty Turner; Montianah Roseburgh; Babbette LaMarca; Sheila Belk; Cameronne Dodd; Lorena M. Amaral

*University of Mississippi Medical Center, Jackson, MS*

10:30 AM ‐ 12:30 PM

**Objective**: **Introduction**: Preeclampsia (PE), new onset hypertension after 20 weeks of gestation, is associated with lower progesterone, increased CD4+T cells, inflammatory cytokines, and autoantibodies to angiotensin II type 1 receptor (AT1‐AA). We have previously shown that placental PE CD4+ T cells cause a PE phenotype in athymic nude rats. Moreover, we have shown that 17‐hydroxyprogesterone caproate (17‐OHPC), lowers blood pressure in PE patients, however, it is unclear if this was mediated via a T cell effect.

**Objective**: Determine if placental CD4+ T cells from 17‐OHPC treated PE women increase blood pressure in athymic nude recipient rats.

**Study Design**: PE women received 17‐OHPC (250 mg, I.M.) with blood draws before and after injection. One million placental CD4+ T cells from normal pregnant (NP) or PE participants were isolated by magnetic beads and injected I.P. into pregnant nude athymic rats on gestational day (GD) 12. On GD18, carotid catheters were inserted. On GD19, mean arterial blood pressure (MAP), blood and tissues were collected. One‐way ANOVA was used for statistical analysis.

**Results**: Participants had matched GA. Maternal blood pressure was 116 +/‐4 mmHg in NP women (n = 8), 147 +/‐ 4 mmHg in PE women (n = 19) and reduced to 136 +/‐ 3 mmHg in PE+17‐OHPC women (n = 14, p< 0.05). AT1‐AAs were 23 +/‐ 5 beats per minute in PE (n = 4) and 10 +/‐2 in PE+17‐OHPC (n = 8; student t‐test, p< 0.05). Placental CD4+T cells were 2+/‐ 1% gate in NP, 6 +/‐ 1 in PE, and 3 +/‐1 % gate in PE+17‐OHPC (n = 4‐5). Circulating TNF‐α was 21 +/‐ 4 pg/mL in NP, 41 +/‐8 in PE (p< 0.05), 21+/‐5 pg/ml in PE+17‐OHPC (n = 6‐8, p< 0.05). In pregnant nude recipient rats, MAP was 98 +/‐ 2 mmHg with NP CD4+ T cells (n = 5), 122 +/‐7 (n = 7, p< 0.05) with PE CD4+ T cells (n = 7) and 102+/‐ 5 with PE+17‐OHPC CD4+ T cells (n = 7, p< 0.05).

**Conclusion**: 17‐OHPC in PE women reduces blood pressure and inflammatory mediators of hypertension during pregnancy. Moreover, PE CD4+ T cells treated with 17‐OHPC do not mediate a PE phenotype in recipient pregnant rats.

## 723 | Delivery and Neonatal Outcomes of Resolved Vasa Previa Cases

Chiara Corbetta‐Rastelli^1^; Molly Zeme^1^; Rachel Mundaden^1^; Mary E. Norton^2^; Kate Swanson^3^

*^1^University of California San Francisco, San Francisco, CA; ^2^Center for Maternal‐Fetal Precision Medicine, University of California San Francisco, San Francisco, CA; ^3^University of California, San Francisco, San Francisco, CA*

10:30 AM ‐ 12:30 PM

**Objective**: Recent expert opinion has questioned the definition of vasa previa. We examined delivery and neonatal outcomes in cases of resolved vasa previa, comparing fetal vessels 2‐5cm from the cervical os versus >5cm away from the os.

**Study Design**: Retrospective cohort study of patients diagnosed with vasa previa at a single academic urban institution between 2004 to 2024. We conducted an electronic chart review, including ultrasound images, to obtain variables at diagnosis and throughout pregnancy, along with delivery and neonatal outcomes. We used descriptive and chi‐square statistics.

**Results**: We identified 112 pregnancies with vasa previa, of which 31 (28%) resolved on a subsequent ultrasound. Of the 31 resolved vasa previa cases, 20 were 2‐5cm from the os and 11 were >5cm. Of those with vessels 2‐5cm from the cervical os, 9 (45%) delivered by scheduled cesarean and 11 (55%) had a trial of labor (TOL). Of those that had a TOL, five (45%) delivered vaginally and six (55%) had a cesarean delivery due to PPROM (n = 2), fetal intolerance of labor (n = 1), arrest of descent (n = 1), unstable lie (n = 1) and worsening pre‐eclampsia (n = 1). Of those with vessels >5cm from the os, one (9%) delivered by scheduled cesarean and 10 (91%) had a TOL, of which 8 (80%) delivered vaginally. The only emergent cesarean delivery and neonatal blood transfusion occurred in a patient with fetal vessels >5cm from the os; the suspected etiology was placental abruption. There were no statistically significant differences in neonatal APGAR scores, umbilical artery pH and intensive care nursery admissions between groups. There were no perinatal deaths.

**Conclusion**: The majority of resolved vasa previa cases with vessels 2‐5cm from the cervical os delivered by cesarean. While many were scheduled, there were urgent cesareans for PPROM, unstable lie, arrest of descent, worsening pre‐eclampsia and fetal intolerance. Most cases with vessels >5cm from the os delivered vaginally. Larger studies are needed to further assess delivery and neonatal outcomes of resolved vasa previa.

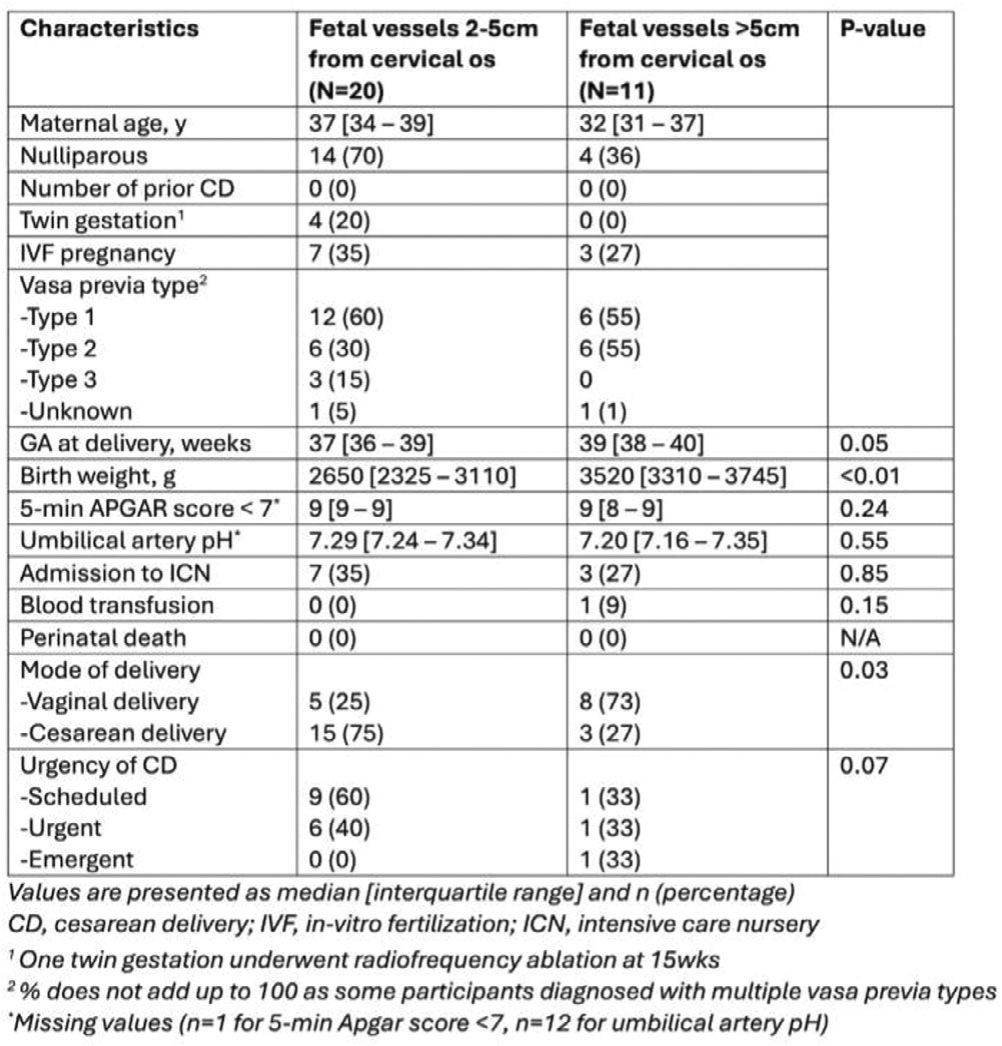

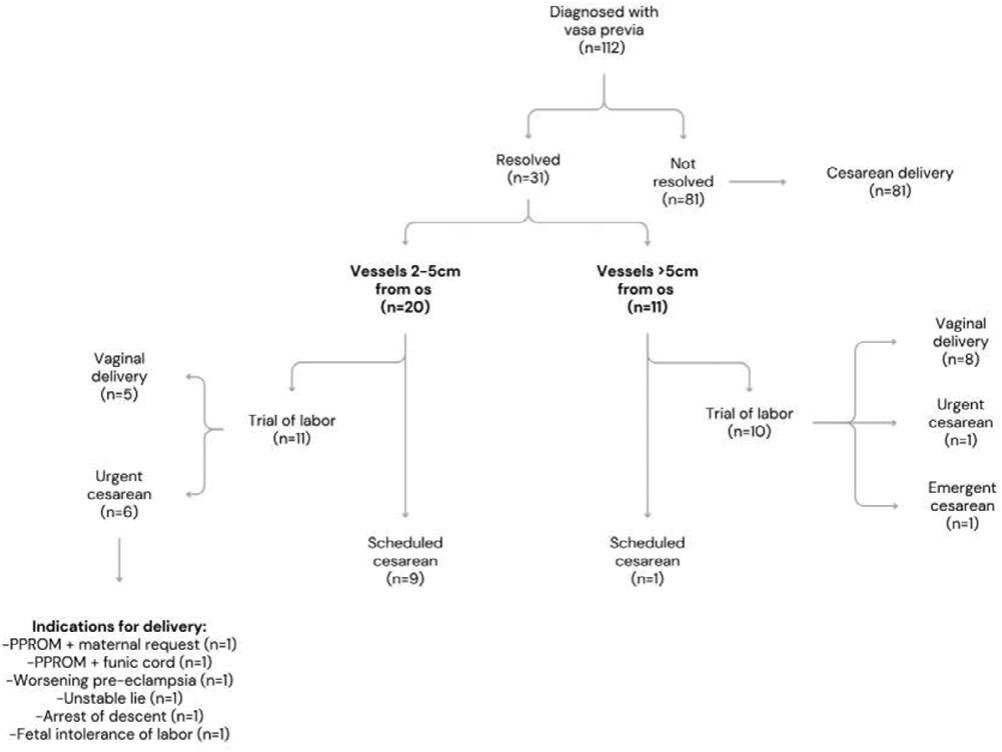

## 724 | Metabolic Recovery at 12 Months Postpartum Among Individuals with Glucose Intolerance in Pregnancy

Christina M. Scifres^1^; Arunabh Sinha^1^; Kaleab Abebe^2^; Christina Lalama^2^; Tina Costacou^2^; Steve Orris^2^; Patrick Catalano^3^; Esa Davis^4^

*^1^Indiana University School of Medicine, Indianapolis, IN; ^2^University of Pittsburgh School of Medicine, Pittsburgh, PA; ^3^Tufts University, Tufts University/Boston, MA; ^4^University of Maryland School of Medicine, Baltimore, MD*

10:30 AM ‐ 12:30 PM

**Objective**: Glucose intolerance in pregnancy is associated with long‐term risk for type 2 diabetes (T2D). We evaluated metabolic characteristics and β‐cell function during pregnancy and at 12 months postpartum among varying levels of glucose intolerance in pregnancy.

**Study Design**: This is a planned follow‐up to the Gestational Diabetes Diagnostic Methods (GDM2) trial, which randomized pregnant individuals to either a 75‐gram oral glucose tolerance test (OGTT) with GDM diagnosed using the IADPSG criteria, or a 100g OGTT with GDM diagnosed by the Carpenter‐Coustan (CC) criteria. All participants with treated GDM (diagnosed by either CC or IADPSG), those with untreated mild glucose intolerance (MGI, one abnormal value on CC criteria), and half of the participants with normal glucose tolerance were invited for a 75g OGTT at 12 months postpartum. Measures assessed at the time of GDM screening and at follow‐up included metabolic characteristics, Stumvoll and Matsuda Indices to evaluate insulin sensitivity and resistance, and the Disposition Index (DI), which integrates insulin sensitivity and response.

**Results**: Of the 407 individuals seen at 12 months, 49 (12%) had MGI and 53 (13%) had treated GDM (CC and IADPSG). MGI was associated with lower insulin sensitivity, lower beta cell function, dyslipidemia, and alterations in leptin and adiponectin similar to those individuals with treated GDM (Table). Measures of metabolic function, insulin sensitivity and β‐cell function demonstrated similar rates of change from pregnancy to postpartum after adjusting for maternal age, BMI, and history of GDM.

**Conclusion**: Patients with MGI have impaired β‐cell function and significant metabolic abnormalities at 12 months postpartum similar to individuals with treated GDM and require ongoing follow‐up for progression to T2D. The similar rate of change from pregnancy to postpartum in insulin sensitivity, β‐cell function, and metabolic assessments among groups indicates that individuals were returning to their baseline levels of glucose tolerance rather than recovering from pregnancy‐induced glucose intolerance.

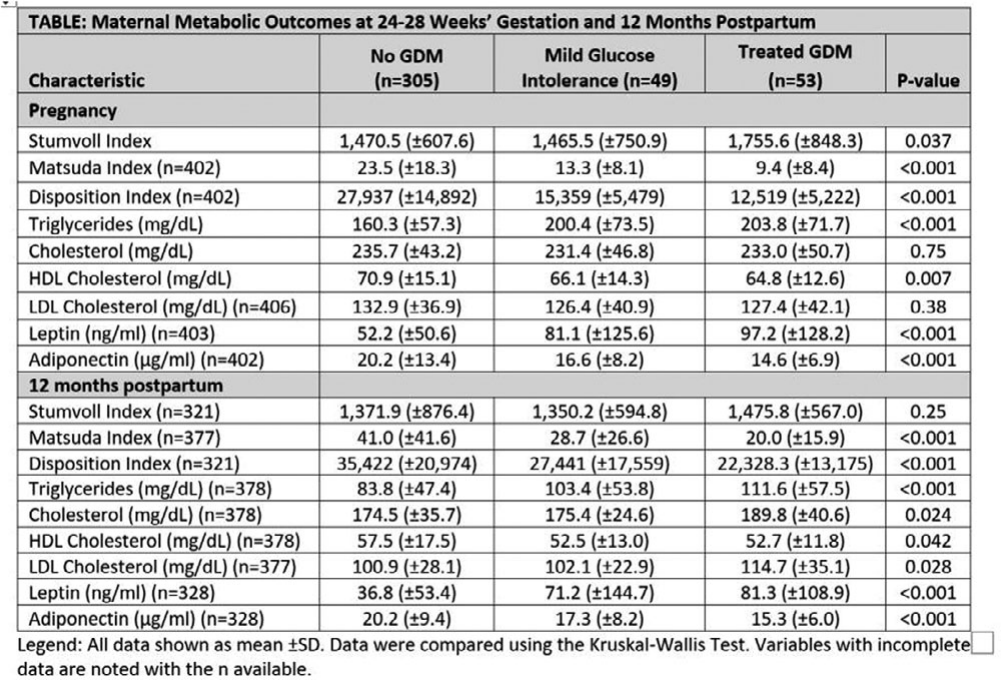

## 725 | Symptoms of Post Traumatic Stress Disorder Among Placenta Accreta Spectrum Providers

Christina M. Duzyj^1^; Alireza A. Shamshirsaz^2^; Daniela A. Carusi^3^

*^1^Massachusetts General Hospital, Boston, MA; ^2^Fetal Surgeon Chief, Division of Fetal Medicine and Surgery Director of the Maternal Fetal Care Center Director of the Perinatal Surgery Fellowship Professor of Surgery, Boston Children's Hospital Harvard Medical School, Boston, MA; ^3^Brigham and Women's Hospital, Boston, MA*

10:30 AM ‐ 12:30 PM

**Objective**: Physicians as a second victim have been increasingly recognized since the COVID‐19 pandemic.  However, physician post‐traumatic stress disorder (PTSD) is less frequently recognized.  We sought to evaluate for symptoms of PTSD among physicians caring for women with placenta accreta spectrum (PAS), a medical condition with known high acuity and high mortality.

**Study Design**: This is a cross‐sectional study consisting of a quantitative survey distributed via email to physicians who either are members of the Pan‐American Society for Placenta Accreta Spectrum, or who attended a PAS Post‐Graduate Course or Scientific Session at the SMFM Annual Meeting in 2024. The survey included the PTSD Checklist for DSM‐5 (PCL‐5), as well as demographic questions, and open ended questions regarding institutional support after severe cases of PAS. Scoring was performed as recommended by the National Center for PTSD.

**Results**: The email containing the survey was opened by 71 individuals, and the survey completed by 21 (30%).  All responders cared for women with PAS, the majority for 3‐5 years (38.1%) although 28.6% reported greater than 10 years experience in caring for PAS, and 38.1% reported caring for a women who died of PAS.  Most (71.4%) reported perceiving personal psychologic impact occasionally from severe PAS cases, with only one respondent never impacted.  66.6% of the cohort met diagnostic criteria for PTSD (PCL‐5 >30), with highest responses being for unwanted memories (42.9%), strong negative feelings, feeling superalert or onguard, and difficulty concentrating (38.1% each).  28.6% reported that their institution provided no psychological support after difficult cases.

**Conclusion**: This preliminary study demonstrates that physicians caring for the obstetric patients at highest risk for mortality are themselves at high risk for post‐traumatic stress disorder.  Although our results are likely biased towards respondents experiencing the outcome of interest, we demonstrate that institutions must build capacity for supporting their physicians when adverse obstetric outcomes occur, particularly among patients with PAS.

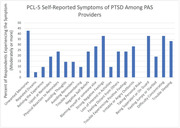

## 726 | Placenta Accreta Spectrum is Associated with Lower Rates of Hypertensive Disorders of Pregnancy

Colleen Sinnott^1^; Olga Grechukhina^2^; Jennifer F. Culhane^2^; Sonya Abdel‐Razeq^2^; Khadija Alshowaikh^2^; Viktoriia Kolesnyk^2^; Yang Yang‐Hartwich^2^; Mert Ozan Bahtiyar^1^; Kevin Dysart^3^

*^1^Yale University, New Haven, CT; ^2^Yale School of Medicine, New Haven, CT; ^3^Nemours Children's Hospital, Wilmington, DE*

10:30 AM ‐ 12:30 PM

**Objective**: Placenta accreta spectrum (PAS) arises from over‐invasion of the placenta, while hypertensive disorders of pregnancy (HDP) are thought to arise from trophoblast under‐invasion; these may represent two ends of a spectrum of disordered placentation. In a single‐institution study, we previously demonstrated clinical evidence suggesting PAS is protective against HDP. Here, we validated this finding in a larger patient cohort.

**Study Design**: This retrospective study utilizes Epic Systems’ Cosmos research platform, an electronic health record database with de‐identified patient‐level data. PAS, HDP (gestational hypertension, preeclampsia, eclampsia, and HELLP) and other clinical and sociodemographic characteristics were extracted from the database for all deliveries from 2017‐2023. Patients with and without HDP were compared across a range of maternal attributes. To account for scheduled preterm deliveries for PAS, such that some patients with PAS deliver before HDP is clinically diagnosed, these comparisons were repeated with cohorts stratified by gestational age (GA) at delivery (< 34 weeks and ≥ 34 weeks). For all cohorts, the crude and adjusted odds of HDP by PAS diagnosis was calculated using logistic regression. All covariates in the bivariate tests of association were included in the multivariable regression models.

**Results**: A total of 4,287,549 delivery encounters were included. Of those, 8,979 (0.21%) had PAS, 869,759 (20.3%) had HDP, and 125,332 deliveries (2.9%) occurred < 34 weeks. In the total cohort, after adjusting for GA at delivery and maternal factors, patients with PAS were half as likely to be diagnosed with HDP (aOR 0.55 [0.51, 0.59]). In both GA‐stratified cohorts, < 34 weeks and ≥34 weeks, this effect remained stable with aOR 0.54 [0.47, 0.62] and aOR 0.42 [0.38, 0.45], respectively.

**Conclusion**: Patients with PAS develop HDP at half the rates seen in those without PAS after controlling for GA at delivery and patient factors. These two seemingly unrelated conditions may result from abnormalities of the same biologic mechanisms and may benefit from future tandem investigation.

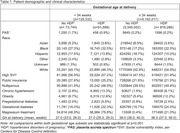

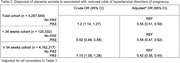

## 727 | Correlation Between Blood Pressures and Markers of Inflammation in the Maternal Circulation in Pregnancy

Corrine K. Nelson^1^; Kylie Cooper^1^; Sylvie Girard^1^; Anna Borchers^1^; Elsa Bernier^2^; Marie‐Eve Brien^3^

*^1^Mayo Clinic, Rochester, MN; ^2^Montreal University, Montreal, PQ; ^3^Sainte‐Justine Hospital Research Center, Montreal, PQ*

10:30 AM ‐ 12:30 PM

**Objective**: Hypertensive diagnostic criteria from the American Heart Association has been revised in non‐pregnant individuals. However, in pregnancy the diagnosis of chronic hypertension, defined as blood pressure >/ = 140/90 prior to pregnancy or 20 weeks gestation, has remained consistent. It is unclear if changes in blood pressure, such as in elevated and stage 1 hypertension, are associated with inflammatory markers. Therefore, we determine if levels of markers of inflammation were associated with blood pressure in a cohort of uncomplicated pregnancies and as compared to patients with gestational hypertension (gHTN).

**Study Design**: Maternal blood samples were obtained in the third trimester close to delivery in a cohort of pregnant patients with uncomplicated pregnancies (n = 96) or with gHTN (n = 36), and plasma isolated for multiplex analysis of inflammatory mediators. In‐depth review of the medical record was performed.  

**Results**: Circulating levels of several mediators were significantly associated with increased blood pressure. Of interest, the pro‐inflammatory chemokine MCP‐1 was positively correlated with blood pressure (r = 0.47, p< 0.001) whilst CXCL10 was negatively correlated (r = ‐0.36, p< 0.01). In addition, pro‐inflammatory cytokines IL‐5 and TNFa presented trending positive correlation with blood pressure (IL‐5: r = 0.22, p = 0.072; TNFa: r = 0.024, p = 0.055). Surprisingly, the anti‐inflammatory cytokine IL‐4 was also positively correlated with blood pressure (r = 0.24, p< 0.05). Of note, MCP‐1 and CXCL10 were both significantly elevated in gHTN while IL‐5 and TNFa were unchanged.

**Conclusion**: Our data suggest that specific inflammatory markers, including MCP‐1–responsible for monocyte recruitment, IL‐5 and TNFa are correlated with increased blood pressure in a cohort of uncomplicated pregnancies. While MCP‐1 was also significantly elevated in gHTN, this was not the case for the other cytokines. Further analysis will ascertain the role of pre‐pregnancy body mass index in the level of these markers. In addition, a larger cohort is needed to have better understanding of the clinical significance of this work.

## 728 | Should Transvaginal Cervical Length Surveillance be Conducted After Cerclage Placement to Predict Spontaneous Preterm Birth?

Dahiana M. Gallo^1^; Rupsa C. Boelig^2^; Julio Mateus Nino^3^; Vincenzo Berghella^4^; Leonardo Pereira^5^; Catalina M. Valencia^6^; Joanne N. Quiñones^7^; Richard M. Burwick^8^; Joseph Bell^9^; Luisa López‐Torres^10^; Adam Taylor^11^; Luis Alvarado^12^; Jorge E. Tolosa^13^

*^1^St. Lukes University Health Network, Bethlehem, PA; ^2^Sidney Kimmel Medical College, Thomas Jefferson University, Philadelphia, PA; ^3^Atrium Health, Wake Forest University School of Medicine, Charlotte, NC; ^4^Sidney Kimmel Medical College of Thomas Jefferson University, Philadelphia, PA; ^5^Oregon Health & Science University, Portland, OR; ^6^Clinica del Prado, Unidad de Medicina Materno Fetal, Medellín, Colombia, Medellin, Antioquia; ^7^Lehigh Valley Health Network, Allentown, PA; ^8^San Gabriel Valley Perinatal Medical Group; Pomona Valley Hospital Medical Center, Pomona, CA; ^9^St. Luke's University Health Network, Department of Obstetrics & Gynecology, Maternal Fetal Medicine, Bethlehem, PA, 18015, Bethlehem, PA; ^10^Universidad Pontificia Bolivariana, Medicina Materno Fetal, Medellín, Colombia, Medellin, Antioquia; ^11^St. Luke's University Health Network, Department of Obstetrics & Gynecology, Bethlehem, PA; ^12^St. Luke's University Health‐network, Bethlehem, PA; ^13^St. Luke's University Health Network, Bethlehem, PA*

10:30 AM ‐ 12:30 PM

**Objective**: Transvaginal cervical length (CL) surveillance after cerclage placement is not universally performed and recommended. The objective of our study was to assess whether a cervical length < 25 mm after cerclage placement affects the risk of spontaneous preterm birth (sPTB).

**Study Design**: Retrospective cohort of the International Collaborative for Cerclage Longitudinal Evaluation and Research (IC‐CLEAR) study included singleton pregnancies managed with cerclage for history, ultrasound, or physical exam indications between June 2016 and August 2020 at 8 sites across the United States (6) and Colombia (2). Exclusion criteria were patients without CL measurement post cerclage and iatrogenic preterm birth < 37 weeks. The exposure of interest was cervical length after cerclage < 25 mm. Statistical analysis included univariable and multivariable models.  Each model was adjusted for the use of vaginal progesterone after cerclage.

**Results**: Of 839 patients managed with cerclage, 408 (49.39%) had a CL measurement after cerclage, but only 219 patients met the criteria. Patients with a CL < 25mm post‐cerclage did not have a significantly increased risk of sPTB < 37 weeks or < 34 weeks (76% vs 73%, p 0.680, 52 vs 42%, p 0.140 respectively) compared to those whose cervix returned to a normal length of >25 mm; however, a CL < 20mm was associated with an increased risk of spontaneous preterm birth < 34 weeks (59% vs 43% p 0.039). After adjusting for the use of vaginal progesterone, there was a reduction in the risk of sPTB before 34 weeks (RR 1.49,95% CI 0.88 ‐2.54, p 0.131).

**Conclusion**: The results indicate that if the cervical length after cerclage is less than 20 mm, there remains an elevated risk of preterm birth before 34 weeks. Monitoring the cervical length following cerclage placement could be beneficial. In these instances, additional treatments such as progesterone might be considered when counseling patients to help lower the rate of early spontaneous preterm birth.

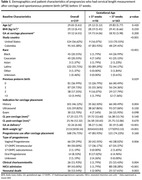

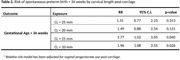

## 729 | An Updated Rapid Review of Pain Management Following Cesarean Birth

Dana Feldman^1^; Bryan L. Aaron^2^; Faith Gacheru^2^; Holli Sharples^2^; Michelle Moniz^2^; Jennifer Waljee^2^; Mark Bicket^2^; Lisa Kane Low^2^; LaTeesa James^2^; Alex Peahl^3^

*^1^Wake Forest University, Winston‐Salem, NC; ^2^University of Michigan, Ann Arbor, MI; ^3^Michigan Medicine, Ann Arbor, MI*

10:30 AM ‐ 12:30 PM

**Objective**: To update our rapid review assessing the evidence for key aspects of pain management following cesarean birth, to inform new clinical practice guidelines.

**Study Design**: We used Ovid MEDLINE, Elsevier's Scopus, Elsevier's Embase, Google Scholar, PubMed, and Web of Science to perform a narrative synthesis of studies that assessed opioid use, analgesia effects, patient‐centered outcomes, and disparities in the general population, patients with opioid use disorder (OUD), chronic pain, and psychiatric conditions. Articles were screened and abstracted by two reviewers. Quality was assessed using the RAND/UCLA Appropriateness Methodology approach.

**Results**: Of 2753 studies screened, 106 were included: 24 RCTs, 5 non‐RCTs, and 77 observational studies (7 with very high/critical, 28 with high/serious, 25 with moderate/some concerns for, and 17 with low risk of bias). Following cesarean birth, multimodal, non‐opioid interventions (e.g. scheduled ibuprofen and acetaminophen) consistently reduced opioid consumption and/or prescribing without increasing pain. discharge prescribing studies showed variability and racial/ethnic disparities following cesarean births. Patients with chronic pain and psychiatric conditions had increased opioid exposure and persistent use. Non‐pharmacologic strategies had limited evidence for pain reduction but high satisfaction. Tailored prescribing reduced outpatient opioid use without worsening pain control. Higher inpatient use, discharge prescription size, and substance abuse history were associated with new persistent opioid use. Patients with chronic pain and psychiatric conditions had increased opioid exposure and persistent use.

**Conclusion**: Multimodal opioid‐sparing strategies provide adequate pain control while limiting opioid use after cesarean birth. Non‐pharmacologic methods may provide low‐risk enhancement in pain management. There are significant evidence gaps for postpartum pain management in patients with OUD, chronic pain, and psychiatric conditions.

## 730 | Non‐Hormonal Treatments for Vasomotor Sequelae in Patients with Postpartum Hemorrhage Requiring Peripartum Hysterectomy

Daniel J. Martingano^1^; Amanda F. Francis Oladipo^2^; Marwah Al‐Dulaimi^3^; Sandra Kumwong^4^; Andrea Ouyang^5^; Lauren Cue^6^; Ashley Nguyen^3^; Francis X. Martingano^7^; Shailini Singh^8^; Mark Rebolos^3^; Kristin Cohen^9^; Alexander Ulfers^10^; Donald Morrish^3^; Iffath A. Hoskins^11^

*^1^St. John's Episcopal Hospital‐South Shore and William Carey University College of Osteopathic Medicine, Far Rockaway, NY; ^2^Hackensack University Medical Center, Hackensack, NJ; ^3^St. John's Episcopal Hospital‐South Shore, Far Rockaway, NY; ^4^Touro College of Osteopathic Medicine‐Harlem Campus, New York, NY; ^5^William Carey University, Hattiesburg, MS; ^6^Rutgers University and the Jersey City Medical Center, Jersey City, NJ; ^7^NYU Grossman School of Medicine ‐ NYU Brooklyn, New York, NY; ^8^AtlantiCare Regional Medical Center, Pamona, NJ; ^9^RWJBarnabas Health ‐ Trinitas Regional Medical Center, Elizabeth, NJ; ^10^Walter Reed National Military Medical Center, Bethesda, MD; ^11^Albert Einstein College of Medicine ‐ Montefiore Medical Center, New York, NY*

10:30 AM ‐ 12:30 PM

**Objective**: Emergent peripartum hysterectomy may be required as a lifesaving procedure for sever postpartum hemorrhage (SPPH). SPHH requiring hysterectomy can rarely be associated with severe complications such as unintended oophorectomy or Sheehan syndrome, which respectively can lead to vasomotor sequelae. This study sought to evaluate the effectiveness of nonhormonal treatment regimens for vasomotor sequelae in patients with pregnancies complicated by SPHH requiring peripartum hysterectomy.

**Study Design**: We conducted a multi‐center, prospective observational study from 7/2019 to 7/2024 comparing all pregnant patients requiring a peripartum hysterectomy due to SPHH and endorsed vasomotor symptoms up to 6‐months postpartum. Monotherapy clonidine 24‐hour patch, paroxetine, gabapentin, black cohosh, sertraline, and fluoxetine were included as covariates. Patients less than 34 0/7 weeks gestation, with prior endocrine or rheumatology disorders, or allergies to or prior use of any of the covariates were excluded. The primary outcomes included patient‐reported resolution of daytime and/or nighttime symptoms confirmed by provider assessment and the need to switch medication, as discrete events.

**Results**: The study included 27 patients who reported vasomotor symptoms following SPHH requiring hysterectomy. Study groups' demographics were not significantly different. 16 (59.3%) patients had complications of unintended oophorectomy, 20 (74.1%) were diagnosed with Sheehan Syndrome, and 9 (33.3%) were diagnosed with both conditions. Patients using the clonidine 24‐hour patch were more likely to achieve daytime vasomotor symptom resolution (92.6% v. 18.5%, p = 0.030) and were less likely to switch medications (85.2% v. 22.2%, p = 0.004). Patients using paroxetine at doses of 10mg or greater were more likely to achieve resolution of both nighttime vasomotor and insomnia symptoms compared to non‐use (88.9% v. 11.1%, p = 0.021).

**Conclusion**: Clonidine and paroxetine are reasonable nonhormonal treatment therapies for vasomotor sequelae in patients with pregnancies complicated by SPHH requiring peripartum hysterectomy.

## 731 | Effect of Oral Magnesium Supplementation as Adjunctive Treatment in Pregnancies Complicated by Chronic Hypertension

Daniel J. Martingano^1^; Amanda F. Francis Oladipo^2^; Marwah Al‐Dulaimi^3^; Sandra Kumwong^4^; Andrea Ouyang^5^; Lauren Cue^6^; Ashley Nguyen^3^; Francis X. Martingano^7^; Shailini Singh^8^; Alexander Ulfers^9^; Mark Rebolos^3^; Kristin Cohen^10^; Donald Morrish^3^; Iffath A. Hoskins^11^

*^1^St. John's Episcopal Hospital‐South Shore and William Carey University College of Osteopathic Medicine, Far Rockaway, NY; ^2^Hackensack University Medical Center, Hackensack, NJ; ^3^St. John's Episcopal Hospital‐South Shore, Far Rockaway, NY; ^4^Touro College of Osteopathic Medicine‐Harlem Campus, New York, NY; ^5^William Carey University, Hattiesburg, MS; ^6^Rutgers University and the Jersey City Medical Center, Jersey City, NJ; ^7^NYU Grossman School of Medicine ‐ NYU Brooklyn, New York, NY; ^8^AtlantiCare Regional Medical Center, Pamona, NJ; ^9^Walter Reed National Military Medical Center, Bethesda, MD; ^10^RWJBarnabas Health ‐ Trinitas Regional Medical Center, Elizabeth, NJ; ^11^Albert Einstein College of Medicine ‐ Montefiore Medical Center, New York, NY*

10:30 AM ‐ 12:30 PM

**Objective**: Intravenous magnesium sulfate remains an essential medication for seizure prophylaxis in the context of severe preeclampsia (SPEC), with oral formulations reserved for non‐severe comorbid conditions with inconsistent results. This study sought to determine the effect of oral magnesium supplementation as adjunctive treatment in pregnancies complicated by chronic hypertension (CHTN).

**Study Design**: We conducted a multi‐center, prospective observational study from 7/2022 to 7/2024 and included all pregnant women diagnosed with chronic hypertension requiring antihypertensive medication with gestational ages ranging from 24 0/7 through 38 0/7 weeks‐gestation. All patients were prescribed low‐dose aspirin prophylaxis. Monotherapy magnesium oxide and magnesium citrate were included as covariates. The primary outcomes included diagnosis of superimposed SPEC, new‐onset headache (NOH) not relieved by acetaminophen monotherapy, and worsening hypertension requiring additional or initiation of antihypertensive medications, as discrete events. Patients with preexisting neurological or cardiac disorders or allergies to any included medications were excluded. Medication choice was determined by physician clinical assessment.

**Results**: The study included 693 patients diagnosed with CHTN. 351 patients were given magnesium oxide and 342 patients were given magnesium citrate. Baseline demographic factors were not significantly different. Patients who received magnesium oxide were less likely to develop SPEC (30.1% v. 69.9% p = 0.001) or NOH (14.1% v. 73.1%, p< 0.001) with a 14% (RR = 0.86, 95% CI 0.68‐0.93, p = 0.004) and 33% (RR = 0.67, 95% CI 0.41‐0.82, p = 0.002) decreased risk in adjusted models, respectively. In stratified analysis, patients with BMI < 30 kg/m2 who received magnesium oxide were less likely to require addition or initiation of antihypertensive medications (54.8% v. 22.9%, *p*  = < 0.001). None of the primary outcomes were significant for patients receiving magnesium citrate.

**Conclusion**: Magnesium oxide supplementation may be beneficial for patients with pregnancies complicated by CHTN.

## 732 | Twin Pregnancy is a Significant Risk Factor for Delivery within 24 hours in P‐PROM

Daniel Gabbai^1^; Itamar Gilboa^1^; Anat Lavie^2^; Yariv Yogev^3^; Emmanuel Attali^1^

*^1^Lis Hospital for Women's Health, Tel Aviv Sourasky Medical Center, Tel‐Aviv, Tel Aviv; ^2^Tel Aviv Sourasky Medical Center, Tel Aviv Sourasky Medical Center, Tel Aviv; ^3^Lis Maternity Hospital, Sourasky Medical Center, Tel Aviv University, Tel Aviv Sourasky Medical Center, Tel Aviv*

10:30 AM ‐ 12:30 PM

**Objective**: To assess the likelihood of delivery within 24 hours after preterm premature rupture of membranes (P‐PROM) in twin pregnancies in comparison to singleton pregnancies.

**Study Design**: We conducted a retrospective cohort study in a tertiary university‐affiliated medical center with approximately 12,500 deliveries annually (2012‐2023). Data collected encompassed demographic and obstetrics characteristics including maternal age, gestational age at delivery, body mass index, parity, mode of delivery, and delivery outcomes. A comparison was made between twin (study group) and singleton (control group) pregnancies with P‐PROM (gestational age 24+0‐36+6). Statistical analyses, including Cox regression and multivariate logistic regression, were performed to evaluate the impact of twin pregnancies on the likelihood of delivery within 24 hours.

**Results**: 1) During the study period, 145,883 women delivered in our center. Of them, 1,498 [1%] women were admitted with P‐PROM, of whom 297 (19.9%) with twin pregnancies.

2) The characteristics of the two groups differ regarding gestational age at delivery, parity, previous cesarean deliveries, and mode of conception [Table 1].

3) In the study group, 234 women (78.8%) delivered within 24 hours, while 697 women (58.3%) in singleton pregnancies (p‐value< 0.001).

4) Using a Cox regression and multivariate logistic regression, twin pregnancy was identified as a significant factor for delivery within 24 hours: HR 2.3 (95%CI 1.9‐2.7, p< 0.001) [Table 2].

**Conclusion**: Twin pregnancy was found to be the most significant risk factor for delivery within 24 hours in women with P‐PROM.

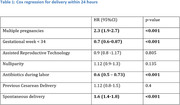

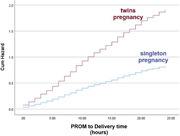

## 733 | The 5‐Year Results of an Institutional Shadowing Program Aiming to Train New Senior “Obstetricians On‐Call”

Daniel Tairy^1^; Eran Weiner^2^; Anat Engel^3^; Yael Ganor Paz^1^

*^1^Edith Wolfson Medical Center, Holon, HaMerkaz; ^2^Wolfson Medical Center, Wolfson Medical Center, Tel Aviv; ^3^Edith Wolfson Medical Center, holon, HaMerkaz*

10:30 AM ‐ 12:30 PM

**Objective**: On‐call night and weekend shifts are pivotal components of the clinical activity of any obstetric unit. Worldwide, these teams are usually led by a senior in‐house “obstetrician on call” (OOC). This study presents the effects of a structured institutional shadowing program to train future OOCs (fOOC) on their clinical competencies and on obstetric adverse outcomes.

**Study Design**: In August 2022 we implemented a training program where senior residents (fOOCs) improve complex obstetric and leadership competencies by performing repeated 24h calls shadowed by an experienced obstetrician. After each 24h call the fOOC received a structured oral and written feedback scoring their competencies. Additionally, the fOOC's self‐assessed their respective competencies upon entering and completing the program using structured questionnaires. Lastly, we compared the occurrence of major maternal and neonatal adverse outcomes before (January 2019 to July 2022 = P1) vs. after (August 2022 to July 2024 = P2) the program implementation.

**Results**: As of August 2024 we performed 151 shadowed 24h shifts, performed by 8 fOOCs trained by 15 obstetricians. The fOOC's skills were evaluated with a higher performance degree when compared between upon entering vs. completing the program as assessed by the fOOCs as well as by the senior obstetricians (p< 0.05 to all). Figure 1 describes an example for the improvement of the clinical competencies as were assessed by the senior obstetricians for the cohort of fOOCs during the program. Additionally, when comparing the 13336 on‐call deliveries in P1 to the 8718 on‐call deliveries in P2, P2 was characterized by lower rates of maternal bleeding and transfusion (p< 0.001), shoulder dystocia (p = 0.04), neonatal Apgar score< 7 (p = 0.009), respiratory morbidity (p = 0.02), Erb's palsy (p = 0.03), and neonatal death (p = 0.05)‐ table 1.

**Conclusion**: The implementation of a shadowing training program to fOOC was associated with a higher reported perception of clinical competencies, and the 2‐year period post implementation was associated with institutional lower rates of important obstetric adverse outcomes.

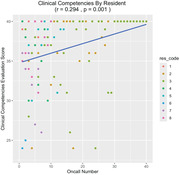

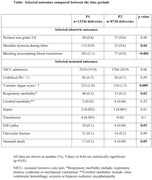

## 734 | Preterm Medically Indicated Labor Induction and Mode of Delivery in Patients with Obesity

Daniella Rogerson^1^; Madison Kent^2^; Minhazur R Sarker^1^; Alice Sutton^1^; Elizabeth Nicole Teal^3^; Cynthia Gyamfi‐Bannerman^3^

*^1^University of California San Diego Health, San Diego, CA; ^2^University of California San Diego, San Diego, CA; ^3^University of California, San Diego, San Diego, CA*

10:30 AM ‐ 12:30 PM

**Objective**: Maternal obesity is associated with cesarean delivery (CD) during induction of labor (IOL) at term; whether the same is true for patients with obesity undergoing preterm IOL is unknown. We sought to evaluate CD rates for patients with and without obese BMI undergoing medically indicated preterm IOL.

**Study Design**: This is a secondary analysis of a multicenter, randomized trial of singleton pregnancies at risk for preterm delivery. Participants with body mass index (BMI) >18.5 who underwent IOL for any indication were included.  Planned CD and spontaneous labor were excluded. We compared delivery mode among participants undergoing preterm IOL by body mass index (BMI) at delivery: non‐obese 18.5‐29.9, class 1 30‐34.9, class 2 35‐39.9, class 3 > 40. The primary outcome was incidence of CD by BMI class. Chi‐square, ANOVA and multivariable regression tests determined the strength of association.

**Results**: Of 1236 included participants, 482 had non‐obese BMI (39%); 288 (23%), 220 (18%) and 246 (20%) had class 1‐3 obesity. Pregnancies with class 1‐3 obesity were more likely to be of Black race (20% for non‐obese; 27%, 29% and 37% for class 1‐3) or have hypertension (HTN) (6% non‐obese; 13%, 21%, 33% class 1‐3) or gestational diabetes (GDM) (6% non‐obese; 13%, 18%, 21% class 1‐3) (p values < .0001). In a univariate analysis, those with obesity were more likely to undergo unplanned CD than their non‐obese counterparts (p< .0001). In a multivariable analysis adjusted for nulliparity, advanced maternal age, HTN, GDM, and treatment group, participants with class 1‐3 obesity were more likely to undergo CD than their non‐obese counterparts (class 1 aOR 1.62, 95% CI 1.12‐2.36, class 2 aOR 2.30, 95% CI 1.55‐3.41, class 3 aOR 2.96, 95% CI 2.02‐4.33).

**Conclusion**: These novel findings suggest that while participants with obesity are more likely to undergo CD during medically indicated pre‐term IOL, the majority still deliver vaginally. This data may aid in counseling patients undergoing medically indicated pre‐term delivery who have obese BMI.

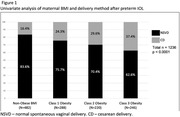

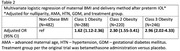

## 735 | Nicu Admission for Neonatal Hypoglycemia and Impact on Breastfeeding Outcomes Among Gravidas with Diabetes

Daniella Rogerson^1^; Marni B. Jacobs^2^; Minhazur R. Sarker^3^; Kim Boggess^4^; Ashley N. Battarbee^5^; Gladys (Sandy) A. Ramos^3^; On behalf of the MOMPOD Study Consortium

*^1^University of California San Diego Health, San Diego, CA; ^2^University of California, San Diego Health, San Diego, CA; ^3^University of California, San Diego, San Diego, CA; ^4^University of North Carolina at Chapel HIll, Chapel Hill, NC; ^5^Center for Women's Reproductive Health, University of Alabama at Birmingham, Birmingham, AL*

10:30 AM ‐ 12:30 PM

**Objective**: Neonatal hypoglycemia (NH) is common in pregnancies with diabetes mellitus (DM) and can lead to neonatal intensive care unit (NICU) admission, a known barrier to breastfeeding (BF). We hypothesized that BF rates are lower after NICU admission among infants with NH compared to NICU admission without NH. This study compares BF rates among gravidas with DM with neonatal NICU admission.

**Study Design**: This is a secondary analysis of the MOMPOD randomized controlled trial of metformin versus placebo in insulin treated DM. Participants with live births and intention to BF were included. NH was defined as glucose < 40mg/dL or requiring IV glucose within 72 hours. A BF questionnaire was collected 30 days postpartum.  Primary outcome was exclusive and partial BF among participants with neonatal NICU admission with NH versus other indications. Secondary outcomes were time and reasons for BF cessation. Characteristics were compared with Chi‐square and t‐tests or Wilcoxon; multivariable regression tests were performed.

**Results**: Of 468 participants who completed a BF survey, 378 were included. 142 neonates were admitted to the NICU: 102 with NH and 40 without. Neonates admitted to the NICU with NH were more often born by cesarean and had higher birthweight than those without (p < 0.05, Table 1). No BF, exclusive BF, and partial BF did not differ between groups; this remained true when adjusting for birthweight and mode of delivery (aOR 0.62, 95% CI 0.25–1.51, aOR 2.72, 95% CI 0.54 –13.60, aOR 1.10, 95% CI 0.44–2.70, Table 2). Participants who stopped BF did so at an average of 2.8 weeks for both groups (p = 0.91) and for similar reasons (trouble latching, pain, poor weight gain, low supply, p > 0.05, data not shown).

**Conclusion**: Among pregnancies complicated by DM, NICU admission with NH did not pose additional barriers to BF compared with NICU admission in general, although analysis is limited by sample size.  Future studies examining specific reasons for BF cessation pertinent to NH are needed to understand which factors affect BF in this population.

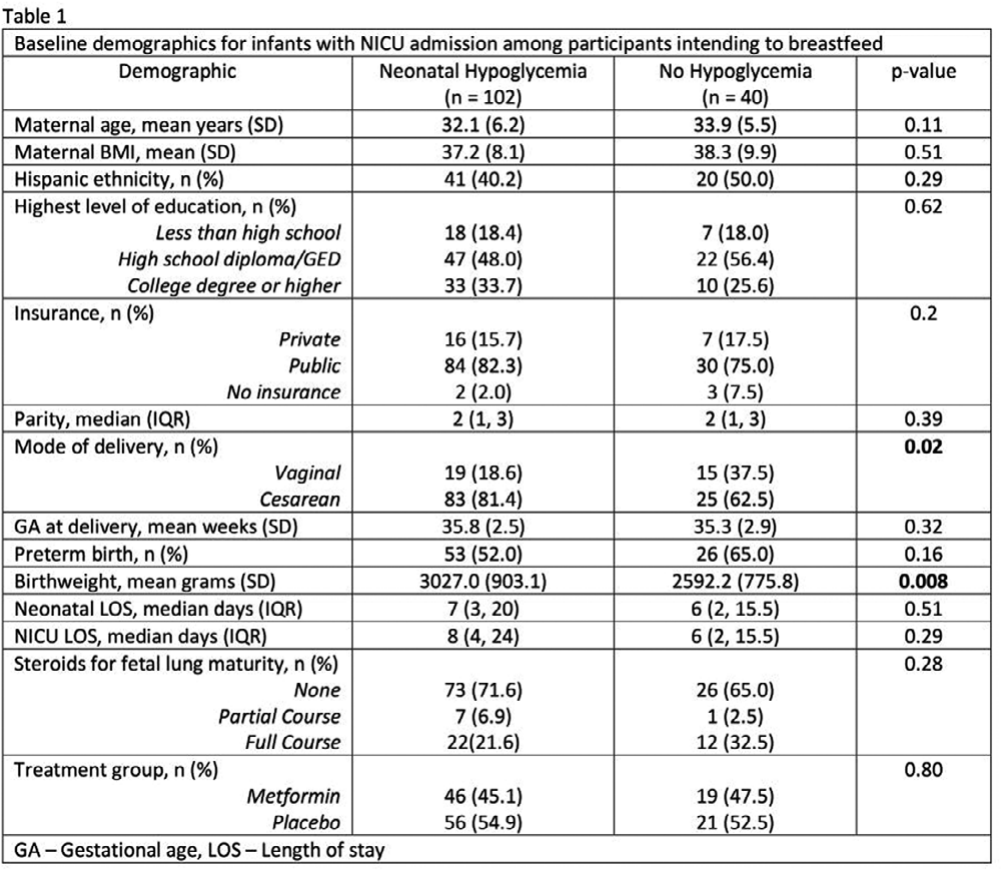

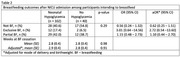

## 736 | The Rate of Weight Gain During Pregnancy and Newborn Head Circumference

Danruo Zhong; Grace Liu; Khadija R. Jones; Elizabeth Werner; Catherine E. Monk

*Columbia University Irving Medical Center, New York, NY*

10:30 AM ‐ 12:30 PM

**Objective**: Weight during pregnancy, a marker of gestational biology and maternal health, has been related to neonatal outcomes. However, no study has examined the effects of the *rate of weight gain* during pregnancy on newborn head circumference, an early indicator of children's neurodevelopment (e.g., autism spectrum disorder). In this study, we aim to examine the association between the initial level and the rate of body mass index (BMI) change during pregnancy and newborn head circumference.

**Study Design**: This is a secondary data analysis from the Prenatal Stress: The Epigenetic Basis of Maternal and Perinatal Effects Study. From n = 187 pregnant participants (M_Age_ = 29.64; SD_Age_ = 6.24), BMI in pregnancy was collected three times at gestational week 15, 25, and 35, respectively. Newborn head circumference, gestational age at birth, and sex were abstracted from the medical record. We hypothesized that BMI intercept (from 15 weeks) and slope would be positively associated with newborn head circumference.   

A latent growth curve model (LGCM) was used to delineate the trajectories of mothers’ BMI across three assessments, treating the intercept (BMI at 15 weeks) and the slope (rate of BMI change) as two latent variables. Additionally, a structural equation model (SEM) was built, predicting newborn head circumference from the intercept and the slope of BMI while controlling for mother's age, newborn gestational age at birth and sex.

**Results**: Sample characteristics are shown in Table 1. Pregnant individuals’ BMI intercept (B = 1.27, *p* < .05) and slope across three assessments (B = 1.19, *p* < .05) were positively associated with newborn head circumference (Figure 1).

**Conclusion**: Individuals’ BMI at the start of the 2^nd^ trimester and its rate of change throughout the 2^nd^ and 3^rd^ trimesters were positively associated with newborn head circumference. These findings suggested that the *rate* of gestational weight gain, in addition to weight status, may play an important role in influencing infant growth and future health.

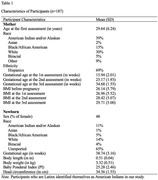

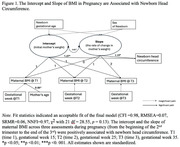

## 737 | Risk Factors for Preeclampsia Following Early Onset Fetal Growth Restriction

David Nadav Sabag^1^; Ronen Fluss^2^; Rakefet Yoeli‐Ullman^3^; Abraham Tsur^4^

*^1^Sheba Medical Center, Ramat Gan, Tel Hashomer, Israel, Givatayim, HaMerkaz; ^2^Gertner Institute, Ramat Gan, HaMerkaz; ^3^Sheba Medical Center, Ramat Gan, HaMerkaz; ^4^Sheba Medical Center, Ramat Gan, Tel Hashomer, Israel, Ramat Gan, HaMerkaz*

10:30 AM ‐ 12:30 PM

**Objective**: Early onset fetal growth restriction (FGR) may precede the development of preeclampsia (PE). The aim of this study was to evaluate risk factors associated with the development of PE in individuals with early onset FGR.

**Study Design**: The study included all consecutive individuals admitted to the antepartum unit of a tertiary university affiliated medical center between 2011‐2024 for the diagnosis of early onset FGR. Maternal and pregnancy characteristics were recorded, including: maternal age, BMI, smoking, parity, gestational age (GA) at diagnosis, chronic hypertension, maternal cardiac disease, pre‐ or gestational diabetes, thrombophilia, chronic renal disease, history of major pregnancy complications, mode of conception, number of fetuses, premature contractions, ultrasound (US) measured abdominal circumference (AC), US measured estimated fetal weight (EFW), umbilical artery doppler PI (UAPI) and middle cerebral artery doppler PI (MCAPI).

AC and EFW were classified as < 5% and < %3 respectively based on local population growth charts. UAPI and MCAPI percentiles calculated per specific gestational age.

We investigated the association of these variables with development of PE using univariate and multivariable logistic regression.

**Results**: During the study period 774 pregnant individuals were admitted for early‐onset FGR, while 79 of them (10.2%) subsequently developed PE. Table 1 presents maternal and pregnancy characteristics according to the development of PE. Using multivariate analysis (Table 2) GA 24‐28 weeks at diagnosis (OR 2.54, CI 1.20‐5.36, p = 0.01), AC below the 5^th^ percentile (OR 2.31, CI 1.10‐4.86, P = 0.02), and UAPI above the 95^th^ percentile (OR 3.35, CI 1.55‐7.20, P< 0.01) were significantly associated with the subsequent development of PE.

**Conclusion**: Early GA at diagnosis of FGR, AC below the 5^th^ percentile and UAPI >95^th^ percentile are significantly associated with the development of PE in pregnancies complicated by early‐onset FGR.

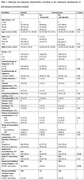

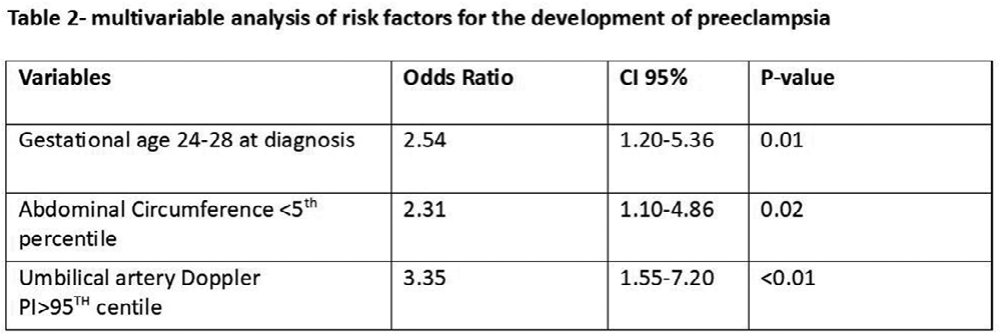

## 738 | Severe Maternal Morbidity In Urban vs Rural Populations Following Patient Safety Bundle Implementation in Hawaii

Deepraj K. Pawar^1^; Mary Tschann^2^; Julie Kathman^3^; Rebecca Delafield^4^; Pai‐Jong Stacy Tsai^2^

*^1^University of Hawaii, Honolulu, HI; ^2^University of Hawaii, John Burns School of Medicine, Honolulu, HI; ^3^Health Association of Hawaii, Honolulu, HI; ^4^University of Hawaii, Manoa, Honolulu, HI*

10:30 AM ‐ 12:30 PM

**Objective**: The Alliance for Innovation on Maternal Health (AIM) has developed evidence‐based patient safety bundles (PSB) to decrease severe maternal morbidity (SMM) for several obstetric outcomes including  hemorrhage. In 2021, 10/12 hospitals with birthing facilities in Hawaii adopted AIM safety bundles.

Hawaii faces challenges in implementing such interventions given its unique geography. Hawaii has 5 urban birthing facilities, all located on the island of Oahu, and 7 rural facilities, located on neighbor islands. Resources, such as blood products, are limited on neighbor islands and transfer to higher level tertiary urban facilities requires air transportation. Our goal was to explore how SMM rates in the setting of obstetric (OB) hemorrhage differ between rural vs urban centers in Hawaii, particularly since AIM PSB implementation.

**Study Design**: We abstracted data from the AIM Data Center, which provided aggregated SMM rates among OB hemorrhage cases (excluding ectopic pregnancy and spontaneous abortion) for 4 urban and 6 rural birthing facilities in the state of Hawaii, and stratified by year from 2016 to 2023.

**Results**: A total of 9,527 births were included. Average SMM rates in OB hemorrhage at rural centers ranged from 18.0% to 27.4% over the course of the 2016 to 2023 study period (Figure 1). At the time of implementation of AIM PSB in 2021, SMM rate was 24.2%, fluctuating to 18.0% and 27.4% in 2022 and 2023 respectively. In Hawaii's urban centers, SMM fluctuated between 18.1% to 24.7% during the study period, with a rate of 22.3% in 2021, decreased to 18.4% by 2023.

**Conclusion**: Our analysis showed that Hawaii's rural centers often have a higher rate of SMM than Hawaii's urban centers. Our analysis also revealed that urban populations did have a reduction in SMM rates among OB hemorrhage after the implementation of AIM PSB in 2021; rural populations did not see a similar decrease.

This data supports previous known association between rural residence and increased SMM. Further studies are warranted to identify specific trends and interventions for the unique challenges rural Hawaiian communities face.

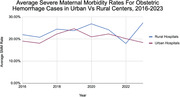

## 739 | Risk Factors Associated with Uterine Rupture in Stillbirth Pregnancies with Prior Cesarean

Dharani Ramaiah^1^; Ashley Boerrigter^2^; Emily A. DeFranco^3^

*^1^University of Kentucky College of Medicine, Lexington, KY; ^2^University of Kentucky, Lexington, KY; ^3^Department of Obstetrics and Gynecology, University of Kentucky, Lexington, KY*

10:30 AM ‐ 12:30 PM

**Objective**: The risk‐benefit ratio of trial of labor after cesarean differs when fetal risk is absent, as in pregnancies complicated by stillbirth. We aimed to quantify risk factors associated with uterine rupture in mothers with stillbirth and prior cesarean to help inform counseling regarding mode of delivery and risk.

**Study Design**: Case‐control study using US vital statistics fetal death data from all stillbirths in the US, 2005‐2021. Study population was limited to patients with prior cesarean and birth at ≥ 20 weeks gestation. The case and controls were deliveries with and without uterine rupture, respectively. Logistic regression estimated the risk of various factors and uterine rupture while adjusting for coexisting risks.

**Results**: There were 422,963 stillbirths during the study period. 15,132(3.6%) were ≥ 20wks and had prior cesarean. 332 (2.2%) cases had uterine rupture and 14,800 (97.8%) controls did not. Hispanic ethnicity was associated with lower odds of rupture, whereas no prenatal care, two or more prior cesareans, preterm delivery < 28 weeks, and TOLAC were associated with higher odds of rupture, even after adjusted analyses. The most significant association was trial of labor after cesarean (TOLAC) being nearly 4.5 times more likely to result in uterine rupture (OR = 4.48 [3.28‐6.13], p< 0.001). The rate of uterine rupture among those undergoing TOLAC with 1, 2 and 3 or greater cesareans was 8.9%, 22.9%, and 19.7%, respectively.

**Conclusion**: Sociodemographic, medical, and obstetric factors influence the risk of uterine rupture among pregnancies complicated by stillbirth and prior cesarean. The highest risk for uterine rupture was observed with no prenatal care, early preterm delivery, higher number of prior cesareans and TOLAC attempt. These data may be useful in counseling regarding mode of delivery and anticipated risks when managing delivery in cases of stillbirth.

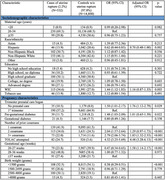

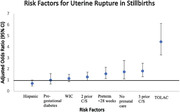

## 740 | The Association Between Maternal Preeclampsia and the Risk for Offspring Endocrine Morbidity– Sibling Matched Analysis

Dor Marciano^1^; Eyal Sheiner^2^; Tamar Wainstock^2^

*^1^Soroka university medical center, Soroka, HaDarom; ^2^Department of Obstetrics and Gynecology, Soroka University Medical Center, Ben‐Gurion University of the Negev, Beer‐Sheva, Israel., Beer Sheva, HaDarom*

10:30 AM ‐ 12:30 PM

**Objective**: Emerging research suggests maternal preeclampsia may be linked to long‐term endocrine morbidities in offspring, but these studies may be biased due to uncontrolled confounding variables. This sibling‐matched study investigates the association between maternal preeclampsia during pregnancy and long‐term endocrine hospitalizations in offspring, rigorously controlling for potential confounders.

**Study Design**: A retrospective population‐based cohort study was conducted with parous women diagnosed with preeclampsia in one pregnancy. A sibling‐matched analysis compared siblings where one was prenatally exposed to maternal preeclampsia, and the other was not. The incidence of hospitalization for endocrine morbidities, including diabetes mellitus, thyroid disorders, and obesity, was compared between siblings, as well as the time to first hospitalization. Multivariable generalized estimation equation (GEE) analysis adjusted for confounding variables.

**Results**: Among 8544 siblings, with half born after a preeclamptic pregnancy, the Kaplan‐Meier survival curve showed no higher cumulative endocrine‐related hospitalization rates in the preeclampsia group (log‐rank P = 0.363). The crude rates of total endocrine hospitalizations were comparable between the preeclampsia and non‐preeclampsia groups (0.6% vs. 0.5%; OR = 0.994; 95% CI 0.992‐0.997 P = 1.0). After adjusting for confounders such as maternal age, gestational age, and induction of labor, the adjusted hazard ratio from the GEE model was not significant (adjusted HR = 1.112; 95% CI 0.598‐2.068 P = 0.737).

**Conclusion**: This sibling‐controlled cohort study found no significant association between maternal preeclampsia and long‐term endocrine‐related hospitalization in offspring after adjusting for potential confounders. These findings suggest that prior associations may have been influenced by unmeasured confounding variables. Further research is needed to validate these results and continue exploring the long‐term endocrine implications of maternal preeclampsia.

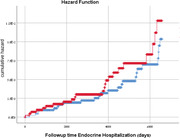

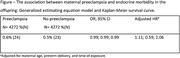

## 741 | Menopausal Pregnancy–should there be a Ceiling for Advanced Maternal Age?

Dvora Kluwgant^1^; Tracy B. Grossman^2^; Caroline Andy^3^

*^1^NYPBrooklyn Methodist Hospital ‐ ‐ Brooklyn, NY, Brooklyn, NY; ^2^NYPBrooklyn Methodist Hospital ‐ Brooklyn, NY, New York, NY; ^3^Weill Cornell Medical School, New York, NY*

10:30 AM ‐ 12:30 PM

**Objective**: It has been well described that pregnancies in women who are ≥ age 35 carry an increased risk of adverse maternal and neonatal outcomes.  The age at which patients are becoming pregnant through reproductive technology has steadily been increasing to age 50 and beyond, potentially pushing the boundaries of what may be considered a safe age for pregnancy.  Because pregnancies in this age range are not well studied, our objective was to describe the rates of adverse pregnancy outcomes of patients age 50 and older and compare them to previously reported rates of outcomes in advanced maternal age (AMA) patients age ≥ 35 and < 50 years.

**Study Design**: This is a retrospective observation study describing maternal and neonatal outcomes of pregnancy ≥ age 50 from 2019–2024 at our institution. The rates of adverse pregnancy outcomes were compared to previously reported outcome data from large meta‐analyses of AMA pregnancy ≥ 35 and < 50 years. Demographic factors, pregnancy and neonatal outcomes were obtained from medical records.

**Results**: 15 patients were included with a total of 14 live births.   Median gestational age at conception was 51 years old, with an age range from 51‐65 years.  Compared to previously reported outcome data of pregnancies in patients ≥ 35 and < 50 years, there was a statistically significant increase in the rate of hypertensive disorders of pregnancy (26.7% vs. 3.1%; p‐value < 0.001) and cesarean delivery (73.3% vs 28.7%; p‐value < 0.001).  Rates of NICU admission and preterm birth were also increased, but not significantly different among the two groups.  

**Conclusion**: There was an increased risk of some adverse pregnancy in patients age 50 or above compared to AMA patients younger than 50. Due to the rarity of these pregnancies, evidenced‐based protocols to optimize pregnancy outcomes in postmenopausal patients have not yet been developed.  Patients planning pregnancy at age 50 or later should be strongly cautioned about the increased risk of adverse outcomes, and alternative options should be considered.

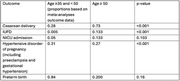

## 742 | Utility of Prenatal Nutrition Consult in Achieving Recommended Weight Gain in Diamniotic Twin Pregnancies

Elena Lands^1^; Andrea Pelletier^2^; Rachel L. Wood^3^; Carolina Bibbo^4^

*^1^University of Pittsburgh Medical Center Magee‐Womens Hospital, Pittsburgh, PA; ^2^Brigham & Women's Hospital, Boston, MA; ^3^Duke University, Durham, NC; ^4^Brigham and Women's Hospital, Boston, MA*

10:30 AM ‐ 12:30 PM

**Objective**: To determine if prenatal nutrition counseling affected maternal weight gain and birth outcomes in diamniotic pregnancies

**Study Design**: A retrospective cohort of diamniotic twin pregnancies cared for by maternal‐fetal medicine specialists at one academic institution from January 2021 to December 2022 was collected. Patients were grouped based on whether they received a nutrition consult or not. Institute of Medicine (IOM) goals for twin pregnancies were used to determine appropriate weight gain based on pre‐gravid BMI. Comparisons were performed with Chi‐square, t‐tests, and multivariable logistic regression adjusted for characteristics that were significantly associated with the outcome.

**Results**: Our cohort included 109 patients with and 70 without a nutrition consult. The rate of monochorionicity was significantly higher in the nutrition group at 49 versus 13% (p< 0.001). The mean pre‐gravid BMI was significantly lower in the nutrition group (26 vs. 28.9, p = 0.006). The groups were otherwise similar (Table 1).

Nutrition consult was associated with a significantly higher mean total weight gain (17.9 vs. 13.4 kg, p< 0.001) and a significantly higher rate of meeting IOM goals (59 vs. 29%, p = 0.003). Mean gestational age at delivery was 5.6 days later in the nutrition group (p = 0.04). When adjusted for age, race, parity, marital status, pregravid BMI, and chorionicity, those who did not receive a nutrition consult had over doubled odds of delivery < 34 weeks (aOR 2.65, 95% CI 1.12‐6.23) and of a very low birthweight neonate (aOR 2.34, 95% CI 1.19‐4.62) (Table 2).

**Conclusion**: Prenatal nutrition consult in diamniotic twin pregnancies resulted in a significantly higher proportion of patients meeting IOM weight gain goals, later gestational age at delivery with significantly fewer births before 34 weeks, and lower risk of very low birthweight neonates. This supports the importance of prenatal nutrition consult for twin pregnancies.

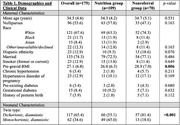

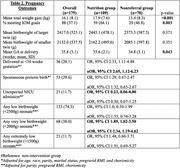

## 743 | Association Between Adverse Pregnancy Outcomes and Awareness of Cardiovascular Risk Factors: a Numom2b‐HHS Secondary Analysis

Elizabeth Wendl^1^; Lauren H. Theilen^2^; Amanda A. Allshouse^2^; Philip Greenland^3^; Lisa D. Levine^4^; William A. Grobman^5^; Natalie A. Cameron^3^; George R. Saade^6^; On behalf of the NuMoM2b‐HHS Network

*^1^University of Utah, North Salt Lake City, UT; ^2^University of Utah, Salt Lake City, UT; ^3^Northwestern, Northwestern/Chicago, IL; ^4^University of Pennsylvania Perelman School of Medicine, Philadelphia, PA; ^5^The Ohio State University, Columbus, OH; ^6^Macon & Joan Brock Virginia Health Sciences Eastern Virginia Medical School at Old Dominion University, Norfolk, VA*

10:30 AM ‐ 12:30 PM

**Objective**: Adverse pregnancy outcomes (APOs) complicate up to 20% of pregnancies and are associated with increased risk of subsequent cardiovascular (CV) disease. We aimed to determine whether having had an APO is associated with greater patient awareness of their own CV risk factors 2‐7 years postpartum.

**Study Design**: This is a secondary analysis of the NuMoM2b‐HHS study. We included participants with a CV risk factor (hypertension [HTN], diabetes/prediabetes [DM], and/or dyslipidemia) identified 2‐7 years after index pregnancy (Table 1). We excluded patients with a CV risk factor during the index pregnancy; a subsequent pregnancy; and patients who did not fast for their blood draw. Exposure was an APO in the index pregnancy: hypertensive disorders of pregnancy, gestational DM, preterm delivery, small‐for‐gestational age, and stillbirth. The primary outcome is a composite of self‐awareness of HTN, DM, or dyslipidemia diagnoses after the index pregnancy utilizing patient questionnaire data. Using logistic regression, we estimated the association between exposure and outcome using three models (unadjusted, fully adjusted, and parsimonious).

**Results**: Among 980 included participants with CV risk factors identified 2‐7 years after the index pregnancy, 77% had only one risk factor with the most common being HTN (34%). APO was present in 35% of patients with the most common being hypertensive disorders of pregnancy. The primary outcome (awareness of a CV risk factor) was present in 22% of women (220/980) ‐ 26% of women with an APO versus 20% without. Prior to adjustment, odds ratio (OR) of APO history and awareness of later CV risk factors was 1.379 (95% CI 1.013‐1.877). After full adjustment and the use of a parsimonious model, there was no significant association between APO history and awareness of later CV risk factors with an OR of 1.346 (95% CI 0.974‐1.859) and 1.313 (95% CI of 0.960‐1.797), respectively (Figure 1).

**Conclusion**: History of APO was not associated with awareness of CV risk factors 2‐7 years later. Women with CV risk factors were infrequently aware of their diagnosis (22%).

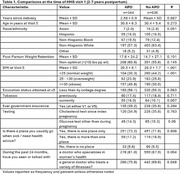

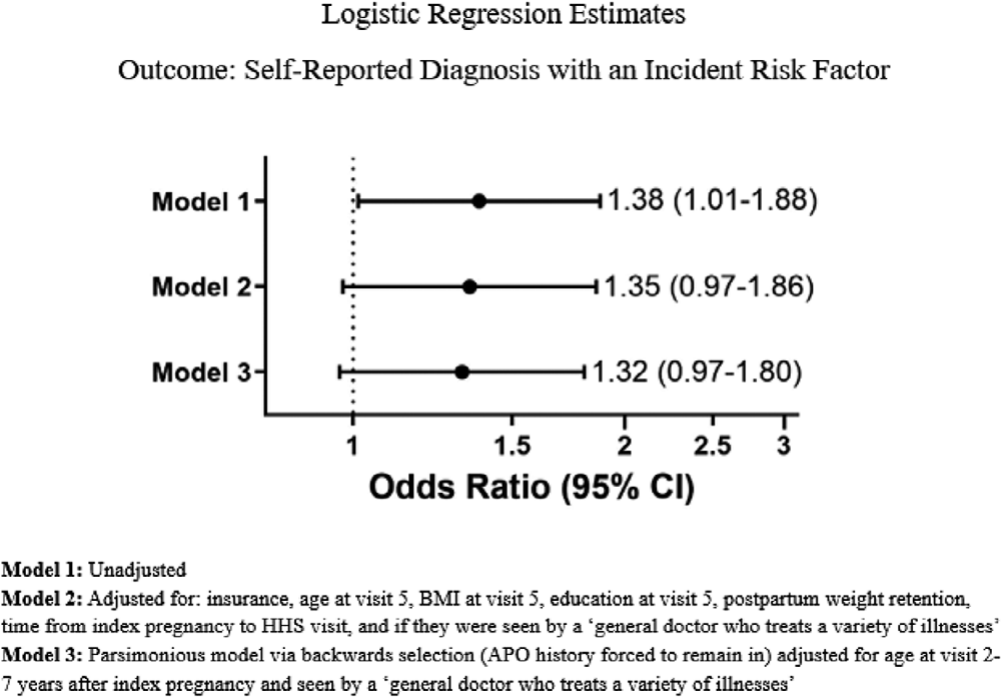

## 744 | Can Doulas Help Improve Equity in the Operating Room?: Clinician Perspectives

Elizabeth Bloom^1^; Caroline Goldfarb^2^; Nicole A. Smith^3^; Alexandria Williams^4^; Rasheca Logendran^2^; Kathleen Clarke^5^; Christina Gebel^6^; Chloe Zera^5^; Elysia Larson^7^

*^1^Beth Israel Deaconess Medical Center, BOSTON, MA; ^2^Harvard Medical School, Boston, MA; ^3^Brigham and Women's Hospital, Boston, MA; ^4^Brigham and Women's Hopsital, Boston, MA; ^5^Beth Israel Deaconess Medical Center, Boston, MA; ^6^Accompany Doula Care, Boston, MA; ^7^Department of Obstetrics and Gynecology, Beth Israel Deaconess Medical Center, Boston, MA*

10:30 AM ‐ 12:30 PM

**Objective**: We aimed to describe obstetric clinical care team members’ perspectives on doulas’ impact on equity for birthing persons in the operating room.

**Study Design**: We used in‐depth, semi‐structured interviews with questions based on the Consolidated Framework for Implementation Research. Using criterion‐I and stratified random sampling, OB/GYNs, labor and delivery nurses, and anesthesiologists were recruited via fliers and emails. Rapid Qualitative Analysis was used for data coding and analysis.  

**Results**: Twenty‐seven clinicians were interviewed: seven anesthesiologists, ten OB/GYNs, and ten labor and delivery nurses. Of these, three quarters expressed a positive view on the impact of doulas on equity, describing benefit from an additional voice advocating for the patient and their goals. Many saw particular benefit for non‐English speaking patients, for whom a language‐concordant doula may significantly improve communication and trust. Participants also often mentioned benefits for their patients of color, who they noted sometimes have distrust in the medical system or may not see themselves reflected in their care team. Participants also observed that doulas improved equity by “translating” across levels of health literacy to help patients understand and accept recommendations, such as the need for cesarean delivery. Clinicians who were uncertain about doulas’ effect on equity cited concerns about possible inequities in the implementation of doula support (cost of doula care, variation in doula experience), while the two clinicians who endorsed a negative view expressed doubt that doulas improve equity, one of whom specifically expressed skepticism about the benefit of a race‐concordant doula.

**Conclusion**: Most clinicians view doulas as beneficial in improving equity for patients undergoing cesarean delivery. These benefits mirror those of prior qualitative work exploring general benefits of doulas for equity, specifically aligning with themes of patient agency, knowledge, connectedness, and respect. Further work should examine impact of doulas on equitable experiences and outcomes in cesarean deliveries.

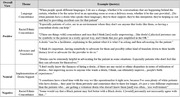

## 745 | Peripartum Hysterectomy: evolving trends over 50 years in a single European city

Elizabeth Tunney^1^; Karen Flood^2^; Ronan Daly^1^; Shahad Al‐Tikriti^3^; Carmen Regan^1^; Declan Keane^4^; Mike P. Geary^5^; Fergal D. Malone^6^

*^1^Royal College of Surgeons in Ireland, Dublin, Dublin; ^2^Royal College of Surgeons in Ireland, Dublin, DE; ^3^The National Maternity Hospital, Dublin, Ireland., Dublin, Dublin; ^4^Royal College of Surgeons in Ireland., Dublin, Dublin; ^5^Rotunda Hospital, Dublin, Ireland., Dublin, Dublin; ^6^Royal College of Surgeons in Ireland, Dubiln, Dublin*

10:30 AM ‐ 12:30 PM

**Objective**: The objective of this study was to determine trends in incidence of, and indications for, peripartum hysterectomy (PH) over 50 years at a single European city.

**Study Design**: A retrospective cohort study was performed between 1966 and 2022 of PH cases performed  in the three large tertiary obstetric hospitals of a single European city. After identifying all cases, data were collected from the published clinical reports of all three hospitals.

**Results**: During the 56 years, there were 1,332,115 deliveries, with 596 PH performed (0.5/1,000 deliveries). When comparing study periods, PH incidence in the most recent seven year period 2016‐2022 (0.74/1,000 deliveries) has returned to 1966‐1975 levels (0.85/1,000 deliveries) despite transient reduced incidence between 1986‐2006 (0.2/1,000 deliveries). The indications for PH have changed significantly over the years. While uterine rupture was the main indication for PH in early years, it is now rare decreasing from 41% to 1% (p< 0.001). Placenta accreta spectrum (PAS) has significantly overtaken other indications for PH from 5% to 32% (p< 0.001). The focus towards antenatal diagnosis of PAS and optimizing surgical management is reflected in our study, with a notable increase in rates of elective PH from 14% to 26% (p< 0.001). A strong correlation was observed between the cesarean delivery rate (6% ‐ 37%) and the prevalence of peripartum hysterectomy in this large obstetric population (p< 0.001).

**Conclusion**: The influence of increased cesarean deliveries internationally is reflected in the increasing incidence of PH, largely for PAS cases and the increased role of elective PH. Our data serve to inform how emergency obstetric practice has evolved over five decades and in turn may be helpful in the focus on training required for PH in the future.

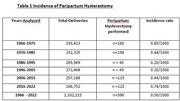

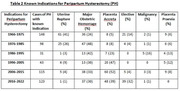

## 746 | Optimal Aspirin Dose in Preeclampsia Prevention: A Retrospective Comparison of 81 mg and 162 mg

Ellen M. Murrin^1^; Scott Sullivan^2^; Garima Sharma^3^; Olayinka Agboola^2^; G. Larry Maxwell^2^; George R. Saade^4^; Antonio F. Saad^5^

*^1^Inova Fairfax Medical Campus, Falls Church, VA; ^2^Inova Fairfax Medical Campus, Fairfax, VA; ^3^Inova Heatlh, Norfolk, VA; ^4^Macon & Joan Brock Virginia Health Sciences Eastern Virginia Medical School at Old Dominion University, Norfolk, VA; ^5^Inova Health, Falls Church, VA*

10:30 AM ‐ 12:30 PM

**Objective**: There is a lack of consensus regarding the optimal dosing for aspirin (ASA) in preeclampsia prevention, with recommendations ranging from 75 to 162 mg daily. This practice reflects evidence suggesting that dosages above 100 mg are most effective at reducing preeclampsia risk. Our objective was to compare the incidence of preeclampsia in patients taking 81 mg versus 162 mg ASA daily using national data.

**Study Design**: A retrospective cohort study was conducted using the Cosmos Epic database. Data from pregnancies between June 2021 and July 2024 in which ASA was prescribed were pooled based on ICD‐10 codes.  Baseline clinical characteristics and risk factors were compared between groups. Two‐sided chi‐square tests and odds ratios were used for statistical analysis.

**Results**: A total of 328,310 pregnant patients taking ASA were identified—81.2% in the 81 mg group and 18.8% in the 162 mg group. The 162 mg ASA group had significantly higher rates of BMI ≥30, multiple gestation, pre‐gestational diabetes, antiphospholipid syndrome, chronic kidney disease, and chronic hypertension (Table 1). Obstetric outcomes were poorer in this group, with increased rates of cesarean delivery and birth weight < 2500 g. More patients in the 162 mg group developed preeclampsia compared with the 81 mg group (OR 1.31, 95% CI 1.29‐1.35). This difference remained significant despite accounting for the above‐mentioned confounding variables (aOR 1.11, 95% CI 1.11‐1.13; Table 2).

**Conclusion**: In this national dataset, a higher daily dose of aspirin (162 mg) was not more effective than a lower dose (81 mg) in reducing the risk of preeclampsia, despite correcting for differences in risk factors that may have prompted clinicians to use a higher dose. More research is needed to confirm these findings and optimize aspirin dosing guidelines for preeclampsia prevention.

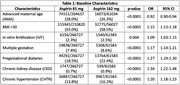

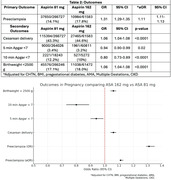

## 747 | Impact of Early Zygosity Determination via NIPT on Maternal and Neonatal Outcomes in Twin Pregnancies

Ellen M. Murrin^1^; Elizabeth Levit‐Smith^2^; Helen Havens Howell^3^; Alyssa Savelli Binsted^4^; Peyton Kalan^2^; Emma Cherayil^2^; Tetsuya Kawakita^4^; George R. Saade^4^; Antonio F. Saad^5^

*^1^Inova Fairfax Medical Campus, Falls Church, VA; ^2^Inova Fairfax Medical Campus, Fairfax, VA; ^3^University of Virginia College of Medicine, Charlottesville, VA; ^4^Macon & Joan Brock Virginia Health Sciences Eastern Virginia Medical School at Old Dominion University, Norfolk, VA; ^5^Inova Health, Falls Church, VA*

10:30 AM ‐ 12:30 PM

**Objective**: Monochorionic (MC) twins have significant morbidity compared with dichorionic twins (DC) due to their risk for unequal placental sharing. Non‐invasive prenatal testing (NIPT) can now determine zygosity, which often informs chorionicity. This study examines pregnancy outcomes in monozygous (MZ) and dizygous (DZ) pregnancies and the correlation between zygosity and chorionicity.

**Study Design**: A retrospective cohort study of twin pregnancies with NIPT zygosity results at two tertiary care centers from January 2020 to 2024 was conducted. Demographics, pregnancy complications, NIPT results, fetal fractions, placental pathology, and delivery outcomes were collected. DZ twins were classified as DC while chorionicity within the MZ group was determined by placental pathology. Statistical tests such as Chi‐square, Fisher exact, Wilcoxon rank sum test, or logistical regression were used as appropriate.

**Results**: In 177 twin pregnancies with NIPT zygosity testing, 66.3% were DZ and 33.7% MZ. Baseline characteristics did not differ among groups except for race and gestational age. After adjusting for these variables, a subgroup analysis was performed to compare MZ DC and MZ MC to DC subtypes. Within the MZ group, 8 (17%) were DC and 38 (82.6%) were MC. MZ MC twins were significantly more likely to experience neonatal morbidities such as hypoglycemia and hyperbilirubinemia requiring phototherapy, NICU admission, and additional respiratory support (Figure & Table). The MZ DC group was not more likely to experience these outcomes. Composite neonatal morbidity was also more common in the MZ MC group (aOR 6.01 [1.89‐10.12], p = 0.002). Maternal outcomes did not differ between groups.

**Conclusion**: The early differentiation of chorionicity and zygosity allows for the implementation of tailored care strategies to enhance outcomes. Our study shows that MZ MC twins have significantly higher neonatal morbidity, thus underscoring the necessity for vigilant monitoring and intervention.

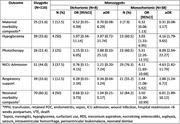

## 748 | The Effect of Glucose Challenge and Oral Glucose Tolerance Tests Timing on Tests' Results

Enav Yefet^1^; Zohar Nachum^2^

*^1^Tzafon Medical Center, Poriya, HaZafon; ^2^Emak Medical Center, Afula, HaZafon*

10:30 AM ‐ 12:30 PM

**Objective**: The usual time for performing glucose challenge test (GCT) and oral glucose tolerance test (OGTT) is between 24‐28 gestational weeks. After which, it is not known whether GCT or OGTT act in the same manner.

In the present study we compared the results of GCT and OGTT when the tests were done between 24‐28 gestational weeks or later.   

**Study Design**: A population‐based retrospective cohort study of women who performed GCT between June 2013 and December 2021. In our institution GCT is recommended even after 28.6 weeks to women who did not perform it before. Women with large for gestational age fetus or polyhydramnios were excluded, since OGTT is performed in those cases. The rates of abnormal GCT (≥140 mg%) and gestational diabetes mellitus (GDM) were compared. GDM was defined as GCT≥200 mg% or at least 2 abnormal OGTT values according to the Carpenter and Coustan criteria.

**Results**: Data were collected for 20,485 women with GCT between 24‐28.6 weeks and 1,806 with GCT thereafter. GCT results for every gestational week are presented in the figure. The rate of abnormal GCT and GDM were similar between the groups (abnormal GCT: 3797 (18.5%) versus 323 (17.9%), respectively; P = 0.49, and GDM: 1063 (5.2%) versus 86 (4.8%), respectively; P = 0.43)

**Conclusion**: In low risk women, GCT values and the rate of GDM are similar from 24 weeks and thereafter.

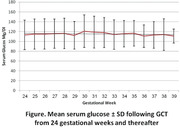

## 749 | Time from Decision to Execution of Manual Exploration of the Uterus and Postpartum Hemoglobin Drop

Enav Yefet^1^; Dana Segal^2^; Omri Zarka^2^; Zohar Nachum^3^

*^1^Tzafon Medical Center, Poriya, HaZafon; ^2^Emek medical center, Afula, HaZafon; ^3^Emak Medical Center, Afula, HaZafon*

10:30 AM ‐ 12:30 PM

**Objective**: Manual exploration of the uterus is performed in women with postpartum hemorrhage to remove blood clots, retained placenta and to repair obstetrical tears. There is very limited data to evaluate the time from when the decision of conducting the manual exploration is made to the actual procedure to avoid complications.

**Study Design**: A retrospective cohort study conducted between 2013 and 2019. The data was collected from women in the maternity ward who underwent a manual exploration of the uterus after a vaginal birth. A comparison was made between women in whom the time from making the decision of performing manual exploration to the time of the procedure was more than 20 minutes (>20 minutes cohort) versus up to 20 minutes (≤20 minutes cohort). The primary outcome was the rate of women with hemoglobin (Hb) decrease of at least 3 g/dL after delivery.

**Results**: Among 696 women who were analyzed, 224 and 472 women underwent manual exploration >20 minutes and ≤20 minutes, respectively. Characteristics and outcomes are presented in tables 1 and 2. Mean time from decision to performing manual exploration was 45±54.8 and 9.5±5.6 minutes in the >20 minutes and ≤20 minutes cohorts, respectively (P< .0001). Mean Hb decrease was greater in the >20 minutes cohort versus ≤20 minutes cohort (3.4 ±1.7 gr∖dl versus 1.8±3.0  gr∖dl, respectively; P = 0.02). The rate of Hb decrease≥3 g/dL was 59% and 50% in the >20 minutes and ≤20 minutes cohorts, respectively (P = 0.06). No other significant differences were observed. The cutoff for performing manual exploration to predict Hb decrease of at least 3 g/dL was 19 minutes (47% sensitivity, 54% specificity)

**Conclusion**: While it seems that the medical staff is skilled in managing the optimal time to perform manual exploration of the uterus, the time from decision to procedure should be up to 20 minutes in order to avoid excessive blood loss.

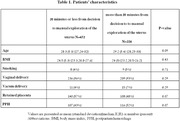

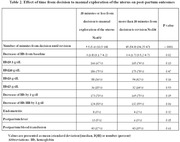

## 750 | Gestational Diabetes Mellitus in Pregnancies with Suspected Growth Restriction: Blessing or Curse?

Misgav Rottenstreich^1^; Sahra Nathoo^1^; Bryon DeFrance^2^; Jon F. Barrett^2^; Eran Ashwal^2^

*^1^McMaster University, Hamilton, ON; ^2^McMaster university, Hamilton, ON*

10:30 AM ‐ 12:30 PM

**Objective**: Fetal growth restriction (FGR) is a condition that occurs when a fetus fails to achieve its growth potential. Gestational diabetes mellitus (GDM) is another common complication during pregnancy associated with accelerated fetal growth. This study aims to evaluate the impact of GDM on perinatal outcomes in pregnancies with suspected fetal growth impermanent.

**Study Design**: This retrospective cohort study enrolled singleton pregnancies without fetal genetic or structural abnormalities and EFW and/or AC below the 10^th^ centile for gestational age on the last sonographic assessment within 14 days from delivery. The study compared pregnancies with GDM to those without GDM. The primary outcome of interest was the o ccurrence of composite adverse perinatal outcomes. Multivariable logistic regressions were performed, adjusting for relevant covariates.

**Results**: A total of 1,583 patients were included in the study, with 234 (14.8%) diagnosed with GDM, while 1,349 (85.2%) did not . Patients with GDM were found to be older, had a higher body weight, and had higher rates of hypertensive disorders compared to those without GDM. Patients with GDM experienced longer pregnancies, with a difference of approximately one week, but there were no significant differences in the rates o f preterm labor (< 37 weeks). The rates of labor induction and mode of delivery were comparable between the GDM and non‐GDM groups. Neonates born to mothers with GDM had higher birthweight and lower adverse perinatal outcome rates than neonates of normoglycemic pregnancies (45.2% vs. 52.2%; p = 0.04). In the multivariate analysis, which controlled for maternal age, parity, oligohydramnios, hypertensive disorders of pregnancy, gestational age at delivery, and birthweight, it was found that GDM was significantly and independently associated with lower adverse perinatal outcome (adjusted odds ratio = 0.65, 95% confidence interval 0.47‐0.9) (Table).

**Conclusion**: GDM significantly impacts perinatal outcomes in pregnancies with suspected fetal growth impairment and can potentially improve the overall outcome in such cases.

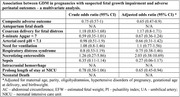

## 751 | Addressing Medical Mistrust Among Black Birthing People Through Community‐Based Doulas

Erica Marion^1^; Katherine Quinn^1^; Jessica Olson^1^; Dalvery Blackwell^2^; Joni Williams^1^; Anna Palatnik^1^

*^1^Medical College of Wisconsin, Milwaukee, WI; ^2^African American Breastfeeding Network Inc., Milwaukee, WI*

10:30 AM ‐ 12:30 PM

**Objective**: Structural and systematic barriers within healthcare systems are associated with medical mistrust between Black birthing people and healthcare providers. This study explored how partnership with community‐based doulas can address medical mistrust between Black birthing people and obstetric healthcare teams.

**Study Design**: A prospective qualitative study that included focus groups and in‐depth interviews using a semi‐structured interview guide with OBGYNs (n = 7), Maternal‐Fetal Medicine specialists (n = 2), certified nurse midwives (n = 2), a perinatal psychologist (n = 1), a prenatal care coordinator (n = 1), clinical and inpatient nurse supervisors (n = 3), birth center nurses (n = 2), an OB‐Anesthesiologist (n = 1), and community‐based doulas (n = 11). Interviews and focus groups were recorded, transcribed verbatim, coded using MAXQDA software, and analyzed using thematic content analysis to understand how community‐based doulas can contribute to improving patient‐clinician relationships specifically for Black birthing people.

**Results**: Analysis yielded the following themes: 1) Doulas have a unique opportunity to sustain patient trust through continuous social support and continuity of care; 2) Doulas serve as liaisons and advocates   between Black birthing people and healthcare teams to assist with navigating conflict and facilitating communication; and 3) Doulas of color provide a unique perspective and shared experience that can foster trust and understanding for both patients and healthcare teams.  As a result, patient‐clinician communication can be improved by community‐based doulas facilitating bidirectional understanding to reduce healthcare mistrust.

**Conclusion**: Our results highlight the vital role of community‐based doulas in mitigating medical mistrust between healthcare professionals and Black birthing people. Data obtained from this qualitative analysis will inform the design of a collaborative community‐based doula‐clinician model focused on improved communication and meaningful engagement to foster trust.

## 752 | Pregnancy Health Information‐Seeking Behaviors and Knowledge of Pregnancy Risks among Male Partners of Pregnant Patients

Erin Kim^1^; Joshua Bellisario^2^; Fanglong Dong^3^; Kristina Galyon^4^; Hindi Stohl, JD^4^

*^1^Western University of Health Sciences COMP Pomona, Los Angeles, CA; ^2^Harbor‐UCLA Medical Center, Los Angeles, CA; ^3^Western University of Health Sciences, Los Angeles, CA; ^4^UCLA David Geffen School of Medicine Harbor‐UCLA Medical Center, Los Angeles, CA*

10:30 AM ‐ 12:30 PM

**Objective**: Partners play an active role in supporting pregnant patients with prenatal and perinatal decisions, yet there are few studies on this population. This study examines the information seeking behavior and pregnancy risk knowledge of male partners of pregnant patients.

**Study Design**: An IRB‐approved 45‐question survey was administered in 2017 to male partners of pregnant patients at Harbor‐UCLA Medical Center prenatal clinics. Men were interviewed one‐on‐one in private areas of the clinic.  Statistical significance was determined using a chi‐squared test with p < 0.05.

**Results**: 127 male partners completed the survey. 86.4% of male partners could not name one of the major pregnancy‐related health risks in an open‐ended question format. When asked about seven specific pregnancy‐related health risks from a multiple‐choice list, only 5.6% of male partners were able to identify all of them correctly. Noticeably, 41.6% were able to correctly identify the increased risk of the two most common complications: diabetes and hypertension. These findings were not associated with higher education (*P* = 0.5055) or fathering a prior pregnancy (*P* = 0.4831). When asked whether they would like more education about pregnancy, 82.1% responded yes, 40.2% of whom wanted the information from their partner's healthcare provider. Male partners who were not interested in learning more about pregnancy had a significantly lower score of correctly reporting the seven specific pregnancy risks (*P* = 0.0003).

**Conclusion**: Our study highlights that a large percentage of male partners of pregnant women are unaware of the health risks associated with pregnancy. Additionally, male partners who are less information‐seeking about pregnancy also have lower knowledge levels of pregnancy risks, underscoring the need for improved pregnancy education.

## 753 | Conversion to Telehealth During COVID‐19 and Depression Symptom Response in a Perinatal Collaborative Care Model

Esther Y. Son^1^; Mayán Alvarado‐Goldberg^2^; Allie Sakowicz^3^; Siyuan Dong^1^; Emily S. Miller^4^; Dorothy Sit^1^; LaTasha Nelson^1^; Michelle A. Kominiarek^1^

*^1^Northwestern Feinberg School of Medicine, Northwestern Feinberg School of Medicine/ Chicago, IL; ^2^Northwestern University, Northwestern University/ Evanston, IL; ^3^Wake Forest University School of Medicine, Winston‐Salem, NC; ^4^Women & Infants Hospital of Rhode Island and Alpert Medical School of Brown University, Providence, RI*

10:30 AM ‐ 12:30 PM

**Objective**: We aimed to assess the association between conversion to telehealth and depression symptom response in a perinatal collaborative care model‐based treatment (PCCM).

**Study Design**: This was a retrospective study of pregnant and postpartum people from 2017‐2022 who engaged in a PCCM for perinatal mental health care. Outcomes were analyzed by epoch, defined as pre‐telehealth (preT) (2017‐2020) and post‐telehealth (postT) (2020‐2022) cohorts, based on when PCCM converted to telehealth as a result of the COVID‐19 pandemic. Sociodemographic (age, race/ethnicity, parity, substance use) and perinatal data (gestational age at delivery, delivery route) and all PHQ‐9 scores were abstracted from the EHR. Bivariate and multivariate analyses were performed to compare depressive symptom response over time between preT and postT cohorts in three ways: change in PHQ‐9 score from initial to final, dichotomized PHQ‐9 score improvement from initial to final, and as a linear mixed effects model to analyze trajectories of PHQ‐9 scores adjusting for random effects within each person.

**Results**: Of 1422 people who met eligibility criteria, 679 (48%) engaged in PCCM postT. There were no differences in age, race/ethnicity, parity, tobacco use, gestational age at delivery, and delivery route between cohorts. People in preT were less likely to have prior substance use, but more likely to have medical comorbidities and prior mental health diagnoses compared to postT. Both groups had decreases in PHQ‐9 scores, consistent with improvement in depressive symptoms, but preT had greater mean decreases and were more likely to have an improvement in scores than postT, after adjusting for potential confounders (Table). There was no difference in PHQ‐9 trajectories between the cohorts (Figure).

**Conclusion**: Overall, we observed improvements in depression symptoms in PCCM with greater improvements in mean PHQ‐9 scores in preT compared to postT, but no differences in scores when accounting for random effects in each person, suggesting that mental health services via telehealth offers similar outcomes compared to in‐person visits.

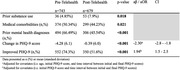

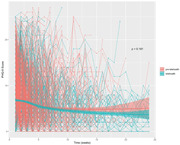

## 754 | Pregnancy During Wartime is Associated with Increased Preterm Rupture of Membranes

Esther Maor‐Sagie^1^; Coral Danenberg^2^; Dima Shabita^3^; Amir Naeh^4^; Rinat Gabbay‐Benziv^3^

*^1^Hille Yaffe medical center, Hillel Yaffe medical canter, Hefa; ^2^Ariel University, The Adelson school of Medicine Ariel University, Yerushalayim; ^3^Hillel Yaffe Medical Center, Hillel Yaffe medical center, Hefa; ^4^Hillel Yaffe Medical Center, Hillel Yaffe Medical Center, HaMerkaz*

10:30 AM ‐ 12:30 PM

**Objective**: Anxiety, depression, and stress during pregnancy negatively impact birth outcomes, including infant weight, head circumference, and APGAR scores. These effects are linked to physiological changes like catecholamine and cortisol secretion, leading to high blood pressure, maternal tachycardia, and increased risk of fetal growth restriction and preterm birth. The Iron Sword war, declared by Israel against Hamas, following the October 7, 2023 attack, has created tremendous psychological stress in the Israeli population. This study aims to assess the consequences of the Iron Sword war on obstetric outcomes related to stress and psychological distress, including preterm labor, premature rupture of membranes, preeclampsia, and preterm births.

**Study Design**: A retrospective cohort analysis was conducted at a single university‐affiliated medical center in Israel. The study included deliveries from October 7, 2023, to November 19, 2023, compared to deliveries during the same period in 2022. Univariate and regression analyses were used to adjust for confounders. Categorical variables were compared using χ^2^ tests, and continuous variables were analyzed with the Mann‐Whitney test. Significance was set at p< 0.05.

**Results**: Overall, 508 women in the study group were compared to 515 women in the control group. There was no difference in gestational age at delivery or neonatal birth weight. Women who delivered during the Iron Sword war experienced a nearly threefold increase in preterm rupture of membranes and 1.5 times more labor inductions, mainly due to rupture of membranes (14.8% vs. 6.3% and 51.4% vs. 34%, respectively, p< 0.001 for both).  Women during the war used more epidural anesthesia (62.4% vs. 48%, p< 0.001) .  Results remained significant after adjusting for maternal age, BMI, nulliparity, and fetus number. No other differences were noted in delivery mode or other perinatal outcomes.

**Conclusion**: The prevalence of premature rupture of membranes increased significantly during the Iron Sword War, potentially linked to maternal stress and anxiety. Further studies are needed to evaluate these outcomes better.

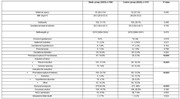

## 755 | Evaluating the Association Between Eczema in Pregnancy and Maternal and Fetal Outcomes

Ethan Bendayan^1^; Andrea R. Spence^2^; Haim A. Abenhaim^3^

*^1^McGill University, Montreal, PQ; ^2^Jewish General Hospital, Montreal, PQ; ^3^Jewish General Hospital, McGill University, Montreal, PQ*

10:30 AM ‐ 12:30 PM

**Objective**: Eczema is a common skin condition on which little literature exists regarding its association in pregnancy and pregnancy‐related outcomes. Our study served to evaluate the association between various obstetric and newborn outcomes and pregnancies affected by eczema.

**Study Design**: Using the Nationwide Inpatient Sample's 2016‐2021 records, a population‐based retrospective cohort study was conducted. To extract pregnancy and delivery records, both affected by eczema and unaffected, ICD‐10 codes were used. ICD‐10 codes were also used to identify baseline characteristics, obstetric outcomes and neonatal outcomes. Adjusting for age and race, multivariate logistic regression models were performed in order to compute odds ratios and p‐values.

**Results**: The sample contained 8634 deliveries affected by eczema and 4,337,612 total deliveries, for an overall prevalence of 199 cases per 100,000 deliveries. Eczema was associated with adverse maternal outcomes, such as sepsis, OR 2.5 (95% CI  2.1‐3.1); need for blood transfusions, 1.8 (1.5‐2.0); pulmonary embolism, 3.2 (1.04‐10.08); length of stay greater than 3 days, 3.0 (2.9‐3.1); preeclampsia, 1.6 (1.4‐1.7); gestational diabetes, 1.1 (1.02‐1.18); anemia, 1.6 (1.5‐1.7); placental abruption, 1.6 (1.4‐1.9); placenta previa, 1.5 (1.2‐1.9); preterm premature rupture of membranes, 1.4 (1.3‐1.5); preterm labor, 1.3 (1.2‐1.4); chorioamnionitis, 2.1 (1.9‐2.3); spontaneous vaginal delivery, 0.53 (0.51‐0.55); caesarean section, 2.1 (2.0‐2.2); instrumental vaginal delivery, 0.69 (0.60‐0.78); and disseminated intravascular coagulation, 3.3 (1.6‐6.5). In newborns, eczema was associated with congenital abnormalities, 2.3 (2.0‐2.7); and intrauterine growth restriction, 1.3 (1.2‐1.5).

**Conclusion**: Pregnancies affected by eczema appear to be at higher risk for various obstetric and neonatal outcomes. Consideration should be given to monitor and manage pregnancies with eczema. Additional research should be conducted on the topic and on potential management.

## 756 | Evaluating the Incidence and Risk Factors of Melanoma and History of Melanoma in Pregnancy

Ethan Bendayan^1^; Andrea R. Spence^2^; Haim A. Abenhaim^3^

*^1^McGill University, Montreal, PQ; ^2^Jewish General Hospital, Montreal, PQ; ^3^Jewish General Hospital, McGill University, Montreal, PQ*

10:30 AM ‐ 12:30 PM

**Objective**: Melanoma is a common form of skin cancer, the incidence of which is uncertain in pregnancy. Previous reports estimated melanoma to be one of the most prevalent cancers in pregnancy. We sought to find the incidence of melanoma or a history of melanoma in pregnancy in a population‐based study.

**Study Design**: The Nationwide Inpatient Sample's 2016‐2021 records were used in order to conduct a retrospective cohort study. To identify pregnancy and delivery records, both affected by melanoma or a history of melanoma and unaffected, ICD‐10 codes were used. ICD‐10 codes were used to isolate the baseline characteristics, as well.

**Results**: The cohort contained 213 admissions with melanoma or a history of it, out of 4,337,612 total delivery admissions. The incidence was calculated to be 4.9 cases per 100,000 deliveries. Of the records with melanoma or history of melanoma, 64% were between 25 and 35 years old and 74% were at, or past, the 50th income percentile.

**Conclusion**: The results suggest that the incidence of melanoma or history of melanoma in pregnancy is extremely low and, unlike previously reported, melanoma is likely one of the least prevalent cancers in pregnancy. Age and income appear to be risk factors for developing melanoma, or having a history of melanoma, in pregnancy. Larger scale studies should be conducted in order to verify the degree of association.

## 757 | The Correlation Between Extent of Invasion in Placenta Accreta Spectrum and Units of Blood Transfused

Eva Hoffmann; Brian Z. Druyan; Carolyn Wheeler; Anna K. Sfakianaki; Paloma Toledo; Michael J. Paidas; Rodrigo Ruano; Pouya Abhari

*University of Miami, Miami, FL*

10:30 AM ‐ 12:30 PM

**Objective**: The primary objective of this study is to determine if there is an association between the extent of invasion in Placenta Accreta Spectrum (PAS) as identified on pre‐operative imaging and transfusion of packed red blood cells (pRBCs). By establishing this correlation, the study aims to enhance preoperative preparation for patients with PAS and reduce the waste of healthcare resources by avoiding the preparation of unnecessary blood products.

**Study Design**: This was a retrospective cohort study, approved by the Institutional Review Board. The study was conducted at a single accreta center of excellence from April 1, 2010 through January 9, 2023. All patients diagnosed pre‐operatively with PAS via ultrasound were included. We excluded patients who did not have clear ultrasound diagnosis of PAS with extent of invasion documented. Data was collected from electronic medical records, including maternal demographics, pre‐operative ultrasound findings, pre‐operative hemoglobin level, blood loss and pRBCs.

Descriptive statistics were used to summarize maternal demographics and clinical outcomes as well as parametric and non‐parametric statistics to compare groups. Data analysis was performed using SPSS version 29.0.2.0. P‐values < 0.05 was statistically significant.

**Results**: Prenatal ultrasound was used to identify 160 patients with PAS with extent of invasion identified as focal accreta (N = 9), accreta (N = 97), increta (N = 29), and percreta (N = 25). There were no differences across demographic, age, race, BMI, number of prior cesarean deliveries and preoperative hemoglobin. There were no differences found in units of pRBCs between groups or estimated blood loss.

**Conclusion**: In conclusion we found no differences in units of pRBCs transfused and blood loss despite differences in preoperative diagnosis. This may be related to overall increased preparedness at a placenta accreta center of excellence with multi‐disciplinary care. This study supports standard protocols and uniformity in available blood despite differences in preoperative diagnosis.

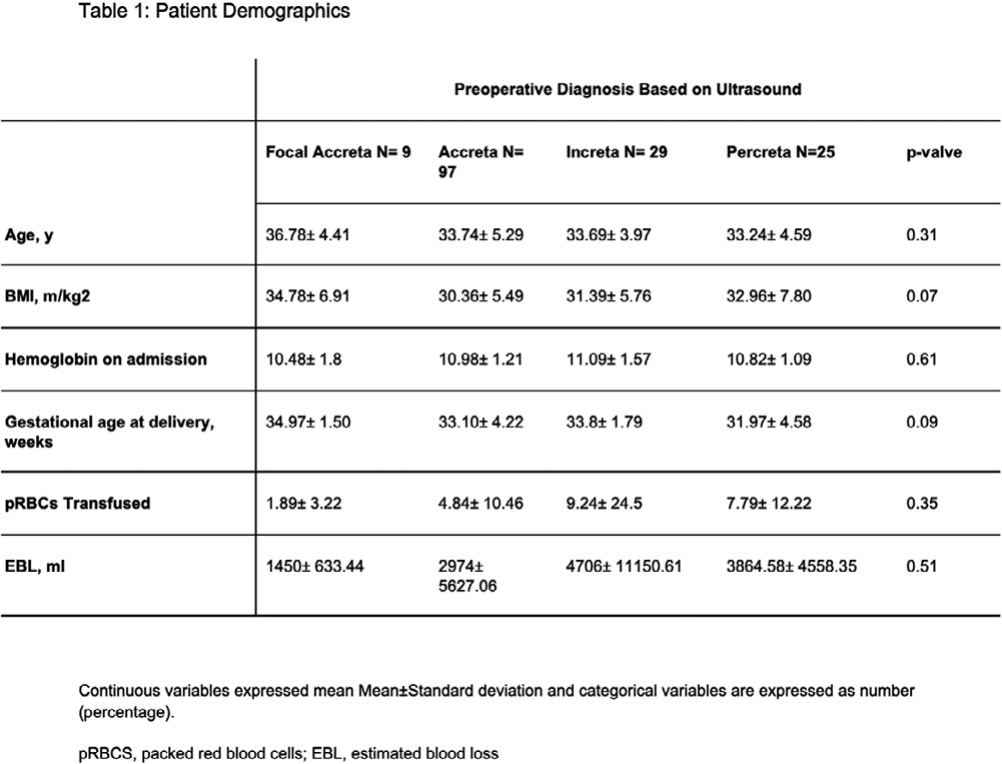

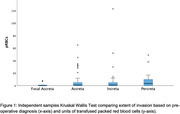

## 758 | Changes in Fetal Heart Rate Baseline For Deliveries 22 to 31.6 Weeks and Adverse Outcomes

Fabrizio Zullo^1^; Roby Lauren^2^; Cassidy A. O'Sullivan^2^; Ha L. Tran^3^; Giuseppe Rizzo^4^; Kelley Kovatis^2^; Matthew K. Hoffman^2^; Anthony C. Sciscione^2^; Suneet P. Chauhan^5^

*^1^University of Rome La Sapienza, Rome, Lazio; ^2^Christiana Care Health System, Newark, DE; ^3^Nemours Children's Hospital, Wilmington, DE; ^4^Sapienza Università di Roma (ROMA), Rome, Lazio; ^5^Delaware Center of Maternal‐Fetal Medicine at Christiana Care, Delaware, DE*

10:30 AM ‐ 12:30 PM

**Objective**: The significance of a change in baseline fetal heart rate tracing (FHRT) is unclear. We aim to estimate the incidence of change in FHR baseline in deliveries at 22.0 to 31.6 weeks gestational age (wga) and associated neonatal morbidity or mortality.

**Study Design**: A retrospective review of all deliveries from Jan to Dec  2023 at a tertiary care referral hospital. The inclusion criteria included delivery at 22‐31 wga, of a non‐anomalous singleton, where at least 40 minutes of a FHRT was available, and neonatal resuscitation was initiated. A physician—blinded to maternal characteristics and peripartum outcomes—reviewed the FHRT (0‐120 min proximal to delivery, at 20 min epochs).  FHR baseline variation was assessed by calculating a delta (D) between the last and the first epoch. Neonatal outcomes were abstracted. The primary outcome was long term neonatal morbidity or mortality (LTNMM); the secondary outcomes were Apgar score of 4‐6 at 5‐min and short‐term neonatal morbidity or mortality (STNMM). Descriptive statistics with 95% confidence intervals (CI) were calculated, and non‐overlapping CI was considered significant.

**Results**: Among the 6,521 deliveries, 169 (2%) occurred at 22‐31.6 wga, of which 97 (57%) met the inclusion criteria. Magnesium sulfate was administered in 97% of the newborns and antenatal corticosteroids in 98%. A positive variation (+ D)  in baseline occurred in 26/97 (26%) patients, and the likelihood of LTNMM in this cohort was 9/26 (34%; 95% CI 18.4‐55.3). A negative variation (‐ D) in baseline occurred in 29/97 patients (29%), and the likelihood of LTNMM in this cohort was 11/29 (37%; 95% CI 21‐57).

A neutral variation (D = 0) occurred in 42/97 (43.3%) of cases with a LTNM rate of 18/42 (42%; 95% CI 2858. Each analyzed cohort, even when stratified for discreet changes, had overlapping CI for all outcomes examined (Table 1).

**Conclusion**: Among newborns delivered at 22.0 to 31.6 weeks, change in baseline was noted in the majority and, irrespective of direction, it was not associated with adverse fetal outcome.

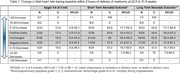

## 759 | Income‐Level and Regional Variations and the Impact of Cesarean Delivery Rates on Maternal Mortality

Fabrizio Zullo^1^; Roby Lauren^2^; Teresa C. Logue^2^; Daniele Di Mascio^3^; Sara Sorrenti^3^; Antonella Giancotti^3^; Giuseppe Rizzo^3^; Suneet P. Chauhan^4^

*^1^University of Rome La Sapienza, Rome, Lazio; ^2^Christiana Care Health System, Newark, DE; ^3^Sapienza Università di Roma (ROMA), Rome, Lazio; ^4^Delaware Center of Maternal‐Fetal Medicine at Christiana Care, Delaware, DE*

10:30 AM ‐ 12:30 PM

**Objective**: To investigate the relationship between cesarean delivery (CD) and maternal mortality ratio (MMR) across different income levels and UNICEF regions.

**Study Design**: We conducted a cross‐sectional analysis from 157 countries pooled from UNICEF database. To analyze interactions, we utilized a linear regression model. The model was built to account for variations due to income classifications by including dummy variables for each income level ‐Low Income (LI), Lower Middle Income (LMI), Upper Middle Income (UMI), and High Income (HI) as defined by the World Bank. We controlled for the impact of income on MMR while isolating the effect of CD rates. Descriptive statistics and correlation analysis were used.

**Results**: Most UNICEF regions exhibit a negative correlation between CD rates and MMR, with the strongest correlations in Sub‐Saharan Africa (‐0.84) and South Asia (‐0.68). In contrast, Eastern Europe and Central Asia show a weak positive correlation (0.43), indicating a slight increase in MMR with higher CD rates. When stratified for income group, CD rates were negatively correlated with MMR across all income groups but HI. The strongest negative correlation (‐0.61) in LI countries indicates a significant decrease in MMR with higher CD rates. HI countries show a weak positive correlation (0.14), suggesting a slight increase in MMR with higher CD rates (Table 1).  The regression analysis revealed that each % increase in CD rates corresponds to a reduction of approximately 4.4 units in MMR (p < 0.001). MMR was significantly higher in LI countries (coefficient: 370.18, p < 0.001) and LMI countries (coefficient: 159.06, p < 0.001) compared to HI countries. UMI countries had a coefficient of 61.84 with a p‐value of 0.079, indicating no clear association with MMR (Table 2).

**Conclusion**: Increasing the rate of CD was associated with a significant reduction in MMR in LI countries. In HI countries the correlation is weakly positive. This suggests that in LI countries an increase in CD rate may lower MMR; conversely in HI countries an increase in CD may be associated with increased MMR.

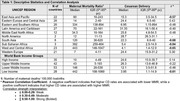

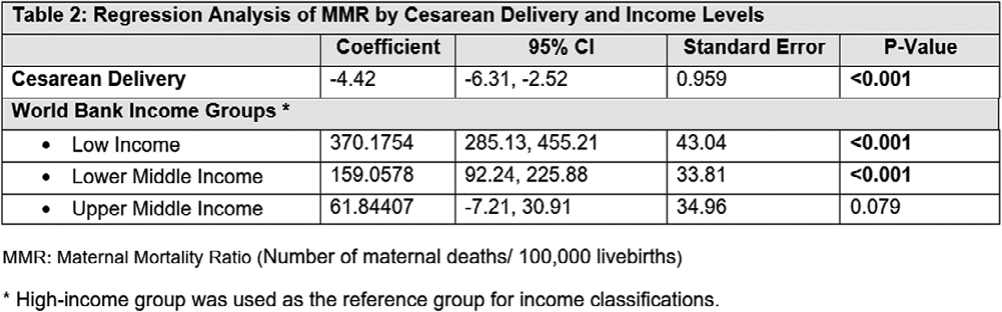

## 760 | Perioperative Risk Factors for Amniotic Band Sequence Following Fetoscopic Laser Photocoagulation for Twin‐Twin Transfusion Syndrome

Felicia V. LeMoine^1^; Sami Backley^1^; Gustavo Vilchez^2^; Jimmy Espinoza^1^; Anthony Johnson^3^; Ramesha Papanna^4^; Eric P. Bergh^5^

*^1^Division of Fetal Intervention, Department of Obstetrics, Gynecology and Reproductive Sciences, McGovern Medical School at UTHealth Houston, Houston, TX; ^2^UTHealth Houston, Houston, TX; ^3^McGovern Medical School University of Texas Health Science Center at Houston (UThealth), Houston, TX; ^4^McGovern Medical School at UTHealth Houston, Houston, TX; ^5^UT Health, Houston, TX*

10:30 AM ‐ 12:30 PM

**Objective**: Observational studies have suggested associations between perioperative characteristics such as gestational age (GA) at fetoscopic laser photocoagulation FLP, chorioamnion separation (CAS), and iatrogenic septostomy (IS) and amniotic band sequence (ABS) after FLP for twin‐twin transfusion syndrome (TTTS). We aim to evaluate the relationship between perioperative factors and ABS in monochorionic, diamniotic (MCDA) twins undergoing FLP for TTTS treatment.

**Study Design**: A secondary analysis of a prospective cohort study involving 816 cases of FLP (2011‐2024) was conducted. Detailed ultrasound evaluation before FLP showed absence of ABS for all cases. ABS was either identified by post‐FLP imaging and confirmed postnatally or identified on postnatal exam. Perioperative factors were compared using t‐test, χ2 test, and Fisher's exact test, where applicable. Multivariate logistic regression was performed to adjust for the following covariates: intertwin discordance, GA at FLP, CAS diagnosed on post‐op day 1 scan, amnioinfusion, and trocar size.

**Results**: 11 cases (1.3%) were complicated by ABS. Prenatal suspicion of ABS prompted prenatal lysis without amputation in 3/11 cases (27.3%; Table 1). Digital amputation occurred in 3/11 cases (27.3%). There was no difference in maternal characteristics. Significant differences in intertwin weight discordance prior to FLP (12.9% ± 8.7% versus 23.2% ± 13%, *p* < 0.05), GA at FLP (17.9 ± 1.1 weeks versus 20.6 ± 2.8 weeks, *p* < 0.05), CAS (63.6% (7/11) versus 10.6% (85/805), *p* < 0.05), and trocar size (*p* = 0.04) were demonstrated between cases with and without ABS (Table 2). Amnioinfusion (aOR 0.17, 95% CI 0.02‐0.76, *p* = 0.04), smaller intertwin weight discordance (aOR 0.90, 95% 0.83‐0.96), and earlier GA at FLP (aOR 0.56, 95% CI 0.34‐0.82) decreased the risk of ABS. Conversely, CAS increased the risk of ABS (aOR 11.90, 95% CI 3.15‐52.30, *p* < 0.05).

**Conclusion**: ABS is a rare complication post‐FLP for TTTS, yet, is associated with significant neonatal sequelae. Perioperative factors, particularly CAS, should prompt detailed post‐FLP evaluation for development of ABS.

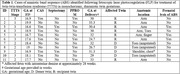

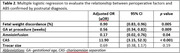

## 761 | Risk Factors for Delivery Less Than 34 Weeks’ Gestation Following Fetoscopic Spina Bifida Repair

Felicia V. LeMoine^1^; Neha Agarwal^2^; Sami Backley^1^; Elisa Garcia^1^; Stephanie Conaway^1^; Ashley Salazar^3^; Lovepreet Mann^1^; Sen Zhu^4^; Stephen A. Fletcher^5^; KuoJen Tsao^6^; Mary T. Austin^7^; Eric P. Bergh^8^; Jimmy Espinoza^1^; Anthony Johnson^9^; Jerrie Refuerzo^10^; Suzanne Lopez^11^; David I. Sandberg^5^; Deijan Lai^1^; Ramesha Papanna^10^

*^1^Division of Fetal Intervention, Department of Obstetrics, Gynecology and Reproductive Sciences, McGovern Medical School at UTHealth Houston, Houston, TX; ^2^Division of Fetal Intervention, Department of Obstetrics, Gynecology and Reproductive Sciences, UTHealth McGovern Medical School, Houston, TX, USA, Houston, TX; ^3^Division of Fetal Intervention, Department of Obstetrics, Gynecology and Reproductive Sciences, McGovern Medical School at UT Health Houston, Houston, TX; ^4^McGovern Medical School at the University of Texas, Houston, TX; ^5^Division of Neurosurgery, Department of Pediatric Surgery, McGovern Medical School at UTHealth Houston, Houston, TX; ^6^McGovern Medical School at the University of Texas Health Science Center at Houston (UTHealth) University of Texas Health Science Center, Houston, TX; ^7^Department of Pediatric Surgery, McGovern Medical School at UT Health Houston, Houston, TX; ^8^UT Health, Houston, TX; ^9^McGovern Medical School University of Texas Health Science Center at Houston (UThealth), Houston, TX; ^10^McGovern Medical School at UTHealth Houston, Houston, TX; ^11^McGovern Medical School. UTHealth Houston, Houston, TX*

10:30 AM ‐ 12:30 PM

**Objective**: Preterm delivery (PTD) is a persistent complication of in‐utero spina bifida repair despite the transition to a minimally‐invasive, fetoscopic approach. We aimed to evaluate the relationship between maternal and perioperative characteristics and PTD < 34 weeks gestation among pregnancies that underwent fetoscopic spina bifida repair.

**Study Design**: A secondary analysis of a prospective cohort study was conducted of 79 completed cases of fetoscopic spina bifida repair between 2020 and 2024 [NCT#04243889 & #06042140]. Maternal and perioperative factors were compared between cases with delivery at < 34 and ≥ 34 weeks’ gestation using the Wilcoxon rank sum test, Welch's *t*‐test, χ^2^ test, and Fisher's exact test, where applicable. Multiple logistic regression was performed to adjust for maternal and perioperative factors. Sub‐group univariate analysis of maternal and perioperative factors between cases with and without placental abruption (PA) were compared using the appropriate comparative statistics.

**Results**: Twenty‐five (31.6%) cases resulted in delivery at < 34 weeks. Maternal age was greater in cases that resulted in delivery < 34 weeks (Table 1). Maternal and perioperative characteristics were, otherwise, similar between the groups. PPROM and placental abruption occurred more frequently among cases with delivery < 34 weeks (Table 1). Multiple logistic regression demonstrated that event of delivering at < 34 weeks was significantly associated with lesion surface area, PPROM, and placental abruption (Table 2). Sub‐group univariate analysis comparing cases with and without PA demonstrated only a smaller lateral ventricular size among cases with PA (10.3 ± 2.8mm) versus cases without PA (12.76 ± 3.8mm, *p* = 0.035).

**Conclusion**: PPROM and placental abruption are significant predictors of PTD before 34 weeks. Further research is needed to elucidate the relationship between PPROM and placental abruption in the context of fetoscopic intervention.

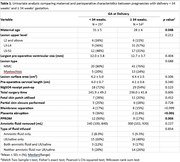

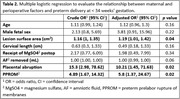

## 762 | TTTS on Tiktok: News Or Noise?

Gabriela F. Tessler; Noelle Breslin; Russell S. Miller

*Columbia University Medical Center, New York, NY*

10:30 AM ‐ 12:30 PM

**Objective**: Patients are increasingly looking to TikTok for health information. We evaluated content and quality of TikTok videos related to twin‐twin transfusion syndrome (TTTS) and fetoscopic laser surgery (FLS).

**Study Design**: The top 50 TikTok videos (TikToks) related to TTTS were identified using search terms “TTTS”, “twin‐twin transfusion”, and “fetal laser surgery”. Data were gathered on video metrics. Three independent reviewers evaluated videos using three standardized quality scales: a modified 5‐point DISCERN scale, the Patient Education Maternal Assessment Tool for Audiovisual Materials (PEMAT A/V), and a 5‐point Likert Global Quality Scale (GQS). TikToks were also graded on creator type, content, tone, and outcomes. Exclusion criteria included non‐English, unrelated, or duplicate videos.

**Results**: 50 TikToks created by 42 users were evaluated. Total views for all TikToks were 63.3 million. The most viewed TikTok had 2.5 million views, the most liked had 537,000 likes, and the most shared had 22,100 shares. 66% of content was created by patients and 14% was created by medical professionals. 26% of videos contained educational content. 34% of videos reported a good fetal outcome and 8% noted a complication or poor outcome. The mean mDISCERN score was 0.8 (SD 0.7). The mean PEMAT A/V understandability score was 56.3% (SD 28.9%), and the mean actionability score was 2.4% (SD 7.5%). The median GQS was 1 [IQR 1‐ 1.6]. TikToks posted by medical professionals had higher quality scores (p< 0.001).  Of the 10 videos with highest GQS, 6 were posted by medical professionals. Of the 10 most‐liked videos, 3 were posted by medical professionals.

**Conclusion**: TikToks related to TTTS and FLS are highly viewed, liked, and shared, yet of overall poor quality. A majority of content was posted by patients and related to individual pregnancy experiences, and was more likely to detail good outcomes than poor outcomes. Medical professionals posted higher‐quality content, much of which was highly viewed and liked. These data support a need and demand for more high‐quality content related to TTTS and FLS on TikTok.

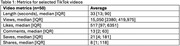

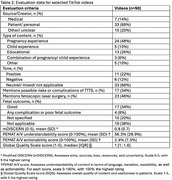

## 763 | Implementation of a New Rolling Circle Replication Assay for Universal Non‐Invasive Prenatal Screening

Gayathri D. Vadlamudi^1^; Kristen Warncke^2^; Jessica E. Pruszynski^3^; Patricia Santiago‐Munoz^1^; Elaine L. Duryea^2^

*^1^UT Southwestern Medical Center, Dallas, TX; ^2^University of Texas Southwestern Medical Center, Dallas, TX; ^3^University of Texas Southwestern, Dallas, TX*

10:30 AM ‐ 12:30 PM

**Objective**: To evaluate the uptake and performance of non‐invasive prenatal aneuploidy screening (NIPS) using a new rolling circle replication assay.

**Study Design**: We conducted a prospective observational study of patients receiving prenatal care at a large county hospital. Patients with singleton pregnancies ≥ 10 weeks gestation were offered NIPS for trisomy 13, 18, and 21 using a non‐sequencing based rolling circle replication assay (Vanadis system, PerkinElmer, Waltham, MA, USA) with analysis performed on‐site. Individuals with abnormal screening results were offered diagnostic testing. We recorded results of invasive diagnostic testing and postnatal genetic testing if performed. Samples with diagnostic confirmation available were used to calculate predictive values.

**Results**: Among 14,127 patients who received prenatal care at our institution between August 2023 to July 2024, 11,631 (82.3%) elected NIPS. The mean BMI in this population was 29.5 ± 6.6 kg/m^2^. There were 106 (0.9%) positive screening results, of which 46 had increased risk for trisomy 21, 32 for trisomy 18, and 28 for trisomy 13. There were 18 no‐call results, with a no‐call rate of 0.15%.  Of patients with diagnostic results available, abnormal screening results were confirmed in 16/19 (84.2%) patients who screened positive for T21, in 11/15 (73.3%) patients who screened positive for T18, and in 2/8 (25.0%) patients screened positive for T13. There were no false negative screens identified for any of these three aneuploidies.

**Conclusion**: Non‐invasive prenatal screening using a rolling circle replication assay offers high sensitivity and specificity for aneuploidy screening with a low rate of no‐call results. Positive predictive values for trisomy 21 and trisomy 18 far exceeded that for trisomy 13 screening.

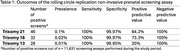

## 764 | Assessing Prioritized Groups Referenced in 2019‐2024 Maternal Mortality Review Committees’ Summaries

Genevieve Kitts^1^; John J. Byrne^2^; Brenda Berumen‐Flucker^3^

*^1^Texas State University, Texas State University, TX; ^2^University of Texas Health San Antonio, San Antonio, TX; ^3^Texas State University, Houston, TX*

10:30 AM ‐ 12:30 PM

**Objective**: Maternal mortality review committees are encouraged to define populations at risk of maternal morbidity and mortality. Summaries should work to include the most recent data among racial/ethnic, socioeconomic, rural, and LGBTQIA+ groups. We investigated states’ most recent summaries to identify which disproportionately affected groups they prioritized.

**Study Design**: We utilized publicly available data from maternal mortality review committees or similar organizations’ summaries online in June 2024. Using Prisma 2020, we established inclusion and exclusion criteria. We compared their priority populations to the CDC's 2022 data set for maternal mortality. We evaluated the utilization of inclusive and gender‐neutral language for LGBTQIA+ individuals. Commonly used phrases within the summaries were birthing people, persons, or individuals.

**Results**: Of the 48 summaries that met inclusion criteria, 91.7% included an excerpt of their priority population based on race or ethnicity. Black, Hispanic, and Native American groups were the most commonly reported minority groups prioritized. Recommendations for diversity training or education were included for 72.9% of reviews, 25.0% did not, and 2.0% were unclear. Evaluating use of inclusive language, 58.3% of summaries explicitly stated intentions to include sexual minority groups using gender‐neutral language; 22.9% were unclear but used terms birthing people or individuals.

**Conclusion**: As of 2024, there continues to be a lack of focus on high‐risk groups within the MMRC reports. This study supports the need for health practitioners to focus on improving inclusive language within MMRC reports. Without acknowledging the groups most in need, providers and community health workers will fail to address maternal morbidity and mortality adequately.

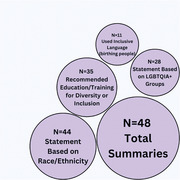

## 765 | Maternal History of Recurrent Pregnancy Loss and the Risk for Offspring Inflammatory Bowel Disease

Gil Gutvirtz^1^; Gali Pariente^2^; Tamar Wainstock^2^; Eyal Sheiner^2^

*^1^Soroka University Medical Center, Metar, HaDarom; ^2^Department of Obstetrics and Gynecology, Soroka University Medical Center, Ben‐Gurion University of the Negev, Beer‐Sheva, Israel., Beer Sheva, HaDarom*

10:30 AM ‐ 12:30 PM

**Objective**: Women with a history of recurrent pregnancy loss (RPL) may harbor a genetic disposition for inflammatory responses causing pregnancy losses. As altered immune response has been implicated in the pathogenesis of inflammatory bowel disease (IBD), we sought to investigate whether offspring of women with RPL are at an increased risk to develop IBD.

**Study Design**: A population‐based cohort study of singleton deliveries was conducted to evaluate the risk for IBD in children (up to the age of 18 years) born to mothers with and without a history of RPL. Data for the diagnosis of IBD of the offspring were extracted from community‐based clinics and hospitalization records. Kaplan‐Meier survival curve was used to compare the cumulative incidence of IBD between the study groups. A cox proportional hazards model was used to control for confounders.

**Results**: During the study period (1991‐2021), 356,356 singleton deliveries were included in the study, of them, 14,235 (4%) deliveries were in women with a history of RPL. Comparable rates of IBD were documented in children born to mothers with and without a history of RPL (0.1% vs. 0.1%, *p* = 0.104, **Figure**). The Kaplan‐Meier survival curve demonstrated a comparable incidence of IBD in offspring of both groups (Log Rank, *p* = 0.919, **Figure**). Likewise, using a Cox proportional hazards model, adjusted for maternal age and gestational age at birth, maternal history of RPL was not associated with IBD of the offspring (adjusted hazard ratio (HR) = 1.11, 95%CI 0.60‐2.05, *p* = 0.731).

**Conclusion**: Maternal history of recurrent pregnancy loss is not a risk factor for IBD in the offspring.

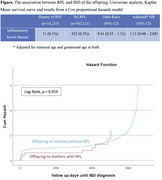

## 766 | Maternal Age at First Delivery and Long‐term Ophthalmic Morbidity of the Mother

Gil Gutvirtz^1^; Eyal Sheiner^2^; Tamar Wainstock^2^; Ruslan Sergienko^3^; Erez Tsumi^4^; Roy Kessous^4^

*^1^Soroka University Medical Center, Metar, HaDarom; ^2^Department of Obstetrics and Gynecology, Soroka University Medical Center, Ben‐Gurion University of the Negev, Beer‐Sheva, Israel., Beer Sheva, HaDarom; ^3^Ben‐Gurion University of the Negev, Beer Sheva, HaDarom; ^4^Soroka, Beer‐Sheva, HaDarom*

10:30 AM ‐ 12:30 PM

**Objective**: Advanced maternal age has been associated with adverse pregnancy outcomes. Complications such as hypertensive disorders, preterm delivery and low birthweight suggest that maternal age is a crucial determinant of vascular adaptation to pregnancy. Few studies have examined these associations with mothers long‐term outcome. We investigated the impact of maternal age at first delivery on long‐term ophthalmic morbidity

**Study Design**: A population‐based retrospective cohort study of women who delivered between the years 1991‐2021 at a tertiary center was conducted. Maternal age at first delivery was divided into 4 categories (< 30 years, between 30‐35, 35‐40 and >40 at first delivery). The long‐term ophthalmic morbidity was compared based on data from both community and hospitalization records involving an ophthalmic morbidity. A Kaplan–Meier survival curve was used to compare the cumulative incidence of ophthalmic morbidity and a Cox regression model was constructed to control for confounders, comparing the groups of maternal age over 30 to a reference group of women under 30

**Results**: A total of 76,131 women were included in the study. 69,530 (91.3%) were under 30 years in their first delivery, 5,066 (6.7%) were between 30‐35, 1,231 (1.6%) were between 35‐40 and 304 (0.4%) were over 40 years old. The cumulative incidence of ophthalmic morbidity over time was higher as maternal age was older in the first delivery (**Figure**). A trend of increased ophthalmic morbidity was noted as maternal age was older but it did not reach a statistical significance (3.7%, 3.6%, 4.1% and 6.3%, *p* = 0.08). The Cox regression model, controlling for fertility treatments and ethnicity, found a dose dependent effect as maternal age in the first delivery was an independent risk factor for long‐term ophthalmic morbidity of the mother as compared with women under 30 years old (**Table**)

**Conclusion**: Advanced maternal age at first delivery is an independent risk factor for long‐term ophthalmic morbidity of the mother. The risk tends to increase in a dose‐response manner over age 30, and specifically for patients over 40 in their first delivery

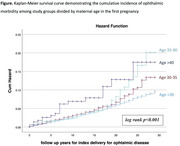

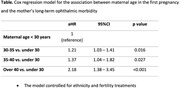

## 767 | Normotension is not Predictive of Duration of Expectant Management of Preterm Preeclampsia with Severe Features

Gillian Piltch^1^; Burton Rochelson^2^; Matthew J. Blitz^2^; Evelina Grayver^2^; Alejandro D. Alvarez^2^; Caroline Pessel^2^

*^1^Northwell, Astoria, NY; ^2^Northwell, New Hyde Park, NY*

10:30 AM ‐ 12:30 PM

**Objective**: To identify the optimal blood pressure range during expectant management of preterm preeclampsia with severe features.

**Study Design**: Multicenter retrospective cohort study of pregnant people with preterm (< 34 weeks gestation) preeclampsia with severe features by severe hypertension criteria who underwent a period of expectant management >48 hours from 2017‐2022. People were excluded if they had chronic hypertension, fetal anomaly, or underwent pregnancy termination. Primary outcomes were completed percentage of maximum potential duration of expectant management until goal of 34 weeks and attainment of 34 weeks gestation. ANOVA, Kruskal‐Wallis test, t‐test, Wilcoxon rank‐sum test, and multivariate regression were performed with p< 0.05 considered statistically significant.

**Results**: 186 people met inclusion criteria. Antepartum blood pressures were normal (< 140/90) 0‐33% of measurements in 36 people (19.4%), 34‐66% in 94 people (50.5%), and 67‐100% in 56 people (30.1%). Percentage of normal blood pressures was not associated with completed percentage of maximum potential duration of expectant management (Figure 1), but increasing percentage of blood pressures in the severe range was associated with lower completed percentage of maximum potential duration of expectant management (Figure 2). Multivariate analysis demonstrated that percentage of blood pressures in the severe range (OR 0.91, CI 0.86‐0.96) and diagnosis of fetal growth restriction (OR 0.41, CI 0.23‐0.74) were associated with a lower completed percentage of maximum potential duration of expectant management while maintenance antihypertensive medication (OR 2.45, CI 1.06‐5.67) was associated with a higher completed percentage. Neither the percentage of normal blood pressures (p = 0.7) nor the percentage of blood pressures in the severe range (p = 0.9) were associated with attaining 34 weeks gestation.

**Conclusion**: Percentage of normal as opposed to elevated blood pressures was not predictive of the duration of expectant management of preterm preeclampsia with severe features.

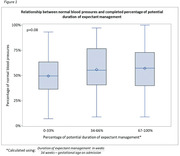

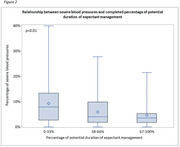

## 768 | Neonatal outcomes following rescue Antenatal Corticosteroids given after 34 weeks of gestation

Hadel Watad^1^; Keren Ofir^2^; Michal Fishel Bartal^3^; Rakefet Yoeli‐Ullman^2^; Shali Mazaki‐Tovi^4^

*^1^Sheba Medical Center, Jatt Village, HaZafon; ^2^Sheba Medical Center, Ramat Gan, HaMerkaz; ^3^UTH Houston & Sheba Medical Center Israel, Houston, TX; ^4^Department of Obstetrics and Gynecology, Sheba Medical Center, Tel HaShomer, Ramat Gan, HaMerkaz*

10:30 AM ‐ 12:30 PM

**Objective**: We aimed to determine neonatal outcomes following administration of rescue antenatal corticosteroid (ACS) at late preterm (>34 weeks) after completion of initial cycle of ACS during early preterm period.

**Study Design**: A retrospective cohort study including all pregnant individuals who delivered singleton late preterm infants and received first dose of ACS before 34 weeks of gestation. Individuals were divided to two groups:1. study group: Received rescue ACS >34 weeks and 2. control group: no rescue ACS after 34 weeks. Data were collected from medical records. Parametric and non‐parametric statistical methods were used for analysis.

**Results**: A total of 757 pregnant individuals met the inclusion criteria. Among them, 21.3% (n = 161) received rescue ACS after 34 weeks of gestation, while 78.7 % (n = 596) did not. Individuals who received rescue ACS had a higher median maternal age, gravidity and parity and received first course earlier during pregnancy compared to individuals without rescue dose (table 1). Neonates born to individuals who received rescue ACS had a significantly lower rates of composite neonatal outcomes compared to those who did not receive ACS (27.6% vs. 39.9%, p = 0.05), lower rates of NICU admission (26.9% vs. 39.7%, P = 0.003), and higher rates of neonatal fever (3.2% vs. 0.7%, P = 0.027). However, rates of RDS (1.3% vs. 3.2 %, P = 0.274) and composite respiratory adverse outcomes (10.3% vs. 13.8%, P = 0.298) were comparable between the groups. Additionally, rates of neonatal hypoglycemia were comparable (11.8% vs. 17.4%, p = 0.085 respectively). Following logistic regression and adjustment for gestational age at first ACS course, maternal age, and gravidity; those not receiving rescue ACS at late preterm had a higher rate of adverse composite neonatal outcomes [aOR 1.13, 95% CI 1.044‐1.186].

**Conclusion**: Rescue ACS administration in late preterm was associated with reduced composite neonatal outcomes and NICU admission. However, it was not associated with a reduced rates of RDS or composite respiratory outcomes. Importantly, there was no significant difference in the prevalence of hypoglycemia.

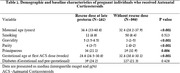

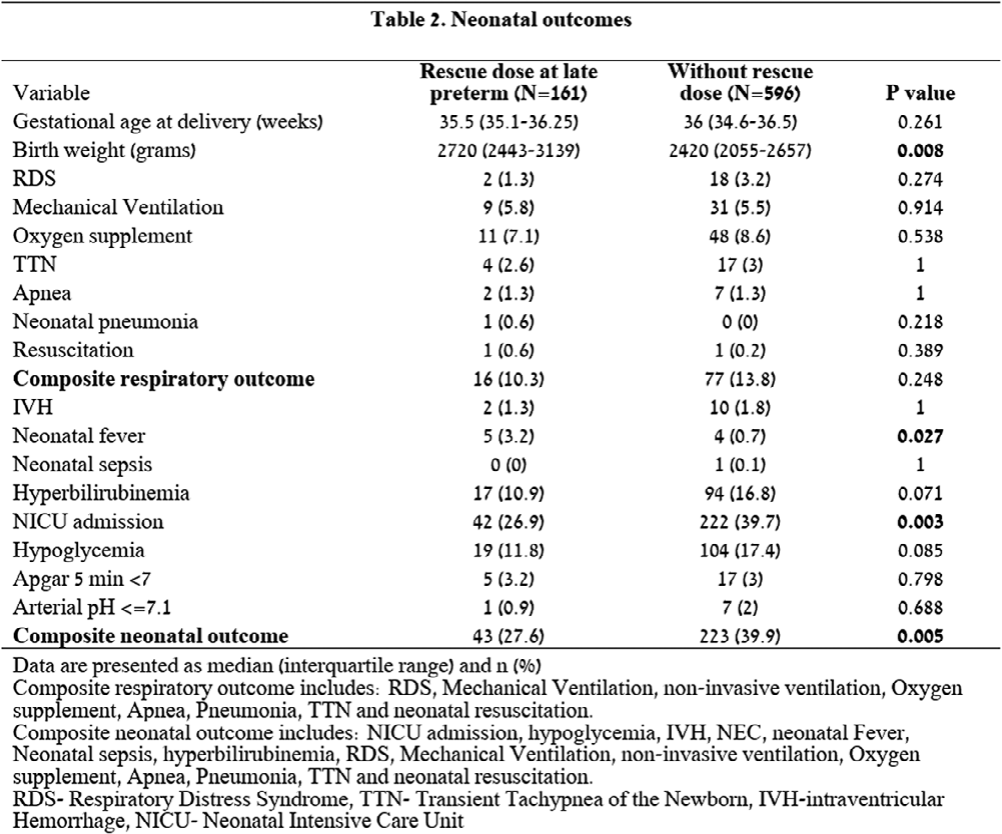

## 769 | Did Increasing Postpartum Oxytocin Infusion to 30 Units Decrease Postpartum Hemorrhage(PPH) at a University Hospital?

Hadley K. Ross^1^; Alixandria F. Pfeiffer^2^; Camille B. Marquez^3^; Nora M. Doyle^4^; Michael Gardner^5^

*^1^Univeristy of Texas Health San Antonio, San Antonio, TX; ^2^University of Texas Health Science Center at San Antonio, San Antonio, TX; ^3^University Health System, San Antonio, TX; ^4^University of Texas Health San Antonio, San Antonio, TX; ^5^University Health Women and Childrens Hospital, San Antonio, TX*

10:30 AM ‐ 12:30 PM

**Objective**: Postpartum hemorrhage is a common occurrence affecting approximately 5% of all pregnancies and 11% of all pregnancy associated mortalities in the U.S. In January of 2024 we changed our institutional protocol to increase the routine use of Oxytocin from 10 units to 30 units immediately after delivery. The primary aim of our study was to see if this protocol decreased PPH at a university hospital. Our secondary aim was to determine if PPH was associated with population characteristics such as BMI.

**Study Design**: We performed a retrospective cohort study of PPH before and after the implementation of the new Oxytocin protocol on 1/16/2024. We initially implemented the change in vaginal deliveries only, but on 5/22/2024 we instituted the higher oxytocin dose at all deliveries. Secondary analysis involved patient demographics such as BMI.

**Results**: The patient demographics were similar between groups with high rates of obesity, cesarean sections, multiparity, and hypertension. The rate of PPH was the same at 14% both before and after the increase in oxytocin postpartum.

**Conclusion**: In an attempt to decrease the high rate of PPH at our institution we implemented a protocol to increase the postpartum dose of Oxytocin to 30 units. We did not find any change in the rate of PPH. Of note, our rate of PPH of 14% both before and after the change is higher than the national reported average of 5%. This may be because of our tertiary care center, our high rate of obesity, or that we routinely use quantitative measurement of postpartum blood loss. Further interventions are needed to address the high rate of PPH seen at our university hospital.

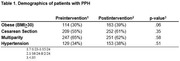

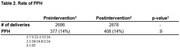

## 770 | The Efficacy of Deep Learning Based Automated Cervical Length Measurement for Predicting Preterm Birth

Hayan Kwon; Ju‐hee yoon; Yun Ji Jung; Ja‐Young Kwon; Suhra Kim

*Yonsei University College of Medicine, Seoul, Seoul‐t'ukpyolsi*

10:30 AM ‐ 12:30 PM

**Objective**: Cervical length measurement of less than 2.5 cm in pregnant women between 16 weeks 0 days and 24 weeks 6 days is commonly used to predict preterm labor. However, conventional measurements are highly operator‐dependent and often underestimate actual cervical length, which can lead to overtreatment. This study aimed to compare conventional cervical length measurement with deep learning‐based automated measurement to determine any differences in preterm prediction.

**Study Design**: The study included 197 women out of 1270 who received antenatal care and delivery at Severance Hospital from 2019 to 2023. Participants had cervical lengths measured conventionally as 2.5 cm or less within the specified gestational period. Those with preterm labor, multiple pregnancies, or who underwent cerclage were excluded. Cervical length was measured using traditional and deep learning methods. Deep learning program utilized a semantic image decomposition network to divide images into pre‐cervix, cervix, and post‐cervix regions for cervical canal identification. The canal was assessed at six points for distance calculation. Participants were divided into Group A (cervical length of 2.5 cm or less by both methods) and Group B (less than 2.5 cm by conventional but more than 2.5 cm by deep learning). The primary endpoint was preterm birth, defined as delivery before 37 weeks.

**Results**: Group A had an average cervical length of 2.32 cm (+‐0.159), while Group B's average was 2.73 cm (+‐0.317). The preterm birth rate was 16.2% (9/152) in Group A and 4.8% (21/197) in Group B. Group B had a significantly lower preterm birth rate (P < 0.05), suggesting that cervical tracing provides longer cervical length measurements, improving preterm birth prediction accuracy.

**Conclusion**: Cervical length measurement through tracing offers more accurate preterm birth predictions than conventional methods. Deep learning for CL measurement can also reduce patient discomfort, shorten examination times, and provide operator independence. Integrating deep learning AI for cervical length measurement has important implications for future practices.

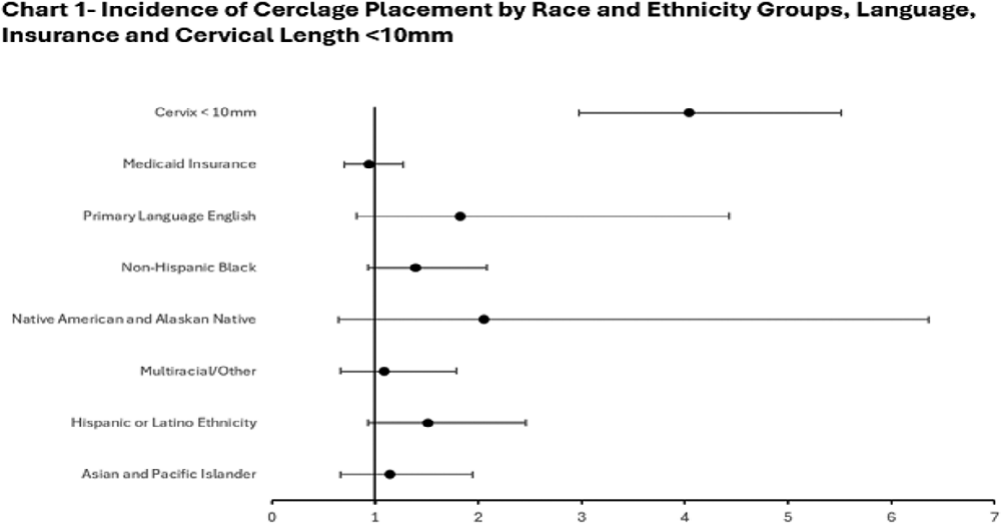

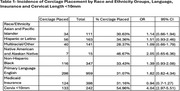

## 771 | Outcomes in Vaginal versus Cesarean Periviable Breech Delivery: A 5‐year, Propensity Score–Matched Study

Helen B. Gomez Slagle^1^; Yongmei Huang^2^; Cande V. Ananth^3^; Uma M. Reddy^4^; Alexander M. Friedman^1^

*^1^Columbia University Irving Medical Center, New York, NY; ^2^Department of Obstetrics and Gynecology, Columbia University Irving Medical Center, New York, NY; ^3^Rutgers Robert Wood Johnson Medical School, New Brunswick, NJ; ^4^Columbia University, New York, NY*

10:30 AM ‐ 12:30 PM

**Objective**: To determine maternal and neonatal outcomes associated with vaginal versus cesarean breech delivery at periviability.

**Study Design**: This study analyzed non‐anomalous, singleton, breech live births at 22w0d to 25w6d gestational age identified in the CDC Linked Birth/Infant Death Records data from 2016 to 2021. Multiple imputation was used to account for missing data. A propensity score (PS) analysis was conducted to establish pseudo‐randomization based on the mode of delivery matching vaginal to cesarean deliveries at a ratio of 1:2 using greedy nearest‐neighbor matching. The balance diagnostic was measured using standardized mean difference. In the outcome model, we estimated the risk difference (RD) and risk ratios (RR) and associated 95% CIs based on generalized estimation equation framework, using log‐linear models with binomial distribution and a log/ identity link, respectively, after re‐adjusting for gestational age (GA).

**Results**: Of 21,461 periviable breech singleton births, 34% (N = 7,289) delivered vaginally. Vaginal delivery was more common in earlier GA. After PS matching, the distributions of baseline factors were balanced between the delivery arms. A composite of adverse neonatal outcomes occurred among 99.0% (N = 7,213) of vaginal and 96.8% (N = 13,716) of cesarean breech births (RD 1.85, 95% CI ‐1.77 to 5.46; RR 1.02, 95% CI 0.98 to 1.06). Neonatal mortality was significantly higher for vaginal than cesarean breech births (73% versus 36%; RD 28.29 95% CI 25.50 to 31.07; RR 1.70, 95% CI 1.61 to 1.79). A composite of adverse maternal outcomes occurred in 1.6% of vaginal breech and 3.1% of cesarean births (RD ‐1.58, 95% CI ‐2.20 to ‐0.96, RR 0.48, 95% CI 0.36 to 0.65).

**Conclusion**: Vaginal breech birth between 22w0d and 25w6d gestation is associated with lower risk of adverse maternal outcomes but higher risk of neonatal mortality.

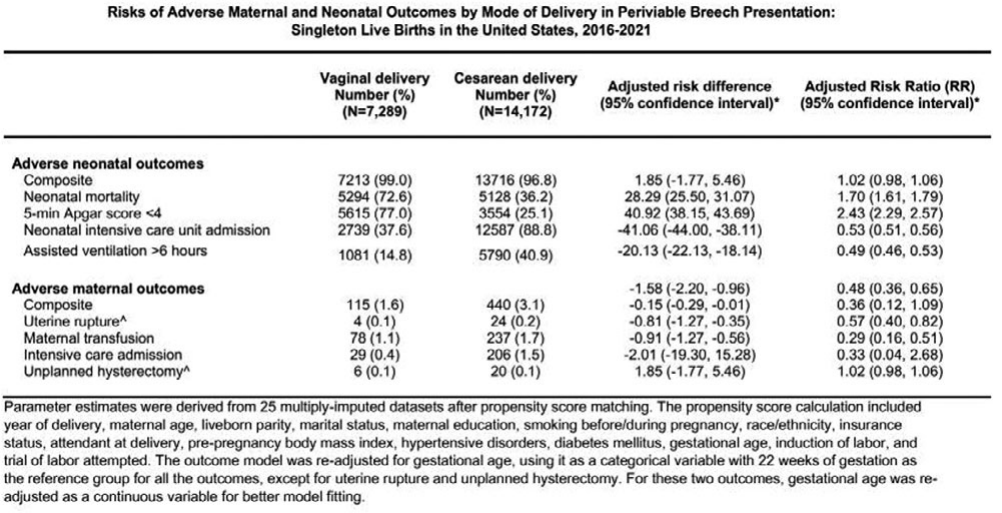

## 772 | Gestational Diabetes Mellitus in the 3RD Trimester After Diagnosing Large for Gestational Age And/Or Polyhydramnios

Henry Lesser; A. Dhanya Mackeen; David Chromey, II; Amanda J. Young; Celia Gray; Michael J. Paglia

*Geisinger Medical Center, Danville, PA*

10:30 AM ‐ 12:30 PM

**Objective**: To evaluate outcomes of patients who rescreen for gestational diabetes mellitus (GDM) in the 3rd trimester based on a diagnosis of large for gestational age (LGA) and/or polyhydramnios.

**Study Design**: This is a retrospective cohort study from 1/1/11‐3/1/24 of term, singleton pregnancies who were rescreened for GDM after being diagnosed with LGA and/or polyhydramnios. Patients with fetal growth restriction, or fetal anomalies were excluded. We compared outcomes in those that passed and failed rescreening using a logistic regression model where birthweight was adjusted for gestational age. Odds ratios and respective 95% confidence intervals and p‐values are reported.

**Results**: We identified 348 pregnancies that completed GDM rescreening: 48 (13.8%) were diagnosed with GDM of which 10 (20.8%) required medication (A2GDM). When comparing patients that passed vs. failed rescreening, there were no statistically significant differences for birthweight (BW), BW ≥4000g, shoulder dystocia, preeclampsia or cesarean delivery. Patients that failed rescreen were more likely to have a newborn with neonatal hypoglycemia (7.0% vs. 22.9%, p< 0.01) and develop GDM in future pregnancies (1.3% vs. 10.4%, < 0.01).

**Conclusion**: The rate of diagnosis of GDM in the 3rd trimester after rescreening is low at only 14%, and similarly the rate of A2GDM is exceedingly low at 3% of all patients that were rescreened. Except for neonatal hypoglycemia, outcomes for patients diagnosed with GDM did not differ from those that passed rescreening. Despite making the diagnosis of GDM, there does not appear to be utility in rescreening due to the lack of improvement in perinatal outcomes.

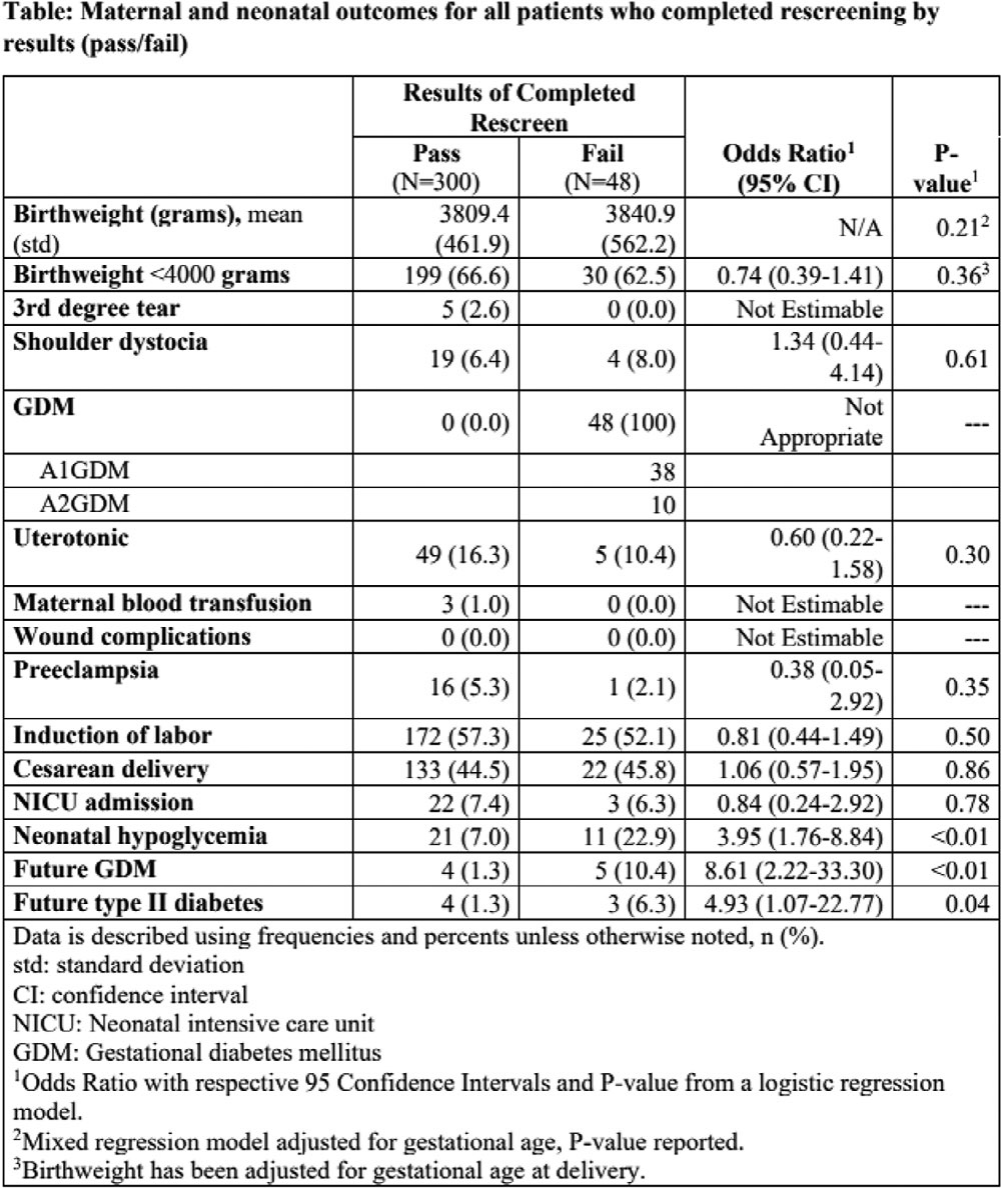

## 773 | Clinical Utility of Various Growth Factors in Preeclampsia

Ho Yeon Kim^1^; Gwan Heup Song^2^

*^1^Korea University School of Medicine, Danwongu AnsanSi, Kyonggi‐do; ^2^Korea University School of Medicine, Danwongu/Ansan City, Kyonggi‐do*

10:30 AM ‐ 12:30 PM

**Objective**: This study aimed to compare the levels of various growth factors in maternal serum and umbilical vein serum between individuals with preeclampsia (PE) and those with normotensive pregnancies.

**Study Design**: One hundred eight pregnant women were enrolled in this prospective study. 67 serum samples of preeclampsia and 41 normotensive mothers were collected. Umbilical vein serum samples of 27 PE and 38 normotensive pregnancies were collected. Serum levels of BDNF, EGF, FGF‐2, HGF, LIF, NGF‐beta, PDGF‐BB, PlGF‐1, SCF, and VEGF‐A and D were measured.

**Results**: EGF, HGF, LIF, PDGF‐BB and SCF levels were significantly higher in PE compared to normotensive group. After adjustment for gestational age, EGF, HGF, LIF and SCF remained significantly high in PE. There were no differences in growth factors in umbilical vein serum between two groups. EGF, NGF‐beta and SCF were associated with the elevation of systolic and diastolic blood pressure, while EGF was negatively correlated with birthweight and positively with protein/creatinine ratio. SCF showed a positive correlation with ALT and creatinine and a decreasing trend as gestational age advances.

**Conclusion**: Circulating growth factors in PE, especially elevated levels of EGF, HGF, LIF, and SCF, may be responsible for the development or consequences associated with the pathogenesis of PE, with evidence demonstrating an association with severe features. These hormones in placenta appear to have different patterns compared to those in the maternal blood, suggesting that the placenta may regulate these factors to prevent them from reaching the fetus. Further experiments are need to explain this phenomenon.

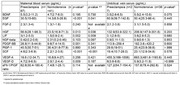

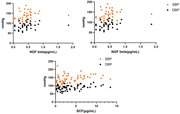

## 774 | Sonographic Features of Placenta Accreta Spectrum with and Without Placenta Previa

Hope Y. Yu^1^; Rishi B. Chopra^2^; Kris Ann Botka^2^; Daniela A. Carusi^2^; Thomas D. Shipp^2^

*^1^Brigham and Women's Hospital / Harvard Medical School, Boston, MA; ^2^Brigham and Women's Hospital, Boston, MA*

10:30 AM ‐ 12:30 PM

**Objective**: To evaluate sonographic features of placenta accreta spectrum (PAS) with placenta previa compared to those without placenta previa.

**Study Design**: Prospective, single referral center study of pregnancies undergoing a standardized, ultrasound protocol for PAS risk assessment in the second and third trimesters. Protocol was applied to pregnancies with placenta previa or request of the obstetric provider based on risk factors. Transvaginal ultrasound was performed if placenta previa was present. Loss of hypoechoic zone (LHZ), retroplacental myometrial thinning (RMT), placental lacunae (PL), placental extension (PE), hypervascularity (HV) and overall PAS risk were assessed. Delivery at >23 weeks gestation from 1/1/2023 to 7/15/2024 with clinical and/or histologic PAS were included. PAS with placenta previa (PAS‐PP) included those with placenta covering the cervical os at the time of delivery. PAS without placenta previa (PAS‐NP) excluded those with low‐lying placenta even if they resolved.

**Results**: Two hundred nineteen patients underwent the PAS ultrasound protocol and 37 diagnosed with PAS, 24 PAS‐PP and 13 PAS‐NP. PAS‐NP was more likely than PAS‐PP to have history of PAS (38% vs 0%, p < 0.01) and less likely to have LHZ, RMT, and HV. There was also a difference in number and size of PL. PAS‐PP was more likely to have sonographic suspicion for PAS (78% vs 33%, p = 0.02) with a PPV and NPV of 76% and 82%, respectively. Among PAS‐NP, PPV and NPV was 44% and 81%, respectively. Twenty‐three cases were non‐invasive, 10 PAS‐PP (42%) and 13 PAS‐NP (100%). Sonographic suspicion for PAS was noted in 60% PAS‐PP and 33% PAS‐NP in the non‐invasive subgroup, however, we were not powered to detect a difference (p = 0.39).

**Conclusion**: Standard ultrasonographic signs of PAS are less likely to be present in PAS without placenta previa resulting in lower detection rates. Larger studies are needed to determine whether these differences persist in non‐invasive PAS. Alternative PAS risk assessment tools should be investigated in non‐previa PAS to improve antenatal detection.

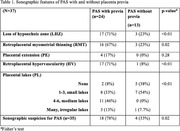

## 775 | Universal Or Selected Policy Of Tranexamic Acid For Preventing Blood Loss After Cesarean Delivery?

Hugo Madar^1^; Thomas Ferté^2^; Antoine Bénard^3^; Kilian Trin^2^; Catherine Deneux‐Tharaux^4^; Loïc Sentilhes^5^

*^1^Bordeaux University Hospital, Bordeaux, Aquitaine; ^2^Public Health Department, Clinical Epidemiology Unit, Bordeaux University Hospital, Bordeaux, France, Public Health Department, Clinical Epidemiology Unit, Bordeaux University Hospital, Aquitaine; ^3^Public Health Department, Clinical Epidemiology Unit, Bordeaux University Hospital, Bordeaux, France, Public Health Department, Clinical Epidemiology Unit, Bordeaux University Hospital, Bordeaux, Aquitaine; ^4^Université Paris Cité, CRESS U1153, Obstetrical, Perinatal and Pediatric Epidemiology Research Team, EPOPé, INSERM, INRAE, Paris, France, Paris, Ile‐de‐France; ^5^Department of Obstetrics and Gynecology, Bordeaux University Hospital, Bordeaux, France, Department of Obstetrics and Gynecology, Bordeaux University Hospital,, Aquitaine*

10:30 AM ‐ 12:30 PM

**Objective**: Prophylactic tranexamic acid has been shown to reduce postpartum blood loss in cesarean deliveries, but its effectiveness may vary based on individual factors and cesarean management. Identifying the women who would benefit most could optimize treatment and minimize unnecessary exposure.

We reanalyzed data from the TRAAP2 trial to determine if the effect of tranexamic acid on postpartum hemorrhage (PPH) varies with women and management of the cesareans’ characteristics.

**Study Design**: We included all women in the modified intention‐to‐treat population of TRAAP2 who received tranexamic acid or placebo. The primary outcome was PPH, defined as blood loss over 1000 ml or a red‐cell transfusion within two days post‐delivery. Predictors for PPH included baseline characteristics and cesarean management details. Both logistic regression with lasso penalization (LRL) and random forest (RF) were used to estimate associations between PPH and predictors. Prediction accuracy was assessed using the area under the ROC curve (AUROC) and the precision‐recall curve (AUPRC).

**Results**: The study included 4,367 women (2,190 in the tranexamic acid group and 2,177 in the placebo group). Lasso logistic regression outperformed random forest, with an AUROC of 0.72 (95% CI: 0.70‐0.74) (Figure 1). The most important predictors of PPH were multiple pregnancy, number of cesareans, body mass index at the end of pregnancy, hemoglobin within 7 days before delivery, gestational age at delivery and cesarean during labor because of protracted labor. Model had poor calibration for benefit of tranexamic acid with an observed benefit almost constant across the quintile of predicted benefit with a discrimination for benefit of 0.04 (95%CI: ‐0.07; 0.15) (Figure 2).

**Conclusion**: Although tranexamic acid is effective for the prevention of calculated blood loss at population level, its individual efficacy is difficult to forecast accurately. This suggests that a targeted prophylactic approach, rather than universal use, may not be appropriate for preventing blood loss in cesarean deliveries.

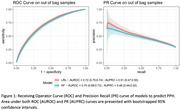

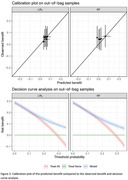

## 776 | Assessment of the Impact of Hemodynamic‐aligned Antihypertensive Therapy in Postpartum Management of Preeclampsia

Ifeoma M. Ogamba‐Alphonso^1^; Erin Miller^2^; Tudi‐Max Brown‐Thomas^2^; Ioanna Katehis^2^; Saige Gitlin^2^; Delphina Maldonado^2^; Gabriella Fernandez De Salvo^2^; Tony Asfour^2^; Keya Shah^2^; Mehak Kapoor^2^; George Gubernikoff^2^; Wendy Kinzler^2^; Martin Chavez^3^; Hye Heo^2^; Patricia Rekawek^2^

*^1^NYU Langone Hospital ‐ Long Island, Island Park, NY; ^2^NYU Langone Hospital, Long Island, NY; ^3^NYU Langone Hospital, New York, NY*

10:30 AM ‐ 12:30 PM

**Objective**: Labetalol and nifedipine are first line antihypertensive agents for hypertensive disorders of pregnancy. There's limited research on selecting medication based on hemodynamic profiles in preeclampsia (PEC), such as high cardiac output (CO) and high systemic vascular resistance (SVR). Our objective was to assess if concordance of antihypertensive treatment with the hemodynamic status on echocardiography (echo) optimizes blood pressure (BP) control in PEC patients.

**Study Design**: This is a retrospective cohort study of patients with PEC with severe features who delivered at our institution and received a postpartum echo, excluding patients with cardiomyopathy. Antihypertensive choice and decision to order echo were provider dependent. CO and SVR were calculated retrospectively from the echo in collaboration with cardiology. Concordance was defined as patients with high CO (>6L/min) initiated on labetalol and high SVR (>1200 dyn.s.cm^5^) initiated on nifedipine. Patients initiated on the opposite medication were grouped as discordant. The primary outcome was time to optimal BP control, defined as the period from the start of BP medication to when no titration was needed. Chi‐squared & Fisher's exact tests were used for categorical variables, and Mann‐Whitney U test for continuous variables.

**Results**: Of the 145 patients, 83 (58%) received concordant therapy. Maternal demographics were similar between groups (Table 1). No patients were on magnesium sulfate during the echo. Most patients had high SVR (74%) vs high CO (26%). Of those with high SVR, 56% were on nifedipine. Overall, 51% of patients received Labetalol and 48% received Nifedipine. There was no difference in the median time to optimal BP control (2 days for both groups, p = 0.4). The median postpartum length of stay was 3 days for both groups (p >0.9) (Table 2).

**Conclusion**: Our study revealed majority of patients with PEC had high SVR, with Nifedipine initiation aligning with most hemodynamic profiles. Although time to optimal BP control did not differ, prospective studies are needed to explore the benefits of hemodynamic aligned therapy in PEC.

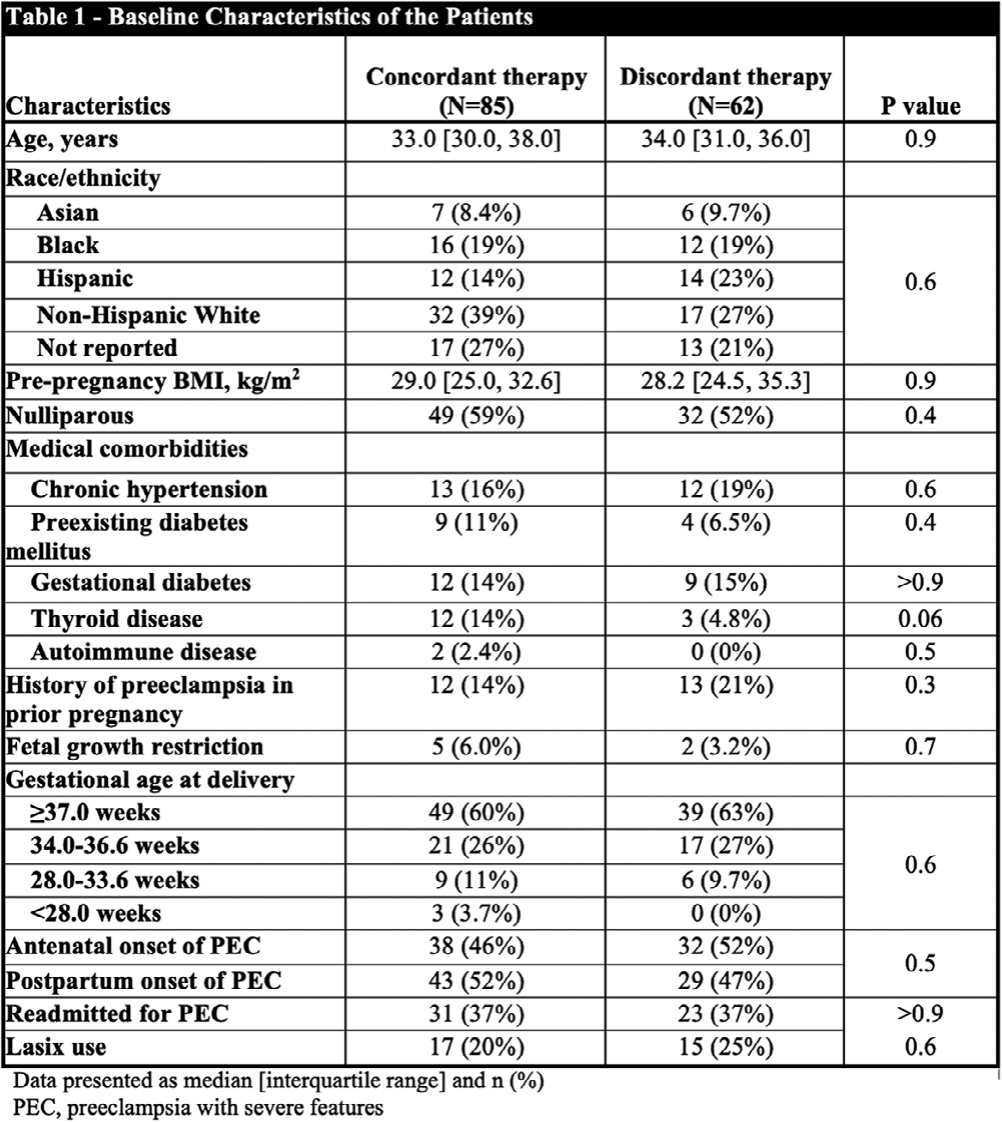

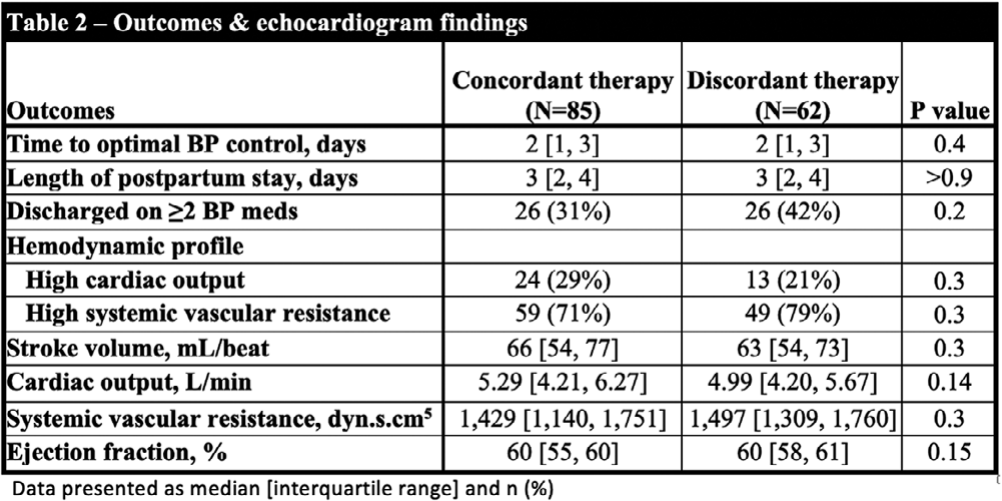

## 777 | Effect of Social Drivers of Health on Screening for Type 2 Diabetes During Delivery Hospitalization

Ingmar N. Bastian; Manisha Gandhi; Mark A. Turrentine

*Baylor College of Medicine, Houston, TX*

10:30 AM ‐ 12:30 PM

**Objective**: To estimate the effect of social and structural drivers of health on completion of screening for type 2 diabetes during the delivery hospitalization in patients with gestational diabetes mellitus (GDM).

**Study Design**: The American College of Obstetricians and Gynecologists updated guidance in May 2024 to offer a 2‐hour oral glucose tolerance test (OGTT) in patients with GDM during the immediate delivery hospitalization. Subsequently, our institution began to offer all patients with GDM this option. Patients with recent (7 days) administration of antenatal corticosteroids and those with out of state follow‐up were excluded. Demographic, social and structural drivers of health were prospectively collected on all patients with GDM. A comparison of variables was performed between those who did versus those who did not complete the OGTT in the immediate delivery hospitalization. P < 0.05 was considered statistically significant.

**Results**: From June 24 through August 1, 2024, 694 deliveries occurred with 40 (5.8%) patients having GDM. 38 patients were offered inpatient OGTT, 1 was not eligible, and 1 was excluded from analysis. 53.8 % (21/39) completed and 46.2% (18/39) did not complete screening (Figure 1). 84.6% (11/13) with medication controlled GDM (A2GDM) and 38.5% (10/26) with diet controlled GDM (A1GDM) completed screening; (OR = 8.8, 95% CI: 1.87‐65.09). Social and structural drivers of health were similar between the two groups (Table 1).

**Conclusion**: A greater proportion of patients with GDM accept screening for type 2 diabetes via the OGTT during delivery hospitalization compared to traditional rates reported at 4 to 12 weeks postpartum (< 30% receive recommended screening with OGTT). Patients with A2GDM were more likely to complete inpatient screening than those with A1GDM. Social and structural drivers of health were similar between groups. Further studies will be needed to identify variables that may increase screening uptake for type 2 diabetes in patients with GDM in the immediate postpartum delivery hospitalization.

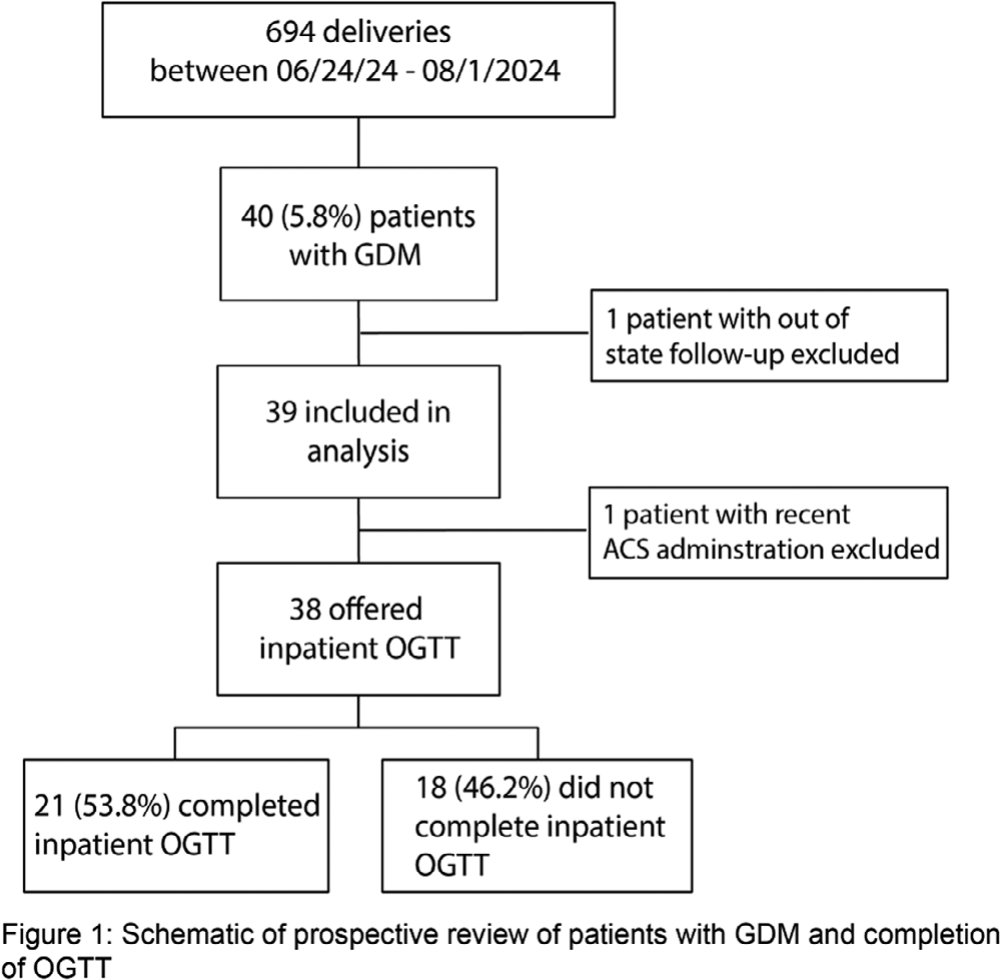

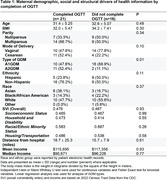

## 778 | Which Cutoff of the 50‐Gram Gct Has a Higher Predictive Value for Gestational Diabetes Mellitus?

Israel Yoles^1^; Tuvia Baevsky^2^

*^1^Clalit Health Services, THe Central District, Beit Gamliel, HaMerkaz; ^2^Clalit Health Services, THe Central District, Rishon Le Tzion, HaMerkaz*

10:30 AM ‐ 12:30 PM

**Objective**: The 50‐gram glucose challenge test (GCT) is used as the initial step in screening for gestational diabetes mellitus (GDM). If the GCT result exceeds the institutional threshold, a 100‐gram oral glucose tolerance test (OGTT) is performed. The objective of this study was to determine which GCT result is high enough to predict a diagnosis of GDM

**Study Design**: The study included all pregnant women who underwent a GCT in in a large Health Maintenance Organization. The study included all pregnant women who underwent a GCT between July 2002 and July 2024. We analyzed various GCT values to determine the percentage of women with at least one abnormal value in the subsequent OGTT. GCT was performed if the value was 140 mg/dL or higher. Normal results for OGTT were defined as follows: fasting < 95 mg/dL, 60 minutes < 180 mg/dL, 120 minutes < 155 mg/dL, and 180 minutes < 140 mg/dL

**Results**: During the study period, 95,562 GCTs were performed, of which 20,797 (21.7%) resulted in a value of 140 mg/dL or higher. Overall, 3,068 women (15%) exhibited at least one abnormal value in the OGTT. The incidence of women with abnormal OGTT results for each GCT group ranged from 24% to 80% (Table 1). The incidence of abnormal OGTT results in each GCT group increased with age, with older women having a higher incidence of these results (Table 2)

**Conclusion**: In this large cohort, even significantly elevated GCT values could be followed by normal OGTT results, especially in young pregnant women. Therefore, it is recommended to perform an OGTT for every GCT result above the institutional cutoff

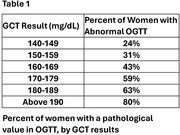

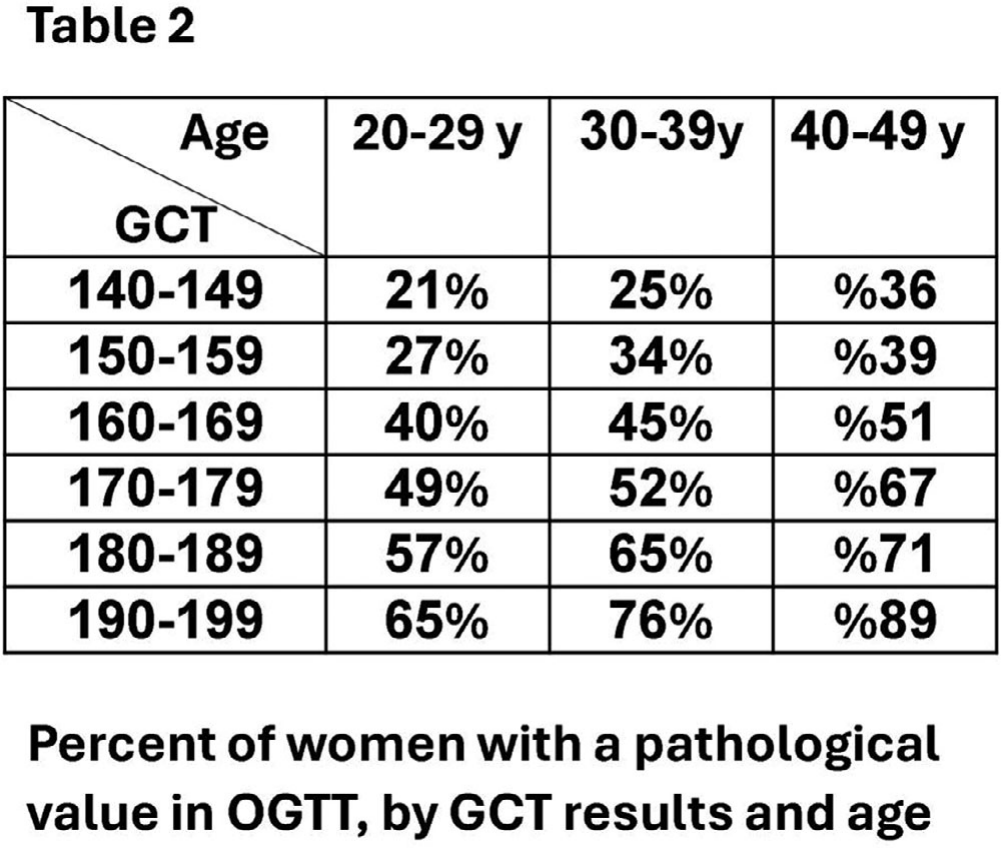

## 779 | Clinical Chorioamnionitis is a Major Risk Factor for Failed Vacuum‐Assisted Deliveries

Itamar Gilboa^1^; Daniel Gabbai^1^; Anat Lavie^2^; Yariv Yogev^3^; Emmanuel Attali^1^

*^1^Lis Hospital for Women's Health, Tel Aviv Sourasky Medical Center, Tel‐Aviv, Tel Aviv; ^2^Tel Aviv Sourasky Medical Center, Tel Aviv Sourasky Medical Center, Tel Aviv; ^3^Lis Maternity Hospital, Sourasky Medical Center, Tel Aviv University, Tel Aviv Sourasky Medical Center, Tel Aviv*

10:30 AM ‐ 12:30 PM

**Objective**: We aimed to determine the impact of  clinical chorioamnionitis during labor and the risk of failed vacuum‐assisted delivery (VAD).

**Study Design**: A retrospective cohort study at a single university‐affiliated tertiary medical center with approximately 12,000 deliveries annually from 2011‐2023. The study group included singleton pregnancies at ≥36 weeks' gestation undergoing a trial of vacuum delivery. The study group, comprising cases of failed vacuum extraction (defined by extraction duration over 20 minutes, more than two cup detachments, or the operator's decision to switch to urgent cesarean delivery), and the control group, consisting of successful vacuum extractions. Clinical chorioamnionitis was diagnosed with an intrapartum fever of ≥38°C and the presence of either a white blood cell count ≥15,000 or documentation of a commonly used broad‐spectrum antibiotic regimen for clinical chorioamnionitis.

**Results**:  

1. Vacuum extraction was attempted in 9,402 out of 111,878 vaginal deliveries (8.4%).

2. The rate of failed VAD was 197/9402 deliveries (2.1%).

3. Multivariate logistic regression analysis identified clinical chorioamnionitis as the major independent risk factor for failed VAD (OR = 3.65, 95% CI 2.3‐5.8, p < 0.001).

4. Additional risk factors for failed VAD included: Occiput posterior position (OR = 2.45, 95% CI 1.6‐3.7, p < 0.001); Fetal head station less than 2 cm below the ischial spine (OR = 1.92, 95% CI 1.4‐2.7, p < 0.001); duration of the second stage of delivery > 3.5 hours (OR = 1.92, 95% CI 1.4‐2.7, p < 0.001); birth weight > 3,500 grams (OR = 1.77, 95% CI 1.3‐2.5, p < 0.001); induction of labor (OR = 1.95, 95% CI 1.4‐2.7, p < 0.001); meconium stained amniotic fluid (OR = 1.52, 95% CI 1.1‐2.2, p = 0.018); Male gender (OR = 1.18, 95% CI 1.0‐2.0, p = 0.042).

**Conclusion**: In addition to well‐known risk factors for failed VAD, clinical chorioamnionitis was found to be the main factor associated with an increased risk of vacuum failure.

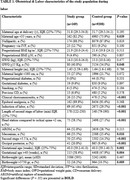

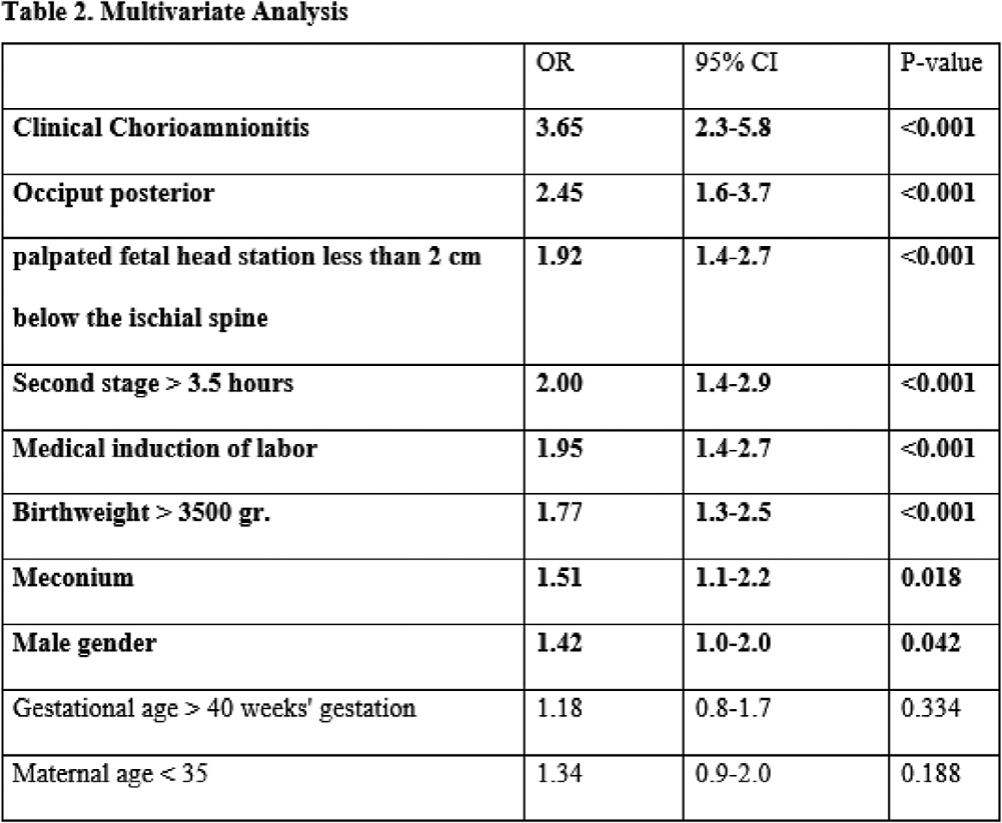

## 780 | is Prenatal Bisphenol and Phthalate Exposure Associated with Fetoplacental Ratio?

Ayushi Bommireddipalli^1^; Jacqueline A. Erler^2^; Whitney Cowell^1^; Kurunthachalam Kannan^3^; Leonardo Trasande^1^; Linda G. Kahn^1^; Shilpi S. Mehta‐Lee^4^

*^1^NYU Grossman School of Medicine, New York, NY; ^2^Rutgers Robert Wood Johnson Medical School, NJ; ^3^NY State Department of Health, Wadsworth Center, Albany, NY; ^4^NYU Langone Health, Brooklyn, NY*

10:30 AM ‐ 12:30 PM

**Objective**: Fetoplacental ratio (FPR), the ratio of birthweight (BW) to placental weight (PW), is a well‐described indicator of placental efficiency that is linked to future health risks. Bisphenols (BPs) and phthalates are endocrine‐disrupting chemicals found in plastics, household products, and vinyl medical tubing. There is no strong evidence that prenatal exposure to BPs or phthalates is associated with neonatal BW, and yet studies show associations with pregnancy complications and adverse child health outcomes. We analyzed prenatal exposure to BPs and phthalates in relation to FPR, BW, and PW as a possible novel explanation.

**Study Design**: 401 participants in the [institution redacted] Children's Health and Environment Study with data on prenatal chemical exposure, BW, and PW from singleton births were included. Chemical concentrations were log‐transformed and adjusted for creatinine. Molar sums of metabolites represented exposure to low and high molecular weight (LMW, HMW) phthalates, diethylhexyl phthalate (DEHP), antiandrogenic phthalates, and BPs; trimester concentrations were averaged. Linear regression models were adjusted for gestational age, fetal sex, maternal demographics, and chemical batch (Table 1). Analyses were stratified by fetal sex.

**Results**: We found no significant associations between chemical exposure and FPR in the combined sample. Among female fetuses, BPs and HMW phthalates were linked with higher FPR, the former association driven by BPS and the latter driven by DEHP. In models with BW and PW as outcomes, we observed no association with BW in combined or stratified models. We found significant negative associations for the same chemical groups and PW among female infants.

**Conclusion**: Among female fetuses, exposures to HMW phthalates and BPs are significantly associated with higher FPR due to reduced PW. The BP findings were driven by exposure to BPS, a recent BPA substitute in many consumer products. Our results suggest that BPs and phthalates may affect placental efficiency, with potential implications for pregnancy complications and child health outcomes.

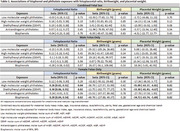

## 781 | a Just Start: a Mixed‐Methods Analysis of Obstetric Health‐Harming Legal Needs Among Low‐Income Pregnant People

Jecca R. Steinberg^1^; Hebron Kelecha^1^; Madeline F. Perry^1^; Nivedita Potapragada^1^; Allie Valenzuela^1^; Erica O'Neill^2^; Ashish Premkumar^3^; Lynn M. Yee^1^

*^1^Northwestern University Feinberg School of Medicine, Chicago, IL; ^2^Stroger Hospital Cook County, Chicago, IL; ^3^Pritzker School of Medicine, University of Chicago, Chicago, IL*

10:30 AM ‐ 12:30 PM

**Objective**: Health‐harming legal needs (HHLN) comprise social barriers to health best addressed through medical‐legal collaboration. The objective of this analysis was to understand patient perspectives and experiences with HHLN in pregnancy and postpartum.

**Study Design**: In this mixed‐methods study, we conducted validated surveys and semi‐structured interviews in English or Spanish with obstetric patients in a public hospital in Chicago, Illinois. Surveys included the Medical‐Legal Partnership Legal Needs Screening Tool and a demographic survey. We applied community‐engaged strategies to collaborate with patients, advocates, physicians, nurses, and social workers to design obstetric HHLN interviews. Interviews focused on four HHLN domains: employment, public benefits, housing, and safety. We applied iterative transcript‐based coding to develop and refine a codebook, assess interrater reliability (Cohen's kappa >0.85), and analyze themes.

**Results**: Forty‐eight obstetric patients (94% pregnant, 6% postpartum), aged 19‐39 years, participated. Twenty participants (42%) identified as Black, 26 as Latine (54%), and 2 as white (4%). All but two participants had public insurance (67%) or were uninsured (27%). In interviews, when asked generally, 92% of participants denied having HHLN. However, 70% of all participants screened positive in the HHLN survey, and when queried about HHLN domains, 95% described obstetric‐specific HHLN (Figure). The majority of participants (77%) experienced two or more HHLN, while few faced only an isolated HHLN or none (Figure). The most prevalent obstetric HHLN related to employment (e.g. pregnancy‐related workplace discrimination), poor access to public benefits, or substandard housing (Table). Participants described limited options for recourse when HHLN negatively affected their pregnancies (Table).

**Conclusion**: HHLN burdened nearly all low‐income obstetric patients in this study, but most did not realize they had unmet legal needs. Evidence‐based interventions are needed to increase awareness of obstetric HHLN and protect pregnant people from the obstetric consequences of HHLN.

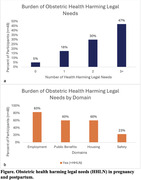

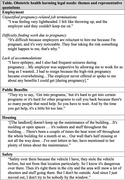

## 782 | Transcerebellar Diameter in Fetal Growth Restriction ‐ Does Concordance with Estimated Fetal Weight Matter?

Jenna S. Silverstein^1^; Emily Schneider^2^; Meralis V. Lantigua‐Martinez^3^; Gabrielle K. Concepcion‐Taveras^3^; Young Mi Lee^4^; Martin Chavez^5^; Ashley S. Roman^4^; Justin S. Brandt^4^; Erinn M. Hade^3^; Steven Friedman^3^; Christina A. Penfield^4^

*^1^Sister of Charity Hospital, Buffalo, NY; ^2^NYU Grossman Long Island School of Medicine, Mineola, NY; ^3^NYU Grossman School of Medicine, New York, NY; ^4^NYU Langone Health, New York, NY; ^5^NYU Langone Hospital, New York, NY*

10:30 AM ‐ 12:30 PM

**Objective**: Fetal growth restriction (FGR) is a significant risk factor for perinatal morbidity and mortality. Differentiating pathologic FGR due to placental insufficiency from a benign constitutionally small fetus remains challenging. We evaluated whether discordance between transcerebellar diameter (TCD) and estimated fetal weight (EFW) was associated with signs of pathologic FGR.

**Study Design**: We conducted a multi‐center prospective study of singleton pregnancies with FGR (EFW < 10th%) diagnosed between 16 and 37.6 weeks gestation. We excluded those without confirmed first trimester dating, or with identifiable anatomic, genetic, or infectious etiologies of FGR. Cerebellum measurement was planned at each growth scan. Concordance between measures was defined as TCD < 25th% and EFW < 10th% and discordance TCD ≥ 25th% and EFW < 10th%. The primary outcome was a composite of signs of placental insufficiency (abnormal umbilical artery Dopplers, oligohydramnios, and/or abnormal antepartum testing). Secondary outcomes included gestational age (GA) at delivery, FGR resolution prior to delivery, neonatal outcomes, and hypertensive disorders of pregnancy (HDP). Associations between discordance and outcomes was estimated by the adjusted relative risk (95% confidence interval) through modified Poisson regression.

**Results**: Of the 128 participants enrolled, 68 had complete data on cerebellar measurements. Pregnancies with discordant measures were more likely to have signs of placental insufficiency compared to those with concordant measures (41.8% vs. 23.1%; aRR 1.63 (95% CI [0.57, 4.69])). Concordant pregnancies were diagnosed with FGR earlier and were more likely to resolve prior to delivery. Groups did not differ in GA at delivery, birthweight, rates of medically‐indicated preterm birth, rates of SGA, other neonatal outcomes, and HDP.

**Conclusion**: FGR pregnancies with discordant TCD and EFW may be associated with signs of placental insufficiency, but due to high variability in the association, further confirmation of these findings is warranted. TCD discordance may be a tool for differentiating pathologic from benign FGR.

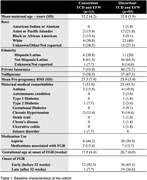

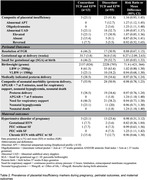

## 783 | Delayed Oral Misoprostol Dosing for Cervical Ripening: association with perinatal outcomes

Suna Njie^1^; Fei Xu^2^; Jennifer M. Baker^2^; Michael W. Kuzniewicz^3^; Monique Hedderson^2^; Mara Greenberg^4^

*^1^Kaiser Permanente Northern California, Oakland, CA; ^2^Kaiser Permanente Northern California, Pleasanton, CA; ^3^Kaiser Permanente Northern California, Kaiser Permanente Northern California, CA; ^4^Kaiser‐Permanente Northern California, Oakland, CA*

10:30 AM ‐ 12:30 PM

**Objective**: To examine dosing characteristics for misoprostol used as a cervical ripening agent in a large integrated health care system and evaluate association between dosing delay and perinatal outcomes.

**Study Design**: Retrospective study of all deliveries without prior cesarean that underwent induction of labor (IOL) from 2011‐2023 at Kaiser Permanente Northern California. For the proportion of patients undergoing IOL who received misoprostol for cervical ripening, dosing characteristics were examined. Delayed dosing among patients who received >1 misoprostol dose was defined as >4.5 hours between doses. Associations between delay characteristics and outcomes including cesarean delivery, chorioamnionitis, postpartum hemorrhage, time to vaginal delivery, and length of stay were examined in unadjusted and multivariable analyses.

**Results**: Among 126,864 induced labors without prior cesareans, 91,776 (72%) received misoprostol and of those 53,734 (58.6%) received >1 dose. Most misoprostol was dosed orally (96.7%) starting with an initial dose of 50mcg (98.8%), and subsequent doses of 100mcg (95%). Delayed dosing was present in 55.6% (29,863), and of those with delays 41% were delayed within 1 hour, 19% from 1‐2 hours and 40% >2 hours. Delay in misoprostol dosing was associated in a dose response manner with perinatal outcomes (Figure 1), with higher degrees of delay associated with higher rates of cesarean delivery, chorioamnionitis, and postpartum hemorrhage, lower rate of achieving vaginal delivery within 24 hours, and longer length of stay. In multivariable analysis, presence and degree of dosing delays were associated with increased adjusted risks of all adverse outcomes examined (Table 1).

**Conclusion**: Delayed oral misoprostol dosing was common in a large diverse cohort of induced labors. Frequency and degree of dosing delay was associated with adverse perinatal outcomes. This presents a potential opportunity to improve quality of intrapartum care and outcomes of labor.

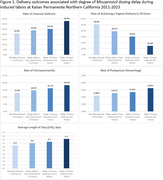

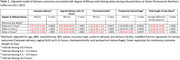

## 784 | The Impact of Pregnancy and Obesity on Glucose Metabolism in Mouse Skeletal Muscle

Jenny B. Koenig^1^; Ella Rust^1^; Liam S. Fitzgerald^1^; Simon Schenk^1^; Lindsey A. Burnett^2^

*^1^UC San Diego, La Jolla, CA; ^2^UC San Diego Medical Center, La Jolla, CA*

10:30 AM ‐ 12:30 PM

**Objective**: Obesity affects over 40% of reproductive‐aged women in the United States. This study aimed to examine the interactive effects of diet‐induced obesity and pregnancy on oral glucose tolerance and skeletal muscle insulin‐stimulated glucose uptake in a mouse model of diet‐induced obesity.

**Study Design**: C57/BL6/NJ mice (4 weeks old) were fed a low‐fat diet (LFD; 10% of calories from fat) or high‐fat diet (HFD; 60% of calories from fat) for 6 weeks before mating. An oral glucose tolerance test (OGTT; 2 g/kg) was conducted in late pregnancy (LP, E12.5‐17.5) or in age‐matched non‐pregnant (NP) mice. Skeletal muscle glucose uptake was assessed using a radiolabeled 2‐deoxyglucose (2DG) approach in paired soleus muscle, with and without a physiological insulin concentration (60 µU/mL). Statistical analysis was performed using 2‐way ANOVA with appropriate post‐hoc analysis and significance of P < 0.05.

**Results**: NP HFD‐fed mice weighed significantly more than LFD‐fed controls (30.20 vs. 19.97 g, P < 0.0001). Oral glucose tolerance as measured by area under the curve (AUC) was significantly different between LFD‐fed and HFD‐fed animals (NP: LFD 18,055 vs. HFD 24,089 mg · min/dL, P < 0.01) but was not between NP and LP within each diet (LFD: NP 18,055 vs. LP 16,074 mg · min/dL, P = 0.66; HFD: NP 24,089 vs. LP 21,311 mg · min/dL, P = 0.32). Specific to skeletal muscle, insulin‐stimulated glucose uptake (insulin 2DG uptake minus basal 2DG uptake) was decreased by HFD‐feeding (NP: LFD 0.69 vs. HFD 0.29 µmol/20 min/g, P = 0.09; LP: LFD 0.98 vs. HFD 0.19 µmol/20 min/g, P < 0.001), but not by pregnancy (LFD: NP vs. LP, P = 0.28; HFD: NP vs. LP, P = 0.90).

**Conclusion**: Diet‐induced obesity, but not pregnancy, significantly impairs oral glucose tolerance and insulin‐stimulated glucose uptake in skeletal muscle. This study provides valuable insights into the interactive effects of pregnancy and obesity on glycemia and skeletal muscle insulin resistance.

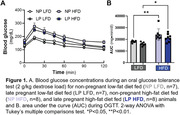

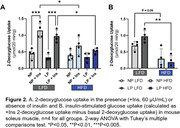

## 785 | Influence of Group Prenatal Care Participation on Attendance at the Postpartum Visit

Kate Tolleson^1^; Jessica Britt^2^; Moonseong Heo^3^; Amy Crockett^4^

*^1^NYU Grossman School of Medicine, New York, NY; ^2^Prisma Health, Prisma Health, SC; ^3^Clemson University, Clemson, SC; ^4^Prisma Health and University of South Carolina School of Medicine, Greenville, SC*

10:30 AM ‐ 12:30 PM

**Objective**: The 6 week postpartum visit (PPV) is an opportunity for family planning, evaluation of physical and mental health, and a link to follow‐up for chronic health conditions. Many patients miss this visit, with attendance averaging 61%.  Participation in group prenatal care (GPNC) encourages patient engagement during pregnancy and may improve PPV attendance. We compared rates of PPV attendance between GPNC and individual prenatal care (IPNC).

**Study Design**: Secondary analysis of a prospective clinical trial comparing GPNC vs IPNC on birth outcomes. The primary outcome variable was PPV attendance. Comparisons were made using 3 analytic approaches: intent‐to‐treat (ITT) including all randomized participants, modified ITT (mITT) including participants attending ≥1 visit in the assigned study arm, and a per compliance (PC) including those receiving ≥5 visits in the assigned study arm. Comparisons with χ‐square and t‐test were performed for mITT and PC groups and logistic regression was used to control for differences between groups.

**Results**: 2348 participants were enrolled with 1175 in GPNC and 1173 in IPNC. Participants were 40% Black, 36% White, and 21% Hispanic. Compared with IPNC, GPNC did not have higher rates of PPV attendance in ITT (61% GPNC vs 61% IPNC, RR 1.01, 95% CI 0.942‐1.07). However, with increasing participation, GPNC had significantly higher PPV attendance in mITT (70% vs 64%, RR 1.10, 95% CI 1.03‐1.17) and PC (76% vs 66%, RR 1.14,95% CI 1.07‐1.21). Adequacy of Prenatal Care Utilization Index ≥ 3 was associated with PPV attendance (RR 2.52,95% CI 2.16‐2.94) regardless of prenatal care type. Participants with 1+ living children had significantly less PPV attendance compared to participants with no living children (58% vs 65%, RR 0.892, 95% CI 0.84‐0.95).

**Conclusion**: There were no significant differences in PPV attendance between GPNC and IPNC in ITT, but increased participation in GPNC was associated with increased PPV attendance. Efforts to improve adequacy of prenatal care will be beneficial to all patients. Innovation in prenatal care delivery is needed to improve overall PPV attendance.

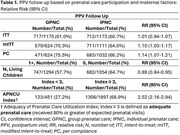

## 786 | Interdisciplinary Pregnancy Hypertension Alert System to Reduce Morbidity Associated with Hypertensive Disorders of Pregnancy (HDP)

Jessica O. Amoako^1^; Aliah L. Fonteh^1^; Jeanie Haggan^1^; Cori VanHouten^1^; Theresa Hyland^1^; Christine Coffey^1^; Rohit Sangal^2^; Katherine Campbell^2^

*^1^Yale New Haven Hospital, New Haven, CT; ^2^Yale School of Medicine, New Haven, CT*

10:30 AM ‐ 12:30 PM

**Objective**: To create a Pregnancy‐Related Hypertension Alert to trigger a timely assessment, diagnosis, and management of hypertensive disorders of pregnancy (HDP) in the Emergency Department (ED).

**Study Design**: Emergency Medicine (EM) and Obstetrics (OB) teams collaborated to develop and implement the Pregnancy‐Related Hypertension Alert. Patients with an elevated blood pressure (BP) defined as a systolic BP of ≥ 150 mmHg and or a diastolic of ≥ 100 mmHg at ≥ 20 weeks gestational age or within 6‐weeks postpartum met criteria for the alert. When triggered, an OB resident, OB nurse and EM team immediately evaluated patient using a standardized HDP care pathway.  Treatment according to the pathway includes the use of oral IR nifedipine, IV labetalol, IV hydralazine, and patients with mild elevated BP received oral antihypertensive.

**Results**: 80 patients (17 pre‐alert; 63 post‐alert) were included in the study. BP recheck within 15 minutes was noted in 41% of patients’ pre‐alert and increased to 86% post‐alert. Median time for patients to receive antihypertensives was 72 minutes [IQR 37.5‐195.5] in the pre‐alert period and improved to 23.5 minutes [IQR 15‐ 67 minutes] with the alert . Pre‐alert the ED median length of stay was 219 minutes [IQR 182‐297 minutes] and reduced to 147 minutes [IQR 73‐231 minutes] with the alert. Patient disposition was to Inpatient Obstetrics Unit (pre‐alert: 41%; post‐alert: 52%), IR/SICU (pre alert: 0%; post alert: 3%), Labor & Birth/Triage (pre alert: 6%; post‐alert:13%), transfer from satellite ED to main ED for additional management (5%), home (pre‐alert: 53%; post‐alert: 25%), other (2%).

**Conclusion**: After alert implementation, more patients achieved guideline recommended care including BP check and delivery of antihypertensives.  Future directions include ongoing optimization of the HDP alert and expanding coverage of the program across the health care system.

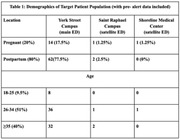

## 787 | The Association Between Maternal Immune Thrombocytopenia Purpura (ITP) and Adverse Perinatal Outcomes

Jill K. Kumasaka; Ava D. Mandelbaum; Bharti Garg; Aaron B. Caughey

*Oregon Health & Science University, Portland, OR*

10:30 AM ‐ 12:30 PM

**Objective**: Immune thrombocytopenia purpura (ITP) is a common cause of thrombocytopenia in pregnancy, however, limited data exist on perinatal complications associated with maternal ITP. This study aimed to evaluate the impact of maternal ITP on adverse perinatal outcomes.

**Study Design**: This is a retrospective cohort study of linked vital statistics and hospital discharge data among singleton, non‐anomalous births delivered between 23‐42 weeks in California (2008‐2020). Patients were stratified by diagnosis of ITP. Maternal outcomes included gestational hypertension, preeclampsia, gestational diabetes (GDM), preterm delivery < 37 weeks, postpartum hemorrhage, and severe maternal morbidity (SMM). Neonatal outcomes included cephalohematoma, intraventricular hemorrhage, and congenital thrombocytopenia. We utilized chi‐squared tests for univariate analyses and multivariable logistic regression analyses to evaluate the association of ITP with outcomes.

**Results**: Among 5,061,386 patients in our final sample, 4,346 (90 cases per 1000 deliveries) had a diagnosis of ITP. Compared to patients without ITP, individuals with ITP had increased rates of gestational hypertension (4.21% vs 3.44%, aOR = 1.18, 95% CI: 1.02‐1.37), preeclampsia (7.23% vs 3.80%, aOR = 1.74, 95% CI: 1.56‐1.95), GDM (11.99% vs 9.58%, aOR = 1.98, 95% CI: 1.10‐1.31), preterm delivery < 37 weeks (10.47% vs 6.39%, aOR = 1.69, 95% CI: 1.54‐1.86), postpartum hemorrhage (9.27% vs 3.37%, aOR = 2.63, 95% CI: 2.38‐2.91), and SMM (11.64% vs 1.18%, aOR = 9.21, 95% CI: 8.42‐10.07) (Table 1). Neonates born to mothers with ITP had higher rates of intraventricular hemorrhage and congenital thrombocytopenia, compared to those without ITP. These associations remained in sub‐analyses of vaginal versus cesarean deliveries only (Table 2).

**Conclusion**: Maternal ITP was associated with increased rates of gestational hypertension, preeclampsia, GDM, preterm delivery < 37 weeks, postpartum hemorrhage, and SMM. Further research to elucidate specific aspects of patients with ITP and perinatal complications will be important to identify those that may benefit from more aggressive interventions.

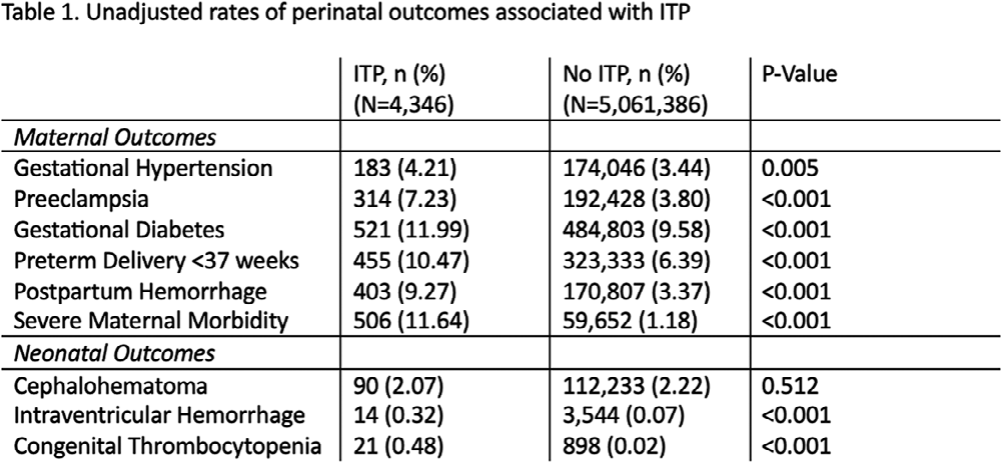

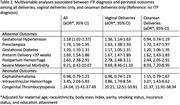

## 788 | Elevated Middle Cerebral Artery Peak Systolic Velocity and Donor Twin Demise in Twin‐Twin Transfusion Syndrome

Jimmy Espinoza^1^; Ramesha Papanna^2^; Anthony Johnson^3^; Eric P. Bergh^4^; Gustavo Vilchez^5^; Edgar A. Hernandez‐Andrade^2^; Sami Backley^1^; Felicia V. LeMoine^1^; Sen Zhu^6^; Arlyn Llanes^7^; Ramen H. Chmait^7^

*^1^Division of Fetal Intervention, Department of Obstetrics, Gynecology and Reproductive Sciences, McGovern Medical School at UTHealth Houston, Houston, TX; ^2^McGovern Medical School at UTHealth Houston, Houston, TX; ^3^McGovern Medical School University of Texas Health Science Center at Houston (UThealth), Houston, TX; ^4^UT Health, Houston, TX; ^5^UTHealth Houston, Houston, TX; ^6^McGovern Medical School at the University of Texas, Houston, TX; ^7^Keck School of Medicine, University of Southern California, Los Angeles, CA*

10:30 AM ‐ 12:30 PM

**Objective**: To determine the association of elevated donor middle cerebral artery peak systolic velocity (MCA‐PSV) without twin anemia polycythemia sequence (TAPS) and fetal demise among pregnancies complicated by twin‐to‐twin transfusion syndrome (TTTS).

**Study Design**: This prospective cohort study included TTTS cases that underwent laser surgery between 2006 and 2023 at two referral centers. The study was designed to: 1) explore the association of elevated donor MCA‐PSV (>1.5 multiples of the median) without TAPS with fetal demise of the donor twin; 2) to evaluate if donor or recipient MCA‐PSV is associated with an increased risk for their corresponding fetal death using receiving operator characteristic curve analysis. TAPS was defined as donor MCA‐PSV >1.5 MoM and recipient MCA‐PSV < 1.0 MoM or intertwin MCA PSV difference >0.5. Uni‐ and multivariable as well as Poisson (Zou's method) regression models were used to estimate odd ratios and relative risks (RR) of elevated donor MCA‐PSV without TAPS for donor demise, adjusted for TAPS, TTTS stage, intertwin size discordance >25%, low donor MCA pulsatility index, gestational age at surgery, and other confounders.

**Results**: Of 1,602 TTTS cases, 3.3% (n = 53) had elevated donor MCA‐PSV without TAPS and comprised our cases, which were compared to the other 1,549 cases (controls). Clinical characteristics are shown in Table 1; donor demise rate was higher among cases than controls  [45.3% (23/53) vs. 13.2% (205/1549), p< 0.001]. 1) Elevated donor MCA‐PSV without TAPS was an independent risk factor for donor fetal demise (RR: 2.86; 95 % CI: 1.78‐4.59; p< 0.001), and conferred the highest relative risk for demise of the donor twin (Table 2). 2) Donor MCA‐PSV was associated with donor fetal demise (AUC: 0.61; p< 0.001), but recipient MCA‐PSV was not associated with recipient fetal demise (AUC: 0.54; p = 0.2).

**Conclusion**: 1) Isolated elevated donor MCA‐PSV prior to laser is an important risk factor for donor fetal demise, adjusted for confounders including TAPS; 2) The association of TAPS with fetal death may be driven by elevated donor MCA‐PSV, not by recipient twin MCA‐PSV.

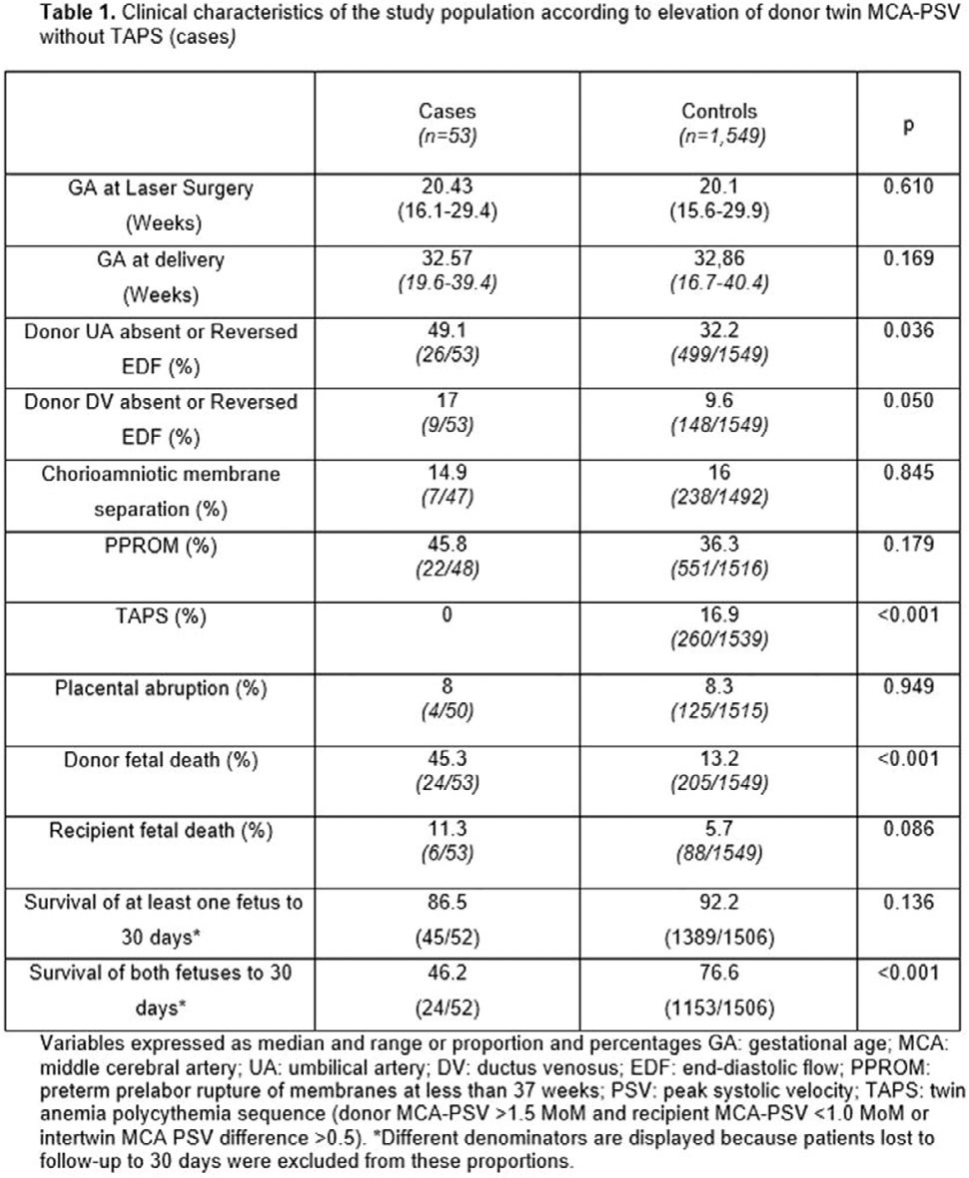

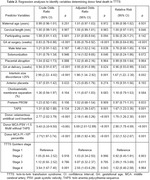

## 789 | Does Premature Rupture of Membranes Affect Adverse Health Outcomes in Second Trimester Medical Abortion

Jocelyn Wascher^1^; Neha Reddy^1^; Camille Johnson^1^; Eryn Wanyonyi^1^; Taytum Kahl^2^; Vanya Manthena^3^; Lahari Vuppaladhadiam^2^; Julie Chor^1^; Beth Plunkett^4^; Isa Ryan^5^; Olivert Mbah^5^; Jungeun Lee^6^; Emily Barker^7^; Laura Laursen^8^; Leanne McCloskey^9^; Sloane York^8^; Ashish Premkumar^3^

*^1^University of Chicago Medicine, Chicago, IL; ^2^University of Chicago, Chicago, IL; ^3^Pritzker School of Medicine, University of Chicago, Chicago, IL; ^4^Northshore Endeavor Health, Chicago, IL; ^5^NorthShore Health, Chicago, IL; ^6^Endeavor Health, Evanston, IL; ^7^Washington University in St Louis, St. Louis, MO; ^8^Rush University, Chicago, IL; ^9^Northwestern Medicine, Chicago, IL*

10:30 AM ‐ 12:30 PM

**Objective**: Previable and peri‐viable preterm rupture of membranes are frequently managed with medication abortion (MAB). Little is known about how ruptured membranes affect labor duration or maternal or health outcomes in MAB when compared to intact membranes.

**Study Design**: This retrospective cohort study examined patients undergoing second‐trimester (2T) MAB of a singleton pregnancy at 4 academic health centers from 2009‐2019. Patients who received a diagnosis of preterm labor or advanced cervical exam prior to MAB were excluded. The primary exposure was rupture of membranes (ROM) or intact membranes (IM) prior to MAB. Patients with infection diagnosed at the start of induction were included. The primary outcome was duration of labor in hours. Secondary outcomes were composite morbidity (uterine rupture, blood transfusion, intensive care unit admission, or readmission) and its components, estimated blood loss (EBL) ^3^500 mL and clinical chorioamnionitis (CC) Bivariate analyses were performed for composite morbidity and its components due to low frequency of outcome occurrence. Multivariate analyses were performed for EBL >500ml and CC. For the primary outcome, a survival analysis, censoring at the time of delivery, was performed.

**Results**: 1,341 patients were included, 344 (25.7%) with ROM and 997 (74.3%) with IM. On bivariate analyses, there were significant differences in baseline characteristics between groups (Table1). ROM was associated with decreased labor duration compared to IM (HR 2.5, 95% CI 2.1‐2.9); this was significant after adjusting for age, race, body mass index, parity and site of delivery (aHR 2.4, 95% CI 2.1‐2.8), (Figure1). There were no significant differences in secondary outcomes, with the exception of a higher likelihood of EBL ^3^500 (aRR 1.54, 95% CI 1.1‐2.2) and CC (aRR 1.9, 95% CI 1.3‐2.7).

**Conclusion**: ROM prior to 2T MAB is associated with shorter labor duration labor than IM. There was an increased likelihood of CC and higher blood loss without need for blood transfusion among people with ROM. These findings can be used in counseling patients undergoing 2T MAB.

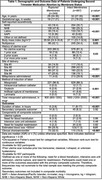

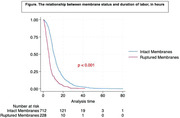

## 790 | Can We Train A.I. Chatbots to Replace Physicians for Counseling on Soft Markers?

Keren Khromchenko^1^; Joseph Canterino^1^; Jonathan D. Baum^1^; Karen Koscica^1^; Sheveta Jain^1^; Jennifer E. Powel^2^; Valeria Distefano^1^; Maria Martins^1^; Jonathan Faro^3^

*^1^Hackensack Meridian Health ‐ Jersey Shore University Medical Center, Neptune, NJ; ^2^Hackensack Meridian Jersey Shore University Medical Center, Neptune, NJ; ^3^Jersey Shore University Medical Center / Hackensack Meridian School of Medicine, Marlboro, NJ*

10:30 AM ‐ 12:30 PM

**Objective**: Chatbots offer a user‐friendly source of information. Recent studies have raised concerns about their accuracy. The World Health Organization (WHO) has warned that untested AI systems could harm patients and erode trust. The detection of soft markers for aneuploidy on ultrasound can cause significant anxiety. The accuracy and completeness of chatbot responses regarding soft markers are unknown. We sought out to evaluate chatbot performance on answering questions regarding soft markers for aneuploidy and whether training improves performance.

**Study Design**: A qualitative analysis was performed. ChatGPT Version 4o and Google Gemini 1.5 pro were queried on 8 isolated soft markers both prior to and after training using Society for Maternal‐Fetal Medicine (SMFM) Consult Series #57: evaluation and management of isolated soft ultrasound markers for aneuploidy in the second trimester. Queries were conducted in July of 2024. Query responses were graded as “acceptable” or “not acceptable” based on accuracy and completeness. Grading of responses were performed by the MFM co‐authors individually and then as a group to formulate a consensus.

**Results**: Pre‐training, 37.5% of ChatGPT responses and 50% of Gemini responses were graded as ‘not acceptable.’ For ChatGPT, all ‘not acceptable’ responses (3/3) were incomplete, with none incorrect. For Gemini, all ‘not acceptable’ responses (4/4) were incomplete, with one response also being incorrect. Post‐training, 25% of ChatGPT responses and 37.5% of Gemini responses were graded as ‘not acceptable.’ For ChatGPT, all ‘not acceptable’ responses (2/2) were incomplete, with none incorrect. For Gemini, all ‘not acceptable’ responses (3/3) were incomplete, with none incorrect.

**Conclusion**: While generative AI chatbots like ChatGPT and Google Gemini show potential as supplementary information sources on soft markers for aneuploidy, their responses often lack completeness, which limits their effectiveness. Training with specific medical publications improves performance, but these chatbots should not replace physician counseling.

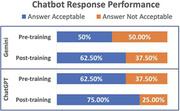

## 791 | Alternative Payment Models for Pregnancy Care: a Difference‐In‐Difference Analysis Among State Medicaid Programs

Jordan S. Stone^1^; Arina Chesnokova^1^; Allison Schachter^2^; Sindhu K. Srinivas^3^; Sunni Mumford^3^

*^1^Hospital of the University of Pennsylvania, Philadelphia, PA; ^2^University of Pennsylvania, Philadelphia, PA; ^3^University of Pennsylvania Perelman School of Medicine, Philadelphia, PA*

10:30 AM ‐ 12:30 PM

**Objective**: There is growing interest in using payment model design to improve pregnancy care delivery and reduce morbidity and mortality, particularly within Medicaid. Despite this, data on alternative payment models (i.e., those that establish a budget for a care episode, coupled with quality incentives) in pregnancy are sparse. The objective of this study is to evaluate whether the introduction of a mandatory Medicaid bundled payment program for pregnancy care was associated with changes in antenatal care delivery and maternal morbidity.

**Study Design**: This is a quasi‐experimental difference‐in‐difference (DiD) analysis comparing two states that implemented mandatory Medicaid bundled payment programs for pregnancy care (TN in 2011 and OH in 2014) to control states (GA, KS, KY, OK). Demographic variables and pregnancy outcomes were collected from birth certificate records between 2009 and 2019. Inclusion criteria were births reimbursed by Medicaid in intervention or control states with concordance between state of residence and state of birth.  A DiD analysis was conducted for co‐primary outcomes—gestational age at entry into prenatal care and maternal transfusion at time of delivery. These were chosen as proxies for modifiable aspects of pregnancy care within the domains of antenatal care delivery and maternal morbidity. The extended two‐way fixed effects estimator was used with the heterogenous DiD regression to account for staggered rollout in the intervention group.

**Results**: Between 2009 and 2019, we identified 2,227,136 births that met inclusion criteria. 55% were in the control group and 45% were in the intervention group. The pre‐intervention parallel trends assumption was satisfied between groups. In the heterogenous DiD regression model, no significant difference‐in‐differences were identified for the specified outcomes between the two groups (see table).

**Conclusion**: Mandatory Medicaid bundled payment programs did not have a significant effect on gestational age at entry into prenatal care or maternal transfusion rates. Additional studies are needed to further evaluate the impact of these models.

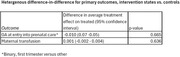

## 792 | Missed Connections: Exploring the Gap in Patient Awareness of Hypertensive Disorders of Pregnancy Diagnoses

Julia Thelen; Alyssa M. Hernandez; Eleanor Saffian; Anna Palatnik

*Medical College of Wisconsin, Milwaukee, WI*

10:30 AM ‐ 12:30 PM

**Objective**: To evaluate patients’ awareness of a hypertensive disorder of pregnancy (HDP) diagnosis following their most recent pregnancy.

**Study Design**: A 24‐question survey was given to patients with a recent HDP diagnosis who delivered at a tertiary care center in 2022‐2024. HDP was defined as gestational hypertension or preeclampsia. Descriptive statistics and bivariate analyses compared demographics, clinical characteristics, and survey responses stratified by ability to recall an HDP diagnosis.

**Results**: Of the 1,072 survey recipients, 355 (33.1%) responded and were included in analysis. The mean interval between childbirth and survey distribution was 11.2 months (±7.7m) and was not significantly different between study groups (p = 0.29). Among respondents, 87 (24.5%) were unaware of or could not recall their HDP diagnosis. 96.1% of patients who recalled their diagnosis recalled discussing blood pressure during their birth admission compared to only 71.6% of patients who did not recall their diagnosis. Patients who were less likely to be aware or were unable to recall a diagnosis of HDP were more likely to be Hispanic (p = 0.02), not college educated (p = 0.01), publicly insured (p = 0.02), WIC‐eligible (p = 0.05), diagnosed during the intra‐ or postpartum period (p = 0.001), and deliver at full term (p< 0.001). Patients with gestational hypertension were less likely to recall their diagnosis compared to those diagnosed with preeclampsia with severe features (p< 0.001).

**Conclusion**: Awareness and recall of a prior HDP diagnosis was influenced by demographic factors and clinical characteristics indicative of disease severity.  Since history of HDP is a significant risk factor for future pregnancies and lifelong cardiovascular health, further research exploring this gap in awareness is needed to optimize patient education during the index pregnancy.

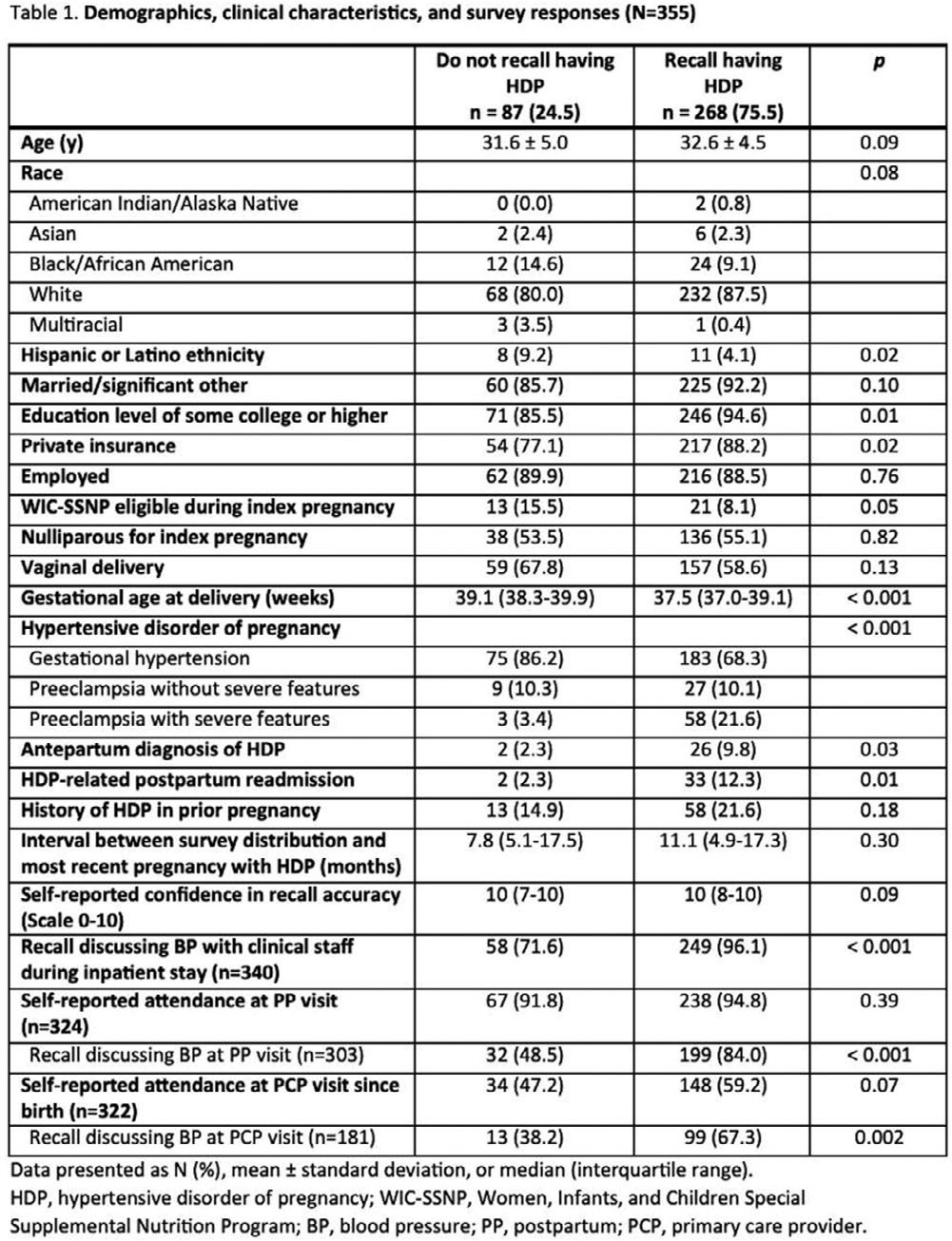

## 793 | Evaluating the Potential Relationship between sFlt‐1:PIGF and Hypertension Across Pregnancy and early Postpartum

Julia L. Berkowitz^1^; Stephen Juraschek^2^; Lara Kovell^3^

*^1^UMass Medical Center, UMass Medical Center, MA; ^2^Beth Israel Deaconess Medical Center, Boston, MA; ^3^UMass Memorial Health, Worcester, MA*

10:30 AM ‐ 12:30 PM

**Objective**: Hypertensive disorders of pregnancy are major contributors to adverse maternal outcomes including stroke, cardiovascular disease, and death. Vascular biomarkers, including the ratio of soluble fms‐like tyrosine kinase 1 and placental growth factor (Sflt‐1: PIGF) are independent predictors of gestational hypertension and preeclampsia. The aim of this study is to examine the trend in Sflt‐1: PIGF in pregnancy and post‐partum in women with and without hypertension (HTN).

**Study Design**: In a prospective, 1:1 case‐control design, we enrolled pregnant women with HTN and without HTN (control group) between 24‐32 weeks gestation from 2019‐2022. HTN was defined by a clinical diagnosis or baseline blood pressure (BP) ≥140/90 mm Hg. The control group had a systolic BP < 120 mm Hg and no HTN diagnosis. Serum was collected at baseline, at delivery admission, and postpartum day 1. Mixed effects tobit models were used to compare Sflt‐1:PIGF across HTN groups and over time, adjusted for age and BMI.

**Results**: At baseline, the HTN group had higher sFLT‐1(pg/mL): PIGF (pg/mL) (mean (SD): 3.9 (0.9)) versus the control group (2.1 (0.5)). Both groups had a significant increase in mean sFLT‐1:PIGF at the delivery admission (HTN: 20.7 (5.3); Control: 27.0 (7.2)) and postpartum (HTN: 56.7 (13.9); Control:  42.0 (10.6)); however, no significant difference was seen between these groups (see Figure 1). Across pregnancy and the early postpartum period, there was no significant difference in the mean sFLT‐1: PIGF ratio between the HTN and control groups (HTN: 13.06 (2.73); Control: 10.44 (2.24)).

**Conclusion**: While women with HTN had greater baseline sFLt‐1: PIGF than controls, no significant differences were found during delivery admission or postpartum. All women demonstrated a significant increase in sFLt‐1:PIGF during pregnancy with elevations continuing postpartum.

Future studies examining the impacts of HTN on subclinical cardiovascular injury and long‐term maternal outcomes are needed.

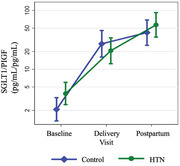

## 794 | The Screening Accuracy of Betke‐Kleihauer Test for Fetal‐Maternal Hemorrhage in the Antepartum and Postpartum Period

Juliana Poli^1^; Yulia Hin^1^; Heather VanderMeulen^1^; Nir Melamed^2^

*^1^University of Toronto, Toronto, ON; ^2^Division of Maternal Fetal Medicine, Department of Obstetrics and Gynecology, Sunnybrook Health Sciences Centre, Toronto, ON*

10:30 AM ‐ 12:30 PM

**Objective**: Accurate detection of fetomaternal hemorrhage (FMH) is important both antenatally (e.g., cases of abdominal trauma) and postpartum (to guide the RhIg dosage in RhD‐negative patients). Betke‐Kleihauer (BK) is the most commonly used quantitative screening test for FMH, but data on its screening accuracy in specific circumstances and the optimal screening threshold are conflicting. The current study aimed to estimate the screening accuracy of the BK test for large FMH and identify the optimal screening threshold in the antepartum and postpartum periods.

**Study Design**: A retrospective cohort study of patients who had a BK test at a single center (2014‐2022). The definitive diagnosis of large FMH (≥ 15 mL) was done by flow cytometry that was sent in cases of a positive BK test (≥ 2 mL). The screening accuracy of the BK test for large FMH was described using the area under the ROC curve (AUC), detection rate (DR), and false‐positive rate (FPR), and was stratified by the timing of testing (antepartum vs. postpartum).

**Results**: A total of 4,628 patients underwent BK testing during the study period, 2217 (47.9%) antenatally and 2,411 (52.1%) postpartum. The incidence of a positive BK was 9.1%. The discriminative accuracy of the BK for large FMH was high in the entire cohort (AUC 0.97 [95%‐CI 0.94‐0.99]) but was lower during the antepartum vs. postpartum period (0.88 [0.80‐0.96] vs. 0.997 [0.94‐1.00], p = 0.02). The highest BK threshold providing a DR of 100% for large FMH was 2.0 mL (FPR of 67% [58%‐76%]). A higher BK threshold of 7.5 mL was associated with a reduced DR (81% [85%‐97%) and lower FPR (9% [3%‐15%]). This threshold (7.5 mL) maintained a DR of 100% in the postpartum period (DR 100%, FPR 8% [3%‐14%]) but performed poorly antepartum (DR 57% [47%‐67%], FPR 14% [7%‐20%]).

**Conclusion**: The BK test is associated with a high FPR when using a conservative threshold of 2 mL that provides a 100% DR. Its screening accuracy is higher during the postpartum period, where a less strict threshold of 7.5 mL maintains the same DR of 100% with a lower FPR.

## 795 | Association of Body Mass Index with Adverse Pregnancy Outcomes When Stratified by Cardiovascular Health

Karen J. Gibbins; Nicole E. Marshall; Amy M. Valent

*Oregon Health & Science University, Portland, OR*

10:30 AM ‐ 12:30 PM

**Objective**: High body mass index (BMI) is associated with adverse pregnancy outcomes, but it is not a direct measure of cardiovascular health (CVH) and misclassifies many. We aimed to evaluate the association between BMI≥30 and adverse pregnancy outcomes stratified by CVH.

**Study Design**: Secondary analysis of nuMoM2b multicenter cohort of nulliparas, excluding pregestational diabetes and chronic hypertension. We used American Heart Association's Life's Simple 7 to assess CVH. Metrics included smoking, physical activity, healthy diet pattern, total cholesterol, and blood pressure. We omitted BMI given our objective, and we added triglycerides as these are a marker of CVH in pregnancy. We assigned 0 points for Poor category, 1 for Intermediate, and 2 for Ideal for each metric, with maximum total score of 14. Total score was categorized as Poor (1‐7), Intermediate (8‐11), or Ideal (12‐14). Outcomes included adverse perinatal outcomes previously linked to BMI (Table 1). Association between exposures and outcomes were calculated via logistic regression.

**Results**: 2462 participants were included. 473 had BMI≥30, 134 (5.4%) had Poor CVH, 1740 (70.7%) had Intermediate CVH, and 517 (21.0%) had Ideal CVH. Overall, BMI≥30 was associated with increased HDP (38.7 vs 19.6%, OR 2.6, 95% CI 2.1‐3.2), severe preeclampsia (8.3 vs 3.1%, OR 2.8, 95% CI 1.9‐4.3), LGA (10.6 vs 4.3%, OR 2.6, 95% CI 1.8‐3.8), GDM (7.4% vs 2.6%, OR 3.0, 95% CI 1.9‐4.6), and CB (37.0 vs 22.4%, OR 2.0, 95% CI 1.6‐2.5). However, when stratified by CVH category, these associations were altered. In the Ideal CVH category, BMI≥30 was only associated with LGA and GDM (Table 1). For some outcomes, like HDP, OR was higher in those with poor CVH and low BMI than those with Ideal CVH and high BMI (Figure 1).

**Conclusion**: Relying on BMI alone to assess pregnancy risk appears to overestimate risk of BMI≥30 in those with otherwise Ideal CVH and underestimate in those with BMI 18.5‐29.9 but Poor CVH. More comprehensive assessment of CVH could result in more accurate risk stratification.

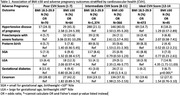

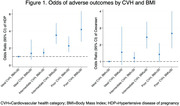

## 796 | Impact of Fetal Head Station on Maternal and Neonatal Outcomes in Second‐Stage Cesarean Delivery

Karolin Sokolik^1^; Asaf Romano^2^; Adi Litmanovich^3^; Ran Matot^2^; Natav Hendin^4^; Eran Hadar^4^; Ohad Houri^4^; Yossi Geron^4^

*^1^Helen Schneider Hospital for Women, Rabin Medical Center, Petach‐Tikva, Petach‐Tikva, HaMerkaz; ^2^Rabin Medical Center, Tel Aviv, HaMerkaz; ^3^Rabin Medical Center, Tel Aviv, Tel Aviv; ^4^Rabin Medical Center, Petach Tikva, HaMerkaz*

10:30 AM ‐ 12:30 PM

**Objective**: We hypothesized that second‐stage cesarean delivery with fetal head station below the ischial spine increases adverse maternal and neonatal outcomes.

**Study Design**: A retrospective cohort study of term pregnant women who underwent cesarean delivery at full cervical dilatation was assessed for adverse maternal and neonatal outcomes. The study was conducted in a tertiary medical center between 2012‐2023. Patients were categorized based on fetal head station in a manual examination: at or above ischial spines and below ischial spines. Maternal outcomes analyzed included cesarean section duration, need for push assistance, uterine extension, postpartum fever, hemoglobin drop, postpartum hemorrhage, blood transfusion, puerperal endometritis, and rehospitalization. Neonatal outcomes evaluated were 5‐minute APGAR scores, arterial pH levels, and NICU admissions.

**Results**: Of the 418 patients studied, fetal head station was at or above ischial spines in 276 patients and below spines in 142 patients. The below ischial spines group had a higher incidence of failed vacuum attempts (50.7% vs. 13.0%, P< 0.001) and required more blood transfusions (16.2% vs. 7.6%, P = 0.011). Additionally, neonatal arterial pH levels < 7.2 were significantly more common in the below ischial spines group (21.8% vs. 13.0%, P = 0.013). The composite neonatal outcomes were more common in the below ischial spines group (16.9% vs. 9.8%, P = 0.041, Table 1). However, in a subgroup analysis excluding cases with failed vacuum attempts, no significant differences in maternal and neonatal adverse outcomes were found, except for differences in surgery duration (Table 2).

**Conclusion**: Fetal head station per se is not associated with adverse maternal and neonatal outcomes in second‐stage cesarean delivery.

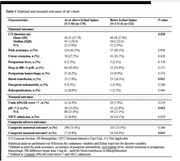

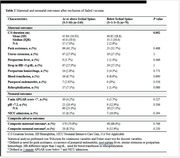

## 797 | Genome Sequencing Versus Exome Sequencing for Fetal Anomalies

Kate Swanson^1^; Teresa N. Sparks^2^; Nuriye Sahin‐Hodoglugil^1^; Billie R. Lianoglou^2^; Katie Tick^1^; Patrick Devine^3^; Mary E. Norton^2^

*^1^University of California, San Francisco, San Francisco, CA; ^2^Center for Maternal‐Fetal Precision Medicine, University of California San Francisco, San Francisco, CA; ^3^Genomic Medicine Laboratory, University of California, San Francisco, San Francisco, CA*

10:30 AM ‐ 12:30 PM

**Objective**: Genomic sequencing is increasingly recommended when fetal anomalies are detected. Exome (ES) and genome sequencing (GS) are both options; there are limited data comparing the two technologies. Our laboratory transitioned from ES to GS in July 2023; our objective was to compare ES versus GS in the setting of fetal anomalies.

**Study Design**: Prospective study of pregnancies with fetal anomalies undergoing ES or GS after non‐diagnostic microarray. ES was performed in our laboratory from 1/2018 to 7/2023, and GS from 7/2023 to 7/2024. Rapid testing was performed in ongoing pregnancies to prioritize turnaround time (TAT) when management might be impacted. The primary outcome was diagnostic yield, defined as a pathogenic or likely pathogenic variant consistent with the presentation. Secondary outcomes were TAT, diagnostic yield in cases of multiple vs single anomalies, and frequency of variants of uncertain significance (VUS) and secondary findings.

**Results**: A total of 384 patients underwent fetal sequencing, 54 with multiple anomalies and 330 with a single anomaly.  In all, 294 had ES and 90 had GS. Patients undergoing ES were more likely to undergo trio sequencing (69% vs 42%; p< 0.01). Multiple anomalies were present in 15% undergoing ES and 10% of GS cases (p = 0.21). Diagnostic yield was higher with multiple versus a single anomaly (31% vs 12%; p< 0.01) but did not differ by sequencing approach (14% for trio, 15% for duo or proband first; p = 0.7). The diagnostic yield was 16% with ES versus 10% with GS (p = 0.16). TAT with rapid sequencing was 30 days for ES vs 13 days for GS and was 51 vs 25 days for ES vs GS with routine (p< 0.01). The detection rate of VUS and secondary findings did not differ (Table).

**Conclusion**: GS for fetal anomalies was not associated with higher diagnostic yield or more VUS results when compared to ES, but was associated with shorter TAT. When decision making and care plans may be impacted by results, GS may be preferred for this reason.

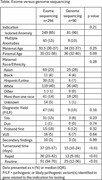

## 798 | Severe Maternal Ehlers‐Danlos Diagnoses Among Patients Referred for Hypermobility

Kate Swanson^1^; Mary E. Norton^2^; Teresa N. Sparks^2^

*^1^University of California, San Francisco, San Francisco, CA; ^2^Center for Maternal‐Fetal Precision Medicine, University of California San Francisco, San Francisco, CA*

10:30 AM ‐ 12:30 PM

**Objective**: Joint hypermobility may indicate multiple conditions, including hypermobile, vascular, and classic Ehlers Danlos syndromes (hEDS, vEDS, cEDS). While hEDS is associated with generally low risks and good pregnancy outcomes, vEDS carries a 5% risk of maternal mortality and cEDS an increased risk of spontaneous preterm birth. Our objective was to describe the prevalence of vEDS and cEDS in a cohort of preconception and pregnant individuals with hypermobility.

**Study Design**: Retrospective cohort study of individuals presenting for maternal genetic evaluation for hypermobility between 2023‐2024. Individuals were offered gene panel testing that includes the genes associated with vEDS and cEDS (COL3A1, COL5A1, COL5A2). A diagnosis of hEDS or hypermobility spectrum disorder (HSD) was made clinically as the genes associated with this condition are poorly understood. Primary outcome was genetic diagnosis of vEDS or cEDS. Secondary outcomes were clinical features with each of these diagnoses.

**Results**: 26 individuals were evaluated for hypermobility. Genetic testing was pursued in 19 patients; an additional 2 patients had already undergone testing with nondiagnostic results and a clinical history consistent with hEDS. Among those tested, 2/19 (11%) were identified to have pathogenic or likely pathogenic variants in COL3A1 consistent with a diagnosis of vEDS, and were managed with cardiac surveillance and cesarean section. One of these two individuals had previously been diagnosed with hEDS by a primary care physician. No individuals were clearly identified with cEDS, though one patient had a variant of uncertain significance in COL5A2. The remaining individuals were clinically diagnosed with hEDS or HSD and had routine obstetric management (Figure). The Table shows clinical features across diagnoses.

**Conclusion**: vEDS was diagnosed in 11% of individuals tested after referral for hypermobility. Genetic evaluation should be considered in individuals with hypermobility due to overlapping features across disorders, as well as significant implications for clinical management of when vEDS or cEDS are identified.

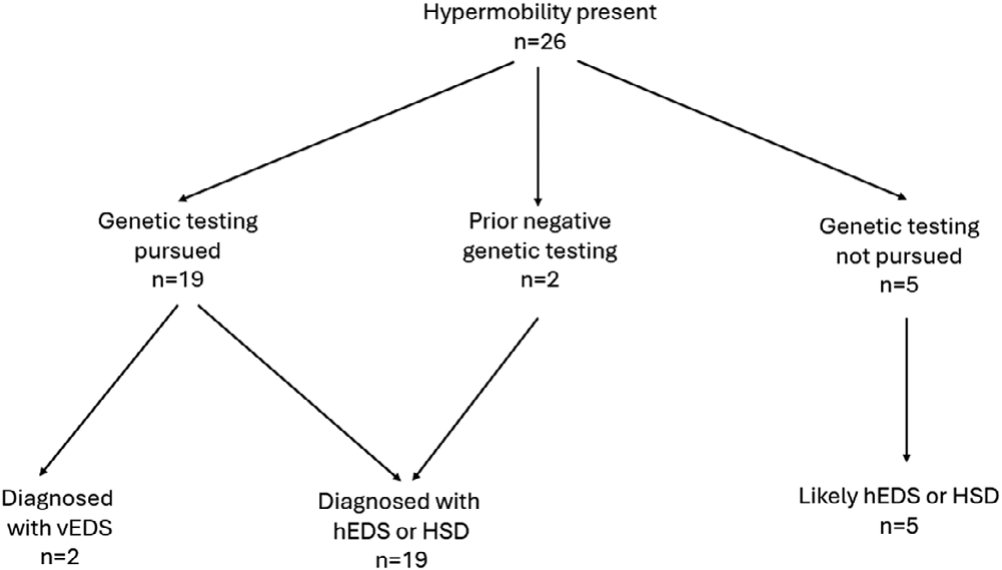

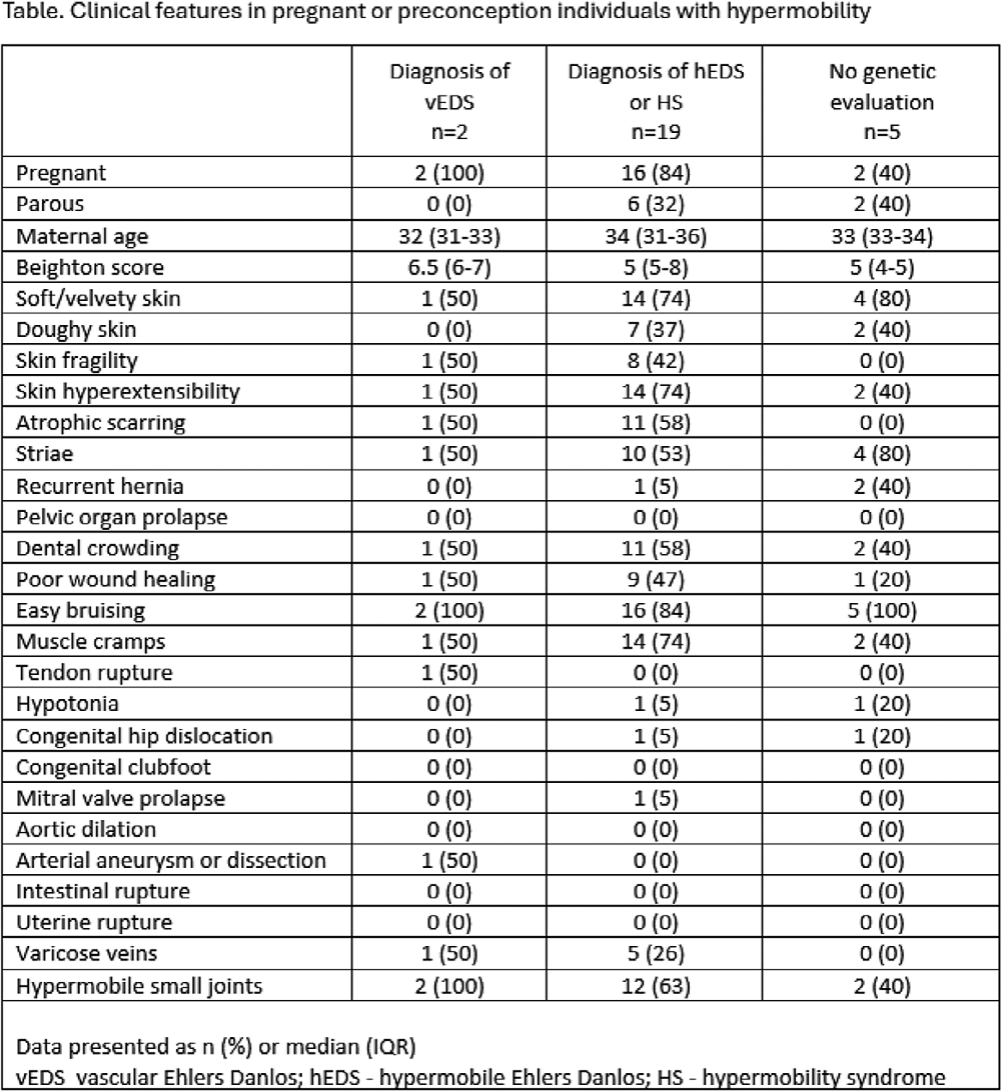

## 799 | Association of Maternal Body Mass Index with Longitudinal Fetal Growth Assessed by Three‐Dimensional Ultrasonography

Kathryn A. Wagner^1^; Jessica L. Gleason^2^; Zhen Chen^3^; Wesley Lee^4^; William A. Grobman^5^; Roger Newman^6^; Cuilin Zhang^7^; Stefanie N. Hinkle^8^; Luis F. Goncalves^9^; Seth Sherman^10^; Daniel W. Skupski^11^; Robert Gore‐Langton^10^; Angela Ranzini^12^; Edward Chien^13^; Ronald J. Wapner^14^; Sabrina Craigo^15^; Jagteshwar Grewal^16^; Katherine L. Grantz^2^

*^1^Massachusetts College of Pharmacy and Health Sciences, Boston, MA; ^2^Epidemiology Branch, NICHD‐NIH, Bethesda, MD; ^3^National Institute of Child Health and Human Development, Bethesda, MD; ^4^Baylor College of Medicine, Houston, TX; ^5^The Ohio State University, Columbus, OH; ^6^Medical University of South Carolina, Columbia, SC; ^7^National University of Singapore; ^8^University of Pennsylvania Perelman School of Medicine, Philadelphia, PA; ^9^Phoenix Children's Hospital, Phoenix, AZ; ^10^The Emmes Company, Rockville, MD; ^11^Weill Cornell Medicine, New York, NY; ^12^The MetroHealth System, Cleveland, OH; ^13^Cleveland Clinic, Cleveland, OH; ^14^Columbia University, New York, NY; ^15^Tufts Medical Center, Boston, MA; ^16^Eunice Kennedy Shriver National Institute of Child Health and Human Development, Bethesda, MD*

10:30 AM ‐ 12:30 PM

**Objective**: The current obesity epidemic is a major public health concern; from 2016‐2019, there was an 11% increase in women entering pregnancy with obesity. Maternal obesity is associated with fetal overgrowth as assessed by two‐dimensional (2D) ultrasonography. However, 3D ultrasonography can characterize fetal lean and fat tissue and organ volumes, which may provide additional insight into fetal metabolic programming than 2D. Therefore, we evaluated fetal 3D measures across pregnancy by maternal pre‐pregnancy BMI.

**Study Design**: In the NICHD Fetal 3D Study (2015‐2019), fetal body composition and organ volumes were measured at up to five ultrasound scans from 15‐40 weeks by certified sonographers. BMI (kg/m^2^), based on self‐reported pre‐pregnancy weight and height, were categorized as normal (18‐< 25; n = 1567), overweight (25‐< 30; n = 767), or obese (≥30; n = 468). Trajectories of fetal 3D measurements were modeled using linear mixed effect models. Overall and weekly mean differences in fetal growth were tested by BMI group, adjusted for covariates.

**Results**: Fetuses of women with overweight or obesity, compared to women with normal BMI, had significantly larger fractional arm and thigh volumes, starting at 25‐26 weeks and continuing through gestation. Proportional to fractional limb volume, fetuses of women with obesity had significantly smaller lean but larger fat limb volumes. Additionally, for overweight and obese groups, fetuses had significantly larger abdominal area and maximum abdominal subcutaneous tissue thickness (SCTT) from 29 weeks onward. Fetuses of women with overweight BMI had significantly larger lung volume from 22‐31 weeks, while those with obesity had significantly smaller lung volume from 17‐20 weeks, compared to those with normal BMI. No overall differences were observed among limb SCTT or other organs.

**Conclusion**: Fetuses of mothers with obesity had relatively smaller lean tissue but greater fat tissue accumulation throughout gestation, compared to normal BMI. Future research should examine whether fetal soft tissue differences may relate to metabolic dysfunction across the life course.

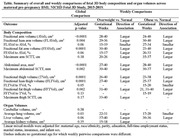

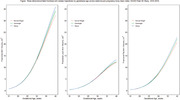

## 800 | Prenatal Clinic Management of High‐Risk Pregnant Patients

Kenechukwu Odenigbo^1^; Harini Pennathur^2^; Rachel A. Clark^1^; Jourdan E. Triebwasser^3^; Shayla Parthasarathy^1^; Michelle Moniz^3^; Lisa Kane Low^3^; Alex Peahl^4^

*^1^University of Michigan Medical School, Ann Arbor, MI; ^2^Texas A&M University School of Engineering Medicine; ^3^University of Michigan, Ann Arbor, MI; ^4^Michigan Medicine, Ann Arbor, MI*

10:30 AM ‐ 12:30 PM

**Objective**: To describe how prenatal clinics in the state of Michigan manage patients with high‐risk conditions in pregnancy.

**Study Design**: Prenatal care providers were identified and recruited through a systematic web search and snowball sampling. Semi‐structured surveys assessed prenatal care practices for birthing people with high‐risk conditions (e.g. pre‐existing diabetes, chronic hypertension). Clinics reported whether they managed, co‐managed, or referred patients. We assessed differences in practice by clinic type: Federally Qualified Health Centers, outpatient clinics linked to hospitals, and private practices using basic tests of comparison. Qualitative content analysis was used to summarize free‐response answers about additional services offered to medically complex patients. Funding provided by Blue Cross Blue Shield of Michigan.

**Results**: In total, 19 clinics representing 8/10 regions, with an average of 408 (range 20‐1700) patients per site, were included. Of the 19 clinics, 9/19 (47.4%) manage, 6/19 (31.6%) co‐manage, and 4/19 (21.1%) refer pregnant patients with pre‐existing type 2 diabetes. More outpatient clinics linked to hospitals managed patients with pre‐existing type 2 diabetes. For patients with chronic hypertension, 11/19 (57.9%) clinics manage, 6/19 (31.6%) co‐manage, and 2/10 (10.5%) refer patients. Management practices for chronic hypertension did not differ by clinic type. Additional services offered to medically complex patients included nurse navigators; care coordination by nurses, faculty, and trainees; support from social workers, therapists, doulas, or community health workers; and access to maternal fetal medicine physicians.

**Conclusion**: Many patients with type 2 diabetes or chronic hypertension require referrals or management from multiple providers. Future work is needed to understand the effects of varying management practices for chronic conditions on care access and outcomes, and the effects of additional services such as care coordination.

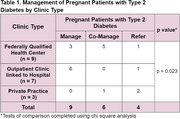

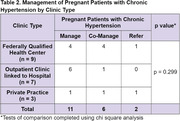

## 801 | Adjunctive Quadratus Lumborum Block to Reduce Opioid Use after Cesarean Delivery

Kevin S. Shrestha^1^; Yumo Xue^2^; Victoria C. Jauk^1^; Hanna Hussey^1^; Ayamo Oben^3^; Annalese Neuenschwander^1^; Michelle Tubinis^1^; Jeff M. Szychowski^4^; Mark Powell^1^; Alan T. Tita^1^; Casey Brian^5^; Ayodeji Sanusi^2^

*^1^University of Alabama at Birmingham, Birmingham, AL; ^2^Center for Women's Reproductive Health, University of Alabama at Birmingham, Birmingham, AL; ^3^Maternal Fetal Consultants of Houston, Houston, TX; ^4^Center for Women's Reproductive Health, University of Alabama at Birmingham, Birmingham, AL; ^5^West Virginia University, Morgantown, WV*

10:30 AM ‐ 12:30 PM

**Objective**: Peripheral nerve blockade in addition to neuraxial morphine (NM) and enhanced recovery protocols may improve analgesia following cesarean delivery (CD). We assessed if adjunctive quadratus lumborum (QL) block will reduce the oral morphine equivalent (OME) consumption for the first 24 and 48 hours after CD.

**Study Design**: Retrospective cohort comprising patients enrolled in a single tertiary center RCT of reduced NM dose (50mcg NM+QL block vs 150mcg NM+QL block) aimed at minimizing OME use and NM adverse effects after scheduled CD; and a historical cohort who received 150mcg of NM without a QL block. Patients in the RCT were excluded for preeclampsia, insulin‐treated diabetes, placental abnormalities or history of opioid use disorder. Intervention groups were 50mcg NM+QL, 150mcg NM+QL, and 150mcg NM without QL block. Comparison groups were 150mcg NM+QL vs. 150mcg NM without QL and 50mcg NM+QL vs. 150mcg NM without QL. Primary outcomes were total OME on postoperative days 1 and 2 and opioid use 24hr prior to discharge. Secondary outcomes included total OME, opioid adverse effects, and pain scores. Linear regression and log‐binomial models were used to calculate risk differences and 95% confidence intervals between groups.

**Results**: Of 243 patients were included, 43 were in the 50mcg NM+QL arm, 42 in the 150mcg NM+QL arm, and 158 in the 150mcg NM without QL arm. Patients in the 150mcg NM without QL arm delivered earlier and had higher rates of preeclampsia, pregestational diabetes and ASA class. There were no significant differences in the primary outcomes between all comparison groups. QL block was associated with lower parenteral opioid use and lower pain scores in the first 6 hours after surgery for patients receiving 150mg NM. There were no other significant differences in secondary outcomes.

**Conclusion**: Routine QL block in addition to multimodal pain management and NM did not significantly reduce total OMEs after scheduled CD however, it reduces parenteral opioid use and improve pain control in the immediate post cesarean period. Larger studies may be needed to confirm.

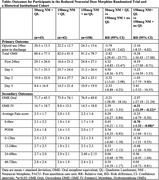

## 802 | Cost Effectiveness Analysis of Outpatient Versus Inpatient Antibiotic Treatment for Syphilis in Pregnancy

Kevin S. Shrestha^1^; Theodoros Giannouchos^1^; Jeff M. Szychowski^2^; Ashley N. Battarbee^3^; Akila Subramaniam^3^; Alan T. Tita^1^; Ayodeji Sanusi^3^

*^1^University of Alabama at Birmingham, Birmingham, AL; ^2^Center for Women's Reproductive Health, University of Alabama at Birmingham, Birmingham, AL; ^3^Center for Women's Reproductive Health, University of Alabama at Birmingham, Birmingham, AL*

10:30 AM ‐ 12:30 PM

**Objective**: Inadequate treatment and inequitable access to care contribute to the rising incidence of congenital syphilis in the US. In pregnancy, inpatient treatment is offered due to increased risk of Jarisch‐Herxheimer reaction (JHR) and other adverse outcomes. Our objective is to determine the cost‐effectiveness of admissions averted by comparing inpatient versus outpatient treatment for initial antibiotic dose in pregnant patients with syphilis.

**Study Design**: We performed a cost effectiveness analysis using a decision tree, for viable (≥23 weeks), pregnancies with syphilis, to compare initial treatment in two settings: (1) inpatient and (2) outpatient. Input parameters included the probability of JHR resulting in a 3‐day admission to the hospital among outpatient treatment, the total cost of treatment in each setting, and the total cost of subsequent admissions. Costs were estimated using a cost‐to‐charge ratio obtained from charges at a US, tertiary, academic hospital in 2024 USD, while the probability of admission was equated to the JHR incidence obtained from the literature (1.72%). An incremental cost‐effectiveness ratio (ICER) was calculated using the ratio of differential total costs and admission probabilities between the two settings from a healthcare system perspective. A probabilistic sensitivity analysis using 100 Monte Carlo simulations was conducted to assess parameter uncertainty.

**Results**: The average treatment cost of pregnant women with syphilis was estimated at $2,720 and $170 in an inpatient and outpatient setting respectively, while the cost of a 3‐day admission following JHR in the outpatient settings was around $9,000. The ICER was estimated at $134,463 (95% Confidence Interval: $121,874‐$149,052) per admission averted.

**Conclusion**: Initial antibiotic treatment in an outpatient setting for syphilis in pregnancy is cost‐effective compared to patient treatment in terms of admissions averted. Future work should explore whether the costs of treating women with syphilis in an inpatient setting are justified by exploring birth‐related health outcomes (e.g. still‐births averted).

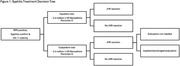

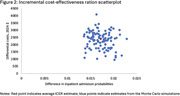

## 803 | Impact of Iron Deficiency in Pregnancy on Childhood Neurodevelopmental Outcomes

Kimberly Ryan; Lucy Ward; Joseph Shatzel; Libby Nousen; Jamie O. Lo; Hanna Goustafsson; Ashley E. Benson; Elinor Sullivan

*Oregon Health & Science University, Portland, OR*

10:30 AM ‐ 12:30 PM

**Objective**: Iron deficiency in pregnancy is common and associated with perinatal morbidity. Retrospective studies suggest an association between prenatal iron deficiency and worsened long term childhood neurodevelopmental outcomes. The objective of this study was to evaluate childhood neurodevelopment from infancy to two years of age and perinatal outcomes associated with iron deficiency in pregnancy.

**Study Design**: Prespecified secondary analysis of a prospective cohort study evaluating the influence of prenatal exposures on child neurodevelopmental outcomes. Patients were recruited prior to 24 weeks gestation. Pregnancy and health information was extracted from medical health records. Prenatal iron deficiency was defined as serum ferritin <30ng/mL. Childhood neurodevelopment was assessed through the Ages and Stages Questionnaire (ASQ) at 1, 6, 12,18 and 24 months of age.  Mean ASQ subscale scores and their 95% confidence intervals were graphed across 1‐24 months.

**Results**: A total of 147 maternal‐child dyads met inclusion criteria; 104 (70.7%) had prenatally diagnosed iron deficiency, 43 (29.3%) did not have iron deficiency. These groups had similar baseline characteristics (Table 1). The mean ferritin in pregnancy in the iron deficient group was 23.8 ng/mL, compared to 64.9 ng/mL in those without iron deficiency. There was a trend towards lower ASQ fine motor skills, persisting from 1 to 18 months of age, in children with maternal prenatal iron deficiency as compared to those without, however, this did not achieve significance (Figure 1). Otherwise, other ASQ scores were similar across the timepoints.   

**Conclusion**: Although prenatal iron deficiency was not associated with significant differences in childhood neurodevelopmental outcomes via the ASQ, larger studies are warranted to evaluate the relationship between iron deficiency and diminished motor skills as this trend has been previously described in retrospective studies.

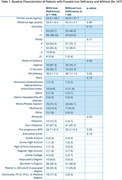

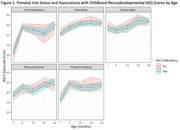

## 804 | Impact of Infant Gestational Age at Birth on Postpartum Healthcare Receipt

Kristan A. Scott^1^; Evan Miller^2^; Jennifer F. Culhane^3^; Jay Greenspan^2^; Sara Handley^4^; Justin Y. Lo^5^; Lindsey Knake^6^; Kathryn M. McKenney^7^; Kevin Dysart^2^; Heather Burris^1^

*^1^Children's Hospital of Philadelphia, Philadelphia, PA; ^2^Nemours Children's Hospital, Wilmington, DE; ^3^Yale School of Medicine, New Haven, CT; ^4^Children's Hospital of Pennsylvania, Philadelphia, PA; ^5^KFF, Washington, DC; ^6^University of Iowa, Iowa City, IA; ^7^University of Colorado, Denver, CO*

10:30 AM ‐ 12:30 PM

**Objective**: Up to 40% of obstetric patients do not attend postpartum visits. Birthing parents of preterm infants are often sicker than those of full‐term infants. Yet parents of preterm infants may forgo postpartum visits to be present in the neonatal intensive care unit (NICU). We evaluated whether parents of preterm infants were less likely to receive postpartum care than parents of full‐term infants.

**Study Design**: Retrospective cohort study of births in Epic Systems’ Cosmos research platform (2018‐2023), a US‐based electronic health record database with anonymized, de‐identified, patient‐level data. We explored bivariate associations of infant gestational age with postpartum visits. Logistic regression models estimated the odds of missing postpartum visits among parents of infants of varying gestational ages compared to parents of full‐term infants (39‐40 weeks). Models first adjusted for patient characteristics (age, parity, body mass index, race and ethnicity, insurance, smoking, year, and CDC Social Vulnerability Index), and then adjusted for morbidities (diabetes, hypertension, and cesarean birth) (Table 1).

**Results**: In bivariate analyses, parents of infants < 24 weeks’ gestation missed postpartum visits significantly more often than parents of full‐term infants (37% vs. 32%, respectively, OR 1.26 [1.13–1.40]) (Table 2). Adjustment for patient characteristics did not meaningfully change estimates. However, with adjustment for morbidities, parents of preterm infants in every gestational age category had significantly higher odds of missing postpartum visits compared to parents of full‐term infants; adjusted odds ratios ranged from 1.11 (1.06–1.17) to 1.20 (1.08–1.33) among parents of infants 24‐27 and < 24 weeks’ gestation, respectively.

**Conclusion**: Parents of preterm infants are at higher risk for not receiving postpartum healthcare than parents of full‐term infants. Interdisciplinary, innovative approaches to deliver care to this high‐risk group, potentially in the NICU, are needed.

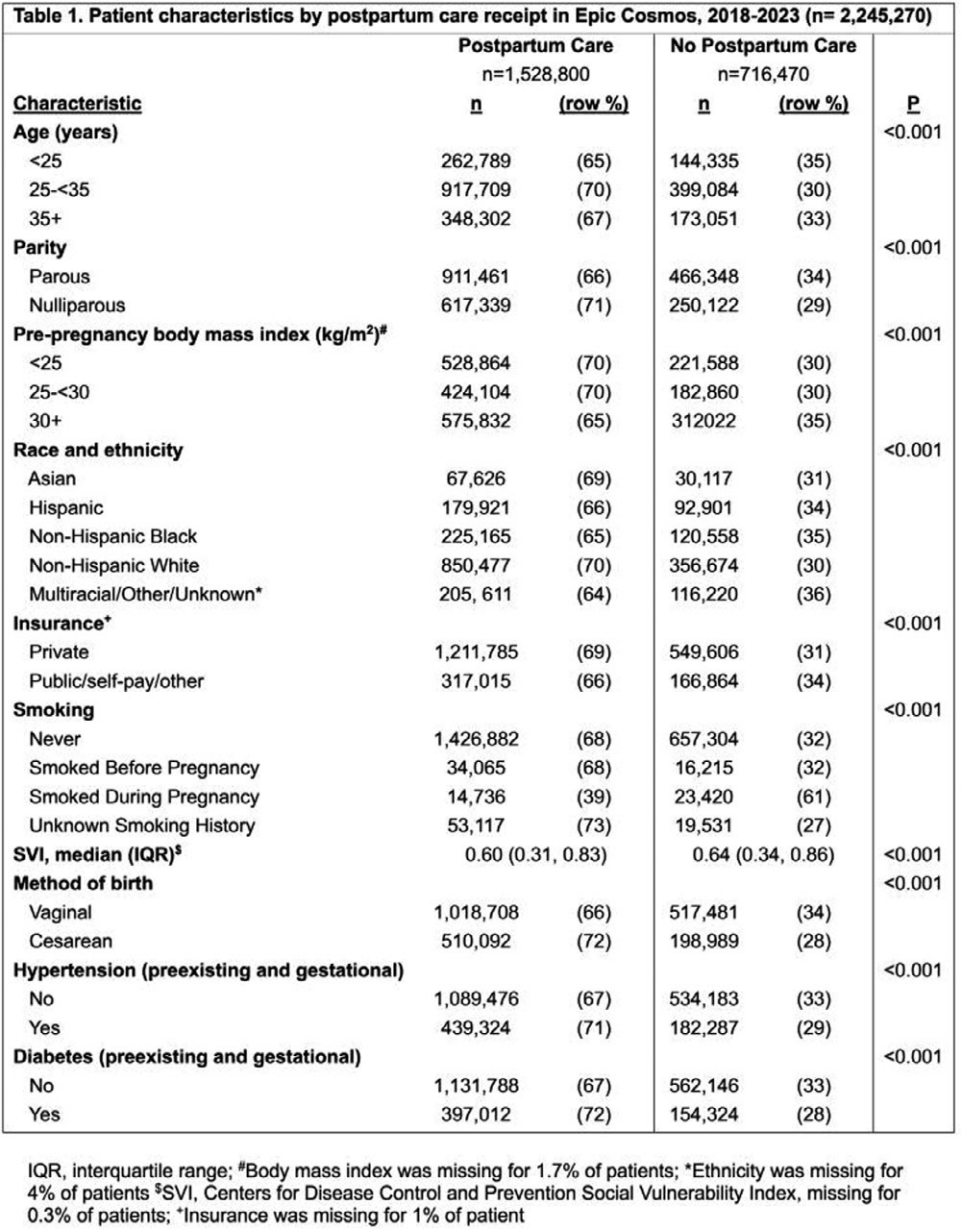

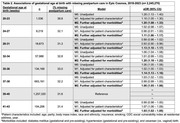

## 805 | Maternal Fetal Medicine Fellow Experience with Placenta Accreta Spectrum Care

Kristen L. Moriarty^1^; Andrea D. Shields^2^; Nicole R. Gavin^3^

*^1^UCONN Health, Farmington, CT; ^2^University of Connecticut Health, Avon, CT; ^3^University of Connecticut, Farmington, CT*

10:30 AM ‐ 12:30 PM

**Objective**: Evaluate MFM fellows' experience with PAS management and their desire to perform PAS surgeries post‐fellowship.

**Study Design**: A 43 question IRB approved survey regarding PAS exposure was validated with experts and novice learners. The survey was distributed via REDCap to fellowship program directors for dissemination from June‐July 2024. Baseline demographics: fellowship year, region, number of fellows in the program, and if home program was a PAS center of excellence. Primary outcome: fellows' desire to participate in PAS post‐fellowship. Secondary outcomes: various aspects of PAS participation. Chi square and pearson's t‐test analyzed outcome variables.

**Results**: 42 MFM fellows responded, distributed evenly by year (PGY5‐7+: 33.3%, 28.6%,  38.1%) and region (Northeast 38.1%, South 31%, Midwest 19%, West 11.9%). Most programs (52.4%) had 5+ fellows, and 64.3% identified as PAS center. Prior to fellowship, 90.5% of fellows participated in some aspect of PAS care, with 52% performing 1‐5 cases. 60% of fellows previously in OBGYN practice had performed PAS cases as primary surgeons. 64.3% of fellows reported training in a PAS program influenced their fellowship ranking. During fellowship, all respondents participated in ultrasound and intrapartum PAS management, with 95.2% involved in surgical management. Over one‐third (35.7%) performed 1‐5 PAS cases during fellowship and there was no difference in the number of hysterectomies performed per fellowship year (p = 0.916). Two‐thirds (66.7%) expressed a desire to perform PAS cases post‐training and those who desired felt more confident managing the entire surgery for PAS (p = < 0.001) as well as managing the routine post‐operative care (p = 0.025) and showed a trend toward training at a PAS center of excellence (p = 0.08).

**Conclusion**: 2/3 of fellows intend to perform PAS cases post‐training and felt confident managing the entire surgery for PAS patients and had a trend to have trained a PAS center of excellence. A solid foundation in PAS identification, management, and care coordination during training is essential for fellows' future participation in PAS care.

## 806 | MEWS Due to Severe Hypertension Prior to Delivery: Clinical Characteristics and Outcomes

Kristen A. Cagino^1^; Beverly Red^1^; Joe Haydamous^2^; Tala Ghorayeb^3^; Sandra Sadek^4^; Sean C. Blackwell^5^; Baha M. Sibai^5^

*^1^UT Houston, Houston, TX; ^2^Division of Maternal‐Fetal Medicine, Department of Obstetrics, Gynecology and Reproductive Sciences, McGovern Medical School at UTHealth Houston, Houston, TX; ^3^McGovern Medical School at UTHealth, Houston, TX; ^4^University of Texas Health Science Center in Houston, McGovern Medical School, Houston, TX; ^5^McGovern Medical School at UTHealth Houston, Houston, TX*

10:30 AM ‐ 12:30 PM

**Objective**: Maternal early warning systems (MEWS) are used to identify individuals at risk for adverse outcomes related to severe hypertension (HTN). We aimed to describe the frequency, demographics, management, and adverse outcomes of MEWS for severe HTN.

**Study Design**: A retrospective cohort study on the pragmatic management of MEWS for severe HTN from March to Dec 2022 at a level IV center. Individuals that triggered a MEWS antenatally or intrapartum for systolic BP > 160 or diastolic BP > 110 mm Hg were included and any subsequent MEWS were collected. Those with MEWS exclusively postpartum were excluded. MEWS were triggered after two sustained severe BPs prompting a provider to bedside within 30 minutes. Composite maternal adverse outcomes (CMAO) included eclampsia, stroke, pulmonary edema, acute kidney injury, myocardial ischemia, placental abruption, or death. Descriptive statistics were performed using N (%), mean (standard deviation [+ SD]) and median (interquartile range [IQR]). 95% confidence intervals (CI) were calculated for CMAO.

**Results**: There were 3933 deliveries during the study period and 100 individuals (2.5%) who met inclusion criteria with a total of 541 MEWS—a median of 4 (IQR 2‐8) per person. 52% were nulliparous, 21% had preeclampsia in a previous pregnancy, and 54% had chronic hypertension. Mean systolic BP and diastolic BP were 172 + 12 and 98 + 13 mm Hg, respectively. Symptoms occurred in only 15% of MEWS. The rate of CMAO was 8% (95% CI; 3‐15%). In response to MEWS, acute antihypertensives were given in 41%, both acute and oral in 20%, and oral only in 7% of cases. 31% of all MEWS (n = 170) were not treated. When evaluating adverse outcomes, only one (acute kidney injury) occurred after an untreated MEWS.  

**Conclusion**: In our population, the likelihood of developing MEWS for severe HTN before delivery was 2.5% and adverse outcomes occurred in approximately 1 in 10. The majority of cases had no symptoms other than elevated BP. BPs were not treated in 31% of MEWS and only 1 out of 170 were associated with the development of CMAO.

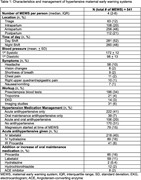

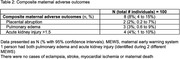

## 807 | Improving Understanding of Macrosomia With Graphics‐Based Educational Tool: A Randomized Clinical Trial (MATE)

Kristen A. Cagino^1^; Myra Kurjee^1^; Emily Hyde^1^; Han‐Yang M. Chen^2^; Hector M. Mendez‐Figueroa^3^; Suneet P. Chauhan^4^

*^1^UT Houston, Houston, TX; ^2^McGovern Medical School at UTHealth Houston, Houston, TX; ^3^McGovern Medical School at UTHealth, Houston, TX; ^4^Delaware Center of Maternal‐Fetal Medicine at Christiana Care, Delaware, DE*

10:30 AM ‐ 12:30 PM

**Objective**: Our pilot study noted that in our population, understanding of risk factors/complications (RF/C) and management options (MO) for macrosomia (birthweight > 4,000 g) was poor. We hypothesized that a graphics‐based education tool (GBET) would improve knowledge about macrosomia.

**Study Design**: Inclusion criteria for our randomized clinical trial (NCTO6281301) comprised of individuals at 18‐55 years, with singleton pregnancy delivering at > 36 weeks. After consent, participants were randomized to either routine care or GBET (Fig 1). To assess the knowledge about macrosomia, a questionnaire consisting of 17 questions relating to the RF/C and MO of suspected macrosomic fetuses was administered to eligible candidates. The primary outcome was the overall score on the questionnaire. Secondary outcomes were individual scores on the RF/C (n = 11) and MO (n = 6). We estimated a priori that 100 participants in each group would provide 90% power to detect a 10% difference in the mean macrosomia questionnaire score (baseline score 56% + SD of 12, alpha = 0.05). Descriptive statistics were used for baseline characteristics. Chi‐squared test was used to compare categorical variables and Student's t‐test for continuous variables.

**Results**: During the study period from Jan to July 2023, 230 eligible individuals were approached and 200 (87%) agreed to participate; of them, 103 received the GBET. Baseline demographics were similar. The majority (42%) of respondents self‐identified as Black, 60% were employed, 56% had some level of college education, and 30% lived below the poverty line. There were 41% nulliparous, 67% with a BMI > 30 kg/m^2^ and 16% with diabetes. The primary outcome was significantly higher in those who received the GBET (70% versus 63%, p < 0.01). The RF/C score was also higher in the GBET group (71% versus 63%, p = 0.01); however, the MO score was similar between the groups (64% versus 67%, p = 0.12).

**Conclusion**: In our population, a graphics‐based education tool improved patient knowledge on the risk factors / complications for macrosomia, but not their management options.

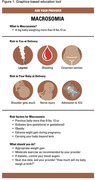

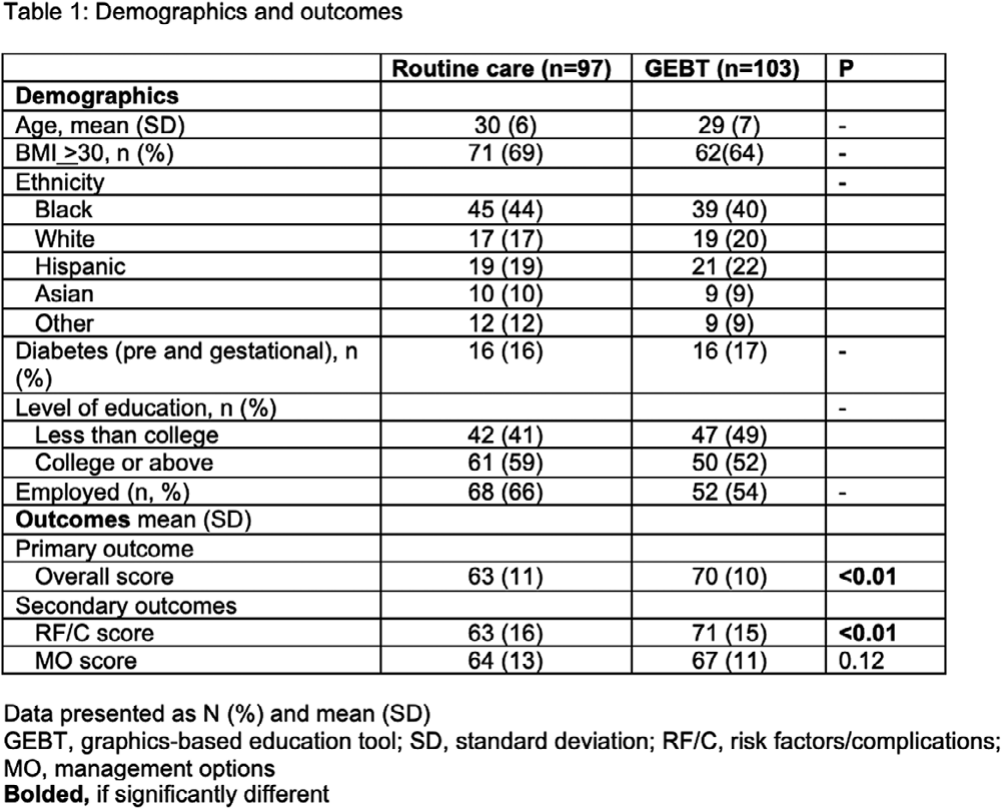

## 808 | Effects of Co‐Occurring Opioid Use and Mood Disorders on Neonatal Outcomes in Pregnancy

Kristin C. Prewitt; Bharti Garg; Kimberly Ryan; Sarena Hayer; Ashley E. Benson; Aaron B. Caughey; Jamie O. Lo

*Oregon Health & Science University, Portland, OR*

10:30 AM ‐ 12:30 PM

**Objective**: Co‐occurring prenatal mood disorders and opioid use disorders are the leading cause of pregnant‐related mortality, but the combined effect on neonatal morbidity is poorly understood. Thus, the objective of this study is to better understand associations between co‐occurring prenatal opioid use and mental health diagnoses on neonatal outcomes.

**Study Design**: This retrospective cohort study used California linked vital statistics and hospital discharge data (2008‐2020). Patients were categorized into opioid‐users, mental health disorders, both, or neither. We included singleton gestations delivered between 23‐42 weeks and excluded individuals using other substances. Exposure (opioid use and mental health disorders) and outcomes were identified using birth certificate and ICD‐9/10 codes. Results were analyzed by chi‐square tests and multivariable Poisson regression models. Adjusted risk ratios (aRR) with 95% confidence intervals (CI) were estimated.

**Results**: A total of 5,423,898 pregnancies were included, and of those,6,285 (0.12%) had opioid‐related diagnosis, 165,121 (3.04%) had mental health disorders and 1,599 (0.03%) had a dual diagnosis. The impact of opioid use and mental health disorders (3.4%; aRR = 3.39 (2.59‐4.43)) appears to have an additive effect compared to either opioid use (2.3%; aRR = 2.62 (2.21‐3.12)) or mental health disorders (1.4%; aRR = 1.48 (1.42‐1.54)) on rates of preterm birth < 32 weeks. Compared to no opioid use or mental health disorders, the risk of neonatal respiratory distress syndrome was higher with dual diagnosis (13.8%; aRR = 4.14 (3.65‐4.68)), opioid use (9.1%; aRR = 3.13 (2.89‐3.39)) and mental health disorders (5.8%; aRR = 1.79 (1.76‐1.83)). Similarly, the risk of NICU admissions was significantly higher in individuals with both opioid use and mental health disorders.

**Conclusion**: Our study suggests that co‐occurring psychiatric disorders with opioid use is associated with worse neonatal outcomes. As the opioid epidemic continues, our findings can shape clinical management.

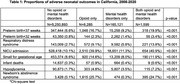

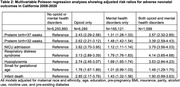

## 809 | The Fetal Membrane Metabolome: Arachidonic Acid and Sex Steroid Metabolism Perturbations Associated with Preterm Rupture

Emilie M. Stylli^1^; Briana Ferguson^1^; Rita Leite^1^; Rachel Ledyard^2^; Heather Burris^2^; Lauren Anton^1^; Kristin D. Gerson^1^

*^1^University of Pennsylvania Perelman School of Medicine, Philadelphia, PA; ^2^Children's Hospital of Philadelphia, Philadelphia, PA*

10:30 AM ‐ 12:30 PM

**Objective**: Fetal membranes serve critical immune and mechanical functions in pregnancy, as well as provide signals initiating term and preterm labor. Despite these roles, this feto‐maternal interface is often overlooked in reproductive research. Metabolomics has emerged as a powerful tool to understand biochemical footprints of cellular function. We sought to identify differences in the fetal membrane metabolome from cases of preterm premature rupture of membranes (PPROM) compared to term controls.

**Study Design**: Untargeted metabolomics was performed on amnion and chorion from cases of PPROM (n = 25) and term controls (n = 25) matched by race, nulliparity, age, and fetal sex from a prospective pregnancy cohort. Log_2_ transformed batch‐normalized data were analyzed by two‐way ANOVA (p < 0.05) with calculation of false discovery rates (*q* < 0.1).

**Results**: Demographic characteristics were similar between groups (Table 1). Amnion from PPROM had higher abundance of eicosanoid, endocannabinoid, phosphatidylcholine, phosphatidylethanolamine, and fatty acid metabolites compared to term (Table 2). Amnion and chorion from PPROM had lower abundance of sex steroid metabolites, including pregnenolone, progestin, estrogenic, and androgenic metabolites compared to term (Table 2).

**Conclusion**: Our study is the first to report a fetal membrane metabolome. We detect evidence of increased metabolism in the arachidonic acid/phospholipid pathway and relative sex steroid deficiency in fetal membranes that rupture preterm. Whether differences in sex steroid metabolites are attributable to PPROM pathophysiology versus advancing gestational age warrants future investigation. Hormonal therapeutics, including local progesterone repletion, may carry potential to mitigate inflammation and fortify membrane integrity, thus reducing preterm birth risk. University of Pennsylvania Research Foundation Award (KG)

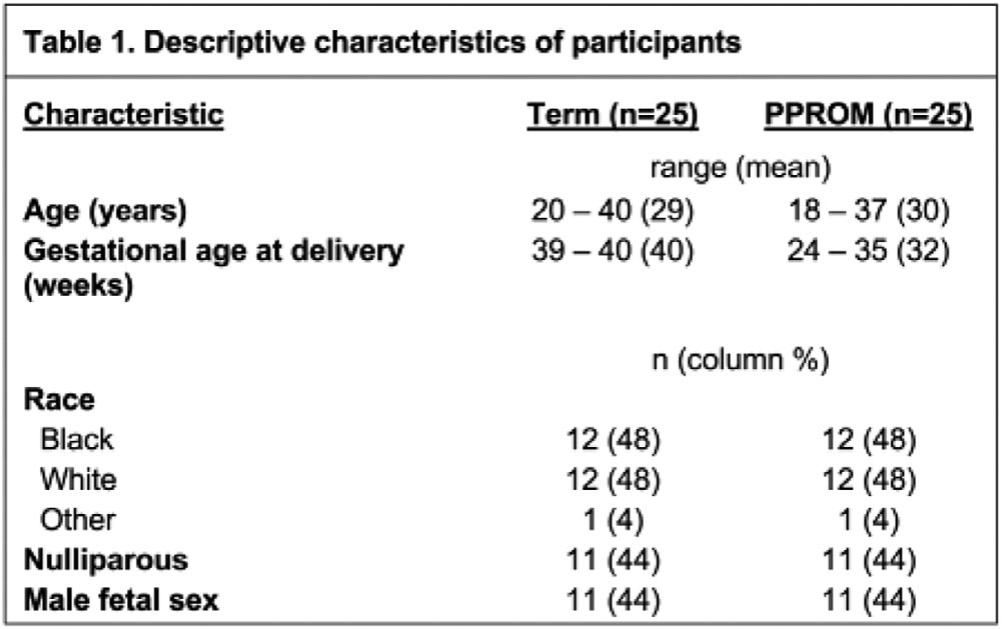

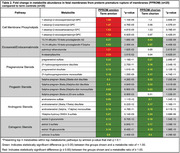

## 810 | Vaginal Polyamines Modify Gardnerella Vaginalis‐Induced Alterations in the Mucosal Transcriptome

Lauren Anton; Briana Ferguson; Aaron Loder; Kristin D. Gerson

*University of Pennsylvania Perelman School of Medicine, Philadelphia, PA*

10:30 AM ‐ 12:30 PM

**Objective**: Anaerobe‐dominant cervicovaginal (CV) microbial communities increase risk of preterm birth. Mucus production, a key part of the host defense system, protects the CV epithelial barrier against anaerobes, including *Gardnerella vaginalis*(GV). Vaginal anaerobes modify mucus properties though molecular mechanisms remain unclear. We previously showed that vaginal metabolites, specifically polyamines, regulate GV‐induced host immune responses. This study aimed to 1) elucidate effects of GV on genes involved in mucus biosynthesis, and 2) determine whether polyamines modify GV‐altered mucosal gene expression.

**Study Design**: Human ectocervical (Ecto), endocervical (Endo) and vaginal (VK2) epithelial cells were treated for 4h +/‐ polyamines, spermine (SPM, 400uM) or putrescine (PUT, 4mM) prior to GV (10^7^ CFU/well) exposure for 24h (n = 3/treatment). RNA‐sequencing was performed. Data were analyzed using DESeq2 to study mucus genes of interest. For genes altered by GV, analysis of polyamine exposure was performed by one‐way ANOVA with Tukey's test for multiple comparisons.

**Results**: Among the 40 mucus‐associated genes studied, GV modified expression of 10 genes in Ectos, 13 genes in Endos, and 6 genes in VK2s (p< 0.05 for all). Genes clustered into functional pathways including mucins, ion channels, protein disulfide isomerases, glycosyl‐transferases, and mucus modifying enzymes (select pathways shown for Ecto in Fig. 1 and Endo in Fig 2.). Among genes altered by GV, SPM partially mitigated these effects in select cells for TMEM38A, SLC12A6, PDIA3, PDIA4, PDIA6, GALNT5, SLPI and NEU1, while PUT potentiated these effects in select cells for TMEM38A, SLC12A6, SLC26A9, PACC1, SLPI, NEU1 (p< 0.05 for all).

**Conclusion**: GV modifies expression of genes in the CV epithelium responsible for increased mucus hydration, formation, and breakdown (sialidases). These alterations in the mucosal transcriptome likely compromise mucus properties, rendering mucus thinner and more penetrable. SPM confers protection against some of these GV‐induced effects and may carry potential as a postbiotic therapeutic to restore mucus function.

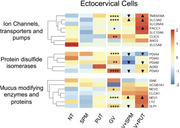

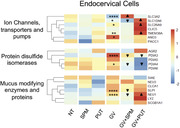

## 811 | Association of Prenatal Care Attendance on Perinatal Outcomes with Amphetamine Exposure

Kristin C. Prewitt; Alyssa R. Hersh; Bharti Garg; Bradley M. Buchheit; Ximena A. Levander; Aaron B. Caughey

*Oregon Health & Science University, Portland, OR*

10:30 AM ‐ 12:30 PM

**Objective**: Prenatal amphetamine use is known to be associated with increased risk of pregnancy‐related morbidity and mortality. Pregnancy can make it difficult to access treatment leading to more limited prenatal care. The purpose of this study is to assess how the number of prenatal care visits impacts perinatal outcomes with amphetamine‐related exposure.

**Study Design**: In this retrospective cohort study of singleton, non‐anomalous births between gestational ages 23‐42 weeks in California (2008‐2020), we assessed the association between amphetamine use identified using ICD‐9/10 codes and a variety of perinatal outcomes. We divided patients into two groupings based on WHO recommendations for minimum number of visits to impact perinatal outcomes ‐ 1) more prenatal care (> = 5 visits) or 2) less prenatal care (< 5 visits). Chi‐squared and multivariable logistic regression were utilized for statistical analyses.

**Results**: There were 21,827 pregnant persons with an amphetamine‐related diagnosis that met our inclusion criteria, with the majority attending more prenatal care (64.2%). Those with less prenatal care were more likely to have placental abruption (3.3% vs 6.5%, aOR 1.32, 95% CI: 1.21‐1.44), preterm premature rupture of membranes (4.1% vs 8.3%; aOR 2.08, 95% CI: 1.83‐2.37), preterm delivery < 37 weeks (16.8% vs 34.0%; aOR 2.45, 95% CI: 2.28‐2.63), and severe maternal morbidity (3.7% vs 7.2%; aOR 1.97, 95% CI: 1.72‐2.25). Fewer visits were associated with worse neonatal outcomes, including respiratory distress (2.2% vs 3.8%; aOR 1.82, 95% CI: 1.52‐2.17), neonatal withdrawal (2.0% vs 6.43%; aOR 1.85, 95% CI: 1.60‐2.14), and NICU admission (24.9% vs 40.4%; aOR 1.89, 95% CI: 1.77‐2.02).

**Conclusion**: Our findings demonstrate an association with adverse perinatal outcomes in individuals with amphetamine‐related diagnoses who attend fewer prenatal care visits. Our results call for efforts to improve prenatal care access in individuals with amphetamine use.

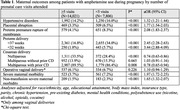

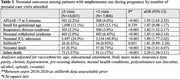

## 812 | Amniotic Fluid Volume and Perinatal Outcomes in Women with Preeclampsia in a Low Resource Country

Kwaku A. Doffour‐Dapaah^1^; Alim Swarray‐Deen^2^; Alex Boateng^3^; Jeff Osei‐Agyapong^1^; Perez Sepenu^4^; Patrick A. Anum^5^; Jerry Coleman^4^

*^1^Greater Accra Regional Hospital, Accra, Greater Accra; ^2^University of Ghana Medical School, University of Ghana Medical School, Greater Accra; ^3^The Trust Hospital, Accra, Greater Accra; ^4^Korle Bu Teaching Hospital, Accra, Greater Accra; ^5^Korle Bu Teaching Hosptal, Accra, Greater Accra*

10:30 AM ‐ 12:30 PM

**Objective**: To determine:

1. The prevalence of oligohydramnios in women with preeclampsia

2. The adverse perinatal outcomes associated with preeclampsia

3. The association between oligohydramnios and preeclampsia disease severity

4. The association between oligohydramnios and adverse perinatal outcomes in women with preeclampsia

**Study Design**: A cross‐sectional study at the Korle Bu Teaching Hospital which assessed a total of 130 pregnant women from 28 weeks to 42 weeks gestation: 10 had preeclampsia and oligohydraamnios on one arm, and the rest had preeclampsia but no oligohydramnios on the other arm. All participants had their amniotic fluid index assessed by ultrasound. This assessment was repeated weekly and the last measurement taken before delivery was used for the analysis of amniotic fluid volume data. The perinatal outcomes were also recorded.

**Results**: The prevalence of oligohydramnios in women with preeclampsia was 7.7%. Adverse perinatal outcomes associated with preeclampsia were preterm delivery(50%), stillbirth(10%), poor APGAR scores at one and five minutes (36.1% and 17.7%), low birth weight (46.1%), and NICU admission (40.0%). There was no association between pre‐eclampsia disease severity and oligohydramnios in women with preeclampsia and there was no association between oligohydramnios and adverse perinatal outcomes in women with preeclampsia. Male sex (p = 0.02), gestational age at delivery (p = 0.010) and birth weight (p = 0.042) were the factors associated with adverse perinatal outcomes in women with preeclampsia.

**Conclusion**: Adverse perinatal outcomes associated with preeclampsia were preterm birth, stillbirth, poor APGAR scores, low birth weight and NICU admission. The lack of association between oligohydramnios and adverse perinatal outcomes; as well as the lack of association between oligohydramnios and severe preeclampsia suggests that it may not be beneficial for clinicians in low resource countries to use amniotic fluid volume as a marker for determining when preeclampsia is severe enough to warrant delivery or less severe enough to allow conservative management

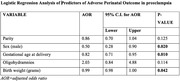

## 813 | Abdominal Circumference versus Estimated Fetal Weight in Fetal Growth Restriction Surveillance and Outcomes

Lama R. Noureddine^1^; Robyn D'Agostino^1^; Vanessa Hwu^1^; Sarah Stavros^1^; Jessica Greenberg^2^; Joseph J. Apuzzio^1^; Shauna F. Williams^1^

*^1^Rutgers New Jersey Medical School, Newark, NJ; ^2^Rutgers New Jersey Medical School, South Orange, NJ*

10:30 AM ‐ 12:30 PM

**Objective**: To compare the incidence of abnormal umbilical artery Doppler (UAD), obstetric and neonatal outcomes in pregnancies affected by fetal growth restriction (FGR) when diagnosed by abdominal circumference (AC) and estimated fetal weight (EFW).

**Study Design**: This is a retrospective cohort study of patients with a singleton pregnancy with FGR who delivered at a tertiary urban academic center between January 2020 and December 2023.The primary outcome was abnormal UAD. Secondary outcomes were obstetric outcomes and Composite Neonatal Outcomes (CNO) including RDS, IVH, ROP, NEC, HIE, TTN, neonatal seizures, sepsis, hypoglycemia. Fisher's exact, chi square, parametric and non‐parametric tests were used.

**Results**: Among 178 patients, 104 (58%) were diagnosed by AC only (Group 1) and 74 (42%) by EFW (Group 2). Abnormal UAD was more frequent in patients with FGR diagnosed by EFW [3(3%) vs 11(15%) in Groups 1 and 2 respectively, p = 0.004]. Gestational age at FGR diagnosis was significantly lower in Group 1 (32w4d vs 35w6d in Groups 1 and 2 respectively, p = 0.008). The incidence of cesarean delivery was similar between the groups[47(37%) vs 30(45%) in Groups 1 and 2 respectively, p = 0.35]. The median GA at delivery was lower in Group 2 [39w0d in Group 1 vs 38w1d in Group 2, p< 0.0001]. Resolution of FGR was more likely when initially diagnosed by AC only [27 (21%) vs 1(1%) in Groups 1 and 2 respectively, p< 0.0001]. Neonatal weight was lower in group 2 (median 2705g vs 2405g in Groups 1 and 2 respectively, p< 0.0001); and incidence of small for gestational age (SGA) and low birth weight (LBW) were higher in group 2 [SGA: 41(39%) vs 51(69%), p = 0.0001;  LBW: 27(26%) vs 50(68%), p< 0.0001, respectively]. Incidence of CNO was higher in Group 2 [18(17%) vs 23(31%), p = 0.04].  

**Conclusion**: Abnormal UAD was rarely seen in FGR fetuses diagnosed by AC and adverse neonatal outcomes were also seen less frequently in this group. FGR fetuses diagnosed by AC should be followed closely, although different surveillance frequency could be considered.

## 814 | The Association Between Diabetes Mellitus During Pregnancy and Retinopathy of Prematurity

Lara Saaida^1^; Victor Novack^2^; Ahed Imtirat^2^; Eilon shani^2^; Tamar Eshkoli^2^

*^1^Haemek medical center, haemek medical center, HaZafon; ^2^Soroka University Medical Center, Soroka University Medical Center, HaDarom*

10:30 AM ‐ 12:30 PM

**Objective**: We aimed to evaluate the association between diabetes mellitus (DM) during pregnancy and retinopathy of prematurity (ROP) in preterm infants younger than 32 gestational weeks or infants with low birthweight (< 1500 grams).

**Study Design**: We conducted a retrospective nested case‐control study of all premature infants who were born alive and survived the post‐delivery hospitalization period in Soroka Medical Center, with either gestational age younger than 32 weeks or birthweight less than 1,500 grams, during the years 2013‐2021. The infants were divided into two groups according to ROP status. Multivariable logistic regression was used to analyze the association between ROP and DM.

**Results**: During the study period, there were 881 pairs of women and newborns who met the inclusion criteria. The ROP group included 345 infants (39.1%). 22 (6.4%) of the mothers in the ROP group were diagnosed with DM during pregnancy compared with 52 of 536 (9.7%) in the control group (P = 0.082). ROP was associated with oxygen treatment (OR 1.05; 95% CI, 1.03‐1.08; P< .001), birthweight < 1250 g` (OR 2.70; 95% CI, 1.93‐3.80; P< 0.001) and advanced maternal age (OR 1.03; 95% CI, 1.01‐1.06; P = .01). Steroid treatment (OR 0.73; 95% CI, 0.60‐0.89; P = .002) and advanced gestational age (OR 0.92; 95% CI, 0.85‐1.00; P = 0.05) were found as protective factors for ROP. Using multivariable logistic regression analysis and adjusting for multiple risk factors, it was found that there is no significant association between maternal DM and the development of ROP.

**Conclusion**: The presence of maternal diabetes mellitus does not increase the likelihood of ROP in pre‐term infants.

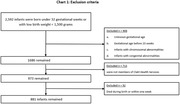

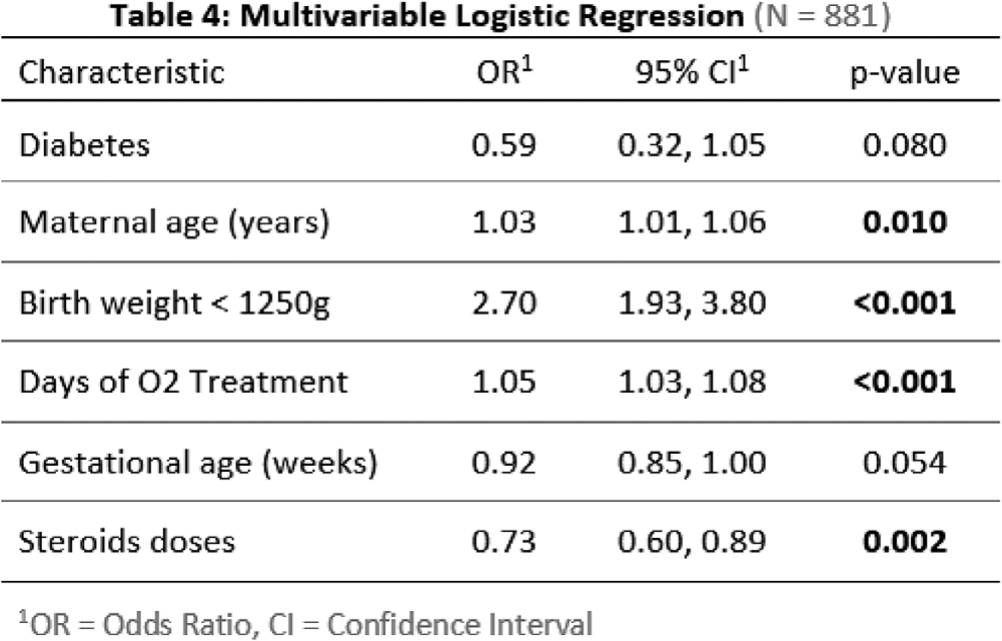

## 815 | Cost‐Effectiveness of Atosiban Versus Placebo in the Treatment of Threatened Preterm Birth (APOSTEL 8 TRIAL)

Larissa I. van der Windt^1^; Martijn A. Oudijk^1^; Job Klumper^1^; Ruben G. Duijnhoven^1^; Joris A. M. van der Post^2^; Carolien Roos^1^; Stavros Petrou^3^; Kate F. Walker^4^

*^1^Amsterdam UMC, location University of Amsterdam, Amsterdam, Noord‐Holland; ^2^Amsterdam UMC, location University of Amsterdam, Amsterdam, Noord‐Brabant; ^3^Nuffield Department of Primary Care Health Sciences, University of Oxford, Oxford, England; ^4^Centre of Perinatal Research University of Nottingham, Queen's Medical Centre, Dublin, England*

10:30 AM ‐ 12:30 PM

**Objective**: Tocolytics such as atosiban as treatment for threatened preterm birth (PTB) is part of routine care in many countries. However, overall costs and benefits of atosiban compared to placebo have not been evaluated. We assessed the cost‐effectiveness of atosiban compared to placebo in improving neonatal outcomes for threatened PTB between 30 and 34 weeks gestation.

**Study Design**: This was an economic analysis from societal perspective alongside the international randomized placebo controlled APOSTEL 8 study, conducted in 26 hospitals in the Netherlands, England and Ireland. Women with threatened PTB  from 30 to 34 weeks of gestation were randomized to atosiban or placebo. We included costs based on data from the case record form (CRF) and additional iMTA questionnaire sent three months after due date. Primary outcome was the difference in total mean costs per child between groups. Health outcome was the prevalence of composite adverse neonatal outcome. Complementary incremental cost‐effectiveness ratio (ICERs) was calculated. Analyses were by intention to treat.

**Results**: In our complete case analysis, we included costs based on the CRFs of 752 mothers with 884 children and 376 completed questionnaires (198 atosiban, 178 placebo). Total mean costs did not significantly differ between groups: €37280 in the atosiban group (449 children) versus €37120 in the placebo group (435 children) (mean difference €160, 95% CI ‐€3936 to €4257). In pre‐ and postnatal costs no significant differences were observed. With an estimated difference of 1% in adverse neonatal outcome in favour of atosiban, the ICER was €16783. However, there was high uncertainty as a substantial distribution was located in the top left quadrant of the ICER plane, indicating scenarios where atosiban is both more expensive and less effective.

**Conclusion**: We observed high uncertainty of cost‐effectiveness of atosiban compared to placebo as treatment for threatened PTB. As the main trial demonstrated that atosiban is not superior to placebo in improving neonatal outcomes, both clinical and economical findings should be considered in future guidelines.

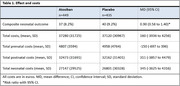

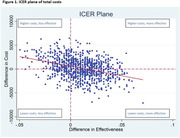

## 816 | Mediation of Socioeconomic Disparities and Preterm Birth by Class‐Based Discrimination and Financial Strain

Lauren S. Keenan‐Devlin^1^; Alexa A. Freedman^2^; Renee Odom‐Konja^3^; Tao Jiang^4^; Janedelie Romero^3^; Linda M. Ernst^3^; Greg E. Miller^2^; Ann EB Borders^5^

*^1^Endeavor Health/ University of Chicago Pritzker School of Medicine, Endeavor Health, IL; ^2^Northwestern University, Chicago, IL; ^3^Endeavor Health, Evanston, IL; ^4^Northwestern University, Northwestern University / Evanston, IL; ^5^Endeavor Health, Evanston Hospital, Evanston, IL*

10:30 AM ‐ 12:30 PM

**Objective**: Various measures of low socioeconomic status (SES) have been associated with elevated preterm birth (PTB) risk. To inform intervention strategies, we sought to identify which SES markers were most predictive of PTB, and identify whether psychosocial factors mediated SES‐related PTB disparities.

**Study Design**: Stress, Pregnancy and Health study participants completed psychosocial and demographic assessments during 2^nd^ and 3^rd^ trimesters. An SES “status” composite was calculated with standardized measures of occupational prestige and highest degree earned in the household; a “financial resources” composite included standardized measures of self‐reported household savings relative to cost of living, household income to poverty ratio, and total savings and assets. Psychosocial mediators included lifetime, daily, and perceived class‐based discrimination (CBD), and subjective financial strain (SFS). PTB was defined as delivery < 37’0 weeks gestation. Multiple regressions tested the association of PTB with the composites and their individual components, as well as potential mediators. Mediation models estimated the proportion of the association mediated by CBD and SFS. Analyses were adjusted for maternal age, race/ethnicity, and marital status.

**Results**: Among 599 participants, about half had the highest educational attainment and IPR. 11.5% delivered preterm. Participants with higher status had lower risk of PTB (RR: 0.72, 95%CI 0.54,0.96), whereas self‐reported financial resources were not associated with PTB (Table 1). In unadjusted models, higher SFS was associated with PTB risk (RR: 1.38, 95%CI 1.13,1.68), but CBD was not. Approximately 20% of the relationship between status and PTB was mediated by SFS, though this was not statistically significant (p = 0.28).

**Conclusion**: Status‐based SES measures were significantly associated with PTB whereas financial resource‐based measures were not. Our results suggest that the experience of being lower SES (i.e. social status/capital and the lived reality that these facilitate) may bear more significantly on pregnancy outcomes than self‐identified monetary resources.

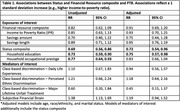

## 817 | A Machine Learning Approach to Intrapartum FHRT Analysis To Identify Neonates Requiring Therapeutic Hypothermia

Lauren M. Silva^1^; Jaber Qezelbash‐Chamak^2^; Dillon Joiner^1^; Minhee Kim^2^; Karen T. Hicklin^3^; Michael D. Weiss^1^; Marie Berg^1^; Cyndi Garvan^1^; John C. Smulian^1^

*^1^University of Florida College of Medicine, Gainesville, FL; ^2^University of Florida Herbert Wertheim College of Engineering, Gainesville, FL; ^3^Icahn School of Medicine at Mount Sinai, New York, NY*

10:30 AM ‐ 12:30 PM

**Objective**: Examine a “sliding window time frame” as a ML approach to fetal heart rate tracing (FHRT) analysis to identify neonates that developed moderate to severe neonatal encephalopathy (NE) and underwent therapeutic hypothermia.

**Study Design**: Retrospective observational case‐control involving neonates born at a single academic institution and admitted to the NICU with cord pHs < 7.15. The study group included neonates meeting both physiologic (low cord pH) and neurologic criteria for moderate to severe NE, making them eligible for therapeutic hypothermia. The control group comprised neonates who, despite having low cord pHs, did not show evidence of moderate or severe NE and thus did not undergo therapeutic hypothermia.

The entire intrapartum FHRT for eligible neonates was analyzed to develop a ML‐based model incorporating an embedded sliding time window. Isolation Forest was used to identify and cluster windows with unusual patterns as anomalies or typical patterns as normal. A Random Forest classifier was then trained to predict abnormal pattern labels. After the optimal sliding time window size was determined, its impact on the ML model's anomaly detection performance was evaluated by calculating the percentage of anomalous time windows during the entire intrapartum course for both subject groups and compared by student t‐test.

**Results**: There were 22 study and 22 control cases with similar clinical characteristics and cord pH values. Using a 90‐minute sliding time window, the ML‐based model identified a significant difference in the proportion of windows with abnormal patterns between the control and study cases (4.7 % vs 73.3%, p < 0.001). The ML classifier applied to these 90‐minute windows achieved 100% accuracy in distinguishing between study and control cases.

**Conclusion**: The ML‐based model incorporating an embedded sliding time window accurately identified neonates with moderate to severe NE. This is a promising approach to intrapartum FHRT assessment with the potential to identify fetuses at risk for neurologic injury and allow for timely intervention.

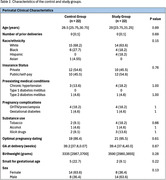

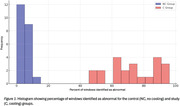

## 818 | The Association Between Excessive Gestational Weight Gain in Obese Patients and Intrapartum and Postpartum Outcomes

Lauren M. Silva^1^; Caitlin Rogers^1^; Emily Astarita^1^; Divya Mahesh^1^; Payton Campbell^1^; Danielle Snyder^1^; Nicole Diaz^1^; Aniela Edwards^1^; Caroline Lamoutte^2^; Rebecca Henderson^3^; Khanh Huynh^1^; Thomas Reilly^1^; Matthew Gurka^4^; Tony Wen^1^; Adetola F. Louis‐Jacques^5^

*^1^University of Florida College of Medicine, Gainesville, FL; ^2^St. Louis University School of Medicine, St. Louis, MO; ^3^University of Alabama School of Medicine, Gainesville, FL; ^4^University of Virginia School of Medicine, Charlottesville, VA; ^5^University of Florida, Gainesville, FL*

10:30 AM ‐ 12:30 PM

**Objective**: Obese women have a longer duration of labor, particularly those undergoing induction of labor (IOL), and higher rates of adverse outcomes. This study aimed to determine the length of the latent phase, total induction time, and frequencies of cesarean delivery and adverse outcomes in obese patients with excessive weight gain (EWG) compared to those with appropriate weight gain (AWG) undergoing IOL.

**Study Design**: Retrospective cohort study including nulliparous patients with a BMI > 30 kg/m2 that underwent IOL at a single academic institution between June 2018 and December 2022. Gestational weight gain was calculated based on the difference between pre‐pregnancy or first trimester weight and weight at delivery, then categorized as AWG or EWG based on recommendations by the Institute of Medicine. The IOL lengths and frequencies of the outcomes of interest (Table 2) were extracted from the electronic medical record. Chi square, t‐test, and Wilcoxon rank sum test were performed where appropriate.

**Results**: Of 469 obese patients, 105 (22.4%) had AWG and 364 (77.6%) had EWG. The median pregravid BMI for patients with AWG was significantly higher than in those with EWG (38.6 vs 35.5 kg/m2, p < 0.001). There were no significant differences in the latent phase lengths, total induction times, or frequencies of cesarean delivery and adverse outcomes.

**Conclusion**: EWG in obese patients undergoing IOL is not associated with increased latent phase lengths, total induction times, or frequencies of cesarean delivery or adverse outcomes. The significantly higher pregravid BMI in the AWG group may have influenced our findings by diminishing the difference in IOL lengths and frequencies of adverse outcomes when compared to the EWG group. Further investigation is necessary to account for this difference in pregravid BMIs and to determine whether EWG is independently associated with higher intrapartum and postpartum adverse outcomes in obese patients undergoing induction of labor.

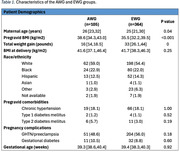

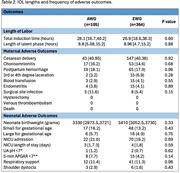

## 819 | Safety of Prostaglandins in the Peripartum Period in Case of Sickle Cell Disease

Mayi GNOFAM^1^; Jeanne SIBIUDE^2^; Louis AFFO^1^; Frederic GALACTEROS^3^; Edouard Lecarpentier^3^; Florence WANG^1^; Marine DRIESSEN^4^; Olivier Graesslin^5^; Gylna LOKO^6^; Giovanna CANNAS^7^; Stéphane Bounan^8^; Véronique Debarge^9^; Caroline Makowski^10^; Axel Fichez^11^; Hervé Fernandez^12^; Laurent Mandelbrot^13^

*^1^Assistance Publique Hôpitaux de Paris, Colombes, Ile‐de‐France; ^2^Assistance Publique Hôpitaux de Paris, Paris, Ile‐de‐France; ^3^Assistance Publique Hôpitaux de Paris, Creteil, Ile‐de‐France; ^4^Assistance Publiqhe Hôpitaux de Paris, Paris, Ile‐de‐France; ^5^CHU de Reims, Reims, Champagne‐Ardenne; ^6^CHU Martinique, Fort de France; ^7^CHU de Lyon, Lyon, Ile‐de‐France; ^8^Centre Hospitalier de Saint Denis, Saint Denis, Ile‐de‐France; ^9^CHU de Lille, Lille, Nord‐Pas‐de‐Calais; ^10^CHU de Grenoble, Grenoble, Rhone‐Alpes; ^11^CHU de Lyon, Lyon, Rhone‐Alpes; ^12^Assistance Publique Hôpitaux de Paris, Kremlin‐Bicêtre, Ile‐de‐France; ^13^Hôpital Louis Mourier, APHP, Université Paris Cité, Colombes, Ile‐de‐France*

10:30 AM ‐ 12:30 PM

**Objective**: To assess the tolerance of peripartum prostaglandin use in women with sickle cell disease (SCD).

**Study Design**: A retrospective cohort study in 10 centers in France. Among all women with SCD (S/S, S/C or S/β‐thalassemia), we compared pregnancies with and without exposure to prostaglandins, overall and specifically according to whether prostaglandins were used for labor induction or for post‐partum hemorrhage (PPH). The main outcome was the occurrence of acute SCD complications, defined as any vaso‐occlusive crisis (VOC) or thromboembolic event, within 7 days after delivery.

**Results**: Prostaglandins were used in 114/411 pregnancies (27.7%). In 106/411 (25.8%), prostaglandins were used to induce labor, with PGE2 (dinoprostone gel or pessary) for 95 patients and misoprostol for 11 patients. The incidence of PPH was 60/411 (14.6%), and 11 cases required second‐line therapy with prostaglandins; sulprostone was used as per French practice guidelines. The incidence of acute complications did not differ between patients exposed and not exposed to prostaglandins (14/114 (12.2%) vs 34/293 (11.6%), respectively, p = 0.87, nor did the incidence of severe VOC (7.0% vs 7.8%, respectively; p = 0.78) or other secondary outcomes. Among patients undergoing labor induction, acute complications occurred in 12/106 (11.6%) exposed to prostaglandins vs 6/77 (7.6%) unexposed (p = 0.46). Among patients with PPH, rates were respectively 2/11 (18.2%) vs 9/49 (18.4%; p = 0.99).

**Conclusion**: The use of prostaglandins in patients with SCD for labor induction or for PPH appeared safe, within the limitations of a retrospective study.

## 820 | Associations Between Non‐Communicable Diseases and Obstetric Complications at a Tertiary Referral Hospital in Southwestern Uganda

Leevan Tibaijuka^1^; Joseph Ngonzi^2^; Jean‐Pierre Van geertruyden^3^; Lisa M. Bebell^4^; Brenda Ainomugisha^2^; Hamson Kanyesigye^2^; Musa Kayondo^2^; Francis Bajunirwe^2^; Yarine Fajardo Tornes^2^; Yves Jacquemyn^5^; Adeline A. Boatin^4^

*^1^Mbarara University of Science and Technology, Mbarara University of Science and Technology, Mbarara; ^2^Mbarara University of Science and Technology, Mbarara, Mbarara; ^3^Global Health Institute, University of Antwerp, Antwerpen; ^4^Massachusetts General Hospital, Massachusetts General Hospital, MA; ^5^University of Antwerp, University of Antwerp, Antwerpen*

10:30 AM ‐ 12:30 PM

**Objective**: Non‐communicable diseases (NCDs) increasingly contribute to maternal morbidity, accounting for 15% of indirect maternal deaths worldwide. We determined the association between NCDs and obstetric complications at Mbarara Regional Referral Hospital (MRRH) in southwestern Uganda.

**Study Design**: We conducted a retrospective cross‐sectional study, and randomly selected records of women admitted for delivery at MRRH each month from January to December 2022, extracting their socio‐demographic and clinical characteristics. We performed multivariable robust Poisson regression analysis to assess the association between NCDs and obstetric complications. Models were adjusted for maternal age, gravidity, referral status, employment status, and HIV serostatus.

**Results**: We abstracted data for 2,336 women with a mean age of 26±5.9 years. At least one NCD was present in 6.4% (n = 149) including anemia (n = 77, 3.3%), chronic hypertension (n = 35, 1.5%), pre‐gestational diabetes (n = 16, 0.7%), asthma (n = 9, 0.4%) and cardiac disease (n = 6, 0.3%). Overall, 542 (23.2%) women had obstetric complications, including pre‐eclampsia (n = 265, 11.3%); preterm labor (n = 67, 2.9%); placental abruption (n = 29, 1.2%); PPH (n = 54, 2.3%); and gestational diabetes (n = 5, 0.2%). Women with NCDs had increased likelihood of having an obstetric complication compared to women without (overall proportion 33.6% vs 22.5% respectively; adjusted prevalence ratio (aPR): overall, 1.8, 95% CI: 1.4‐2.3; pre‐eclampsia (1.8, 95%CI: 1.2‐2.8), gestational diabetes (12.0, 95%CI: 2.0‐72.7), deep venous thrombosis (6.0, 95%CI: 1.3‐27.1), placenta abruption (4.4, 95%CI: 1.5‐12.6) and postpartum hemorrhage (4.3, 95%CI: 2.2‐8.3).

**Conclusion**: We found that NCDs were associated with a nearly two‐fold increased risk of obstetric complications. Our findings highlight the need for further research to understand the impact of this risk, particularly on maternal and fetal outcomes. Additionally, these findings suggest strengthened NCD surveillance, as means to increased preparedness, and management for potential obstetric complications among pregnant women in Uganda.

## 821 | a Comparative Study of Stillbirth Classification Schemes

Lena A. Shay; Mohamad Ali Maktabi; April D. Adams

*Baylor College of Medicine, Houston, TX*

10:30 AM ‐ 12:30 PM

**Objective**: Stillbirth, defined as fetal death at ≥20 weeks’ gestation, complicates ∼1 in 160 pregnancies and causes vary widely. The most helpful tests in determining cause of stillbirth are fetal autopsy, placental pathology, and genetic testing, yet uptake is often low or incomplete. Several methodologies exist to classify stillbirth into phenotypic groups based on pathologic and clinical findings, each varying in complexity and ease of use. Our objective was to determine the agreement between three published classification systems (PASS, INCODE, and ReCoDe) using a cohort of patients who had complete diagnostic evaluation after stillbirth.

**Study Design**: Retrospective cohort study was conducted examining stillbirths occurring at two tertiary care centers from 2016 to 2022 with completed genetic and pathologic evaluation (n = 85). Three independent reviewers examined the cases according to selected classification schemes.  Descriptive statistics were calculated and reliability analysis using Fleiss’ kappa was performed to determine agreement.

**Results**: Leading cause of stillbirth among the classification schemes was placental in origin followed by fetal (µ = 48%, µ = 33%) (Table 1). Each scheme had low rate of unclassified cases (µ = 3%). Agreement between the three classification schemes was fair (κ = 0.38, CI 0.30‐0.46; p< 0.001). 26% had positive genetic findings on diagnostic testing. Sub‐analysis of cases with a genetic diagnosis did not demonstrate increased agreement (κ = 0.33, CI 0.14‐0.53; p< 0.001).

**Conclusion**: Findings demonstrate tested classification schemes perform similarly on most stillbirth cases regarding minimization of unclassified diagnoses, but incompletely agree on all classifications. This highlights the diagnostic imprecision of stillbirth. These classification systems are limited by the user's interpretation of findings as well as failure to account for pathophysiologic interactions in cases affected by multiple clinical, genetic, or pathologic factors. Future work should be aimed at using tools such as machine‐learning to develop classification algorithms to address these limitations.

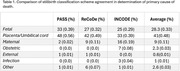

## 822 | Evaluating an Artificial Intelligence Platform Answering Common Pregnancy Questions: Satisfactory but Inconsistent

Leonardo F. Antelo^1^; Lois Brustman^2^; Zainab Al‐Ibraheemi^2^; Graham Ashmead^3^

*^1^Mount Sinai West Obstetrics and Gynecology, New York, NY; ^2^Icahn School of Medicine at Mount Sinai West, New York, NY; ^3^Mount Sinai West, New York, NY*

10:30 AM ‐ 12:30 PM

**Objective**: The availability of artificial intelligence platforms is rapidly increasing. We assessed the platform ChatGPT 3.0 and its ability to answer questions about common pregnancy conditions. This study analyzed the appropriateness of responses, the consistency of responses over multiple iterations, and the presence of misinformation within the responses.

**Study Design**: Seven questions were generated for each of the following pregnancy conditions to yield a total of 28 questions: Gestational Diabetes, Preeclampsia, Fetal Growth Restriction, and Oligohydramnios. Questions were entered into ChatGPT 3.0 in an identical manner three separate times, generating 84 total responses. Since ChatGPT forms a unique response even when exposed to the same prompt, each set of responses was reviewed by one of three board‐certified MFM physicians. The accuracy of responses to each specific question was rated 1) Satisfactory, 2) Incomplete, or 3) Inadequate. ChatGPT responses contain information beyond the scope of the question. This additional information was evaluated for the presence or absence of misinformation by each reviewer.

**Results**: The responses of 13 out of 28 questions received consistent Satisfactory ratings over the three separate iterations (47%). 26 out of 28 questions had at least one response that was satisfactory (93%). When assessing the total 84 responses for misinformation, 22 out of 84 contained misinformation (27%). 51 out of 84 were rated as both Satisfactory and did not contain misinformation (61%). Only 7 out of the 28 questions generated consistent answers by the reviewers, receiving both satisfactory ratings and also not containing misinformation (25%).

**Conclusion**: Our results suggest that Chat GPT 3.0 is able to generate satisfactory responses to common pregnancy questions. However, we found it to be inconsistent over multiple iterations of the same question. This platform should be used with caution, and the presence of misinformation should be noted when counseling patients on the use of AI.

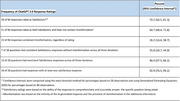

## 823 | Inflammatory Lesions in Placentas of Endometriosis Patients and Their Relation to Maternal and Neonatal Outcomes

Liat Mor^1^; Roni Goldenberg^2^; Ella Toledano^1^; Ran Keidar^1^; Letizia Schreiber^1^; Eran Weiner^3^; Elad Barber^1^

*^1^Edith Wolfson Medical Center, Holon, HaMerkaz; ^2^Tel Aviv University, Faculty of Medicine, Tel aviv, Tel Aviv; ^3^Wolfson Medical Center, Wolfson Medical Center, Tel Aviv*

10:30 AM ‐ 12:30 PM

**Objective**: This study aimed to investigate the relationship between endometriosis and placental pathology, aiming to elucidate potential mechanisms behind adverse pregnancy outcomes associated with endometriosis.

**Study Design**: This retrospective cohort study analyzed data of patients with endometriosis who delivered at a single tertiary center between 2008‐2023. Inclusion criteria were singleton term deliveries with placentas sent for histopathological examination. Maternal characteristics, pregnancy outcomes, and placental histopathology were compared between patients with endometriosis (n = 50) and a control group without endometriosis (n = 150) matched for maternal and gestational age.

**Results**: The demographic characteristics and labor outcomes did not differ significantly between the groups. Patients with endometriosis exhibited higher rates of placental lesions, including placental hemorrhage (10% vs. 2.6%, p = 0.045), maternal inflammatory response lesions (16% vs. 6%, p = 0.039), fetal inflammatory response lesions (8% vs. 1.3%, p = 0.035), chronic villitis (8% vs. 1.3%, p = 0.035), and chronic deciduitis (14% vs. 2.6%, p = 0.006). Moreover, composite adverse neonatal outcomes were significantly higher in the endometriosis group (16% vs. 4%, p = 0.007). Multivariable regression analyses confirmed significant associations between endometriosis and all inflammatory placental lesions, both acute and chronic. Additionally, endometriosis was significantly and independently associated with adverse neonatal outcomes.

**Conclusion**: The presence of endometriosis is associated with increased placental pathology and adverse neonatal outcomes. The findings suggest that inflammatory lesions in the placenta may play a significant role in the pregnancy complications observed in women with endometriosis, highlighting the need for targeted monitoring and management strategies in this population.

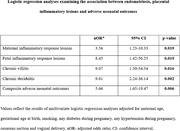

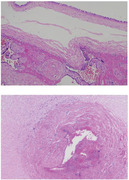

## 824 | Machine Learning‐based Pregnancy and Pregnancy Trimesters Prediction Using Electrocardiogram

Lihong Mo^1^; Shantanu Milind Joshi^2^; Sonul Gupta^1^; Vivian Pae^1^; Ijeoma Uche^1^; Hana Shaik^3^; Chen‐Nee Chuah^3^; Philip Strong^1^; Uma Srivasta^1^; Imo Ebong^1^; Herman L. Hedriana^1^; Elaine Waetjen^1^

*^1^UC Davis Health, Sacramento, CA; ^2^Computer Science, UC Davis, Davis, CA; ^3^Electrical and Computer Engineering, UC Davis, Davis, CA*

10:30 AM ‐ 12:30 PM

**Objective**: Pregnancy is a physiologic high‐volume state. Physiologic electrocardiogram (ECG) changes in pregnancy have been reported but not well characterized. We hypothesize that the physiologic cardiac volume and compliance changes in pregnancy will result in changes in ECG features that can be used to distinguish pregnant from non‐pregnant state. The objective of this study is to establish a machine learning (ML) model to predict pregnant from non‐pregnant state based on ECG.

**Study Design**: This is an analysis of a database that includes 22,034 ECGs on 7,298 individual patients from 18‐50 years of age who were pregnant at least once between 2011 to 2024 in a single tertiary medical center. After excluding patients with pre‐existing cardiac morbidity, along with those who developed hypertensive disorders of pregnancy, 3,581 ECGs were included (53% negative versus 47% positive label). A supervised ML method (Random Forest) was compared to multiple logistic regression to predict pregnancy state based on four ECG features (QRS interval, QTc interval, PR interval, and heart rate).

**Results**: Random forest ML model performs better than multivariate logistic regression in the prediction of pregnancy state (Table). From the decision tree display of Random Forest ML model, higher QTc, higher heart rate, and lower QRS duration were associated with the classification of pregnancy state (Figure).

**Conclusion**: Four features that are commonly reported in clinical assessment of ECG can assist in the prediction of pregnancy state, especially when ML methods are used. By establishing ECG features associated with pregnancy by trimester, we can begin to characterize patterns associated with abnormal pregnancies.

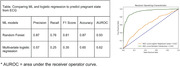

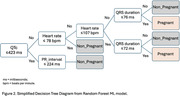

## 825 | Using Supervised Machine Learning to Predict Spontaneous Preterm Birth in Patients Presenting with Preterm Contractions

Vivian Pae; Philip Strong; Herman L. Hedriana; Lihong Mo

*UC Davis Health, Sacramento, CA*

10:30 AM ‐ 12:30 PM

**Objective**: Threatened preterm labor with the occurrence of preterm contractions is the most common reason for antepartum hospital admission, encompassing between 44–59% of all antenatal hospitalizations. Only half of the women who present with TPTL ultimately experience spontaneous preterm birth (sPTB). The nature of preterm contractions and sPTB relationship is not fully understood. The objective of this study was to understand what contributes to sPTB when patients present with preterm contraction symptoms using machine learning (ML).

**Study Design**: A retrospective case‐control study of individuals who experienced preterm contractions prior to sPTBs between January 2022 to March 2024 in a single tertiary medical center. A total of 3,245 patients were manually screened. Two hundred twenty‐four patients who presented with preterm contractions < 36 weeks were included, with 42 delivering > 37 weeks and 182 delivering < 37 weeks. Three supervised ML methods (Random Forest, Support Vector Machine, and k‐Nearest Neighbors) were compared to predict sPTB based on key clinical features (twins versus singleton, gestational age at presentation, body mass index ‐ BMI, smoking history, concurrent PPROM status, history of PTB, and cervical dilation). Missing information in features were imputed from median.

**Results**: All three ML methods achieved promising AUROC in sPTB prediction (Table 1). The top four features related to the sPTB predictions are cervical dilation (measured in centimeters), BMI, gestational age at presentation, and concurrent PPROM status (Table 2).

**Conclusion**: Spontaneous PTB can be predicted via clinical information readily available with ML methods. The key clinical features used in the prediction are similar across all three ML methods used. Single center data and rarity of cases were main limitations. A multicenter or state database should be used to further validate findings.

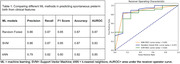

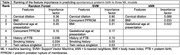

## 826 | Risk Factors for Sexually Transmitted Infection (STI) Partner Management for Pregnant Patients in Southeast Texas

Likhita Nandigam^1^; Sara Vincent^1^; Alison N. Goulding^1^; Irene A. Stafford^2^; Julie Gutierrez^3^; Kaitlyn Stark^4^; Anthony Chartier^5^

*^1^Baylor College of Medicine, Houston, TX; ^2^McGovern Medical School at UTHealth Houston, Houston, TX; ^3^McGovern Medical School at The University of Texas Health Science Center at Houston (UTHealth), Houston, TX; ^4^University of Texas Health Science Center at Houston ‐ McGovern Medical School, Houston, TX; ^5^Department of Obstetrics, Gynecology and Reproductive Sciences, McGovern Medical School at UTHealth, Houston, TX*

10:30 AM ‐ 12:30 PM

**Objective**: Pregnancy provides an opportunity for routine STI testing. However, partner testing and treatment remain challenging, leading to reinfection cycles. Our study sought to evaluate risk factors for incomplete partner treatment for STIs  with and without public health surveillance.

**Study Design**: We performed a retrospective cohort study of all pregnant women who received care at two safety‐net hospitals in Harris County, TX between 2019–2022. Record review was performed for all patients who tested positive for chlamydia, gonorrhea, hepatitis B, and/or syphilis during pregnancy. If any gaps were identified in documentation, patients were contacted for a brief interview about partner(s). Reasons for incomplete partner treatment were stratified into lack of education, lack of contact with patient, financial or other barriers to treatment, tested negative, or treatment status unknown. Partner treatment for hepatitis B and syphilis was confirmed with the regional health department. Differences in categorical variables between groups were examined using chi‐square test or Fisher's exact test. P< 0.05 was considered statistically significant.

**Results**: Of 20,108 pregnant patients who received care between 2019‐2022, 369 patients met inclusion criteria (Table 1). Partner treatment was most successful for patients with chlamydia (53.6%) and gonorrhea (45.5%), followed by syphilis (43.4%) and hepatitis B (1.2%) (Table 2). Lack of ongoing contact was the most common reason for insufficient partner treatment in patients with chlamydia (43.1%) or gonorrhea (75.0%). For syphilis and hepatitis B, the predominant reason remains unknown (42.9%, 72.5%, respectively).

**Conclusion**: While reasons for partner treatment are well‐documented for clinician‐monitored STIs, partner STI status remains relatively unknown for syphilis and hepatitis B. Because these infections are subject to public health surveillance, there may be an overreliance on public health authorities for managing these STIs. Increased education for providers for all STIs should be prioritized, and existing gaps in this area should be addressed.

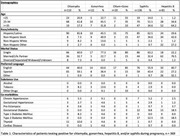

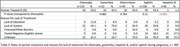

## 827 | Anemia Recovery After Pyelonephritis

Lisa R. Thiele^1^; Sara I. Jones^2^; Sainath Kondapaneni^1^; Jessica E. Pruszynski^1^; Elaine L. Duryea^3^; Catherine Y. Spong^3^

*^1^University of Texas Southwestern, Dallas, TX; ^2^Duke Univeristy, Durham, NC; ^3^University of Texas Southwestern Medical Center, Dallas, TX*

10:30 AM ‐ 12:30 PM

**Objective**: While pregnant patients with pyelonephritis commonly develop anemia secondary to endotoxin‐induced hemolysis, the rate of hematologic recovery is not known. This study aimed to detail the hematologic trajectory during and after antepartum pyelonephritis.

**Study Design**: Patients with antepartum admissions for pyelonephritis between January 1, 2022 and December 1, 2022 who delivered at our institution at least 7 days following admission for pyelonephritis were included. The primary outcome was change in hematocrit (Hct) from its nadir during admission for pyelonephritis to follow‐up. Data collected included hematologic data, patient demographics, medical comorbidities, and characteristics regarding pyelonephritis and delivery admissions. Statistical analysis included t‐tests, Kruskal‐Wallis, and chi‐square tests.

**Results**: Of 78 patients meeting inclusion criteria, the majority were nulliparous, of Hispanic ethnicity, and had an average age of 25.4. During pyelonephritis admissions, the average admission Hct was 33.2% (32.0‐35.2) with an average nadir of 28.8% (26.7‐30.6) occurring on hospital day 2 (1‐3). 60% of patients had anemia at discharge from pyelonephritis admission. The mean rise in Hct from pyelonephritis admission nadir to first follow‐up was 6.0+/‐3.3% with an average rise of 1.63+/‐2.4% per week (Figure).  6% of patients had anemia at delivery admission, with an average admission Hct of 35.9% (33.8‐38.2). Patients with a greater Hct rise were found to have a lower mean nadir, more rapid rise, and higher Hct at delivery admission (p < 0.001), with no difference in initial Hct at pyelonephritis admission.

**Conclusion**: Following pyelonephritis infection in pregnancy, Hct levels steadily rise by an average of 1.63% per week, resulting in resolution of anemia prior to delivery. Patients with lower Hct during pyelonephritis admission demonstrate faster hematologic recovery and are less likely to be anemic at presentation for delivery.

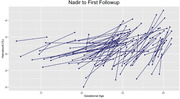

## 828 | Postlaser persistent polyhydramnios after resolution of Twin to Twin Transfusion Syndrome

Mackenzie J. Simon‐Collins^1^; Camille Shantz^2^; Rachana Tanksali^3^; Maryn Day^3^; Julie Alan^1^; Sarah Millard^1^; Michelle Kush^1^; Jena L. Miller^1^; Ahmet A. Baschat^1^; Mara Rosner^1^

*^1^Johns Hopkins Medicine, Baltimore, MD; ^2^Johns Hopkins University, Baltmore, MD; ^3^Johns Hopkins University, Baltimore, MD*

10:30 AM ‐ 12:30 PM

**Objective**: Fluid abnormalities should resolve after fetoscopic laser surgery (FLS) for Twin to Twin Transfusion Syndrome (TTTS), but polyhydramnios occasionally persists. We sought to compare patients with persistent polyhydramnios (PP) to those with resolved polyhydramnios (RP) after FLS for TTTS.

**Study Design**: Single‐center retrospective review of monochorionic twins who underwent FLS for TTTS from 6/2014 to 1/2024. Patients with PP had a maximum vertical pocket (MVP) > 8 cm in the former TTTS recipient  > 14 days after laser. RP patients had resolution of polyhydramnios < 14 days after laser. High order multiples and those with TTTS recurrence were excluded. Pre‐treatment characteristics and birth outcomes were compared between PP and RP groups via Pearson or Fischer's Exact Chi Squared and Mann‐Whitney U. ROC analysis was performed to explore the relationship between pre‐laser MVP and PP.

**Results**: 377/405 (93%) patients had RP after laser and 28 (7%) patients had PP (Figure 1). PP recipients had higher prelaser MVP (11.3 vs 10.1 cm, p = 0.013), higher amnioreduction volumes (1900 vs 1500 cc, p = 0.034) and higher post operative day 1 MVPs (7.4 vs 6.0 cc, p = 0.010) (Table 1). An ROC analysis demonstrated that a prelaser recipient MVP of 11.0 cm predicted PP with 64% sensitivity and 64% specificity. Model quality Gini Index 0.53, area under the curve 0.64 (95% CI: 0.538‐0.753), p = 0.014. Remaining pre‐treatment and intraoperative findings were not different between groups, though there was a trend towards more pyelectasis in PP recipients (10.7 vs 2.7%, p = 0.053). Average time to resolution of polyhydramnios was 35 days in the PP group, versus 3 days in RP (p< 0.001), and was documented in 95% of cases. Gestational age at delivery, birth survival, and birthweight discordance were not different between groups.

**Conclusion**: Persistent polyhydramnios occurred in 7% of patients after laser for TTTS, but ultimately resolved in 95% and was not associated with adverse obstetric outcomes. A trend towards more pyelectasis in PP recipients suggests persistent polyuria as a potential etiology of PP.

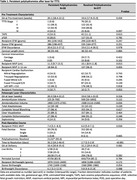

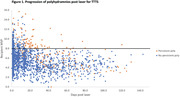

## 829 | Targeted Regulation of Abortion Providers and Infant Mortality

Madeline F. Perry; Joe M. Feinglass; Lynn M. Yee

*Northwestern University Feinberg School of Medicine, Chicago, IL*

10:30 AM ‐ 12:30 PM

**Objective**: Targeted regulation of abortion providers (TRAP laws) are medically unindicated restrictions limiting the provision of abortion, even in states where abortion is legal. Restrictions on reproductive rights have been associated with adverse maternal and neonatal outcomes. We aimed to evaluate the association of the existence of TRAP laws in a state and state rates of infant mortality.

**Study Design**: This cross‐sectional analysis was performed utilizing 2016‐2021 Phase 8 data from the Pregnancy Risk Assessment Monitoring System (PRAMS). PRAMS data were linked with Guttmacher Institute data about state TRAP laws from 2016‐2021. Infant death rates were calculated using PRAMS data, which uses respondent self‐report. In the PRAMS questionnaire, participants are asked “Is your baby alive now?” If participants answer “no,” they are coded as having an infant death. Multivariable Poisson regression testing the association between the existence of TRAP laws in a state and infant mortality was performed, adjusting for individual‐level sociodemographic factors from PRAMS.

**Results**: Among 183,849 participants, representing a weighted population of 9,792,533, 45.2% people lived in states (n = 18) that had at least one TRAP law during the study period (Figure). During the 2016‐2021 study period, 0.4% of participants reported an infant death. In adjusted analysis, there was a 29% increased likelihood of infant mortality (aIRR 1.29, 95% CI 1.08, 1.55) in states with TRAP laws in comparison to those without TRAP laws (Table).

**Conclusion**: TRAP laws are associated with an increased likelihood of infant mortality, even after accounting for individual‐level confounders. Restricted access to abortion may result in people with high‐risk maternal or fetal health conditions to continue pregnancy rather than receive desired abortion services.

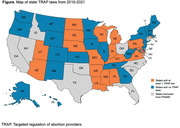

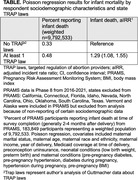

## 830 | Targeted Regulation of Abortion Providers and Intimate Partner Violence Before and During Pregnancy

Madeline F. Perry; Joe M. Feinglass; Lynn M. Yee

*Northwestern University Feinberg School of Medicine, Chicago, IL*

10:30 AM ‐ 12:30 PM

**Objective**: Targeted regulation of abortion providers (TRAP) laws have been associated with an increased rate of intimate partner violence (IPV)‐related homicide. Pregnancy is a uniquely dangerous time for people who experience IPV, yet it is unclear whether abortion restriction on a state level is associated with IPV risk before or during pregnancy. Our objective was to evaluate the association between state‐level TRAP laws and IPV before and during pregnancy.

**Study Design**: We performed a cross‐sectional analysis using data from the Pregnancy Risk Assessment Monitoring System (PRAMS) from Phase 8, 2016‐2021. PRAMS data were linked to Guttmacher Institute data about TRAP laws from 2016 to 2021. PRAMS asks participants if they were “push[ed], hit, slap[ped], kick[ed], choke[d], or physically hurt in any way?” by their current or ex‐partner/husband either in the 12 months before or during pregnancy. IPV before and during pregnancy was defined as answering “yes” to that question. Multivariable Poisson regression analyzing the association between state‐level TRAP laws and state percent IPV before and during pregnancy was performed, controlling for individual sociodemographic factors.

**Results**: In this analysis of 196,451 participants representing a weighted population of 10,267,609 people, 2.5% and 1.7% reported experiencing IPV before and during pregnancy, respectively. Forty‐five percent of PRAMS participants lived in states with at least one TRAP law during the study period (Figure). Multivariable Poisson regression found that participants living in states with TRAP laws had a 14% increased likelihood of IPV before pregnancy and 21% increased likelihood of IPV during pregnancy compared with participants living in states that do not have TRAP laws (aIRR 1.14, 95% CI 1.05, 1.25; aIRR 1.21, 95% CI 1.10, 1.35, respectively; Table).

**Conclusion**: State restriction to abortion access through TRAP laws is associated with increased rates of reported IPV before and during pregnancy. Further research evaluating how state policies have the potential to both exacerbate and mitigate IPV and maternal health are warranted.

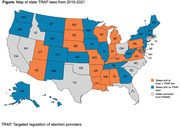

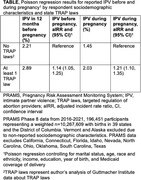

## 831 | Quality Improvement Initiative for Respiratory Syncytial Virus Vaccination In Pregnancy‐ How Can We Do Better?

Madeline Suppiger^1^; Michelle R. Petrich^2^; Miriam Rivkin^3^; Ruizhi Huang^4^; Andrew Storm^5^; Niraj R. Chavan^5^

*^1^Saint Louis University School of Medicine, Webster Groves, MO; ^2^Saint Louis University School of Medicine, Saint Louis University / St. Louis, MO; ^3^St. Louis University, Saint Louis University School of Medicine/ St. Louis, MO; ^4^St. Louis University, St. Louis, MO; ^5^Saint Louis University School of Medicine, St. Louis, MO*

10:30 AM ‐ 12:30 PM

**Objective**: This study was undertaken to evaluate patterns of perinatal respiratory syncytial virus (RSV) vaccination and to identify strategies to increase vaccine uptake through an institutional quality improvement (QI) initiative.

**Study Design**: We evaluated rates of perinatal RSV vaccination during the most recent season of vaccine eligibility from October 2023 to February 2024 among patients seeking prenatal care between 32 to 36 weeks gestation at a single academic tertiary medical center. Maternal demographic characteristics, gestational age at vaccination, and number of prenatal visits during eligibility period were abstracted from electronic records and compared across patients who accepted and refused vaccination. Student's t test and chi2 tests were used to compare continuous and categorical data, respectively. We then administered a structured survey at the end of the RSV season among patients who were previously eligible for vaccination to examine reasons for vaccine hesitancy and identify strategies for enhancing vaccine education.

**Results**: Of 548 eligible patients, 200 (36%) accepted RSV vaccination while 348 (63.5%) did not. Unvaccinated patients were more likely to be Black (p = .002), have public insurance (p = .007) and attend fewer prenatal visits during eligibility period (p< .001). Evaluation of vaccination patterns demonstrated increase in vaccination rates throughout the RSV season with highest rates attained at earlier gestational ages (Figures 1 and 2). Of the 278 patients compliant through postpartum follow up–90 completed the survey (response rate = 32.3%). Primary reasons for vaccine hesitancy were identified as–lack of understanding about vaccine efficacy (40%) and concerns about fetal (30%) and maternal (30%) side effects. Respondents identified handouts (44.1%) and text‐based messaging (37.3%) as their preferred approach for receiving vaccine education.

**Conclusion**: Vaccine education using text‐based messaging and handouts with content focused on the clinical benefit and safety of vaccination has the potential to address RSV vaccine hesitancy and enhance perinatal vaccination.

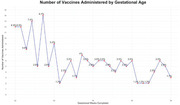

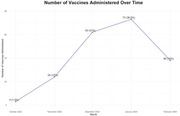

## 832 | Roll‐Over Test as a Screening Tool for Hypertension in Pregnancy ‐ A Prospective Pilot Study

Madhurima K. Keerthy; Vivek Katukuri; Christina Yarrington; Christopher Cox; Conrad Chao; Chloe Fournier‐Hall; Mingma Sherpa

*University of New Mexico, Albuquerque, NM*

10:30 AM ‐ 12:30 PM

**Objective**: Pre‐eclampsia occurs in 3‐8% of pregnant patients globally and is more detrimental to rural communities with limited access to care. Early diagnosis and management improve maternal and neonatal outcomes. The screening modalities that are available include mean Blood pressures, uterine artery Dopplers and various angiogenic markers. However, these are not available to rural areas consistently. The aim of our study to evaluate the potential of the low‐cost Roll‐Over Test to predict hypertensive disease of pregnancies in rural populations.

**Study Design**: This is a single center pilot prospective cohort study. 56 patients were recruited. Demographic data, Uterine artery Doppler and blood for sflt1/Plgf ratio was obtained between 19wk0d–24w6d. Roll‐over Test was conducted by obtaining blood pressures in left lateral and Supine positions, mean change in BP was collected. T‐test (continuous) and Chi Square test(categorical) were used for demographic variables.

Receiver Operating Characteristic (ROC) curves generated for ROT change in SBP, DBP and HR for prediction of primary outcome.  ROC curves generated for UA PI, First SBP (Initial Systolic BP in pregnancy) and First DBP (Initial Diastolic BP in pregnancy).

Sensitivity and specificity calculated for ROT, Ut PI, First SBP and First DBP.

**Results**: 14 (26.9%) had primary outcome. 5 (9%) had preeclampsia and 9 (17%) had gestational hypertension. Of the 5, one (1.9%) had HELLP syndrome. A 17‐point change in systolic BP on ROT had an optimal sensitivity of 60% and specificity of 80% in diagnosing the primary outcome. A mean diastolic change of 9.6 points was identified to provide a sensitivity of 85% and a specificity of 55%. In this population, Uterine artery Dopplers and angiogenic markers did not perform well in predicting primary outcome.

**Conclusion**: The low‐cost Roll‐Over Test is a reasonable screening tool to predict hypertensive disease in pregnancy in rural settings.

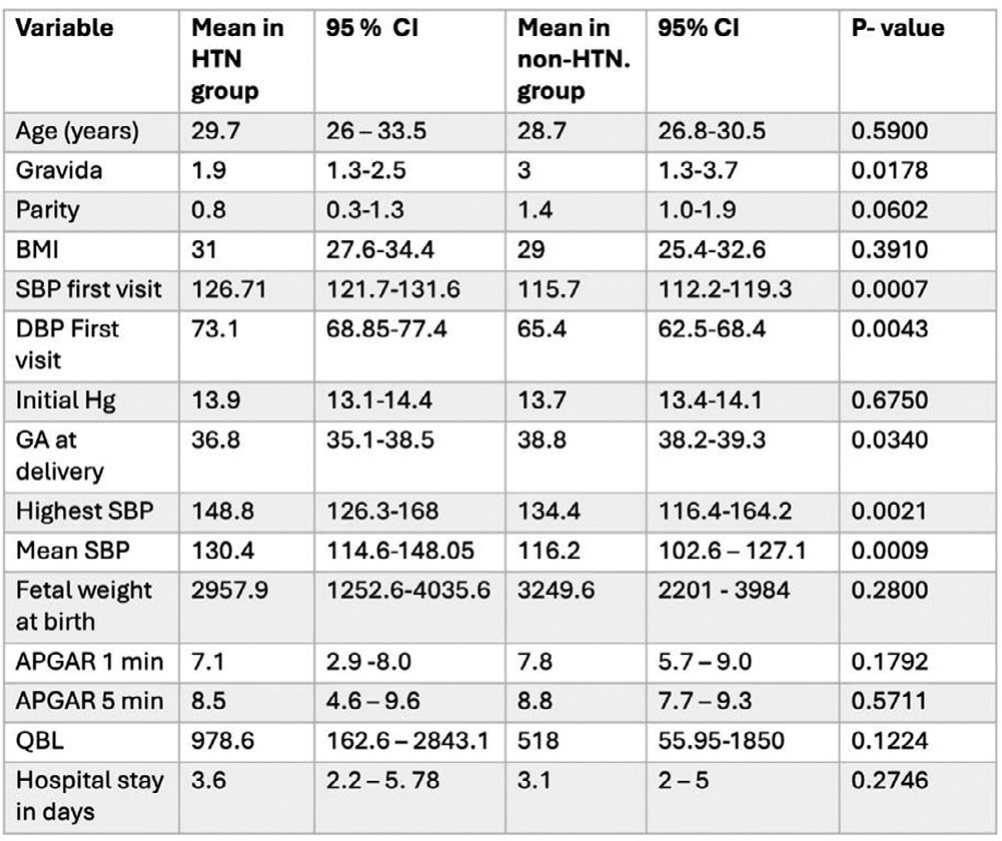

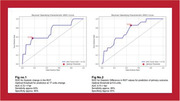

## 833 | Association of Inter‐Twin Growth Discordance >25% with Fetosocpic Laser Surgery Outcomes in Twin‐Twin Transfusion Syndrome

Manasa G. Rao^1^; Christy Gandhi^1^; Russell S. Miller^1^; Meghan Angley^1^; Rosalie Ingrassia^1^; Lynn L. Simpson^2^; Noelle Breslin^1^

*^1^Columbia University Medical Center, New York, NY; ^2^Columbia University Irving Medical Center, New York, NY*

10:30 AM ‐ 12:30 PM

**Objective**: Inter‐twin growth discordance is associated with adverse pregnancy outcomes in monochrionic‐diamniotic (MCDA) twin pregnancies with ≥25% considered a cutoff for pathological discordance. We compared outcomes of fetoscopic laser surgery (FLS) for twin‐twin transfusion syndrome based on presence of absence of discordance ≥25%.

**Study Design**: Retrospective review of MCDA twins with TTTS that underwent FLS at a single center from 2009‐22. Pregnancies with pre‐operative inter‐twin discordance ≥25% were compared to those < 25%. Primary outcome was donor twin survival at first sonogram after FLS.

**Results**: 169 MCDA pregnancies underwent FLS and met inclusion criteria. 62 (37%) had discordance ≥25%. Patients with discordance ≥25% were younger (32 ± 5 vs 30 ± 6).  GA at TTTS diagnosis and at FLS were similar between groups. Donor twin selective fetal growth restriction (sFGR) was more likely with discordance ≥25% (74.2% vs 23.4%, p< 0.01). Pre‐FLS Quintero stage differed between the two groups (p = 0.014), with higher likelihood of stage III and IV in the ≥25% group. Donor twin survival was significantly lower with discordance ≥25% at first post‐FLS sonogram (75.8% vs 89.7%, p = 0.02) and at hospital discharge (52.8% vs 73.6%, p = 0.04). After adjusting for age, Quintero stage, and donor FGR, donor twin survival at first sonogram remained lower in patients with discordance ≥25% (p = 0.01). In a subgroup analysis to evaluate discordance in pregnancies with donor sFGR, donor twin survival did not differ at first sonogram post‐FLS (78.3% vs 96.0%, p = 0.08) or at hospital discharge (61.5% vs 73.9%, p = 0.3) between the groups. In a subgroup analysis of normally‐grown twins, donor twin survival did not differ at first sonogram post‐FLS (68.8% vs 87.7%, p = 0.1) but was lower in the ≥25% discordance group at hospital discharge (80.33% vs 45.45%, p = 0.02).

**Conclusion**: Discordance ≥25% was associated with lower donor twin survival after FLS. While some effect may be attributable to co‐existing donor FGR, discordance was associated with survival difference in normally‐grown donor twins.

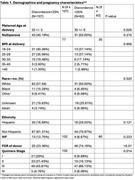

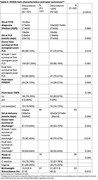

## 834 | Identifying Optimal Discordance Cutoff for Predicting Donor Survival After Fetosocpic Laser for Twin‐Twin Transfusion Syndrome

Manasa G. Rao^1^; Christy Gandhi^1^; Noelle Breslin^1^; Meghan Angley^1^; Rosalie Ingrassia^1^; Lynn L. Simpson^2^; Russell S. Miller^1^

*^1^Columbia University Medical Center, New York, NY; ^2^Columbia University Irving Medical Center, New York, NY*

10:30 AM ‐ 12:30 PM

**Objective**: An optimal inter‐twin discordance cutoff for predicting survival outcomes in monochrionic‐diamniotic (MCDA) twin pregnancies is unclear. This study aimed to evaluate the association of inter‐twin discordance with donor twin survival after fetosopic laser surgery (FLS) for twin‐twin transfusion syndrome (TTTS), and to identify an optimal discordance cutoff.

**Study Design**: Retrospective review of monochorionic diamniotic (MCDA) twins with TTTS that underwent FLS at a single center from 2009‐22. Primary outcome was donor twin survival at first sonogram after FLS. Logistic regression models estimated association between inter‐twin discordance and donor survival at different thresholds. Receiver operating characteristic (ROC) curve analysis was performed to assess predictive accuracy and Youden index was used to determine the optimum cut‐point for predictive accuracy.

**Results**: 169 MCDA pregnancies met inclusion criteria. Discordance cutoffs of ≥25% and ≥30% were significantly associated with donor twin survival at first sonogram post‐FLS (p = 0.03 and p< 0.001, respectively) and this was true after adjusting for donor fetal growth restriction and Quintero stage pre‐FLS. In fitting a logistic regression model, discordance as a continuous variable was significantly associated with predicting donor twin survival (p = 0.02). The AUC for predicting donor twin survival was 0.63 (95% CI: 0.49, 0.74, p = 0.060). Based on the Youden index, the optimum cut point is a discordance of 29%, which has a sensitivity of 0.75, specificity of 0.58, positive predictive value of 0.91 and negative predictive value of 0.29 in predicting donor twin survival.

**Conclusion**: Inter‐twin discordance is a poor predictor of donor twin survival after FLS for TTTS. If it is to be considered in clinical practice, a cutoff value of 29% was most predictive of donor twin survival in this cohort.

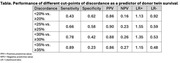

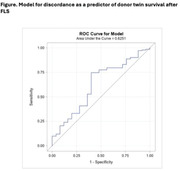

## 835 | Health System Level Intervention to Decrease Early Screening Rates for Gestational Diabetes: Associated Perinatal Outcomes

Mara Greenberg^1^; Yeyi Zhu^2^; Jun Shan^2^; Amanda Ngo^2^; Monique Hedderson^3^; Charles Quesenberry^2^; Assiamira Ferrara^2^

*^1^Kaiser‐Permanente Northern California, Oakland, CA; ^2^Kaiser Permanente Northern California, Oakland, CA; ^3^Kaiser Permanente Northern California, Pleasanton, CA*

10:30 AM ‐ 12:30 PM

**Objective**: To evaluate a system level intervention intended to decrease rates of gestational diabetes (GDM) screening prior to 24 weeks gestation in relation to perinatal outcomes.

**Study Design**: Retrospective study of all pregnancies without overt diabetes delivered in Kaiser Permanente Northern California 2018‐2022. We performed interrupted time series (ITS) analysis to examine changes in rates of GDM screening and perinatal outcomes in relation to a system‐wide intervention to decrease screening < 24 weeks. To reflect the level of exposure to the intervention that started in 4/2020, dates of delivery were categorized in 3 time periods: T1 unexposed (1/2018‐3/2020), T2 partially exposed (4/2020‐12/2020), and T3 fully exposed (1/2021‐12/2022). Primary composite outcome included cesarean delivery, preeclampsia, severe maternal morbidity, large for gestational age neonate, shoulder dystocia, preterm birth, NICU admission, and neonatal hypoglycemia. Patient level factors were examined as potential confounders.

**Results**: Among 221,068 deliveries, early GDM screening rate decreased from 31.1% in T1, to 20.6% in T2 and 4.3% in T3 (Standardized Mean Difference (SMD) T3 vs T1 ‐0.75).  Patient level characteristics did not vary significantly over time, including measures of social drivers of health and comorbid medical conditions. There was no change in the prevalence of GDM (9.7% in both T1 and T2, 9.0% in T3, SMD T3 vs T1 ‐0.02). There was no change in the prevalence of the composite primary outcome, which occurred in 43.1% in T1, 44.2% in T2 and 45.2% in T3 (SMD T3 vs T1 0.04). Interrupted time series analysis adjusted for covariates showed no change in risk of the composite outcome during T1 (percent change per 4 weeks [95% CI]): 0.06 [‐0.01 to 0.13]), T2 (0.02 [‐0.21 to 0.25]), nor T3 (‐0.02 [‐0.26 to 0.23]). No meaningful changes in prevalence of each individual component of the composite outcome were observed.

**Conclusion**: A substantial decrease in early GDM screening rate did not impact perinatal outcomes in this large diverse cohort of deliveries within an integrated health care system.

## 836 | The Illinois Perinatal Syphilis Warmline: A Public Health Initiative to Address the Syphilis Epidemic

Mariana Espinal^1^; Laurie Ayala^2^; Maura Quinlan^3^; Danucha Brikshavana^3^; Irina Tabidze^4^; Helen Cejtin^2^; Nigel Madden^5^; Nkechinyelum Ogu^2^; Stephanie A. Fisher^2^; Lynn M. Yee^2^

*^1^for the Eunice Kennedy Shriver National Institute of Child Health and Human Development, Bethesda, MD; ^2^Northwestern University Feinberg School of Medicine, Chicago, IL; ^3^Illinois Department of Public Health, Chicago, IL; ^4^Chicago Department of Public Health, Chicago, IL; ^5^Beth Israel Deaconess Medical Center, Boston, MA*

10:30 AM ‐ 12:30 PM

**Objective**: In response to the sharp rise in congenital syphilis (CS) cases in the United States and the complexities of perinatal syphilis management, the Illinois Department of Public Health (IDPH) implemented the first statewide perinatal syphilis clinical support warmline to aid CS prevention. We aimed to evaluate the first 9 months of warmline activity and characterize the public health need for perinatal syphilis consultation.

**Study Design**: The IDPH Perinatal Syphilis Warmline offers remote medical consultation with MFM, adult infectious disease, and pediatric infectious disease experts regarding syphilis diagnosis and treatment during pregnancy and the newborn period, coordinates record searches of prior testing and treatment, and assists in mandatory reporting and linkage to local health department resources. In this prospective cohort study, we performed descriptive analysis of calls to the Warmline from its inception in November 2023 through July 15, 2024.

**Results**: The Warmline served providers from Chicago (55%), other Illinois areas (40%), and outside Illinois (5%). Of 62 consultations, 53% were from outpatient clinics, 21% from labor and delivery units, and 19% from newborn care units. Consultations were primarily requested by obstetric nurses or nurse practitioners (21%), obstetricians (16%), or pediatricians (16%). Most consultations focused on antepartum (40%) or immediately postpartum (31%) management, mainly due to a reactive syphilis test (Fig. 1). Among 46 consultations for syphilis staging in pregnant or recently postpartum individuals, we identified 2 cases of primary syphilis, 6 early latent, 32 late latent, 4 unknown stage, and 2 false positives (Fig. 2). Among 24 neonatal consultations, by CDC CS surveillance case definitions, 9 were possible CS cases, 8 less likely, 3 unlikely, and 4 unknown (Fig. 2).

**Conclusion**: This statewide public health initiative has provided critical, timely expertise on diagnosing and managing perinatal syphilis. We offer a blueprint for other public health jurisdictions to adopt this successful model of enhanced public health surveillance and consultation.

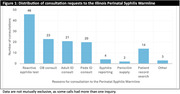

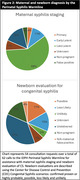

## 837 | Vascular Remodeling of the Maternal Cerebrovascular System

Marie‐Laurence Bilodeau; Christine Wilk; Charley Wing; Virginie Gillet; Genevieve Quesnel; Kevin Whittingstall; Annie Ouellet

*Université de Sherbrooke, Sherbrooke, PQ*

10:30 AM ‐ 12:30 PM

**Objective**: Pregnancy induces significant changes in the peripheral maternal vasculature, yet whether these changes are also present in the brain is unclear. Magnetic resonance angiography through Time Of Flight (MRA‐TOF) technology allows high‐resolution visualization of the cerebral vasculature without the need of contrast agent. The purpose of this study is to investigate maternal intracerebral vessel diameters and intracerebral flow distribution during the third trimester of pregnancy.

**Study Design**: Five pregnant women experiencing normal pregnancy voluntarily agreed to a MRA‐ToF during the third trimester of their pregnancy. The scans took place in 2021 at the Centre Hospitalier Universitaire de Sherbrooke in Fleurimont, Quebec. The images of their main cerebral vasculature were analyzed using an automated software method (eICAB) and compared with 42 non‐pregnant women from publicly available database. The mean diameters of each intracerebral vessel forming the Circle of Willis were collected. Statistical standard test was used for comparison.

**Results**: The mean diameters of the maternal cerebral arteries demonstrated an increase of 23,9% for the internal carotids (4,62 ± 0,2 mm in pregnancy vs 3,73 ± 0,7 mm, p = 0,0001), of 14,8% for the basilar (3,25 ± 0,5 mm vs 2,83 ± 0,6 mm, p = 0,01), of 5,33% for the anterior (2,37 ± 0,1 mm vs 2,25 ± 0,6 mm, p = 0,2) and of 9,4% for the middle cerebral arteries (2,78 ± 0,2 mm vs 2,54 ± 0,7 mm, p = 0,01). However, a decrease of 4,11% was observed in the posterior cerebral artery with diameters of 2,10 ± 0,3 mm vs 2,19 ± 0,4 mm, p = 0,3.

**Conclusion**: Our preliminary results suggests that most cerebral arteries in the brain slightly dilate during pregnancy. A notable exception is the PCA, which tends to slightly constrict. This may be due to the stimulation of the nervous system in the posterior region, though further study will be needed to confirm these findings.

Overall, these results broaden our understanding of the physiological adaptations of maternal intracerebral vascular flow and distribution during normal pregnancy in the different brain areas.

## 838 | Financial Benefits of Audacious but Achievable Goals for Improving Maternal and Neonatal Mortality

Mark I. Evans^1^; Gregory F. Ryan^2^; Christian R. Macedonia^3^; David W. Britt^4^; Jaqueline M. Worth^1^; George M. Mussalli^1^; Sean WD Carter^5^; Matthew Kemp^5^; Mahesh A. Choolani^5^; Lawrence D. Devoe^6^

*^1^Icahn School of Medicine at Mount Sinai, New York, NY; ^2^Ryan Consulting Services, Burlongton, MA; ^3^Lancaster MFM, Lancaster, PA; ^4^Fetal Medicine Foundation of America, New York, NY; ^5^Yong Loo Lin School of Medicine National University of Singapore, Singapore; ^6^Medical College of Georgia at Augusta University, Augusta, GA*

10:30 AM ‐ 12:30 PM

**Objective**: The United States has the highest maternal and neonatal mortality rates (MMR, NMR), of High‐Income Countries, disproportionately for people of color. Inadequate prenatal care contributes to these outcomes, producing increased neonatal costs and diminished economic contributions having long‐term financial consequences < ![if !supportAnnotations] >[MK1]< ![endif] > (FC). Efforts to improve outcomes and reduce disparities have been hampered by laws requiring most states’ require balanced budgets. Unfettered economic arguments are required to justify to gov't agencies the long‐term benefits of upfront expenditures for better outcomes for mother and babies and resultant financial benefits for individuals and states.  We have introduced a new financial metric (DEVELOP score) measuring long‐term FC of governmental (gov't) fiscal policies, including the value of maternal and neonatal lives saved vs typical metrics of costs versus social benefits. Herein, we calculated lives saved and FC of even incremental improvements for every US state by improving its MMR and NMR.

**Study Design**: Using public, vetted national 2021 databases, we ranked MMR and NMR for all US states and DC from best to worst calculating lives saved and FC (by DEVELOP Score) by modeling better care if every state moved up just 1 spot in the state's ranking (i.e., to next highest ranked state's statistics).

**Results**: Nationally, moving up just 1 spot on a states’ rankings saves 451 neonates and 45 mothers generating an annual increased total FC of $504,313,479 (resultant improved economics of $457,149,465 for neonates and $47,164,015 for mothers. Fig 1).

**Conclusion**: Mixed financial/social benefit constructs have mostly failed to convince the Federal and State gov'ts to invest sufficiently to substantively improve prenatal and neonatal outcomes. By modeling even modest, reachable gains, our approach provides “cover” for bureaucracies to justify a longer‐ term vision. Instead of focusing exclusively on curbing expenditures, these monies should be considered as investments which will be considerably profitable in the long run.

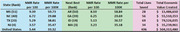

## 839 | High Rural Water Per‐ and Polyfluorinated Alkyl Substances (PFAS) Levels and Preeclampsia

Ellie Baltes^1^; Darrin Thompson^1^; Heath A. Davis^2^; Serena Gumusloglu^1^; Hanna Stevens^1^; David Cwiertny^1^; Donna A. Santillan^1^; Mark K. Santillan^1^

*^1^University of Iowa, Iowa City, IA; ^2^University of Iowa, Institute for Clinical and Translational Science, Iowa City, IA*

10:30 AM ‐ 12:30 PM

**Objective**: Per‐ and Polyfluorinated Alkyl Substances (PFAS) are synthetic chemicals used in pesticides. PFAS are found in humans with drinking water as the key route of entry. PFAS exposure in human and preclinical models is associated with preeclampsia (PreE). Yet, adequate control for covariates are limited in previous studies. The objective of this study to estimate the effect of PFAS levels in rural populations on the development of PreE.

**Study Design**: This retrospective cohort study uses a dataset from the Intergenerational Health Knowledgebase (N = 78,726 pregnancies, IRB#20101369) an integrated datamart of all EHR data of maternal, pediatric, and pregnancy care at the University of Iowa Health Care system. Pregnancies from high PFAS (hiP) areas (N = 1880) were identified by zip codes in which the municipal water tested higher than the maximum contaminant level (MCL) for one PFAS chemical as determined by the Iowa Department of Natural Resources. Low PFAS (loP) pregnancies (N = 1602) were identified by zip codes in which the MCL for any PFAS was not exceeded. Baseline characteristics were compared for both groups (alpha = 0.05).  Logistic regression models were constructed to evaluate the association of municipal water PFOS levels and PreE.

**Results**: Pregnancies from hiP areas in comparison to loP were significantly more rural by RUCA code (36% vs 3%, p< 0.001), had higher obesity rates (48% vs. 35%, p< 0.001), and higher PreE rates (12% vs. 9%, p = 0.014). Rates of diabetes, racial distribution, and rates of adverse neonatal outcomes were similar between the two groups.  After controlling for obesity, race, and diabetes diagnosis, hiP pregnancies were associated with higher odds of developing PreE in comparison to loP pregnancies (aOR = 1.4 [1.1‐1.8], p = 0.020).

**Conclusion**: High rural municipal water PFAS levels are associated with a higher odds of developing PreE even after controlling significant covariates. Future studies should investigate PFAS level exposure at the participant level to determine a more granular association with PFAS levels and PreE development.

## 840 | Hypertensive Diseases of Pregnancy Are Significantly Associated with Development of Long‐Term Neonatal Seizures

Mark K. Santillan^1^; Donna A. Santillan^1^; Serena Gumusoglu^1^; Heath A. Davis^2^; Baojian Xue^1^; Grant Tiarks^1^; Brittany Todd^1^; Angela Wong^1^; Vinit Mahajan^3^; Alan Johnson^1^; Polly Ferguson^1^; Elizabeth Newell^1^; Jason Misurac^1^; Alexander Bassuk^1^

*^1^University of Iowa, Iowa City, IA; ^2^University of Iowa, Institute for Clinical and Translational Science, Iowa City, IA; ^3^Stanford University, Stanford, CA*

10:30 AM ‐ 12:30 PM

**Objective**: Hypertensive Diseases of Pregnancy (HDP) are associated with many long term maternal and child neurologic conditions. While neurodevelopmental and psychiatric conditions and maternal seizures are known to be associated with HDP, the relationship to neonatal seizures (NSz) is not well documented given the overall low rate of NSz.  Data suggest common vascular mechanisms in HDP and maternal and offspring neuropathology. The objective of this study is to address the hypothesis that HDP is associated with an increased risk of long‐term NSz.

**Study Design**: This case‐control study uses a dataset from the Intergenerational Health Knowledgebase (N = 78,726 pregnancies, IRB#20101369) an integrated datamart of all short‐term and long‐term EHR data of maternal, pediatric, and pregnancy care at the University of Iowa Healthcare (UIHC) system. A composite case definitions of NSz (G40, R56, P90, R25.9, R40.4) and HDP (O10‐11, 13‐14, 16, I 10,15) were constructed using ICD‐10 codes.  Baseline characteristics were compared between cases and controls (alpha = 0.05).  Logistic regression models were constructed to evaluate the association between the development of NSz ad HDP.

**Results**: NSz pregnancies (N = 1370) exhibited significantly higher rates of maternal BMI >40 (16% vs. 12%, p< 0.001), adverse neonatal outcomes (ANO, 59% vs. 35%, p< 0.001), HDP (37% vs. 33%, p< 0.001) in comparison to controls (N = 34297). NSz and control pregnancies had similar gestational ages at delivery (37.7 ± 31.6 vs. 38.1 ± 25.6 weeks, p = 0.571).  After controlling for age, gravida,  BMI, diabetes in, and race, pregnancies affected by HDP are at higher odds of developing subsequent seizures in the offspring (aOR = 1.132 [1.003‐1.278], p = 0.045).

**Conclusion**: Although overall incidence of NSz is low, our data demonstrate a clear association of a high risk of long‐term NSz in neonates born from pregnancies affected by HDP. Further studies should be directed at the human and preclinical shared mechanisms that link hypertensive diseases during pregnancy and neuropathology.

## 841 | The Association Between Maternal Comorbidity Burden and Severe Neonatal Morbidity

Matthew J. Blitz^1^; Insaf Kouba^2^; Ji Yoon Lee^1^; Annemarie Stroustrup^1^

*^1^Northwell, New Hyde Park, NY; ^2^Northwell, St. Petersburg, FL*

10:30 AM ‐ 12:30 PM

**Objective**: To determine the association between maternal comorbidity burden and severe neonatal morbidity (SNM), and to evaluate whether racial and ethnic group differences help to explain any disparities in outcomes.

**Study Design**: This was a retrospective cohort study of all deliveries at ≥23 weeks gestational age at two tertiary hospitals in New York from 2019‐2022. Exclusion criteria were fetal demise and multiple gestation. The primary exposure was obstetric comorbidity index (OB‐CMI) score, calculated at admission for delivery. This score is based on 24 weighted comorbidity indicators identified by ICD‐10 codes and clinical documentation. Covariate factors assessed included race and ethnicity, health insurance, marital status, parity, and preferred language. The primary outcome was SNM, a composite neonatal adverse outcome indicator which includes pre‐determined diagnoses and procedures. Multivariable logistic regression was used to model the probability of SNM as a function of OB‐CMI score group, while adjusting for covariate factors.

**Results**: A total of 54,248 patients were included. Non‐Hispanic White patients constituted the largest race and ethnicity group (42.8%), followed by Asian or Pacific Islander (18.7%), Non‐Hispanic Black (14.5%), and Hispanic (12.2%). The overall SNM rate was 7.6% (n = 4,127). SNM increased from 5% among patients with an OB‐CMI score of zero to greater than 30% when OB‐CMI scores were 9 or more (Figure 1). Results of regression modeling are presented in Figure 2. Each successive OB‐CMI group had an increased odds of SNM, and patients with OB‐CMI ≥4 had more than 4 times greater odds of SNM compared to patients with an OB‐CMI = 0. Non‐Hispanic Black patients and Hispanic patients were at increased risk for SNM compared to non‐Hispanic White Patients.

**Conclusion**: OB‐CMI score is positively correlated with SNM. Racial and ethnic disparities in SNM were observed. Future studies should assess if these differences can be better explained by social determinants of health, environmental exposures, genetics, and nutrition.

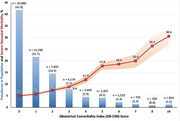

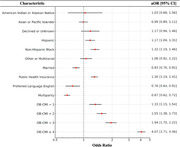

## 842 | The use of a Fetal Pillow Device at Full Dilation Cesarean Delivery

Maya Ronen^1^; carmel porat^2^; moshe betser^3^; lee Segev^2^

*^1^Shamir Medical Center, Shamir medical Center, HaMerkaz; ^2^Shamir Medical Center, Beer Yaacov, HaMerkaz; ^3^Shamir Medical Center, beer yaacov, HaMerkaz*

10:30 AM ‐ 12:30 PM

**Objective**: Impacted fetal head at full dilation cesarean delivery (CD) is a major cause of adverse maternal and neonatal outcomes. The Fetal Pillow is a device designed to reduce these complications. Our study aims to evaluate the outcomes of full‐dilation CD with and without the use of Fetal Pillow.

**Study Design**: This retrospective cohort study included full‐dilation CDs performed from January 2018 to July 2023, at a single tertiary center.  Indications for CDs were arrest of decent, fetal distress and failed vacuum extraction. The study cohort included cases (Fetal Pillow group) matched to controls (without the use of Fetal Pillow) according to the indication to CD, in a 1: 2 ratio. The study evaluated maternal outcomes such as uterine incision extensions, maternal blood loss (ml), maternal postoperative infection and postoperative length of stay (days). Neonatal outcomes included NICU admissions, cord arterial blood pH, Apgar scores, respiratory distress, birth trauma, and encephalopathy.

**Results**: The study cohort included a total of 138 patients who underwent CD at full dilation. Among them, 46 were in the Fetal Pillow group, and 92 were controls. There were no significant differences between the groups in maternal characteristics such as age, BMI, previous CDs and obstetrics complications. There were also no differences between the groups in the rate of uterine extensions, maternal blood loss, postoperative infection or postoperative length of stay . The fetal pillow group had a lower rate of neonatal NICU admissions compared to the controls (17.3% versus 33.7%, respectively, p = 0.04). There were no differences between the groups in any other fetal outcomes.

**Conclusion**: The use of Fetal Pillow for impacted fetal head during full dilation CDs was not associated with any maternal complications. The use of Fetal Pillow may reduce NICU admissions.

## 843 | Chronic Hypertension: Timing of Delivery and Associated Morbidity

Mayra Shafique^1^; Xilin Chen^1^; Molly J. Stout^2^; Lisa Kane Low^1^; Alex Peahl^3^; Michelle Moniz^1^; Jourdan E. Triebwasser^1^

*^1^University of Michigan, Ann Arbor, MI; ^2^University of Michigan Medical Center, Ann Arbor, MI; ^3^Michigan Medicine, Ann Arbor, MI*

10:30 AM ‐ 12:30 PM

**Objective**: The American College of Obstetricians and Gynecologists (ACOG) recommends delivery for uncomplicated chronic hypertension (cHTN) at 37w0d‐39w6d. We assessed delivery timing for cHTN and its association with cesarean birth, severe maternal morbidity (SMM), and severe neonatal morbidity (SNM) in a quality collaborative.

**Study Design**: Retrospective cohort study of nulliparous term singleton vertex births across 71 hospitals using clinically abstracted values from the Obstetrics Initiative, a quality collaborative supported by Blue Cross Blue Shield of Michigan and Blue Care Network. We included births complicated by cHTN from 01/2020 to 12/2023. The exposure was week of delivery and outcomes were cesarean rate, SMM (Centers for Disease Control definition), and SNM (unexpected complications in term newborns, PC‐06). Adjusted odds ratios (aOR) were calculated with generalized linear mixed models with hospital random effects (to control for clustering) and adjustment for age, body mass index, diabetes, substance use, and social vulnerability index. A sensitivity analysis excluding 37‐week deliveries was performed to assess delivery timing with assumption of better controlled cHTN at later gestational age.

**Results**: Among 3963 (3.6%) births with cHTN, delivery occurred at 37 (31.1%), 38 (33.8%), 39 (25.4%), and 40+ weeks (9.7%). Compared to 37 weeks, cesarean was lower at 38 (aOR 0.79, 95% CI 0.75‐0.84) and higher at 40+ (aOR 1.15, 95% CI 1.10‐1.21). SMM and SNM were lower at all gestational weeks compared to 37 weeks (**Table 1**). In the sensitivity analysis SMM was lower (aOR 0.92, 95% CI 0.92‐0.93) and SNM was higher (aOR 1.02, 95% CI 1.02‐1.02) in births at 39+ weeks versus those at 38 weeks with no difference in cesarean (aOR 0.92, 95% CI 0.82‐1.15).

**Conclusion**: Almost 10% of patients with cHTN delivered at 40+ weeks, outside ACOG recommendations for cHTN. Delivery at 37 weeks is associated with morbidity that is likely related to severity of cHTN or superimposed preeclampsia. If delivery is not indicated at 37 weeks, there was no clear benefit of delivery at 38 vs. 39 weeks.

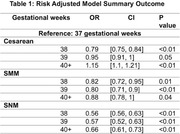

## 844 | Corticosteroid Impact on Umbilical Artery Dopplers in Fetal Growth Restriction Pregnancies: Systematic Review and Meta‐Analysis

Meagan Kline^1^; Kendra M. Gray^2^; Rosanne Kho^1^; Sarah Kellerhals^1^; Pamela Garcia‐Fillion^3^; N. Brandon Barba^3^

*^1^University of Arizona College of Medicine ‐ Phoenix / Banner University Medical Center ‐ Phoenix, Phoenix, AZ; ^2^Dignity Health, Phoenix, AZ; ^3^University of Arizona College of Medicine ‐ Phoenix, Phoenix, AZ*

10:30 AM ‐ 12:30 PM

**Objective**: Current guidelines recommend the administration of antenatal corticosteroids (ACS) for pregnancies with fetal growth restriction (FGR) and abnormal umbilical artery Doppler (UAD) velocimetry. This systematic review examined the change in UAD after the administration of ACS in pregnancies complicated by FGR and any associations with perinatal outcomes.

**Study Design**: Seven electronic databases (PubMed, Cochrane Library, OVID Medline, Global Index Medicus, Scopus, Google Scholar and Embase) were searched from 1980 to May 2024 using MESH‐terms for studies in English. Eligible studies provided a FGR definition and documentation of UAD prior to and after ACS administration. All studies were independently evaluated following PRISMA guidelines. Risk of bias was assessed via the ROBIN‐I tool. Meta‐analysis was conducted using the metafor package for R.

**Results**: A total of 13 studies were included with a total of 585 pregnancies.  Six studies reported changes in end‐diastolic flow (EDF) while 7 studies reported on pulsatility index (PI). Baseline PI decreased from 2.06 (95% CI, 1.63‐2.49) to 1.78 (95% CI, 1.39‐2.17) after administration of ACS, but subsequently returned to a baseline PI of 2.00 (95% CI, 1.56‐2.43) at time of final Doppler evaluation. In studies evaluating EDF, 44% (95% CI, 33‐55%) of patients showed improvement. Patients with improvement in EDF were found to have a lower rate of perinatal mortality compared to persistent absent or reversed EDF (6% vs. 12%).

**Conclusion**: Evidence suggests that UAD assessment after administration of ACS in fetuses with FGR may result in a transient change in UAD. Improvement of UAD after ACS may prognosticate more favorable perinatal outcomes.  This systematic review and meta‐analysis did highlight that the studies evaluating UAD changes in pregnancies affected by FGR are inconsistent in the metrics used to evaluate these changes. A set of standardized metrics is necessary for reporting ultrasound changes and perinatal outcomes in studies of Doppler changes following ACS in pregnancies affected by FGR.

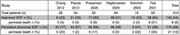

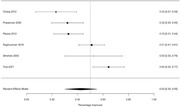

## 845 | Leveraging Pharmacogenomic Data for Methadone Dosing in Pregnancy

Megan B. Raymond^1^; Dennis Hand^2^; Michael Tarallo^1^; Walter Kraft^3^; Rupsa C. Boelig^4^

*^1^Thomas Jefferson University, Philadelphia, PA; ^2^Jefferson Center for Maternal Addiction Treatment Education and Research, Philadelphia, PA; ^3^Thomas Jefferson University Hospital, Philadelphia, PA; ^4^Sidney Kimmel Medical College, Thomas Jefferson University, Philadelphia, PA*

10:30 AM ‐ 12:30 PM

**Objective**: Opioid use disorder (OUD) in pregnancy remains a significant cause of maternal/infant morbidity in the United States, making appropriate OUD treatment in pregnancy critical. Pregnancy increases methadone metabolism, and studies in non‐pregnant adults demonstrate that CYP2B6 genetic variants are associated with altered methadone metabolism. Current protocols for methadone management in pregnancy do not take into consideration the potential impact of CYP2B6 phenotype, and data on how CYP2B6 phenotype impacts methadone dosing in pregnancy are lacking.

**Study Design**: This is a prospective study of pregnant patients with OUD admitted for inpatient methadone stabilization. Methadone stabilization followed a standardized protocol involving withdrawal assessments every four hours, as‐needed methadone doses based on withdrawal scores, and daily dose increases until stability. Participants underwent sampling for a pharmacologic study, collection of medical history and details of dosing through hospitalization, and CYP2B6 genotyping. CYP2B6 phenotype was characterized as either rapid metabolizer or not (slow, intermediate or normal). Primary outcome was final dose at discharge.

**Results**: Of N = 40 enrolled, 29 had successful genotyping (Table 1), and 3/29 (10%) were rapid metabolizers. Rapid metabolizers had a higher mean dose at discharge, although this was not statistically significant (177+/‐90mg vs 125mg +/‐75mg, mean difference 52mg (‐43mg to 147mg), p = 0.27). These results were results similar when restricted to participants in the 2^nd^ trimester. Two patients who were rapid metabolizers left against medical advice prior to stable dose. The last dose they received was used for analysis.

**Conclusion**: Pregnant patients treated for OUD with a CYP2B6 rapid metabolizer phenotype had a trend toward a higher mean dose of methadone than non‐rapid metabolizers; although our cohort was underpowered for statistical significance. This may support an impact of CYP2B6 genotype on methadone systemic exposure, which has implications for optimizing dosing protocol in pregnancy.

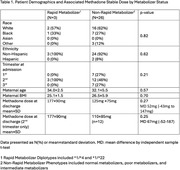

## 846 | Prolonged Second Stage: How Does Obesity Influence Likelihood of Successful Vaginal Delivery?

Xiang Yu Feng^1^; Christina Frasik^1^; Megan C. Oakes^2^

*^1^University of California, Irvine Division of Maternal‐Fetal Medicine, University of California, Irvine, CA; ^2^MemorialCare Miller Children's and Women's Hospital Long Beach, Miller Children's and Women's Hospital, Long Beach, CA*

10:30 AM ‐ 12:30 PM

**Objective**: In 2024, ACOG revised clinical guidelines supporting the definition of a prolonged second stage labor as ≥ 3 hours. We sought to evaluate the difference in vaginal delivery rate for nulliparous patients with a singleton, term, vertex fetus (NTSV) with prolonged second stage between those with and without obesity.

**Study Design**: A retrospective cohort study of NTSV patients with a prolonged second stage of labor (≥ 3 hours) delivered at a single tertiary care hospital from 6/1/2023‐12/31/2023. Obesity was defined as body mass index (BMI) >30 kg/m^2^ at delivery. The primary outcome was vaginal delivery and secondary outcomes were select maternal and neonatal morbidities. Categorical variables were compared using Chi square, Fisher's exact and normally distributed continuous variables using Student's T test, as appropriate. Multivariable logistic regression was used to adjust for confounders.

**Results**: 173 patients with a prolonged second stage were included, 99 (57.2%) without obesity and 74 (42.8%) with obesity. Patients with obesity were more likely to have hypertension (p = 0.04) and hypertensive disorders of pregnancy (p = 0.02), as well as utilize oxytocin in the second stage (p = 0.04). 94 (94.9%) patients without obesity achieved a vaginal delivery compared to 62 (83.8%) with obesity (RR 0.88, 95% CI 0.67‐0.98). After adjusting for confounders and compared to patients without obesity, patients with obesity were noted to have an adjusted relative risk of 0.81 (95% CI 0.54‐0.96) for vaginal delivery. There was no difference between the groups with regard to operative vaginal delivery and maternal and neonatal morbidities. These findings persisted after adjusting for confounders.

**Conclusion**: While patients with obesity had a lower likelihood of vaginal delivery after a prolonged second stage compared to patients without obesity, both groups were overall highly successful in delivering vaginally and no differences in likelihood for select maternal or neonatal complications occurred. These findings may aid in counseling patients with a prolonged second stage.

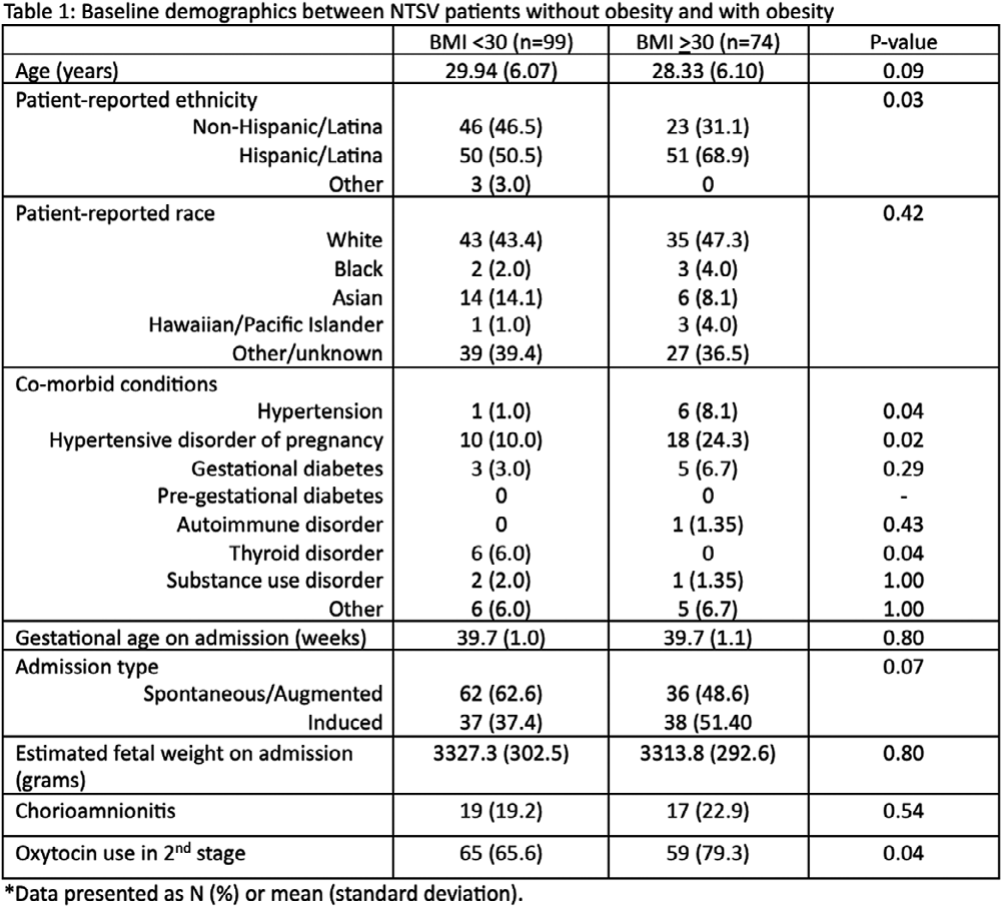

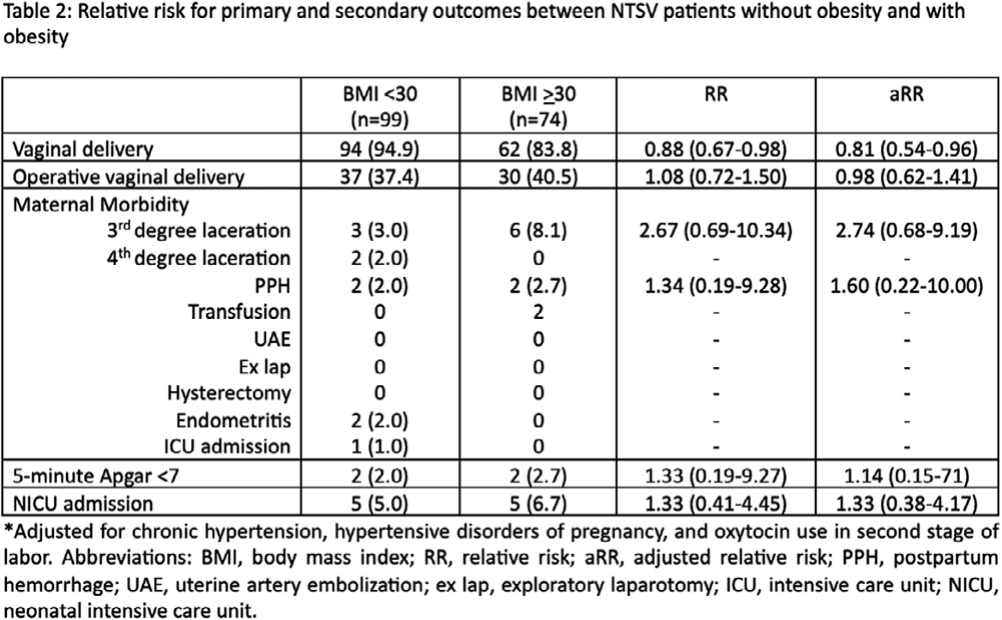

## 847 | Perinatal Outcomes in Pregnant Patients with a History of Migraine Headaches

Megha Arora; Bharti Garg; Aaron B. Caughey

*Oregon Health & Science University, Portland, OR*

10:30 AM ‐ 12:30 PM

**Objective**: To examine maternal and neonatal outcomes among pregnant patients with and without a history of migraine headaches.

**Study Design**: We conducted a retrospective cohort study of births in the state of California from 2008‐2020 to compare perinatal outcomes between those with and without history of migraine headaches. Singleton, non‐anomalous deliveries at 23‐42 weeks of gestation were used. Outcomes included gestational hypertension, preeclampsia, severe maternal morbidity (SMM), preterm birth (< 37 weeks), NICU admission, and respiratory distress syndrome (RDS).  We performed multivariable logistic regression controlling for age, race/ethnicity, education, pre‐pregnancy BMI, parity, and insurance status to estimate adjusted odds ratios (aOR) with 95% confidence intervals.

**Results**: In the cohort of 5,065,732 individuals, there were 48,518 (0.96%) individuals with history of migraines.  There were higher rates of migraines among those who were white, highly educated, and privately insured. On multivariable regression, those with migraines had higher odds of gestational hypertension (7.1% vs 3.4%; aOR 1.75, 95% CI 1.69‐1.82), preeclampsia (6.7% vs 3.8%; aOR 1.70, 95% CI 1.64‐1.76), SMM (2.0% vs 1.2%; aOR 1.71, 95% CI 1.61‐1.83), preterm delivery (8.8% vs 6.4%; aOR 1.45, 95% CI 1.40‐1.50), and neonatal RDS (4.5% vs 2.3%; aOR 1.75, 95% CI 1.67‐1.83).

**Conclusion**: In this large cohort of pregnant patients, those with a history of migraines were more likely to experience adverse perinatal outcomes, despite the presence of multiple protective social determinants with respect to socioeconomic status and experience of racism. This signals that there may be physiologic or structural vulnerabilities among individuals with migraines contributing to adverse outcomes during pregnancy which require further characterization.

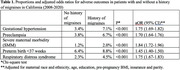

## 848 | Association of Interpregnancy Interval and Perinatal Outcomes Among Individuals with History of Pprom

Megha Arora; Bharti Garg; Ava D. Mandelbaum; Aaron B. Caughey

*Oregon Health & Science University, Portland, OR*

10:30 AM ‐ 12:30 PM

**Objective**: To examine the association of interpregnancy interval with adverse perinatal outcomes among individuals with a history of preterm prelabor rupture of membranes (PPROM).

**Study Design**: This was a retrospective cohort study of two consecutive births in California between 2008‐2020 among patients with a history of PPROM in the index pregnancy. Interpregnancy interval (IPI) was categorized as < 12 months, 12‐23 months, 24‐35 months, 36‐59 months, and ≥60 months. Outcomes included gestational hypertension, preeclampsia, preterm birth (< 37 weeks), severe maternal morbidity (SMM), and primary cesarean delivery. Chi squared and multivariable logistic regression were used for statistical analysis.

**Results**: In our cohort of 15,271 individuals with a history of PPROM in an index pregnancy, 3,299 (21.6%) had a short IPI (< 12 months) and 1,537 had a long IPI (≥60 months). As compared to IPI of 12‐23 months, pregnancies with IPI< 12 months had greater odds of preterm birth (24.10% vs 19.54%; aOR 1.22, 95% CI 1.09‐1.37), SMM (1.52% vs 0.86%; aOR 1.58, 95% CI 1.03‐2.43), and primary cesarean delivery (11.3% vs 9.26%; aOR 1.28, 95% CI 1.08‐1.53). Pregnancies with IPI≥60 months also had increased odds of adverse outcomes including preeclampsia (6.57% vs 3.63%; aOR 1.66, 95% CI 1.28‐2.15), preterm birth (24.20% vs 19.54%; aOR 1.22, 95% CI 1.06‐1.41), SMM (1.95% vs 0.86%; aOR 1.85, 95% CI 1.12‐3.03), and primary cesarean (13.63% vs 9.26%; aOR 1.45, 95% CI 1.17‐1.80).

**Conclusion**: Both short (< 12 months) and long (≥60 months) IPIs were associated with adverse perinatal outcomes among patients with a history of PPROM. These findings highlight the importance of counseling those with PPROM in a prior pregnancy on optimal pregnancy timing to mitigate risks in subsequent pregnancies.

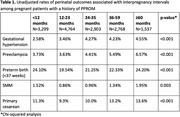

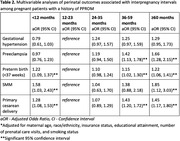

## 849 | Preconception Hormone Levels are Associated with Preterm Birth and Measures of Maternal Stress and Mood

Melissa Scott^1^; Sharon Hunter^2^; M. Camille Hoffman^2^

*^1^University of Colorado School of Medicine, Department of Obstetrics & Gynecology, University of Colorado, CO; ^2^University of Colorado School of Medicine, Aurora, CO*

10:30 AM ‐ 12:30 PM

**Objective**: Chronic stress during pregnancy has been associated with adverse pregnancy outcomes (APOs) including preterm birth (PTB). In addition, progesterone is crucial in the maintenance of pregnancy and deficiency can increase the risk of miscarriage and PTB. Assessment of hormone levels in human hair provides a stable, non‐invasive measurement of chronic hormonal activity. Our objective was to determine the association between preconception maternal hair cortisol and progesterone levels and APOs.

**Study Design**: Preconception hair hormone levels (HHLs), mood, and APOs were evaluated in participants who were part of a larger prospective cohort study during which maternal hair was cut 3 times during pregnancy: around 16 weeks, 28 weeks, and delivery. As the proximal 1cm of hair from the scalp represents ∼one preceding month of hair growth, segments longer than 3 cm at 16 weeks were considered to represent the preconception period.  HHLs were analyzed using mass spectrometry.  Participant data was collected via chart review and interview.  Our primary outcome was the relationship between preconception HHLs and delivery < 37 weeks (PTB). Secondary outcomes included: pre‐eclampsia/PIH (PREE) and maternal mood questionnaires including the State‐Trait Anxiety Inventory (STAI), Center for Epidemiologic studies‐depression scale (CESD). Data were analyzed using Spearman correlations, p < 0.05 considered significant.

**Results**: Of 163 participants, 110 and 34 participants had preconception cortisol and progesterone HHLs available for analysis, respectively. Correlations were noted between cortisol and PTB (r = 0.20, p = 0.04) and progesterone and STAI and CESD scores (r = 0.34, p = 0.03 and r = 0.33, p = 0.042). Additional correlations (p >0.05) included positive relationships between cortisol and: PREE and CESD score at all gestational ages.

**Conclusion**:   Higher preconception cortisol is correlated with higher risk of PTB and higher preconception progesterone correlates with depression and anxiety.  These data improve our understanding of the complex relationships between preconception stress, mood, and pregnancy outcomes.

## 850 | Can Remote Blood Pressure Monitoring Reduce Racial Disparities in Outcomes Among Postpartum Patients with Hypertension?

Shana D. Talbot^1^; Tracy L. Jackson^2^; Scott Machado^2^; Nicole Konecke^2^; William Hunt^2^; Maria Mejia Castillo^2^; Danielle Simmons^2^; Heather A Smith^1^; Nina K. Ayala^1^; William A. Grobman^3^; Adam K. Lewkowitz^2^; Methodius G. Tuuli^1^

*^1^Women & Infants Hospital of Rhode Island / Alpert Medical School of Brown University, Providence, RI; ^2^Women & Infants Hospital of Rhode Island / Alpert Medical School of Brown University., Providence, RI; ^3^The Ohio State University, Columbus, OH*

10:30 AM ‐ 12:30 PM

**Objective**: Remote self‐measured blood pressure (SMBP) monitoring programs have been shown to reduce racial inequity in postpartum blood pressure ascertainment. However, their effect on disparities in healthcare utilization and severe maternal morbidity (SMM) is less clear. We aimed to compare outcomes between participants in our remote SMBP program who self‐identified as Black versus non‐Hispanic White.

**Study Design**: Postpartum individuals with hypertension (HTN) at our tertiary hospital are offered enrollment in our remote SMBP program. For this analysis, participants who did not self‐identify as Black or non‐Hispanic White were excluded. The primary outcome was a composite of HTN‐related postpartum readmission or ED visit within 30 days of delivery. Secondary outcomes were individual components of the primary composite, HTN‐related SMM, and anti‐hypertension medication initiation or titration. A log‐link binomial generalized linear model was used to estimate relative risks (aRR) adjusting for potential confounders.

**Results**: Among 2003 participants in the SMBP program from 2022 ‐ 2024, 1363 met inclusion criteria. Of these, 988 (49.3%) self‐identified as non‐Hispanic White and 375 (18.7%) as Black. Compared to non‐Hispanic White participants, Black participants were younger, and more likely to have public insurance and gestational HTN (**Table 1**). After adjusting for these factors, there was no significant difference in the composite of HTN‐related postpartum readmission or ED visit between Black and non‐Hispanic White participants (19.2% vs 16.7%; aRR 1.29, 95% CI 0.98, 1.69). Risk of HTN‐related readmission alone was significantly higher among Black participants (12.2% vs 9.9%; aRR 1.48, 95% CI 1.03, 2.11). There was no significant difference in HTN‐related SMM (12.5% vs 11.7%; aRR 1.00, 95% CI 0.72, 1.42). There was also no difference in anti‐hypertensive medication initiation or titration (**Table 2**).

**Conclusion**: Our remote SMBP program for postpartum patients with HTN eliminated racial disparities in overall HTN‐related healthcare utilization and HTN‐related SMM.

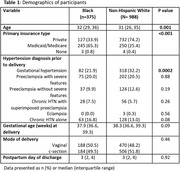

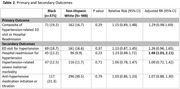

## 851 | Should Fasting Blood Glucose Value on the Ogtt be Incorporated Into Initial Gestational Diabetes Counseling?

Mia A. Heiligenstein^1^; Erica Glaser^2^; Elianna Kaplowitz^2^; Guillaume Stoffels^3^; Camila Johanek^3^; Thomas Owens^4^; Olivia Grubman^5^; Xiteng Yan^2^; Sophia Scarpelli‐Shchur, RN, CDE^2^; David Cole^2^; Zainab Al‐Ibraheemi^4^; Andrei Rebarber^6^; Lois Brustman^4^

*^1^Mount Sinai West, Astoria, NY; ^2^Mount Sinai West, New York, NY; ^3^Icahn School of Medicine at Mount Sinai, New York, NY; ^4^Icahn School of Medicine at Mount Sinai West, New York, NY; ^5^Westchester Medical Center, Westchester, NY; ^6^Icahn School of Medicine at Mount Sinai Hospital, New York, NY*

10:30 AM ‐ 12:30 PM

**Objective**: Hypoglycemic agents are used to optimize glycemic control in the treatment of GDM.The aim of this study was to assess the predictability of the fasting blood glucose value(FBS) on the OGTT in predicting the need for hypoglycemic agents in patients with GDM.

**Study Design**: This study was a single‐center retrospective cohort study of singleton pregnancies with GDM between 2018‐2023.Diagnosis was made by Carpenter‐Coustan(CC) criteria.Initial management involved dietary and lifestyle modifications, with hypoglycemic agents introduced if blood glucose values were above threshold(FBS > 90mg/dl, 2hpp > 120mg/dl or 1hpp > 140mg/dl).Receiver operating characteristic curves(ROC) were generated to determine predictability of FBS on the OGTT for the need for hypoglycemic agents.Predictability of FBS on OGTT was evaluated using the clinical cut‐off values of 81mg/dl(based on the Youden's Index),95mg/dl(based on CC) and 105mg/dl(based on NDDG). Sensitivity, specificity, positive and negative predictive values were calculated.

**Results**: 1,407 patients diagnosed with GDM, 567(40%) were prescribed hypoglycemic agents. There was a statistically significant difference between patients that required hypoglycemic agents in regards to age, BMI, race, and history of GDM.FBS on OGTT was predictive of need for hypoglycemic agents based on the ROC curve with an AUC of 0.76 (95% CI 0.74‐0.79), indicating a 76% chance that FBS on GTT can distinguish between patients who did and did not need hypoglycemic agents(Figure 1). The value with the highest Youden's Index was 81mg/dl.A cut‐off value of 95mg/dL had the highest positive predictive value(PPV); of women who had ≥ 95mg/dL FBS on OGTT, 75.25% required hypoglycemic agents, whereas 32.9% of patients with a FBS < 95mg/dl needed hypoglycemic agents(Table 1).

**Conclusion**: Our data suggests that if a patient has a FBS of  ≥ 95mg/dL there is a 75% chance that they will require hypoglycemic agents.This can be used during initial counseling to educate patients diagnosed with GDM on the possibility of the need for hypoglycemic agents and provide reassurance to patient's with FBS < 95mg/dL.

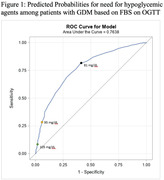

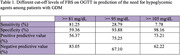

## 852 | What Best Predicts for Need for Hypoglycemic Agents in Patients with Gestational Diabetes?

Mia A. Heiligenstein^1^; Erica Glaser^2^; Elianna Kaplowitz^2^; Guillaume Stoffels^3^; Camila Johanek^3^; Thomas Owens^4^; Olivia Grubman^5^; Xiteng Yan^2^; Leslie Rebarber^2^; Sophia Scarpelli‐Shchur, RN, CDE^2^; David Cole^2^; Zainab Al‐Ibraheemi^4^; Lois Brustman^4^

*^1^Mount Sinai West, Astoria, NY; ^2^Mount Sinai West, New York, NY; ^3^Icahn School of Medicine at Mount Sinai, New York, NY; ^4^Icahn School of Medicine at Mount Sinai West, New York, NY; ^5^Westchester Medical Center, Westchester, NY*

10:30 AM ‐ 12:30 PM

**Objective**: This study aimed to identify antenatal characteristics that best predict the need for hypoglycemic agents in patients with gestational diabetes (GDM).

**Study Design**: This study was a single‐center retrospective cohort study of patients with singleton pregnancies between 2018‐2023, diagnosed with GDM between 24‐34 weeks gestation by Carpenter‐Coustan criteria on a 3hour OGTT. Hypoglycemic agents were added when optimal control was not achieved as determined by serial finger stick analysis (FBS < 95mg/dl, postprandial values at 1h 140mg/dl, 2h 120mg/dl).Logistic regression models were developed to determine the need for hypoglycemic agents and included commonly associated predictors of disease severity. Data collected included age, body mass index (BMI), race, gestational age (GA) at diagnosis, history of GDM, and the 1h, 2h, and 3h OGTT values.Model 1 utilized only the fasting OGTT value as the predictor.Model 2 included the aforementioned variables excluding the fasting OGTT value.Model 3 incorporated the fasting OGTT value along with the other predictors from Model 2.

**Results**: 1,407 patients were diagnosed with GDM;567(40%) were prescribed hypoglycemic agents.

A comparative analysis of the three models was performed to evaluate their predictive performance(Table 1, Figure 1).Model 1, using only the fasting OGTT value, had an area under the curve (AUC) of 0.77.Model 2, which excluded the fasting OGTT value, had an AUC of 0.71.Model 3, incorporating both the fasting OGTT value and the other predictors from Model 2, achieved an AUC of 0.76.This was significantly different from Model 2 (p  < 0.0001) and Model 1 (p = 0.0021) (Table 1).  

**Conclusion**: Our model suggests that the fasting value on the OGTT alone (Model 1) is a significant predictor of the need for hypoglycemic agents when compared to common predictors for disease severity (Model 2). Therefore, the isolated fasting value is a valuable clinical predictor in assessing the risk for hypoglycemic agents and should be used when counseling patients.

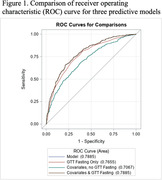

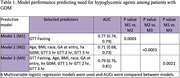

## 853 | Randomized Controlled Trial of Detemir versus NPH in Gestational and Type 2 Diabetes: DETERMINE Study

Michael Richley^1^; Kandace Fung^2^; Megan C Oakes^3^; Ann Nguyen^4^; Aisling M. Murphy^5^; Thalia Mok^5^; Jenny Lester^2^; Lorna Kwan^2^; Maral Demirjian^2^; Rebecca Gerl^2^; Dana Levin‐Lopez^2^; Matthew Freeby^2^; Alice Sherman‐Brown^4^; Judith H. Chung^6^; Christina S. Han^2^

*^1^University of Washington, Seattle, WA; ^2^UCLA, Los Angeles, CA; ^3^MemorialCare Miller Children's and Women's Hospital, Long Beach, CA; ^4^UC Irvine, Orange, CA; ^5^University of California, Los Angeles, Los Angeles, CA; ^6^University of California‐ Irvine, Irvine, CA*

10:30 AM ‐ 12:30 PM

**Objective**: Diabetes mellitus (DM) is one of the most common complications in pregnancy. Neonatal hypoglycemia (HG) as a result of DM is associated with NICU admissions and delay to milestones. We hypothesized that the longer‐acting insulin detemir may yield lower rates of neonatal HG, compared to intermediate‐acting NPH in pregnancies with GDM or T2DM.

**Study Design**: We conducted a prospective, multi‐center, unblinded, randomized‐controlled trial. Subjects requiring insulin initiation during pregnancy were randomized 1:1 to detemir or NPH. IRB approval and clinicaltrials.gov registration were obtained. Primary outcomes were rates of neonatal HG (< 45 mg/dl) in the first 24 hours of life, and persistent HG (< 50 mg/dl) beyond 24 hours. Secondary outcomes were rates of maternal, antenatal and neonatal complications. Data were analyzed using Wilcoxon Rank sum, Chi‐Square or Fisher's exact tests. Uni‐ and multivariable logistic regressions were performed. Planned enrollment was 336, but the trial was halted early due to discontinuation of detemir in 2024.

**Results**: 73 subjects were recruited (NPH: 38, Detemir: 35). Demographic differences were not noted for most categories, but intake A1c (NPH: 5.7%, IQR 5.5‐6.0%; Det: 6.8%, IQR 6.0‐8.7%; p = 0.021) and race (more Asian/Caucasian in NPH versus more Black in detemir) differed, likely due to early termination of study. No differences were noted in rates of HG (44.4 v 36.4%, p = 0.495) or persistent HG (5.6 v 6.1%, p = 1.0), even after controlling for DM type, preterm birth, BMI and site. No differences were noted for secondary neonatal outcomes (Table 2).  Rates of postpartum hemorrhage were higher for NPH (36.8 v 8.8%, p = 0.005), but no other differences in maternal outcomes were noted, including mode of delivery, HTN, insulin drip, lacerations, or infection (Table 1).

**Conclusion**: No difference in rates of neonatal HG were seen between NPH and detemir groups. This study is limited by early termination of the trial due to discontinued production of detemir. Further studies will need to be performed to compare currently available basal insulins in management of DM in pregnancy.

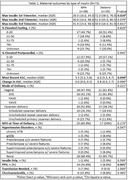

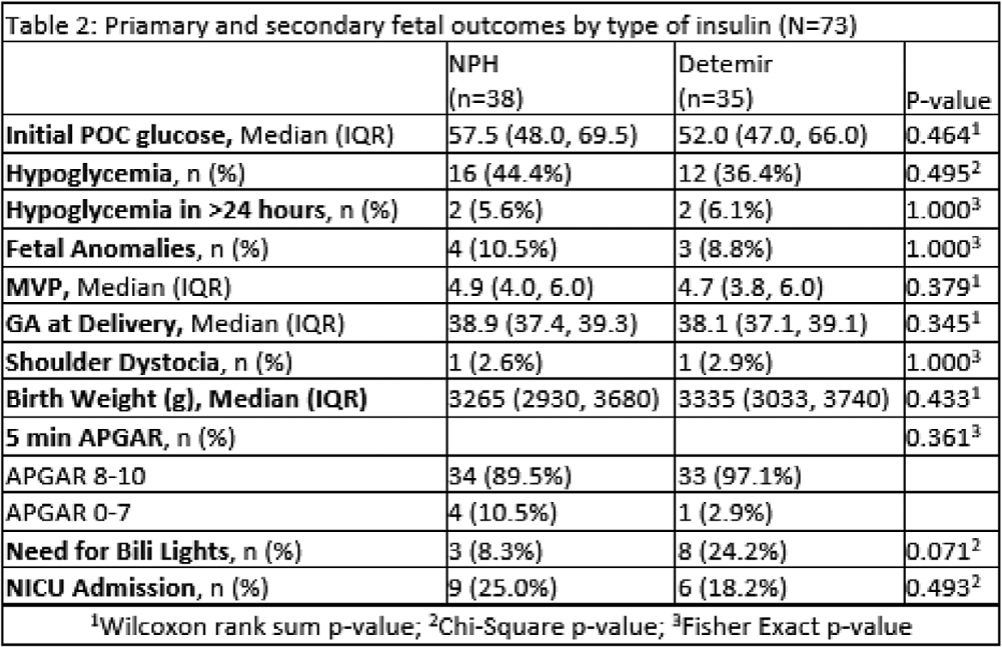

## 854 | Development of a Benchtop Model of Cervical Laceration with Cerclage in Situ

Alyssa L. Trochtenberg^1^; Alexandra Denisevich^2^; Skylar Murphy^2^; Joanna Chase^3^; Mireyda Perez Hernandez^3^; Tim Looney^4^; Walfre Franco^3^; Devon Campbell^5^; Michael House^6^

*^1^Tufts University School of Medicine, Tufts University School of Medicine, MA; ^2^Cx Therapeutics, Lowell, MA; ^3^University of Massachusetts, Lowell, Lowell, MA; ^4^Northeast Biomedical, Tyngsboro, MA; ^5^Prodct, LLC, Lexington, MA; ^6^Tufts Medical Center, Boston, MA*

10:30 AM ‐ 12:30 PM

**Objective**: Our long‐term objective is the development of a novel medical device as a cerclage alternative. Cervical laceration is a known complication of cerclage. One goal of the medical device is to reduce the risk of cervical laceration. However, no benchtop model of cervical laceration exists. Thus, we sought to develop a three‐dimensional (3D) benchtop model to study cervical laceration using a silicone‐based cervix that mimics the stiffness of the human cervix in pregnancy.

**Study Design**:  The silicone cervix model consisted of two parts (see “Cervix” image in Figure). The upper part (blue color) was composed of Smooth‐Sil 950 (Smooth‐On, Macungie, PA). The lower part (the cervix, tan color) was composed of Ecoflex, a soft silicone (Smooth‐On). The silicone cervix formula was selected to match the mechanical properties of human cervical tissue based on pilot studies, as follows: 1‐part Ecoflex 00‐30 Part A; 1‐part Ecoflex Part B; 3‐parts SmoothOn silicone thinner . Ecoflex was first poured into the bottom portion of a 3D printed mold and cured to make the silicone cervix. Next,  Smooth Sil 950 was added to fill the mold. The stiffness of the silicone cervix was tested with the Pregnolia system (Pregnolia, Switzerland) (N = 9). To assess cervical laceration, the silicone cervix was mounted on a test fixture attached to an Instron machine. A 3D printed fetal head (diameter 8.9 cm) was pushed through the silicone cervix (Figure, top right) (N = 6). Outcomes were compared between silicone cervices with no cerclage and with an O‐Vicryl cerclage suture. Silicone tearing was used as an estimate of the risk of cervical laceration

**Results**: The mean (± SD) stiffness of the silicone cervix was 168 ± 17 millibar, which is similar to the stiffness of the human cervix in previously published studies. Silicone cervices without a cerclage had no tearing. However, tearing occurred in all silicone cervices with a cerclage in place (*P* = 0.002).

**Conclusion**:  We designed a benchtop model that reliably simulates cervical laceration, which can be used to study alternate devices for the treatment of cervical insufficiency.

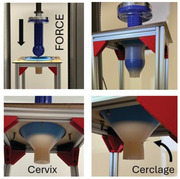

## 855 | The Prevalence of Food Insecurity at Time of Delivery and Associated Peripartum Outcomes

Miriam L. Hernandez‐Zepeda; Monica Rincon; Amy M. Valent; Bharti Garg

*Oregon Health & Science University, Portland, OR*

10:30 AM ‐ 12:30 PM

**Objective**: Food insecurity is the social condition of having limited or uncertain access to adequate food. Nutritional requirements increase in pregnancy, however the impact of food insecurity in pregnancy is not well understood. The objective of this study was to determine the incidence of food insecurity at our institution, and to investigate the relationship between food insecurity and peripartum and neonatal outcomes.

**Study Design**: Prospective cohort study of all pregnant or postpartum people admitted to Oregon Health and Science University from August 2023 to February 2024 to 1) determine the prevalence of food insecurity and 2) compare perinatal outcomes between those that screened positive for food insecurity to those secure. After informed consent, participants filled out the United States Department of Agriculture (USDA) Six‐Item Short Form to screen for food insecurity. Chi‐square and t‐tests were used for statistical analysis.

**Results**: Of the 1273 pregnant people admitted during this study period, 232 were screened for food insecurity and 51 screened positive (22%). Of the 51 people who screened positive, 56.9% received a social work consult, 35.3% declined a social work consult, and 27.5% were eligible but not enrolled in food assistance programs. People with food insecurity were younger (28.8 ± 5.7 vs 33.4 ± 5.1 years old, P < 0.001), not married (31.4% vs 79.6%, P < 0.001), among underrepresented racial/ethnic groups (54.9% vs 28.8%, P < 0.001), have government insurance (80.4% vs 21.0%, P < 0.001), and have higher prenatal EPDS scores (10.4 vs 4.4, P < 0.001). People with food insecurity trended to have higher rates of iron deficiency anemia, higher postpartum EPDS scores, and lower rates of breastfeeding. Neonatal outcomes were similar between both groups.

**Conclusion**: Food insecurity is an important social driver of health that can affect anyone. Understanding the relationship between food insecurity and peripartum and neonatal outcomes as well as individual perceptions to receiving help is critical to addressing inequities that impact health.

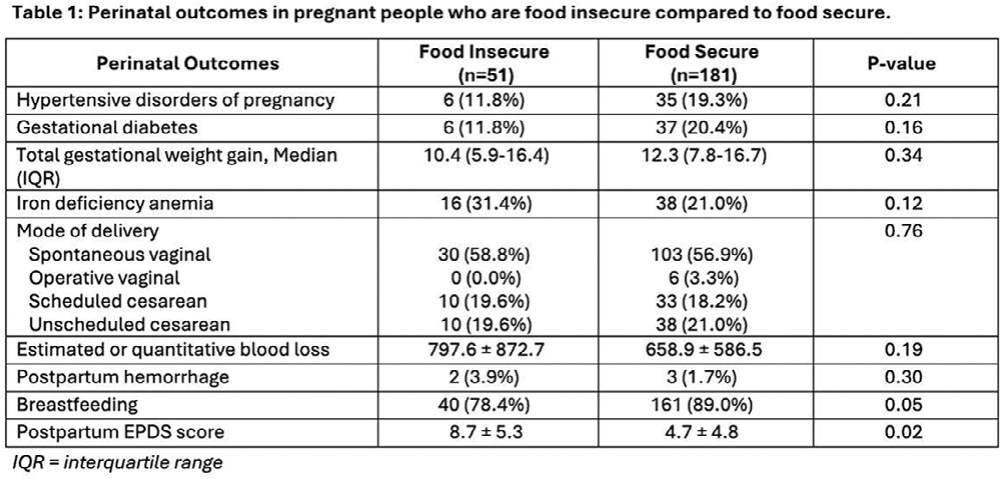

## 856 | Association of Co‐Occurring Chronic Hypertension and Oligohydramnios on Neonatal Outcomes

Miriam L. Hernandez‐Zepeda; Bharti Garg; Alyssa R. Hersh; Kristin C. Prewitt; Aaron B. Caughey

*Oregon Health & Science University, Portland, OR*

10:30 AM ‐ 12:30 PM

**Objective**: Oligohydramnios has been associated with uteroplacental insufficiency and occurs at a higher rate among patients with chronic hypertension. However, the additional risk associated with oligohydramnios and adverse neonatal outcomes has not been well elucidated in this population, which is already at higher risk of adverse outcomes. The objective of this study was to investigate the association between oligohydramnios and chronic hypertension on neonatal outcomes.

**Study Design**: This is a retrospective cohort study comparing pregnant persons with chronic hypertension who had oligohydramnios versus no oligohydramnios. We used a California cohort of singleton, non‐anomalous pregnancies with late preterm or term births (34 to 41 weeks) who delivered between 2008 and 2020. Chi‐squared and multivariable logistic regression models were used for statistical analysis with a p‐value of 0.05.

**Results**: Of the 434,362 pregnant persons with chronic hypertension who met inclusion criteria, 14,867 had oligohydramnios (3.4%). Pregnant persons with chronic hypertension with oligohydramnios were significantly different by race/ethnicity, body mass index, and parity compared to those without oligohydramnios. The rate of preexisting diabetes was similar between groups (4.3% vs 4.0%, P = 0.13). Oligohydramnios was associated with a higher adjusted odds of preterm birth (aOR 1.58; 1.51‐1.65), NICU admission (aOR 1.13; 95% CI 1.07‐1.19), small for gestational age (aOR 2.43; 95% CI 2.34‐2.53), and stillbirth (aOR 1.81; 95% CI 1.05‐3.12).

**Conclusion**: This study demonstrates that oligohydramnios with chronic hypertension is associated with adverse neonatal outcomes. Our study can enhance patient knowledge and provider counseling to mitigate neonatal impacts of co‐occurring diagnoses.

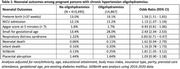

## 857 | Association Between Prenatal Exposure to Environmental Phthalates and Phenols with Adverse Pregnancy Outcomes

Misa Hayasaka^1^; George R. Saade^2^; Tetsuya Kawakita^2^

*^1^Department of Obstetrics and Gynecology, Macon and Joan Brock Virginia Health Sciences at ODU, Norfolk, VA; ^2^Macon & Joan Brock Virginia Health Sciences Eastern Virginia Medical School at Old Dominion University, Norfolk, VA*

10:30 AM ‐ 12:30 PM

**Objective**: To evaluate the association between prenatal exposure to phthalates and phenols with adverse pregnancy outcomes.

**Study Design**: This prospective cohort study used data from the Environmental Influences on Child Health Outcomes (ECHO) consortium, which includes longitudinal data on over 30,000 pregnancies from 69 cohorts across the US. Participants were singleton pregnant individuals with data on urinary chemical concentrations at least one time during pregnancy and pregnancy outcomes. Urinary chemical measurements were performed separately by cohort. Individuals were classified into quartiles of various urinary chemical levels. Outcomes examined included preterm birth (PTB), small for gestational age (SGA), and gestational diabetes mellitus (GDM). Generalized estimating equations models with Poisson distribution and robust variance were used to estimate relative risks (RR) with 95% confidence intervals (95%CI) using the lowest quartile as the reference. All models were adjusted for maternal age, education, race and ethnicity, prepregnancy body mass index, marital status, and study cohorts.

**Results**: Among the 5,749 participants, 543 (9.4%) had PTB, 335 (5.8%) had SGA, and 401 (7.0%) had GDM. Table 1 presents the urinary levels of the chemicals assayed and the number of samples. Models showed that higher quartiles of phthalates and phenols were associated with an increased risk of PTB and SGA, whereas no significant associations were found for GDM (Table 2). The highest risk of PTB was noted with the 4^th^ quartile of phthalic acid (RR 1.67; 95% CI 1.22‐2.28) compared to the 1^st^ quartile. The highest risk for SGA was observed with the 4^th^ quartile of mono‐isononyl phthalate (RR 2.37; 95%CI 1.57‐3.58).

**Conclusion**: Pregnancy exposure to phthalates and phenols, ubiquitous environmental chemicals, could be preventable risk factors for PTB and SGA. Further study is needed to confirm these observations and identify the exposure sources.

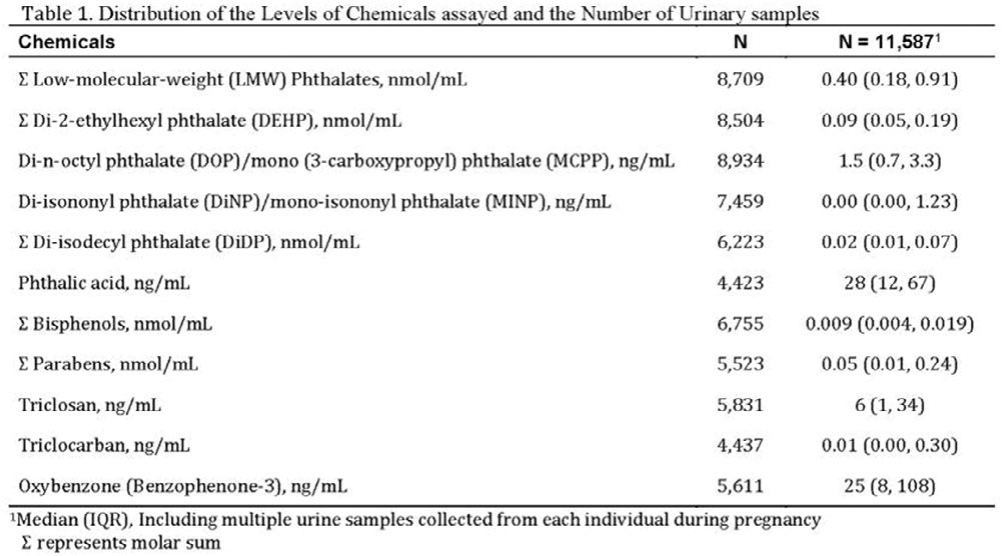

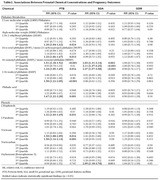

## 858 | Association of Postpartum Hemorrhage Management with Risks of Hysterectomy, Icu Admission, and Severe Maternal Morbidity

Moeun Son^1^; Jennifer F. Culhane^2^; Evan Miller^3^; Sara Handley^4^; Heather Burris^5^; Jay Greenspan^3^; Kathryn M. McKenney^6^; Lindsey Knake^7^; Robert Whetsel^8^; Kristie Baisden^8^; Leah Berhane^8^; Kevin Dysart^3^

*^1^Weill Cornell Medicine, New York, NY; ^2^Yale School of Medicine, New Haven, CT; ^3^Nemours Children's Hospital, Wilmington, DE; ^4^Children's Hospital of Pennsylvania, Philadelphia, PA; ^5^Children's Hospital of Philadelphia, Philadelphia, PA; ^6^University of Colorado, Denver, CO; ^7^University of Iowa, Iowa City, IA; ^8^US Food and Drug Administration, Silver Spring, MD*

10:30 AM ‐ 12:30 PM

**Objective**: To examine if varying management strategies of postpartum hemorrhage (PPH) is associated with hysterectomy, intensive care unit (ICU) admission, and severe maternal morbidity (SMM).

**Study Design**: This retrospective cross‐sectional study utilized Epic Systems’ Cosmos research platform, a US‐based electronic health record database with de‐identified patient‐level data. Individuals with an inpatient birth, PPH ICD‐10‐CM code, and PPH treatment from 1/1/17‐12/31/23, without a placenta accreta spectrum ICD‐10‐CM code were included. Management strategies were categorized into 4 groups: “less aggressive medication use only” (2 types with < 2 doses of either or 1 type with < 3 doses); “more aggressive medication use only” (3 types, 2 types with ≥2 doses of either, or 1 type with ≥3 doses; “less aggressive medication plus procedure(s)”; and “more aggressive medication plus procedure(s)”. Medications included carboprost, methylergonovine, and tranexamic acid. Procedures included insertion of intrauterine device, curettage, and uterine artery embolization. Outcomes were hysterectomy, ICU admission, and SMM with and without transfusion during the birth encounter. Bivariable and multivariable logistic regression analyses were performed.

**Results**: Of 277,585 patients treated for PPH, 84.9% received less aggressive medication use only (Table). There were 1,951 (7 per 1,000) hysterectomies, 6,383 (2.3%) ICU admissions, and 18,474 (6.7%) SMM cases without and 45,238 (16.3%) with transfusion. Patients with PPH procedures regardless of medication use, were more likely to undergo hysterectomy and experience SMM than those with less aggressive medication use only. Compared to patients with less aggressive medication use only, those with aggressive medication use only were significantly less likely to experience SMM without transfusion.

**Conclusion**: Higher odds of hysterectomy and morbidity among patients with PPH procedures could represent delay in definitive treatment or confounding by indication (more severe PPH cases). Either way, these procedures are markers of increased risk for hysterectomy, ICU admission, and SMM.

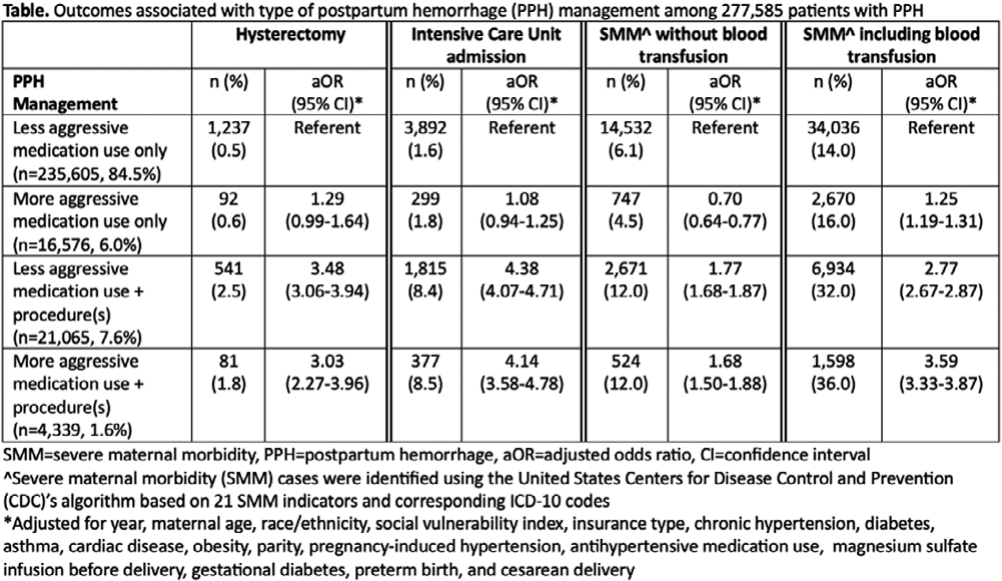

## 859 | The Association Between Umbilical Artery End Diastolic Flow and Growth Velocity in Growth Restricted Fetuses

Mohammad Salameh^1^; Joshua Brunton^2^; Megan E. Branda^1^; Regan Theiler^1^

*^1^Mayo Clinic, Rochester, MN; ^2^Virginia Commonwealth University, Richmond, VA*

10:30 AM ‐ 12:30 PM

**Objective**: Growth velocity is receiving increasing attention as a potential marker of fetal wellbeing. We aimed to investigate the relationship between umbilical artery diastolic flow, an established surveillance tool, and growth velocity.

**Study Design**: We conducted a retrospective cohort analysis of pregnant patients aged 18‐45 years old who delivered between 2012 and 2022. Pregnancies with congenital abnormalities or multiple gestations were excluded. Growth velocity was defined as the difference between estimated fetal weight percentiles at 26‐32 weeks and at 32‐36 weeks divided by the time between ultrasound (percentile change per week). The last systolic to diastolic (S/D) ratio obtained between 32‐36 weeks was chosen as the marker of umbilical artery end diastolic flow. A linear correlation model was used to investigate the relationship between S/D ratio percentile and growth velocity.

**Results**: 324 patients met inclusion criteria, and 74 patients had at least one umbilical artery study between 32‐26 weeks. The range of growth velocity was ‐7.58‐20.40/week (mean = 0.31/week). The range of S/D ratio percentiles was 2.46%‐99.98% (mean = 63.70%); all patients had forward diastolic flow. There was a negative correlation between S/D ratio percentile and growth velocity with r = ‐0.31 (p< 0.01). This is shown in Figure 1.

**Conclusion**: Decreasing umbilical artery diastolic flow correlated with fetal growth deceleration over the weeks preceding the umbilical artery doppler exam. Additional studies are needed to determine whether using growth velocity combined with umbilical artery doppler studies improves prediction of fetal compromise and adverse neonatal outcomes.

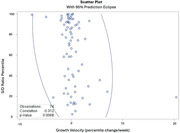

## 860 | Leveraging Deep Neural Networks of Transvaginal Cervical Characteristics to Predict Delivery Timing

Peinan Zhao^1^; Joshua Goyert^2^; Methodius G. Tuuli^3^; Adam K. Lewkowitz^4^; Julie Tumbarello^5^; Emily Dively, BSN^6^; Madeline Felske^5^; Wendy Sparks^5^; Giselle Kolenic^2^; Molly J. Stout^7^

*^1^Washington University, St. Louis, MO; ^2^University of Michigan, Ann Arbor, MI; ^3^Women & Infants Hospital of Rhode Island / Alpert Medical School of Brown University, Providence, RI; ^4^Women & Infants Hospital of Rhode Island / Alpert Medical School of Brown University., Providence, RI; ^5^University of Michigan Hospital, Ann Arbor, MI; ^6^Washington University School of Medicine, St. Louis, MO; ^7^University of Michigan Medical Center, Ann Arbor, MI*

10:30 AM ‐ 12:30 PM

**Objective**: To utilize deep neural networks of biomechanical characteristics obtained from transvaginal ultrasound videos to predict timing of delivery within 7 days at term.

**Study Design**: This is a prospective longitudinal cohort of patients who underwent fully quantitative cervical elastography (FQ‐CES) starting at 37 weeks and weekly thereafter until delivery. FQ‐CES quantifies cervical tissue stiffness utilizing a transvaginal ultrasound probe to quantify both pressure applied and tissue deformation to allow operator independent comparisons. Singleton pregnancies at 37 weeks without delivery planed before 39 weeks were enrolled. FQ‐CES and cervical length (CL) were collected weekly until delivery. Patients remained enrolled regardless of their eventual timing or indication for delivery. Transvaginal ultrasound videos from FQ‐CES exams were used to build a deep convolutional neural network with *Xception* architecture. The primary outcome is delivery within 7 days.

**Results**: 118 individuals with 241 FQ‐CES ultrasound exams were included. 80% of data was randomly selected as training dataset with a 5‐fold cross‐validation split. Among 241 FQ‐CES exams, 102 (42.3%) delivered  ≤7 days and 139 (57.7%) delivered >7 days. Frames from ultrasound videos were treated as individual image inputs. For multiple image predictions inside one FQ‐CES exam, majority vote method was applied. The deep convolutional neural network showed high predictive performance including high 92% sensitivity, 90% specificity, and accurate prediction of delivery within 7 days in 91% of the population. In contrast, the network utilizing CL < 2cm showed lower prediction performance (Table)

**Conclusion**: Deep learning models based on FQ‐CES videos have much higher predictive performance for delivery within 7 days. This may be due to the ability to capture characteristics related to anatomy, biomechanical properties, and cervical tissue interaction with surrounding tissues. This technique could be be leveraged to capture intricate cervical characteristics to predict delivery timing.

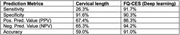

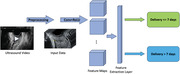

## 861 | Vaccine Perceptions Among Hispanic Pregnant and Lactating Individuals During the COVID‐19 Vaccine Introduction

Monica Sosa; Mindy Pike; Porshia Underwood; Carolina Martinez‐King; Rjika Lottes; Janet A. Englund; Linda Eckert; Alisa B. Kachikis

*University of Washington, Seattle, WA*

10:30 AM ‐ 12:30 PM

**Objective**: To investigate vaccine perceptions and hesitancy of Hispanic pregnant and lactating individuals in the setting of the COVID‐19 vaccination implementation.

**Study Design**: An online prospective cohort study of people who were pregnant, lactating, or planning pregnancy launched in January 2021 shortly after approval of the COVID‐19 vaccination. Race and ethnicity data were collected using the Centers for Disease Control and Prevention's National Health Interview Survey categories. Participants in this IRB‐exempt study completed surveys via REDCap online including one on vaccine perceptions. We performed statistical analysis using Chi‐square tests and Fisher's exact tests for categorical variables and t‐tests and Wilcoxon rank sum tests for normal and non‐normal continuous variables, respectively.

**Results**: Among the 26,322 participants, 1,618 identified as Hispanic and 24,704 as non‐Hispanic. Most participants were well‐educated (94.0%) and worked in healthcare (53.0%). Hispanic participants were less likely to gain knowledge about vaccines from physicians compared to non‐Hispanic participants (n = 1,190, 73.6%; n = 19,020, 77.0%; p = 0.001) and reported using the internet (n = 266, 16.4%; n = 3,512, 14.2%; p = 0.01), social media (n = 105, 6.5%; n = 1.201, 4.9%; p = 0.003) and radio (n = 65, 4.0%; n = 763, 3.1%; p = 0.04) more than non‐Hispanic participants. Compared to non‐Hispanics, Hispanics trusted physicians less (p = 600, 37.8%; p = 10,104, 41.6% p = 0.002) and medical literature more (p = 366, 23.0%; p = 4,696, 19.4%; p = 0.002) regarding knowledge on vaccines. On average Hispanic participants reported a lower understanding on which vaccines they and their children should receive (p< 0.001) however no difference noted regarding understanding on how vaccines work or vaccine schedules (p = 0.81 and p = 0.11, respectively) compared to non‐Hispanic participants.

**Conclusion**: When compared to non‐Hispanic people, Hispanic participants report a greater use of non‐traditional methods to gain vaccines knowledge and report trusting their physicians less and medical literature more on vaccines.  

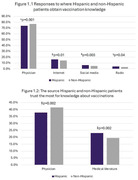

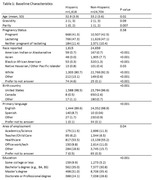

## 862 | The Impact of Antepartum Fibroid Characteristics on Severe Maternal Morbidity

Moti Gulersen^1^; David Krantz^2^; Burton Rochelson^2^; Matthew J. Blitz^2^

*^1^Sidney Kimmel Medical College of Thomas Jefferson University, Philadelphia, PA; ^2^Northwell, New Hyde Park, NY*

10:30 AM ‐ 12:30 PM

**Objective**: To evaluate the association between uterine fibroids and their characteristics (e.g. number, size) with severe maternal morbidity (SMM).

**Study Design**: Multicenter retrospective cross‐sectional study of all singletons who had a prenatal ultrasound at ≥ 18 weeks and delivered from 2019‐2023. Fibroid characteristics were documented when identified on ultrasound. Patients with >1 delivery during the study period had only their first included for analysis. The primary outcomes were SMM, defined based on the Centers for Disease Control and Prevention list of 21 indicators, and non‐transfusion SMM. Both were assessed based on the presence of any fibroid, fibroid number (1, 2, ≥3), and size (largest dimension < 5cm, 5‐10cm, >10cm), compared to patients with no fibroids. Multivariate logistic regression was performed to adjust for maternal race/ethnicity and obstetric comorbidity index, a validated screening tool used to predict SMM. Data were presented as adjusted odds ratios (aOR) with 95% confidence intervals (CI).

**Results**: Of the 79,802 patients included, 4,756 (6.0%) had at least one fibroid. The prevalence of SMM and non‐transfusion SMM were 4.3% and 1.1%, respectively. Outcomes are displayed in the Table. The presence of any fibroid, regardless of number or size, was associated with an increased risk of SMM. There was no statistically significant association between fibroids and non‐transfusion SMM. Patients with more and larger fibroids were at higher risk of SMM compared to those without fibroids. Fibroid number and size did not impact the risk of non‐transfusion SMM. After adjusting for fibroid number and size, patients with fibroids in the lower uterine segment or cervix were at higher risk of SMM compared to those without fibroids in that location (aOR 1.48, 95% 1.12‐1.96).

**Conclusion**: The association between fibroids and their characteristics with risk of SMM is likely related to hemorrhage. Providers should consider these risks and interventions to help reduce such risk when caring for patients with fibroids during pregnancy and their delivery.

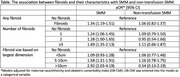

## 863 | The Impact of Antenatal Corticosteroids on Severe Neonatal Morbidity in Preterm Births

Moti Gulersen^1^; Frank I. Jackson^2^; Ashley N. Battarbee^3^; Vincenzo Berghella^1^; Matthew J. Blitz^2^

*^1^Sidney Kimmel Medical College of Thomas Jefferson University, Philadelphia, PA; ^2^Northwell, New Hyde Park, NY; ^3^Center for Women's Reproductive Health, University of Alabama at Birmingham, Birmingham, AL*

10:30 AM ‐ 12:30 PM

**Objective**: Studies evaluating the impact of antenatal corticosteroids (ACS) have typically included a wide gestational age (GA) range of preterm births. We aimed to evaluate whether antenatal corticosteroid (ACS) exposure is associated with a decreased risk of severe neonatal morbidity (SNM) in the periviable, early, and late preterm periods.

**Study Design**: Retrospective cohort study of all singleton preterm births (PTBs) between 22 0/7‐36 6/7 weeks’ gestation at 2 tertiary academic medical centers from 2019‐2022. Patients who had neonates that were not actively resuscitated at birth, intrauterine fetal demise, and missing data were excluded. The primary outcome of SNM, a standardized composite neonatal adverse outcome indicator which includes diagnoses and procedures from the neonatal intensive care unit indicative of severe morbidity, was compared between PTBs exposed to ACS prior to delivery and those unexposed. We compared SNM by ACS exposure stratified by 4 gestational age of delivery groups: 22 0/7‐25 6/7, 26 0/7‐29 6/7, 30 0/7‐33 6/7, and 34 0/7‐36 6/7 weeks of gestation. Multivariate logistic regression was performed to adjust for potential confounders. Data were presented as adjusted odds ratios (aOR) with 95% confidence intervals (CI).

**Results**: Of the 5,070 preterm births included, 2,456 (48.4%) were exposed to ACS prior to delivery. ACS was associated with a lower odds of SNM among PTBs occurring at 22 0/7‐25 6/7 weeks of gestation, but the association at 26 0/7‐29 6/7 and 30 0/7‐33 6/7 weeks was not statistically significant (Table). Among late PTBs (34 0/7‐36 6/7 weeks), ACS exposure was associated with an increased risk of SNM compared to those unexposed (Table).

**Conclusion**: Our data suggest that the effect of ACS on neonatal outcomes varies by GA at delivery with improved outcomes at periviable GA and worse outcomes in the late preterm period. This differential effect should be considered when counseling patients and deciding if and when to administer ACS.

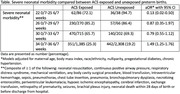

## 864 | Preterm Labor with Intact Membranes: the Differential Diagnosis Between Intraamniotic Infection and Sterile Intraamniotic Inflammation

Mousa Eissa^1^; Roberto Romero^2^; David R. Bryant^3^; Fatima Ali^4^; Arun Mezzayhagan^3^; Tinnakorn Chaiworapongsa^3^

*^1^Detroit Medical Center, Detroit, MI; ^2^Pregnancy Research Branch, Division of Obstetrics and Maternal‐Fetal Medicine, Division of Intramural Research, Eunice Kennedy Shriver NICHD, NIH, DHHS, Bethesda, Maryland, USA, Bethesda, MD; ^3^Wayne State University School of Medicine, Detroit, MI; ^4^Detroit Medical Center/Wayne State University, Detroit, MI*

10:30 AM ‐ 12:30 PM

**Objective**: Preterm labor (PTL) is a syndrome caused by multiple pathologic processes. Intraamniotic inflammation is a major cause of preterm birth and neonatal morbidity/mortality. The two causes of intraamniotic inflammation [intraamniotic infection (IAI) and sterile intraamniotic inflammation (S‐IAI)] can be treated in humans. The optimal test to identify intraamniotic inflammation is amniotic fluid (AF) interleukin (IL)‐6. This study was designed to determine if AF IL‐6 concentration can be used in the differential diagnosis between IAI and S‐IAI.

**Study Design**: The value of AF IL‐6 was tested in two independent cohorts of patients with PTL (20–35 weeks of gestation). AF was analyzed for microorganisms using culture and PCR. IL‐6 concentration was determined by ELISA. IAI was diagnosed by the combination of bacteria and inflammation (AF IL‐6 ≥ 2.6 ng/ml). S‐IAI was diagnosed if inflammation was present without microorganisms. ROC analysis was used from the first cohort (n = 142) to identify patients with IAI. The results were tested in a second cohort (n = 166).

**Results**: 1) Patients with IAI had the highest AF IL‐6 concentrations among the groups (Figure); 2) an AF IL‐6 ≥ 28.9 ng/ml had a sensitivity of 87% and a specificity of 97% to identify patients with IAI [AUC 0.97 (95% CI, 0.94–1.00)]; 3) an AF IL‐6 ≥ 9 ng/ml identified all patients with IAI with a false‐positive rate of 19%; 4) all patients with an AF IL‐6 concentration between 2.6 and 9 ng/mL were diagnosed with S‐IAI, and this IL‐6 interval identified 38.5% (15/39) of patients with S‐IAI; and 5) results were replicated in the second cohort. AF IL‐6 ≥ 28.9 ng/ml had a sensitivity of 83% and a specificity of 83% for IAI. AF IL‐6 ≥ 9 ng/ml identified all patients with IAI, and IL‐6 between 2.6 and 9 ng/ml identified 39.5% with sterile intraamniotic inflammation.

**Conclusion**: AF IL‐6 concentration can be used for the rapid assessment and diagnosis of IAI and S‐IAI.

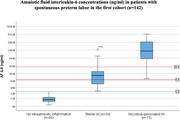

## 865 | Placental Pathology in Nulliparas with Gestational Hypertension and Preeclampsia

Nadine Sunji^1^; Melodee Liegl^1^; Amy Y. Pan^1^; Alexa A. Freedman^2^; Linda M. Ernst^3^; Anna Palatnik^1^

*^1^Medical College of Wisconsin, Milwaukee, WI; ^2^Northwestern University, Chicago, IL; ^3^Endeavor Health, Evanston, IL*

10:30 AM ‐ 12:30 PM

**Objective**: To investigate placental pathology patterns in first‐time mothers with gestational hypertension and preeclampsia.

**Study Design**: This was a secondary analysis of the Nulliparous Pregnancy Outcomes Study: Monitoring Mothers‐to‐be. The present analysis included nulliparous participants with singleton gestations who developed gestational hypertension or preeclampsia and had placental pathology reports available. Placental characteristics and pathologies were described and compared between different types of hypertensive disorders of pregnancy (HDP) and by gestational age at HDP diagnosis using univariate and bivariate analyses.

**Results**: Of 543 participants who met inclusion criteria, 297 (54.7%) had gestational hypertension, and 246 (45.3%) had preeclampsia. Of these, 228 (42.3%) had an HDP diagnosis prior to 37 weeks. The rate of acute fetal and maternal placental inflammation was higher among those with term preeclampsia (46.2%) and gestational hypertension (42.1%) and lower among placentas of preterm preeclampsia (12.6%, p< .001; Table 1). In contrast, the rate of maternal vascular malperfusion was highest in preterm preeclampsia (62.1%) and lowest in gestational hypertension (29.6%, p< .001). The rates of fetal vascular malperfusion did not differ between HDP phenotypes. Table 2 examined placental findings by gestational age at diagnosis of ≥37 weeks, 34‐36 weeks, and < 34 weeks. Presence of acute maternal and fetal inflammatory response increased with advancing gestation (19.5% at < 34 weeks, 23.8% at 34‐36 weeks, and 49.8% at ≥37 weeks; p< .001) while the prevalence of maternal vascular malperfusion decreased with advancing gestation (48.8% at < 34 weeks, 44.8% at 34‐36 weeks, and 31.2% at ≥37 weeks; p< .001).

**Conclusion**: Maternal vascular malperfusion lesions in the placenta were more common in early‐onset HDP while acute inflammatory lesions were more common in late‐onset HDP. This distinction helps in understanding that early‐ and late‐onset HDP may have different underlying pathological mechanisms.

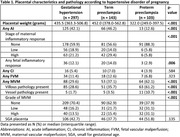

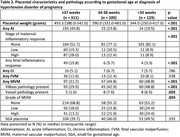

## 866 | Insomnia is an Independent Risk Factor for Severe Morbidity During Pregnancy

Naima E. Ross^1^; Rebecca J. Baer^2^; Scott P. Oltman^2^; Dana R. Gossett^3^; Rashmi N. Aurora^3^; Laura L. Jelliffe‐Pawlowski^3^; Justin S. Brandt^3^

*^1^NYU Grossman School of Medicine, New York, NY; ^2^University of California San Francisco School of Medicine, San Francisco, CA; ^3^NYU Langone Health, New York, NY*

10:30 AM ‐ 12:30 PM

**Objective**: Sleep disorders, such as insomnia and obstructive sleep apnea (OSA), are associated with pregnancy complications. In this study, we aimed to determine the impact of insomnia on the risk of severe morbidity (SM) and to compare the magnitude of the associations between insomnia/OSA and SM.  

**Study Design**: We performed a cross‐sectional study of liveborn singleton births in California (2011‐2020). Birth certificates were linked to hospital discharge records for birthing people and their infants. Insomnia and OSA were identified as exposures using ICD‐9 and 10 codes. The primary outcome was SM, as defined by the US Centers for Disease Control and Prevention. Secondary outcomes included components of SM. The relative risks, and corresponding 95% confidence intervals (CI), were calculated for outcomes by sleep disorder using Poisson regression models.

**Results**: During the study period, there were 4,145,166 singleton live births with linked records. The prevalence of insomnia and OSA were 117.4 and 138 per 1000 live births, respectively. Among people with sleep disorders, only 72 had both diagnoses (0.7%). Compared to people with no sleep disorders (reference), a higher percentage of people with sleep disorders were age >35 years, White, privately‐insured, BMI >30 kg/m^2^, and had cesarean deliveries. The RR of SM was 3.4 fold higher (95% CI 3.1, 3.8) and 4.3 fold higher (95% CI 3.9, 4.6) for those with insomnia and OSA, respectively (Table). This risk of SM was even greater for non‐transfusion SM. The magnitude of risk for disseminated intravascular coagulation, puerperal cerebrovascular disorders, sepsis, shock, and hysterectomy was slightly higher for patients with insomnia versus those with OSA (Table). The magnitude of risk for all other components of SM was greater in OSA versus insomnia.

**Conclusion**: While both insomnia and OSA confer an increased risk for SM, they are associated with distinct demographic traits. Further study is need to identify baseline patient characteristics associated with each of these sleep disorders during pregnancy and to design targeted preventative interventions.

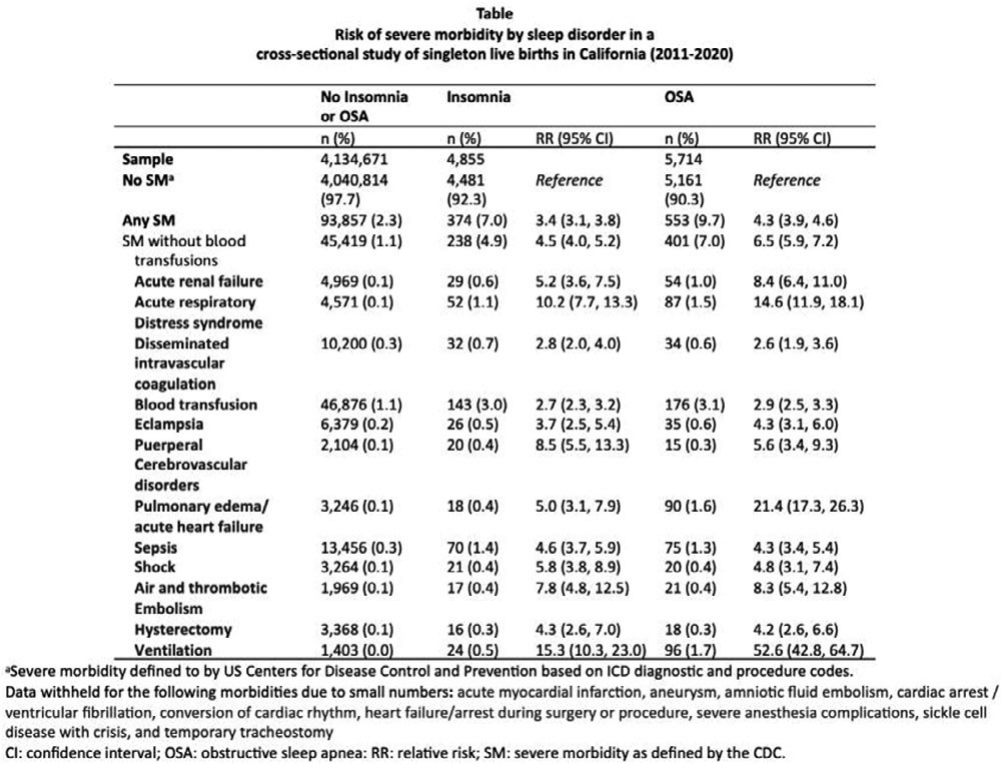

## 867 | Maternal Trauma in Pregnancy ‐ How Bad Are the Consequences?

Nata T. Willner^1^; Nitza Newman^2^; Tamar Wainstock^3^; Eyal Sheiner^3^

*^1^Soroka University Medical Center, Soroka University Medical Center, HaDarom; ^2^Soroka University Medical Center, Be'er Sheva, HaDarom; ^3^Department of Obstetrics and Gynecology, Soroka University Medical Center, Ben‐Gurion University of the Negev, Beer‐Sheva, Israel., Beer Sheva, HaDarom*

10:30 AM ‐ 12:30 PM

**Objective**: Abdominal trauma during pregnancy may compromise placental perfusion and fetal wellbeing. This study aimed to assess perinatal outcomes following abdominal trauma due to motor vehicle accidents (MVA) and falls.

**Study Design**: This population‐based cohort study included all pregnant women presenting to a tertiary‐care center emergency room between the years 1991‐2021. Trauma victims were categorized as vehicle occupant MVA, pedestrian MVA, or following a fall. Comorbidities and pregnancy outcomes were compared between patients with and without trauma. Generalized estimation equation (GEE) models were used to control for confounders such as maternal age and gravidity.

**Results**: A total of 356,356 births were recorded, including 66 (0.02%) following vehicle occupant MVA, 46 (0.01%) following pedestrian MVA, and 142 (0.04%) following a fall.  Placental abruption, non‐reassuring fetal heart rate (NRFHR), preterm delivery and perinatal mortality were all more common among trauma patients. After controlling for maternal age and gravidity, using GEE models, abdominal trauma was significantly associated with placental abruption (adjusted OR 21.7 for vehicle occupant MVA, adjusted OR 4.7 for pedestrian MVA, OR 2.5 for fall), NRFHR (adjusted OR 3.6 for vehicle occupant MVA, adjusted OR 2.6 for pedestrian MVA, adjusted OR 1.6 for fall), and preterm delivery (adjusted OR 4.1 for vehicle occupant MVA, adjusted OR 1.2 for pedestrian MVA, adjusted OR 2.8 for fall) as compared to non‐trauma parturients. Perinatal mortality was higher among vehicle occupant MVA (adjusted OR 9.8) and fall (adjusted OR 4.0) than among non‐trauma parturients (Table).

**Conclusion**: Abdominal trauma during pregnancy is an independent risk factor for adverse perinatal outcomes. The greatest risk seems to be among occupants of a vehicle involved in a collision.

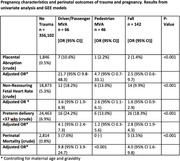

## 868 | Fetal Pleural Effusions: Natural History and Response to Interventions

Natalie B. Gulrajani^1^; Teresa N. Sparks^2^; Juan Gonzalez^2^

*^1^University of California, San Francisco, School of Medicine, San Francisco, CA; ^2^Center for Maternal‐Fetal Precision Medicine, University of California San Francisco, San Francisco, CA*

10:30 AM ‐ 12:30 PM

**Objective**: Primary fetal pleural effusions (FPEs) require close surveillance, and thoracentesis or thoracoamniotic shunts are often considered for management. However, it can be challenging to predict which FPEs will evolve or respond to interventions. We aimed to analyze the prenatal course of primary FPEs and response to interventions.

**Study Design**: Retrospective cohort study of pregnancies with primary fetal FPEs referred to our center between 2017‐2024. We included isolated unilateral FPEs or asymmetric bilateral FPEs with one side dominant. All ultrasound reports and records were reviewed for the course of the FPEs, other complications, fetal interventions, and relevant test results. Each case was categorized as improved or resolved FPE, stable FPE, or worsened FPE (including progression to non‐immune hydrops fetalis).

**Results**: Among 24 cases identified, 9 underwent thoracentesis and/or thoracoamniotic shunt. Median gestational age at diagnosis and first intervention was 19.4 and 20.7 weeks, respectively, for cases with an intervention. A majority (60%) of cases without intervention improved or remained stable (Table). All 9 cases with intervention had additional markers of severity (i.e. mediastinal shift or ascites), compared to only 53% (8/15) without intervention. Each case with an intervention had a thoracentesis, 44% (4/9) had more than one thoracentesis, and 33% (3/9) later had thoracoamniotic shunt placement (Fig). FPEs were chylous in 63% (5/8) of sampled cases; 60% (3/5) of chylous and all (3/3) serous effusions improved after intervention. All cases had a negative viral workup, and among 22 cases that underwent genetic screening and 13 that had diagnostic testing including exome sequencing, only 1 case was identified to have fetal trisomy 21.

**Conclusion**: Many primary FPEs improved or resolved spontaneously without intervention, and genetic diagnoses were uncommon. Most cases requiring intervention responded to thoracentesis and few ultimately required a thoracoamniotic shunt. These data are useful for anticipating the course of and response to interventions among these complicated pregnancies.

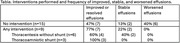

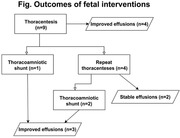

## 869 | The Correlation of Antenatal Sonographic Parameters and Neurodevelopmental Outcomes in Fetal Growth Restriction

Natalie T. Simon^1^; Roopjit K. Sahi^2^; Emma EH Peek^3^; John Hobbins^2^; Manesha Putra^2^

*^1^University of Colorado, Denver, CO; ^2^University of Colorado Anschutz Medical Campus, Aurora, CO; ^3^University of Colorado, Aurora, CO*

10:30 AM ‐ 12:30 PM

**Objective**: To determine how different antenatal sonographic parameters correlate with early childhood neurodevelopmental outcomes in fetuses categorized as fetal‐growth restricted (FGR) or small‐for‐gestational‐age (SGA).

**Study Design**: This was a prospective cohort of singleton FGR or SGA fetuses diagnosed after 20 weeks of gestation. FGR and SGA were defined using Delphi criteria. Using abdominal circumference (AC), umbilical vein flow (UVF) percentiles by gestational age (GA), corpus callosum length (CCL MoM), corpus vermian height (CVH MoM), transverse cerebellar diameter (TCD MoM), and the ratio of the insula circumference to head circumference (IC) from the last ultrasound before delivery, we tested for correlations to neurodevelopmental outcomes. We evaluated neurodevelopmental outcomes using the Bayley‐III Scales of Infant Development (BSID). Mann‐Whitney U tests compared the measurements and scales between FGR and SGA. Pearson correlation was used to compare the sonographic parameters to the BSID subscale and sum percentile scores. Using significant ultrasound parameters as predictors, a multivariate linear regression model was created for each BSID scale as a dependent variable.

**Results**: A total of 90 pregnancies were included in the study. BSID scores were available for 32 infants. SGA fetuses had higher UVF (p = 0.001) and higher motor scores (p = 0.014) compared to FGR (Table 1). EFW and AC centile correlate with motor scores, UVF correlates with language, adaptive behavior, and sum scores, and IC correlates with adaptive behavior. In the multivariate model, EFW was excluded given collinearity with AC.  In the final multivariate analyses, AC was the only significant predictor for motor, UVF was a significant predictor for language, social‐emotional, and sum scores, while insula predicted adaptive behavior and sum score (Table 2).

**Conclusion**: AC, UVF, and IC may be useful predictors of neurodevelopmental outcomes of the growth‐restricted fetus. Long‐term follow‐up studies with larger cohorts are needed to better evaluate the impact of antenatal sonographic parameters on later neurodevelopment.

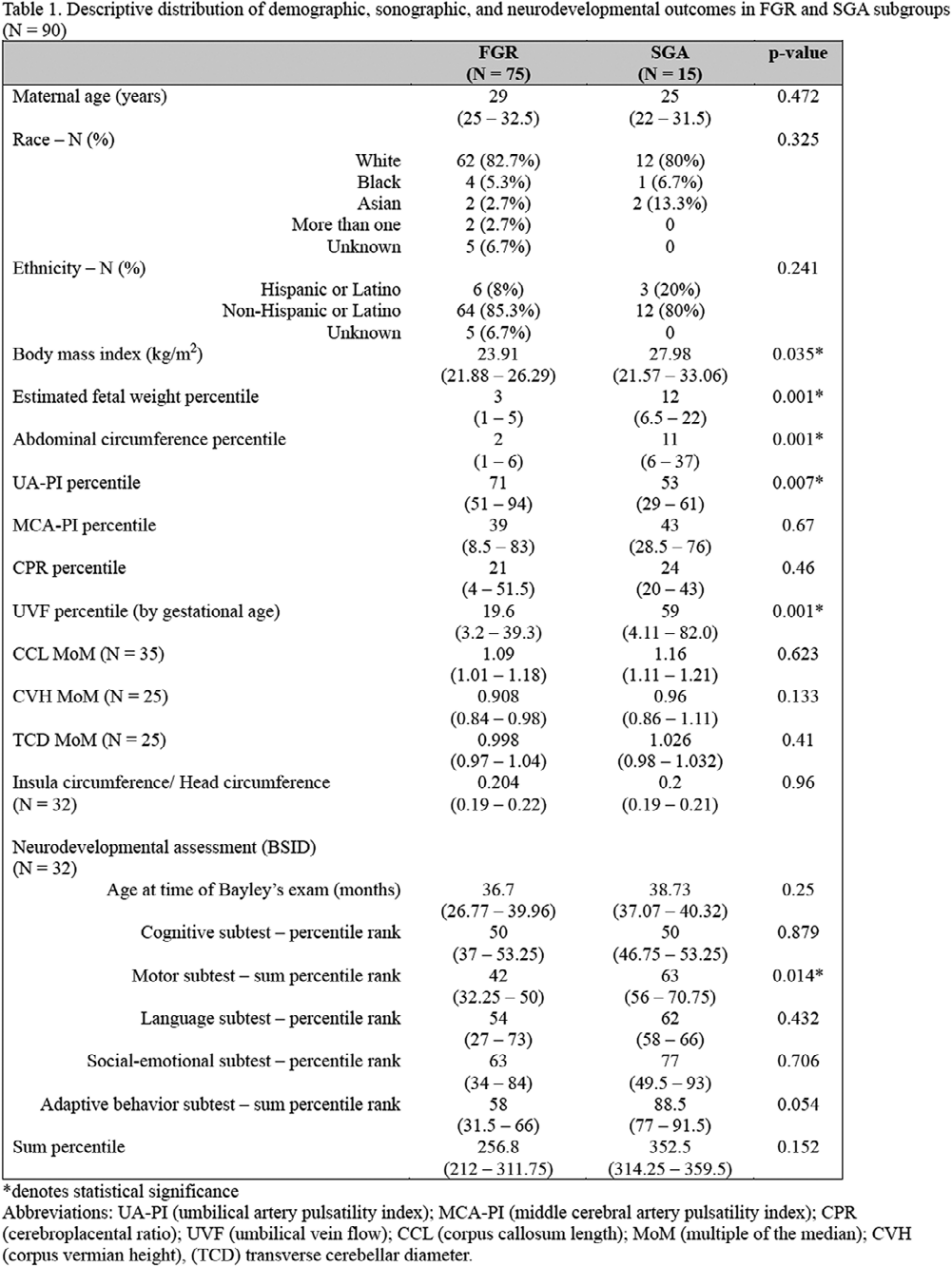

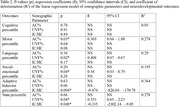

## 870 | Impact of Pregnancy Intention on Immediate Pregnancy Outcomes and Postpartum Mental Health

Nehaa Khadka^1^; Vicki Y. Chiu^2^; Michael J. Fassett^3^; Darios Getahun^2^

*^1^Kaiser Permanente Southern California, Kaiser Permanente Southern California, CA; ^2^Kaiser Permanente Southern California, Department of Research & Evaluation, Kaiser Permanente Southern California/Pasadena, CA; ^3^Kaiser Permanente West Los Angeles Medical Center, Department of Obstetrics & Gynecology, Kaiser Permanente West Los Angeles Medical Center/Los Angeles, CA*

10:30 AM ‐ 12:30 PM

**Objective**: To estimate rates of pregnancy intention, describe their characteristics, and examine pregnancy and postpartum mental health outcomes among intended vs. unintended pregnancies using electronic health records (EHRs) of pregnant patients in a large integrated healthcare system.

**Study Design**: We conducted a retrospective cohort study using EHRs on singleton pregnancies delivered at ≥20 weeks in Kaiser Permanente Southern California (KPSC) healthcare system (01/01/2014‐12/31/2023). Data on pregnancy intentions (wanted, mistimed, unwanted) were extracted from a self‐reported questionnaire administered at the first prenatal visit and in combination with an ICD‐10 code (Z64.0) for unintended pregnancy. We extracted postpartum mental health outcomes using diagnostic codes. Multivariable logistic regression models were used to estimate adjusted odds ratios (OR) and 95% confidence intervals (CI).

**Results**: Of 149,739 who had a record of pregnancy intentions, 34% (n = 51,262) were intended and 66% (n = 98,477) were unintended. Young age, lower socioeconomic status, and late/no prenatal care initiation were more common among unintended pregnancies. This group had higher rates of smoking (5.6% vs 2.6%) and illicit drug use (7.9% vs 3.5%) during pregnancy than those with intended. Unintended pregnancy was associated with higher odds of (pre)eclampsia (OR: 1.07, 95% CI: 1.02, 1.12), and lower odds of gestational diabetes (OR: 0.91, 95% CI: 0.87, 0.94), placenta previa (OR: 0.93, 95% CI: 0.88, 0.98) and cesarean delivery (OR: 0.93, 95% CI: 0.91, 0.96). Additionally, those with unintended pregnancies had higher odds of postpartum depression (OR: 1.24, 95% CI: 1.20, 1.28) and anxiety disorders (OR: 1.19, 95% CI: 1.15, 1.23)

**Conclusion**: Among completed records, unintended pregnancies were over half and independently associated with (pre)eclampsia and postpartum mental health outcomes. Pregnancy intention has a positive influence on selected birth outcomes. Findings suggest that mental health assessments and integrated services supporting high‐risk pregnancy management are vital in improving pregnancy outcomes.

## 871 | Early Vs. Late Placement of Cervical Cerclage Performed due to Cervical Insufficiency in Twins

Neriya Zion Yohay^1^; Tamar Wainstock^2^; Zehava Yohay^3^; Debi Elharar^2^; Talia Birenstock^4^; Guy Elharar^5^; Eyal Sheiner^2^

*^1^Department of Military Medicine and “Tzameret”, Faculty of Medicine, Hebrew University of Jerusalem and Medical Corps, Israel Defense Forces, Jerusalem, Israel, Beer Sheva, HaDarom; ^2^Department of Obstetrics and Gynecology, Soroka University Medical Center, Ben‐Gurion University of the Negev, Beer‐Sheva, Israel., Beer Sheva, HaDarom; ^3^Pain Unit, Soroka University Medical Center, Beer Sheva, Israel, Beer Sheva, HaDarom; ^4^Department of Public Health, Faculty of Health Sciences, Ben‐Gurion University of the Negev, Beer‐Sheva, Israel., Beer Sheva, HaDarom; ^5^Sackler Faculty of Medicine, Tel Aviv university., Beer Sheva, HaDarom*

10:30 AM ‐ 12:30 PM

**Objective**: The study was aimed to assess the impact of early vs. late (above 15 weeks gestation) cervical cerclage placement performed due to cervical insufficiency in twins on perinatal outcome.

**Study Design**: A retrospective cohort study was conducted including all patients with twin pregnancy (n = 92 women) with cervical cerclage. Deliveries occurred between the years 1990 and 2023 in a tertiary medical center. Perinatal outcome was compared between women who underwent cervical cerclage placement ≤15 weeks and women who underwent cervical cerclage placement >15 weeks. Adverse perinatal outcome was defined as either 5 minute Apgar scores < 7 or neonatal intensive care unit (NICU) admission of at least one of the twins. Multiple logistic regression models were used to control for confounders.

**Results**: A total of 92 women were included in the study, complete data was available for 77 women. Late placement of cervical cerclage (>15 weeks, n = 34), was found as a risk factor for PTD less than 35 weeks (79.4% vs. 58.1%, OR 2.77, 1.01–8.33; P = 0.048). No significant association was noted between late cerclage placement due to cervical insufficiency in twins and adverse perinatal outcome (29.4% vs. 15.6%, OR 2.5, 0.75–7.14; P = 0.138) as compared with early placement (Table). Using multivariable analysis, controlling for maternal age, late cerclage placement was noted as an independent risk factor for PTD < 35weeks gestation (adjusted OR 2.94, 95% CI 1.02 ‐ 8.33; P = 0.047).

**Conclusion**: Late placement of cervical cerclage (>15 weeks) in twin pregnancies, performed due to cervical insufficiency, is an independent risk factor for preterm delivery of less than 35 weeks gestation. Nevertheless, in our population, late vs. early placement of cerclage is not a risk factor for adverse perinatal outcome.

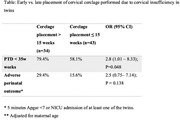

## 872 | Decreasing Trend of Gastroschisis Prevalence in the United States between 2014 and 2022

Nikan Zargarzadeh^1^; May Abiad^2^; Kevin Moss^3^; Erin Perrone^4^; Brian W. Gray^5^; Terry Buchmiller^6^; Kjersti M. Aagaard^7^; Alireza A. Shamshirsaz^8^; Hiba J. Mustafa^9^

*^1^Boston Children's Hospital, Harvard Medical School, Boston, MA; ^2^Maternal Fetal Care Center, Boston Children's Hospital, Boston, MA; ^3^Department of Biostatistics and Health Data Science, Indiana University School of Medicine, IN, USA, Department of Biostatistics and Health Data Science, Indiana University School of Medicine, IN, USA, IN; ^4^Department of Pediatric Surgery, University of Michigan, C.S Mott's Children's Hospital, MI, USA, Ann Arbor, MI; ^5^Department of Pediatric Surgery, Indiana University School of Medicine, Indianapolis, IN; ^6^Department of Pediatric Surgery, Boston Children's Hospital, Harvard Medical School, Boston, MA; ^7^Boston Children's Hospital, Division of Fetal Medicine and Surgery, Boston, MA; HCA Healthcare and HCA Healthcare Research Institute, Nashville, TN; HCA Texas Maternal Fetal Medicine, Houston, TX; Baylor College of Medicine and Texas Children's Hospital, Houston, TX, Boston, MA; ^8^Fetal Surgeon Chief, Division of Fetal Medicine and Surgery Director of the Maternal Fetal Care Center Director of the Perinatal Surgery Fellowship Professor of Surgery, Boston Children's Hospital Harvard Medical School, Boston, MA; ^9^Indiana University and Riley Children's Hospital, Indianapolis, IN*

10:30 AM ‐ 12:30 PM

**Objective**: To investigate the prevalence trend and characteristics of congenital gastroschisis in the United States between 2014 and 2022

**Study Design**: A cross‐sectional retrospective analysis of the Centers for Disease Control and Prevention database for United States live births between 2014 and 2022. Neonatal singleton live births with documented isolated gastroschisis were included, and neonates with other major congenital anomalies and known chromosomal abnormalities were excluded. Prevalence per 10,000 live births along with 95% confidence intervals were estimated, with comparisons to unaffected birth rates in same aged cohorts.

**Results**: Among 32,088,301 singleton live births (2014–2022), 6,804 cases of isolated gastroschisis were identified (point prevalence: 2 per 10,000 live births). Starting in 2018, a significant decline in gastroschisis prevalence was observed, decreasing from 2.86 in 2014 to 1.55 per 10,000 live births in 2022 (*p*< 0.001). The risk of gastroschisis was significantly higher in teen and nulliparous gravidae, among non‐Hispanic indigenous Americans, with maternal tobacco use, and among vulnerable populations (underweight, < 12th grade education, Medicaid). The declining rate in neonates with gastroschisis, compared to neonates without gastroschisis, is attributable to declines in births among gravidae under 25 (p = 0.02), with significant declines in prevalence of other attributable maternal factors (*p*< 0.001). Despite early reports suggesting a mediating effect of COVID‐19, there was no significant difference of the pandemic on the declining trend rate.

**Conclusion**: This comprehensive analysis of over 32 million births highlights a notable decline in the prevalence of neonates with gastroschisis, which is attributable to a declining birth rate in the highest at‐risk strata of vulnerable gravidae. Given recent increases in birth rates in these same populations, our findings anticipate a reverse of the trend of declining gastroschisis disease prevalence. Further research is necessary to understand the role of access to reproductive care in rates of common congenital anomalies.

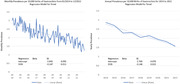

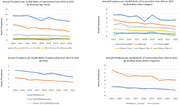

## 873 | Evaluating Surgical Approaches for Prenatal Repair of Open Spina Bifida: A Systematic Review and Meta‐Analysis

Nikan Zargarzadeh^1^; Enaja Sambatur^2^; May Abiad^3^; Ehsan Rojhani^4^; Ali Javinani^1^; Weston Northam^1^; Eyal Krispin^5^; Ramen H. Chmait^6^; Kjersti M. Aagaard^7^; Alireza A. Shamshirsaz^8^

*^1^Boston Children's Hospital, Harvard Medical School, Boston, MA; ^2^Fetal Care and Surgery Center, Division of Fetal Medicine and Surgery, Boston Children's Hospital and Harvard School of Medicine, Boston, MA; ^3^Maternal Fetal Care Center, Boston Children's Hospital, Boston, MA; ^4^Maternal Fetal Care Center (MFCC), Division of Fetal Medicine and Surgery, Boston Children's Hospital, Harvard Medical School, Boston, MA; ^5^Harvard Medical School, Boston, MA; ^6^Keck School of Medicine, University of Southern California, Los Angeles, CA; ^7^Boston Children's Hospital, Division of Fetal Medicine and Surgery, Boston, MA; HCA Healthcare and HCA Healthcare Research Institute, Nashville, TN; HCA Texas Maternal Fetal Medicine, Houston, TX; Baylor College of Medicine and Texas Children's Hospital, Houston, TX, Boston, MA; ^8^Fetal Surgeon Chief, Division of Fetal Medicine and Surgery Director of the Maternal Fetal Care Center Director of the Perinatal Surgery Fellowship Professor of Surgery, Boston Children's Hospital Harvard Medical School, Boston, MA*

10:30 AM ‐ 12:30 PM

**Objective**: Over the past decade, prenatal repair of open spina bifida has become well‐established. Since the MOMS trial, a widely varying number of surgical approaches have emerged, each focused on optimizing outcomes while minimizing risks. This study aims to compare the outcomes of these different surgical techniques.

**Study Design**: This systematic review and meta‐analysis synthesizes data from 38 studies between 2010 and 2024. Eligible studies included pregnant patients diagnosed with open spina bifida who underwent the following intrauterine repair techniques: open, mini‐hysterotomy, laparotomy‐assisted fetoscopic, and percutaneous fetoscopic repair. The primary outcome investigated was gestational age (GA) at delivery, while secondary outcomes were preterm premature rupture of membranes (PPROM), preterm delivery, vaginal birth, and perinatal mortality.

**Results**: In this study, 2,333 prenatal repair of open spina bifida procedures were analyzed across 14 countries. Of these, open repair accounted for 65.66%, mini‐hysterotomy 14.40%, laparotomy‐assisted fetoscopic 5.36%, and percutaneous fetoscopic 14.57%. Subgroup analyses revealed a mean GA at birth of 34.20 weeks for open repair, 34.30 weeks for mini‐hysterotomy, 35.38 weeks for laparotomy‐assisted, and 32.48 weeks for percutaneous fetoscopic method, with no significant difference noted between subgroups. Overall rates of preterm delivery before 37 weeks and PPROM were 0.75 and 0.30, respectively. Both showed significant differences between subgroups (P< 0.01). Vaginal birth rates had significant subgroup differences (P< 0.01), with the laparotomy‐assisted fetoscopic group more likely to have vaginal deliveries (0.02, 0.04, 0.49, 0.18 for open, mini, laparotomy, and percutaneous, respectively)

**Conclusion**: Collectively, the data from this meta‐analysis suggests that gestational age does not significantly differ by the surgical technique employed for prenatal repair of open spina bifida.

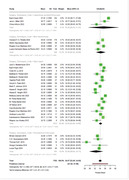

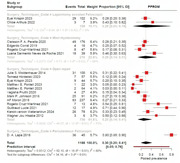

## 874 | Planned Versus Emergent Uterine Preserving Cesarean Delivery for Placenta Accreta Spectrum

Nirit Maliyanker^1^; Elias Cstel^2^; Nizan Mor^3^; Lior Fridrich^3^; Shlomi Toussia Cohen^3^; Aviran Ohayon^3^; Alina Weissmann‐Brenner^3^; Gabriel Levin^4^; Raanan Meyer^5^

*^1^Sheba Medical Center, Givatayim, Tel Aviv; ^2^Sheba Medical Center, Ramat gan, HaMerkaz; ^3^Sheba Medical Center, Ramat Gan, HaMerkaz; ^4^McGill University, Montreal, PQ; ^5^Cedars Sinai Medical Center, Los Angeles, CA*

10:30 AM ‐ 12:30 PM

**Objective**: Uterine preserving cesarean delivery is increasingly used to manage placenta accreta spectrum (PAS). Outcomes of emergent compared to elective cesarean hysterectomy were previously studied.  The aim of this study is to compare maternal and neonatal outcomes between women who had emergent and elective uterine preserving cesarean delivery for PAS cases.

**Study Design**: This is a retrospective study conducted at a single tertiary center. PAS cases scheduled for uterine preserving surgery between 3/2011 to 11/2020 were retrieved and analyzed. Delivery was defined as elective when performed at a time and date planned by a dedicated multidisciplinary team, all other cases were defined as emergent. The primary outcome is composite maternal outcome defined by one or more of these variables: unplanned hysterectomy, intensive care unit admission, transfusion of six or more red blood cell units, disseminated intra‐vascular coagulation. ureteric injury, bowel injury, unintentional cystotomy and relaparotomy. Secondary outcome included neonatal outcome such as APGAR score, cord pH, admission to the neonatal intensive care and mechanical ventilation.

**Results**: 274 women with PAS diagnosis were scheduled to uterine preserving surgery. 215 underwent elective surgery and 59 had emergent surgery. Composite maternal outcome occurred in 81 women (29.6%) of all women, no significant difference was found between the elective surgery group and the emergent surgery group (28.8% vs 32.2% p = 0.631). Significant differences were found in neonatal outcomes: longer hospital stay (8.42 ± 7.06 vs 16.28 ± 13.98 p< 0.01), higher rate of mechanical ventilation (7% vs 18.6% p< 0.01) and neonatal intensive care unit hospitalization in the emergent cesarean group (24.2% vs 55.9% p< 0.01).

**Conclusion**: Emergent uterine preserving surgery in PAS cases is not associated with increased maternal morbidity. This should be considered against prematurity complication when scheduling uterine preserving surgery. Higher rate of neonatal adverse outcomes was found in the emergency group can be attributed to an earlier gestational week at delivery.

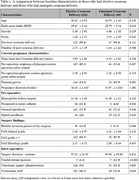

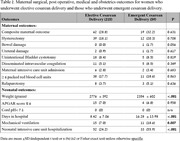

## 875 | Angiogenic Factors for Prediction of Adverse Outcomes in Pregnancies Complicated by Isolated Fetal Growth Restriction

Noa Gonen^1^; Uri Shemesh^2^; Keren Zloto^3^; Itay Manor^4^; Sharon Kronkop^5^; Tal Dadon^6^; Rakefet Yoeli‐Ullman^3^; Shali Mazaki Tovi^7^; Yoav Yinon^7^; Michal Fishel Bartal^8^; Abraham Tsur^9^

*^1^Sheba Medical Center, Ramat gan, HaMerkaz; ^2^Sheba Medical Center, Beer Sheba, HaMerkaz; ^3^Sheba Medical Center, Ramat Gan, HaMerkaz; ^4^Tel aviv university, Tel aviv university, Tel Aviv; ^5^Tel Aviv university, Tel Aviv university, HaMerkaz; ^6^Sheba Medical Center, Ramat Gan, Tel Aviv; ^7^The Sheba Medical Center, Ramat Gan, HaMerkaz; ^8^UTH Houston & Sheba Medical Center Israel, Houston, TX; ^9^Sheba Medical Center, Ramat Gan, Tel Hashomer, Israel, Ramat Gan, HaMerkaz*

10:30 AM ‐ 12:30 PM

**Objective**: To evaluate whether abnormal sFlt‐1/PlGF ratio predicts latency to delivery and development of hypertensive disorders of pregnancy (HDP) among individuals presenting with fetal growth restriction (FGR)

**Study Design**: A retrospective study including individuals at ≥ 23 weeks presenting to a tertiary care center with suspected FGR (< 10th percentile) (08/2023 to 04/2024). During this period, sFlt‐1/PlGF levels were obtained for all individuals with FGR but were not used to guide

clinical management. Patients with HDP on presentation were excluded. Median interval to delivery, rate of HDP, and other adverse outcomes were compared between those with and without abnormal sFLT‐1/PLGF ratio (≥ 38).

Odds ratio and 95% confidence interval (95%CI) were calculated.

**Results**: Of 104 patients with suspected FGR, 36 (34.6%) had an abnormal ratio of sFlt‐1/PlGF (≥38). Those with abnormal sFlt‐1/PlGF levels were more likely to be nulliparous compared to those with normal testing (Table 1). The rate of HDP was higher among those with abnormal sFlt‐ 1/PlGF ratio (30.6% Vs 2.94%, p< 0.01, OR14.5 (3‐70.2)) with a shorter median latency from test to delivery (20 vs 36 days, p< 0.01) compared to normal testing. Furthermore, those with abnormal sFlt‐ 1/PlGF ratio delivered earlier (35.4 vs. 37.1, p< 0.01) with a lower birthweight (1876 vs 2182 gram, p< 0.01) compared to those with normal testing.

**Conclusion**: Abnormal sFlt‐1/PlGF ratio in normotensive individuals with suspected FGR may predict short interval to delivery, development of HDP, and adverse perinatal outcomes.

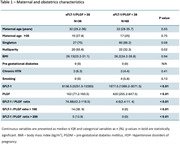

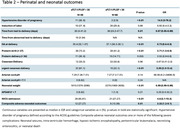

## 876 | Does an Early Isolated Decrease in Fetal Abdominal Circumference Heighten the Risk of Growth Restriction?

Patrick S. Kim^1^; Tiffany A. Lowtan^1^; David J. Rivera Vazquez^1^; Kelsey M. Pozerski^1^; Krista Howard^2^; Bradley H. Sipe^3^; Rachelle Schwartz^3^

*^1^Orlando Health Bayfront Hospital, St. Petersburg, FL; ^2^Texas State University, San Marcos, TX; ^3^Johns Hopkins All Children's Hospital, St. Petersburg, FL*

10:30 AM ‐ 12:30 PM

**Objective**: To determine if an isolated second trimester fetal abdominal circumference (AC) < 10th %tile is an independent risk factor for fetal growth restriction (FGR) and small for gestational age (SGA) neonates.

**Study Design**: This multi‐center retrospective cohort study analyzed patients who delivered term singletons between 1/1/2019‐1/1/2024. The exposed group involved normally grown fetuses with isolated AC's < 10th %tile, measured between 18 and 24 weeks gestation. Control subjects were randomly selected from the same time period. Congenital anomalies were excluded. Statistical analyses included independent t‐tests for continuous variables, chi‐squared tests for categorical variables, and binary logistic regression models both unadjusted and adjusted for FGR and SGA.

**Results**: Of the 1,213 patients screened, 589 met the inclusion criteria (AC< 10 n = 213; Control n = 376). The mean AC was 6.3%tile in the exposed group compared to 51.2%tile in the controls. Birthweight in grams (2,782 vs. 3,257, p< 0.001) and %tiles (18.3% vs. 41.8%, p< 0.001) were lower in the AC< 10 group. AC was found to be an independent risk factor for both FGR (AOR 12.5 [6.7, 23.4]) and SGA (AOR 5.08 [3.32, 7.76]). Logistic regressions analysis for primary and secondary outcomes are shown in Table 2. Although there were more pregestational diabetic mothers in the AC< 10 group (10.3% vs. 3.7%, p = 0.001), pregestational diabetes was ultimately protective of FGR (AOR 0.15 [0.04, 0.54]). As maternal BMI increased, the likelihood of FGR (AOR 0.95 [0.90, 0.995]) and SGA (AOR 0.94 [0.91, 0.97]) decreased. Demographically, Black patients were more prevalent in the AC< 10 group compared to controls (36.2% vs. 25.8%, p = 0.027). There were no differences in parity, gestational diabetes, chronic or gestational hypertension, pre‐eclampsia, BMI, or mode of delivery between groups.

**Conclusion**: An isolated second trimester AC< 10th %tile in an otherwise normally grown fetus is an independent risk factor for both FGR and SGA. Increased maternal BMI was protective of both FGR and SGA. A history of pregestational diabetes was protective of FGR but not SGA.

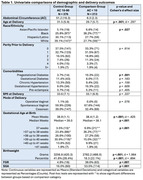

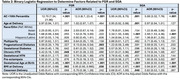

## 877 | Maternal and Fetal Outcomes of Pregnancies in Women with Cystic Fibrosis

Paul Wasuwanich^1^; Tony Wen^2^

*^1^Naples Comprehensive Health, Naples, FL; ^2^University of Florida College of Medicine, Gainesville, FL*

10:30 AM ‐ 12:30 PM

**Objective**: Cystic fibrosis (CF) is a genetic disease that had previously made pregnancy near impossible. With advancements in therapy, pregnancies in women with CF are increasing. We aim to describe the maternal and fetal outcomes in a large cohort of pregnancies with CF in the U.S.

**Study Design**: We utilized the National Inpatient Sample and extracted cases of CF‐associated pregnancies and deliveries. We described the characteristics and perinatal outcomes of pregnant women with CF and compared them to general non‐CF pregnancies in the U.S. from 1998‐2021. Trends were analyzed with Poisson regression and rates were analyzed with chi‐squared test.

**Results**: There was a total of 11,496 pregnancies with CF. From 1998 to 2021, hospitalization rates of CF pregnancies and deliveries have increased by 5‐fold and 7‐fold, respectively (Figure 1). Median age was 26 years in the CF pregnant cohort compared to 28 years in the non‐CF cohort (p< 0.0001). Median length of hospital stay was longer in the CF cohort (3 days vs 2 days; p< 0.0001). Maternal deaths occurred at significantly higher rates in the CF cohort (0.496% vs 0.013%; p< 0.0001). Rates of diabetes (6.79% vs 1.11%; p< 0.0001), obesity (4.59% vs 3.81%; p = 0.049), hepatitis C infection (0.51% vs 0.28%; p = 0.033), cirrhosis (0.435% vs 0.006%; p< 0.0001), intravenous drug use (1.16% vs 0.61%; p< 0.001), and coagulopathy (1.24% vs 0.32%; p< 0.0001) were significantly higher in the CF pregnancy group compared to the non‐CF pregnancy group. Poor fetal growth was more common in the CF cohort (3.05% vs 2.28%; p = 0.013). Rates of pre‐eclampsia, eclampsia, and intrauterine fetal demise were similar between the two groups (p >0.05). Among the 11,496 CF pregnancies, 6,786 (59.0%) had record of delivery. Rates of stillbirths (1.31% vs 0.76%; p = 0.020) and preterm births (17.30% vs 7.83%; p< 0.0001) were higher in the CF cohort. Maternal death during delivery was also higher in the CF cohort (0.147% vs 0.006%; p< 0.0001).

**Conclusion**: CF pregnancies and deliveries are increasing over time, but morbidity and mortality in this group is higher than in the general pregnant population.

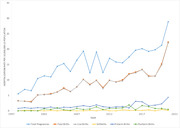

## 878 | Association Between Hypertensive Disorders and Neonatal Mortality Among Periviable Deliveries

Peeraya S. Sawangkum^1^; Jean Paul P. Tanner^1^; Jason L. Salemi^1^; Jose R. Duncan^2^

*^1^Department of Obstetrics and Gynecology, University of South Florida, Tampa, FL; ^2^Department of Obstetrics and Gynecology, University of South Florida, Morsani College of Medicine, Tampa, FL*

10:30 AM ‐ 12:30 PM

**Objective**: The objective of this study was to determine the association between hypertensive disorders of pregnancy (including chronic hypertension, gestational hypertension, and pre‐eclampsia) and 1‐year survival among periviable neonates delivered at 22w0d‐25w6d.

**Study Design**: This is a retrospective cohort study using a maternal‐infant linked database of live births in the state of Florida from 2006 to 2019. Singleton deliveries of infants born at 22w0d to 25w6d in which neonatal resuscitation was attempted were included. Adjusted hazard ratios (aHRs) were estimated to compare survival across exposures groups, overall and by week of gestation.

**Results**: Of the 6,898 periviable deliveries identified during the study period, 1,405 (20.4%) were affected by a hypertensive disorder. Patients affected with hypertension were also more likely to be of advanced maternal age, overweight/obese, non‐Hispanic Black, have pre‐existing diabetes, have connective tissue / autoimmune conditions, and have a diagnosis of fetal growth restriction. Among all periviable births, hypertensive disorders were associated with increased risk of death during the first year of life (aHR 1.24, 95% CI 1.13‐1.38). However, when stratified by gestational age week, hypertensive disorder had no effect on risk of death at 1‐year among 22‐ and 23‐week neonates (aHR 0.73, 95% CI 0.50‐1.07 and aHR 1.17, 95% CI 0.96‐1.43 respectively), but increased risk of death among 24‐ and 25‐ week neonates (aHR 1.37, 95% CI 1.16‐1.62 and aHR 1.41, 95% CI 1.17‐1.69 respectively).

**Conclusion**: This study demonstrates that hypertensive disorders of pregnancy are associated with increased neonatal mortality among periviable neonates born at 24‐ and 25‐weeks gestation, but not at 22‐ and 23‐weeks gestation. These findings may help guide perinatologists when counseling patients in the periviable period.

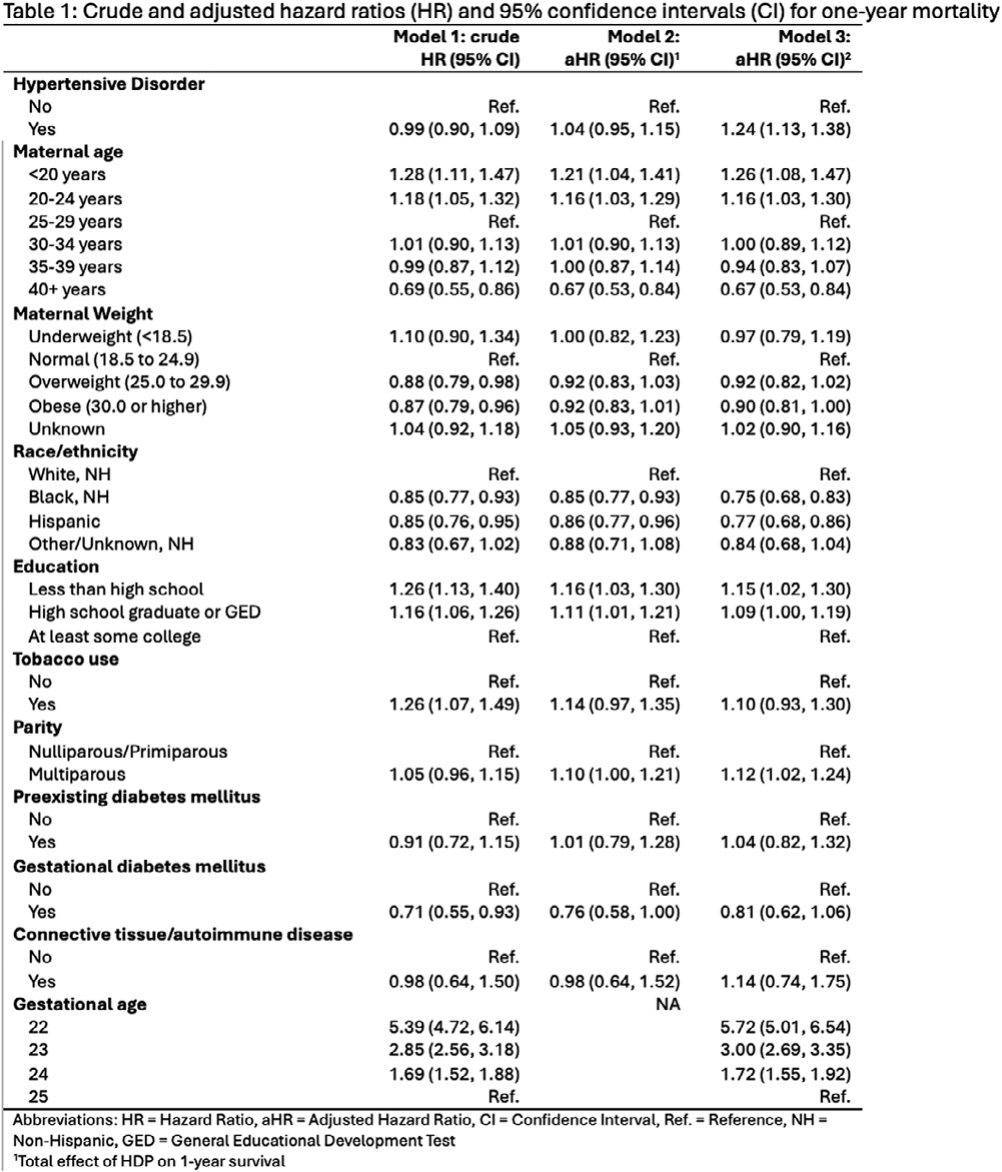

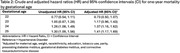

## 879 | Assessment of Cardiac Outcomes in Pregnant Women with Congenital Heart Disease: A Single Center Experience

Poojita Dasika^1^; Shreya Ramineni^2^; Anna E. Bortnick^3^; Manoj Gupta^3^; Daphne Hsu^3^; Diana S. Wolfe^3^

*^1^Albert Einstein College of Medicine, The Bronx, NY; ^2^Albert Einstein College of Medicine, Bronx, NY; ^3^Montefiore Medical Center/Albert Einstein College of Medicine, Bronx, NY*

10:30 AM ‐ 12:30 PM

**Objective**: Women with congenital heart disease (CHD) have a significant risk of cardiovascular complications during pregnancy. This study examined the association between current cardiac risk stratification tools and cardiovascular outcomes in adult pregnant women with CHD.

**Study Design**: A retrospective observational study was performed via chart review of all patients ≥ 18 years of age with underlying mild, moderate, or complex CHD who received care at the Albert Einstein College of Medicine at the Montefiore Medical Center, Bronx, New York from January 2015 until April 2024. Pregnancy risk scores were calculated using the mWHO, ZAHARA, CARPREG I, and CARPREG II risk classification models. The primary outcome was to compare the observed versus predicted occurrence of maternal cardiovascular complications by each model. Secondary outcomes included identifying individual risk factors for maternal cardiovascular complications. A univariate analysis was performed to identify significant predictors of cardiovascular complications.

**Results**: Of 170 pregnancies, 16 (9.4%) had cardiovascular complications antepartum or up to 6 months postpartum. The ratio of weighted average observed risk within the cohort to weighted average predicted risk was calculated for each model: 0.63 for mWHO, 0.73 for ZAHARA, 0.73 for CARPREG I, and 0.84 for CARPREG II (Figure 1). Significant (p< 0.05) univariate risk factors for maternal cardiovascular complications included anticoagulation during pregnancy, prior cardiac event or arrhythmia, cardiac medication during pregnancy, moderate to severe pulmonary hypertension, systemic ventricular dysfunction, cardiac medication before pregnancy, NYHA functional class >II, and late pregnancy assessment (Table 1).

**Conclusion**: All four risk models overpredicted maternal cardiac risk. CARPREG II was the most accurate representation of cardiac risk in our cohort of pregnant women in the Bronx with CHD. Though not included in any of the risk models, anticoagulation during pregnancy was the most significant individual predictor of maternal cardiovascular complications.

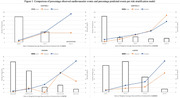

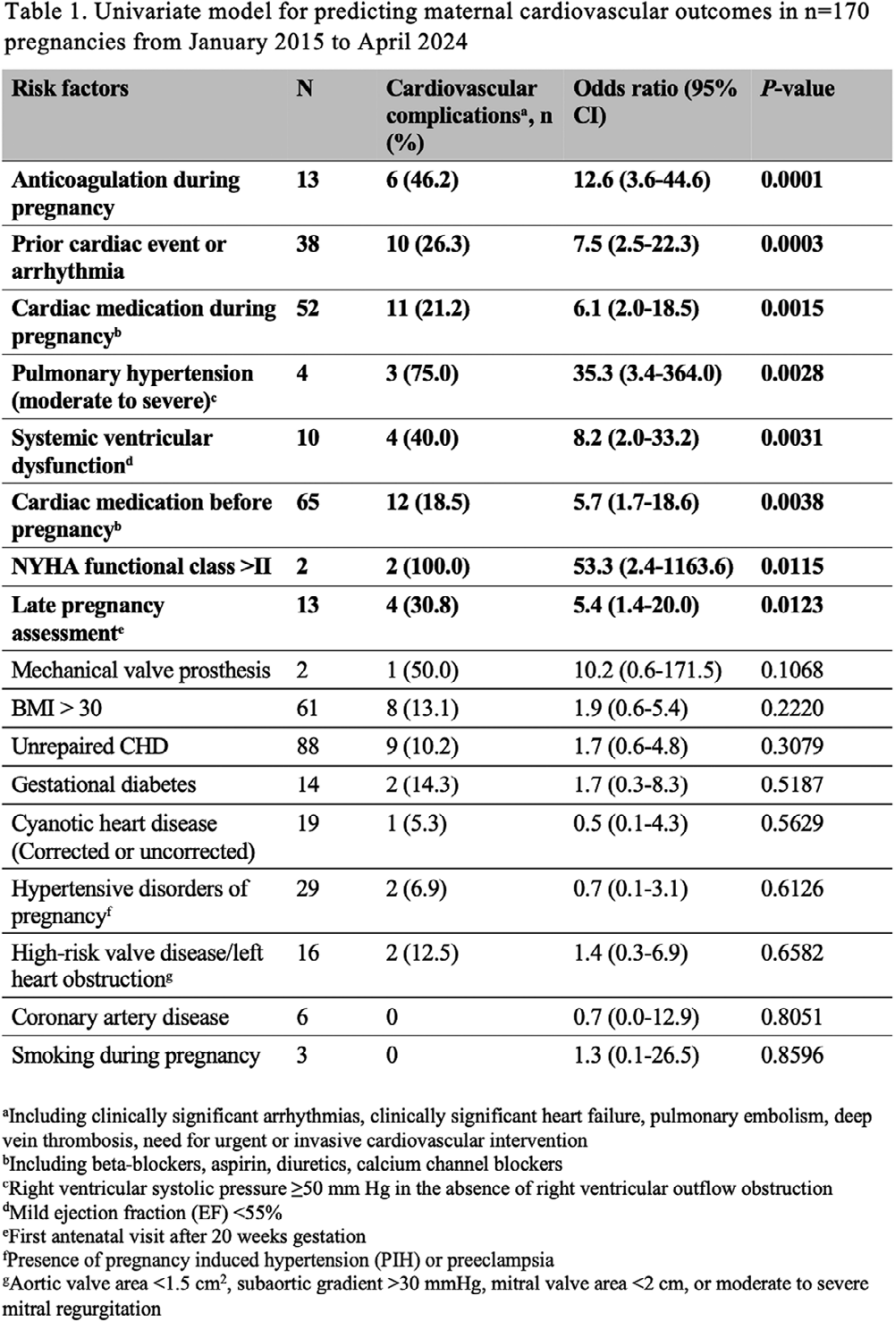

## 880 | Treating Women with Isolated Intrapartum Fever as Chorioamnionitis: A Cost Effectiveness Analysis

Prakrunya Subhasree Badrinarayan; Megha Arora; Aaron B. Caughey

*Oregon Health & Science University, Portland, OR*

10:30 AM ‐ 12:30 PM

**Objective**: Intrapartum fever (≥ 38°C) affects many deliveries annually in the U.S., posing risks to mothers and neonates. Standard treatments include expectant management for isolated intrapartum fever and ampicillin‐gentamicin for chorioamnionitis. Antibiotic treatment helps reduce infection risk due to expectant management but may increase costs. This study evaluated the cost‐effectiveness of treating isolated intrapartum fever as chorioamnionitis.

**Study Design**: We constructed a decision‐analytic model in TreeAge Pro to compare outcomes between isolated intrapartum fever (expectant management) versus chorioamnionitis (ampicillin/gentamicin). Our theoretical cohort was 222,000 patients, representing vaginal singleton pregnancies with intrapartum temperature ≥ 38°C in the U.S. annually. Outcomes were incremental cost per quality‐adjusted life years (QALYs), postpartum endometritis, neonatal early‐onset sepsis (nEOS), neonatal death, neonatal neurodevelopmental delay (NDD), and acute kidney injury (AKI) from gentamicin toxicity. The willingness‐to‐pay threshold was $100,000/QALY. We discounted QALYs at a rate of 3%. Model inputs were derived from literature and assessed using univariate sensitivity analyses.

**Results**: In our theoretical cohort of 222,000 individuals, antibiotic treatment reduced 7,481 cases of postpartum endometritis (8,649 vs 16,131), 6,047 cases of nEOS (961 vs 7,009), 677 neonatal deaths (1,350 vs 2,027), and 331 cases of NDD (15,485 vs 15,815) compared to expectant management. However, gentamicin use for chorioamnionitis resulted in 21,305 more cases of AKI (31,069 vs 9,764). Despite higher costs, treating intrapartum fever as chorioamnionitis was cost‐effective with a cost‐effectiveness ratio (ICER) of $5,708/QALY. Sensitivity analyses showed treatment remained cost‐effective until AKI probability exceeded 70%.

**Conclusion**: Treating intrapartum fever with antibiotics is a cost‐effective strategy to reduce the adverse outcomes of untreated intrapartum fever. Adopting a protocol to treat all intrapartum fevers ≥ 38°C as chorioamnionitis with appropriate antibiotics could benefit health systems.

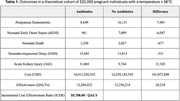

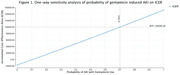

## 881 | Circumstances and Trends of Pregnancy‐Associated Suicides in the United States from 2018 to 2021

Qing Li^1^; Emily S. Miller^2^; Sarah G. Obican^3^; Daniel Romer^4^

*^1^Independent Researcher, Denver, CO; ^2^Women & Infants Hospital of Rhode Island and Alpert Medical School of Brown University, Providence, RI; ^3^Department of Obstetrics and Gynecology, University of South Florida, Tampa, FL; ^4^Annenberg Public Policy Center, University of Pennsylvania, Philadelphia, PA*

10:30 AM ‐ 12:30 PM

**Objective**: Pregnancy‐associated suicide is a common cause of maternal mortality in the US. Our objective was to investigate the trends and occurrence of circumstances surrounding pregnancy‐associated suicide before and during the COVID‐19 pandemic, with a specific focus on understanding its relationship with intimate partner adversity (IPA).

**Study Design**: In a cross‐sectional study, we analyzed 315 pregnancy‐associated suicide incidents from 34 states, the District of Columbia, and Puerto Rico with complete case reporting (Figure) in the 2018‐2021 National Violent Death Reporting System, which is a national surveillance system for violent death and the largest dataset on suicide decedents. Information was captured by standardized coding in death certificates, records from coroner and medical examiners and law enforcement, and toxicology reports. IPA was defined as a divorce, breakup, argument, jealousy, conflict, or violence among intimate partners, which appeared to have contributed to suicide. The characteristics of the victim, incident, and circumstances were compared, stratified by the presence of documented IPA, using bivariate analyses.

**Results**: Pregnancy‐associated suicide counts decreased 12.5% from 168 (2018‐2019) to 147 (2020‐2021). Half died between 43 to 365 days postpartum (Table). Firearms were the primary lethal means (41%) followed by hanging, strangulation, or suffocation (40%). Among 315 cases, 63% were White, 60% had a previously identified mental health condition (MHC), and 46% had IPA. Compared to cases without IPA, IPA‐involved cases did not vary by the presence of MHC or injury location (p >0.05) but were more likely to involve a suicide following the homicide (p = 0.04) and alcohol use (p = 0.02).

**Conclusion**: Pregnancy‐associated suicide decreased from two years before to two years during the pandemic in the US. MHC, IPA, and firearm use were common. Policies and programs to screen and respond to MHC, firearm use, IPA and violence among women of reproductive age may be beneficial public health strategies to prevent pregnancy‐associated suicide.

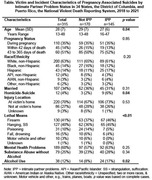

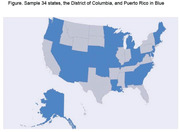

## 882 | Features of Additional Uterotonic Use Without Postpartum Hemorrhage

Rachel L. Wiley^1^; Ipsita Ghose^2^; Hector M. Mendez‐Figueroa^3^; Suneet P. Chauhan^4^

*^1^University of California, San Diego, San Diego, CA; ^2^Baylor College of Medicine, Houston, TX; ^3^McGovern Medical School at UTHealth, Houston, TX; ^4^Delaware Center of Maternal‐Fetal Medicine at Christiana Care, Delaware, DE*

10:30 AM ‐ 12:30 PM

**Objective**: Use of uterotonics beyond prophylactic ranges from 2‐25%, while postpartum hemorrhage (PPH; blood loss > 1000 mL) ranges from 3‐5%. We examined factors in individuals without PPH who received additional uterotonics versus those who did not.

**Study Design**: This retrospective cohort included all singleton live births > 22 weeks with 24‐hour blood loss < 1000 mL at a Level IV center over 24 months. Clinical and demographic elements were abstracted by medical staff, and additional uterotonic use was defined as any agent utilized after prophylactic oxytocin, including additional doses of oxytocin, misoprostol, methylergonovine, tranexamic acid or carboprost. Individuals were stratified by route of delivery and examined by students' t‐test and chi‐square.

**Results**: Of 8,623 deliveries, 7,616 (88%) were included with 4,369 (57.4%) vaginal deliveries (VD) and 3,247 (42.6%) cesarean deliveries (CD). VD without PPH was significantly more likely to receive additional uterotonics compared to CD (14% vs. 11%, p< 0.001). The average blood loss was higher with vs. without uterotonics (398 ± 211.24 mL vs 241 ± 149.92 mL, p< 0.001 for VD; 700 ± 135.43 mL vs 633 ± 129.62 mL, p< 0.001 for CD). While some pre‐existing characteristics differed, they were inconsistent across routes except for BMI, hypertensive disorders, low platelets, magnesium sulfate and oxytocin use (Table 1 & 2). There were no significant differences in provider characteristics. PPH risk stratification was only different in VD. Deliveries without PPH who received additional uterotonics were more likely to be transfused, receive additional surgical intervention and be admitted to intensive care (Table 2).

**Conclusion**: About 1 in 10 deliveries without PPH received uterotonics beyond prophylactic. No risk factors or patient characteristic predicted this behavior. Blood loss and interventions were increased with additional uterotonics, suggesting clinicians may be pre‐emptively treating clinical concern rather than risk factors.  Additional exploration into risks, benefits, and threshold of early intervention with uterotonics is warranted.

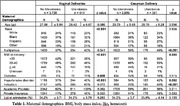

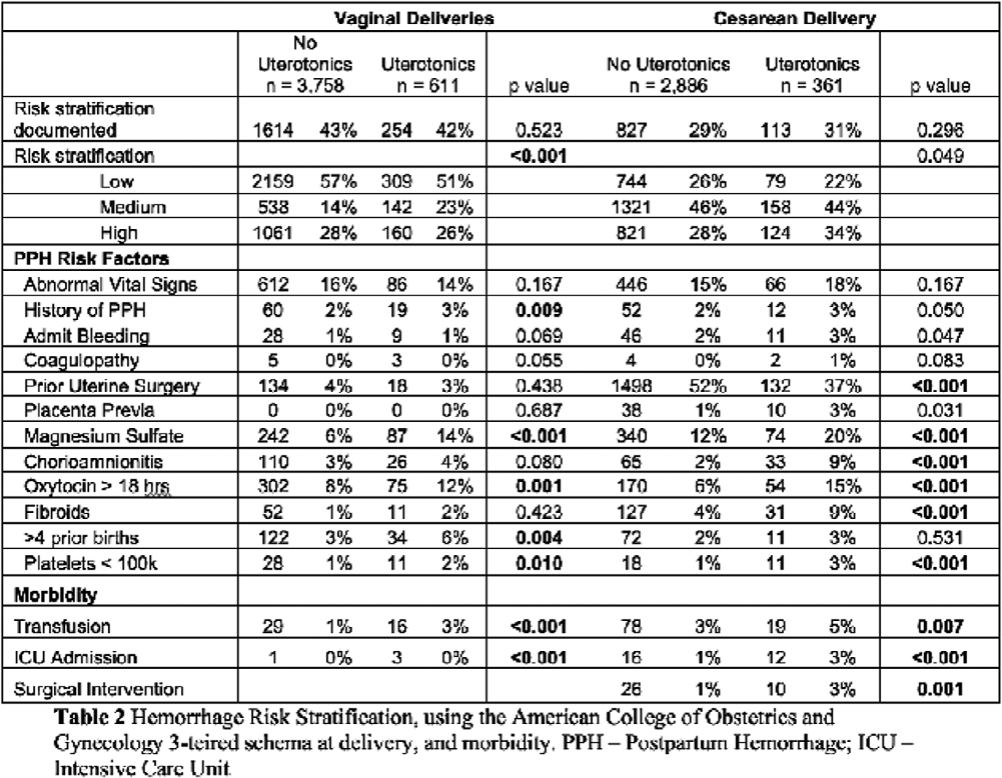

## 883 | Hypertension in Pregnancy Without a Documented Diagnosis: A Missed Window of Opportunity

Rachel D. Seaman; Caitlin Partridge; Lisbet S. Lundsberg; Jennifer F. Culhane; Anna Denoble

*Yale School of Medicine, New Haven, CT*

10:30 AM ‐ 12:30 PM

**Objective**: Obstetric patients with chronic hypertension (HTN) and hypertensive disorders of pregnancy (HDP) may not receive corresponding ICD‐10 codes due to missed diagnoses or documentation errors. We aimed to compare (1) healthcare engagement at 6 weeks postpartum (PP) and (2) American College of Cardiology (ACC) HTN staging at 6 months PP between hypertensive patients with and without corresponding ICD‐10 codes.

**Study Design**: This was a retrospective study of patients delivering within a healthcare system from 2013‐2023 with ≥1 prenatal visit. Exclusion criteria were < 2 recorded blood pressures (BPs) at < 20 weeks’ gestation and no evidence of elevated BPs or cHTN/HDP by ICD‐10 code. The electronic medical record was used to identify patients with HTN; these were then classified as “BP only” (≥2 BPs of ≥140/90 mmHg at < 20 weeks and/or 20 weeks to delivery discharge *without* an ICD‐10 code for cHTN/HDP) or “ICD‐10” (*with* an ICD‐10 code for cHTN/HDP). Demographic characteristics and ACC HTN stages at 6 months were compared between groups using chi‐square tests. The primary outcomes ‐ attendance at any PP visit or HTN‐focused visit within 6 weeks PP ‐ were compared using multivariate logistic regression, adjusted for significant covariates.

**Results**: Of 21,296 patients, 9,833 (46.2%) were identified as BP only and 11,463 (53.8%) as ICD‐10. Compared to the BP only group, the ICD‐10 group was significantly more likely to attend any PP visit [80.1% vs. 65.6%; aOR 2.0 (95% CI 1.8‐2.1)] or PP HTN‐focused visit [36.4% vs. 2.5%; aOR 17.3 (95% CI 15.1‐19.8)]. Both BP only and ICD‐10 patients demonstrated elevated BP by ACC staging at 6 months PP: elevated (13.1% vs 13.3%), Stage 1 (14.7% vs 22.6%), and Stage 2 (2.3% vs 9.4%).

**Conclusion**: Elevated BP in pregnancy in the absence of an associated ICD‐10 diagnosis code is associated with lower healthcare engagement up to 6 weeks PP. Despite this, patients with elevated BPs without an ICD‐10 diagnosis of cHTN or HDP remain at risk for ongoing BP elevation for at least 6 months from delivery, highlighting a need for improved recognition and documentation of antenatal HTN.

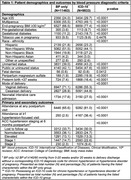

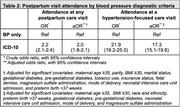

## 884 | Association of IVF with Preeclampsia with Severe Features Among Very Advanced Maternal Age Patients

Rachel Solmonovich; Frank I. Jackson; Jamie Green; Brenda Lin; Randi Goldman; Sarah H. Abelman; Matthew J. Blitz

*Northwell, New Hyde Park, NY*

10:30 AM ‐ 12:30 PM

**Objective**: This study aims to evaluate the association between in‐vitro fertilization (IVF) and preeclampsia with severe features (sPEC) among very advanced maternal age (vAMA) patients.

**Study Design**: Retrospective cohort study of vAMA patients who delivered at a large academic health system in New York between January 2019 and December 2022. vAMA was defined as ≥ 45 years old at delivery. Rates of sPEC and other hypertensive disorders of pregnancy were determined for IVF versus non‐IVF patients. A multivariate logistic regression was used to estimate the relationship between IVF and sPEC while controlling for obesity, nulliparity, race and ethnicity, public insurance, and primary language using R version 4.3.1. A p‐value of < 0.05 was considered statistically significant.

**Results**: A total of 591 vAMA pregnancies were included, of which 218 (37%) were IVF pregnancies and 373 (63%) were non‐IVF pregnancies.  Rates of hypertensive disorders were similar between groups (24.7% in the non‐IVF cohort and 29.4% in the IVF cohort, p = 0.212). The sPEC rate among IVF vAMA patients IVF was 16.1%, compared to 9.7% in the non‐IVF group (p = 0.021). IVF was associated with an increased likelihood of sPEC among vAMA patients in an unadjusted model, OR 1.79 (95% CI, 1.09 ‐ 2.95); however this was not the case after adjusting for potential confounders, aOR 1.57 (95% CI 0.92 ‐ 2.67).

**Conclusion**: Although vAMA and IVF have each been independently associated with an increased risk of sPEC, this is the first study to evaluate whether IVF is specifically linked to an increased risk of sPEC and hypertensive disorders of pregnancy in vAMA patients. In this cohort of patients with vAMA, IVF did not independently increase the risk of sPEC or hypertensive disorders of pregnancy.

## 885 | Increases in Routinely Measured Red Cell Indices During Pregnancy are Associated with Obstetric Complications

Rachel Petherbridge^1^; Veronica Tozzo^2^; Kaitlyn E. James^1^; Sarah Hsu^1^; Chloe Michalopoulos^1^; Brody Foy^3^; Tanayott Thaweethai^1^; Christopher Mow^1^; Jacqueline Maya^1^; Carolina Batlle Camero^4^; Lydia L. Shook^1^; Kathryn J. Gray^3^; Logan Mauney^1^; John R. Higgins^1^; Camille E. Powe^1^

*^1^Massachusetts General Hospital, Boston, MA; ^2^Massachusetts General Hospital, Massachusetts General Hospital, MA; ^3^University of Washington, Seattle, WA; ^4^University of Puerto Rico, School of Medicine, San Juan, Puerto Rico*

10:30 AM ‐ 12:30 PM

**Objective**: Pregnancy alters hematological indices, but intra‐pregnancy changes in complete blood count (CBC) values have not been rigorously evaluated as markers of pregnancy health.

**Study Design**: We retrospectively analyzed CBCs routinely measured at 7‐14 and 26‐29 weeks’ gestation to determine the association of rare changes in nine CBC indices (hematocrit, hemoglobin, red cell count, white cell count, platelets, mean red cell volume, mean red cell hemoglobin, red cell volume distribution width, and mean red cell hemoglobin concentration) with a composite outcome (hypertensive disorders of pregnancy, small for gestational age birthweight, preterm birth [both indicated and spontaneous]) and its individual components. Rare changes, defined as statistically uncommon changes in magnitude and direction, were identified in a discovery cohort of 29,162 pregnancies. Direction was chosen as the least frequent variation exceeding established non‐pregnant biological variation, and magnitude as the threshold of change with the highest positive predictive value and significant OR (p< 9×10^−6^ with Bonferroni correction). Identified associations were tested in an out‐of‐sample validation cohort of 50,603 pregnancies.

**Results**: In validation, increases in red cell indices between 7‐14 and 26‐29 weeks’ gestation were associated with the composite outcome with an OR [95% CI] of 1.49 [1.30, 1.70] for hematocrit, 1.57 [1.33, 1.86] for hemoglobin and 1.69 [1.48, 1.93] for red cell count. The strongest association with individual outcomes was observed for increases in hemoglobin (>0.67 g/dL, OR 2.03 [1.58, 2.59]) or red cell count (>0.07 106/mm3, OR 2.13 [1.74, 2.6]) and preterm birth (Table 1). These rare increases in hemoglobin and red cell count were observed on average 7 weeks before preterm birth; we did not observe a bias for detection of either spontaneous or indicated preterm birth.

**Conclusion**: Deviations of routinely measured red blood cell indices from typical longitudinal trajectories during pregnancy may allow for early detection of obstetric complications.

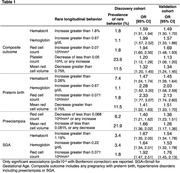

## 886 | The Predictive Value of Angiogenic Factors in Suspected Preeclampsia

Rachel C. Schell; C. Edward Wells; David M. Owen; Donald D. McIntire; David B. Nelson; F. Gary Cunningham

*University of Texas Southwestern Medical Center, Dallas, TX*

10:30 AM‐12:30 PM

**Objective**: To evaluate the ability of circulating maternal levels of angiogenic factors–soluble FMS‐like tyrosine kinase 1 (sFlt‐1) and placental growth factor (PlGF)–to predict the development of preeclampsia with severe features (SPE).

**Study Design**: This was a single‐center prospective observational study of pregnant patients admitted for evaluation of hypertensive disorders of pregnancy (HDP) between 24 0/7 weeks and 36 6/7 weeks. At enrollment, a single plasma sample was obtained for the measurement of sFlt‐1 and PlGF. All patients underwent standardized evaluation for HDP and were diagnosed with SPE based on standard laboratory and clinical criteria. Patients were examined in two cohorts: those who developed SPE within 1 week (Cohort 1) and those who did not (SPE > 1 week or never, Cohort 2). The predictive value of sFlt‐1, PlGF and sFlt‐1:PlGF ratio for the development of SPE was analyzed using logistic regression. Demographic, maternal, and neonatal outcomes were compared between cohorts.

**Results**: From Nov 2022 to May 2023, 152 patients were enrolled, including 60 (39%) diagnosed with SPE, 14 (9%) within 1 week of enrollment (Cohort 1). There were no demographic differences between groups. Patients in Cohort 1 had higher levels of sFlt‐1 (43.4 vs 20.3 ng/mL, p‐value < 0.001), lower levels of PlGF (150.9 vs 319.0 pg/mL, p‐value < 0.001), and higher sFlt‐1:PlGF (460 vs 79, p‐value < 0.001) versus patients in Cohort 2 (Figure 1). Levels of sFlt‐1 and sFlt‐1:PlGF were predictive of the development of SPE within 1 week (AUC 0.85 (95%CI 0.74, 0.95) and 0.82 (95%CI 0.71, 0.93), respectively) (Figure 2). At a cutoff of 28 ng/mL, sFlt‐1 was associated with a negative predictive value of 0.97, effectively ruling out SPE in these patients.

**Conclusion**: In pregnant patients with elevated blood pressure between 24 and 36 weeks gestation, elevated sFlt‐1 and low PlGF levels are associated with the development of SPE. Levels of sFlt‐1 below 28 ng/mL may serve as a triage tool to rule out SPE within 1 week.

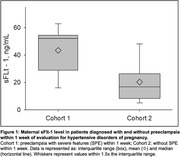

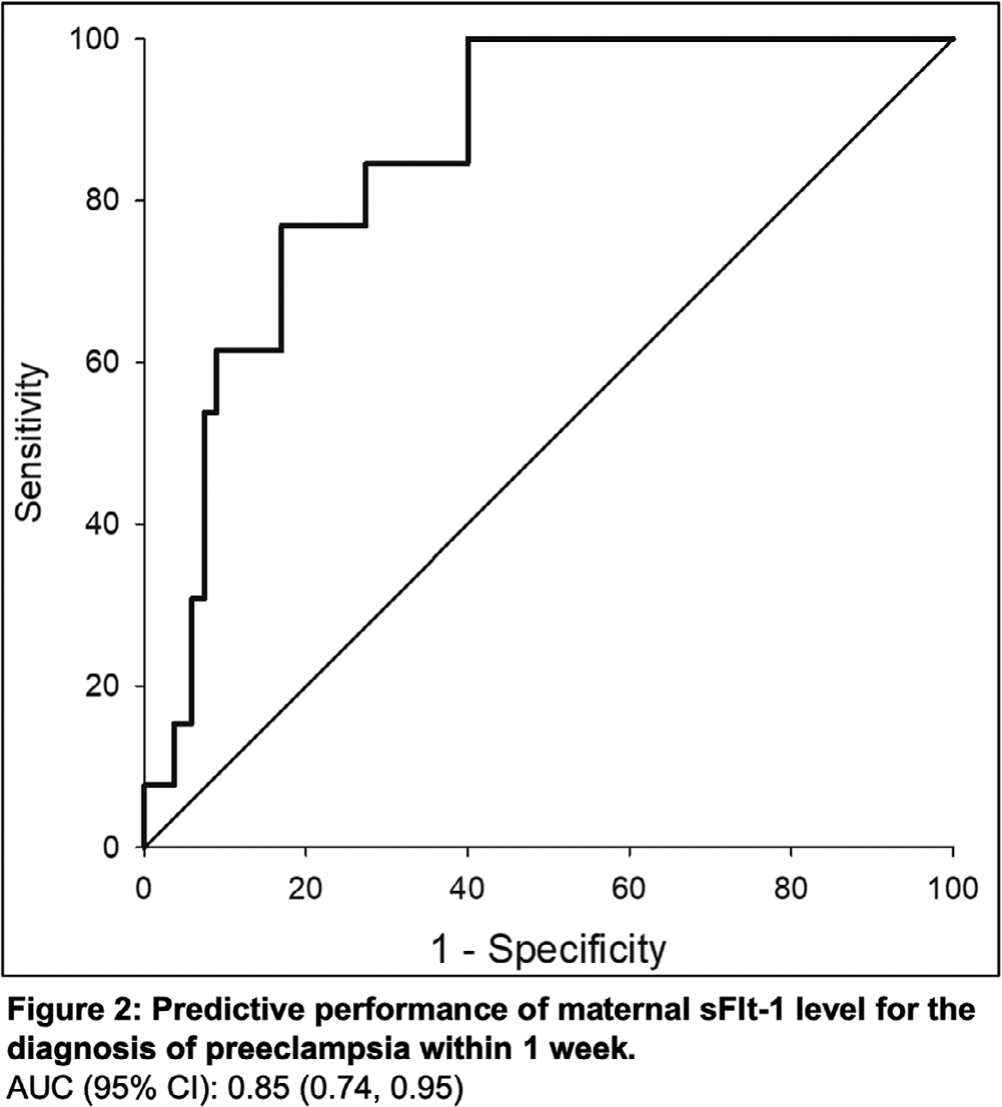

## 887 | Association of Features of Fetal Heart Rate Tracings and Adverse Neonatal Outcomes at Term

Rachel L. Wiley^1^; Kristen A. Cagino^2^; Aaron W. Roberts^3^; Claudia J. Ibarra^4^; Shareen Patel^4^; Christina Cortes^4^; Khalil M. Chahine^4^; Natalie L. Neff^5^; Kimen S. Balhotra^4^; Tala Ghorayeb^4^; Holly Flores^6^; Hector M. Mendez‐Figueroa^4^; Suneet P. Chauhan^7^

*^1^University of California, San Diego, San Diego, CA; ^2^UT Houston, Houston, TX; ^3^McGovern Medical School at UTHealth Houston, Houston, TX; ^4^McGovern Medical School at UTHealth, Houston, TX; ^5^McGovern Medical School at UT Health, Houston, TX; ^6^University of Texas Health Science Center, Houston, TX; ^7^Delaware Center of Maternal‐Fetal Medicine at Christiana Care, Delaware, DE*

10:30 AM ‐ 12:30 PM

**Objective**: To ascertain if independent features of term fetal heart rate tracings (FHRT) are associated with adverse neonatal outcomes.

**Study Design**: FHRTs of consecutive deliveries within 15 months at a Level IV center were reviewed by physicians blinded to outcomes. In 20‐min segments, the last 60 minutes available before delivery of all term (> 37 weeks), non‐anomalous singletons who labored were included. Each feature (variability, decelerations, accelerations) was independently categorized as present < vs. >  50% of the time 60 minutes before delivery. The primary outcome was the rate of composite adverse neonatal outcome (CANO; defined in Table 1). Odds ratio (OR), likelihood ratio (LR) with post‐test probability (PTB).

**Results**: Of 5,160 deliveries, 3,166 (61%) met the inclusion criteria for analysis, of which 49 (1.5%) had CANO. Baseline characteristics differed by age, hypertensive disorders, and neuraxial anesthesia use (Table 1). Individually, absent variability (0.2% vs. 2.0%, unadjusted OR 10.8, 95% CI 1.28‐91.5), marked variability (0.4% vs. 6.1%, unadjusted OR 14.5, 95% CI 4.02–52.0) and severe variable decelerations (4.5% vs 16.3%, unadjusted OR 4.18, 95% CI 1.92‐9.09) were associated with CANO if present the majority of the time. Moderate variability or the presence of accelerations were not associated with reduced CANO. Moderate variable decelerations were associated with reduced CANO (15.4% vs. 4.1%, OR 0.24, 95% CI 0.06–0.97). LR varied from 0.2 (moderate variables) to 14 (marked variability), with post‐test probability being 0 and 18%, respectively (Table 2).

**Conclusion**: Among term deliveries, during the last 60 min of labor, some FHT elements are individually associated with adverse outcomes. CANO is possible for every 1 in 7 for minimal variability, 1 in 5 for marked variability and 1 in 18 for severe variable decelerations. The presence of majority of time with moderate variability or accelerations were not associated with reduced rates of CANO. Further exploration of the elements of FHRTs that predict adverse outcomes and interventions that mitigate them are warranted.

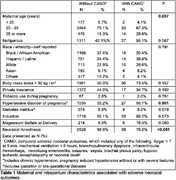

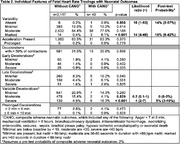

## 888 | Changes in Risk‐Appropriate Care After Implementation of Maternal Level of Care Designation in Texas

Rajesh Reddy^1^; Justin A. Drake^2^; Corwin Zigler^2^; Jeny Ghartey^2^; Alison G. Cahill^3^; Lorie M. Harper^1^

*^1^Dell Medical School, Austin, TX; ^2^University of Texas at Austin, Austin, TX; ^3^Dell Medical School Health Transformation Research Institute, Dell Medical School, The University of Texas at Austin, Austin, TX*

10:30 AM ‐ 12:30 PM

**Objective**: Perinatal regionalization rests on the assumption that designating hospital levels of care can direct patterns of appropriate site of delivery to improve patient outcomes. We evaluate if a state‐mandated maternal level of care program increased risk‐appropriate site of care in Texas.

**Study Design**: Retrospective cross‐sectional analysis of all delivery hospitalizations in Texas from 2016‐2023 using quarterly discharge data in the Texas Inpatient Public Use Data File. Of 221 designated hospitals, we included the 215 that reported deliveries in the study period (32 Level IV, 43 Level III, 86 Level II, 54 Level I). As 99% of facilities received their requested designation level, the pre‐mandate level was assumed to be the same. Deliveries at sites that were not designated maternal facilities (89 total) were categorized as Undesignated. We organized comorbidities by minimum recommended level of care (highest Level IV, lowest Level I) using criteria in the Texas Administrative Code, ACOG/SMFM Consensus, and literature. We chose a baseline of 2016(Q1) to 2018(Q4) due to (Q4)2015 introduction of ICD‐10 and 2018 Texas rule adoption, then a post‐intervention of 2021(Q4) to 2023(Q3) based on the September 2021 implementation deadline. We categorized patients into minimum level of care by their highest risk comorbidity and used two‐proportion z‐tests to compare rates of deliveries at inappropriately low facilities per each risk category. Undesignated site deliveries were considered low level.

**Results**: The proportion of births at inappropriate level facilities increased for all risk categories (Figure 1). Level III risk and severe maternal cardiac disease patients were more likely to deliver at inappropriately low facilities (Table 1).  The exception was a 4.12% decrease for placenta previa with prior uterine surgery. The proportion of undesignated hospital births decreased for all maternal risk groups.

**Conclusion**: Despite statutory adoption of levels of care, the proportion of deliveries at inappropriately low level facilities increased for all risk groups. Access barriers and transfer behavior may limit efforts.

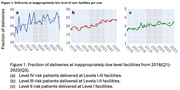

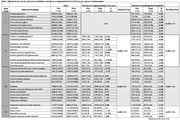

## 889 | Changes in Severe Maternal Morbidity Rates in Texas After State‐Mandated Perinatal Regionalization

Rajesh Reddy^1^; Justin A. Drake^2^; Corwin Zigler^2^; Jeny Ghartey^2^; Alison G. Cahill^3^; Lorie M. Harper^1^

*^1^Dell Medical School, Austin, TX; ^2^University of Texas at Austin, Austin, TX; ^3^Dell Medical School Health Transformation Research Institute, Dell Medical School, The University of Texas at Austin, Austin, TX*

10:30 AM ‐ 12:30 PM

**Objective**: Seeking to curb severe maternal morbidity (SMM) and mortality, Texas adopted statutory levels of maternal care rules. We evaluated if the state‐mandated maternal level of care designation law decreased SMM.

**Study Design**: Retrospective cross‐sectional analysis of all delivery hospitalizations in Texas from 2016‐2023 using quarterly discharge data in the Texas Inpatient Public Use Data File. Out of 221 designated hospitals, we included the 215 that reported deliveries in the study period (32 Level IV, 43 Level III, 86 Level II, 54 Level I). As 99% of facilities received their requested designation level, we assumed the same pre‐mandate level. Deliveries at sites that were not designated maternal facilities (89 total) were categorized Undesignated. We organized comorbidities by minimum recommended level of care (highest Level IV, lowest ‘No risk’) using criteria in the Texas Administrative Code, ACOG/SMFM Consensus, and literature. We chose a baseline of 2016(Q1) to 2018(Q4) due to (Q4)2015 introduction of ICD‐10 and 2018 Texas rule adoption, then a post‐intervention of 2021(Q4) to 2023(Q3) based on the September 2021 implementation deadline. We used the CDC's definition of SMM and ICD‐10 codes to calculate SMM rates. We categorized patients into minimum level of care by their highest risk comorbidity and used a z‐test for two proportions to compare the SMM rates for each risk group by delivery at appropriate or inappropriate level of care.

**Results**: SMM rates increased overall during the study period. SMM decreased among patients with maternal risk levels II‐IV regardless of appropriate level of care, except for a stable rate in risk level IV patients at inappropriate level facilities (Figure 1). Maternal risk level I patients and those with no risks experienced increases in SMM (Table 1).

**Conclusion**: SMM decreased for high‐risk patients, particularly when delivered at appropriate level facilities, while SMM increased for low‐risk patients and overall. As low‐risk patients constitute the overwhelming share of births, this cohort may benefit from targeted policies.

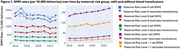

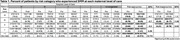

## 890 | Development of a Novel Machine‐Learning‐Based Prediction Model to Stratify Risk for Adverse Perinatal Outcomes

Rajet Vatsa^1^; Siguo Li^2^; Jessica L. Cohen^3^; Mark A. Clapp^2^

*^1^Harvard University, Boston, MA; ^2^Massachusetts General Hospital, Boston, MA; ^3^Harvard T.H. Chan School of Public Health, Boston, MA*

10:30 AM ‐ 12:30 PM

**Objective**: An effective risk assessment tool for perinatal deaths is critically needed, given that over one‐third of stillbirths after 28 weeks of gestation are potentially preventable. While current guidelines emphasize the role of clinical risk factors (e.g., medical comorbidities), the value of geographic, sociodemographic, and utilization‐related factors in risk stratification is less known.

**Study Design**: We utilized electronic health records data from a large integrated healthcare system between 4/2017 and 6/2023. The analysis was restricted to births ≥ 28 weeks. We developed and internally validated two classes of machine learning models to predict a composite adverse outcome, including stillbirth, early newborn death, and 5‐minute Apgar < 7, using features known before 28 weeks. The first class (“clinical”) only leveraged clinical risk factors (e.g., chronic hypertension) known to be associated with perinatal death, while the second class (“clinical+”) added additional geographic (e.g., distance to hospital), sociodemographic (e.g., household income), and utilization‐related (e.g., prenatal care use) features. We tested and compared the top‐performing models in each class with respect to their ability to prospectively stratify the risk of adverse perinatal outcomes.

**Results**: The clinical+ model (AUC: 0.63 [95%‐CI: 0.59‐0.69]) had modest discrimination overall, similar to the clinical model (AUC: 0.58 [95%‐CI: 0.53‐0.64]). However, the clinical+ model stratified pregnancies into quartiles of predicted risk with significantly different rates of the composite adverse outcome compared to the clinical model, which did not. For the clinical+ model, the adverse outcome rate was 0.6% (95%‐CI: 0.3‐0.9%) in the lowest and 4.6% (95%‐CI: 3.9‐5.3%) in the highest risk quartile.

**Conclusion**: A machine‐learning‐based model developed using clinical, geographic, sociodemographic, and utilization‐related factors known at the start of the third trimester may more accurately stratify risk than using clinical factors alone. Prospective and external validation studies are needed to demonstrate its utility in practice.

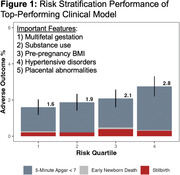

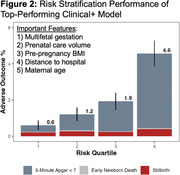

## 891 | Antepartum Fetal Surveillance Use and Rates of Stillbirth by Predicted Risk of Adverse Perinatal Outcomes

Rajet Vatsa^1^; Siguo Li^2^; Jessica L. Cohen^3^; Mark A. Clapp^2^

*^1^Harvard University, Boston, MA; ^2^Massachusetts General Hospital, Boston, MA; ^3^Harvard T.H. Chan School of Public Health, Boston, MA*

10:30 AM ‐ 12:30 PM

**Objective**: Over a third of stillbirths after 28 weeks of gestation are thought to be potentially preventable, with antepartum fetal surveillance (AFS) constituting the primary clinical tool for prevention. We sought to compare the rates of stillbirths among those who did and did not receive AFS, stratified by their baseline risk.

**Study Design**: We utilized electronic health records data from a large integrated healthcare system between 4/2017 and 6/2023 to evaluate the association between AFS, specifically biophysical profiles and nonstress tests conducted in the outpatient setting prior to one's admission for childbirth, and stillbirths occurring after 28 weeks of gestation. Patients were stratified into quartiles of risk for an adverse perinatal outcome using a machine‐learning‐based model that incorporated information known by the start of the third trimester. Characteristics of patients at highest risk were compared between those who did and did not receive AFS.

**Results**: Overall, 51% of pregnancies received at least one AFS test. After prospectively stratifying pregnancies into risk quartiles, rates of AFS utilization ranged from 38% in the lowest‐risk to 66% in the highest‐risk quartile. Among pregnancies at highest risk, there was a significant negative association between receipt of AFS and rates of stillbirth: 8.8 per 1,000 births (95% CI: 3.4‐14.3) among un‐surveilled pregnancies vs. 1.4 per 1,000 births (95% CI: 0.0‐2.9) among surveilled pregnancies (p = 0.01). High‐predicted‐risk individuals who did not receive AFS lived further from the hospital and obtained less prenatal care but had lower rates of nearly all traditional clinical risk factors.

**Conclusion**: Using a machine‐learning‐based model to stratify risk of adverse perinatal outcomes, we demonstrated that AFS was associated with an over 80% lower rate of third‐trimester stillbirth among those at highest risk. Further studies are required to causally identify the effect of AFS by risk; however, these findings suggest a potential role for policies to address barriers that may prevent high‐risk individuals from accessing AFS to prevent stillbirth.

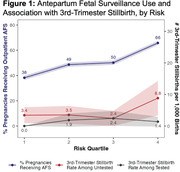

## 892 | Maternal Mental Health After Stillbirth Delivery

Rana Jawish; Amanda A. Allshouse; Robert M. Silver

*University of Utah, Salt Lake City, UT*

10:30 AM ‐ 12:30 PM

**Objective**: Stillbirth has a significant impact on maternal mental health. The decision to pursue autopsy following stillbirth can yield knowledge about cause of death (COD), both of which may impact subsequent mental health outcomes for parents.  Our objective was primarily to examine associations between autopsy decision and known cause of death with subsequent poor mental health after stillbirth, and secondarily to determine characteristics and factors associated with poor mental health after stillbirth.

**Study Design**: This was a secondary analysis of singleton stillbirth cases in the Stillbirth Collaborative Research Network case‐control study. Primary exposures were decision to perform autopsy (none, partial, full) and autopsy results (known COD, otherwise.) The primary outcome was a mental health composite assessed 1‐3 years after the index pregnancy (Edinburgh depressive scale, elevated grief, elevated distress, low post‐traumatic growth, or self‐reported increase in drinking or smoking).

**Results**: Of 620 parents, 272 participated in the follow up interview (44%), with autopsy decision full (66%), partial (19%), and none (14%), and known COD 57% (82% of full autopsy, 52% of partial, 0% of no autopsy). 54% had poor mental health at follow up including depression (26%), elevated distress (25%), grief (18%), increased smoking or drinking (15%), and low resilience (11%).) Neither autopsy decision nor knowledge from autopsy were associated with mental health at follow up (Table 1).   Factors associated with poor mental health after stillbirth were childhood physical/emotional abuse/trauma, angry feeling internalization, chronic autoimmune conditions, prior mental health conditions, low income, and feeling blamed for stillbirth (Table 2). When modeled jointly, only childhood trauma (OR: 4.1 (1.6‐10.7)) and low income (2.3 (1.3‐4.0)) remained significantly associated with poor mental health.

**Conclusion**: Adverse mental health outcomes were remarkably high after stillbirth and were associated with low income, and prior trauma. Additional follow‐up and screening tools could optimize mental health after stillbirth.

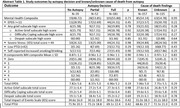

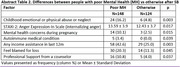

## 893 | Obstetric Emergencies, When to Provide Abortions, and State Restrictions: a Survey of Us Emergency Physicians

Rauvynne N. Sangara^1^; Priscilla Garza^2^; Emma Lantos^2^; Sophie Terp^2^; Sarah Axeen^2^; Brian Nguyen^2^

*^1^Division of Maternal Fetal Medicine, Department of Obstetrics and Gynecology, University of Southern California, Los Angeles, CA; ^2^University of Southern California, Los Angeles, CA*

10:30 AM ‐ 12:30 PM

**Objective**: To explore emergency medicine (EM) provider attitudes about obstetric emergencies, the appropriateness of their management via abortion, and the influence of abortion restrictions on clinical decision making.

**Study Design**: We distributed an anonymous, cross‐sectional, electronic survey to EM providers nationwide using social media platforms and specialty listservs. Using Likert‐type scales, the survey listed several obstetric presentations; participants then reported (1) which might qualify as obstetric emergencies, (2) the appropriateness of abortion as management for these conditions, (3) the availability of abortion at their site, and (4) their beliefs on obligation to provide abortion if against state law. We examined variations based on the abortion permissiveness or restrictiveness of the participant's state of practice via bivariate analysis.

**Results**: Most respondents (N = 203) identified as non‐Hispanic white (69%), female (57%), attending physicians (74%), in abortion‐permissive states (61%). A majority thought that ruptured ectopic pregnancy (96%), hemorrhage (82%), septic abortion (59%), pulmonary embolism (51%), and hypertensive disease (51%) were obstetric emergencies requiring immediate, life‐saving intervention. Abortion was viewed as often/always appropriate for ectopic pregnancy (97%), septic abortion (89%), incomplete abortion (83%), hemorrhage (65%), hypertensive disease (52%), pregnancy loss (50%), infection (45%), preterm premature rupture of membranes (38%), and pulmonary embolism (11%). Many (21%) believed legal restrictions would prevent EM providers from considering abortion for obstetric emergencies; 9% reported that abortion restrictions personally affected their clinical decision‐making. Most (84%) agreed that EM clinicians must provide and/or facilitate abortion for obstetric emergencies, even if prohibited by state law. Responses did not vary by abortion permissive versus restrictive states (p = 0.48).

**Conclusion**: EM providers consider many diagnoses to be obstetric emergencies with abortion being an appropriate treatment in many cases, even if prohibited by state law.

## 894 | Does First Trimester Blood Pressure Affect Uterine Artery Doppler Changes During Pregnancy?

Rebecca Horgan^1^; Erkan Kalafat^2^; Elena Sinkovskaya^1^; Alfred Z. Abuhamad^1^; George R. Saade^1^

*^1^Macon & Joan Brock Virginia Health Sciences Eastern Virginia Medical School at Old Dominion University, Norfolk, VA; ^2^Koc University Hospital, Istanbul, Istanbul*

10:30 AM ‐ 12:30 PM

**Objective**: First trimester blood (BP) has been shown to have an association with preeclampsia (PE) development later in pregnancy. Our objective was to determine whether this association is dependent on change in uterine perfusion which is also known to be associated with PE.

**Study Design**: This was a prospective longitudinal cohort study within the Human Placenta Project which enrolled patients at ≤ 13+6 weeks’ gestation. Data was obtained by trained research coordinators. At the 1st trimester study visit, BP was measured and categorized according to the American Heart Association (AHA) classification as normal, elevated, or hypertension which included stage 1 and stage 2. Uterine artery Doppler pulsatility index (UtA‐PI) was obtained at eight timepoints during pregnancy beginning in the first trimester (Figure). Doppler values were modeled with mixed‐effects regression and longitudinal trajectories were formed with k‐means clustering. Additive effect of Doppler values to blood pressure was assessed with Cochrane‐Armitage test for trend. A mediation analysis was undertaken to elucidate how much of the effect of blood pressure (BP) is exerted over Doppler values.

**Results**: 608 pregnancies were included in the analysis, with 60 developing PE. Longitudinal assessment of UtA‐PI revealed 3 trajectory groups (normal, elevated, abnormal, Figure). Addition of UtA‐PI to first trimester blood pressure categorization improved the risk classification for AHA elevated BP (PE developed in 0.0%, 9.8%, 20.0% of UtA normal, elevated, and abnormal, P = 0.042) and AHA hypertension categories (PE developed in 15.0%, 17.1%, 38.2% of UtA normal, elevated, and abnormal, P = 0.021) (Figure). On the mediation analysis, the odds ratio of elevated BP (OR: 2.23, 95% CI: 0.96‐4.88 vs. 2.23, 95% CI: 0.97‐4.84) and hypertension categories (OR: 4.94, 95% CI: 2.69‐9.25 vs. 4.89, 95% CI: 2.68‐9.11) changed very little with the addition of Doppler values.

**Conclusion**: First trimester BP categorization according to AHA is an independent risk factor for preeclampsia and related risk is not exerted over uterine artery function.

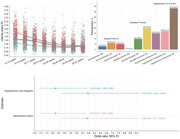

## 895 | First and Third Trimester Ultrasound Markers for Prediction of Placenta Accreta Spectrum & Clinical Outcomes

Rebecca Horgan^1^; Elizabeth Miller^2^; Yara Hage Diab^1^; Juliana Martins^3^; George R. Saade^1^; Camille Kanaan^1^; Jerri A. Waller^1^

*^1^Macon & Joan Brock Virginia Health Sciences Eastern Virginia Medical School at Old Dominion University, Norfolk, VA; ^2^Macon & Joan Brock Virginia Health Sciences Eastern Virginia Medial School at Old Dominion University, Norfolk, VA; ^3^Macon & Joan Brock Virginia Health Sciences Eastern Virginia Medial School at Old Dominion University, Virginia Beach, VA*

10:30 AM ‐ 12:30 PM

**Objective**: To evaluate the predictive accuracy of ultrasound markers for placenta accreta spectrum (PAS) and associated clinical outcomes in patients with prior cesarean delivery and placenta previa.

**Study Design**: This retrospective cohort study included patients with prior cesarean delivery and placenta previa who received prenatal care at our tertiary center from Jan 2015 to June 2022. Ultrasound markers evaluated in the first and third trimester included lacunae presence/count, bladder wall interruption, loss of retroplacental clear zone, bridging vessels, and parametrial vascularization. Ultrasounds were reviewed blindly by an MFM physician. Primary outcome was PAS and secondary outcomes included PAS severity (accreta, increta, percreta), visceral injury during surgery (bladder, ureter, bowel), and severe blood loss (EBL >1000 mL or blood transfusion). Shallow gradient booster models assessed prediction model performance with area under the curve (AUC) values and feature importance plots.

**Results**: Of 111 pregnancies, the final diagnosis was PAS in 33 and placenta previa without PAS in 78. Third‐trimester ultrasound markers were highly predictive of PAS (AUC: 0.92, 95% CI: 0.86‐0.98) vs. first‐trimester markers (AUC: 0.79, 95% CI: 0.63‐0.93) (P = 0.047). The most predictive feature of PAS severity was bridging vessels, followed by loss of clear zone. Increta or percreta diagnosis rates were 34.4% (both features present), 18.2% (one feature), and 4.4% (absent). Visceral injury prediction was highest with loss of clear zone, followed by bridging vessels: 21.9% (both present), 18.2% (one), 2.9% (absent). Severe blood loss predictors were bridging vessels and parametrial vascularization: 89.7% (both present), 82.1% (one), 44.4% (absent).

**Conclusion**: Third‐trimester ultrasound is an excellent predictor of PAS diagnosis. PAS ultrasound features vary in predictive potential for clinical outcomes. Top predictors of PAS severity, visceral injury, and severe blood loss are bridging vessels, loss of clear zone, and parametrial vascularization.

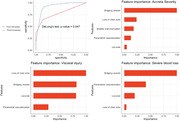

## 896 | Machine Learning Prediction of Gestational Hypertensive Complications at Point‐of‐Care

Reetam Ganguli^1^; Julia Sroda Agudogo^2^; Stephen Wagner^2^

*^1^Elythea, San Jose, CA; ^2^Beth Israel Deaconess Medical Center, Boston, MA*

10:30 AM ‐ 12:30 PM

**Objective**: Hypertensive disorders of pregnancy (HDP) pose significant risks to both maternal and fetal health, potentially leading to early induction and NICU admission. Early identification and intervention have been shown to prevent up to 80% of preeclampsia cases. This study aims to develop and evaluate a machine learning model to predict preeclampsia using routinely collected prenatal variables.

**Study Design**: An extreme gradient boosting model was developed to predict the risk of HDP, inclusive of preeclampsia and gestational hypertension. The model was trained on data from the American College of Surgeons National Surgical Quality Improvement Program database comprising prenatal and demographic variables from a large cohort of pregnant women from 2018 to 2021 and tested on data from 2022.

Inclusion criteria were term deliveries without missing data for hypertensive complications. Patients missing hypertensive complication data were excluded. The model incorporated clinical history, demographic information, and early‐stage comorbidities. To address class imbalance, weight scaling algorithms were utilized and patients from 2014‐2017 with hypertensive complications to enhance the representation of the positive class, with the exception of 2015 patients due to corrupted data.

**Results**: 35 factors across 11,298,212 patients were used to develop the model. Of these patients, 1,551,321 (13.7%) had prenatal hypertensive complications, The model was tested on 3,258,442 total patients, of which 907,974 (27.9%) had hypertensive complications. The XGBClassifier model achieved an AUC‐ROC of 0.70, with a 74.9% accuracy, 48.2% sensitivity and 0.75 F1 score.

**Conclusion**: Early‐stage predictive models used at the point of care can detect nearly half of all hypertensive complications at the point of care with discriminative ability. Early identification through such models may allow for targeted clinical interventions, harboring potential for the prevention of hypertensive cases and cost savings for payors.

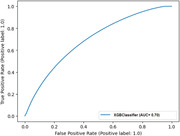

## 897 | Transfer Status and Severe Hemorrhage Risk in Cesarean Section Patients

Reetam Ganguli^1^; Joshua Woo^2^; Alice Lin^2^; Maguire Anuszewski^2^; Julia Sroda Agudogo^3^; Stephen Wagner^3^

*^1^Elythea, San Jose, CA; ^2^Warren Alpert Medical School, Brown University, Providence, RI; ^3^Beth Israel Deaconess Medical Center, Boston, MA*

10:30 AM ‐ 12:30 PM

**Objective**: Transfer status influences severe hemorrhage risk in cesarean section patients, highlighting the importance of tailored preoperative management strategies to mitigate complications. This study evaluated the association between transfer indications and severe hemorrhage necessitating transfusion in patients undergoing cesarean sections.

**Study Design**: A retrospective cohort study was conducted using data from the American College of Surgeons National Surgical Quality Improvement Program (ACS NSQIP) database from January 2009 to December 2021. Included were patients undergoing cesarean sections identified by Current Procedural Terminology (CPT) codes 59510, 59514, 59515, 59618, 59620, and 59622. Exclusion criteria included missing data for the outcome variable indicating severe hemorrhage necessitating transfusion. Chi‐square tests determined statistical significance, and odds ratios (OR) with 95% confidence intervals (CI) quantified the risk of severe hemorrhage for patients with different transfer statuses compared to patients who were not transferred.

**Results**:  In our cohort of 43,713 patients, 61.27% were not transferred (admitted from home). Transfers from home/permanent residence had a significantly lower risk of severe hemorrhage (OR = 0.755, 95% CI: 0.677‐0.842, p < 0.001). Conversely, transfers from outside emergency departments (OR = 4.307, 95% CI: 2.057‐9.020, p < 0.001) and other facilities (OR = 5.653, 95% CI: 1.263‐25.302, p = 0.079) exhibited markedly higher risk. Acute care hospital transfers were also associated with a significantly lower risk of severe hemorrhage (OR = 0.336, 95% CI: 0.218‐0.517, p < 0.001). Transfers from acute care hospital inpatient and unknown transfer reasons did not show statistically significant differences.

**Conclusion**: Cesarean deliveries transferred from outside emergency departments and other facilities are at higher hemorrhage risk, making enhanced preoperative management vital. These findings emphasize considering transfer status in risk stratification and preoperative planning to mitigate hemorrhage risks in cesarean section patients.

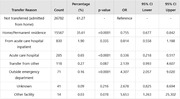

## 898 | Weighing the Options: A Six‐Year Single‐Center Analysis of Second No‐Call Non‐Invasive Prenatal Testing Results

Ronan Daly^1^; Fianat Bligh^2^; Sirisha Bellamkonda^2^; Claire O'Rourke^2^; Elizabeth Tunney^1^; Patrick Dicker^1^; Sieglinde Mullers^3^; Fergal D. Malone^4^; Karen Flood^5^

*^1^Royal College of Surgeons in Ireland, Dublin, Dublin; ^2^RCSI Rotunda Hospital, Dublin, Dublin; ^3^Rotunda Hospital Dublin, Dublin, Dublin; ^4^Royal College of Surgeons in Ireland, Dubiln, Dublin; ^5^Royal College of Surgeons in Ireland, Dublin, DE*

10:30 AM ‐ 12:30 PM

**Objective**: While a low‐risk Non‐invasive Prenatal Screening (NIPS) can offer reassurance for expectant parents, a no‐call result (NCR) can cause significant anxiety. A definitive result is not guaranteed with repeat NIPS and a second NCR may compound patient uncertainty. This study aims to inform clinical practice and counselling for patients who receive NCRs by analyzing the demographics and outcomes of NCRs over the past six years.

**Study Design**: This retrospective cohort study was conducted at a large obstetric hospital in Ireland, utilizing a SNP‐based cell‐free fetal DNA test. We identified NCRs through an integrated electronic healthcare record. Data were collected on all patients who received NCRs from November 2017 to January 2024.

**Results**: 10,025 NIPS were conducted with 376 of patients receiving an initial NCR (3.8%). The median gestational age at first NIPS draw was 10.4 weeks [IQR: 9.7,11.7] with no significant difference across weight groups (p = 0.566). 354 (94%) underwent a second NIPS with a result yielded in 76% (n = 269) and 85 patients (24%) receiving a second NCR. A significant difference was found in the distribution of second NCRs by weight (p = 0.001), with 40% of patients in the > 90 kg group receiving a further NCR. Fetal fraction (FF) at second NIPS decreased significantly according to maternal weight, with a median FF of 6.5% [IQR: 4.3,10.0] in patients < 65kgs and a median FF of 3.1% [IQR: 2.5,4.3] in those > 90kgs (p = 0.001). 24% of second NCRs (n = 20) underwent invasive testing, with half of these pregnancies affected by fetal aneuploidy. Table 1 summarizes the trends in NCRs and FF according to maternal weight.

**Conclusion**: Despite repeat NIPS often providing a definitive result following an initial NCR, our study shows a significant correlation between maternal weight above 90kgs and the likelihood of a second NCR. Given this 40% risk of a further NCR and the underlying risk of fetal aneuploidy, we recommend offering invasive diagnostic options following an initial NCR to provide certainty for this patient group.

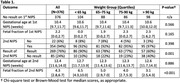

## 899 | Assessing Antenatal and Postnatal Growth in Severe and Non‐Severe Fetal Growth Restriction

Roopjit K. Sahi; Manesha Putra; John Hobbins

*University of Colorado Anschutz Medical Campus, Aurora, CO*

10:30 AM ‐ 12:30 PM

**Objective**: Our aim was to determine if severity of fetal growth restriction (FGR) is related to growth trajectory after birth, and whether an in‐utero marker of body composition, including thigh volume, correlates with growth after birth.

**Study Design**: In this retrospective observational study, FGR pregnancies were included, and subdivided as severe or non‐severe (severe FGR indicating estimated fetal weight, EFW or abdominal circumference, AC less than 3^rd^ percentile, with or without Doppler abnormalities). Measures of EFW, AC, total thigh volume (TTV), lean mass volume (LMV) were extracted from antenatal scans. Fat mass volume (FMV) was calculated by subtracting TTV from LMV. Postnatally, weight (and z‐scores) was measured at birth, 1‐3 months, 6 months, 1‐year, 3 years, and 5 years of age. Median values (and interquartile range) of indices were compared between both groups. Using Pearson correlation analysis, correlation coefficients (r) were calculated between antenatal and postnatal parameters.

**Results**: The cohort includes 42 severe and 14 non‐severe FGR pregnancies. In the severe FGR group, median values of TTV, LMV, FMV, EFW, EFW percentile, and AC percentile were lower compared to those in non‐severe group (p< 0.05); ultrasounds done at the median gestational age of 35.93 weeks in both groups. Incidence of ‘accelerated growth’ (increase in weight z‐score greater than 0.8) was higher in severe FGR group (80.9% versus 35.7%, p 0.01), typically observed between 6 months‐3 years of age (Figure 1(a), 1(b)). By 5 years of age, weight (and z‐score) of infants from severe group was lower than non‐severe group (p 0.04). Birthweight (BW) correlated well with TTV (r 0.62, p < 0.01), and EFW percentile (r 0.69, p < 0.01). However, no significant correlations were observed during subsequent follow‐ups.

**Conclusion**: In severe FGR group, despite accelerated growth of infants between 6 months‐3 years, the median weight is lesser compared to non‐severe group at 5 years of age, which could result in false reassurance in earlier years. In‐utero markers, TTV and EFW, correlate significantly and similarly with BW.

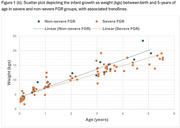

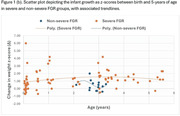

## 900 | Prediction Model for Spontaneous Onset of Active Labor After Pre‐Labor Rupture of Membranes at Term

Roza Berkovitz‐Shperling^1^; Omri Dominsky^2^; Yariv Yogev^3^; Shai Ram^4^

*^1^Tel Aviv Sourasky Medical Center, Tel Aviv Sourasky Medical Center, Tel Aviv; ^2^Tel Aviv Sourasky Medical Center, Tel Aviv Sourasky Medical Center, HaMerkaz; ^3^Lis Maternity Hospital, Sourasky Medical Center, Tel Aviv University, Tel Aviv Sourasky Medical Center, Tel Aviv; ^4^ICHILOV, Tel Aviv, HaMerkaz*

10:30 AM ‐ 12:30 PM

**Objective**: Term pre‐labor rupture of membranes (PROM) is a significant event that may pose risks for maternal and fetal complications. We aimed to develop a predictive model for the spontaneous onset of active labor within 24 hours following membrane rupture at term.

**Study Design**: 1. 1. A retrospective cohort study in a single, university‐affiliated tertiary medical center which included women who presented at term (37‐41 weeks) with PROM and were managed conservatively upon patient request, without induction of labor, (January 2011–December 2023).

2. The primary outcome was spontaneous onset of active labor, defined as cervical dilation to 6 centimeters (cm), within 24 hours after membrane rupture.

3. Exclusion criteria included: women who received oxytocin during the 24 hours after PROM and before reaching active labor (6 cm dilatation), women with non‐clear amniotic fluid, the necessity for cesarean delivery, suspected infection and non‐reassuring fetal monitoring.

4.Potential risk factors including demographic, medical and obstetric characteristics were examined.

5. Repeat amniotomy was defined as the need for additional amniotomy, after admission with PROM.

6. Prediction model was built using a backward variable selection based upon significant risk factors which were found in the multivariable logistic regression (*P* < .05).

**Results**: 1. Overall, during the study period 10,633 women met the inclusion criteria and were enrolled.

< 2.Within 24 hours after PROM, 6,961 (65.4%) had spontaneous onset of active labor.

< 3. Spontaneous onset of labor after PROM was associated with parity, cervical dilatation and cervical length at admission, repeat amniotomy, and GBS status.

< !4. The prediction model for onset of labor within 24 hours after PROM had an area under the curve (AUC) of 0.813 with accuracy of 75.94% 

**Conclusion**: The predictive model effectively identifies women at higher chance of spontaneous active labor within 24 hours following PROM. This model may improve clinical decision‐making and labor management strategies after term PROM.

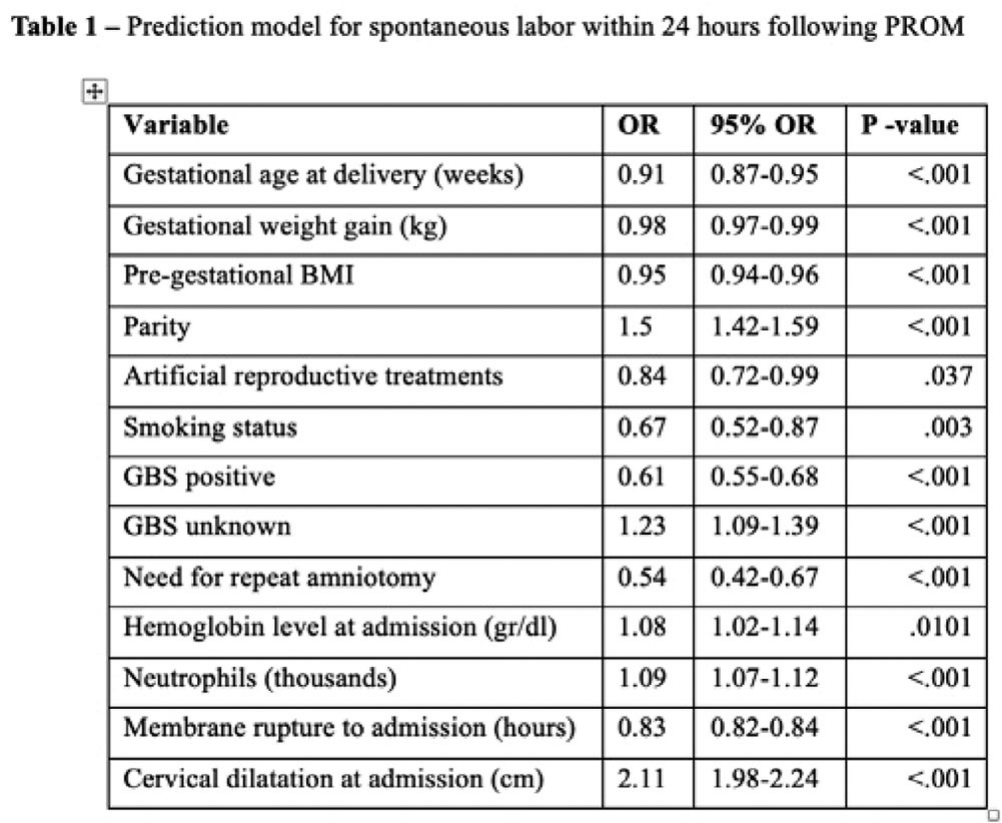

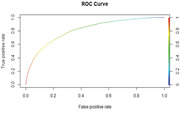

## 901 | Birth Location and Risk of Infant Death in Twin Pregnancies

Rang Kim^1^; Stephen Contag^2^; Kriti N. Vedhanayagam^3^; Sergio Karageuzian^4^; Ilish Gedestad^5^; Synia Chunn^3^; Megan Marquez^1^; Ruofan Yao^3^

*^1^Loma Linda University School of Medicine, Loma Linda University, CA; ^2^University of Minnesota, Minneapolis, MN; ^3^Loma Linda University School of Medicine, Loma Linda, CA; ^4^Loma Linda University School of Medicine, Pasadena, CA; ^5^Loma Linda University School of Medicine, Redlands, CA*

10:30 AM ‐ 12:30 PM

**Objective**: To evaluate the association between birth location (birth center vs. hospital) and the risk of infant death in twin pregnancies.

**Study Design**: This retrospective cohort study analyzed data from the NCHS linked multiple birth and infant death database from 2016 to 2020. Inclusion criteria were matched twin pregnancies without congenital anomalies. Infant mortality was the primary outcome. Chi‐square tests and logistic regression models were used to assess the association between birth location and infant mortality, adjusting for gestational age at delivery (GA ≤ 34 weeks and GA > 34 weeks) and order of birth (Twin A and Twin B).

**Results**: Among 607,355 twin births, the overall rate of neonatal death was 0.97%. Neonatal death was higher in birth centers (3.04%) compared to hospitals (0.96%) (p < 0.001). For GA > 34 weeks, neonatal death was 0.82% in birth centers vs. 0.18% in hospitals (p < 0.001, OR 4.59 [95% CI 2.75‐7.67]). For GA ≤ 34 weeks, neonatal death was 13.68% in birth centers vs. 4.24% in hospitals (p < 0.001, OR 3.58 [95% CI 2.67‐4.81]). In Twin A, neonatal death was 3.65% in birth centers vs. 0.93% in hospitals (p < 0.001, OR 4.06 [95% CI 3.00‐5.50]), while in Twin B, neonatal death was 2.30% in birth centers vs. 1.00% in hospitals (p < 0.001, OR 2.32 [95% CI 1.53‐3.52]).

**Conclusion**: Birth center delivery is associated with a significantly higher risk of infant mortality in twin pregnancies compared to hospital delivery. This increased risk persists across different gestational ages and the order of birth. These findings suggest that hospital deliveries might offer safer outcomes for twins, particularly those at higher risk of neonatal complications.

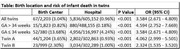

## 902 | is Expectant Management of Late Pprom an Option in Patients with Gbs Colonization?

Sabina Razdolsky; Elior Eliasi; Elana Minic; Hadel Jamal; Ariel Mani; Miriam Lopian

*Mayanei Hayeshua Medical Center, Mayanei Hayeshua Medical Center, Tel Aviv*

10:30 AM ‐ 12:30 PM

**Objective**: To determine if  carriers of group B streptococcus (GBS) have adverse obstetric and neonatal outcomes when preterm premature rupture of membranes (PPROM) occurs between 34 to 37 weeks of pregnancy.

**Study Design**: A retrospective cohort study was conducted at a single university‐affiliated medical center between 2012 and 2022. Patients who had preterm premature rupture of membranes (PPROM) between 34 to 37 weeks of pregnancy carrying a singleton gestation, with no contraindications to expectant management were included in the study group. According to the department protocol, all patients with PPROM receive 1G of IV Azithromycin once and 4g of IV penicillin every 4 hours. Antibiotics are discontinued in patients negative for GBS and continued for one week in GBS positive patients. Outcomes were compared to those with a negative GBS culture. Baseline demographic characteristics were compared between the groups. The primary outcome was latency from PPROM till delivery.  Secondary outcomes included cesarean delivery, composite adverse maternal outcome (PPH, chorioamnionitis, abruption of placenta) and composite neonatal outcome (NICU admission, respiratory distress syndrome, early onset GBS disease).

**Results**: Two hundred and seventeen patients were included in the study. 98(45.2%) were GBS carriers and 119 (54.8%) were noncarriers. There were no significant between group differences in mean maternal age (27.3±6,29.7±6.1;p = 0.37) or gestational age at delivery (252 days±5,252 days±6;p = 0.4), rate of diabetes (10.1%,10.2%;p = 0.97 ) neonatal mean birthweight (2602g,2698g;p = 0.32).

There were no significant differences between the groups for the primary outcome of duration from membrane rupture until delivery (2.83 ± 2.9 days,3.03 ± 2.9 days;*P* = 0.98) nor  the rate of cesarean delivery (9.8%,10.9%;p = 0.45) or in composite  maternal (2.7%,1.9%;p = 0.26) or neonatal outcomes(23%,22%;p = 0.34).

**Conclusion**: Expectant management of late PPROM in GBS carriers is not associated with an increased risk of adverse maternal or neonatal outcomes.

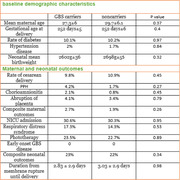

## 903 | Perinatal Mortality and Other Severe Outcomes Following Planned Birth at 39 Weeks Versus Expectant Management

Kylie Crawford^1^; Waldemar A. Carlo^2^; Anthony O. Odibo^3^; Aris T. Papageorghiou^4^; William Tarnow‐Mordi^5^; Sailesh Kumar^6^; On behalf of the Mater Research Institute and The University of Queensland

*^1^Mater Research Institute‐The University of Queensland, Brisbane, Queensland; ^2^University of Alabama at Birmingham, Birmingham, AL; ^3^University of Missouri ‐ Kansas City, Leawood, KS; ^4^University of Oxford, Oxford, England; ^5^The University of Sydney, Sydney, New South Wales; ^6^The University of Queensland, Brisbane, Queensland*

10:30 AM ‐ 12:30 PM

**Objective**: To investigate differences in perinatal and maternal outcomes in low‐risk women following **any type of planned birth** (induction of labour or scheduled caesarean section) at 39^+0^–39^+6^ weeks versus expectant management.

**Study Design**: Retrospective cohort study. Study outcomes were perinatal mortality, severe neonatal neurological morbidity, severe neonatal non‐neurological morbidity, severe maternal outcome, maternal‐infant separation, severe perineal trauma, shoulder dystocia, and caesarean birth. Subgroup analyses according to parity and birthweight were also performed.

**Results**: Of 472,520 low‐risk pregnancies >39^+0^ weeks planned birth at 39^+0^–39^+6^ weeks occurred in 97,438 (20.6%) women of whom 39,697 (40.7%) underwent induction of labour and 57,741 (59.3%) had scheduled caesarean delivery. Compared with expectant management, planned birth at 39^+0^–39^+6^ weeks was associated with lower odds of perinatal mortality (aOR 0.48; 95%CI 0.30, 0.76, p = 0.002), antepartum stillbirth (aOR 0.38; 95%CI 0.15, 0.97, p = 0.04), and intrapartum stillbirth (aOR 0.16; 95%CI 0.04, 0.66, p = 0.01). Planned birth was also associated with reduction in the odds of severe neonatal neurological morbidity (aOR 0.46; 95%CI 0.39, 0.53, p = 0.00004), severe neonatal non‐neurological morbidity (aOR 0.65; 95%CI 0.62, 0.68, p = 0.00004), severe maternal outcome (aOR 0.95; 95%CI 0.92,0.99, p = 0.008) and increased odds of maternal‐infant separation (aOR 1.04; 95%CI 1.00, 1.08, p = 0.075). Planned birth by induction of labour was associated with reduced odds of caesarean delivery (aOR 0.54; 95%CI 0.51, 0.58, p = 0.00004), severe perineal trauma (aOR 0.53; 95%CI 0.45, 0.63, p = 0.00004), and shoulder dystocia (aOR 0.73; 95%CI 0.64, 0.84, p = 0.00004).

**Conclusion**: Planned birth between 39^+0^–39^+6^ weeks in low‐risk women compared to expectant management was associated with lower odds of perinatal mortality, severe neonatal neurological and non‐neurological morbidity and severe maternal outcome. For women who were induced, it was associated with lower odds of severe perineal trauma, shoulder dystocia, and caesarean birth.

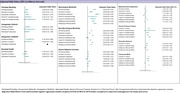

## 904 | Twin Twin Transfusion Syndrome (TTTS) Stage 1: Do We Manage Expectantly vs. Treat Immediately?

Sami Backley^1^; Eric P. Bergh^2^; Jimmy Espinoza^1^; Felicia V. LeMoine^1^; Neha Agarwal^3^; Gustavo Vilchez^4^; Percy N. Pacora‐Portella^5^; Anthony Johnson^6^; Edgar A. Hernandez‐Andrade^7^; Ashley Salazar^8^; Sen Zhu^9^; Ramesha Papanna^7^

*^1^Division of Fetal Intervention, Department of Obstetrics, Gynecology and Reproductive Sciences, McGovern Medical School at UTHealth Houston, Houston, TX; ^2^UT Health, Houston, TX; ^3^Division of Fetal Intervention, Department of Obstetrics, Gynecology and Reproductive Sciences, UTHealth McGovern Medical School, Houston, TX, USA, Houston, TX; ^4^UTHealth Houston, Houston, TX; ^5^Division of Fetal Intervention, Dpt Obstetrics,Gynecol and Reprod Sciences, McGovern Medical School at UTHealth Houston, Houston, TX; ^6^McGovern Medical School University of Texas Health Science Center at Houston (UThealth), Houston, TX; ^7^McGovern Medical School at UTHealth Houston, Houston, TX; ^8^Division of Fetal Intervention, Department of Obstetrics, Gynecology and Reproductive Sciences, McGovern Medical School at UT Health Houston, Houston, TX; ^9^McGovern Medical School at the University of Texas, Houston, TX*

10:30 AM ‐ 12:30 PM

**Objective**: Randomized controlled trial (RCT) for twin‐twin transfusion syndrome (TTTS) Stage 1 showed no difference in survival between expectant management and immediate fetoscopic laser surgery (FLS). However, higher perinatal survival with immediate FLS for TTTS Stage 1 has been reported and may justify immediate treatment. We aimed 1) to compare perinatal survival between expectant management and immediate treatment in TTTS Stage 1 and 2) to compare perinatal survival across TTTS Stages 1‐4 who underwent FLS for treatment of TTTS.

**Study Design**: Perinatal survival of TTTS Stage 1 undergoing expectant management reported by Stirnemann et al, were compared to perinatal survival rates at our center utilizing prospective data from 90 monochorionic, diamniotic twin pregnancies complicated by TTTS Stage 1. All pregnancies underwent immediate FLS between 2011‐2024. Pregnancies with cervical length < 1.5cm, non‐isolated TTTS, missing delivery or neonatal outcomes were excluded. The primary outcome was dual perinatal survival. Survival outcomes for TTTS Stage 1 were also compared to TTTS stages 2‐4.

**Results**: Our center's dual survival to birth was higher than the previously reported rates with expectant management of TTTS Stage 1 (Table 1). There was no difference in dual perinatal survival between our center and the previously reported rates in expectantly managed TTTS Stage 1 (Table 1); however, the sample size was not sufficient to detect the 10% difference in dual perinatal survival. Perinatal survival was higher in TTTS Stage 1 with immediate FLS when compared to other TTTS stages with FLS (Table 2).

**Conclusion**: Perinatal outcomes following immediate FLS for TTTS Stage 1 are more favorable when compared to other TTTS Stages and similar, or better, than expectant management. Immediate FLS should be considered a viable treatment option for TTTS stage 1, though a larger randomized trial to test the efficacy of immediate FLS should be considered.

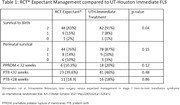

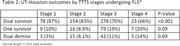

## 905 | Risk Factors for PPROM Following Laser Surgery in Twin‐to‐Twin Transfusion Syndrome

Sami Backley^1^; Eric P. Bergh^2^; Felicia V. LeMoine^1^; Neha Agarwal^3^; Gustavo Vilchez^4^; Ashley Salazar^5^; Edgar A. Hernandez‐Andrade^6^; Anthony Johnson^7^; Ramesha Papanna^6^; Jimmy Espinoza^1^

*^1^Division of Fetal Intervention, Department of Obstetrics, Gynecology and Reproductive Sciences, McGovern Medical School at UTHealth Houston, Houston, TX; ^2^UT Health, Houston, TX; ^3^Division of Fetal Intervention, Department of Obstetrics, Gynecology and Reproductive Sciences, UTHealth McGovern Medical School, Houston, TX, USA, Houston, TX; ^4^UTHealth Houston, Houston, TX; ^5^Division of Fetal Intervention, Department of Obstetrics, Gynecology and Reproductive Sciences, McGovern Medical School at UT Health Houston, Houston, TX; ^6^McGovern Medical School at UTHealth Houston, Houston, TX; ^7^McGovern Medical School University of Texas Health Science Center at Houston (UThealth), Houston, TX*

10:30 AM ‐ 12:30 PM

**Objective**: Preterm prelabor rupture of membranes (PPROM) is the most frequent complication of in‐utero surgery. The intraoperative use of distention media, such as lactated Ringer's, is thought to be caustic to the amniotic membrane. We sought to identify risk factors of PPROM following fetoscopic laser surgery (FLS) for twin‐twin transfusion syndrome (TTTS).

**Study Design**: This single‐center prospective cohort study included monochorionic‐diamniotic twin pregnancies who underwent FLS between 2011 and 2024 for management of TTTS. PPROM was defined as PROM < 34 weeks’ gestation. Pregnancies with missing delivery or neonatal outcomes were excluded. Maternal characteristics and perioperative factors were compared using comparative statistics. Multiple logistic regression was performed to evaluate the relationship between PPROM and the following factors: gestational age (GA) at FLS, amnioinfusion, and other confounders. Cox proportional hazard modeling was performed to evaluate factors influencing the procedure‐to‐delivery interval (days).

**Results**: PPROM occurred in 292 (39%) of 748 eligible pregnancies. Clinical characteristics are displayed in Table 1. GA at delivery was earlier and smaller amnioreduction volumes were seen in pregnancies with PPROM compared pregnancies without PPROM (Table 1). GA at FLS (adjusted odds ratio [aOR] 0.85, 95% confidence interval [CI] 0.77‐0.94), pre‐operative cervical length (aOR 0.98, 95% CI 0.96‐0.99), placental abruption (aOR 1.94, 95% CI 1.01‐3.72), collagen plug placement (aOR 2.17, 95% CI 1.18, 3.98) and chorioamnion membrane separation (CAS) (aOR 5.42, 95% CI 2.54‐11.56) were associated with the diagnosis of PPROM. PPROM, CAS, cervical length prior to FLS, placental abruption, and GA at FLS independently influenced procedure‐to‐delivery interval (Figure 1).

**Conclusion**: PPROM remains a major complication of FLS resulting in an earlier GA at delivery. CAS is the most important risk factor for PPROM after FLS surgery while amnioinfusion of ≥1 L is not a risk factor for PPROM following FLS for the management of TTTS.

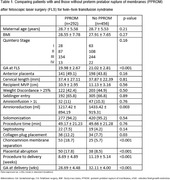

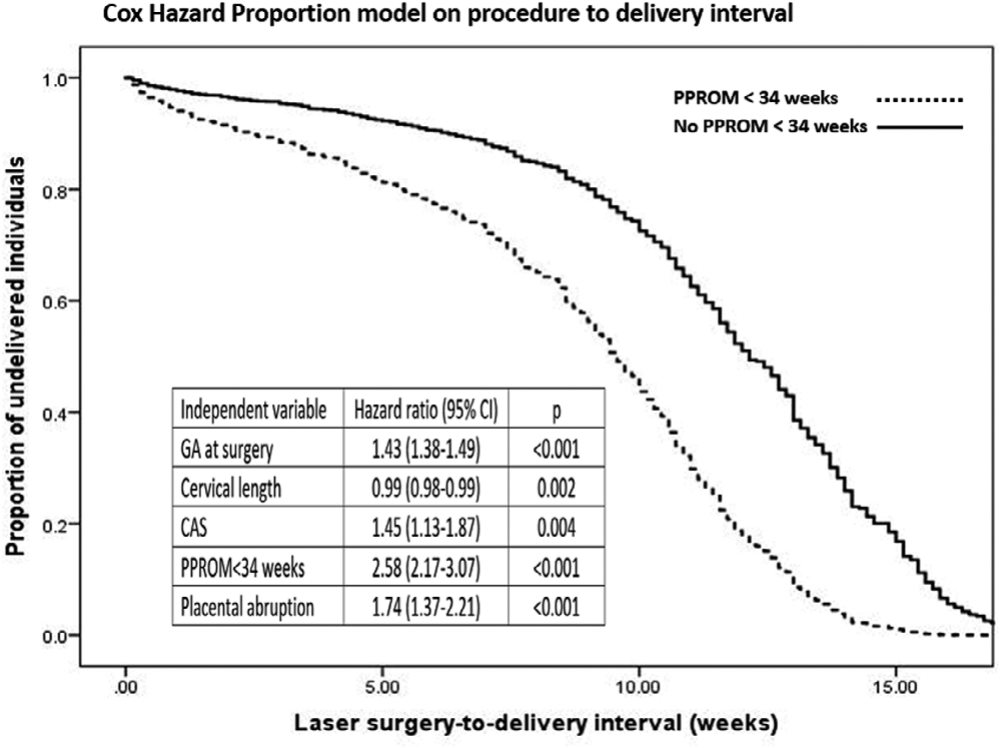

## 906 | Rurality is Associated with Increased Risk for Severe Maternal Morbidity Among Socially Vulnerable Communities

Sara EK Phillips; Bharti Garg; Sophie Neuner Weinstein; Aaron B. Caughey

*Oregon Health & Science University, Portland, OR*

10:30 AM ‐ 12:30 PM

**Objective**: To evaluate the association between severe maternal morbidity (SMM) and rural residence among communities of high social vulnerability.

**Study Design**: We performed a retrospective cohort study using California's linked vital statistics‐hospital discharge data (2018‐2020). The study population composed of individuals with singleton, non‐anomalous pregnancies delivered between 20‐44 weeks’ gestation with maternal residence (at time of birth) in communities deemed high on the Center for Disease Control's (CDC) Social Vulnerability Index (SVI) in 2020. Our primary outcome was a composite of SMM, defined using the CDC's published list of 21 indicators. Multivariable Poisson regression was used to calculate an adjusted risk ratio to assess whether rural residence in socially vulnerable communities was associated with SMM.

**Results**: Of the 605,301 births meeting inclusion criteria, 38,350 (6.3%) were from rural California. Rural Californians were more likely to be Non‐Hispanic White (34.2% vs 21.8%), less than 20 years of age (6.4% vs 4.3%), publicly insured (71.2% vs. 55.9%) and have an education level of high school or below (54.1% vs 41.9%). Compared to urban centers, the prevalence of SMM was greater in rural communities of high SVI (1.75% vs. 1.46%, p< 0.001). The indicator with the highest proportion was maternal blood transfusion (1.5% for rural, 1.1% for urban, p< 0.001). There was an increased risk of SMM in rural communities after adjusting for maternal race/ethnicity, age, education, insurance, parity, and preexisting diabetes and hypertension (aRR 1.21; 1.12‐1.31).

**Conclusion**: SVI reflects a community‐level resilience for combating external stressors. Our findings suggest that individuals from rural communities with high social vulnerability may face additional disadvantages compared to their urban counterparts. More research is needed to explore the root causes of rural maternal disparities, so we may design meaningful interventions to optimize the health of our rural patients.

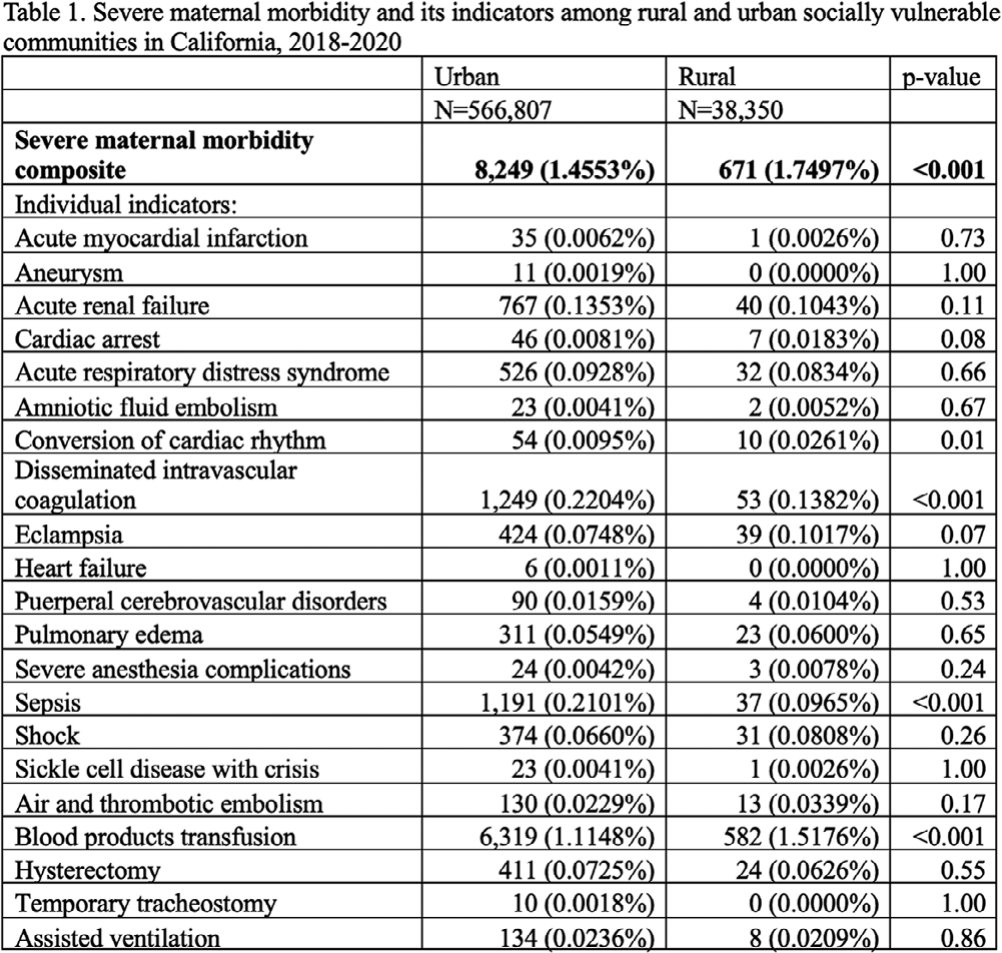

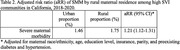

## 907 | Rurality is Associated with Decreased Risk for Adverse Neonatal Outcomes Among Socially Vulnerable Communities

Sara EK Phillips; Bharti Garg; Sophie Neuner Weinstein; Aaron B. Caughey

*Oregon Health & Science University, Portland, OR*

10:30 AM ‐ 12:30 PM

**Objective**: To assess the relationship between rural residence and adverse neonatal outcomes among socially vulnerable communities.

**Study Design**: This is a retrospective cohort study using California's linked vital statistics‐hospital discharge data (2018‐2020). Our study population included all singleton, non‐anomalous pregnancies delivered between 20‐44 weeks’ gestation with maternal residence in communities deemed high on the Center for Disease Control's (CDC) Social Vulnerability Index (SVI). The outcome of interest was an adverse neonatal composite including preterm delivery, neonatal intensive care unit (NICU) admission, small for gestational age, low birth weight, seizures, birth injury, and neonatal sepsis. Adjusted risk ratios were calculated using multivariable Poisson regression to assess whether rural residence in socially vulnerable communities was associated with the neonatal composite.

**Results**: Of the 605,301 births within high SVI communities, 38,350 (6.3%) were from rural California. The rural cohort were more likely to be Non‐Hispanic White (34.2% vs 21.8%), younger than 20 years of age (6.4% vs 4.3%), publicly insured (71.2% vs. 55.9%) and have an education level of high school or below (54.1% vs 41.9%). The adverse neonatal composite was significantly higher among urban communities compared to their rural counterparts (23.8% vs 21.8%, P< 0.001). After adjusting for maternal race/ethnicity, age, education level, insurance status, parity, and preexisting diabetes and hypertension, the risk of the adverse neonatal composite was lower among the rural cohort (aRR = 0.94; 95% CI: 0.92‐0.95).

**Conclusion**: Social Vulnerability measures a community's ability to face external stressors and public health emergencies. Our analysis suggests that rural communities with high SVI may have protective factors that positively impact reproductive outcomes compared to their urban counterparts. However, more research is needed to better understand disparities in health outcomes between rural and urban birthing individuals.

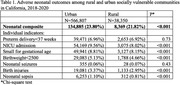

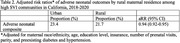

## 908 | Blood Pressure Patterns in Pregnancy using In‐Clinic and Remote Monitoring and Hypertensive Disorders of Pregnancy

Sara M. Sauer^1^; Timothy Wen^2^; Noam Finkelstein^1^; Mia Charifson^1^; Chloe Li^1^; Adesh Kadambi^1^; Shreyas Kadambi^1^; Kartik K. Venkatesh^3^; Isabel Fulcher^1^

*^1^Delfina Care Inc, Delfina Care Inc, CA; ^2^University of California, San Diego, Irvine, CA; ^3^The Ohio State University, Columbus, OH*

10:30 AM ‐ 12:30 PM

**Objective**: Despite the established association of in‐clinic blood pressure (iBP) with hypertensive disorders of pregnancy (HDP) and the increasing use of remote blood pressure monitoring (rBP), integrating both modalities of BP monitoring to inform HDP risk remains understudied. We identified BP pattern groups in early pregnancy using both iBP and rBP measures and examined their collective ability to predict HDP.

**Study Design**: Prospective cohort study of pregnant individuals without chronic hypertension who delivered at one of three clinics enrolled in a digital health platform from 2022‐2024. BP data < 20 weeks gestation was transformed into a total of 30 summary statistics characterizing centrality, spread, trends, and concordance of iBP and rBP measures. These BP summary statistics were computed separately for iBP versus rBP data and systolic versus diastolic data. The outcome was HDP. K‐means clustering identified BP pattern groups, and the association between these groups and HDP was estimated using logistic regression. Area under the curve (AUC) was used to assess predictive performance.

**Results**: Using both iBP and rBP measures from 106 assessed individuals, we identified three BP pattern groups. Individuals in group 1 (n = 35, 2.9% with HDP) were more likely to have within range BP values and different values between rBP and iBP (Figure 1). Individuals in group 2 (n = 38, 7.9% with HDP) generally had within range BP values and similar values between rBP and iBP. Individuals in group 3 (n = 33, 33.3% with HDP) were more likely to have high average BP, high BP variability, and concordance between rBP and iBP. BP pattern groups arising from both iBP and rBP data were able to accurately predict subsequent HDP diagnosis (AUC = 0.77, 95% CI: 0.66, 0.89).

**Conclusion**: K‐means clustering of BP measures based on iBP and rRP identified three distinct BP pattern groups that were predictive of HDP. Further research is needed to determine the optimal integration of rBP with iBP to inform HDP risk and care delivery.

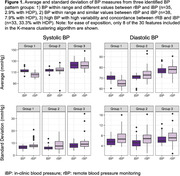

## 909 | Prevalence of Undiagnosed Placenta Accreta Spectrum After Unplanned Postpartum Hysterectomy

Sarah T. Mehl^1^; Abigail S. Zamorano^2^; Beatrice Valentini^3^; Zakaria Doughan^4^; Elias Kassir^5^; Edgar A. Hernandez‐Andrade^1^; Sean C. Blackwell^1^; Baha M. Sibai^1^; Farah H. Amro^1^

*^1^McGovern Medical School at UTHealth Houston, Houston, TX; ^2^Division of Gynecology Oncology, Department of Obstetrics, Gynecology, and Reproductive Sciences, University of Texas Health Science Center in Houston, McGovern Medical School, Houston, TX; ^3^University of Parma, University of Parma, Emilia‐Romagna; ^4^Division of Maternal‐Fetal Medicine, Department of Obstetrics, Gynecology and Reproductive Sciences, McGovern Medical School at UTHealth Houston, Houston, TX; ^5^University of Texas Health Science Center at Houston, McGovern Medical School, Houston, TX*

10:30 AM ‐ 12:30 PM

**Objective**: Antenatal diagnosis of placenta accreta spectrum (PAS) is crucial for maternal counseling and surgical planning. There is minimal data describing frequency of missed diagnosis of PAS by ultrasound, particularly in those without risk factors. Our objective was to identify rate and reason of undiagnosed PAS in unplanned postpartum hysterectomy.

**Study Design**: A retrospective cohort study of pregnancies resulting in unplanned postpartum hysterectomy from 2019–2024 and delivered within our institution's hospital system. Pregnancy outcomes were evaluated in two groups: PAS and no PAS. Maternal characteristics, pre‐existing risk factors for PAS, antenatal ultrasound findings, and outcomes including PAS severity by pathology were analyzed.

**Results**: A total of 45 unplanned postpartum hysterectomies were reviewed. All but 1 patient had prenatal care. PAS by pathology was identified in 14 (31%) patients. Table 1 describes characteristics including prior uterine surgery and placenta previa. Similar risk factors were seen between those with PAS and without. Among the 14 cases of PAS, 11 (79%) did not have placenta previa, 8 (57%) did not have prior CD, and 5 (42%) did not have anterior placenta. All 8 cases without prior CD also did not have placenta previa yet had PAS. Table 2 further describes additional risk factors among those with PAS. Accreta was the most common pathology finding, seen in 10 (71%) cases.

**Conclusion**: In this cohort, approximately 30% of unplanned postpartum hysterectomies were due to PAS. Routine risk factors such as previa and prior CD were not present in 57% of cases. Our findings suggest a need for detailed placental evaluation for all pregnant women regardless of prior uterine surgery or previa.

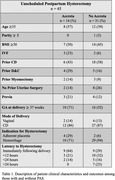

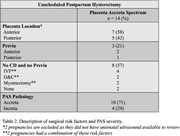

## 910 | Severe Neonatal Morbidity Among Preterm Infants After Standard Versus Accelerated Betamethasone Dosing

Frank I. Jackson; Sarah H. Abelman; Nathan A. Keller; Luis A. Bracero; Annemarie Stroustrup; Matthew J. Blitz

*Northwell, New Hyde Park, NY*

10:30 AM ‐ 12:30 PM

**Objective**: For patients expected to deliver before 34 weeks, betamethasone is usually given in two doses, 24 hours apart, to promote fetal lung maturity. If delivery is anticipated in less than 24 hours, some providers use an accelerated dosing regimen, but evidence for this practice is limited. Our objective was to determine the rate of severe neonatal morbidity (SNM) among early preterm deliveries (< 34 weeks) that occurred < 24 hours after the first dose of betamethasone was administered, and to compare the group that received one dose (standard) versus two doses (accelerated).

**Study Design**: This is a retrospective cohort study of singleton preterm deliveries from 2019‐2023 at two tertiary hospitals in New York. Patients were included if they delivered at less than 34‐0/7 weeks of gestation, received at least one dose of betamethasone prior to delivery, and delivered less than 24 hours after the first dose of betamethasone was administered. The primary outcome was SNM, a composite neonatal adverse outcome indicator which includes diagnoses and procedures indicative of severe morbidity. A logistic regression model was used to compare the rates of SNM among individuals who received one (standard) or two doses (accelerated) of betamethasone within the 24 hours prior to delivery, controlling for gestational age at birth, as well as race and ethnicity group.

**Results**: A total of 79 patients were included for analysis: 45 (56%) received standard betamethasone dosing and 34 (43%) received an accelerated dosing regimen. On adjusted analysis, there was no difference in composite SNM between the two groups (OR 1.16, 0.39‐3.42, P = 0.79). There was also no difference in rates of neonatal respiratory distress syndrome (OR 2.78, 0.81‐10.35, P = 0.11).

**Conclusion**: For infants born before 34 weeks who delivered within 24 hours of a first betamethasone dose, there was no difference in SNM between those who received an accelerated second dose and those who did not.

## 911 | Maternal and Neonatal Morbidity after Prolonged Latent Labor

Sarah Heerboth; Emma Trawick Roberts; Joy McNeal; Tracy A. Manuck

*University of North Carolina, Chapel Hill, NC*

10:30 AM ‐ 12:30 PM

**Objective**: Cesarean delivery (CD) is often recommended after 12‐18h of latent labor (LL) due to concern for futility/risk of morbidity. We sought to quantify the odds of morbidity and CD among individuals with prolonged LL.

**Study Design**: Secondary analysis of two MFMU labor studies: (1) APEX and (2) ARRIVE. We included those with singleton pregnancies ≥37w admitted for IOL who had LL ≥9h. We defined LL as <5cm dilated with rupture of membranes and exposure to oxytocin. Participants with LL 9‐12h were compared to those with LL >12h (prolonged LL). The primary outcome was maternal morbidity (ICU admission, sepsis, postpartum hemorrhage [PPH], unplanned procedure during hospitalization other than CD, thromboembolism); secondary outcomes were neonatal morbidity, PPH, intraamniotic infection (IAI), and CD. We used Liu cutpt methodology to evaluate the duration of LL that optimized sensitivity and specificity for morbidity.

**Results**: We included 2193 participants, 1000 of which had LL >12h. Of those with LL ≥12h, 12% had maternal and 16% had neonatal morbidity. Clinical characteristics are in Table 1. In the prolonged LL group, 61% delivered by CD.  Outcomes are shown in Fig 1a. Those with LL >12h had increased odds of maternal morbidity (aOR 2.4, 95% CI 1.7‐3.2), neonatal morbidity (aOR 1.4, 95% CI 1.1‐1.8), IAI (aOR 1.4, 95% CI 1.1‐1.7), PPH (aOR 2.1, 95% CI 1.5‐2.9), and CD (aOR 1.9, 95% CI 1.6‐2.2). However, the duration of LL alone was a poor predictor of maternal morbidity (AUC 0.61 at 12h). Because the alternative to prolonged LL is CD, outcomes after >12h of LL were compared to a referent group of those having CD after 9‐12h LL: there were increased odds of maternal morbidity and PPH among those who had a CS after >12h LL; there were not significantly increased odds of maternal morbidity, neonatal morbidity, PPH, or IAI among those who ultimately delivered vaginally. (Fig1b)

**Conclusion**: Prolonged LL was associated with increased overall morbidity, and CD may increase the risks associated with prolonged LL. The time point when morbidity associated with prolonged LL can be best mitigated with CD remains unknown.

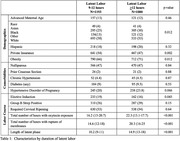

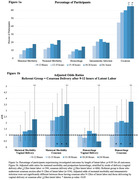

## 912 | Interval to Ultrasound‐Indicated Cerclage Placement and Delivery Timing in Patients with Prior Preterm Birth

Sarah H. Abelman; Frank I. Jackson; Nathan A. Keller; Julie Chen; Luis A. Bracero; Cara S. Wetcher; Matthew J. Blitz

*Northwell, New Hyde Park, NY*

10:30 AM ‐ 12:30 PM

**Objective**: Patients with a history of preterm birth and a cervical length less than 25mm benefit from cerclage to prevent recurrence. The urgency of cerclage placement after diagnosing a short cervix varies. This study aimed to determine if the time from diagnosis to cerclage placement affects delivery timing.

**Study Design**: This was a retrospective cohort study of all patients with a history of preterm birth who received an ultrasound‐indicated cerclage in a subsequent pregnancy from 2018‐2023 within a large health system in New York. The primary exposure was time interval from diagnosis of short cervix (< 25mm) to cerclage placement. The primary outcome was gestational age at delivery. Outcomes were compared using a linear mixed model regression analysis and were then adjusted for obesity, gestational age at diagnosis of a short cervix and cervical length at diagnosis.

**Results**: A total of 49 patients were included for analysis. The mean time interval for cerclage placement after diagnosis was 5.9 days and mean gestational age at cerclage placement was 19.0 ± 2.6 weeks. The mean cervical length on ultrasound at time of diagnosis of a short cervix was 1.8 ± 0.6 cm. The mean gestational age at delivery was 36.6 ± 3.3 weeks. Overall, for each additional day that elapsed between diagnosis of a short cervix and cerclage placement, pregnancy on average was shortened by 1.6 days (*P* = 0.01) (figure 1).

**Conclusion**: For patients with a history of preterm birth receiving an ultrasound‐indicated cerclage, earlier placement after diagnosis of short cervix significantly benefited pregnancy length, resulting in a later gestational age at delivery.

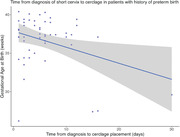

## 913 | Impact of Nurse Support on Readmission Rates Among Patients in a Postpartum Blood‐Pressure Monitoring Program

Sarah Shim^1^; Jeanne Larson Moore^1^; Sarah Fladhammer^1^; Karen Wutolia^1^; Tiffany Corlin^1^; Martina S. Burn^2^; Sarah A. Wernimont^1^; Bethany Sabol^1^

*^1^University of Minnesota, Minneapolis, MN; ^2^University of Minnesota, Minneapolis,, MN*

10:30 AM ‐ 12:30 PM

**Objective**: To assess the impact of nursing support on postpartum readmission rates for patients participating in our home observation of postpartum elevated blood pressure (HOPE‐BP) monitoring program.

**Study Design**: HOPE‐BP is a 6‐week postpartum remote monitoring program for patients with hypertensive disorders of pregnancy (HDP) discharging after birth from our institution. It was implemented in 2023 and uses Epic Care Companion and a registered nurse (RN) driven protocol, with physician oversight, for antihypertensive medication initiation/titration. Blood pressures (BPs) are monitored by the RN team Monday‐Friday from 8AM‐5PM. Outside of these hours, patients are directed to call the on‐call provider for severe hypertension or escalating symptoms; however, active surveillance or response to increasing BPs does not occur during this unsupported time. We hypothesized that readmission rates would be higher during times when RN support was unavailable.

**Results**: We evaluated postpartum readmissions for all patients participating in HOPE‐BP from the start of the program in November 2023 through June 2024. Among those enrolled in HOPE‐BP (N = 280; 75% of eligible patients), 2.8% were readmitted (N = 8). 7 of the 8 were admitted for a new diagnosis of preeclampsia with severe features based on blood pressure criteria (chronic hypertension N = 3, gestational hypertension N = 3, and preeclampsia without severe features N = 1). We observed, that as hypothesized, 75% (N = 6) of these readmissions occurred at times without RN coverage. Figure 1 demonstrates the impact of HOPE‐BP on the reduction of postpartum readmissions during RN‐supported hours.

**Conclusion**: Our data uniquely demonstrates the impact RN support has on reducing postpartum readmissions. Supported BP surveillance and management is critical to the program's success in the prompt recognition and treatment of worsening hypertension and reducing preventable readmissions.

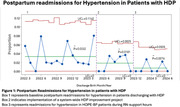

## 914 | Impact of Maternal Anxiety and Depression on Perinatal Outcomes

Sarah K. Dzubay; Bharti Garg; Aaron B. Caughey

*Oregon Health & Science University, Portland, OR*

10:30 AM ‐ 12:30 PM

**Objective**: To investigate whether anxiety, depression, or both anxiety and depression have differential impacts on perinatal outcomes relative to those without anxiety or depression.

**Study Design**: We performed a retrospective cohort study among singleton, non‐anomalous births delivered at 23‐42 weeks in California between 2008 and 2020. We compared perinatal outcomes between pregnant individuals without depression or anxiety to those with anxiety, depression, or both anxiety and depression. Births were excluded if the mother had bipolar disorder. We performed a multivariable Poisson regression, controlling for race/ethnicity, age, pre‐pregnancy BMI, parity, insurance type, education level, and smoking during pregnancy.

**Results**: Of the 5,048,487 births meeting inclusion criteria, 97.12% of mothers had no anxiety or depression, 1.17% had depression, 1.25% had anxiety, and 0.47% had anxiety and depression (Table 1). Individuals with anxiety had higher risk of gestational hypertension (aIRR = 1.62; 95% CI: 1.57‐1.67), preeclampsia (aIRR = 1.48 (1.43‐1.53)), and SMM (aIRR = 2.00 (1.90‐2.11)); however, individuals with depression had higher risk of gestational diabetes (aIRR = 1.20 (1.17‐1.23) and postpartum hemorrhage (aIRR = 1.77 (1.71‐1.83)) (Table 2). With regards to neonatal outcomes, individuals with both depression and anxiety had higher risk of preterm birth < 37 weeks (aIRR = 1.27 (1.23‐1.33)), NICU admission (aIRR = 1.11 (1.07‐1.15)), lower Apgar scores (aIRR = 2.34 (2.15‐2.54)), respiratory distress syndrome (aIRR = 1.65 (1.49‐1.81)), hypoglycemia (aIRR = 1.96 (1.83‐2.09) and low birth weight (aIRR = 1.15 (1.06‐1.24)).

**Conclusion**: Individuals with anxiety, depression, or both had higher risk of maternal and neonatal outcomes relative to those without a mood disorder. Interestingly, individuals with depression or anxiety had higher risk of maternal outcomes while individuals with both depression and anxiety had higher risk for neonatal outcomes. Prenatal screening and treatment for anxiety and depression may be beneficial in protecting against adverse outcomes.

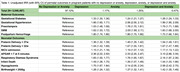

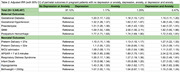

## 915 | Fetal Doppler Progression and Outcomes in Pregnancies with Late Onset Fetal Growth Restriction and Preeclampsia

Scott Infusino; Jeanette Larson; Natalia Gontarczyk Uczkowski; Amy Godecker; Jacquelyn H. Adams; Jesus Iruretagoyena

*University of Wisconsin Hospitals and Clinics, Madison, WI*

10:30 AM ‐ 12:30 PM

**Objective**: To determine if fetal Doppler abnormalities, as well as pregnancy outcomes, in late onset fetal growth restriction (FGR) are impacted by preeclampsia.

**Study Design**: This was a retrospective cohort study from 2016 to 2021 at a single academic center evaluating singleton, nonanomalous pregnancies with a diagnosis of late onset FGR (≥32 weeks) who underwent serial fetal Doppler evaluation. Doppler evaluation included umbilical artery (UA) pulsatility index (PI) and characterization of UA end diastolic flow (forward, absent, or reversed). Pregnancy outcomes were also collected.

**Results**: Five hundred forty‐five pregnancies were diagnosed with late onset FGR and met inclusion criteria. Ten percent of pregnancies (n = 56) developed preeclampsia. When compared to patients with only a diagnosis of FGR, those with both FGR and preeclampsia were more likely to have an earlier diagnosis of FGR (34.3 vs 35.2 weeks, p = 0.003), deliver at an earlier gestational age (36.2 vs 38.2 weeks p< 0.001), and have a shorter interval from diagnosis of FGR to delivery (13.5 vs 21 days, p< 0.001). Pregnancies with both FGR and preeclampsia were more likely to have an elevated UA PI at the time of delivery (p< 0.001). When an elevated UA PI was diagnosed, the interval from diagnosis to delivery was significantly shorter in the preeclamptic group (6.8 vs 22.3 days, p< 0.001).

**Conclusion**: Pregnancies complicated by both late onset fetal growth restriction and preeclampsia were more likely to have an elevated UA PI at the time of delivery, an earlier gestational age at delivery, and a shorter interval to delivery. This information can help providers appropriately manage these complex pregnancies and adequately counsel patients.

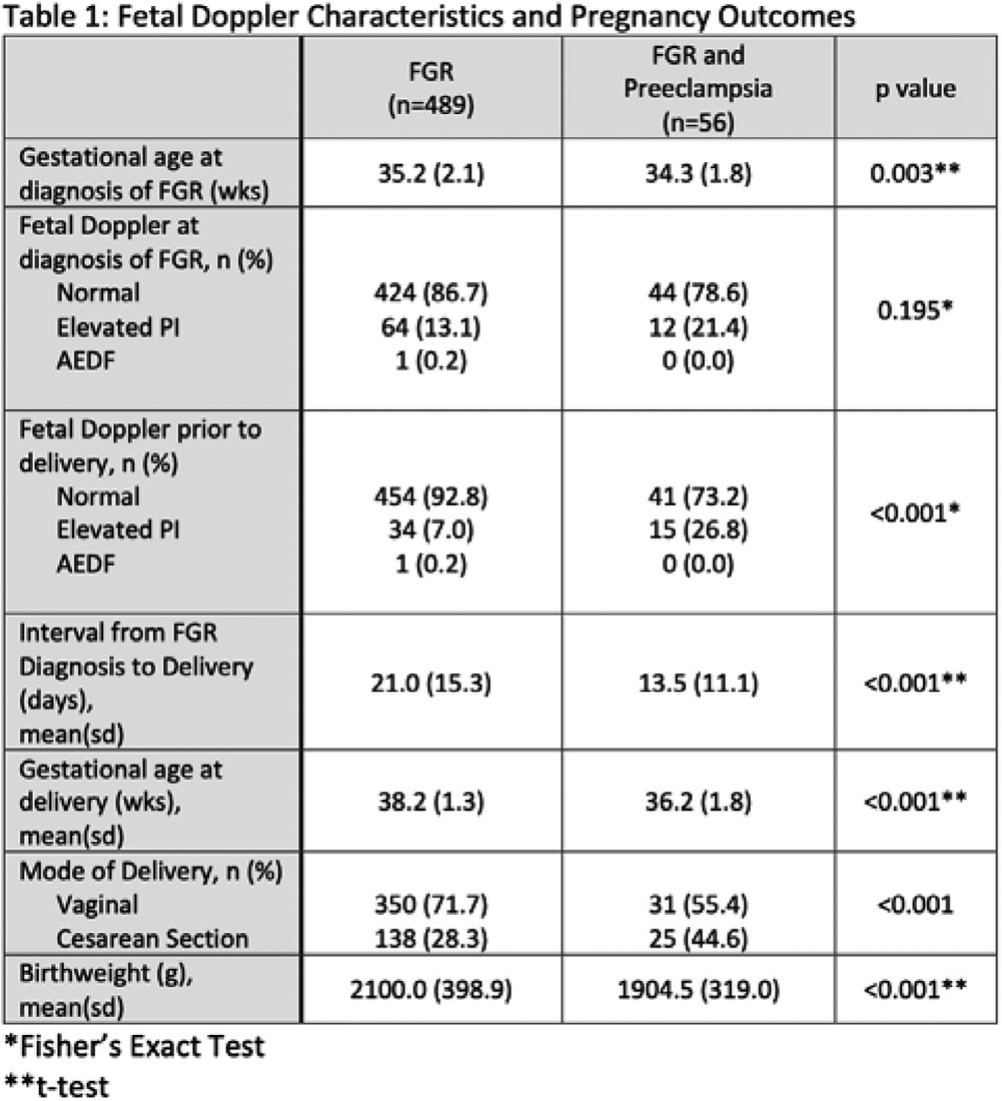

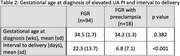

## 916 | Middle Cerebral Artery Pulsatility Index and Cerebroplacental Ratio in Late Onset Growth Restriction and Preeclampsia

Scott Infusino; Jeanette Larson; Natalia Gontarczyk Uczkowski; Amy Godecker; Jacquelyn H. Adams; Jesus Iruretagoyena

*University of Wisconsin Hospitals and Clinics, Madison, WI*

10:30 AM ‐ 12:30 PM

**Objective**: To determine if the presence of preeclampsia impacts the rate of abnormal middle cerebral artery (MCA) pulsatility index (PI) and cerebroplacental ratio (CPR) in pregnancies complicated by late onset fetal growth restriction (FGR).

**Study Design**: A retrospective cohort study was performed from 2016 to 2021 at a single academic medical center of singleton, nonanomalous pregnancies with a diagnosis of late onset FGR (>32 weeks) who underwent weekly fetal Doppler evaluation. MCA PI and CPR were considered abnormal if they were noted to be less than the 5^th^ percentile for gestational age. Gestational age at delivery was recorded for all patients.

**Results**: Five hundred forty‐five pregnancies were diagnosed with late onset FGR and met inclusion criteria. Fifty‐six (10%) patients were also diagnosed with preeclampsia (Table 1). When compared to pregnancies with only a diagnosis of FGR, those with both FGR and preeclampsia were more likely to have an abnormal MCA PI (31.7% vs 11.5%, p = 0.001) or CPR (39.0% vs 16.0%, p< 0.001). Patients with both preeclampsia and FGR had an earlier diagnosis of abnormal CPR (34.4 vs 35.5 weeks, p = 0.028) and delivered at an earlier gestational age (35.7 vs 37.6 weeks p< 0.001). Gestational age at diagnosis of abnormal MCA PI did not significantly differ between groups.

**Conclusion**: Pregnancies complicated by both late onset FGR and preeclampsia were more likely to have abnormal MCA PI and CPR, than pregnancies only complicated by FGR. An abnormal cerebroplacental ratio was associated with the greatest difference in findings between groups. Future work should evaluate neonatal outcomes related to these Doppler characteristics in the presence of fetal growth restriction and preeclampsia.

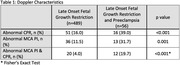

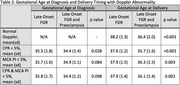

## 917 | Non‐Invasive Prenatal Detection of Copy‐Number Variations Based on Maternal Cfdna Fragmentomics

Shahar Shohat Koren^1^; Sapir Saar^1^; Dolev Rahat^2^; Ravit Mesika^2^; Reut Tomashov Matar^3^; Lina Basel‐Salmon^3^; Noa Liscovitch‐Brauer^4^; Noam Shomron^2^

*^1^Identifai Genetics, Identifai Genetics, Tel Aviv; ^2^Identifai Genetics, Tel Aviv, Israel; ^3^Raphael Recanati Genetic Institute, Rabin Medical Center, Beilinson Hospital, Tel Aviv; ^4^Identifai Genetics, Tel Aviv*

10:30 AM ‐ 12:30 PM

**Objective**: Cell‐free DNA (cfDNA) based prenatal screening determines the risk of genetic abnormalities using cfDNA of fetal origin circulating in maternal plasma. Current methods detect chromosomal scale aberrations or very large (>1 Mb) copy number variants (CNVs), missing smaller ones which account for up to 15% of developmental disorders in children. To identify shorter CNVs, we developed a whole genome sequencing (WGS) approach that utilizes both read coverage and fragmentomics features.

**Study Design**: Our cohort includes 10 women with singleton pregnancies for whom WGS was performed on cfDNA and maternal gDNA. Seven out of the ten pregnancies were sampled in the 1st trimester, with a median fetal fraction (FF) of 7.7%. CNVs causing genetic disorders are typically de novo and dominant, or, in the case of a recessive condition, inherited alongside another pathogenic variant from the other parent, resulting in compound heterozygosity. Therefore we focused on the detection of *de novo* CNVs >0.1 Mb and smaller CNVs that could cause compound heterozygosity. Paternal CNVs are detected in a similar manner to de novo variants and so were also included in the analysis.

**Results**: We implemented an algorithm which calculates a Z‐score for the difference in cfDNA read lengths across the genome. Two deletions, of size 690 KB and 209 KB were detected in two families. Validation with fetal WGS confirmed the deletions as true positives, with no other relevant deletions found. In a third family, the mother is a known carrier of a pathogenic ∼5 KB deletion in the CERS3 gene, and the father carries a pathogenic SNV in the same gene. Our algorithm successfully determined that the fetus did not inherit the maternal CNV (Figure 1).

**Conclusion**: We present a method to identify CNVs in a cfDNA‐based prenatal screening and show that combining read length with coverage data allows reliable CNV detection. Importantly, we demonstrate our method on mostly 1st trimester pregnancies with a low FF, representing a clinically relevant scenario, thus enhancing the potential scope and resolution of noninvasive prenatal screening.

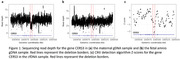

## 918 | Can Blood Pressure Monitoring Reduce Racial Disparities in Healthcare Utilization and Morbidity Among Postpartum Patients?

Shana D. Talbot^1^; Ntami P. Echeng^1^; Scott Machado^2^; Tracy L. Jackson^2^; Nicole Konecke^2^; William Hunt^2^; Maria Mejia Castillo^2^; Danielle Simmons^2^; Heather A Smith^1^; Nina K. Ayala^1^; William A. Grobman^3^; Adam K. Lewkowitz^2^; Methodius G. Tuuli^1^

*^1^Women & Infants Hospital of Rhode Island / Alpert Medical School of Brown University, Providence, RI; ^2^Women & Infants Hospital of Rhode Island / Alpert Medical School of Brown University., Providence, RI; ^3^The Ohio State University, Columbus, OH*

10:30 AM ‐ 12:30 PM

**Objective**: Remote self‐measured blood pressure (SMBP) monitoring programs have been shown to reduce racial inequity in postpartum blood pressure ascertainment. However, their effect on disparities in healthcare utilization and severe maternal morbidity (SMM) is less clear. We aimed to compare outcomes between participants in our remote SMBP program who self‐identified as Black versus non‐Hispanic White.

**Study Design**: Postpartum individuals with hypertension (HTN) at our tertiary hospital are offered enrollment in our remote SMBP program. For this analysis, participants who did not self‐identify as Black or non‐Hispanic White were excluded. The primary outcome was a composite of HTN‐related postpartum readmission or ED visit within 30 days of delivery. Secondary outcomes were individual components of the primary composite, HTN‐related SMM, and anti‐hypertension medication initiation or titration. A log‐link binomial generalized linear model was used to estimate relative risks (aRR) adjusting for potential confounders.

**Results**: Among 2003 participants in the SMBP program from 2022 ‐ 2024, 1363 met inclusion criteria. Of these, 988 (49.3%) self‐identified as non‐Hispanic White and 375 (18.7%) as Black. Compared to non‐Hispanic White participants, Black participants were younger, and more likely to have public insurance and gestational HTN (**Table 1**). After adjusting for these factors, there was no significant difference in the composite of HTN‐related postpartum readmission or ED visit between Black and non‐Hispanic White participants (19.2% vs 16.7%; aRR 1.29, 95% CI 0.98, 1.69). Risk of HTN‐related readmission alone was significantly higher among Black participants (12.2% vs 9.9%; aRR 1.48, 95% CI 1.03, 2.11). There was no significant difference in HTN‐related SMM (12.5% vs 11.7%; aRR 1.00, 95% CI 0.72, 1.42). There was also no difference in anti‐hypertensive medication initiation or titration (**Table 2**).

**Conclusion**: Our remote SMBP program for postpartum patients with HTN eliminated racial disparities in overall HTN‐related healthcare utilization and HTN‐related SMM.

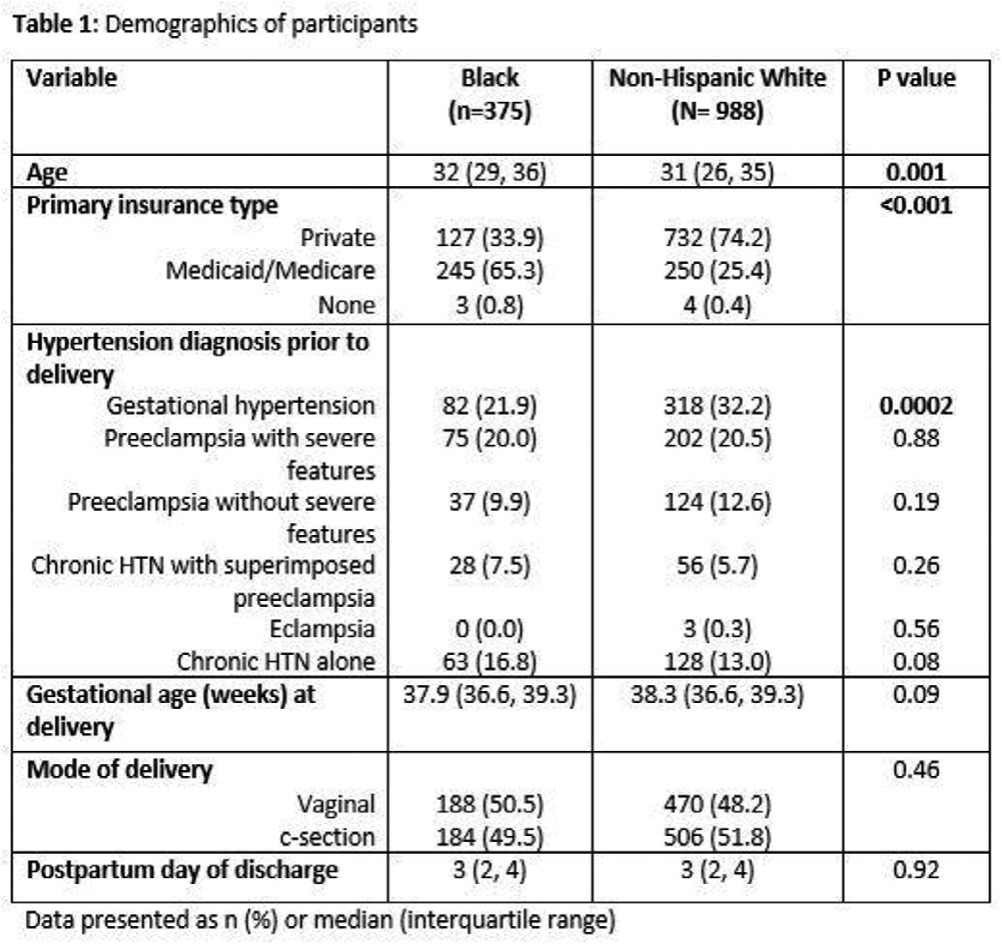

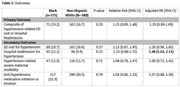

## 919 | The Effect of Pre‐Pregnancy and Delivery Bmi on Tolac Success in a High‐Risk Cohort

Shannon R.O Moran; James A. Shelton; Dawei David Wang

*University at Buffalo, Buffalo, NY*

10:30 AM ‐ 12:30 PM

**Objective**: Rates of trial of labor after cesarean section (TOLAC) and obesity have increased in today's population. Vaginal birth after cesarean section (VBAC) has been shown to reduce maternal morbidity. Published predictive models for VBAC success take into account both body mass index (BMI) and chronic hypertension. However, no previous studies have specifically analyzed the effect of BMI on TOLAC success in those with any form of hypertension. Therefore, we sought to investigate whether pre‐pregnancy BMI or delivery BMI has a greater effect on TOLAC success rate in this high‐risk cohort.

**Study Design**: This was a retrospective cohort study of national birth certificate data from 2022. We included all singleton, vertex livebirths with no fetal anomalies who had 1 prior cesarean delivery and a diagnosis of either chronic hypertension or a hypertensive disorder of pregnancy who attempted TOLAC. Those with prior vaginal deliveries were excluded. Patients were divided into 6 groups by pre‐pregnancy and delivery BMI. Two logistic regression models, one for pre‐pregnancy and one for delivery BMI, were created. These were compared using Akaike Information Criterion (AIC) and receiver operating characteristic (ROC) curves. Confounding variables included were maternal age and race, maternal weight gain, chronic and gestational diabetes, gestational age, birthweight, and induction of labor.

**Results**: 5,144 patients were included. 2,382 had a repeat cesarean section. 2,762 had a VBAC. For both pre‐pregnancy and delivery BMI, the rate of VBAC decreased as obesity class increased. In the AIC analysis, pre‐pregnancy BMI and delivery BMI produced almost identical results (6685 versus 6670, respectively). Furthermore, in the ROC curve, again pre‐pregnancy BMI and delivery BMI produced almost identical results (0.631 versus 0.634, respectively).

**Conclusion**: Pre‐pregnancy BMI and delivery BMI have similar effects on TOLAC success in those with hypertension of any form, with decreased rates of VBAC seen with increasing BMI. Both should be used to counsel patients on TOLAC in this high‐risk cohort throughout the antepartum period.

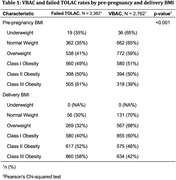

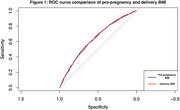

## 920 | The Impact of Pre‐Pregnancy Anxiety Or Depression and Adverse Neonatal Outcomes

Shannon E. Beermann^1^; Nandini Raghuraman^2^; Bridget C. Huysman^1^; Jeannie C. Kelly^2^; Antonina I. Frolova^3^; Candice Woolfolk^4^

*^1^Washington University in St. Louis, St. Louis, MO; ^2^Washington University School of Medicine in St. Louis, St. Louis, MO; ^3^Washington University School of Medicine, St. Louis, MO; ^4^Washington University School of Medicine in Saint Louis, St. Louis, MO*

10:30 AM ‐ 12:30 PM

**Objective**: Nearly 30% of females are affected by mental illness, particularly anxiety and depression. Prior studies investigating the relationship between prenatal anxiety and depression and adverse neonatal outcomes have inconsistent findings. We sought to examine the relationship between pre‐pregnancy anxiety or depression or a dual diagnosis and adverse neonatal outcomes.

**Study Design**: This is a secondary analysis of a prospective cohort study of singleton pregnancies at single tertiary medical center from 2017 to 2020. The primary outcomes were preterm birth and composite neonatal morbidity (Table 1). The secondary outcomes were spontaneous preterm birth, small for gestational age (SGA), and 5‐minute APGAR< 7. Secondary analyses compared dual diagnosis of anxiety and depression to patients with no diagnosis. Multivariable logistic regression was used to estimate adjusted odds ratios (aOR) and 95% confidence intervals (CI) after adjusting for potential confounders.

**Results**: Of 1220 patients with live births, 253 (20.7%) had pre‐pregnancy anxiety or depression and 967 (79.3%) did not. After adjusting for maternal race and advanced maternal age, patients with pre‐pregnancy anxiety or depression were significantly more likely to have preterm birth [19.8% vs 10.9%, aOR 2.07 95% CI 1.42, 3.02] and composite neonatal morbidity [35.2% vs 23.2%, aOR 1.80 95% CI 1.31, 2.41]. Spontaneous preterm birth, SGA, and 5‐minute APGAR< 7 were significantly more likely among patients with anxiety or depression. Secondary analyses comparing dual diagnosis to no diagnosis showed an even greater likelihood of preterm birth, 5‐minute APGAR< 7, and composite neonatal morbidity compared to patients without anxiety or depression (Table 2).

**Conclusion**: Patients with pre‐pregnancy anxiety or depression are at higher risk of adverse neonatal outcomes. This risk is further increased with concurrent diagnoses. Further research is needed to determine if optimization of pre‐pregnancy anxiety and depression can improve neonatal outcomes.

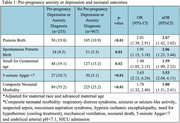

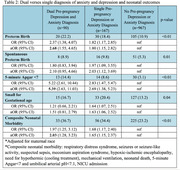

## 921 | Impact of Maternal Age on Likelihood of Cesarean Delivery

Shaun R. Wesley; Sarah Crimmins

*University of Rochester, Rochester, NY*

10:30 AM ‐ 12:30 PM

**Objective**: To evaluate the impact of maternal age on the likelihood of undergoing cesarean delivery (CD) while adjusting for clinical and demographic confounders, focusing on clinically meaningful odds ratios.

**Study Design**: This retrospective cohort study utilized the United States Vital Statistics Natality Birth Data from 2018 to 2022, comprising 18,524,899 births. Multivariable logistic regression was performed to analyze the association between maternal age and CD, adjusting for body mass index (BMI), pre‐pregnancy hypertension, pre‐pregnancy diabetes, previous cesarean section, parity, and chorioamnionitis. The analysis highlights odds ratios greater than 2.0 or less than 0.5, indicating clinical significance in delivery outcomes.

**Results**: The final analysis included 18,062,643 cases after excluding missing data. Compared to individuals under 20, cesarean delivery rates increased with age. Individuals aged 35‐39 had significantly increased odds of CD (aOR: 2.65, 95% CI: 2.63, 2.66), and those aged 40 and older had even higher odds (aOR: 3.70, 95% CI: 3.67, 3.73). Previous cesarean delivery strongly predicted future CD (aOR: 35.74, 95% CI: 35.60, 35.88). Conversely, being multiparous significantly decreased odds of CD (aOR: 0.32, 95% CI: 0.32, 0.33). Notably, individuals with Class III obesity (BMI ≥ 40.0) had more than double the odds of CD with all other covariates held constant (aOR: 3.36, 95% CI: 3.33, 3.39).

**Conclusion**: Maternal age is a significant predictor of CD, especially for those over 35, showing a clinically significant increase in CD likelihood. Our data emphasizes factors representing meaningful clinical thresholds, aiding healthcare providers in decision‐making and patient counseling. These findings enhance risk assessment strategies and inform future research on targeted interventions for higher risk populations.

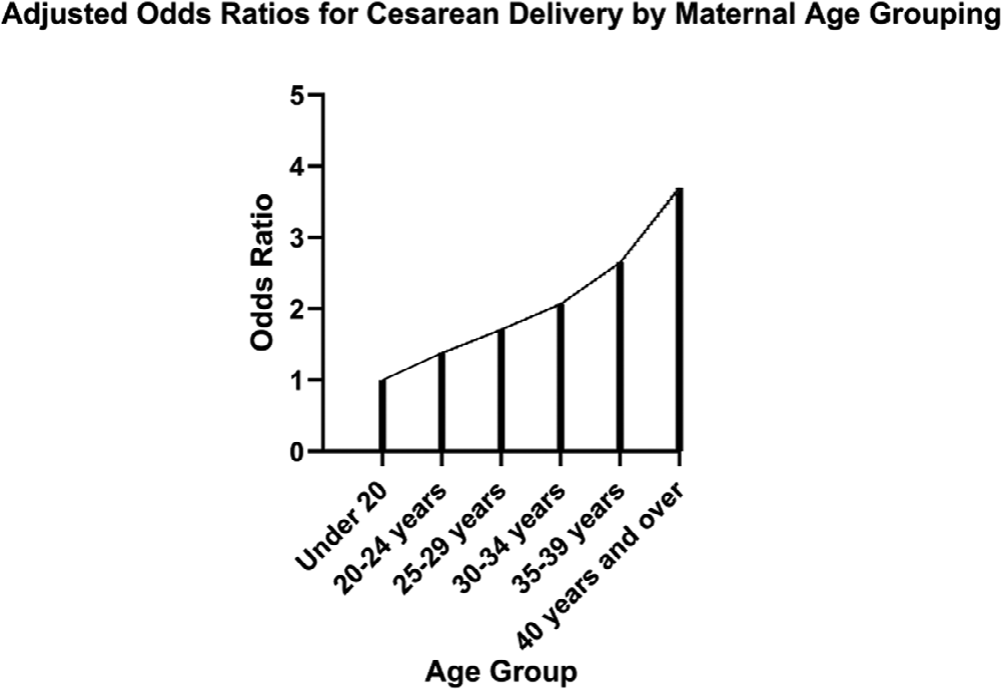

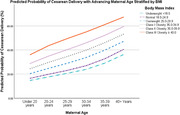

## 922 | Association of Ultrasound Determined Hysterotomy Thickness and Intraoperative Scar Thinning/Dehiscence After Fetal Spina Bifida Closure

Shelly Soni^1^; Federica Bellussi^2^; Suzanne Debari^2^; Nahla Khalek^1^; Juliana S. Gebb^3^; Christina Paidas Teefey^1^; N. Scott Adzick^4^; Julie S. Moldenhauer^1^

*^1^Richard D. Wood Jr. Center for Fetal Diagnosis and Treatment at CHOP, Philadelphia, PA; ^2^The Children's Hospital of Phildelphia, Philadelphia, PA; ^3^Richard D. Wood, Jr Center for Fetal Diagnosis and Treatment, Children's Hospital of Philadelphia, Philadelphia, PA; ^4^The Children's Hosiptal of Philadelphia, Philadelphia, PA*

10:30 AM ‐ 12:30 PM

**Objective**: To study the association between ultrasound determined myometrial thickness and intraoperative findings of scar thinning or dehiscence at the time of cesarean delivery in patients undergoing open maternal fetal surgery (OMFS) for fetal spina bifida (fSB) closure.

**Study Design**: A retrospective review of all cases of OMFS for fSB closure from 2016‐2022. Hysterotomy scar thickness was measured on archived images at 30 weeks, 32 weeks, 34 weeks and 36 weeks. Three measurements of myometrial thickness were taken for every timepoint in sagittal plane. The group with intact hysterotomy was compared to those with scar thinning/ dehiscence.

**Results**: 111 patients met the inclusion criteria. The average hysterotomy scar thickness was 0.83 ± 0.18 cm, 0.78 ±0.19 cm, 0.72 ± 0.18 cm and 0.71 ± 0.22 cm at 30, 32, 34 and 36 weeks, respectively. 54.1% patients had at least some degree of scar thinning and 6.3% patients had some degree of dehiscence including focal. Scar thickness was significantly lower at 34 weeks (p = 0.02) and 36 weeks (p< 0.0001) in patients that had thinning/dehiscence. δ scar thickness between 30 and 36 weeks was significantly more in patients with scar thinning/dehiscence (p = 0.0009). AUC was 0.63 with a p‐value of 0.02 for hysterotomy thickness at 34 weeks predicting absence of thinning. AUC was 0.82 with a p‐value of < 0.0001 for hysterotomy thickness at 36 weeks predicting absence of thinning. A thickness of ≥ 0.71 cm was 68.6% sensitive and 84.9% specific in predicting absence of thinning. AUC was 0.76 with a p‐value of 0.0003 for ð hysterotomy thickness between 30‐36 weeks predicting presence of thinning/dehiscence. A change of ≥ 0.13 cm was 69.7% sensitive and 73.5% specific in predicting presence of thinning/dehiscence.

**Conclusion**: In patients undergoing OMFS for fSB repair, gradual thinning of hysterotomy scar was observed with significantly less scar thickness at 34 and 36 weeks in patients diagnosed with scar thinning/ dehiscence. δ scar thickness between 30 and 36 weeks can be used to predict presence of thinning/dehiscence.

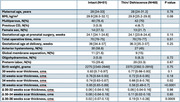

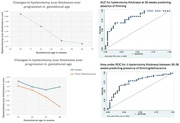

## 924 | The Accuracy of the sFlt‐1/PlGF Ratio Assay in Predicting Time‐to‐Delivery in Patients with Preeclampsia

Siobhan Wilson^1^; Elaine Carson^2^; Lei Fu^3^; Nir Melamed^4^

*^1^University of Toronto, Toronto, ON; ^2^Department of Maternal Fetal Medicine, Leeds Hospital Trust, Leeds, England; ^3^Sunnybrook Health Sciences Centre, Toronto, ON; ^4^Division of Maternal Fetal Medicine, Department of Obstetrics and Gynecology, Sunnybrook Health Sciences Centre, Toronto, ON*

10:30 AM ‐ 12:30 PM

**Objective**: Preeclampsia (PET) is a leading cause of maternal and fetal morbidity and mortality, but the clinical course of PET is often difficult to predict. This can make management decisions such as the administration of antenatal corticosteroids (ACS) challenging, given that the optimal ACS timing is 1‐7 days before birth. Abnormal levels of the angiogenic proteins soluble fms‐like tyrosine kinase (sFLT‐1) and placental growth factor (PlGF) have been shown to improve the diagnostic accuracy for PET. However, data on their accuracy in predicting time to delivery are limited. The objective of the current study was to evaluate the accuracy of these proteins in predicting the time‐to‐delivery in patients with PET.

**Study Design**: A retrospective cohort study of patients with a singleton pregnancy evaluated in a single center for suspected preeclampsia using the Roche Elecsys sFlt1/pPlGF Ratio Assay between 2020‐2023. Modified Poisson regression was used to estimate the associations of the sFlt1/PlGF ratio with the risk of delivery within 3 and 7 days. Time‐to‐event analysis was used to describe the cumulative risk of delivery by the sFlt1/PlGF ratio result.

**Results**: Of the 509 patients who met the study criteria, 40.6% had a final diagnosis of PET. Compared with a low‐risk result (≤ 38), a high‐risk ratio (> 85) was associated with an increased risk of preterm birth < 32 weeks (RR 16.4 [95%‐CI 6.2‐42.6]) and delivery within 3 or 7 days (RR 3.5 [2.3‐5.2] and 3.6 [2.6‐4.9], respectively). A moderate‐risk (38‐85) and high‐risk (> 85) ratios were associated with a shorter time‐to‐delivery compared to a low‐risk ratio (p< .001) (Figure 1). The positive predictive value of a high‐risk ratio and the negative predictive value of a low‐risk ratio for delivery within 7 days were 63% and 99%, respectively.

**Conclusion**: The sFLT‐1/PlGF ratio is a useful prognostic tool to predict the time to delivery in patients with PET, and can therefore guide management decisions such as the administration of antenatal corticosteroids.

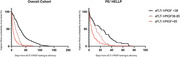

## 925 | A New Reference for Umbilical Artery Doppler in the Second and Third Trimesters

Sophia Rahimi^1^; John C. Kingdom^2^; Arietta Vayenas^3^; Vasilica Stratulat^4^; Nir Melamed^5^

*^1^Temerty Faculty of Medicine, University of Toronto, Toronto, OR; ^2^Dept OBGYN Mount Sinai Hospital University of Toronto, Toronto, ON; ^3^Sunnybrook Health Sciences Centre, Toronto, OR; ^4^Mount Sinai Hospital, Toronto, ON; ^5^Division of Maternal Fetal Medicine, Department of Obstetrics and Gynecology, Sunnybrook Health Sciences Centre, Toronto, ON*

10:30 AM ‐ 12:30 PM

**Objective**: Umbilical artery (UA) Doppler is a key component in the diagnosis of early‐onset fetal growth restriction (FGR). However, the interpretation of UA Doppler is complicated by the considerable variability of available UA Doppler references, which can have a profound impact on the proportion of small fetuses diagnosed with FGR. Our aim was to explore the methodological factors contributing to the heterogeneity of existing references and develop a UA Doppler pulsatility index (UA‐PI) reference using an approach that addresses some of the limitations of prior studies.

**Study Design**: This was a retrospective longitudinal study of individuals with an uncomplicated singleton pregnancy who underwent assessment of UA Doppler at a single academic center (2012‐2022) where UA Doppler is measured routinely in all ultrasound exams. We explored the effect of estimated fetal weight (EFW) percentile threshold (>10^th^, >25^th^, or >50^th^ centile), parity, and statistical modeling approach (LMS, quantile regression, and quantile sheets) on the reference values. Based on these findings, a final UA‐PI chart was developed and compared to existing charts.

**Results**: A total of 25,069 UA‐PI measurements from 12,394 patients were analyzed. The study population's EFW percentile threshold had a considerable impact on the UA‐PI reference (**Figure 1**), whereas the effect of parity was minimal. LMS and quantile regression methods yielded similar UA‐PI reference charts, unlike the quantile sheets method. Therefore, our final UA‐PI reference was based on observations from fetuses with EFW >50^th^ percentile using the LMS method. The differences between the new chart and previously published charts in the UA‐PI 95^th^ percentile are illustrated in **Figure 2**.

**Conclusion**: To our knowledge, this study is the first to explore the impact of methodological factors on a UA‐PI reference chart. The newly developed chart addresses several limitations of previous studies. As such, it may reflect more accurately the distribution of UA‐PI in uncomplicated singleton pregnancies and improve the accuracy of FGR diagnosis.

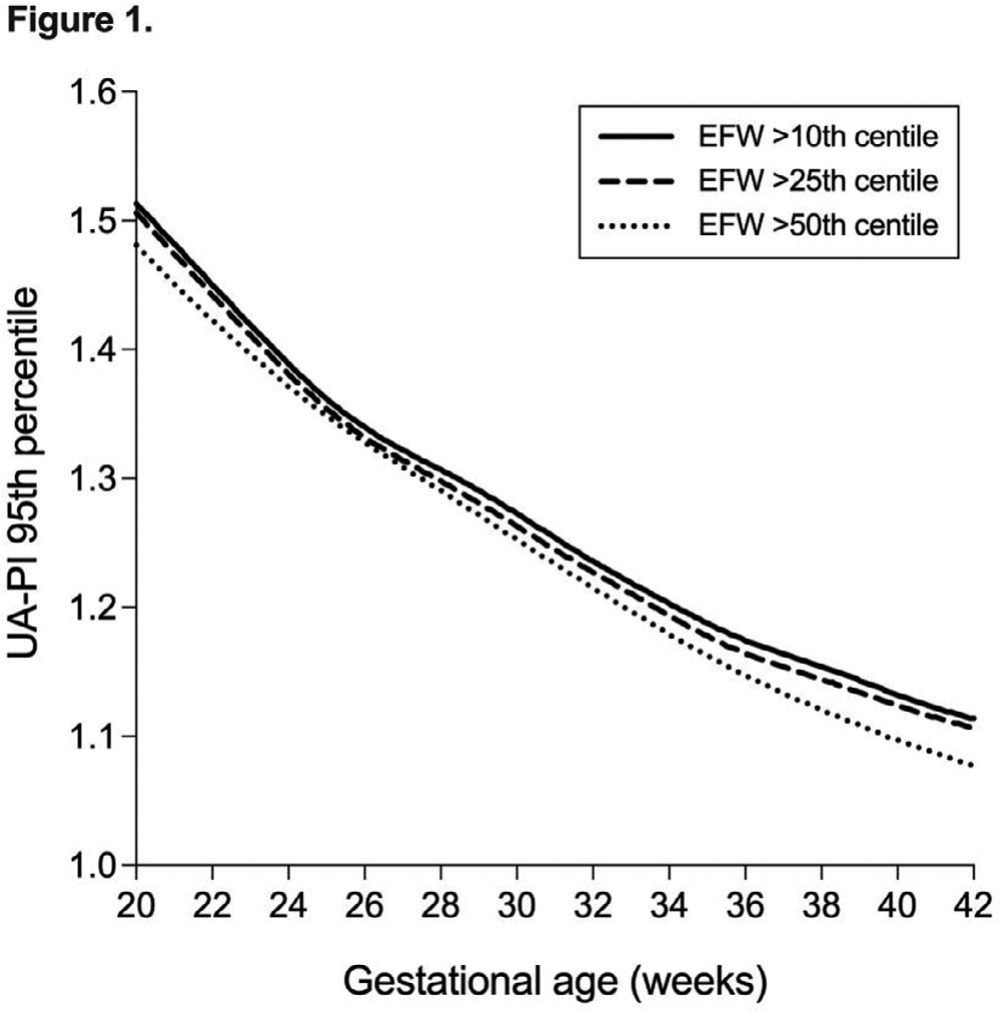

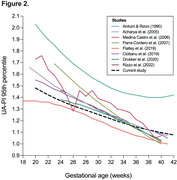

## 926 | a Fetal Blood‐Brain‐Barrier Microphysiological System to Study the Effect of In‐Utero Toxicant Exposure

Sourabh Sharma^1^; Lauren Richardson^2^; Ramkumar Menon^2^

*^1^University of Texas Medical Branch Galveston, Galveston, TX; ^2^University of Texas Medical Branch, Galveston, TX*

10:30 AM ‐ 12:30 PM

**Objective**: Glutamate dysregulation resuling in neuronal damage is associated with the development of many neurological disorders. The lack of physiologically relevant in‐vitro models has limited our mechanistic understanding. This study evaluated the effect of environmental pollutant Polybrominated Diphenyl Ethers (PBDE), using a microphysiologic (MPS) model of human fetal blood‐brain‐barrier (FB).

**Study Design**: The FB‐OOC is composed of 3‐cell culture chambers, connected by microchannels, containing 1)brain microvessel endothelial cells, 2)human vascular pericytes, and 3)a triculture of neurons, astrocytes, and microglia in a 5:2:1 ratio, respectively. To assess the effect of toxicants on glutamate dysregulation and neuroinflammation, endothelial cells were exposed to PBDE(150ng/ml). To mimic the passage of PBDE through the placenta, endothelial cells were exposed to conditioned PDBE media from a placenta‐OOC (1:1). In parallel, triculture cells were directly treated in a 96‐well plate. Dextran propagation over 72 hours and zonula occludens‐1 expression confirmed FB barrier function. Cell morphology (microscopy), cell cytotoxicity, and cytokines were measured. Statistical significance was determined by unpaired t‐tests (N = 5; p< 0.05).  

**Results**: Control FB‐OOC were characterized by 1)viable cell cultures expressing standard cell morphologies and cell‐specific markers, 2)barrier formation confirmed by lack of dextran propagation over 72 hours, and 3) baseline pro‐inflammatory cytokines. On‐chip PBDE and placenta‐derived metabolites of PBDE treatment in the endothelial chamber induced cell cytotoxicity and upregulation of glutamate in the triculture but did not induce neuroinflammation. Conversely, 2D triculture experiments showed direct PBDE treatment‐induced neuroinflammation compared to PBDE placenta‐derived metabolites or controls.  

**Conclusion**: This study established a model of FB that assessed the role of glutamate dysregulation independent of neuroinflammation. Advanced MPS models are needed to evaluate the impact of various exposures at the FB, as 2D neuronal cultures cannot model intercellular interactions.

## 927 | Prenatal Anemia: Examining Trends and Racial Disparities Before and After Standardized Clinical Interventions

Supraja Rachuri^1^; Edward Lievanos^1^; Aida Shirazi^1^; Deanna Fink^2^; Zahra Samiezade‐Yazd^3^; Lauren Gong Barres^1^

*^1^Kaiser Permanente San Francisco, Kaiser Permanente San Francisco, CA; ^2^Kaiser Permanente, Oakland, CA; ^3^Kaiser Permanente Division of Research, Kaiser Permanente San Francisco, CA*

10:30 AM ‐ 12:30 PM

**Objective**: Anemia is a common complication of pregnancy that disproportionately affects Black women and is linked to adverse maternal outcomes including cesarean delivery, postpartum hemorrhage, intraamniotic infections and need for blood transfusions. The Controlling Anemia in Pregnancy (CAP) project introduced a clinical algorithm that standardized diagnosis and treatment of anemia across a large healthcare institution. We sought to assess the impact of this algorithm on the incidence of prenatal anemia and identify racial disparities.

**Study Design**: We conducted a retrospective cohort study of adult pregnant patients with term, singleton live‐born deliveries. Data was collected from two cohorts: Cohort 1 (January‐December 2016) pre‐CAP and Cohort 2 (January‐December 2019) post‐CAP implementation. Primary outcomes included incidence of both prenatal anemia overall and on admission to labor and delivery. Secondary outcomes included prenatal ferritin and IV iron orders. Outcomes were stratified by ethnicity.

**Results**: 68,839 patients were included in the study. The incidence of anemia was 38.7%. After the CAP project, diagnosis of anemia increased across all ethnicities 1,344 (3.8%) in 2016 vs. 4,901 (14.4%) in 2019 (P< 0.0001). The percentage of patients with anemia on admission to labor and delivery decreased 4,723 (36%) in 2016 vs. 4,398 (32.6%) in 2019 (P< 0.0001). Ferritin orders increased 2,802 (21.3%) in 2016 vs. 7,377 (54.6%) in 2019 (P< 0.0001) and IV iron infusions increased 407 (3.1%) in 2016 vs. 1,392 (10.3%) in 2019 (P< 0.0001). Black women had a 1.8 times higher incidence (IRR [95%CI]:1.8 [1.7‐1.9], p< 0.001) of anemia compared to white women. Identification of anemia increased in both black and white women after implementation of the CAP project 218 (8.9%) in 2016 vs 642 (25.2%) in 2019 in black women and 295 (2.2%) in 2016 vs 1290 (10.6%) in 2019.

**Conclusion**: The CAP protocol effectively improved the identification and treatment of prenatal anemia, with increased ferritin orders and IV iron treatments. Despite this, Black women remain disproportionately affected by anemia.

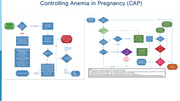

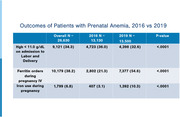

## 928 | Association Between Gestational Weight Gain and Short‐term Pregnancy Outcomes

Susan Dalton^1^; Amanda A. Allshouse^1^; Torri D. Metz^1^; Ann M. Bruno^2^

*^1^University of Utah, Salt Lake City, UT; ^2^University of Utah Health, Salt Lake City, UT*

10:30 AM ‐ 12:30 PM

**Objective**: Despite established Institute of Medicine (IOM) gestational weight gain (GWG) guidelines, there is ongoing debate as to whether these cutoffs are too liberal or strict, especially for pregnant people with obesity. We aimed to evaluate short‐term adverse pregnancy outcomes by GWG above or below IOM guidelines.

**Study Design**: Secondary analysis of a prospective cohort study of nulliparous, singleton pregnancies (2010‐2013). Pregnancies > 20 weeks’ were included while those with genetic or structural anomalies or missing outcome data were excluded. The exposure was GWG classified as below, within, or above IOM guidelines. Primary outcomes were composites of maternal and neonatal morbidity and mortality (Table), and secondary outcomes were their individual components. Those above or below recommended GWG were compared with those within IOM guidelines. Rates of outcomes by GWG category are reported. Multivariable modeling estimated the association between GWG and the selected outcomes.

**Results**: Of 8,997 pregnancies analyzed, 449 (32.2%) were below, 741 (30.5%) were within, and 2,205 (43.2%) were above IOM guidelines. Individuals within guidelines were more likely to have a normal pre‐pregnancy body mass index. The overall prevalence of composite maternal morbidity was 38.1% and composite neonatal morbidity was 24.0%. GWG above IOM guidelines was associated with higher maternal morbidity (43.2% vs 30.5%, aOR 1.48 95% CI 1.33‐1.65; Table). GWG above IOM guidelines was associated with a lower odds of neonatal morbidity (26.3% vs 27.2%, aOR 0.85, 95% CI 0.76‐0.95), with preterm delivery and SGA driving this relationship. GWG below IOM guidelines was associated with higher neonatal morbidity (aOR 1.55, 95% CI 1.34‐1.78; Figure).

**Conclusion**: While there is an association between GWG above IOM guidelines and maternal morbidity, there was an inverse relationship with neonatal morbidity. A better understanding of both short and long‐term adverse pregnancy outcomes based on GWG is needed.

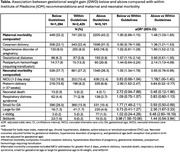

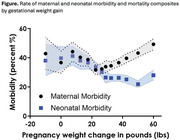

## 929 | Association Between the Intensity and Type of Exercise and the Risk of Gestational Diabetes

Takaki Tanamoto^1^; George R. Saade^2^; Tetsuya Kawakita^2^

*^1^United States Naval Hospital Yokosuka, Yokosuka City, Kanagawa; ^2^Macon & Joan Brock Virginia Health Sciences Eastern Virginia Medical School at Old Dominion University, Norfolk, VA*

10:30 AM ‐ 12:30 PM

**Objective**: While some studies have shown that exercise reduces the incidence of gestational diabetes mellitus (GDM), the recommended intensity of exercise has been anecdotal. Our objective was to examine the rate of (GDM) according to exercise intensity during pregnancy.   

**Study Design**: We analyzed data from the Nulliparous Pregnancy Outcomes Study: Monitoring Mothers‐to‐Be (nuMoM2b), in which patients were enrolled prospectively starting in the first trimester, and data was obtained by trained research coordinators. Individuals with pregestational diabetes or delivery < 20 weeks were excluded. The primary exposure was the intensity of exercise performed in the first trimester categorized according to metabolic equivalent hours per week (MET‐hr/week) into: none, low‐intensity (< 7.5 MET‐hr/week), moderate‐intensity (7.5‐14.9 MET‐hr/week), or high intensity (≧15 MET‐hr/week). Our primary outcome was GDM. We also examined a composite of adverse pregnancy outcomes, including preeclampsia, gestational hypertension, preterm birth, small‐for‐gestational‐age birth, and stillbirth. Adjusted relative risks (aRR) with 95% confidence intervals (95% CI) were calculated using modified Poisson regression, adjusting for potential confounders.

**Results**: Of 9,551 individuals, 3,132 (29.7%) had no exercise, 2,531 (26.5%) had low‐intensity exercise, 1,959 (20.5%) had moderate‐intensity exercise, and 2,223 (23.3%) had high‐intensity exercise. Demographics and socioeconomic status were different across the groups (Table 1). Compared to individuals with no exercise, those who performed high‐intensity exercise had a lower risk of GDM (5.1% vs. 2.8%; aRR 0.61; 95% CI 0.45‐0.82). However, other secondary outcomes remained similar (Table 2). Moderate and low‐intensity exercise compared to no exercise were not associated with significant differences in GDM or other outcomes.

**Conclusion**: High‐intensity exercise was associated with a lower risk of GDM compared to no exercise. Future intervention studies focusing on exercise to prevent GDM are warranted.

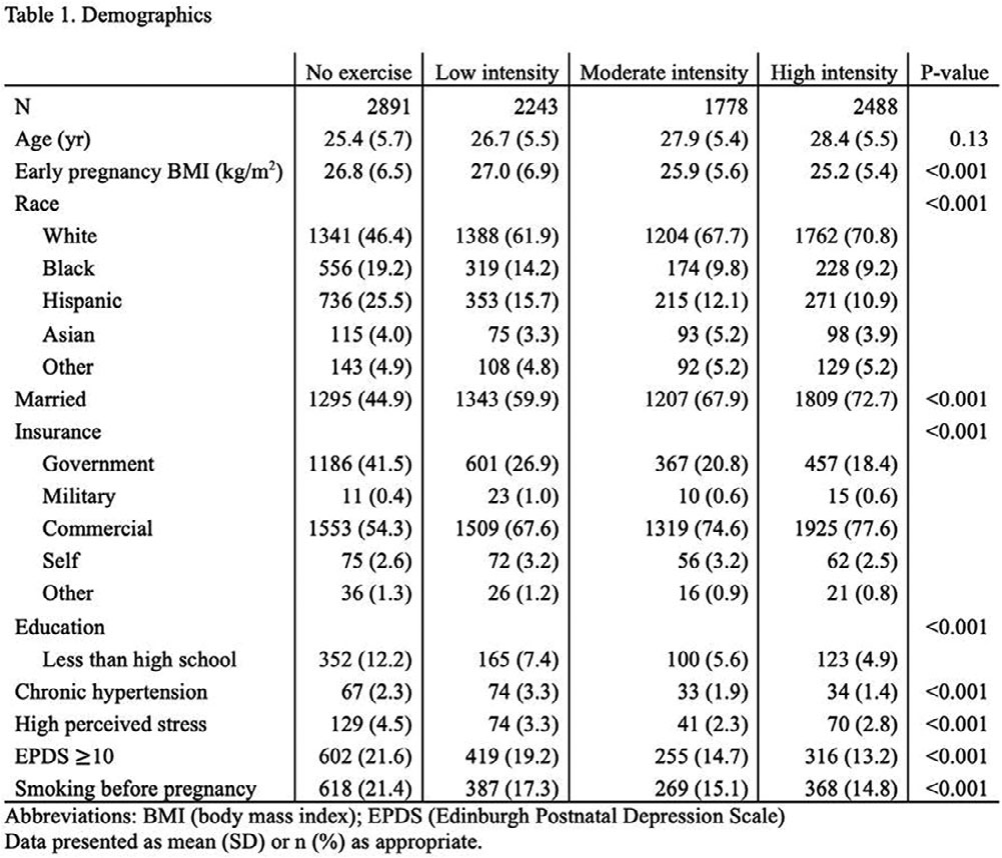

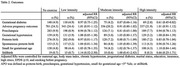

## 930 | Glycemic Status and Fetal Growth in Twin Gestations

Tasneem Sannah^1^; Stefanie Hinkel^2^; Lorraine Dugoff^2^

*^1^Corewell Health Grand Rapids, Grand Rapids, MI, MI; ^2^University of Pennsylvania, Philadelphia, PA*

10:30 AM ‐ 12:30 PM

**Objective**: In this study, we compared fetal growth curves in twin pregnancies affected by gestational diabetes with normoglycemic twin pregnancies. We hypothesized that fetal growth curves in twin gestations affected by gestational diabetes mellitus (GDM) would be significantly different from fetal growth curves for normoglycemic twin gestations, given the well‐established fetal growth acceleration which occurs in singleton pregnancies affected by GDM.

**Study Design**: This study is a secondary analysis of a retrospective cohort study of twin pregnancies conducted at 17 centers in the US. Our primary exposure group was GDM, and our primary outcome was estimated fetal weight (EFW) as a function of gestational age. Dichorionic twin pregnancies with data from at least one ultrasound were included in the study. Demographic data were collected and compared between the two groups. The means of EFW at each gestational week were calculated for both groups, plotted onto curves, and subsequently compared.

**Results**: Our results demonstrated no statistically significant difference between groups in terms of demographic characteristics. However, the comparison between fetal growth curves also revealed no significant difference between the two groups, so we were unable to reject our null hypothesis after this initial analysis of the primary outcome.

**Conclusion**: We aimed to evaluate the association of GDM with longitudinal fetal growth in twin pregnancies. Contrary to our hypothesis, we did not find a statistically significant difference in longitudinal fetal growth between normoglycemic twin pregnancies and twin pregnancies affected by GDM. These results are not entirely inconsistent with what has been demonstrated in prior literature; namely, that GDM likely does not affect fetal growth in twin pregnancies nearly as dramatically as it has been shown to affect fetal growth in singleton pregnancies. This may be because any accelerated growth caused by GDM in twins would be masked by the expected growth deceleration that has been demonstrated in twins in the third trimester.

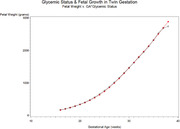

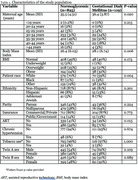

## 931 | Food Desert Proximity and Gestational Weight Gain During Pregnancy

Taylor Martin^1^; Babatamilore Alade^2^; Mary Taylor Winsten^3^; Kylie Mulvaney^4^; Felicia Hamilton^3^; Alexandra Piselli^2^; Fatimah Z. Fahimuddin^4^

*^1^Georgetown School of Medicine, Washington, DC; ^2^Georgetown University, Washington, DC; ^3^Medstar Washington Hospital Center, Washington, DC; ^4^MedStar Washington Hospital Center, Washington, DC*

10:30 AM ‐ 12:30 PM

**Objective**: A lack of access to healthy and nutritious food has been identified as an independent risk factor for pregnancy morbidity, but there is a lack of evidence outlining the relationship between gestational weight gain (GWG) and food desert (FD) proximity. In this study, we sought to evaluate the impact of living in FDs on GWG during pregnancy.

**Study Design**: This single‐center, retrospective study examined patients undergoing cesarean delivery (CD) at MedStar Washington Hospital Center from July 2021 to December 2022. Exclusion criteria included multifetal gestation, prenatal care outside of our hospital system, and entry into prenatal care after 14 weeks of gestation. Patients were classified as having low food access (LFA) if they lived more than 1 mile (urban) or 10 miles (rural) from the nearest supermarket. GWG was defined as inadequate, adequate, or excessive weight gain based on pre‐pregnancy BMI as delineated by ACOG recommendations. Multivariate multinomial logistic regression models compared patients with LFA and GWG as the primary outcome, adjusting for insurance type, marital status, and low income area.

**Results**: A total of 370 patients were included in the study, of whom 73 (19.7%) lived in an LFA neighborhood. Women in the LFA category had an average weight gain of 13.4 kg as compared to 11.84 kg for those that lived in food secure neighborhoods, although this difference was not statistically significant (p = 0.105). When assessed by pre‐pregnancy BMI category, we found a directional trend suggesting that living in LFA neighborhood was associated with excessive weight gain, but this result was also not statistically significant (aOR: 0.60 [0.324‐1.101]).

**Conclusion**: Living in a LFA community showed no statistically significant correlation for excessive weight gain when compared to patient's living in communities with adequate food access. This study emphasizes the need for further exploration of alternative factors that may be impacting the discrepancy in pregnancy morbidity in these populations.

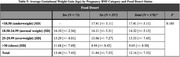

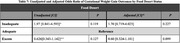

## 932 | Association between Social Vulnerability Index and Adverse Pregnancy Outcomes

Tetsuya Kawakita^1^; Lindsay S. Robbins^1^; George Mcleod^2^; George R. Saade^1^

*^1^Macon & Joan Brock Virginia Health Sciences Eastern Virginia Medical School at Old Dominion University, Norfolk, VA; ^2^Old Dominion University, Norfolk, VA*

10:30 AM ‐ 12:30 PM

**Objective**: To examine the association between the social vulnerability index (SVI) and adverse pregnancy outcomes, including maternal mortality.

**Study Design**: This was a cross‐sectional study of county‐level data analysis of individuals who delivered in the United States from 2016 to 2021. The study population includes individuals aged 10‐44 at the time of delivery who gave live births, stillbirths, or suffered maternal death. The SVI ranges 0‐1, with 1 being the highest vulnerability. Counties were categorized based on the SVI quartile (Q1 < 25^th^ percentile, Q2 25‐49^th^ percentile, Q3 50‐74^th^ percentile, Q4 75^th^ or greater). Our primary outcome was maternal mortality, defined as “the death of a woman while pregnant or within 42 days of termination of pregnancy.” Secondary outcomes included maternal mortality up to one year postpartum, stillbirth, and preterm birth < 37 weeks. We conducted Bayesian spatial models with Conditional Autoregressive Priors to calculate log relative risk (RR) with 95% confidence intervals (95%CI), which considered spatial autocorrelation.

**Results**: Of 22,667,460 births, there were 6,731 maternal mortalities, 9,702 mortalities up to one year postpartum, 275,884 stillbirths, 2,285,797 preterm births, and 7,225,076 cesarean deliveries from 3,012 counties. Maternal mortality rate (MMR) per 100,000 births and SVI categories are shown in Figure 1. As the SVI increases, incidences of maternal mortality increase (Q1 22.8; Q2 25.7; Q3 31.1; and Q4 34.5 per 100,000 births) (Table 1). Higher SVI groups had increased risks of maternal mortality (Q2 RR 1.10, 95%CI 0.99‐1.22; Q3 RR 1.27, 95%CI RR 1.15‐1.41; Q4 RR 1.54, 95%CI 1.39‐1.72). Higher SVI groups also had increased risks of other adverse pregnancy outcomes (Table 1).

**Conclusion**: High SVI is associated with an increased risk of adverse pregnancy outcomes. The SVI could be used to identify counties that need policies to address health disparities.

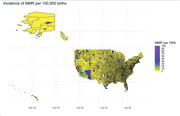

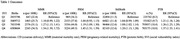

## 933 | Association Between the Dietary Inflammatory Index and Adverse Pregnancy Outcomes

Ummayhany F. Bharmal^1^; George R. Saade^2^; Tetsuya Kawakita^2^

*^1^Medical College Baroda, Medical College Baroda, Gujarat; ^2^Macon & Joan Brock Virginia Health Sciences Eastern Virginia Medical School at Old Dominion University, Norfolk, VA*

10:30 AM ‐ 12:30 PM

**Objective**: The impact of pre‐conception inflammatory load has not been adequately evaluated. We sought to determine whether a pre‐conception diet that promotes inflammation, as measured by the dietary inflammatory index (DII), is associated with adverse pregnancy outcomes.

**Study Design**: This is a secondary analysis of data from the Nulliparous Pregnancy Outcomes Study: Monitoring Mothers‐to‐Be (nuMoM2b) study, in which dietary habits were assessed for three months before conception via a food frequency questionnaire. Data was obtained through patient interviews and chart abstraction. Individuals with spontaneous abortion or missing dietary data were excluded. DII was calculated from 28 pro‐and anti‐inflammatory food items. Participants were classified into DII quartiles, with the highest quartile indicating the most pro‐inflammatory diet. The primary outcome was a composite of adverse pregnancy outcomes including preeclampsia, preterm birth less than 37 weeks, small for gestational age less than 5th percentile, and stillbirth. Adjusted relative risks (aRR) along with the 95% confidence intervals (CI) were calculated for all outcomes, using modified Poisson regression while controlling for confounders.

**Results**: Of the 7,911 participants included in this analysis, 1,443 (18.2%) had an adverse pregnancy outcome. Demographics and socioeconomic factors including age, race, marital status, insurance, education, and depression varied across the groups (Table 1). Outcomes according to DII quartiles are presented in Table 2. Individuals in the highest DII quartile compared with those in the lowest quartile had an increased risk of the primary outcome (20.8% vs 17.4%, aRR: 1.17, 95% CI: 1.02‐1.34) as well as stillbirth (0.9% vs. 0.3%; aRR 2.94, 95% CI: 1.04‐8.32). Other secondary outcomes were not statistically significant.

**Conclusion**: A pro‐inflammatory diet in the immediate preconception period is associated with a composite of adverse pregnancy outcomes. Investigation of pre‐pregnancy nutritional interventions is warranted.

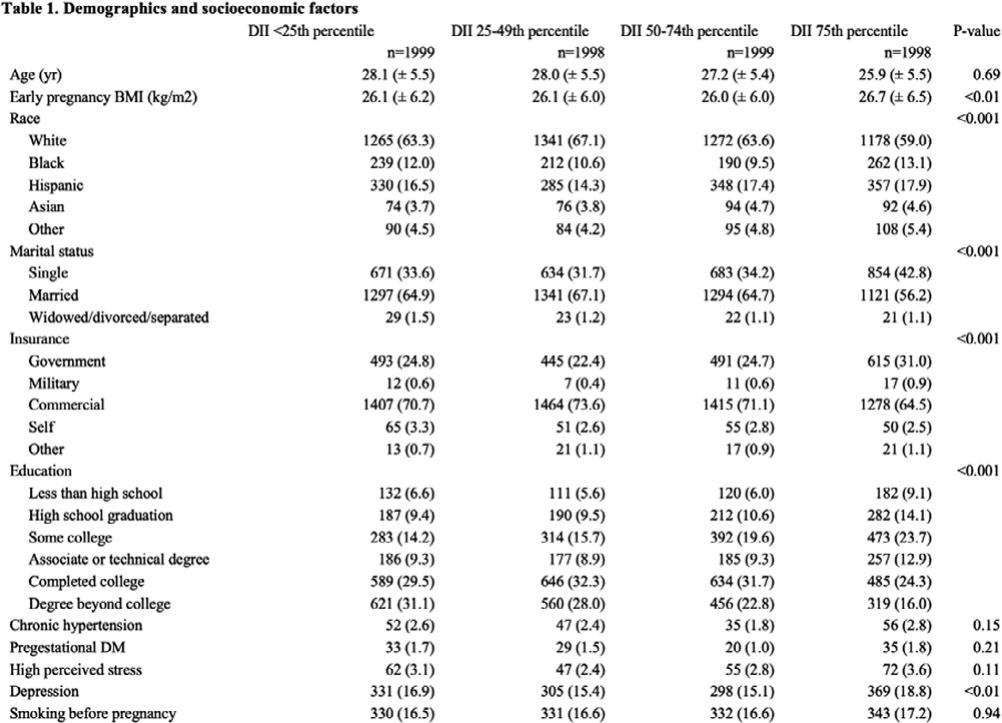

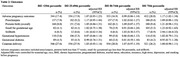

## 934 | Association between Stillbirth and the Social Vulnerability Index

Tetsuya Kawakita^1^; Ann Harper^2^; John Brush^2^; Misa Hayasaka^3^; George R. Saade^1^

*^1^Macon & Joan Brock Virginia Health Sciences Eastern Virginia Medical School at Old Dominion University, Norfolk, VA; ^2^Sentara Health Research Center, Norfolk, VA; ^3^Department of Obstetrics and Gynecology, Macon and Joan Brock Virginia Health Sciences at ODU, Norfolk, VA*

10:30 AM ‐ 12:30 PM

**Objective**: To examine the association between stillbirth and the social vulnerability index using electronic data from a large health system.

**Study Design**: This was a retrospective cohort study of pregnant individuals aged 18‐50 who delivered singleton fetuses at any of 6 hospitals within our healthcare system from 2015 to 2022 and were 20 weeks or greater. We excluded pregnancies with known major fetal anomalies or chromosomal abnormalities and intrapartum stillbirth. Individuals with their unavailable addresses were also excluded. Stillbirth was defined as the birth of a fetus without signs of life at 20 weeks’ gestation or greater. The social vulnerability index (SVI) was obtained based on the census tract that pregnant individuals lived in. The overall SVI and the four domains of SVI (Socioeconomic status, household characteristics, racial and ethnic minority status, and housing type and transportation) were compared between stillbirths and live births. Multivariable logistic regression was used to calculate adjusted odds ratios (aOR) with 95% confidence intervals (95%CI), adjusting for confounders (1^st^ quartile as referent).

**Results**: Of 61,323 pregnancies, 603 (1.0%) had stillbirths and 60720 (99.0%) had live births. The distribution of the SVI in our region is presented in Figure 1. Compared to live births, stillbirths were associated with increased odds of higher overall SVI quartiles (3^rd^ quartile aOR 1.54, 95%CI 1.22‐1.96; 4^th^ quartile aOR 1.45, 95%CI 1.13‐1.87; Table 1). In the socioeconomic status domain, stillbirths were associated with increased odds of 3^rd^ quartile SVI (but not 4^th^ quartile). Household characteristics status domain SVI was not associated with increased odds of stillbirth. In the racial and ethnic minority status domain and housing type and transportation domain, stillbirth was associated with 4^th^ quartile SVI.

**Conclusion**: The overall SVI and the socioeconomic status, racial and ethnic minority status, and housing type and transportation SVI domains are significantly associated with increased odds of stillbirth.

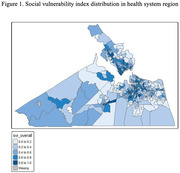

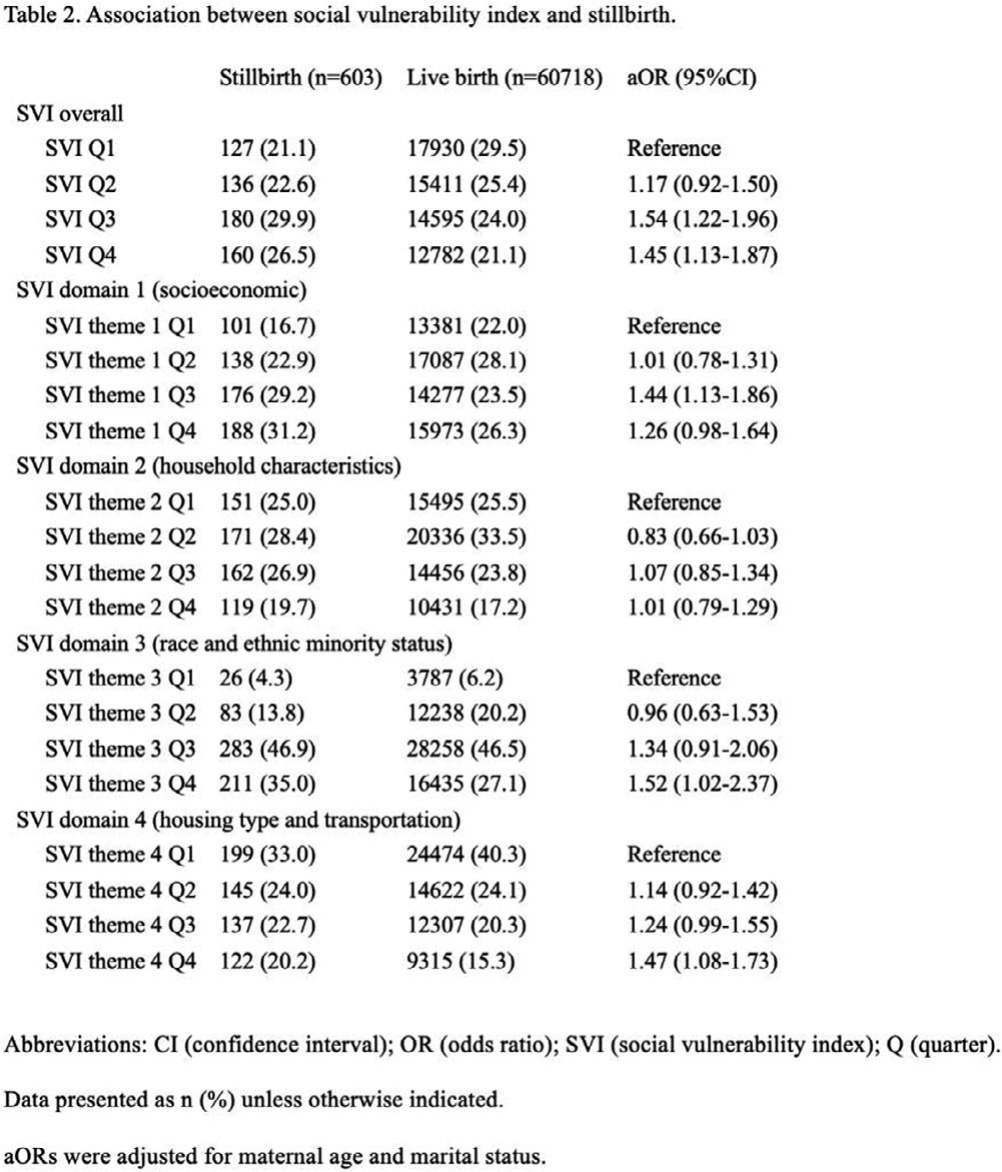

## 935 | Ethnic/Racial Differences in Pregnancy Outcomes for Patients Requiring Antepartum Antihypertensive Maintenance for Non‐severe Hypertension

Tiffany Yang^1^; Hannah K. Agoglia^2^; David Garry^1^; Samantha Gobioff^3^; Cassandra Heiselman^1^

*^1^Stony Brook University Hospital, Stony Brook, NY; ^2^Renaissance School of Medicine at Stony Brook University, Stonybrook, NY; ^3^Stony Brook University, Stonybrook, NY*

10:30 AM ‐ 12:30 PM

**Objective**: This study aimed to determine differences in pregnancy outcomes and hypertension control for pregnant patients requiring antepartum antihypertensive maintenance across race/ethnicity groups.

**Study Design**: Patients who received prenatal care at an academic institution with a non‐severe hypertensive disorder (chronic, gestational, preeclampsia) requiring oral antihypertensive maintenance medications were included. Medications had to be initiated prior to 34 weeks for a minimum of 2 weeks. Pregnancies with fetal anomalies and multiples were excluded. Patients were stratified by self‐identified race/ethnicity. Hypertension outcomes included dosage changes and adherence, escalation in hypertension diagnosis (i.e. chronic to preeclampsia), and acute management of severe hypertension intrapartum. Pregnancy outcomes included mode of delivery, composite peripartum complications, GA at delivery, and BW. Statistics done with significance level of p < 0.05.

**Results**: 121 patients met inclusion criteria: 86 (71.1%) White, 16 (13.1%) Black, 6 (5.0%) Asian, and 10 (8.3%) Hispanic. Non‐White patients experienced higher rates of antenatal dosage changes (80% v 55%, p = 0.046), escalation in hypertensive diagnosis (64% v 38%, p = 0.02), and need for acute antihypertensives intrapartum (42% v 21%, p = 0.02) compared to White patients (Table 1). Black patients had higher rates of medically‐indicated preterm delivery (69% v 36%, p = 0.01), peripartum complications (31% vs 11%, p = 0.04), and medication non‐adherence (33% v 8%, p = 0.01) compared to non‐Black patients (Table 2). Asian patients had higher initial diastolic pressures upon delivery (99.5±26.1 v 85.8±13.9, p = 0.03) compared to non‐Asian patients. Hispanic patients were less likely to have cesarean delivery (30% v 64.9%, p = 0.04) compared to all others.

**Conclusion**: Non‐White patients have a higher risk for inadequate blood pressure control, requiring more antihypertensive medication, along with greater difficulty with medication adherence. To optimize maternal outcomes equally across all racial/ethnic groups, understanding specific risks and needs remain paramount.

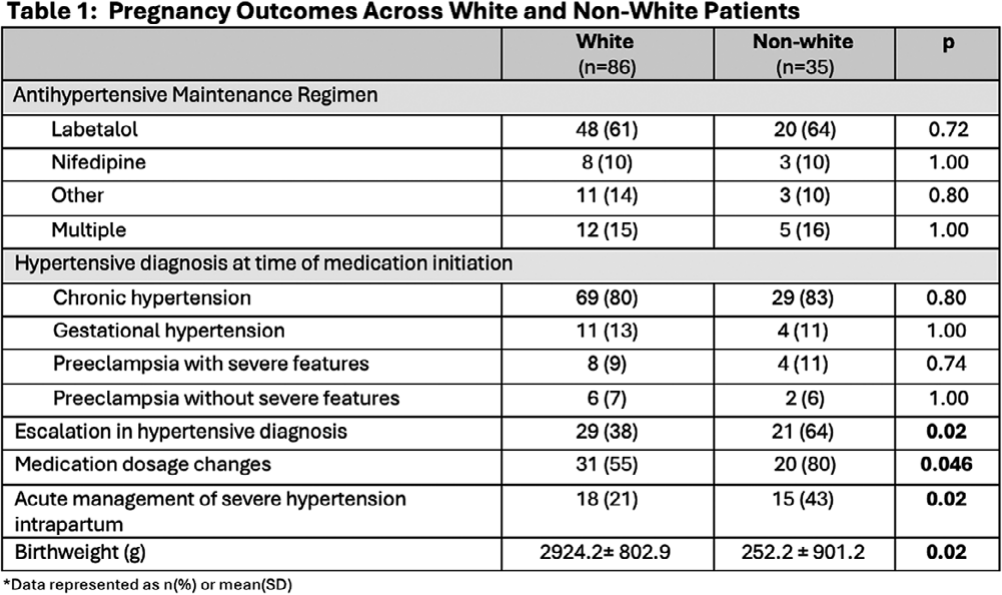

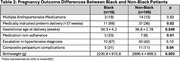

## 936 | The Impact of Inter‐Pregnancy‐Interval After Delivery of SGA Fetus on Recurrence Rate ‐ Multicenter Study

Tzory Jan Misgav^1^; Hen Y. Y. Sela^1^; Tzuria Peled^2^; Sorina Grisaru Granovsky^3^; Misgav Rottenstreich^4^

*^1^Shaare Zedek, Jerusalem, Yerushalayim; ^2^Shaare Zedek Medical Center, Shaare Zedek Medical Center, Yerushalayim; ^3^Hebrew University, Jerusalem, Yerushalayim; ^4^McMaster University, Hamilton, ON*

10:30 AM ‐ 12:30 PM

**Objective**: To evaluate the association between IPI after delivering an SGA infant and the recurrence of this condition in the subsequent pregnancy. This study aims to identify high‐risk populations for recurrence and provide counseling on the recommended waiting period before the next pregnancy.

**Study Design**: **Methods**: A multi‐center retrospective cohort study. The study population included all women who delivered a live SGA infant between 24‐42 weeks of gestation and subsequently conceived and delivered in all university‐affiliated obstetrical centers in a single geographic area between 2003 and 2021. The study population was divided into several IPI groups, with an IPI of 18 to 23 months serving as the reference group. The primary outcome measured was the recurrence rate of SGA in the subsequent pregnancy.

**Results**: During the study period, 12,689 women who delivered an SGA infant and subsequently delivered again were identified. The control group (IPI between 18‐23 months) included 1,765 women. The overall recurrence rate of SGA in the study population was 19.3%. Univariate analysis showed that an IPI of less than 3 months and an IPI between 6 to 11 months were associated with higher rates of SGA recurrence (24.6% vs. 17.5%, p< 0.01 and 20% vs. 17.5%, p = 0.03, respectively). However, multivariate analysis controlling for confounders such as maternal age, gravidity, parity, birthweight percentile at first delivery, and smoking showed no significant association between different IPI groups and SGA recurrence compared to the control group.

**Conclusion**: Approximately one‐fifth of women with a previous SGA child will experience a recurrence. No significant association was found between IPI and SGA recurrence. Further studies are needed to identify factors that can prevent SGA recurrence.

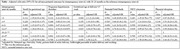

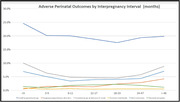

## 937 | Clinical Outcomes and Placental Findings in Pregnant Women with Covid‐19 Wild‐Type, Delta, and Omicron Waves

Lindsay B. Howard^1^; Vincent Tang^1^; Shekinah Dosunmu^1^; Joanne N. Quiñones^1^; Kyle Shaak^2^; Janae Cornwall^3^; Aishwarya Pattnaik^3^; Victoria Loven^1^; Albert Sarno^1^; Matthew P. Romagano^1^

*^1^Lehigh Valley Health Network, Allentown, PA; ^2^Network Office of Research and Innovation, Lehigh Valley Health Network, Allentown, PA; ^3^University of South Florida Morsani College of Medicine, SELECT Program, Tampa, FL*

10:30 AM ‐ 12:30 PM

**Objective**: To compare placental histologic findings to perinatal outcomes from pregnant patients with COVID‐19 based on predominant SARS‐CoV‐2 variant at time of infection.

**Study Design**: Retrospective cohort study of pregnant patients between March 1, 2020 to February 28, 2023 with confirmed PCR‐positive COVID‐19 in singleton pregnancy > 6 weeks and available placental pathology. Exclusion criteria: patients with preexisting risk factors for abnormal placentation. Exposures: COVID‐19 wild‐type, Delta and Omicron strains. Primary outcomes: placental findings [maternal vascular malperfusion (MVM), fetal vascular malperfusion (FVM), and infectious/inflammatory findings], development of hypertensive disorders of pregnancy (HDP), and spontaneous or indicated preterm birth. Placental findings were categorized by strain, infection severity, trimester. Delivery timing was categorized by placental findings. Univariate techniques were utilized.

**Results**: 708 out of 1536 patients met inclusion criteria. COVID symptoms and placental findings were similar across strains (Table 1). The three cases of severe placental findings were seen in patients with Omicron that were asymptomatic or had mild symptoms. Four patients with severe/critical symptoms had non severe placental findings. HDP after COVID infection was similar across all strains (Table 1). The proportion of patients with placental findings was similar across all trimesters (Table 2). MVM was more common in full‐term deliveries, likely explained by the common finding of villous infarction < 5%. Placental inflammatory/infectious findings were significantly associated with spontaneous preterm birth (45.9% vs. 24.5% full‐term, 20.0% indicated preterm; p = 0.01).

**Conclusion**: Our data does not suggest a difference in placental findings based on COVID strain, infection severity, or trimester. Placental inflammatory/ infectious findings were most common in patients who experienced spontaneous preterm birth.

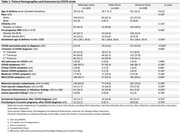

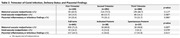

## 938 | Perinatal Outcomes Following Implementation of a Remote Patient Monitoring Program for Hypertensive Disorders in Pregnancy

Vincent Tang; Matthew P. Romagano; Joanne N. Quiñones; Danielle Durie; Shae Duka; Nichola Bomani Gonzalez; Ebtisam Zeynu; Meredith Rochon

*Lehigh Valley Health Network, Allentown, PA*

10:30 AM ‐ 12:30 PM

**Objective**: Telehealth and remote patient monitoring programs (RPM) are used increasingly in obstetrics to improve care and decrease hospital admissions. Our goal was to evaluate the impact on perinatal outcomes of an RPM for management of antepartum hypertensive disorders of pregnancy (HDP).

**Study Design**: Retrospective cohort study of singleton gestations with HDP < 37 weeks expectantly managed before and after implementation of an RPM for HDP (controls 6/2020‐5/2022, RPM 6/2022‐4/2024). Patients were excluded if they had severe features or an indication for immediate delivery. RPM management was outpatient and included remote vital signs, nurse phone visits, and fetal surveillance. Control group management was both inpatient and outpatient at the discretion of the obstetrician. Primary outcome was gestational age (GA) at delivery. Secondary outcomes were readmission rates and select perinatal outcomes.

**Results**: 162 patients were identified: 66 RPM and 96 controls (Table 1). Median GA at enrollment and duration in RPM was 32.9 wks (IQR 29.9‐34.6) and 20.0 days (IQR 9‐31), respectively. RPM patients were more likely to have underlying chronic hypertension (cHTN); demographics were otherwise similar. Controls were more likely to deliver during the index hospitalization and develop severe features; RPM were more likely to be readmitted. RPM participants had a longer latency period between HDP diagnosis and delivery (27.5 vs 14.0 days, p = 0.0036), delivered at a later GA (35.9 vs 35.4 wks, p = 0.0051), and were more likely to have a planned delivery at 37 wks (31.8% vs 0%, p< 0.0001). NICU admission rate was higher in the control group (78.1% vs 57.6%, p = 0.0051), primarily due to prematurity. After adjusting for cHTN, preeclampsia history and BMI, RPM was associated with pregnancy prolongation of 0.949 wks (95% CI 0.188, 1.710), an 83% reduction in development of severe features [AOR 0.169 (95% CI 0.073, 0.391), p< 0.0001] and a 60% reduction in NICU admission [AOR 0.398 (95% CI 0.191, 0.833), p = 0.0144).

**Conclusion**: Use of RPM for outpatient management of preterm HDP is safe and may improve select perinatal outcomes.

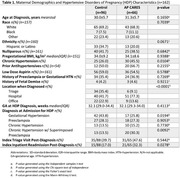

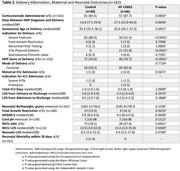

## 939 | Randomized Trial of Postpartum Aspirin and Impact on Nt‐Probnp as a Marker of Maternal Health

Virginia Y. Watkins; Anthony E. Melendez Torres; Maria C. Prado; Eden Singh; Jerome J. Federspiel; Sarah C. Snow; Sarahn M. Wheeler; Shakthi Unnithan; Alaattin Erkanli; Brenna L. Hughes

*Duke University School of Medicine, Durham, NC*

10:30 AM ‐ 12:30 PM

**Objective**: To compare levels of NT‐proBNP, a marker of cardiac dysfunction, and clinical outcomes in patients at risk of preeclampsia randomized to continuation of low‐dose aspirin (LDA) or placebo for 6 weeks postpartum.

**Study Design**: This double‐blinded randomized‐controlled trial included patients at risk of preeclampsia randomized in a 1:1 fashion to LDA (81 mg) versus an identical‐appearing placebo continued for 6 weeks postpartum (Table 1). The primary outcome was NT‐proBNP levels at the postpartum visit. A sample size of 90 was required to detect a clinically significant difference in NT‐proBNP between LDA and placebo with 80% power. A sample size of 110 subjects was planned due to anticipated loss to follow‐up in the postpartum period. Pre‐specified secondary outcomes were postpartum preeclampsia, eclampsia, hospital readmission, initiation or titration of blood pressure medications, and blood transfusion. The primary outcome was compared using the Wilcoxon rank sum test. Secondary outcomes are reported as counts (%) and were compared using Fisher's exact test after adjusting for multiple testing using Bonferroni correction.

**Results**: From July 2023 to March 2024, 110 participants were randomly assigned to LDA (n = 55) or placebo (n = 55). Baseline demographics were similar between groups (Table 1). There was no difference in the primary outcome of NT‐proBNP levels between groups (median (IQR), 36.5 ng/mL (36.0, 61.0) vs. 39.5 ng/mL (36.0, 74.0), p = 0.49). Rates of postpartum preeclampsia in patients randomized to LDA vs. placebo (5.6% vs. 12.7%) were numerically but not statistically significantly lower, with similar rates of hospital readmission and bleeding complications (Table 2). There were no cases of postpartum eclampsia or blood transfusion. Adherence was higher in the LDA (75% vs. 69%) than the placebo group.

**Conclusion**: Continuation of LDA after delivery was not associated with decreased NT‐proBNP levels compared to placebo. Additional studies powered to detect differences in maternal outcomes are needed to evaluate the role of LDA in the postpartum period.

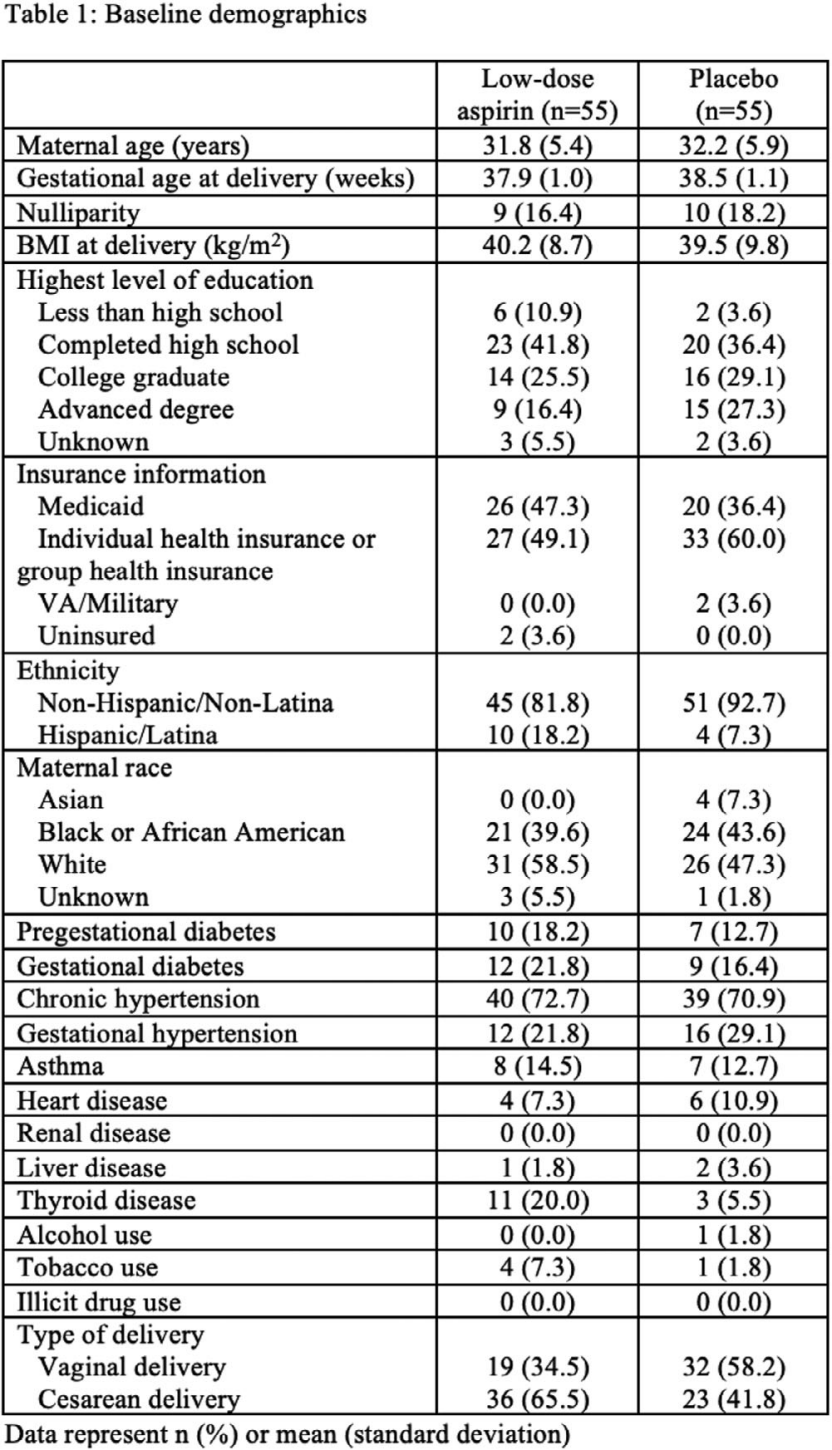

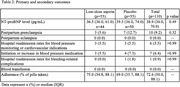

## 940 | Concordance of Clinical GBS Status with Bacterial Culture Obtained at Delivery Admission

William L. Riley^1^; Callie Reeder^1^; Jill M. Maples^2^; Kimberly B. Fortner^1^; Jessica Da Conceicao Mendonca^3^; Zachary Burcham^3^; Lindsey Burcham^3^

*^1^University of Tennessee Graduate School of Medicine, Knoxville, TN; ^2^University of Tennessee Health Science Center, College of Medicine, Knoxville, TN; ^3^University of Tennessee Knoxville, Knoxville, TN*

10:30 AM ‐ 12:30 PM

**Objective**: To evaluate the vaginal microbiome at term and compare routine prenatal GBS screening with culture data obtained through specimen collection at delivery hospital admission.

**Study Design**: This prospective cohort study, funded by an institutional Human Health and Wellness grant, enrolled 50 term‐pregnant patients to assess the vaginal microbiome through culture and 16S full‐length sequencing approaches at delivery admission. Following consent, a sterile speculum examination was performed for vaginal swab and lavage. Records were reviewed for demographics, clinical GBS status, and obstetric outcomes.

**Results**: Clinical GBS status was ascertained from routine antenatal care records, and bacterial culture data were obtained from vaginal swab at the time of delivery admission for 39 participants. Of these, 9/38 (24%) were GBS‐positive and 19/38 (50%) were GBS‐negative by both sampling methods, giving concordant results in 28/38 patients (74%). Four (11%) participants had negative routine GBS screening but were GBS‐positive by culture at admission, three (8%) had positive GBS clinical screens without GBS detected by culture at admission, and three (8%) were missing either data point (Figure 1). One participant with GBS bacteriuria followed by negative routine GBS screen was GBS‐positive by culture on admission. Twenty‐eight (74%) patients were colonized with opportunistic pathogens including *S. agalactiae, E. faecalis, E. faecium, S. aureus, S. epidermidis* and *E. coli*.

**Conclusion**: While ∼75% of clinical GBS status and admission cultures agreed, 14% likely received unnecessary antibiotics during labor. More importantly, 11% of participants did not receive antibiotic prophylaxis due to negative routine screening; however, were found to be colonized with GBS by culture. Future work includes complete microbiome analyses to evaluate unrecognized GBS colonization due to opportunistic pathogen overgrowth and determination of GBS capsular serotype distribution for downstream molecular analyses. Clinical questions still exist regarding the optimal method, timing, and cost efficiency for determining GBS status.

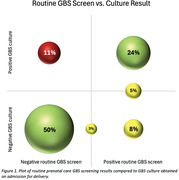

## 941 | Race, Ethnicity, and Outcomes in Pregnancies with Gestational Diabetes: a Secondary Analysis of Numom2B Dataset

Xiang Yu Feng^1^; Jenny Chang^2^; Argyrios Ziogas^2^; Rebecca J. Post^2^; Judith H. Chung^3^; On behalf of the nuMoM2b Network

*^1^University of California, Irvine Division of Maternal‐Fetal Medicine, University of California, Irvine, CA; ^2^University of California Irvine, Orange, CA; ^3^University of California‐ Irvine, Irvine, CA*

10:30 AM ‐ 12:30 PM

**Objective**: Studies have demonstrated higher rates of gestational diabetes in racial and ethnic minorities. Management of gestational diabetes requires various dietary and lifestyle changes in pregnancy. This study evaluates the association between race, ethnicity, specific risk factors and outcomes in nulliparous pregnancies affected by gestational diabetes.

**Study Design**: This study is a secondary analysis of the Nulliparous Pregnancy Outcomes Study: Monitoring Mothers‐to‐be (nuMoM2b), a prospective cohort study following nulliparas throughout pregnancy. Patients diagnosed with gestational diabetes were analyzed by racial/ethnic groups. Primary outcome was neonatal hypoglycemia. Secondary outcomes were selected neonatal and maternal morbidities. ANOVA test and Fisher's exact were used for analysis of difference in race/ethnicity group. Multivariate logistic regression was used to adjust for confounders.

**Results**: Of the cohort of 10,037 patients, 396 were diagnosed with gestational diabetes, with 54.5% patients identifying as non‐Hispanic White, 10.4% non‐Hispanic Black, 18.7% Hispanic, and 16.4% all other. Variance by race/ethnicity was found for multiple characteristics such as age, body mass index, dietary caloric and carbohydrate intake, chronic hypertension, socioeconomic status, health literacy, and experience of racism (all p values < 0.047). After adjusting for these potential confounders, rates of neonatal hypoglycemic were not statistically significant. However, rate for cesarean was higher for non‐Hispanic Black patients (aOR 2.18, 95% CI 1.02‐4.70) and macrosomia higher for Hispanic patients (aOR 2.98, 95% CI 1.10‐8.09).

**Conclusion**: Variance was observed in racial/ethnic groups for multiple characteristics including dietary caloric intake and carbohydrate intake in patients with gestational diabetes. Although odds of neonatal hypoglycemia were not significant between groups, differences in cesarean delivery and macrosomia rates were seen. These findings may impact developing culturally individualized care in counseling and management of gestational diabetes.

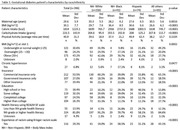

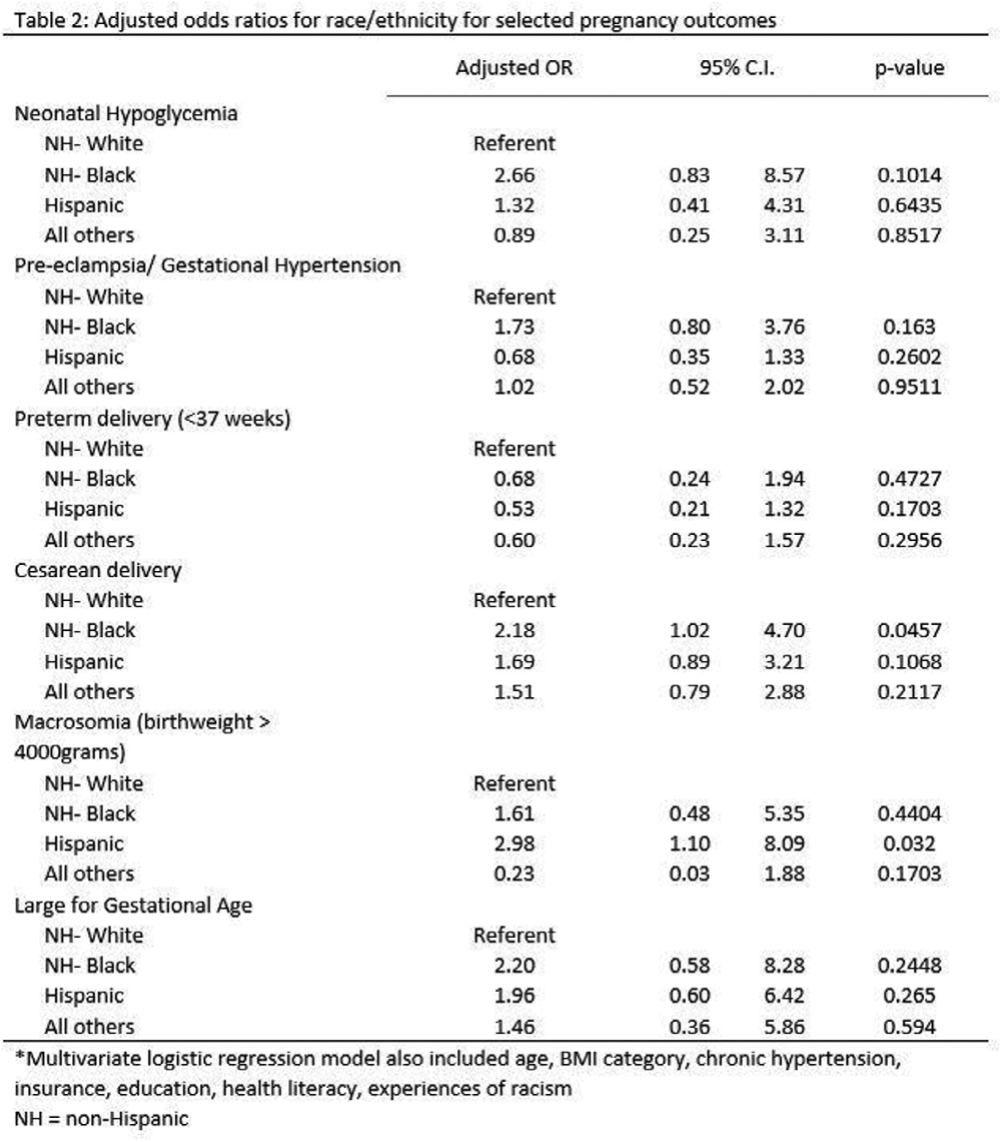

## 943 | Betamethasone for Preterm Premature Rupture of Membranes at Late Prematurity

Yossi Bart^1^; Michal Fishel Bartal^2^; Farah H. Amro^1^; Sean C. Blackwell^1^; Baha M. Sibai^1^

*^1^McGovern Medical School at UTHealth Houston, Houston, TX; ^2^UTH Houston & Sheba Medical Center Israel, Houston, TX*

10:30 AM ‐ 12:30 PM

**Objective**: As of 2020, the American College of Obstetricians and Gynecologists extended the upper limit of expectant management of preterm premature rupture of membranes (PPROM) to 36 weeks. Treatment with betamethasone (BMZ) in the subset of patients with PPROM in the late preterm period has not been well studied. Therefore, we sought to investigate this.

**Study Design**: A secondary analysis of the Antenatal Late Preterm Steroids (ALPS) trial. All individuals enrolled in the parent trial were included. The primary outcome was a composite of respiratory support at 72 h, including continuous positive airway pressure or high flow nasal cannula ≥ 2 h, oxygen with an inspired fraction of ≥ 30% for ≥ 4 h, or mechanical ventilation, together with extracorporeal membrane oxygenation, or stillbirth or neonatal death within 72 h after delivery. Poisson regression was implemented to adjust for confounders.

**Results**: Overall, 22% (620/2,831) had PPROM upon enrollment. Among individuals with PPROM, compared to those without, the median latency period was 15.6 hours (IQR 8.3‐24.1) versus 45.0 hours (IQR 20.0‐158.0; P< 0.001) (Figure). Among the patients who received BMZ, two doses were given to 26% (83/316) of those with PPROM vs. 70% (775/1,110; P< 0.001) of those without. Latency ≥ 48 hours, compared to < 48 hours, was associated with a lower primary outcome rate: 9% (94/1,078) vs. 16% (273/1,749); P< 0.001.

Among patients with PPROM, 49% (304) received BMZ and 51% (316) received placebo. The BMZ and placebo groups had similar baseline characteristics. In this subgroup, there was no difference in the primary respiratory outcome rate (adjusted RR 0.91, 95% CI 0.60‐1.38) between BMZ and placebo (Table). Secondary neonatal and maternal outcomes did not differ as well.

**Conclusion**: Compared to those without, individuals with PPROM in the late preterm period had shorter latency; three out of four delivered within 24 hours from randomization. Among individuals with PPROM, there were no differences in neonatal morbidity following treatment with BMZ compared to placebo. However, our study was underpowered to detect such differences.

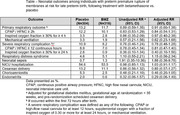

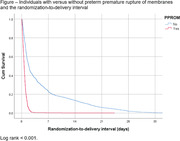

## 944 | Novel Roles of Et‐1 in Superimposed Preeclampsia

Yuye Wang^1^; Rebecca Ssengonzi^2^; Feng Li^2^; Nobuyo Maeda‐Smithies^2^

*^1^University of North Carolina at Chapel Hill, Durham, NC; ^2^University of North Carolina at Chapel Hill, Chapel Hill, NC*

10:30 AM ‐ 12:30 PM

**Objective**: This study aims to determine if low maternal endothelin‐1 (ET‐1) expression causes preeclampsia‐like phenotypes during pregnancy using mice with modified *Edn1* alleles (L) that have ∼30% of the wild type (WT) circulating ET‐1 levels.

**Study Design**: At 8‐10 weeks of age, *Edn1^L/L^* females were mated with *Edn1^L/L^* males, and WT females were mated with WT males. One batch of dams from each group was sacrificed at 18.5 days post coitus (dpc) and maternal urine, plasma, and kidneys were collected. Urine albumin, and serum markers of preeclampsia—soluble fms‐like tyrosine kinase 1 (sFlt‐1), vascular endothelial growth factor (VEGF), and soluble endoglin (sENG)—were measured using ELISA kits. Electron microscopy of the glomeruli was examined at 18.5 dpc. A separate batch of dams from each group was reared through term delivery (19.5 to 21.5 dpc). Tail‐cuff blood pressure measurement was performed on WT and *Edn1^L/L^* female mice before pregnancy, at 13.5 dpc, at 17.5 dpc, and at 7 days postpartum.

**Results**: Virgin *Edn1^L/L^* females were hypertensive but did not demonstrate any other obvious abnormalities. *Edn1^L/L^* females developed the full spectrum of preeclampsia‐like phenotypes during pregnancy, including elevated systolic blood pressure and urinary albumin secretion, and glomerular endothelial damage. On the day of delivery, *Edn1^L/L^* dams had fewer live neonates. At 18.5 dpc, *Edn1^L/L^* dams also had fewer live fetuses and reduced fetal weight. The placental weights were not different between *Edn1^L/L^* and WT dams. Interestingly, all serum markers of PE were lower in *Edn1^L/L^* dams compared to WT dams.

**Conclusion**: Our results show low maternal ET‐1 expression causes preeclampsia‐like phenotypes during pregnancy and leads to adverse pregnancy outcomes in mice.

## 945 | Evaluating Associations Between a Continuous Measure of Midtrimester Cervical Length and Gestational Age at Delivery

Zahava P. Michaelson^1^; Daniel L. Kuhr^1^; Nicola F. Tavella^2^; Raina Kishan^2^; Nicole Parkas^1^; Helen Feltovich^1^; Kimberly B. Glazer^3^

*^1^Division of Maternal‐Fetal Medicine, Icahn School of Medicine at Mount Sinai, New York, NY; ^2^Icahn School of Medicine at Mount Sinai, New York, NY; ^3^Department of Population Health Science and Policy; Department of Obstetrics, Gynecology and Reproductive Science, Blavatnik Family Women's Health Research Institute, Icahn School of Medicine at Mount Sinai, New York, NY*

10:30 AM ‐ 12:30 PM

**Objective**: A dichotomous measure of short cervical length (CL) (< 25 mm) is correlated with spontaneous preterm birth (sPTB) but cannot predict gestational age (GA) at delivery. Our aim was to examine the relationship between continuous CL and delivery GA.

**Study Design**: This was a retrospective cohort study of 1,000 patients who delivered at a single tertiary, urban academic hospital from January 2013‐August 2023 after transvaginal CL was measured at 16w0d‐24w6d GA. We abstracted delivery GA and CL taken closest to 20w0d from the electronic medical record. We used linear regression to model associations between a 1 mm increase in CL and mean change in GA at delivery for the full cohort as well as a sub‐cohort with CL < 25 mm. We used model r^2^ to measure the proportion of variance in GA explained by CL in each group.

**Results**: 1,038 pregnancies in 1,000 patients met inclusion criteria. 21.4% of pregnancies were in patients with prior sPTB (Table 1), consistent with a high‐risk population. 13.3% of index pregnancies ended in sPTB. CLs were normally distributed with a mean (standard deviation) of 38 (7) mm (Table 2). The median (min, max) delivery GA was 39.0 (21.0, 42.0) weeks. CL and GA were weakly positively correlated (Spearman r = 0.07, p = 0.02). In the overall cohort, 1 mm CL increase was associated with a mean increase of 0.5 (95% confidence interval (CI) 0.4‐0.7) days GA at delivery (r^2^ = 0.06). In the short cervix cohort, 1 mm increased CL was associated with increased GA of 2.7 (95% CI 0.9‐4.4) days (r^2^ = 0.25).

**Conclusion**: Continuous CL was positively associated with delivery GA in all patients, but small changes in CL explained more variation in GA at delivery in patients with CL < 25 mm. Because every additional day of gestation improves neonatal outcomes in sPTB, further research should investigate the use of continuous CL along with other predictors for risk stratification in high‐risk patients.

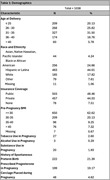

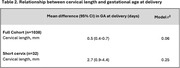

## 946 | Cesarean Section Rates and Maternity Populations at Risk: a Single Center 16 Year Follow Up

Maxim Shapiro^1^; Zvi Ehrlich^2^; Rivka Farkash^1^; Yael Lerner^1^; Ronit Calderon Margalioth^1^; Sorina Grisaru Granovsky^3^

*^1^SZMC, SZMC, Yerushalayim; ^2^Shaare Zedek Medical Center, Jerusalem, Yerushalayim; ^3^Hebrew University, Jerusalem, Yerushalayim*

10:30 AM ‐ 12:30 PM

**Objective**: To estimate the association between CS rates and the different maternity populations as classified Robson 10‐group Classification (RGC) at a single large maternity center with uniform clinical protocols, between 2005‐2021.

**Study Design**: 235,280 recorded births were categorized using RGC. CS risk was assessed by logistic regressions using both univariate and adjusted multivariate models. All models were re analyzed for parity groups in the RGC, nulliparous and multiparous.  Within the nulliparous groups, RG1 was a reference for RG 2a, 2b, 6 and 8‐10, and for multiparous, RG 3 was a reference for RG 4, 5, 7 and 8‐10).

**Results**: CS rate increased from 11.0% to 13.8% during the study period (Figure). The largest groups were nulliparous and multiparous women with single cephalic, spontaneous labor at term (RG1 and RG3, respectively); 71.1%. The group that contributed the most to overall CS rate was RG 5 (multiparous, single cephalic pregnancy at term with prior CS), 33.6% of CS. RG5 also had the highest risk among the multiparous analysis (aOR = 45.2, 95% CI: 42.5‐48.0). Apart from malpresentations, the groups with the highest risk for CS were RG8 (all multifetal pregnancies at any gestational age) overall and among nulliparous women (aOR = 6.1 95% CI: 5.4‐6.8, and aOR = 24.7 95% CI: 20.4‐29.8, respectively). Notably, labor induction increased the risk for CS in both nulliparous and multiparous (aOR = 3.5 95% CI: 3.5‐4.0, and aOR = 3.7 95% CI: 3.2‐3.9, respectively). (Table)

**Conclusion**: CS rates are still rising, even in populations with a basic low rate. Interventions to reduce CS rates should be focused on decreasing multifetal pregnancies rate in the setting of assisted reproduction and a careful revision of the limited trial of labor after cesarean. Labor induction was shown to increase the risk for CS in both nulliparous and multiparous groups, warranting inspection of this rising practice.

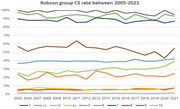

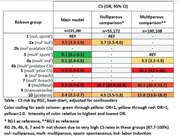

## 947 | Association between Robson 10 Group Classification (RGC) and the risk adverse maternal and neonatal outcomes

Maxim Shapiro^1^; Zvi Ehrlich^2^; Rivka Farkash^1^; Liav Berkovits^1^; Ronit Calderon Margalioth^1^; Sorina Grisaru Granovsky^3^

*^1^SZMC, SZMC, Yerushalayim; ^2^Shaare Zedek Medical Center, Jerusalem, Yerushalayim; ^3^Hebrew University, Jerusalem, Yerushalayim*

10:30 AM ‐ 12:30 PM

**Objective**: To analyze the association between the different maternity women groups classified by Robson 10 Group Classification (RGC) and the risk for  adverse maternal and neonatal outcomes, at a single large maternity center.

**Study Design**: An observational study including 235,280 births. Outcomes included post‐partum hemorrhage (PPH), puerperal fever, extended length of hospitalization (LOH ≥4 days), maternal readmission within 42 days, in‐hospital neonatal mortality, 5‐minutes APGAR score< 7 and NICU ≥3 days. The associations and risks were assessed by adjusted logistic regression models for the entire populations as well as by maternal age, background medical condition prior to delivery, GDM, hypertensive disorder during pregnancy, prior miscarriage, gestational age. Analysis was conducted separately by parity. For nulliparous groups, RG1 was a reference for RG 2a, 2b, 6 and 8‐10, and for multiparous, RG 3 was a reference for RG 4, 5, 7 and 8‐10). In these comparisons, groups 8‐10 were selected by their appropriate parity for each analysis.

**Results**: All multifetal pregnancies (RG8) had the higher risk for PPH (aOR = 1.7, 95% CI: 1.4‐2.0) and extended LOH (aOR = 4.7, 95% CI: 4.0‐5.5). RG 2b and 4b; Nulliparous and multiparous pre‐labor CS at term, respectively, as well as RG 6; nulliparous breech (unrelated to the mode of delivery), had the highest risk for neonatal mortality (aOR = 9.1, 8.0 and 8.0, 95% CI, respectively). Both RG2a and RG4a groups; nulliparous and multiparous single cephalic labor induction at term, markedly showed that labor induction was significantly associated with low 5' APGAR and neonatal mortality, both for nulliparous and multiparous (RG2a low APGAR aOR = 1.7 95% CI: 1.4‐2.0, mortality aOR = 4.2 95% CI: 2.6‐6.7.  RG4a‐ low APGAR aOR = 3.3 95% CI: 2.7‐3.9, mortality aOR = 7.7 95% CI: 6.0‐9.9) Tables 1 & 2.

**Conclusion**: The maternal and neonatal risk for adverse perinatal outcome is strongly associated with breech presentation, multifetal pregnancies at any gestational age and labor induction. Focused interventions to these groups may reduce the perinatal adverse outcomes in specific populations.